# Hypothesis-Driven, Structure-Based Design in Photopharmacology: The Case of eDHFR Inhibitors

**DOI:** 10.1021/acs.jmedchem.1c01962

**Published:** 2022-03-08

**Authors:** Piermichele Kobauri, Nicole S. Galenkamp, Albert M. Schulte, Jisk de Vries, Nadja A. Simeth, Giovanni Maglia, Sebastian Thallmair, Dušan Kolarski, Wiktor Szymanski, Ben L. Feringa

**Affiliations:** † Stratingh Institute for Chemistry, University of Groningen, Nijenborgh 4, 9747 AG Groningen, The Netherlands; ‡ Groningen Biomolecular Science and Biotechnology Institute, University of Groningen, Nijenborgh 4, 9747 AG Groningen, The Netherlands; § Institute for Organic and Biomolecular Chemistry, University of Goettingen, Tammannstr. 2, 37077 Göttingen, Germany; ∥ Groningen Biomolecular Sciences and Biotechnology Institute and Zernike Institute for Advanced Materials, University of Groningen, Nijenborgh 7, 9747 AG Groningen, The Netherlands; ⊥ Frankfurt Institute for Advanced Studies, Ruth-Moufang-Straße 1, 60438 Frankfurt am Main, Germany; # DWI-Leibniz Institut für interaktive Materialien e.V., RWTH Aachen University, Forckenbeckstraße 50, 52074 Aachen, Germany; ∇ Department of Radiology, Medical Imaging Center, University of Groningen, University Medical Center Groningen, Hanzeplein 1, 9713 GZ Groningen, The Netherlands

## Abstract

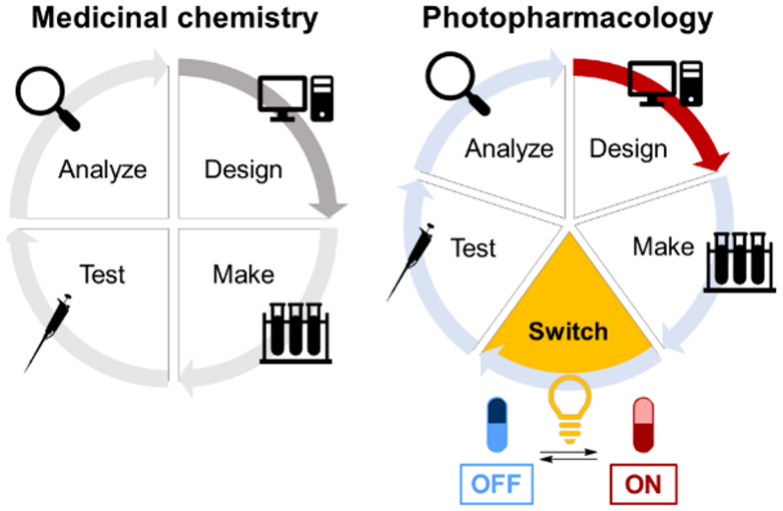

Photopharmacology uses light to regulate the biological activity of drugs. This precise control is obtained through the incorporation of molecular photoswitches into bioactive molecules. A major challenge for photopharmacology is the rational design of photoswitchable drugs that show light-induced activation. Computer-aided drug design is an attractive approach toward more effective, targeted design. Herein, we critically evaluated different structure-based approaches for photopharmacology with *Escherichia coli* dihydrofolate reductase (eDHFR) as a case study. Through the iterative examination of our hypotheses, we progressively tuned the design of azobenzene-based, photoswitchable eDHFR inhibitors in five design–make–switch–test–analyze cycles. Targeting a hydrophobic subpocket of the enzyme and a specific salt bridge only with the thermally metastable *cis*-isomer emerged as the most promising design strategy. We identified three inhibitors that could be activated upon irradiation and reached potencies in the low-nanomolar range. Above all, this systematic study provided valuable insights for future endeavors toward rational photopharmacology.

## Introduction

**Figure 1:**
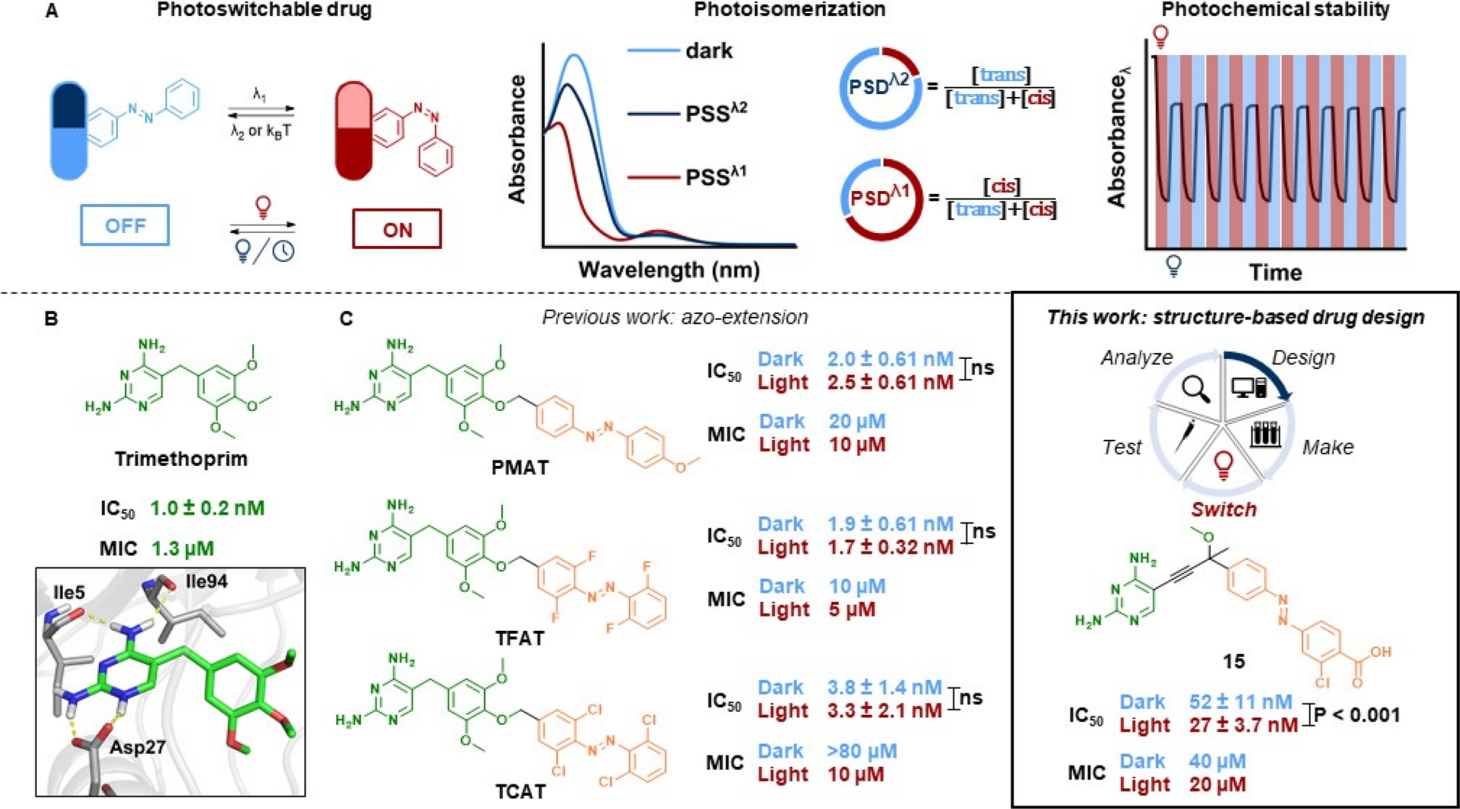
(A) Main features of a photoswitchable drug, employing azobenzene as a representative photoswitch. Irradiation with light at wavelength λ_1_ or λ_2_ induces *trans*-to-*cis* or subsequent *cis-*to-*trans* photoisomerization until a PSD is reached. The photochemical stability of the drug is analyzed via repeated cycles of irradiation. (B) Molecular structure and predicted binding mode of TMP to eDHFR (PDB ID: 3DAU).^13^ (C) While previous work^8^ applied the azo-extension strategy, the current study focuses on iterative structure-based drug design with a photopharmacological adaptation of the DMTA cycle. All IC_50_ values were obtained in this work (ns: not significant; *P*: *P* value). Extra sum-of-squares F test was used for statistical analysis.

Photopharmacology takes advantage of light-responsive molecular tools to achieve photocontrol over the potency of bioactive molecules, with the goal of improving the selectivity profile of drugs.^1−6^ A combination of suitable photochemistry and synthetic accessibility makes azobenzene currently the most commonly used photoswitch in photopharmacology.^5^ Upon irradiation with light of wavelength λ_1_, azobenzene undergoes photochemical isomerization from the thermally stable *trans*-isomer to the metastable *cis*-isomer (Figure 1A). The photoswitch then isomerizes back to the *trans*-isomer by thermal relaxation or by irradiation with light of wavelength λ_2_.^5^ The distinctive geometrical and electronic differences between the isomers are exploited for the regulation of the potency of the drug.^1−6^

Among other possible future uses, photopharmacology has appeared as an exciting, unconventional strategy to tackle poor selectivity in antibacterial therapies.^7−11^ Similarly to the majority of applications,^12^ the ideal scenario for light-controlled antibiotics would require low or no potency of the thermally stable isomer of the drug, while irradiation should result in its activation (“*cis*-on”, Figure 1A).^8^ Currently, one of the biggest challenges in photopharmacology is the rational, informed design of *cis*-on photoswitchable drugs with a large difference in potency between the isomers.

Due to the strongly interdisciplinary nature of the field, the molecular design of photopharmacological agents lies at the intersection of organic synthesis, photochemistry, chemical biology, and medicinal chemistry. The resulting challenges go beyond typical drug design: One has to design two forms, guided by the structures of known switches, and maximize their difference in potency, aiming at a higher activity of the metastable state. Concurrently, other properties need to be optimized (Figure 1A): the irradiation wavelengths that promote photoisomerization (toward the visible/NIR part of the electromagnetic spectrum for better tissue penetration and lower toxicity^12,14,15^); the photostationary state distribution (PSD), which quantifies the enrichment of a specific isomer under irradiation at a specific wavelength; and the photochemical stability and the thermal half-life of the metastable isomer.^12^ Finally, if the design comes from the modification of existing molecules, the alteration of the original pharmacological properties should be kept at a minimum. In a nutshell, photopharmacology needs to examine classical medicinal chemistry considerations^6^ through the lenses of photochemistry,^3,12^ and vice versa.

Going beyond trial-and-error approaches, rational design in photopharmacology can take two forms: azo-extension and azologization.^2^ In the first approach, an azobenzene is appended to the drug core after structure–activity relationships (SAR) evaluations. In the second approach, bioisosteres of azobenzene (“azosteres”) are identified and substituted.^16^ Nevertheless, these methods often lead to “*trans*-on” ligands^17^ as well as less predictable outcomes.

A partial solution for the rational design of *cis*-on photoswitchable drugs is provided by the azologization of *cisoid* azosteres, that is, moieties that are geometrically and electrostatically similar to *cis*-azobenzene:^18^ Azologization of *cis*-stilbene in combretastatin A-4,^19^*N*-methylbenzilaniline in methotrexate,^20,21^ benzophenone in a CRY1 activator,^22^ and biaryl sulfonamide in kinase, phospholipase and histone deacetylase inhibitors^18,23^ resulted in *cis-*on ligands. Very recently, the atypical azologization of an adamantyl group has been reported in the design of a photoswitchable cannabinoid receptor ligand.^24^ However, most drugs do not feature *cisoid* substructures that can be substituted, thus highlighting the need for a strategy with a broader utility.

A more general approach is the application of structure-based drug design, which is a mature technique in drug discovery.^25,26^ This method would broaden the scope of rational design in photopharmacology, because it can be applied not only to targets with resolved structures but also to other targets via homology modeling.^27^ Such an approach has been gaining momentum also in photopharmacology in recent years, resulting in different applications for modeling: computer-assisted design^18,22,28^ and *a posteriori* rationalization of the observed results.^29−32^ However, proper structure-based design^33,34^ (analysis of substitutions, reiterated cycles of design-test) is still an underexplored pathway in small-molecule-based approaches to regulating biological activity with light.

Here, we compared and evaluated different strategies for rational design in photopharmacology taking *Escherichia coli* dihydrofolate reductase (eDHFR), a relevant drug target for antibiotics, as a model case. Starting from the concept of design–make–test–analyze (DMTA) cycles^35^ common in early stage drug discovery efforts, we conducted several cycles of iterative rational design, synthesis, photochemical characterization, biological evaluation, and thorough analysis of results (design–make–switch–test–analyze, DMSTA). At every stage, the main goal was the design of *cis*-on photoswitchable antibiotics with enhanced difference in potency between the isomers.

## Results and Discussion

### Target and Approach

The ubiquitous enzyme dihydrofolate reductase (DHFR) uses NADPH as a cofactor to catalyze the reduction of dihydrofolate to tetrahydrofolate, which is an important cofactor for the biosynthesis of DNA bases and amino acids. The inhibition of this enzyme blocks cell division, synthesis, and repair of DNA/RNA and protein synthesis in rapidly proliferating cells of different organisms.^36^ Widely used DHFR inhibitors include methotrexate, a long-standing chemotherapeutic agent that targets human DHFR,^37^ and trimethoprim (TMP, Figure 1B), a broad-spectrum antimicrobial mainly used to treat urinary tract infections.^38^ DHFR is a highly suitable target for structure-based drug design, because it is a small (∼18 kDa), well-characterized enzyme with >6000 deposited PDB structures. For example, the DHFR inhibitor iclaprim, currently under clinical trials,^39^ was discovered through computer-aided design.^40^ Moreover, modulating DHFR activity with light has seen promising results in previous studies.^8,20,21^

In pioneering efforts toward light-controlled antimicrobial agents, our group developed three photoswitchable TMP conjugates that displayed from 2- to 8-fold differences in antibacterial activity (Figure 1C).^8^ Since these compounds had been tested only for their minimal inhibitory concentration (MIC) values, we characterized their inhibition of eDHFR as a first step for the rational improvement of the design. Computational studies suggested that the different isomers would bind to the enzyme but with no relevant differences (Figures S81–S83). The azobenzene part of the inhibitors was predicted to be highly solvent-exposed and flexible, thus providing only transient, nonspecific interactions. These predicted properties were confirmed by inhibition assays showing that the compounds were indeed potent eDHFR inhibitors, although no significant differences between the photoisomers were observed (Supporting Information (SI), Section S3.2). These initial results are in accordance with the known tolerance of long linkers in the *para* position of the trimethoxyphenyl ring of TMP.^41^ Furthermore, they indicate that the observed differences in antibacterial activities might arise from other coexisting mechanisms (e.g., different accumulation in bacteria), as recently suggested by our follow-up study of resistance development against the best candidate.^42^ Furthermore, a cheminformatics analysis of key physicochemical parameters revealed that these TMP derivatives have molecular weights and calculated log *P*s outside of the drug-like ranges (SI, Section S5.3). Overall, despite being responsive to visible light, these previously designed compounds still offer major room for improvement and are an attractive starting point for structure-based design in photopharmacology.

**Scheme 1:**
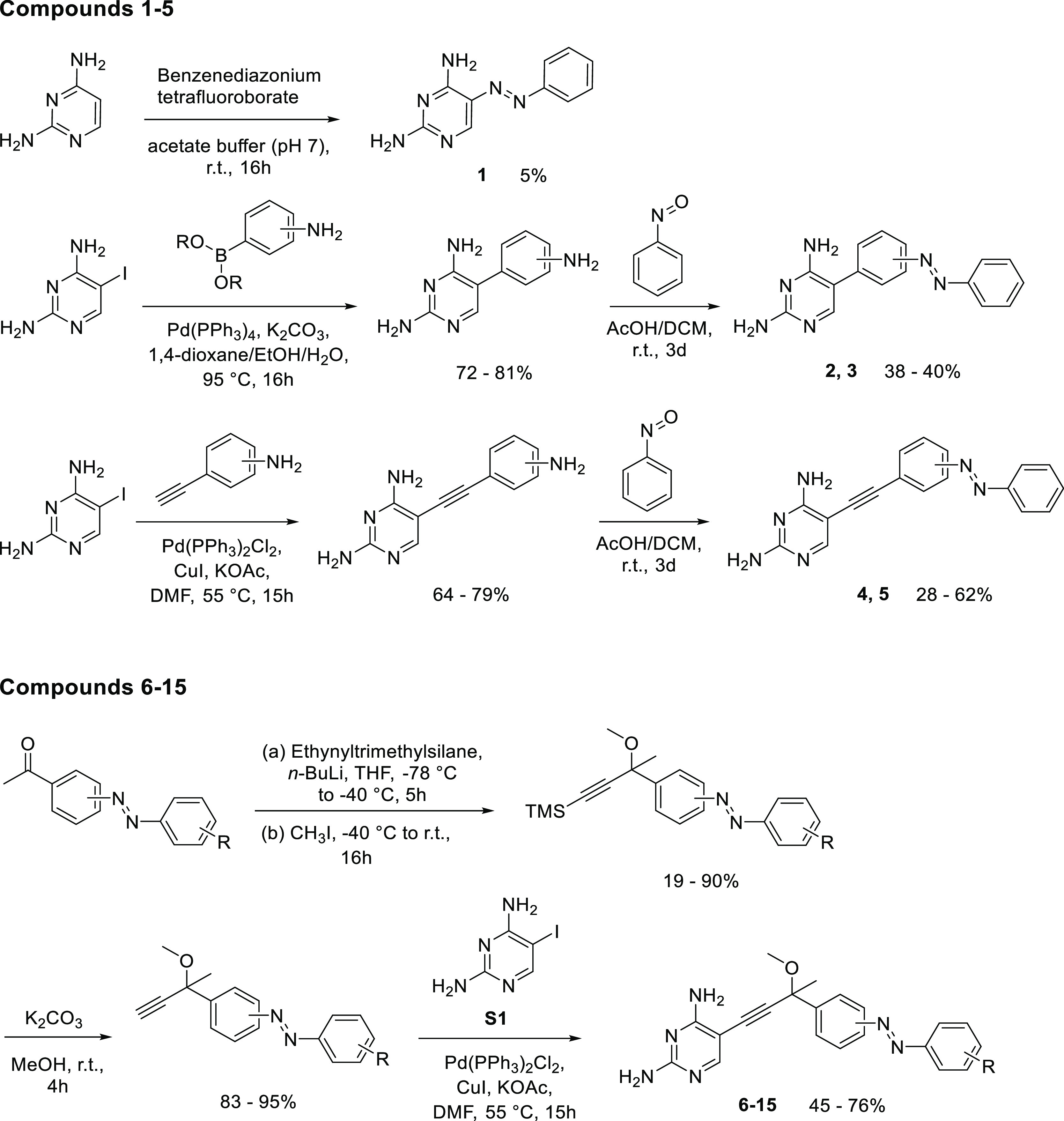
General Synthetic Strategies for Compounds **1**–**15** For complete synthetic details (numbers for intermediate compounds, R substituents) see SI, section S1.

The focus of our current work is the hypothesis-driven design of photoswitchable eDHFR inhibitors, through structure- and photochemistry-based, rational strategies for hit discovery and hit-to-lead optimization (Figure 1C). Each cycle started with computer-assisted design, namely performing induced fit docking^43^ (IFD) to incorporate some enzyme flexibility, followed by three replicas of 100 ns molecular dynamics (MD) simulations. The IFD results were visually inspected in detail, while docking scores were not considered in our analysis. Despite their popularity, docking scores have been optimized to discriminate only between binding and nonbinding compounds in vast libraries, and they should always be supported by other selection criteria (e.g., ligand strain, interactions with key residues).^44^ Docking scores are rough and inaccurate estimations of binding affinities,^45^ and it has been suggested that visual inspection of docking poses can outperform simple evaluations of docking scores.^46^ Moreover, our computational protocol enabled us to assess ligand stability (root-mean-square deviation, RMSD) and the persistence of specific interactions over time^47^ as well as to compute useful descriptors such as solvent accessible surface area (SASA).^48^ Second, the compounds were synthesized and fully characterized (^1^H NMR, ^13^C NMR, HRMS, Scheme 1 and SI). Next, UV–vis and ^1^H NMR spectroscopies were used to investigate their photochemical properties in DMSO and eDHFR assay buffer, that is, optimal photoisomerization wavelengths λ_1_ and λ_2_, PSD, and fatigue upon multiple photoisomerization cycles in the presence of the reducing agent glutathione (GSH). Additionally, the thermal half-life of the metastable isomer was determined in DMSO and aqueous buffer at 25 and 37 °C. The key hypothesis behind each approach was then evaluated by *in vitro* and *in cellulo* pharmacological characterization of eDHFR inhibition and antibacterial activity against *Escherichia coli* mutant strain CS1562. Finally, we analyzed computational and experimental data to generate insights and inspire the working hypothesis for the next step in the iterative cycle.

### Hit Discovery: Ensuring the Hydrogen-Bond Network only in the *cis* State

**Figure 2:**
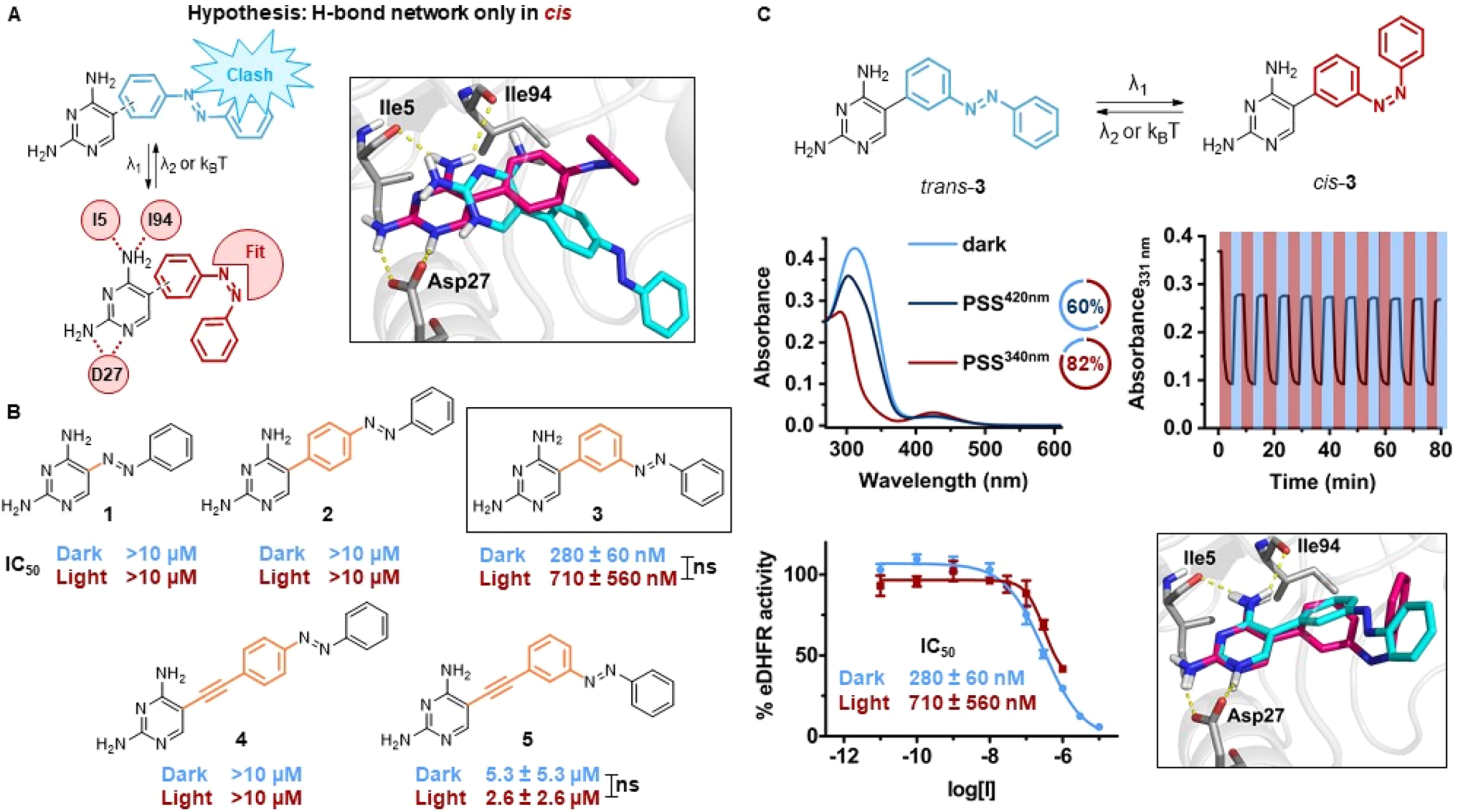
First design. (A) Design hypothesis: The diaminopyrimidine head can form its hydrogen-bond network with the DHFR protein (yellow dashed lines) only upon photoisomerization. The 3D visualization is obtained from IFD poses of *trans*- (cyan) and *cis*-**4** (magenta) into eDHFR (PDB ID: 3DAU). (B) Overview of molecular structures (linker shown in orange) and IC_50_ values of inhibitors **1**–**5**. (C) Photochemical and pharmacological evaluation of compound **3**. Top left: UV–vis spectra (20 μM, 1% DMSO in eDHFR assay buffer) at the thermal equilibrium (light blue), PSS^420nm^ (dark blue) and PSS^340nm^ (red). Doughnut charts of the respective PSDs in DMSO-*d*_6_ (2 mM). Top right: Repeated photoisomerization with λ = 340 and 420 nm light (20 μM, 1% DMSO in eDHFR assay buffer, 10 mM GSH). Bottom left: Dose–response curves against eDHFR before and after irradiation with λ = 340 nm. Bottom right: IFD poses of *trans*- (cyan) and *cis*-**3** (magenta) into eDHFR.

It has been argued that developing a photoswitchable ligand starting from a photoswitchable hit might lead to larger differences in activity, in comparison to the attachment of a photoswitch in peripheral regions of an optimized ligand.^6^ In particular, the inclusion of a photoresponsive unit in the pharmacophore, that is, an intrinsic photoswitch, should have more critical effects on binding.^18^ We tested this design principle by incorporating the azobenzene in a crucial position, that is, very close to the 2,4-diaminopyrimidine head (Figure 2). The hydrogen bonds between this ring, Asp27, and the peptide backbone carbonyl groups of Ile5 and Ile94 of eDHFR mimic the enzyme–substrate interactions^49^ and are essential for the molecular recognition of TMP (Figure 1B; docking poses in agreement with the recently published structure of a TMP-eDHFR complex,^49^ see Figure S78). As an example, photocaging one of the amino groups proved to be an effective strategy for irreversible photocontrol of TMP binding to eDHFR.^41^ Considering the angle of the methylene bridge in TMP, the introduction of a linear and stiff *trans*-azobenzene should cause steric clashes of the ligand with the enzyme and the disruption of the above-mentioned hydrogen-bond network (Figure 2A). On the other hand, the bent geometry of *cis*-azobenzene would allow a better accommodation of the pyrimidine head and result in higher affinities. A related principle has been successfully applied to the azologization of methotrexate both in bacterial^20^ and human DHFR.^21^

To test this hypothesis, we screened linkers of different lengths (no linker, phenyl and phenylacetylene linker)^50^ between the pharmacophore and the azobenzene (Figure 2B). For synthetic accessibility, we did not introduce substituents at the azobenzene moiety at this stage. The decision was supported by a previous report, which showed that a TMP analogue with an unsubstituted outer ring lost some potency, yet still retained low nanomolar activity (IC_50_ values ranging from 2 to 160 nM).^51^

Starting from TMP, the most straightforward design was the substitution of the methylene bridge with an azo bond. Here, the photoswitching unit was included directly in the pharmacophore. Modeling studies suggested that both isomers of compound **1** could fit the binding pocket, because of their small structural change (Figure S86). In the second design, we increased the distance between the azobenzene and the pyrimidine head with a phenyl linker (resembling pyrimethamine), exploring both *para* and *meta* substitution patterns. IFD and MD calculations indicated that compound **2** would engage in a hydrogen-bond network only in the *cis* form (Figure S87), while compound **3** showed negligible differences in predicted binding behaviors (Figures 2C and S88). Finally, we further extended the linear region with a longer phenylacetylene linker. Related structures have only been studied as inhibitors of protozoal DHFR,^50^ thus we decided to explore the activity of this linker on eDHFR. Both *meta*- and *para*-substituted azobenzene were computationally predicted to fit, with negligible differences between the isomers.

Compound **1** was obtained in one step by a challenging azo coupling, albeit with very low yields, adapting a published procedure (Schemes 1 and S1).^52^ For the single-bond and the acetylenic series, iodination of 2,4-diaminopyrimidine at the position 5 provided a common intermediate (compound **S1**) that could be subjected to Suzuki or Sonogashira cross-coupling, respectively (Scheme 1; for the synthesis of all intermediates and final compounds, see the Experimental Section and SI, Section S1). Next, compounds **1**–**5** were photochemically characterized (Figures S1–20). As an illustrative example, photoisomerization of **3** was achieved with λ_1_ = 340 nm and λ_2_ = 420 nm in eDHFR assay buffer (Figure 2C), reaching a PSD in DMSO-*d*_6_ of 82% *cis* and 60% *trans*, respectively. The compound showed no sign of fatigue after repeated cycles of irradiation in the presence of 10 mM GSH, which represents the reducing environment that is typically found in cells (Figure 2C).^53^

To determine their inhibitory potency, we performed a colorimetric assay on compounds **1**–**5** in their dark-adapted and irradiated states (see Experimental Section and SI for details). Compounds **1**, **2**, and **4** did not show considerable activity up to 10 μM (Figure 2B). The azopyrimidine derivative **1** was inactive, probably due to the lack of methoxy substituents on the outer ring, in addition to the fact that the overall structure of the TMP has been modified by replacing the methylene bridge with an azo bond. However, the *cis*-isomer of this compound showed an extremely short lifetime (*t*_1/2_ = 10 s in eDHFR buffer at 25 °C, Figure S3), which would hinder any applications. Molecules **2** and **4** appeared to be too elongated and stiff to bind in either isomer form, although **2** had emerged as the most promising design from IFD calculations (Figure S87). On the other hand, derivatives bearing a *meta*-substituted azobenzene inhibited eDHFR with no significant difference between the two forms. In particular, we observed that compound **5**, which featured the longer phenylacetylene linker, showed low inhibition (dark IC_50_ = 5.3 μM and light IC_50_ = 2.6 μM), while compound **3** with the phenyl spacer showed satisfactory activities (dark IC_50_ = 280 nM and light IC_50_ = 710 nM, Figure 2C).

The bioactivity data indicated that derivatives with a phenylacetylene linker had lower potency. A re-examination of the MD simulations provided another possible explanation for the discrepancies between computational predictions and experimental results. To engage in the key hydrogen bonds with Asp27, Ile5, and Ile94, the alkyne linker of both isomers of compounds **4** and **5** had to deviate from its flat, linear geometry throughout the simulations (Figures S90 and S92). Such unusual torsional strains are known to lead to lower binding affinities.^54^

Nevertheless, the isomer-specific formation of the hydrogen bonds between the 2,4-diaminopyrimidine head and Asp27, Ile5, and Ile94 was not a sufficiently high driving force for a large difference in potency between the isomers. Our photoswitchable inhibitors had similar affinities in both forms: either none of them displayed binding or both did. We also verified the binding contribution of the 2,4-diaminopyrimidine head, which fell in the range of activity for small fragments (IC_50_ = 0.36 mM, see SI, Section S3.2). The design approach investigated here appears to give better results when applied to larger structures, as in the case of the azologization of methotrexate.^20,21^ When the photoswitching unit is incorporated in the middle of an extended structure, the geometrical change is more substantial. In contrast, TMP is much smaller and proved not to be suitable. Moreover, we did not apply azologization strictly, but rather aimed at the binding modulation of the TMP pharmacophore. As a result, it turned out to be extremely challenging to pursue *de novo* photopharmacology, that is, to find a structurally novel photoswitchable hit compound and build up activity from there. However, the results presented in this section also provided a key insight, insofar that the space in the *meta* position needs to be explored with the next design hypothesis.

### Hit Discovery: *meta*-Substituted Biaryl Scaffolds as *cisoid* Azosteres

**Figure 3:**
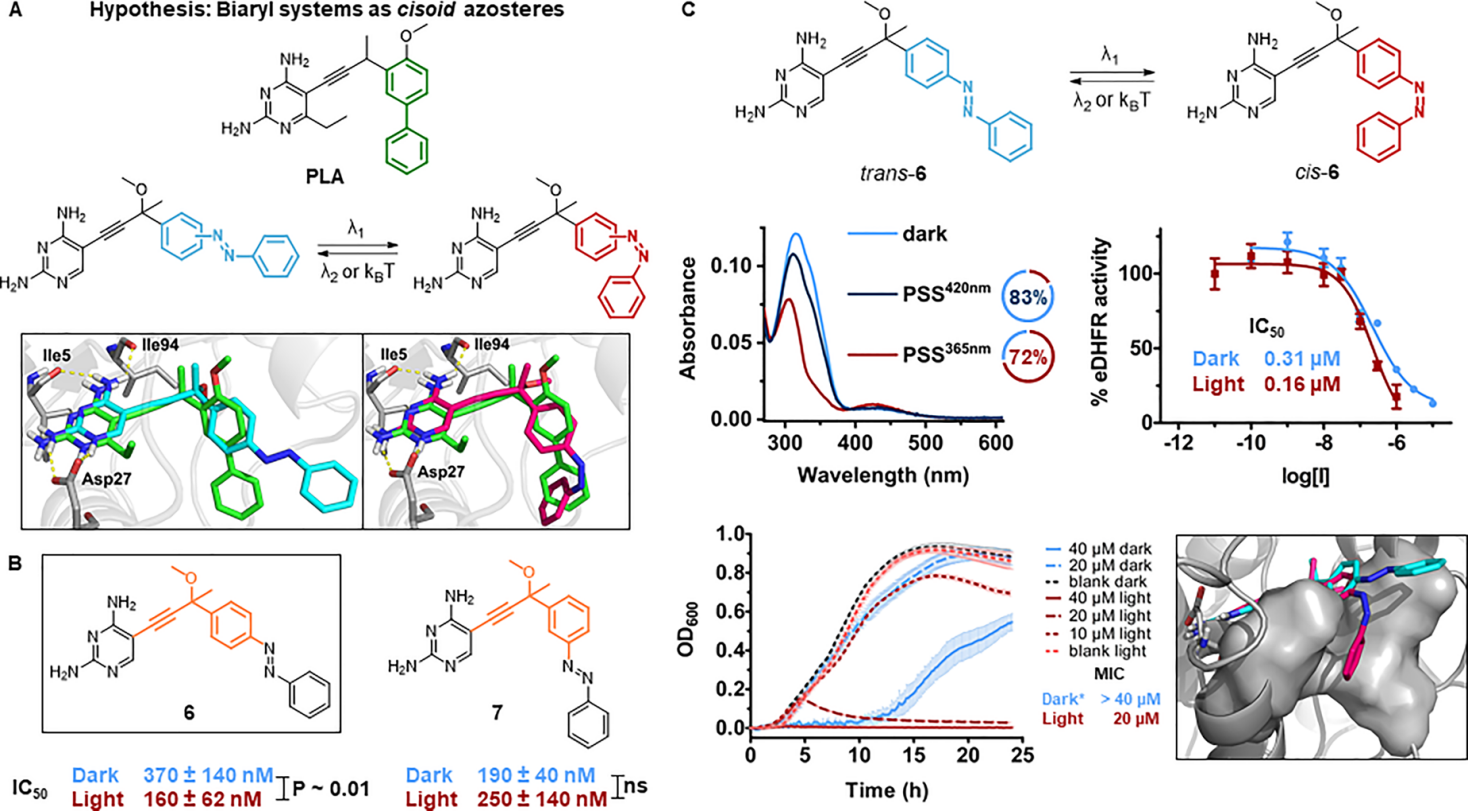
Second design. (A) Design hypothesis: Azologization of the biaryl system in PLA can give a *cis*-on inhibitor. The 3D visualization is obtained from IFD poses of PLA (green), *trans*- (cyan) and *cis*-**6** (magenta) into eDHFR. (B) Overview of molecular structures (linker shown in orange) and IC_50_ values of inhibitors **6** and **7**. (C) Photochemical and pharmacological evaluation of **6**. Top left: UV–vis spectra (5 μM, 20% DMSO in eDHFR assay buffer) at the thermal equilibrium (light blue), PSS^420nm^ (dark blue), and PSS^365nm^ (red). Doughnut charts of the respective PSDs in DMSO-*d*_6_ (2 mM). Top right: Dose–response curves against eDHFR before and after irradiation with λ = 365 nm. Bottom left: Bacterial growth curves of *E. coli* CS1562 at different concentrations of **6** before and after irradiation with λ = 365 nm. (* = The concentration range was limited by solubility in LB medium.) Bottom right: IFD poses of *trans*- (cyan) and *cis*-**6** (magenta) into eDHFR (hydrophobic subpocket as gray surface).

Inspired by the structure of propargyl-linked antifolates (PLAs) with high potency against eDHFR,^55^ we envisioned that the bent geometry of this linker could exploit the available space. We hypothesized that *meta*-substituted biaryl systems are *cisoid* azosteres for *para*-substituted azobenzenes (Figure 3A), in agreement with one reported example.^56^ To test this hypothesis, we designed two photoswitchable inhibitors with both *para*- and *meta*-substituted azobenzene (Figure 3B). For synthetic convenience, we simplified the scaffold by removing the ethyl chain on the pyrimidine ring. In addition, the design included one methoxy group, which was predicted to occupy a similar region of the binding pocket as the methoxy groups of TMP do (Figure S93). Methyl substitution at the benzylic position of these antifolates was shown to be tolerated, and the position of the proximal methoxy group could also be changed, retaining the high potency.^55^ These considerations supported our design featuring a disubstituted propargyl linker, which was necessary for synthetic reasons to avoid the formation of allene side-product in the deprotection of the trimethylsilyl alkyne.

The alkynyl moiety was introduced via the addition of lithium acetylide to an acetyl group, followed by quenching with methyl iodide (Schemes 1 and S2). Subsequently, Sonogashira coupling with the iodopyrimidine intermediate **S1** yielded the target compounds. Although the synthesis route created a stereogenic center, the racemic mixture was not resolved because the methyl configuration at this benzylic position was previously shown to have limited influence on eDHFR inhibition.^55^

Photochemical isomerization of compounds **6** and **7** was investigated in eDHFR assay buffer, and, in spite of decreased solubility (limited to 5 μM with 20% DMSO as an additive), they revealed robust photoswitching in the presence of 10 mM GSH (Figures 3C and S26). Also, the thermal lifetimes were long enough for the biological testing (*t*_1/2_ > 24h at 25 and 37 °C, Figure S23 and S27). However, it must be noted that compound **6** displayed a PSD^365nm^ of 72% *cis*, and hence, the irradiated sample contains significant amounts of the *trans*-isomer (Figure 3C).

Both derivatives **6** and **7** achieved higher potencies than the compounds **1**–**5** studied in the first approach cycle. Here, the propargyl linker has a more favorable geometry than the phenylacetylene linker (Figures S94 and S98), because it derives from an optimized structure.^55^ Supporting our hypothesis, the photocontrol of inhibition caused opposite effects depending on the substitution pattern on the azobenzene. UV irradiation with λ_1_ = 365 nm resulted in a promising 2-fold activation of compound **6**, toggling between a dark IC_50_ of 370 nM to an irradiated IC_50_ of 160 nM, while the light-induced changes in potency were not significant for compound **7**. These observations suggest that *meta*-substituted biaryl systems are better mimicked by *para*-substituted *cis*-azobenzenes. On the other hand, the *meta*-substituted *trans*-azobenzene of compound **7** perfectly overlaps with the biaryl moiety of **PLA** (Figure S97).

Since compounds **6** and **7** displayed promising eDHFR inhibition, we determined their antibacterial activities against *E. coli* CS1562 before and after irradiation with λ_1_ = 365 nm. Compound **6** maintained its *cis*-on activity, showing a > 2-fold difference in bacterial growth inhibition (Figure 3C), with a MIC > 40 μM in the dark (the concentration range was limited by solubility in LB medium) and 20 μM after irradiation. Instead, compound **7** inhibited bacterial growth equally in both forms, as its MIC of 20 μM was not affected by the photoisomerization (Section S4).

Since eDHFR inhibitor **6** displayed the desired *cis*-on behavior with promising enzymatic and antibacterial activity, we selected it as our photopharmacological hit for further optimization studies. Our findings at the end of the hit discovery stage reinforce the common assumption in photopharmacology that greater chances of success are associated with the rational modification of optimized bioactive structures. Nevertheless, *de novo* design of a photoswitchable hit remains an underexplored approach that holds great potential in spite of its intrinsic challenges.^1−4,6^

Analysis of biological and computational data suggested a correlation between the binding affinity of the isomers and their solvent exposure. When the lipophilic outer ring of azobenzene was solvent-exposed, the isomer showed weaker inhibition. Conversely, we observed an increase in potency to the submicromolar range when that outer ring formed van der Waals interactions with the lipophilic subpocket formed by Phe31, Leu28, Leu54, and Ile50 (Figures 3C, S95, S96, S99, and S100). This enzyme region has been targeted by the cyclopropyl group of iclaprim (Figures S79 and 80).^40^ Shape complementarity with these hydrophobic residues seems to drive the encouraging photoinduced changes, which were obtained notwithstanding the nonquantitative photoisomerization of compound **6** upon irradiation with λ_1_ = 365 nm. Therefore, we aimed to address this weakness in the next design round.

### Hit-to-Lead: *Cis*-Enriched PSD to Enhance Activation

**Figure 4:**
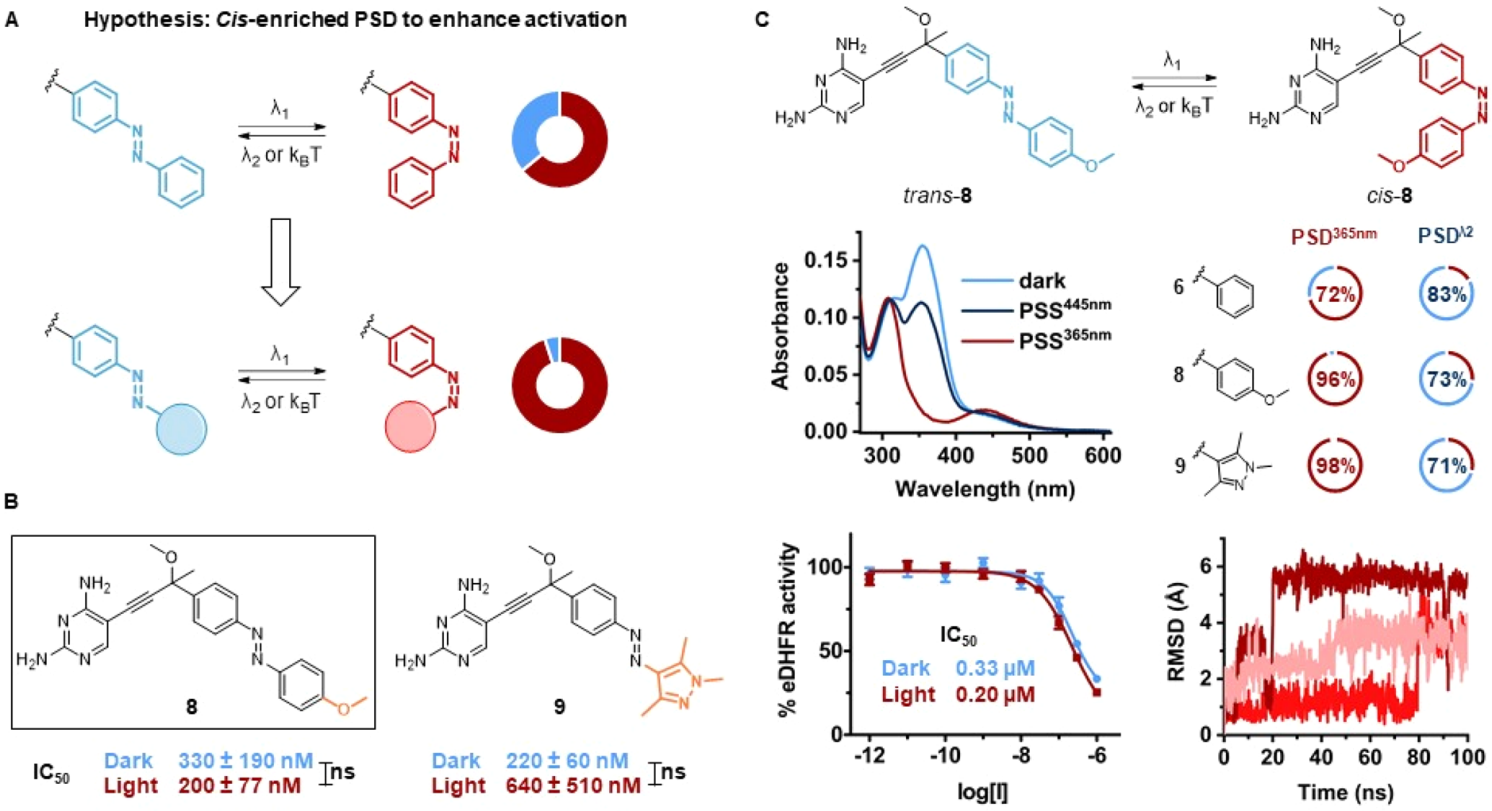
Third design. (A) Design hypothesis: Increasing PSD can boost the activation effect of irradiation. (B) Overview of molecular structures (photochemistry-driven modifications shown in orange) and IC_50_ values of inhibitors **8** and **9**. (C) Photochemical and pharmacological evaluation of **8**. Top left: UV–vis spectra (5 μM, 50% DMSO in eDHFR assay buffer) at the thermal equilibrium (light blue), PSS^445nm^ (dark blue), and PSS^365nm^ (red). Top right: Doughnut charts of PSDs in DMSO-*d*_6_ (2 mM). λ_2_ = 420 nm for **6** and λ_2_ = 445 nm for **8** and **9**. Bottom left: Dose–response curves against eDHFR before and after irradiation with λ = 365 nm. Bottom right: Ligand RMSD from three replicas of 100 ns MD simulations of the protein–ligand complex.

As mentioned earlier, photopharmacological design combines elements of medicinal chemistry and photochemistry. The identification of the *cis*-on hit **6** prompted us to optimize it by improving its photochemical properties.^12^ Irradiation of this compound with λ_1_ = 365 nm light resulted in a 2-fold gain in activity, although it only generated a PSD of 72% *cis*. Thus, we reasoned that a higher PSD could result in a more effective activation by light (Figure 4A). Two strategies established in our earlier studies to increase the population of the metastable isomer are the introduction of a *para*-methoxy substituent^57,58^ and the replacement of azobenzene with azopyrazole (Figure 4B), which is known to give quantitative photoswitching in both directions^59,60^ as well as increased solubility.^61,62^ Although azobenzene and azopyrazole have clear differences in shape and electrostatics, nitrogen-containing aromatic heterocycles (pyridine, imidazole) were shown to be tolerated in previous SAR studies.^55^

To complicate further the intricate relationship between drug design and photochemical optimization in photopharmacology, it should be mentioned that the *para*-methoxy group^63^ and the trimethylpyrazole moiety^64^ are hydrogen-bond acceptors that could potentially engage in additional hydrogen bonds in the *cis*-isomer, when pointing toward the binding pocket. However, MD simulations suggested that they would not be able to form persistent interactions. In more detail, we observed a high RMSD for the whole ligands, along with a large RMS fluctuation (RMSF) of the ether oxygen and the pyrazole nitrogen (Figures S103 and S105).

In agreement with our expectations, the structural modifications improved the PSDs of the series from 72% observed for compound **6** to > 95% recorded for compounds **8** and **9** (Figure 4C). However, an additional decrease in solubility for compound **8** required us to study its photoswitching behavior with a 5 μM solution in eDHFR assay buffer with 50% DMSO as an additive (Figures 4C and S29–S32). On the other hand, the introduction of an azopyrazole resulted in better aqueous solubility for compound **9** (Figures S33–S36).

The biological activity results, however, did not support our design hypothesis, as we found that a *cis*-enriched PSD was not enough to promote a boost in eDHFR inhibition. A subtle drop in potency was observed for derivative **8**, whereas the azopyrazole analogue **9** could be deactivated upon irradiation, thereby additionally losing the desired *cis*-on character (although the differences in IC_50_ were not significant, see Figure 4B,C and SI Section S3.2). When evaluated for their antibacterial properties, compounds **8** and **9** showed lower potency against *E. coli* CS1562 than the parent compound **6**. While **8** was completely inactive up to 80 μM, **9** did not display any light-dependent potency difference at the tested concentrations (Figure S70).

In an effort to rationalize the results and conceive the next hypothesis, we reconsidered the MD simulations data. Both ligands showed high flexibility in the binding pocket with a RMSD > 4 Å, a structural feature which has been linked to lower inhibitory activities.^65^ This mobility highlights the need to shift the focus to isomer-specific stronger interactions that could lock the *cis* form in the active site of eDHFR.

### Hit-to-Lead: Salt Bridge Formed only by the *cis* Isomer

**Figure 5:**
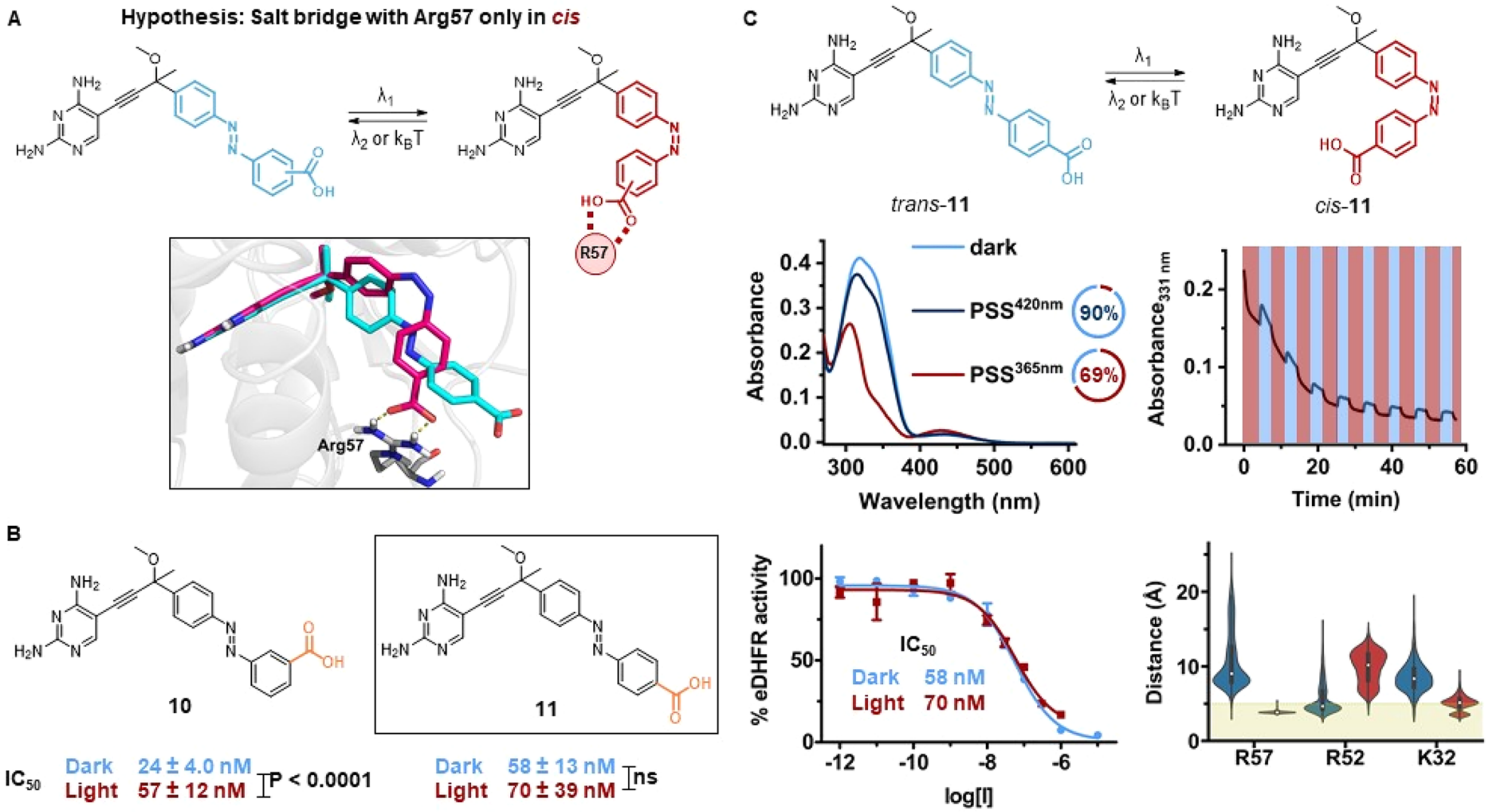
Fourth design. (A) Design hypothesis: The salt bridge between carboxylate and Arg57 (yellow dashed lines) can be formed only in the *cis* form. The 3D visualization is obtained from IFD poses of *trans*- (cyan) and *cis*-**10** (magenta) into eDHFR. (B) Overview of molecular structures (exploration of regioisomers shown in orange) and IC_50_ values of inhibitors **10** and **11**. (C) Photochemical and pharmacological evaluation of **11**. Top left: UV–vis spectra (20 μM, 5% DMSO in eDHFR assay buffer) at the thermal equilibrium (light blue), PSS^420nm^ (dark blue), and PSS^365nm^ (red). Doughnut charts of the respective PSDs in DMSO-*d*_6_ (2 mM). Top right: Repeated photoisomerization with λ = 365 and 420 nm light (20 μM, 5% DMSO in eDHFR assay buffer, 10 mM GSH). Bottom left: Dose–response curves against eDHFR before and after irradiation with λ = 365 nm. Bottom right: Violin plots of the distance between carboxylate and selected eDHFR residues for *trans*- (blue) and *cis*-**11** (red) throughout three replicas of 100 ns MD simulations (5 Å threshold as green surface).

After scrutinizing the residues in the binding pocket, we decided to seek a salt bridge with Arg57, by introducing a carboxylic acid moiety into the structure of the photoswitchable inhibitors, in a position that would enable only the *cis* isomer to form the salt bridge (Figure 5A). This specific interaction is found in the binding modes of the endogenous ligand dihydrofolate and of methotrexate and has been exploited also by some PLAs.^66^ Moreover, using one isomer to target a charge-assisted hydrogen bond between a carboxylic acid and an arginine has been recently implemented in photopharmacology.^67^ However, targeting a particular interaction with one of the two isomers is still an underexplored approach for reversible photopharmacology.^24,68^ We designed two compounds, exploring both *meta* and *para* substitution on the outer benzene ring (Figure 5B). We first evaluated our hypothesis computationally, and IFD poses and subsequent MD simulations suggested that compound **11** could engage in the desired salt bridge only in the case of the *cis*-isomer (Figure 5A). On the other hand, derivative **10** appeared able to interact with Arg57 with both isomers (Figures S106–S108), thus indicating that the *cis*-on activity might be less probable.

The photochemical characterization of compounds **10** and **11** was performed with smaller amounts of DMSO in eDHFR buffer, because the ionizable carboxylic acid improved the aqueous solubility of the compounds. This enhancement came at the cost of the resistance to reduction, especially for **11** (Figure 5C).

Gratifyingly, compounds **10** and **11** were determined to be potent eDHFR inhibitors, with IC_50_ values in the 2-digit nanomolar range (Figure 5B). Compound **10** exhibited a significant deactivation upon irradiation (dark IC_50_ of 24 nM against an irradiated IC_50_ of 57 nM), in line with the modeling observations. On the other hand, the difference in activity between the isomers of **11** was not significant, making this potent derivative more attractive for further fine-tuning toward a *cis*-on behavior.

The *in vitro* light-dependent potency that was observed for compound **10** did not translate into a photocontrolled antibacterial activity: Both forms completely inhibited bacterial growth at 20 μM (SI Section S4). Compound **11**, on the contrary, showed a similar inhibition pattern in the antibacterial assay, with a MIC value of 40 μM both before and after irradiation with λ_1_ = 365 nm light.

The biological evaluation of the “interaction in *cis*” hypothesis revealed that the differences in potency were not as we predicted by monitoring only the salt bridge with Arg57. Re-examination of the MD trajectories suggested that an explanation might be the simultaneous salt bridges formation of **10** and **11** with Arg52 and, to a lesser extent, Lys32 (Figures 5C and S108). It is likely that these undesired interactions are quite weak because those residues are more solvent-exposed than Arg57 (Figures S110 and S116), while only buried salt bridges contribute significantly to ligand affinities.^69^ They are also more flexible (Figures S109 and S115), hence a higher entropy cost is associated with the engagement in these interactions. However, these additional contributions might be responsible for the unexpected light-controlled behavior, together with the neglected desolvation costs of the carboxyl acid moieties. In fact, the energetic gain of a salt bridge is often compensated by desolvation penalties, which can cancel out the increase in affinity of charge-assisted hydrogen bonds.^69^ In our case, we decided to keep the carboxylic acid in the next design hypothesis because it provided a notable boost in activity.

### Hit-to-Lead: Increased Hydrophobic Contacts in the *cis* Form

**Figure 6:**
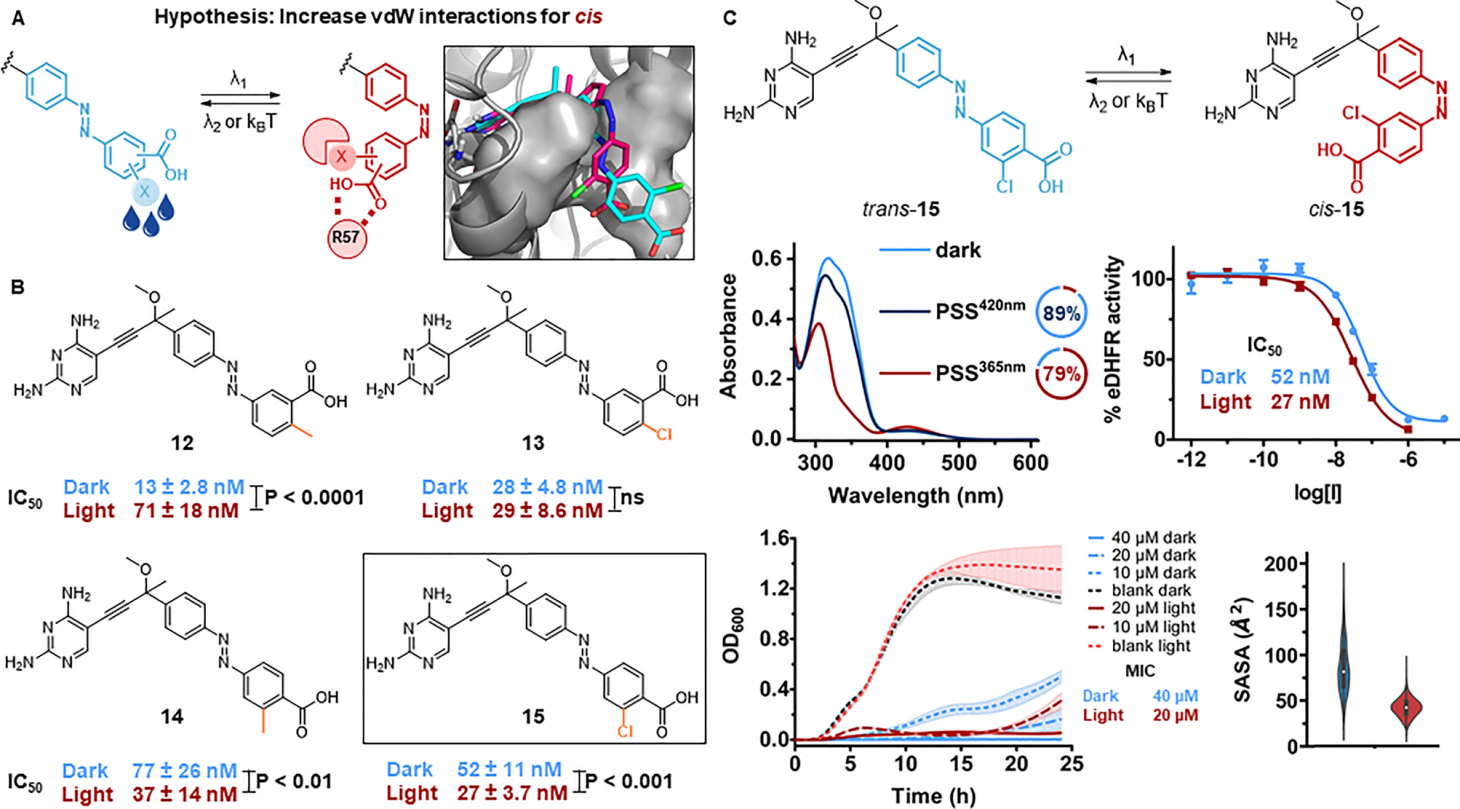
Fifth design. (A) Design hypothesis: Additional hydrophobic contacts can be formed only with the *cis* isomer. The 3D visualization is obtained from IFD poses of *trans*- (cyan) and *cis*-**15** (magenta) into eDHFR (hydrophobic subpocket as gray surface). (B) Overview of molecular structures (exploration of regioisomers shown in orange) and IC_50_ values of inhibitors **12**–**15**. (C) Photochemical and pharmacological evaluation of **15**. Top left: UV–vis spectra (20 μM, 1% DMSO in eDHFR assay buffer) at the thermal equilibrium (light blue), PSS^420nm^ (dark blue), and PSS^365nm^ (red). Doughnut charts of the respective PSDs in DMSO-*d*_6_ (2 mM). Top right: Dose–response curves against eDHFR before and after irradiation with λ = 365 nm. Bottom left: Bacterial growth curves of *E. coli* CS1562 at different concentrations of **15** before and after irradiation with λ = 365 nm. Bottom right: Violin plots of the SASA of the lipophilic subpocket for *trans*- (blue) and *cis*-**15** (red) throughout three replicas of 100 ns MD simulations.

To conceive the final structure-based hypothesis, we chose to merge the knowledge that was obtained from the design ideas that gave the best outcomes. In the second hypothesis/stage, hydrophobic interactions with the lipophilic subpocket emerged as the driving force for the *cis*-on inhibition, even though the ligands displayed high flexibility during MD studies due to the solvent-exposed binding pocket. Evaluating the fourth hypothesis revealed that the targeted salt bridge enhanced the potencies of the new series, along with locking the outer ring in its bound conformation (see low RMSD in Figures S106 and S112). Therefore, we decided to combine these observations and use the acid-arginine interaction as an anchoring point to enhance additional van der Waals contacts with hydrophobic substituents only in the *cis*-isomer (Figure 6A). Furthermore, we hypothesized that exposing these hydrophobic moieties to the solvent should further penalize the *trans*-isomer (Figure 6A).

Starting from compounds **10** and **11**, we substituted the outer benzene ring with a methyl group or a chlorine atom in *meta* or *para* positions. The hydrophobic interactions can be formed with two different sides of the lipophilic subpocket: Leu28 and Phe31 or Leu54 and Ile50. IFD indicated that contacts with Leu28 and Phe31 were more favorable, and MD simulations gave additional time-domain support to the hypothesis. Both the lipophilic substituents and residues displayed a decrease in SASA upon switching (Figures 6C, S121, S122, S126–S128, S132, S133, and S137–139).

Photochemical evaluation of compounds **12**–**15** revealed that the addition of methyl and chlorine substituents enhanced the resistance to reduction by GSH (Figures S46, S50, S54, and S58). We also observed an increase in PSD^365nm^ compared to the parent compounds **10** and **11** (Figure S61). Furthermore, the original solubility of compound **7** in eDHFR buffer was improved with a substituent in *ortho* position to the carboxylic acid, possibly able to disrupt the crystal packing of the flat structure of *trans*-**7**.

We established that the starting point for the optimization clearly influenced the outcome of the design hypothesis, given that all photoswitchable inhibitors retained high potencies against eDHFR (Figure 6B). When the hydrophobicity hypothesis was tested on the *trans*-on compound **10** (i.e., analogues **12** and **13**), the approach gave less satisfactory results. In fact, **13** displayed no difference in activity between the isomers, while UV irradiation caused a remarkable 5-fold increase in IC_50_ for **12**. Conversely, modification of compound **11**, which had no significant difference in activity between the isomers, resulted in two *cis*-on ligands (i.e., analogues **14** and **15**). Irradiation with λ_1_ = 365 nm light induced the desired photoactivation of both derivatives, with a 2-fold increase in IC_50_. In the case of **15**, we observed a dark IC_50_ of 52 nM versus an irradiated IC_50_ of 27 nM. To our delight, this 2-fold activation was maintained for the antibacterial activity (Figure 6C), as the dark MIC of 40 μM was successfully increased to 20 μM upon irradiation. The biological results indicate that, when the binding pocket is solvent-exposed, the photoisomerization of azobenzene can be used to toggle between solvent exposure and burial of hydrophobic groups to induce differences in activity upon irradiation. This final hypothesis, which combines the insights obtained in the whole study presented herein, has also provided the best design in terms of general potency and its difference for the isomers, with the *cis* being more potent.

## Conclusions and Outlook

We presented here a systematic investigation of strategies inspired by structure-based drug design and applied to photopharmacology. The iterative design cycles of photoswitchable eDHFR inhibitors as a case study culminated in three (**6**, **14**, and **15**) *cis*-on ligands with low-nanomolar potencies, but especially gave precious insights that can serve as guidance for future design efforts. We have shown that computational techniques such as molecular docking and MD can offer hypothetical explanations of effects observed experimentally and can be useful for repeated DMSTA cycles. Obviously, structural biology methods would be more accurate, although they are generally more costly and time-consuming. Nevertheless, recent structural elucidations of the molecular recognition of the metastable isomers^70−72^ certainly have the potential to stimulate further structure-based approaches in photopharmacology. Qualitative analysis of docking and MD simulations (ligand strains, specific interactions, SASA) can promote the pragmatic understanding of biological results to guide the design of photopharmacological agents. For this purpose, a significant statistical ensemble (three replicas) and medium length (at least 100 ns) are recommended for the MD simulations, as a trade-off between computational cost and quality of the outcomes is necessary. These simulations are computationally cheaper than free energy calculations, but they do not allow quantitative predictions.^47^

The application of a structure-based approach instead of the more straightforward azo-extension led to a more compact molecular structure than that of the previous TMP conjugates (Figure 1C).^8^ As a result, we obtained compounds with physicochemical descriptors in better agreement with the ChEMBL data set with antibacterial activity against *E. coli* and with inhibitory activity against eDHFR (see SI Section S5.3). In particular, our photoswitchable antibiotics featured calculated log *P*s < 3.5 and molecular weights < 450 g/mol, whereas the best candidate of the previous series (TCAT) had a log *P* of 7.3 and a molecular weight of > 600 g/mol. This indirect physicochemical refinement highlights a crucial issue of the azo-extension strategy, that is, how to achieve switching in aqueous environment facing the substantial increase in lipophilicity and molecular weight that naturally stems from the attachment of an azobenzene to an existing drug.^62^ In response to such a tendency, we have shown that structure-based optimization can help photopharmacology to obtain more elegant molecular designs. Nevertheless, the above-mentioned improvements came at the expense of the inhibitory potencies of our compounds, with the best derivative (compound **15**) being 1 order of magnitude less potent than the previously reported TMP conjugates (Table S3).

In this study, we evaluated the performance of modeling predictions as guiding tools for the design of *cis*-on photoswitchable drugs. The isolated use of docking calculations and scores can lead to inaccurate conclusions,^46^ even more so in photopharmacology. In fact, we believe that comparisons of docking scores between the photoisomers of a compound should be avoided. On the other hand, our workflow demonstrated that a combination of molecular docking, MD simulations, and chemical intuition can create detailed knowledge of drug-target interactions at the molecular level. When the computer-aided predictions led to unexpected experimental results, these were carefully analyzed by re-examining the simulations in order to improve our subsequent hypotheses and our understanding of the system. Although strictly speaking the computational data stay hypothetical until structural data are collected, multiple iterations of DMSTA cycles can build a certain degree of local consensus with useful practical implications. In contrast to brute-force screenings that lead to serendipitous findings and to photocontrolled “me-too” drugs, hypothesis-driven drug design allows photopharmacology to go beyond trial-and-error approaches, addressing multiparameter issues, and is able to provide valuable insights for future endeavors.

## Experimental Section

### Synthesis

#### General Remarks

All chemicals for synthesis were obtained from commercial suppliers (Sigma-Aldrich, Combi-blocks, and Boom) and used as received, unless stated otherwise. Solvents used were reagent grade for synthesis and technical grade for isolation if not otherwise stated. Thin-layer chromatography was carried out on aluminum sheets coated with silica gel 60 F254 (Merck). The developed chromatogram was analyzed by UV lamp (254 nm) for the detection of components. Alternatively, oxidative staining using aqueous basic potassium permanganate solution (KMnO_4_) or aqueous acidic cerium phosphomolybdic acid solution (Seebach’s stain) was used. Flash chromatography was performed on silica gel (Screening devices B.V.) with a particle size of 40–64 μM and a pore size of 60 Å or on Buchi FlashPure silica columns (4–25 g, 40–63 μM, 60 Å) using a Buchi Reveleris X2 system. Preparative HPLC purification was performed on a Shimadzu HPLC system with a Phenomenex Kinetex 5 μm EVO C18 100 Å column. Schemes and detailed characterization data are reported in the Supporting Information (SI).

#### Analytical Procedures

Nuclear magnetic resonance (NMR) spectra were recorded on an Agilent Technologies 400-MR (400/54 Premium Shielded) spectrometer (400 MHz). All spectra were measured at room temperature (22–24 °C). Chemical shifts for the specific NMR spectra were reported relative to the residual solvent peak in ppm (δ_H_ = 7.26 and δ_C_ = 77.16 for CDCl_3_; δ_H_ = 2.49 and δ_C_ = 39.5 for DMSO-*d*_6_]. The multiplicities of the signals are denoted by s (singlet), d (doublet), t (triplet), q (quartet), m (multiplet), br (broad), app (apparent). All ^13^C NMR spectra are ^1^H-broadband decoupled. High-resolution mass spectrometry was performed on a Thermo scientific LTQ Orbitrap XL in positive (ACPI/ESI) or negative (ESI) mode. Melting point ranges were determined on a Stuart analogue capillary melting point SMP11 apparatus. All compounds whose IC_50_ values were determined had purities ≥95% (SI). Purities were determined with a Thermo Ultimate 3000 QExactive Orbitrap instrument (Thermo Scientific) equipped with a C18 column (Acquity UPLC BEH C18, 150 × 2.1 mm, 1.7 μm) and operating at 25 °C. The injection volume of each sample in acetonitrile was 1 μL. The flow rate of the mobile phases was 0.3 mL/min. The elution was carried out using water containing 0.1% formic acid as mobile phase A and acetonitrile as mobile phase B. Elution conditions: From 0 until 1 min, 95% phase A + 5% phase B; at 8 min, 40% phase A + 60% phase B; from 10 until 11 min, 10% phase A + 90% phase B; from 12 until 17 min, 95% phase A + 5% phase B. Chromatograms were detected at 254 and 360 nm.

#### General Procedure for Baeyer–Mills Reactions (A)

The aromatic nitroso compound (1.2 equiv) and the aniline (1.0 equiv) were dissolved in a minimal volume of AcOH/DCM (1:1, v/v). The mixture was stirred at r.t. in absence of light for 3 d. The mixture was neutralized with sat. aq. NaHCO_3_ and extracted with EtOAc. The organic layer was washed with brine, and dried over MgSO_4_, and the solvent was removed under reduced pressure. The crude product was purified by flash chromatography.

#### General Procedure for Deprotections of TMS-Acetylene (B)

The TMS-alkyne (1.0 equiv) was dissolved in MeOH (0.1 M). K_2_CO_3_ (2.0 equiv) was added, and the mixture was stirred for 4 h at r.t. MeOH was removed under reduced pressure, and the residue was dissolved in EtOAc. The organic layer was washed with sat. aq. NH_4_Cl and brine and dried over MgSO_4_, and the solvent was removed under reduced pressure. Unless stated otherwise, the crude product was used directly in the next step without purification.

#### General Procedure for Sonogashira Couplings (C)

The protocol was obtained by adapting a literature procedure.^50^ To a flame-dried three-necked round-bottom flask under N_2_ atmosphere, the terminal alkyne (1.1 equiv), compound **S1** (1 equiv), Pd(PPh_3_)_2_Cl_2_ (0.08 equiv), CuI (0.05 equiv), and KOAc (10.0 equiv) were added. After the solids were loaded, the flask was evacuated under vacuum and flushed with N_2_ three times. Subsequently, the solids were dissolved in dry DMF (0.1 M solution of alkyne). If the terminal alkyne was an oil, it was separately dissolved in dry DMF under N_2_ and added at this stage. The reaction mixture was deoxygenated by bubbling dry N_2_ through it for 10 min. The mixture was stirred for 2 d at 55 °C, filtered over Celite, and diluted with EtOAc. The organic layer was washed with sat. aq. NaHCO_3_ and brine and dried over MgSO_4_, and the solvent was removed under reduced pressure. The crude product was purified by flash chromatography.

#### General Procedure for Nucleophilic Additions of TMS-Acetylene Followed by Methylation (D)

In a flame-dried, three-necked round-bottom flask under N_2_ atmosphere, trimethylsilylacetylene (varying equiv) was dissolved in dry THF (0.1 M). The solution was cooled to −78 °C with an acetone/liquid N_2_ bath, and *n*-BuLi (1.6 M in hexanes, varying equiv) was added dropwise. After stirring at −78 °C for 30 min, the cooling bath was removed, and the mixture was stirred at r.t. for 1 h. Subsequently, a 0.2 M solution of the ketone (1.0 equiv) in dry THF was added dropwise at −78 °C. The mixture was kept at −78 °C for 3 h and then was allowed to reach −40 °C, and it was stirred for an additional 2 h. Then, iodomethane (10 equiv) was added at −40 °C, and the solution was allowed to reach r.t. overnight. The reaction mixture was neutralized with sat. aq. NaHCO_3_ and diluted with EtOAc. The organic layer was washed with sat. aq. NH_4_Cl and brine and dried over MgSO_4_, and the solvent was removed under reduced pressure. The crude product was purified by flash chromatography.

#### General Procedure for Hydrolyses of Methyl Esters (E)

The methyl ester (1.0 equiv) was dissolved in MeOH and THF, then LiOH (6.0 equiv) in water was added (THF/MeOH/H_2_O, 1:1.4:0.7, v/v). The reaction mixture was stirred at 32 °C for 16 h. The mixture was quenched with sat. aq. NH_4_Cl and extracted with 15% isopropyl alcohol in EtOAc. The organic layer was washed with brine and dried over Na_2_SO_4_, and the solvent was removed under reduced pressure. The crude product was purified by either flash chromatography or preparative HPLC.

#### 5-Iodopyrimidine-2,4-diamine (**S1**)

Prepared following a literature procedure (see SI Section S1.2).^50^

#### Benzenediazonium Tetrafluoroborate (**S2**)

Prepared following a literature procedure (see SI Section S1.2).^73^

#### (*E*)-5-(Phenyldiazenyl)pyrimidine-2,4-diamine (**1**)

Prepared adapting a literature procedure.^52^ To an ice-cooled solution of 2,4-diaminopyrimidine (0.20 g, 1.8 mmol) in acetate buffer NaOAc (pH 7, 15 mL), **S2** (0.50 g, 2.7 mmol) was added in small portions, and the reaction mixture was stirred overnight. The mixture was extracted with EtOAc (3 × 20 mL), washed with brine (3 × 20 mL), and dried over MgSO_4_, and the solvent was removed under reduced pressure. The crude product was purified by flash chromatography (DCM/MeOH = 96:4, v/v) to afford **1** as a yellow solid (21.0 mg, 98 μmol, 5%). *R*_*f*_: 0.30 (DCM/MeOH = 96:4, v/v). ^1^H NMR (400 MHz, DMSO-*d*_6_) δ 8.71 (br s, 1H), 8.49 (s, 1H), 7.77 (d, *J* = 7.5 Hz, 2H), 7.70 (br s, 1H), 7.49 (t, *J* = 7.5 Hz, 2H), 7.38 (t, *J* = 7.5 Hz, 1H), 6.97 (br s, 2H). ^13^C NMR (101 MHz, DMSO-*d*_6_) δ 162.7, 162.1, 155.6, 152.3, 129.2, 128.9, 124.3, 121.3. HRMS (ESI^+^) *m*/*z* calcd for [M + H]^+^ (C_10_H_11_N_6_^+^): 215.1040, found: 215.1038. Mp > 250 °C.

#### 5-(4-Aminophenyl)pyrimidine-2,4-diamine (**S3a**)

In a flame-dried, three-necked round-bottom flask, under N_2_ atmosphere, **S1** (0.48 g, 2.0 mmol), 4-aminophenylboronic acid pinacol ester (0.40 g, 1.8 mmol), and anhydrous K_2_CO_3_ (0.51 g, 3.7 mmol) were added to a mixture of 1,4-dioxane (5.0 mL), ethanol (1.5 mL) and water (3.3 mL). The reaction mixture was deoxygenated by bubbling dry N_2_ through it for 20 min. Subsequently, Pd(PPh_3_)_4_ (0.11 g, 91 μmol) was added, and the reaction mixture was stirred and heated under reflux at 95 °C. After 16 h, it was filtered over Celite and diluted with EtOAc (10 mL) and water (10 mL). The phases were separated, and the aqueous layer was extracted with EtOAc (3 × 10 mL). The organic layer was washed with brine and dried over Na_2_SO_4_, and the solvent was removed under reduced pressure. The crude product was purified by flash chromatography (DCM/MeOH/Et_3_N = 95:4:1, v/v) to afford **S3a** as a brown solid (0.30 g, 1.5 mmol, 81%). *R*_*f*_: 0.45 (DCM/MeOH/Et_3_N = 95:4:1, v/v). ^1^H NMR (400 MHz, DMSO-*d*_6_) δ 7.51 (s, 1H), 6.97 (AA′BB′, 2H), 6.60 (AA′BB′, 2H), 5.88 (br s, 4H), 5.12 (br s, 2H). ^13^C NMR (101 MHz, DMSO-*d*_6_) δ 161.7, 161.4, 154.4, 147.6, 129.0, 122.7, 114.2, 109.2. HRMS (ESI^+^) *m*/*z* calcd for [M + H]^+^ (C_10_H_12_N_5_^+^): 202.1087, found: 202.1085. Mp (dec.): 214 °C.

#### 5-(3-Aminophenyl)pyrimidine-2,4-diamine (**S3b**)

In a flame-dried, three-necked round-bottom flask, under N_2_ atmosphere, **S1** (0.48 g, 2.0 mmol), 3-aminophenylboronic acid (0.25 g, 1.8 mmol) and anhydrous K_2_CO_3_ (0.51 g, 3.7 mmol) were added to a mixture of 1,4-dioxane (5.0 mL), ethanol (1.5 mL) and water (3.3 mL). The reaction mixture was deoxygenated by bubbling dry N_2_ through it for 20 min. Subsequently, Pd(PPh_3_)_4_ (0.11 g, 91 μmol) was added, and the reaction mixture was stirred while heated under reflux at 95 °C. After 16 h, it was filtered over Celite and diluted with EtOAc (10 mL) and water (10 mL). The phases were separated, and the aqueous layer was extracted with EtOAc (3 × 10 mL). The organic layer was washed with brine and dried over Na_2_SO_4_, and the solvent was removed under reduced pressure. The crude product was purified by flash chromatography (DCM/MeOH/Et_3_N = 95:4:1, v/v) to afford **S3b** as a yellow solid (0.26 g, 1.3 mmol, 72%). *R*_*f*_: 0.41 (DCM/MeOH/Et_3_N = 95:4:1, v/v). ^1^H NMR (400 MHz, DMSO-*d*_6_) δ 7.58 (s, 1H), 7.05 (t, *J* = 7.7 Hz, 1H), 6.54–6.49 (m, 2H), 6.46 (d, *J* = 7.7, 1H), 6.10 (br s, 4H), 5.17 (br s, 2H). ^13^C NMR (101 MHz, DMSO-*d*_6_) δ 161.4, 161.2, 153.5, 136.1, 129.4, 115.7, 113.7, 112.6, 109.4. HRMS (ESI^+^) *m*/*z* calcd for [M + H]^+^ (C_10_H_12_N_5_^+^): 202.1087, found: 202.1085. Mp: 62–65 °C.

#### (*E*)-5-[4-(Phenyldiazenyl)phenyl]pyrimidine-2,4-diamine (**2**)

**S3a** (0.12 g, 0.60 mmol) was reacted following the general procedure A. The residue was purified by flash chromatography (DCM/MeOH = 97:3, v/v) to afford compound **2** as an orange solid (65 mg, 0.22 mmol, 38%). *R*_*f*_: 0.52 (DCM/MeOH = 9:1, v/v). ^1^H NMR (400 MHz, DMSO-*d*_6_) δ 7.96–7.88 (m, 4H), 7.77 (s, 1H), 7.64–7.54 (m, 5H), 6.23 (br s, 2H), 6.11 (br s, 2H). ^13^C NMR (101 MHz, DMSO-*d*_6_) δ 162.8, 161.0, 156.5, 152.1, 150.2, 139.9, 131.4, 129.5, 129.0, 123.2, 122.5, 107.4. HRMS (ESI^+^) *m*/*z* calcd for [M + H]^+^ (C_16_H_15_N_6_^+^): 291.1353, found: 291.1356. Mp: 199–201 °C.

#### (*E*)-5-[3-(Phenyldiazenyl)phenyl]pyrimidine-2,4-diamine (**3**)

**S3b** (0.1 g, 0.50 mmol) was reacted following the general procedure A. The residue was purified by flash chromatography (DCM/MeOH = 97:3, v/v) to afford compound **3** as an orange solid (57 mg, 0.20 mmol, 40%). *R*_*f*_: 0.53 (DCM/MeOH = 9:1, v/v). ^1^H NMR (400 MHz, DMSO-*d*_6_) δ 7.94–7.89 (m, 2H), 7.85 (s, 1H), 7.81 (d, *J* = 7.8 Hz, 1H), 7.74 (s, 1H), 7.65–7.56 (m, 4H), 7.53 (d, *J* = 7.8 Hz, 1H), 6.19 (br s, 2H), 6.06 (br s, 2H). ^13^C NMR (101 MHz, DMSO-*d*_6_) δ 162.8, 161.2, 156.3, 152.4, 152.0, 137.6, 131.7, 131.6, 130.1, 129.6, 122.7, 122.6, 120.8, 107.7. HRMS (ESI^+^) *m*/*z* calcd for [M + H]^+^ (C_16_H_15_N_6_^+^): 291.1353, found: 291.1357. Mp: 209–211 °C.

#### 4-[(Trimethylsilyl)ethynyl]aniline (**S4a**)

Prepared following a literature procedure (see SI Section S1.2).^74^

#### 3-[(Trimethylsilyl)ethynyl]aniline (**S4b**)

Prepared following a literature procedure (see SI Section S1.2).^75^

#### 4-Ethynylaniline (**S5a**)

**S4a** (0.40 g, 2.1 mmol) was reacted following the general procedure B. The crude product was purified by flash chromatography (pentane/EtOAc = 8:2, v/v) to afford **S5a** as a light pink solid (0.24 g, 2.0 mmol, 95%). *R*_*f*_: 0.49 (pentane/EtOAc = 8:2, v/v). ^1^H NMR (400 MHz, CDCl_3_) δ 7.30 (AA′BB′, 2H), 6.59 (AA′BB′, 2H), 3.81 (br s, 2H), 2.95 (s, 1H); spectrum in agreement with literature data.^76^ HRMS (ESI^+^) *m*/*z* calcd for [M + H]^+^ (C_8_H_8_N^+^): 118.0651, found: 118.0650. Mp: 96–98 °C (lit. 99–100 °C).^77^

#### 3-Ethynylaniline (**S5b**)

**S4b** (1.5 g, 8.2 mmol) was reacted following the general procedure B. Purification by flash chromatography was not required. The product **S5b** was obtained as a colorless solid (0.89 g, 7.6 mmol, 93%). *R*_*f*_: 0.60 (Pentane/EtOAc = 8:2, v/v). ^1^H NMR (400 MHz, DMSO-*d*_6_) δ 7.12 (m, 3H), 6.79 (d, *J* = 7.8 Hz, 1H), 5.31 (br s, 2H), 2.48 (s, 3H); spectrum in agreement with literature data.^78^ HRMS (ESI^+^) *m*/*z* calcd for [M + H]^+^ (C_8_H_8_N^+^): 118.0651, found: 118.0652. Mp: 86–87 °C (lit. not reported).

#### 5-[(4-Aminophenyl)ethynyl]pyrimidine-2,4-diamine (**S6a**)

**S5a** (0.15 g, 1.3 mmol) was reacted following the general procedure C. The residue was purified by flash chromatography (DCM/MeOH = 9:1, v/v) to afford **S6a** as a brown solid (0.16 g, 0.70 mmol, 64%). *R*_*f*_: 0.52 (DCM/MeOH = 9:1, v/v). ^1^H NMR (400 MHz, DMSO-*d*_6_) δ 7.85 (s, 1H), 7.19 (AA′BB′, 2H), 6.52 (AA′BB′, 2H), 6.43 (br s, 2H), 6.22 (br s, 2H), 5.41 (br s, 2H). ^13^C NMR (101 MHz, DMSO-*d*_6_) δ 163.2, 161.8, 158.5, 148.8, 132.2, 113.5, 109.4, 95.6, 90.9, 80.5. HRMS (ESI^+^) *m*/*z* calcd for [M + H]^+^ (C_12_H_12_N_5_^+^): 226.1087, found: 226.1089. Mp: 198–201 °C.

#### 5-[(3-Aminophenyl)ethynyl]pyrimidine-2,4-diamine (**S6b**)

**S5b** (0.31 g, 2.7 mmol) was reacted following the general procedure C. The residue was purified by flash chromatography (DCM/MeOH = 9:1, v/v) to afford **S6b** as a yellow solid (0.18 g, 0.79 mmol, 37%). *R*_*f*_: 0.40 (DCM/MeOH = 9:1, v/v). ^1^H NMR (400 MHz, DMSO-*d*_6_) δ 7.90 (s, 1H), 7.00 (d, *J* = 8.0 Hz, 1H), 6.70 (s, 1H), 6.70 (d, *J* = 8.0 Hz, 1H), 6.53 (d, *J* = 8.0 Hz, 1H), 6.51 (br s, 1H), 6.32 (br s, 2H), 5.13 (br s, 2H). ^13^C NMR (101 MHz, DMSO-*d*_6_) δ 163.3, 162.1, 159.3, 148.5, 128.9, 123.5, 118.6, 115.9, 113.8, 95.1, 90.0, 82.8. HRMS (ESI^+^) *m*/*z* calcd for [M + H]^+^ (C_12_H_12_N_5_^+^): 226.1087, found: 226.1085. Mp: 155–158 °C.

#### (*E*)-5-{[4-(Phenyldiazenyl)phenyl]ethynyl}pyrimidine-2,4-diamine (**4**)

**S6a** (86 mg, 0.38 mmol) was reacted following the general procedure A. The residue was purified by flash chromatography (DCM/MeOH = 97:3, v/v) to afford compound **4** as an orange solid (74 mg, 0.24 mmol, 62%). *R*_*f*_: 0.39 (DCM/MeOH = 96:4, v/v). ^1^H NMR (400 MHz, DMSO-*d*_6_) δ 8.01 (s, 1H), 7.90 (AA′BB′, 2H), 7.78 (AA′BB′, 2H), 7.60 (m, 3H), 6.76 (br s, 2H), 6.45 (br s, 2H). ^13^C NMR (101 MHz, DMSO-*d*_6_) δ 163.4, 162.4, 160.0, 152.0, 150.4, 131.7, 131.6, 129.5, 126.8, 122.8, 122.6, 94.5, 89.3, 88.2. HRMS (ESI^+^) *m*/*z* calcd for [M + H]^+^ (C_18_H_15_N_6_^+^): 315.1353, found: 315.1355. Mp: 220–222 °C.

#### (*E*)-5-{[3-(Phenyldiazenyl)phenyl]ethynyl}pyrimidine-2,4-diamine (**5**)

**S6b** (46 mg, 0.20 mmol) was reacted following the general procedure A. The residue was purified by flash chromatography (DCM/MeOH = 96:4, v/v) to afford compound **5** as an orange solid (18 mg, 57 μmol, 28%). *R*_*f*_: 0.56 (DCM/MeOH = 9:1, v/v). ^1^H NMR (400 MHz, DMSO-*d*_6_) δ 8.09 (s, 1H), 8.00 (s, 1H), 7.92 (d, *J* = 7.3 Hz, 2H), 7.85 (d, *J* = 8.0 Hz, 1H), 7.74 (d, *J* = 8.0 Hz, 1H), 7.66–7.56 (m, 4H), 6.76 (br s, 2H), 6.40 (br s, 2H). ^13^C NMR (101 MHz, DMSO-*d*_6_) δ 163.5, 162.4, 159.9, 151.9, 133.4, 131.8, 129.6, 129.5, 128.9, 124.8, 124.3, 122.6, 122.0, 120.0, 93.6, 89.3, 85.9. HRMS (ESI^+^) *m*/*z* calcd for [M + H]^+^ (C_18_H_15_N_6_^+^): 315.1353, found: 315.1358. Mp: 207–209 °C.

#### 1-(4-Nitrosophenyl)ethan-1-one (**S7a**)

Prepared following a literature procedure.^79^ To a solution of 4′-aminoacetophenone (10 g, 74 mmol) in DCM (0.20 L), a solution of oxone (78 g, 0.13 mol) in water (0.40 L) was added, and the suspension stirred vigorously at r.t. for 1.5 h. The reaction mixture was diluted with DCM (0.20 L) and washed with 1 M aq. HCl (2 × 0.10 L), saturated aq. NaHCO_3_ (2 × 0.10 L), and brine (2 × 0.10 L), and dried over MgSO_4_, and the solvent was removed under reduced pressure. The yellow solid (crude yield: 95%) was used directly for the next step without purification.

#### 1-(3-Nitrosophenyl)ethan-1-one (**S7b**)

To a solution of 3′-aminoacetophenone (3.4 g, 25 mmol) in DCM (60 mL), a solution of oxone (15 g, 25 mmol) in water (80 mL) was added, and the suspension stirred vigorously at r.t. for 1.5 h. The reaction mixture was diluted with DCM (60 mL) and washed with 1 M aq. HCl (2 × 30 mL), saturated aq. NaHCO_3_ (2 × 30 mL), and brine (2 × 30 mL), and dried over MgSO_4_, and the solvent was removed under reduced pressure. The brown solid (crude yield: 64%) was used directly for the next step without purification.

#### (*E*)-1-[4-(Phenyldiazenyl)phenyl]ethan-1-one (**S8a**)

Aniline (1.0 g, 11 mmol) was reacted following the general procedure A. The residue was purified by flash chromatography (Pentane/EtOAc = 95:5, v/v) to afford compound **S8a** as an orange solid (1.5 g, 6.7 mmol, 61%). *R*_*f*_: 0.23 (petroleum ether/EtOAc = 95:5, v/v). ^1^H NMR (400 MHz, CDCl_3_) δ 8.11 (AA′BB′, 2H), 8.00–7.93 (m, 4H), 7.57–7.49 (m, 3H), 2.67 (s, 3H), spectrum in agreement with literature data.^80^ HRMS (APCI^+^) *m*/*z* calcd for [M + H]^+^ (C_14_H_13_N_2_O^+^): 225.1022, found: 225.1022. Mp: 108–112 °C (lit. 102–104).^80^

#### (*E*)-1-[3-(Phenyldiazenyl)phenyl]ethan-1-one (**S8b**)

Aniline (2.0 g, 13 mmol) was reacted following the general procedure A. The residue was purified by flash chromatography (Pentane/EtOAc = 9:1, v/v) to afford compound **S8b** as an orange solid (1.1 g, 4.7 mmol, 35%). *R*_*f*_: 0.66 (Pentane/EtOAc = 8:2, v/v). ^1^H NMR (400 MHz, CDCl_3_) δ 8.45 (s, 1H), 8.08 (d, *J* = 7.8 Hz, 1H), 8.04 (d, *J* = 7.8 Hz, 1H), 7.93 (d, *J* = 7.8 Hz, 2H), 7.57 (t, *J* = 7.8 Hz, 1H), 7.50 (m, 3H), 2.66 (s, 3H), spectrum in agreement with literature data.^81^ HRMS (APCI^+^) *m*/*z* calcd for [M + H]^+^ (C_14_H_13_N_2_O^+^): 225.1022, found: 225.1030. Mp: 81–84 °C (lit. 88–90).^82^

#### (*E*)-1-{4-[2-Methoxy-4-(trimethylsilyl)but-3-yn-2-yl]phenyl}-2-phenyldiazene (**S9a**)

**S8a** (0.80 g, 3.6 mmol) was reacted following the general procedure D. The residue was purified by flash chromatography (Pentane/EtOAc = 90:10, v/v) to afford compound **S9a** as an orange oil (0.69 g, 2.1 mmol, 58%). *R*_*f*_: 0.70 (petroleum ether/EtOAc = 96:4, v/v). ^1^H NMR (400 MHz, DMSO-*d*_6_) δ 7.95–7.87 (m, 4H), 7.73 (AA′BB′, 2H), 7.61 (m, 3H), 3.16 (s, 3H), 1.67 (s, 3H), 0.25 (s, 9H). ^13^C NMR (101 MHz, DMSO-*d*_6_) δ 151.9, 151.5, 145.5, 131.6, 129.5, 126.8, 122.6, 122.6, 105.1, 92.3, 75.8, 51.8, 31.6, −0.2. HRMS (ESI^+^) *m*/*z* calcd for [M + H]^+^ (C_20_H_25_N_2_OSi^+^): 337.1731, found: 337.1739.

#### (*E*)-1-{3-[2-Methoxy-4-(trimethylsilyl)but-3-yn-2-yl]phenyl}-2-phenyldiazene (**S9b**)

**S8b** (0.77 g, 3.4 mmol) was reacted following the general procedure D. The residue was purified by flash chromatography (Pentane/EtOAc = 97:3, v/v) to afford **S9b** as an orange oil (1.0 g, 3.1 mmol, 90%). *R*_*f*_: 0.70 (Pentane/EtOAc = 95:5, v/v). ^1^H NMR (400 MHz, CDCl_3_) δ 8.20 (s, 1H), 7.94 (d, *J* = 7.8 Hz, 2H), 7.87 (d, *J* = 7.8 Hz, 1H), 7.72 (d, *J* = 7.8 Hz, 1H), 7.52 (m, 4H), 3.26 (s, 3H), 1.78 (s, 3H), 0.29 (s, 9H). ^13^C NMR (101 MHz, CDCl_3_) δ 152.8, 152.8, 144.1, 131.2, 129.2, 129.2, 128.7, 123.0, 122.9, 120.6, 105.1, 93.0, 76.8, 52.7, 32.6, 0.1. HRMS (ESI^+^) *m*/*z* calcd for [M + H]^+^ (C_20_H_25_N_2_OSi^+^): 337.1731, found: 337.1732.

#### (*E*)-1-[4-(2-Methoxybut-3-yn-2-yl)phenyl]-2-phenyldiazene (**S10a**)

**S9a** (0.33 g, 1.0 mmol) was reacted following the general procedure B. Purification by flash chromatography was not required. The product **S10a** was obtained as an orange solid (0.24 g, 0.95 mmol, 93%). *R*_*f*_: 0.60 (petroleum ether/EtOAc = 95:5, v/v). ^1^H NMR (400 MHz, DMSO-*d*_6_) δ 7.96–7.87 (m, 4H), 7.75 (AA′BB′, 2H), 7.64–7.55 (m, 3H), 3.92 (s, 1H), 3.16 (s, 3H), 1.69 (s, 3H). ^13^C NMR (101 MHz, DMSO-*d*_6_) δ 151.9, 151.5, 145.4, 131.6, 129.5, 126.8, 122.6, 122.6, 83.0, 79.0, 75.5, 51.9, 31.7. HRMS (ESI^+^) *m*/*z* calcd for [M + H]^+^ (C_17_H_17_N_2_O^+^): 265.1335, found: 265.1344. Mp: 54–56 °C.

#### (*E*)-1-[3-(2-Methoxybut-3-yn-2-yl)phenyl]-2-phenyldiazene (**S10b**)

**S9b** (1.0 g, 3.0 mmol) was reacted following the general procedure B. Purification by flash chromatography was not required. The product **S10b** was obtained as a dark orange oil (0.67 g, 2.5 mmol, 85%). *R*_*f*_: 0.80 (Pentane/EtOAc = 95:5, v/v). ^1^H NMR (400 MHz, CDCl_3_) δ 8.18 (s, 1H), 7.94 (d, *J* = 6.8 Hz, 2H), 7.86 (d, *J* = 7.8 Hz, 1H), 7.73 (d, *J* = 7.8 Hz, 1H), 7.56–7.46 (m, 5H), 3.29 (s, 3H), 2.79 (s, 1H), 1.81 (s, 3H). ^13^C NMR (101 MHz, CDCl_3_) δ 152.9, 152.8, 143.8, 131.2, 129.2, 128.6, 123.0, 121.9, 121.4, 83.5, 76.4, 76.1, 52.8, 32.6. HRMS (ESI^+^) *m*/*z* calcd for [M + H]^+^ (C_17_H_17_N_2_O^+^): 265.1335, found: 265.1331.

#### (*E*)-5-{3-Methoxy-3-[4-(phenyldiazenyl)phenyl]but-1-yn-1-yl}pyrimidine-2,4-diamine (**6**)

**S10a** (0.15 g, 0.57 mmol) was reacted following the general procedure C. The residue was purified by flash chromatography (DCM/MeOH = 96:4, v/v) and subsequent recrystallization (Pentane/EtOAc = 80:20, v/v) to afford compound **6** as an orange solid (25 mg, 67 μmol, 11%). *R*_*f*_: 0.42 (DCM/MeOH = 96:4, v/v). ^1^H NMR (400 MHz, DMSO-*d*_6_) δ 7.99 (s, 1H), 7.96–7.87 (m, 4H), 7.81 (AA′BB′, 2H), 7.60 (m, 3H), 6.40 (br s, 4H), 3.22 (s, 3H), 1.79 (s, 3H). ^13^C NMR (101 MHz, DMSO-*d*_6_) δ 163.5, 162.4, 160.1, 152.0, 151.4, 146.4, 131.6, 129.5, 127.0, 122.6, 93.7, 88.9, 82.3, 76.4, 52.0, 31.8. HRMS (ESI^+^) *m*/*z* calcd for [M + H]^+^ (C_21_H_21_N_6_O^+^): 373.1771, found: 373.1775. Mp: 149–150 °C.

#### (*E*)-5-{3-Methoxy-3-[3-(phenyldiazenyl)phenyl]but-1-yn-1-yl}pyrimidine-2,4-diamine (**7**)

**S10b** (0.11 g, 0.43 mmol) was reacted following the general procedure C. The residue was purified by flash chromatography (DCM/MeOH = 95:5, v/v) to afford compound **7** as an orange solid (0.12 mg, 0.31 mmol, 72%). *R*_*f*_: 0.40 (DCM/MeOH = 95:5, v/v). ^1^H NMR (400 MHz, DMSO-*d*_6_) δ 8.11 (s, 1H), 7.99 (s, 1H), 7.91 (dd, *J* = 7.9, 1.6 Hz, 2H), 7.87 (d, *J* = 7.9 Hz, 1H), 7.80 (d, *J* = 7.9 Hz, 1H), 7.65 (t, *J* = 7.8 Hz, 1H), 7.62–7.55 (m, 3H), 6.40 (br s, 4H), 3.22 (s, 3H), 1.81 (s, 3H). ^13^C NMR (101 MHz, DMSO-*d*_6_) δ 163.5, 162.4, 160.0, 151.9, 144.7, 131.6, 129.6, 129.5, 129.0, 122.6, 122.2, 119.7, 93.7, 88.9, 82.3, 76.5, 52.0, 31.9. HRMS (ESI^+^) *m*/*z* calcd for [M + H]^+^ (C_21_H_21_N_6_O^+^): 373.1771, found: 373.1776. Mp: 105–107 °C.

#### (*E*)-1-{4-[(4-Methoxyphenyl)diazenyl]phenyl}ethan-1-one (**S11a**)

4-Methoxyaniline (1.8 g, 14 mmol) was reacted following the general procedure A. The residue was purified by recrystallization from EtOAc to afford compound **S11a** as an orange solid (1.0 g, 4.0 mmol, 28%). *R*_*f*_: 0.18 (petroleum ether/EtOAc = 95:5, v/v). ^1^H NMR (400 MHz, CDCl_3_) δ 8.09 (AA′BB′, 2H), 7.94 (m, 4H), 7.03 (AA′BB′, 2H), 3.90 (s, 3H), 2.65 (s, 3H). ^13^C NMR (101 MHz, CDCl_3_) δ 197.6, 162.9, 155.4, 147.2, 138.0, 129.5, 125.4, 122.7, 114.5, 55.8, 26.9. HRMS (ESI^+^) *m*/*z* calcd for [M + H]^+^ (C_15_H_15_N_2_O_2_^+^): 255.1128, found: 255.1134. Mp: 143–144 °C.

#### (*E*)-1-{4-[(1,3,5-Trimethyl-1H-pyrazol-4-yl)diazenyl]phenyl}ethan-1-one (**S11b**)

The compound was synthesized adapting a published procedure.^83^ 1-(4-Aminophenyl)ethan-1-one (11 mmol, 1.0 equiv) was dissolved in AcOH (30 mL) and aq. HCl (conc., 1.5 mL), and NaNO_2_ (920 mg, 13 mmol, 1.2 equiv) dissolved in water (8.0 mL) was added dropwise and stirred for 20 min. Then, the resulting mixture was added to acetyl acetone (1.4 g, 1.5 mL, 14 mmol, 1.3 equiv) and NaOAc (2.7 g, 33 mmol, 3.0 equiv) in ethanol (15 mL). The reaction mixture was stirred for 3 h at ambient temperature. The formed yellow precipitate was filtered off and dried under high vacuum. The solid (1.0 equiv) was dissolved in ethanol (0.16 L), methyl hydrazone (0.40 g, 8.8 mmol, 1.0 equiv., 0.46 mL) was added, and the mixture stirred at 85 °C for 5 h. The solvent was evaporated, and the crude material was purified by flash chromatography (10 → 100% EtOAc in petroleum ether). Compound **S11b** was isolated as a yellow solid (2.0 g, 7.7 mmol, 69% over two steps). ^1^H NMR (400 MHz, DMSO-*d*_6_) δ 8.08 (AA′BB′, 2H), 7.81 (AA′BB′, 2H), 3.75 (s, 3H), 2.61 (s, 3H), 2.57 (s, 3H), 2.38 (s, 3H). ^13^C NMR (101 MHz, DMSO-*d*_6_) δ 197.3, 155.6, 140.7, 136.8, 134.9, 129.5, 121.4, 36.0, 26.8, 13.8, 9.5. HRMS (ESI^+^) *m*/*z* calcd for [M + H]^+^ (C_14_H_17_N_4_O^+^): 257.1397, found: 257.1400. Mp: 112–114 °C.

#### (*E*)-1-{4-[2-Methoxy-4-(trimethylsilyl)but-3-yn-2-yl]phenyl}-2-(4-methoxyphenyl)diazene (**12a**)

**S11a** (0.70 g, 2.8 mmol) was reacted following the general procedure D. The residue was purified by flash chromatography (petroleum ether/EtOAc = 88:12, v/v) to afford compound **S12a** as an orange solid (0.89 g, 2.4 mmol, 89%). *R*_*f*_: 0.73 (petroleum ether/EtOAc = 88:12, v/v). ^1^H NMR (400 MHz, CDCl_3_) δ 7.93 (AA′BB′, 2H), 7.87 (AA′BB′, 2H), 7.72 (AA′BB′, 2H), 7.02 (AA′BB′, 2H), 3.90 (s, 3H), 3.24 (s, 3H), 1.74 (s, 3H), 0.27 (s, 9H). ^13^C NMR (101 MHz, CDCl_3_) δ 162.3, 152.5, 147.2, 145.0, 127.0, 125.0, 122.6, 114.4, 105.1, 92.7, 76.7, 55.7, 52.6, 32.6, 0.1. HRMS (ESI^+^) *m*/*z* calcd for [M + H]^+^ (C_21_H_27_N_2_O_2_Si^+^): 367.1836, found: 367.1842. Mp: 59–62 °C.

#### (*E*)-4-{[4-(2-Methoxy-4-(trimethylsilyl)but-3-yn-2-yl)phenyl]diazenyl}-1,3,5-trimethyl-1H-pyrazole (**12b**)

**S11b** (1.5 g, 5.9 mmol) was reacted following the general procedure D. The residue was purified by flash chromatography (petroleum ether/EtOAc = 7:3, v/v) to afford compound **S12b** as an orange oil (1.9 g, 1.9 mmol, 89%). *R*_*f*_: 0.31 (petroleum ether/EtOAc = 7:3, v/v). ^1^H NMR (400 MHz, CDCl_3_) δ 7.77 (AA′BB′, 2H), 7.67 (AA′BB′, 2H), 3.78 (s, 3H), 3.22 (s, 3H), 2.57 (s, 3H), 2.50 (s, 3H), 1.73 (s, 3H), 0.26 (s, 9H). ^13^C NMR (101 MHz, CDCl_3_) δ 153.3, 143.7, 142.6, 138.9, 135.3, 126.8, 121.7, 105.3, 92.6, 76.7, 52.6, 36.1, 32.6, 14.0, 10.1, 0.1. HRMS (ESI^+^) *m*/*z* calcd for [M + H]^+^ (C_20_H_29_N_4_OSi^+^): 369.2105, found: 369.2110.

#### (*E*)-1-[4-(2-Methoxybut-3-yn-2-yl)phenyl]-2-(4-methoxyphenyl)diazene (**S13a**)

**S12a** (0.60 g, 1.4 mmol) was reacted following the general procedure B. Purification by flash chromatography was not required. The product **S13a** was obtained as an orange solid (0.31 mg, 0.89 mmol, 63%). An analytical sample was prepared by recrystallization from MeCN/Pentane to give **S13a** as orange crystals. *R*_*f*_: 0.55 (petroleum ether/EtOAc = 9:1, v/v). ^1^H NMR (400 MHz, CDCl_3_) δ 7.93 (AA′BB′, 2H), 7.87 (AA′BB′, 2H), 7.73 (AA′BB′, 2H), 7.02 (AA′BB′, 2H), 3.90 (s, 3H), 3.26 (s, 3H), 2.77 (s, 1H), 1.77 (s, 3H). ^13^C NMR (101 MHz, CDCl_3_) δ 162.3, 152.5, 147.2, 144.5, 126.9, 125.0, 122.6, 114.4, 83.5, 76.4, 75.9, 55.8, 52.8, 32.6. HRMS (ESI^+^) *m*/*z* calcd for [M + H]^+^ (C_18_H_19_N_2_O_2_^+^): 295.1441, found: 295.1444. Mp: 93–95 °C.

#### (*E*)-4-{{4-(2-Methoxybut-3-yn-2-yl)phenyl]diazenyl}-1,3,5-trimethyl-1H-pyrazole (**S13b**)

**S12b** (1.9 g, 5.1 mmol) was reacted following the general procedure B. The residue was purified by flash chromatography (petroleum ether/EtOAc = 8:2, v/v) to afford compound **S13b** as an orange solid (1.5 mg, 5.1 mmol, 95%). *R*_*f*_: 0.33 (petroleum ether/EtOAc = 1:1, v/v). ^1^H NMR (400 MHz, CDCl_3_) δ 7.77 (AA′BB′, 2H), 7.69 (AA′BB′, 2H), 3.78 (s, 3H), 3.25 (s, 3H), 2.76 (s, 1H), 2.58 (s, 3H), 2.50 (s, 3H), 1.76 (s, 3H). ^13^C NMR (101 MHz, CDCl_3_) δ 153.4, 143.3, 142.6, 139.0, 135.4, 126.8, 121.8, 83.6, 76.4, 75.8, 52.7, 36.2, 32.7, 14.0, 10.1. HRMS (ESI^+^) *m*/*z* calcd for [M + H]^+^ (C_17_H_21_N_4_O^+^): 297.1710, found: 297.1714. Mp: 60–62 °C.

#### (*E*)-5-{3-Methoxy-3-[4-((4-methoxyphenyl)diazenyl]phenyl}but-1-yn-1-yl)pyrimidine-2,4-diamine (**8**)

**S13a** (0.25 g, 0.85 mmol) was reacted following the general procedure C. The residue was purified by flash chromatography (DCM/MeOH = 97:3, v/v) to afford compound **8** as an orange solid (0.23 g, 0.57 mmol, 76%). *R*_*f*_: 0.44 (DCM/MeOH = 96:4, v/v). ^1^H NMR (400 MHz, DMSO-*d*_6_) δ 7.99 (s, 1H), 7.90 (AA′BB′, 2H), 7.87 (AA′BB′, 2H), 7.78 (AA′BB′, 2H), 7.14 (AA′BB′, 2H), 6.40 (br s, 4H), 3.87 (s, 3H), 3.21 (s, 3H), 1.78 (s, 3H). ^13^C NMR (101 MHz, DMSO-*d*_6_) δ 163.5, 162.4, 162.1, 160.1, 151.5, 146.2, 145.6, 126.9, 124.6, 122.2, 114.6, 93.8, 88.9, 82.2, 76.4, 55.7, 52.0, 31.8. HRMS (ESI^+^) *m*/*z* calcd for [M + H]^+^ (C_22_H_23_N_6_O_2_^+^): 403.1877, found: 403.1884. Mp: 186–189 °C.

#### (*E*)-5-(3-Methoxy-3-(4-((1,3,5-trimethyl-1H-pyrazol-4-yl)diazenyl)phenyl)but-1-yn-1-yl)pyrimidine-2,4-diamine (**9**)

**S13b** (0.62 g, 2.1 mmol) was reacted following the general procedure C. The residue was purified by flash chromatography (DCM/MeOH = 95:5, v/v) to afford compound **9** as an orange solid (0.49 g, 1.2 mmol, 70%). An analytical sample was further purified by preparative HPLC. *R*_*f*_: 0.49 (DCM/MeOH = 9:1, v/v). ^1^H NMR (400 MHz, CDCl_3_) δ 8.13 (s, 1H), 7.79 (AA′BB′, 2H), 7.71 (AA′BB′, 2H), 5.19 (br s, 2H), 4.96 (br s, 2H), 3.79 (s, 3H), 3.29 (s, 3H), 2.58 (s, 3H), 2.50 (s, 3H), 1.84 (s, 3H). ^13^C NMR (101 MHz, CDCl_3_) δ 164.0, 162.0, 160.2, 153.5, 143.8, 142.6, 139.0, 135.4, 126.7, 121.9, 96.0, 92.0, 80.9, 77.2, 52.8, 36.2, 32.8, 14.0, 10.12. HRMS (ESI^+^) *m*/*z* calcd for [M + H]^+^ (C_22_H_20_ClN_6_O_3_^+^): 405.2146, found: 405.2151. Mp: 162–164 °C.

#### Methyl (*E*)-3-[(4-Acetylphenyl)diazenyl]benzoate (**S14a**)

Methyl 3-aminobenzoate (2.0 g, 14 mmol) was reacted following the general procedure A. The residue was purified by flash chromatography (toluene/EtOAc = 96:4, v/v) to afford compound **S14a** as an orange solid (2.7 g, 9.5 mmol, 69%). *R*_*f*_: 0.55 (toluene/EtOAc = 95:5, v/v). ^1^H NMR (400 MHz, CDCl_3_) δ 8.60 (s, 1H), 8.19 (AA′BB′, 1H), 8.16–8.10 (m, 3H), 8.00 (AA′BB′, 2H), 7.62 (t, *J* = 7.8 Hz, 1H), 3.98 (s, 3H), 2.67 (s, 3H). ^13^C NMR (101 MHz, CDCl_3_) δ 197.5, 166.6, 154.9, 152.6, 138.9, 132.5, 131.6, 129.6, 129.5, 127.4, 124.4, 123.2, 52.6, 27.0. HRMS (APCI^+^) *m*/*z* calcd for [M + H]^+^ (C_16_H_15_N_2_O_3_^+^): 283.1077, found: 283.1084. Mp: 123–125 °C.

#### Methyl (*E*)-4-[(4-Acetylphenyl)diazenyl]benzoate (**S14b**)

Methyl 4-aminobenzoate (0.82 g, 5.5 mmol) was reacted following the general procedure A. The residue was purified by flash chromatography (toluene/EtOAc = 98:2, v/v) to afford compound **S14b** as an orange solid (0.86 g, 3.0 mmol, 56%). *R*_*f*_: 0.45 (toluene/EtOAc = 98:2, v/v). ^1^H NMR (400 MHz, CDCl_3_) δ 8.21 (AA′BB′, 2H), 8.13 (AA′BB′, 2H), 8.03–7.97 (m, 4H), 3.97 (s, 3H), 2.68 (s, 3H). ^13^C NMR (101 MHz, CDCl_3_) δ 197.5, 166.5, 155.1, 155.0, 139.0, 132.6, 130.8, 129.6, 123.3, 123.1, 52.6, 27.0. HRMS (APCI^+^) *m*/*z* calcd for [M + H]^+^ (C_16_H_15_N_2_O_3_^+^): 283.1077, found: 283.1084. Mp: 185–189 °C.

#### Methyl (*E*)-3-{[4-(2-Methoxy-4-(trimethylsilyl)but-3-yn-2-yl)phenyl]diazenyl}benzoate (**S15a**)

**S15a** (1.5 g, 5.3 mmol) was reacted following the general procedure D. The residue was purified by flash chromatography (pentane/Et_2_O = 95:5, v/v) to afford compound **S15a** as an orange solid (1.0 g, 2.6 mmol, 48%). *R*_*f*_: 0.38 (pentane/Et_2_O = 95:5, v/v). ^1^H NMR (400 MHz, DMSO-*d*_6_) δ 8.38 (s, 1H), 8.17 (m, 2H), 7.98 (AA′BB′, 2H), 7.77 (m, 3H), 3.92 (s, 3H), 3.16 (s, 3H), 1.67 (s, 3H), 0.25 (s, 9H). ^13^C NMR (101 MHz, DMSO-*d*_6_) δ 165.6, 151.9, 151.4, 146.0, 131.8, 131.0, 130.3, 127.8, 126.9, 122.9, 122.1, 105.1, 92.4, 75.9, 52.5, 51.9, 31.6, −0.1. HRMS (ESI^+^) *m*/*z* calcd for [M + H]^+^ (C_22_H_27_N_2_O_3_Si^+^): 395.1786, found: 395.1794. Mp: < 50 °C.

#### Methyl (*E*)-4-{[4-(2-Methoxy-4-(trimethylsilyl)but-3-yn-2-yl)phenyl]diazenyl}benzoate (**S15b**)

**S14b** (0.40 g, 1.4 mmol) was reacted following the general procedure D. The residue was purified by flash chromatography (pentane/Et_2_O = 70:30, v/v) to afford compound **S15b** as an orange solid (0.18 g, 0.46 mmol, 32%). *R*_*f*_: 0.58 (petroleum ether/EtOAc = 95:5, v/v). ^1^H NMR (400 MHz, CDCl_3_) δ 8.20 (AA′BB′, 2H), 7.95 (AA′BB′, 2H), 7.94 (d, *J* = 8.7 Hz, 2H), 7.76 (d, *J* = 8.7 Hz, 2H), 3.96 (s, 3H), 3.25 (s, 3H), 0.27 (s, 9H). ^13^C NMR (101 MHz, CDCl_3_) δ 166.7, 155.3, 152.3, 146.6, 132.0, 130.8, 127.1, 123.2, 122.8, 104.9, 93.0, 52.7, 52.5, 32.6, 29.9, 0.1. HRMS (ESI^+^) *m*/*z* calcd for [M + H]^+^ (C_22_H_27_N_2_O_3_Si^+^): 395.1786, found: 395.1791. Mp: 86–89 °C.

#### Methyl (*E*)-3-{[4-(2-Methoxybut-3-yn-2-yl)phenyl]diazenyl}benzoate (**S16a**)

**S15a** (0.9 g, 2.3 mmol) was reacted following the general procedure B. Purification by flash chromatography was not required. The product **S16a** was obtained as an orange solid (0.71 g, 2.2 mmol, 95%). *R*_*f*_: 0.49 (pentane/EtOAc = 95:5, v/v). ^1^H NMR (400 MHz, CDCl_3_) δ 8.55 (s, 1H), 8.12 (d, *J* = 7.8 Hz, 1H), 8.07 (d, *J* = 7.8 Hz, 1H), 7.92 (AA′BB′, 2H), 7.75 (AA′BB′, 2H), 7.55 (t, *J* = 7.8 Hz, 1H), 3.94 (s, 3H), 3.25 (s, 3H), 2.78 (s, 1H), 1.76 (s, 3H). ^13^C NMR (101 MHz, CDCl_3_) δ 166.5, 152.6, 152.1, 145.7, 131.8, 131.3, 129.2, 127.0, 126.9, 124.1, 123.1, 83.3, 76.3, 76.1, 52.7, 52.4, 32.5. HRMS (APCI^+^) *m*/*z* calcd for [M + H]^+^ (C_19_H_19_N_2_O_3_^+^): 323.1390, found: 323.1389. Mp: 59–61 °C.

#### Methyl (*E*)-4-{[4-(2-Methoxybut-3-yn-2-yl)phenyl]diazenyl}benzoate (**S16b**)

**S15b** (0.16 g, 0.41 mmol) was reacted following the general procedure B. Purification by flash chromatography was not required. The product **S16b** was obtained as an orange solid (0.13 g, 0.39 mmol, 95%). *R*_*f*_: 0.45 (pentane/EtOAc = 95:5, v/v). ^1^H NMR (400 MHz, CDCl_3_) δ 8.20 (AA′BB′, 2H), 7.95 (AA′BB′, 2H), 7.95 (AA′BB′, 2H), 7.77 (AA′BB′, 2H), 3.96 (s, 3H), 3.27 (s, 3H), 2.78 (s, 1H), 1.78 (s, 3H). ^13^C NMR (101 MHz, CDCl_3_) δ 166.7, 155.3, 152.3, 146.1, 132.0, 130.8, 127.0, 123.3, 122.8, 83.3, 76.4, 76.1, 52.8, 52.5, 32.6. HRMS (APCI^+^) *m*/*z* calcd for [M + H]^+^ (C_19_H_19_N_2_O_3_^+^): 323.1390, found: 323.1388. Mp: 119–122 °C.

#### Methyl (*E*)-3-{[4-(4-(2,4-Diaminopyrimidin-5-yl)-2-methoxybut-3-yn-2-yl]phenyl}diazenyl)benzoate (**S17a**)

**S16a** (0.24 g, 0.75 mmol) was reacted following the general procedure C. The residue was purified by flash chromatography (DCM/MeOH = 96:4, v/v) to afford **S17a** as an orange solid (0.22 g, 0.52 mmol, 69%). *R*_*f*_: 0.42 (DCM/MeOH = 96:4, v/v). ^1^H NMR (400 MHz, DMSO-*d*_6_) δ 8.39 (s, 1H), 8.19 (d, *J* = 7.7 Hz, 1H), 8.15 (d, *J* = 7.7 Hz, 1H), 7.98 (m, 3H), 7.83 (AA′BB′, 2H), 7.78 (t, *J* = 7.7 Hz, 1H), 6.40 (br s, 4H), 3.92 (s, 3H), 3.22 (s, 3H), 1.79 (s, 3H). 13C NMR (101 MHz, DMSO-*d*_6_) δ 165.6, 163.5, 162.4, 160.1, 151.9, 151.2, 147.0, 131.7, 131.0, 130.2, 127.8, 127.1, 122.8, 122.0, 93.6, 88.9, 82.3, 76.4, 52.5, 52.0, 31.8. HRMS (ESI^+^) *m*/*z* calcd for [M + H]^+^ (C_23_H_23_N_6_O_3_^+^): 431.1826, found: 431.1835. Mp (dec.): 175 °C.

#### Methyl (*E*)-4-{[4-(4-(2,4-Diaminopyrimidin-5-yl)-2-methoxybut-3-yn-2-yl)phenyl]diazenyl}benzoate (**S17b**)

**S16b** (0.24 g, 0.75 mmol) was reacted following the general procedure C. The residue was purified by flash chromatography (DCM/MeOH = 96:4, v/v) to afford **S17b** as an orange solid (0.22 g, 0.52 mmol, 53%). *R*_*f*_: 0.39 (DCM/MeOH = 96:4, v/v). ^1^H NMR (400 MHz, DMSO-*d*_6_) δ 8.20–8.15 (AA′BB′, 2H), 7.99 (m, 5H), 7.84 (AA′BB′, 2H), 6.39 (br s, 4H), 3.91 (s, 3H), 3.23 (s, 3H), 1.79 (s, 3H). ^13^C NMR (101 MHz, DMSO-*d*_6_) δ 165.6, 163.5, 162.4, 160.1, 154.5, 151.4, 147.3, 131.6, 130.6, 127.1, 122.9, 122.7, 93.5, 88.9, 82.4, 76.4, 52.5, 52.0, 31.8. HRMS (ESI^+^) *m*/*z* calcd for [M + H]^+^ (C_23_H_23_N_6_O_3_^+^): 431.1826, found: 431.1827. Mp (dec.): 205 °C.

#### (*E*)-3-{[4-(4-(2,4-Diaminopyrimidin-5-yl)-2-methoxybut-3-yn-2-yl)phenyl]diazenyl}benzoic acid (**10**)

**S17a** (0.14 g, 0.33 mmol) was reacted following the general procedure E. The residue was dispersed in 10 mL of a 9:1 mixture of DCM and MeOH and sonicated. The solid was filtered off to afford compound **10** as an orange solid (0.11 g, 0.26 mmol, 78%). *R*_*f*_: 0.50 (DCM/MeOH/AcOH = 9:1:0.1, v/v). ^1^H NMR (400 MHz, DMSO-*d*_6_) δ 8.38 (s, 1H), 8.14 (t, *J* = 8.0 Hz, 2H), 8.01–7.94 (m, 3H), 7.83 (AA′BB′, 2H), 7.74 (t, *J* = 8.0 Hz, 1H), 7.31 (s, 1H), 6.41 (br s, 4H), 3.22 (s, 3H), 1.79 (s, 3H). ^13^C NMR (101 MHz, DMSO-*d*_6_) δ 166.7, 163.5, 162.3, 159.9, 151.9, 151.3, 146.9, 132.5, 131.9, 130.0, 127.3, 127.1, 122.8, 122.2, 93.6, 88.9, 82.2, 76.4, 52.0, 31.8. HRMS (ESI^+^) *m*/*z* calcd for [M + H]^+^ (C_22_H_21_N_6_O_3_^+^): 417.1670, found: 417.1674. Mp (dec.): 190 °C.

#### (*E*)-4-{[4-(4-(2,4-Diaminopyrimidin-5-yl)-2-methoxybut-3-yn-2-yl)phenyl]diazenyl}benzoic acid (**11**)

**S17b** (64 mg, 0.15 mmol) was reacted following the general procedure E. The residue was dispersed in 10 mL of a 9:1 mixture of DCM and MeOH and sonicated. The solid was filtered off to afford compound **11** as an orange solid (25 mg, 60 μmol, 40%). *R*_*f*_: 0.47 (DCM/MeOH/AcOH = 90:9:1, v/v). ^1^H NMR (400 MHz, DMSO-*d*_6_) δ 8.13 (AA′BB′, 2H), 7.99–7.92 (m, 5H), 7.81 (AA′BB′, 2H), 6.38 (br s, 4H), 3.20 (s, 3H), 1.77 (s, 3H). ^13^C NMR (101 MHz, DMSO-*d*_6_) δ 166.8, 163.5, 162.3, 159.9, 154.3, 151.4, 147.2, 133.2, 130.7, 127.2, 122.9, 122.6, 93.7, 89.0, 82.3, 76.5, 52.1, 31.8. HRMS (ESI^+^) *m*/*z* calcd for [M + H]^+^ (C_22_H_21_N_6_O_3_^+^): 417.1670, found: 417.1675. Mp (dec.): 248 °C.

#### Methyl (*E*)-5-[(4-Acetylphenyl)diazenyl]-2-methylbenzoate (**S18a**)

Methyl 5-amino-2-methylbenzoate (1.9 g, 12 mmol) was reacted following the general procedure A. The residue was purified by flash chromatography (petroleum ether/DCM = 50:50, v/v) to afford compound **S18a** as an orange solid (2.6 g, 8.9 mmol, 76%). *R*_*f*_: 0.22 (petroleum ether/DCM = 50:50, v/v). ^1^H NMR (400 MHz, CDCl_3_) δ 8.50 (d, *J* = 2.1 Hz, 1H), 8.10 (AA′BB′, 2H), 7.96 (m, 3H), 7.40 (d, *J* = 8.2 Hz, 1H), 3.95 (s, 3H), 2.69 (s, 3H), 2.66 (s, 3H). ^13^C NMR (101 MHz, CDCl_3_) δ 197.5, 167.4, 155.0, 150.7, 144.4, 138.6, 132.8, 130.5, 129.5, 126.2, 125.7, 123.1, 52.2, 27.0, 22.0. HRMS (ESI^+^) *m*/*z* calcd for [M + H]^+^ (C_17_H_17_N_2_O_3_^+^): 297.1234, found: 297.1232. Mp: 108–111 °C.

#### Methyl (*E*)-5-[(4-Acetylphenyl)diazenyl]-2-chlorobenzoate (**S18b**)

Methyl 5-amino-2-chlorobenzoate (2.0 g, 11 mmol) was reacted following the general procedure A. The residue was purified by flash chromatography (petroleum ether/EtOAc = 9:1, v/v) to afford compound **S18b** as an orange solid (1.6 g, 5.1 mmol, 47%). *R*_*f*_: 0.36 (petroleum ether/EtOAc = 9:1, v/v). ^1^H NMR (400 MHz, CDCl_3_) δ 8.43 (d, *J* = 2.4 Hz, 1H), 8.11 (d, *J* = 8.6 Hz, 2H), 8.02–7.99 (m, 3H), 7.62 (d, *J* = 8.6 Hz, 1H), 3.99 (s, 3H), 2.67 (s, 3H). ^13^C NMR (101 MHz, CDCl_3_) δ 197.5, 165.6, 154.7, 150.6, 139.0, 137.0, 132.2, 131.0, 129.6, 126.6, 126.3, 123.3, 52.9, 27.0. HRMS (APCI^+^) *m*/*z* calcd for [M + H]^+^ (C_16_H_14_ClN_2_O_3_^+^): 317.0688 (^35^Cl, 75%), 319.0658 (^37^Cl, 25%); found: 317.0694 (^35^Cl, 75%), 319.0660 (^35^Cl, 25%). Mp: 124–126 °C.

#### Methyl (*E*)-4-[(4-Acetylphenyl)diazenyl]-2-methylbenzoate (**S18c**)

Methyl 4-amino-2-methylbenzoate (2.6 g, 16 mmol) was reacted following the general procedure A. The residue was purified by flash chromatography (petroleum ether/DCM = 50:50, v/v) to afford compound **S18c** as an orange solid (3.9 g, 13 mmol, 83%). *R*_*f*_: 0.63 (pentane/EtOAc = 8:2, v/v). ^1^H NMR (400 MHz, CDCl_3_) δ 8.12 (AA′BB′, 2H), 8.07 (d, *J* = 8.3 Hz, 1H), 7.99 (AA′BB′, 2H), 7.82–7.76 (m, 2H), 3.94 (s, 3H), 2.71 (s, 3H), 2.67 (s, 3H). ^13^C NMR (101 MHz, CDCl_3_) δ 197.5, 167.6, 155.0, 154.1, 141.7, 138.9, 132.2, 131.8, 129.5, 126.4, 123.2, 120.1, 52.2, 27. 0, 22.0. HRMS (APCI^+^) *m*/*z* calcd for [M + H]^+^ (C_17_H_17_N_2_O_3_^+^): 297.1234, found: 297.1237. Mp: 99–100 °C.

#### Methyl (*E*)-4-[(4-Acetylphenyl)diazenyl]-2-chlorobenzoate (**S18d**)

Methyl 4-amino-2-chlorobenzoate (3.0 g, 16 mmol) was reacted following the general procedure A. The residue was purified by flash chromatography (Toluene/EtOAc = 98:2, v/v) to afford compound **S18d** as an orange solid (2.0 g, 6.4 mmol, 40%). *R*_*f*_: 0.49 (Toluene/EtOAc = 98:2, v/v). ^1^H NMR (400 MHz, CDCl_3_) δ 8.13 (AA′BB′, 2H), 8.00 (m, 4H), 7.89 (dd, *J* = 8.4, 1.8 Hz, 1H), 3.98 (s, 3H), 2.68 (s, 3H). ^13^C NMR (101 MHz, CDCl_3_) δ 197.4, 165.7, 154.7, 154.3, 139.3, 135.0, 132.4, 132.1, 129.6, 125.0, 123.5, 121.8, 52.8, 27.0. HRMS (ESI^+^) *m*/*z* calcd for [M + H]^+^ (C_16_H_14_ClN_2_O_3_^+^): 317.0688 (^35^Cl, 75%), 319.0658 (^37^Cl, 25%); found: 317.0689 (^35^Cl, 75%), 319.0659 (^37^Cl, 25%). Mp: 123–125 °C.

#### Methyl (*E*)-5-{[4-(2-Methoxy-4-(trimethylsilyl)but-3-yn-2-yl)phenyl]diazenyl}-2-methylbenzoate (**S19a**)

**S18a** (1.6 g, 5.4 mmol) was reacted following the general procedure D. The residue was purified by flash chromatography (petroleum ether/EtOAc = 98:2, v/v) to afford compound **S19a** as an orange solid (1.4 g, 3.4 mmol, 63%). *R*_*f*_: 0.59 (petroleum ether/EtOAc = 9:1, v/v). ^1^H NMR (400 MHz, CDCl_3_) δ 8.48 (d, *J* = 2.1 Hz, 1H), 7.96 (dd, *J* = 8.2, 2.1 Hz, 1H), 7.92 (AA′BB′, 2H), 7.74 (AA′BB′, 2H), 7.39 (d, *J* = 8.2 Hz, 1H), 3.95 (s, 3H), 3.25 (s, 3H), 2.69 (s, 3H), 1.74 (s, 3H), 0.27 (s, 9H). ^13^C NMR (101 MHz, CDCl_3_) δ 167.6, 152.2, 150.8, 145.9, 143.5, 132.7, 130.5, 127.0, 125.9, 125.5, 122.9, 105.0, 92.8, 76.7, 52.7, 52.2, 32.6, 21.9, 0.1. HRMS (ESI^+^) *m*/*z* calcd for [M + H]^+^ (C_23_H_29_N_2_O_3_Si^+^): 409.1942, found: 409.1950. Mp < 50 °C.

#### Methyl (*E*)-2-Chloro-5-{[4-(2-methoxy-4-(trimethylsilyl)but-3-yn-2-yl)phenyl]diazenyl}benzoate (**S19b**)

**S18b** (0.60 g, 1.9 mmol) was reacted following the general procedure D. The residue was purified by flash chromatography (petroleum ether/EtOAc = 9:1, v/v) to afford compound **S19b** as an orange solid (0.15 g, 0.36 mmol, 19%). *R*_*f*_: 0.68 (petroleum ether/EtOAc = 9:1, v/v). ^1^H NMR (400 MHz, CDCl_3_) δ 8.39 (d, *J* = 2.4 Hz, 1H), 7.97 (dd, *J* = 8.5, 2.4 Hz, 1H), 7.92 (AA′BB′, 2H), 7.75 (AA′BB′, 2H), 7.59 (d, *J* = 8.5 Hz, 1H), 3.98 (s, 3H), 3.25 (s, 3H), 1.74 (s, 3H), 0.27 (s, 9H). ^13^C NMR (101 MHz, CDCl_3_) δ 165.7, 152.0, 150.8, 146.6, 136.2, 132.1, 130.9, 127.1, 126.3, 126.2, 123.1, 104.8, 92.9, 76.7, 52.8, 52.7, 32.6, 0.1. HRMS (ESI^+^) *m*/*z* calcd for [M + H]^+^ (C_22_H_26_ClN_2_O_3_Si^+^): 429.1396 (^35^Cl, 75%), 431.1366 (^37^Cl, 25%); found: 429.1391 (^35^Cl, 75%), 431.1350 (^37^Cl, 25%). Mp: 55–57 °C.

#### Methyl (*E*)-4-{[4-(2-Methoxy-4-(trimethylsilyl)but-3-yn-2-yl)phenyl]diazenyl}-2-methylbenzoate (**S19c**)

**S18c** (0.90 g, 3.0 mmol) was reacted following the general procedure D. The residue was purified by flash chromatography (petroleum ether/EtOAc = 98:2, v/v) to afford compound **S19c** as an orange oil (0.55 g, 1.4 mmol, 45%). *R*_*f*_: 0.49 (petroleum ether/EtOAc = 98:2, v/v). ^1^H NMR (400 MHz, CDCl_3_) δ 8.06 (d, *J* = 8.2 Hz, 1H), 7.93 (AA′BB′, 2H), 7.75 (m, 4H), 3.93 (s, 3H), 3.25 (s, 3H), 2.71 (s, 3H), 1.74 (s, 3H), 0.27 (s, 9H). ^13^C NMR (101 MHz, CDCl_3_) δ 167.7, 154.3, 152.3, 146.4, 141.7, 131.8, 131.5, 127.1, 126.0, 123.1, 119.9, 104.9, 92.9, 76.7, 52.7, 52.2, 32.6, 22.0, 0.1. HRMS (ESI^+^) *m*/*z* calcd for [M + H]^+^ (C_23_H_29_N_2_O_3_Si^+^): 409.1942, found: 409.1944. Mp: 69–72 °C.

#### Methyl (*E*)-2-Chloro-4-{[4-(2-methoxy-4-(trimethylsilyl)but-3-yn-2-yl)phenyl]diazenyl}benzoate (**S19d**)

**S18d** (1.6 g, 5.1 mmol) was reacted following the general procedure D. The residue was purified by flash chromatography (petroleum ether/EtOAc = 97:3, v/v) to afford compound **S19d** as an orange solid (0.55 g, 1.3 mmol, 25%). *R*_*f*_: 0.43 (petroleum ether/EtOAc = 96:4, v/v). ^1^H NMR (400 MHz, CDCl_3_) δ 8.02–7.97 (m, 2H), 7.94 (AA′BB′, 2H), 7.85 (dd, *J* = 8.2, 1.9 Hz, 1H), 7.76 (AA′BB′, 2H), 3.97 (s, 3H), 3.26 (s, 3H), 1.74 (s, 3H), 0.27 (s, 9H). ^13^C NMR (101 MHz, CDCl_3_) δ 165.8, 154.6, 152.0, 147.1, 134.9, 132.4, 131.4, 127.1, 124.7, 123.4, 121.6, 104.8, 93.0, 76.7, 52.8, 52.7, 32.6, 0.1. HRMS (ESI^+^) *m*/*z* calcd for [M + H]^+^ (C_22_H_26_ClN_2_O_3_Si^+^): 429.1396 (^35^Cl, 75%), 431.1366 (^37^Cl, 25%); found: 429.1394 (^35^Cl, 75%), 431.1356 (^37^Cl, 25%). Mp: 50–52 °C.

#### Methyl (*E*)-5-{[4-(2-Methoxybut-3-yn-2-yl)phenyl]diazenyl}-2-methylbenzoate (**S20a**)

**S19a** (1.0 g, 2.5 mmol) was reacted following the general procedure B. Purification by flash chromatography was not required. The product **S20a** was obtained as an orange solid (0.71 g, 2.1 mmol, 83%). *R*_*f*_: 0.36 (petroleum ether/EtOAc = 96:4, v/v). ^1^H NMR (400 MHz, CDCl_3_) δ 8.48 (d, *J* = 2.1 Hz, 1H), 7.96 (dd, *J* = 8.2, 2.1 Hz, 1H), 7.92 (AA′BB′, 2H), 7.76 (AA′BB′, 2H), 7.39 (d, *J* = 8.2 Hz, 1H), 3.95 (s, 3H), 3.27 (s, 3H), 2.78 (s, 1H), 2.69 (s, 3H), 1.78 (s, 3H). ^13^C NMR (101 MHz, CDCl_3_) δ 167.6, 152.3, 150.8, 145.5, 143.6, 132.7, 130.5, 127.0, 125.9, 125.5, 123.0, 83.4, 76.4, 76.0, 52.8, 52.2, 32.6, 21.9. HRMS (ESI^+^) *m*/*z* calcd for [M + H]^+^ (C_20_H_21_N_2_O_3_^+^): 337.1547, found: 337.1553. Mp: 90–92 °C.

#### Methyl (*E*)-2-Chloro-5-{[4-(2-methoxybut-3-yn-2-yl)phenyl]diazenyl}benzoate (**S20b**)

**S19b** (0.14 g, 0.32 mmol) was reacted following the general procedure B. Purification by flash chromatography was not required. The product **S20b** was obtained as an orange solid (99 mg, 0.28 mmol, 88%). *R*_*f*_: 0.65 (petroleum ether/EtOAc = 8:2, v/v). ^1^H NMR (400 MHz, CDCl_3_) δ 8.39 (d, *J* = 2.4 Hz, 1H), 7.97 (dd, *J* = 8.5, 2.4 Hz, 1H), 7.92 (AA′BB′, 2H), 7.76 (AA′BB′, 2H), 7.60 (d, *J* = 8.5 Hz, 1H), 3.98 (s, 3H), 3.27 (s, 3H), 2.78 (s, 1H), 1.77 (s, 3H). ^13^C NMR (101 MHz, CDCl_3_) δ 165.7, 152.1, 150.8, 146.1, 136.2, 132.1, 130.9, 127.0, 126.3, 126.2, 123.2, 83.3, 76.4, 76.1, 52.8, 32.6. HRMS (ESI^+^) *m*/*z* calcd for [M + H]^+^ (C_19_H_18_ClN_2_O_3_^+^): 357.1000 (^35^Cl, 75%), 359.0971 (^37^Cl, 25%); found: 357.1003 (^35^Cl, 75%), 359.0968 (^37^Cl, 25%). Mp: 67–68 °C.

#### Methyl (*E*)-4-{[4-(2-Methoxybut-3-yn-2-yl)phenyl]diazenyl}-2-methylbenzoate (**S20c**)

**S19c** (0.50 g, 1.2 mmol) was reacted following the general procedure B. The residue was purified by flash chromatography (petroleum ether/EtOAc = 98:2, v/v) to afford compound **S20c** as an orange solid (0.38 mg, 1.1 mmol, 92%). *R*_*f*_: 0.40 (petroleum ether/EtOAc = 98:2, v/v). ^1^H NMR (400 MHz, CDCl_3_) δ 8.06 (d, *J* = 8.2 Hz, 1H), 7.96–7.90 (m, 2H), 7.79–7.72 (m, 4H), 3.93 (s, 3H), 3.27 (s, 3H), 2.78 (s, 1H), 2.71 (s, 3H), 1.78 (s, 3H). ^13^C NMR (101 MHz, CDCl_3_) δ 167.7, 154.3, 152.4, 145.9, 141.7, 131.8, 131.5, 127.0, 126.1, 123.2, 119.9, 83.3, 76.4, 76.1, 52.8, 52.2, 32.6, 22.0. HRMS (ESI^+^) *m*/*z* calcd for [M + H]^+^ (C_20_H_21_N_2_O_3_^+^): 337.1547, found: 337.1551. Mp: 68–72 °C.

#### (*E*)-2-Chloro-4-{[4-(2-methoxybut-3-yn-2-yl)phenyl]diazenyl}benzoic acid (**S22**)

**S19d** (0.60 g, 1.4 mmol) was reacted following the general procedure B. The residue was purified by flash chromatography (DCM/MeOH/AcOH = 90:9:1, v/v) to afford compound **S22** as an orange solid (0.31 mg, 0.89 mmol, 63%). *R*_*f*_: 0.78 (DCM/MeOH/AcOH = 90:9:1, v/v). ^1^H NMR (400 MHz, CDCl_3_) δ 8.19 (d, *J* = 8.3 Hz, 1H), 8.02 (d, *J* = 1.7 Hz, 1H), 7.96 (AA′BB′, 2H), 7.89 (dd, J = 8.3, 1.8 Hz, 1H), 7.79 (AA′BB′, 2H), 3.29 (s, 3H), 2.79 (s, 1H), 1.78 (s, 3H). ^13^C NMR (101 MHz, CDCl_3_) δ 169.5, 155.1, 152.1, 146.8, 135.9, 133.4, 129.9, 127.1, 125.1, 123.5, 121.6, 121.1, 83.2, 76.4, 76.3, 76.2, 52.9, 32.6, 29.9. HRMS (ESI^–^) *m*/*z* calcd for [M-H]^−^ (C_18_H_14_ClN_2_O_3_^–^): 341.0688 (^35^Cl, 75%), 343.0658 (^37^Cl, 25%); found: 341.0696 (^35^Cl, 75%), 343.0665 (^37^Cl, 25%). Mp: 160–164 °C.

#### Methyl (*E*)-5-{[4-(4-(2,4-Diaminopyrimidin-5-yl)-2-methoxybut-3-yn-2-yl)phenyl]diazenyl}-2-methylbenzoate (**S21a**)

**S20a** (0.59 g, 1.7 mmol) was reacted following the general procedure C. The residue was purified by flash chromatography (DCM/MeOH = 97:3, v/v) to afford compound **S21a** as an orange solid (0.57 g, 1.28 mmol, 45%). *R*_*f*_: 0.45 (DCM/MeOH = 97:3, v/v). ^1^H NMR (400 MHz, DMSO-*d*_6_) δ 8.30 (d, *J* = 2.3 Hz, 1H), 7.99 (m, 2H), 7.93 (d, *J* = 8.7 Hz, 2H), 7.81 (d, *J* = 8.7 Hz, 2H), 7.55 (d, *J* = 8.2 Hz, 1H), 6.41 (br s, 4H), 3.88 (s, 3H), 3.22 (s, 3H), 2.60 (s, 3H), 1.79 (s, 3H). ^13^C NMR (101 MHz, DMSO-*d*_6_) δ 166.6, 163.5, 162.4, 160.1, 151.3, 149.9, 146.7, 142.9, 133.0, 130.3, 127.0, 125.9, 123.9, 122.7, 93.6, 88.9, 82.3, 76.4, 52.2, 52.0, 31.8, 21.1. HRMS (ESI^+^) *m*/*z* calcd for [M + H]^+^ (C_24_H_25_N_6_O_3_^+^): 445.1983, found: 445.1986. Mp: 173–175 °C.

#### Methyl (*E*)-2-Chloro-5-{[4-(4-(2,4-diaminopyrimidin-5-yl)-2-methoxybut-3-yn-2-yl)phenyl]diazenyl}benzoate (**S21b**)

**S20b** (96 mg, 0.27 mmol) was reacted following the general procedure C. The residue was purified by flash chromatography (DCM/MeOH = 97:3, v/v) to afford compound **S21b** as an orange solid (45 mg, 97 μmol, 41%). *R*_*f*_: 0.37 (DCM/MeOH = 96:4, v/v). ^1^H NMR (400 MHz, CDCl_3_) δ 8.40 (d, *J* = 2.4 Hz, 1H), 8.14 (s, 1H), 7.98 (dd, *J* = 8.6, 2.4 Hz, 1H), 7.94 (AA′BB′, 2H), 7.78 (AA′BB′, 2H), 7.60 (d, *J* = 8.6 Hz, 1H), 5.21 (br s, 2H), 5.02 (br s, 2H), 3.99 (s, 3H), 3.32 (s, 3H), 1.85 (s, 3H). ^13^C NMR (101 MHz, CDCl_3_) δ 165.7, 163.9, 162.0, 160.4, 152.1, 150.7, 146.6, 136.3, 132.1, 131.0, 127.0, 126.3, 126.2, 123.3, 95.6, 91.8, 81.2, 52.9, 52.8, 32.7. HRMS (ESI^+^) *m*/*z* calcd for [M + H]^+^ (C_23_H_22_ClN_6_O_3_^+^): 465.1436 (^35^Cl, 75%), 467.1407 (^37^Cl, 25%); found: 465.1438 (^35^Cl, 75%), 467.1404 (^37^Cl, 25%). Mp: 152–153 °C.

#### Methyl (*E*)-4-{[4-(4-(2,4-Diaminopyrimidin-5-yl)-2-methoxybut-3-yn-2-yl)phenyl]diazenyl}-2-methylbenzoate (**S21c**)

**S20c** (0.23 g, 0.68 mmol) was reacted following the general procedure C. The residue was purified by flash chromatography (DCM/MeOH = 97:3, v/v) to afford compound **S21c** as an orange solid (0.17 g, 0.38 mmol, 62%). *R*_*f*_: 0.47 (DCM/MeOH = 96:4, v/v). ^1^H NMR (400 MHz, CDCl_3_) δ 8.10 (s, 1H), 8.06 (d, *J* = 8.2 Hz, 1H), 7.95 (AA′BB′, 2H), 7.80–7.71 (m, 4H), 5.47 (br s, 4H), 3.93 (s, 3H), 3.31 (s, 3H), 2.71 (s, 3H), 1.85 (s, 3H). ^13^C NMR (101 MHz, CDCl_3_) δ 167.7, 164.0, 154.3, 152.4, 146.3, 141.7, 131.8, 131.6, 126.9, 126.1, 123.3, 119.9, 95.9, 92.1, 80.7, 52.9, 52.2, 32.7, 22.0. HRMS (ESI^+^) *m*/*z* calcd for [M + H]^+^ (C_24_H_25_N_6_O_3_^+^): 445.1983, found: 445.1991. Mp (dec.): 165 °C.

#### (*E*)-5-{[4-(4-(2,4-Diaminopyrimidin-5-yl)-2-methoxybut-3-yn-2-yl)phenyl]diazenyl}-2-methylbenzoic acid (**12**)

**S21a** (0.30 g, 0.68 mmol) was reacted following the general procedure E. The residue was purified by flash chromatography (DCM/MeOH/AcOH = 90:9:1, v/v) to afford compound **12** as an orange solid (56 mg, 0.13 mmol, 20%). *R*_*f*_: 0.70 (DCM/MeOH/AcOH = 90:9:1, v/v). ^1^H NMR (400 MHz, DMSO-*d*_6_) δ 8.28 (d, *J* = 2.0 Hz, 1H), 7.98–7.90 (m, 4H), 7.79 (AA′BB′, 2H), 7.51 (d, *J* = 8.3 Hz, 1H), 6.38 (br s, 4H), 3.20 (s, 3H), 2.60 (s, 3H), 1.77 (s, 3H). ^13^C NMR (101 MHz, DMSO-*d*_6_) δ 168.3, 163.5, 162.4, 160.1, 151.3, 149.9, 146.5, 142.7, 132.8, 132.0, 127.1, 125.4, 124.0, 122.6, 93.7, 88.9, 82.3, 76.5, 52.0, 31.8, 21.3. HRMS (ESI^+^) *m*/*z* calcd for [M + H]^+^ (C_23_H_23_N_6_O_3_^+^): 431.1826, found: 431.1830. Mp (dec.): 221 °C.

#### (*E*)-2-Chloro-5-{[4-(4-(2,4-diaminopyrimidin-5-yl)-2-methoxybut-3-yn-2-yl)phenyl]diazenyl}benzoic acid (**13**)

**S21b** (24 mg, 52 μmol) was reacted following general procedure E. The residue was purified by flash chromatography (DCM/MeOH/AcOH = 90:9:1, v/v) to afford compound **13** as an orange solid (18 mg, 40 μmol, 77%). *R*_*f*_: 0.60 (DCM/MeOH/AcOH = 90:9:1, v/v). ^1^H NMR (400 MHz, DMSO-*d*_6_) δ 8.18 (d, *J* = 2.3 Hz, 1H), 8.02–7.92 (m, 4H), 7.82 (AA′BB′, 2H), 7.73 (d, *J* = 8.5 Hz, 1H), 6.46 (br s, 4H), 3.22 (s, 3H), 1.79 (s, 3H). ^13^C NMR (101 MHz, DMSO-*d*_6_) δ 166.8, 163.5, 162.1, 159.7, 151.2, 150.1, 147.0, 134.8, 134.0, 131.7, 127.1, 125.4, 123.9, 122.8, 93.6, 89.0, 82.2, 76.5, 52.1, 31.8. HRMS (ESI^+^) *m*/*z* calcd for [M + H]^+^ (C_22_H_20_ClN_6_O_3_^+^): 451.1280 (^35^Cl, 75%), 453.1250 (^37^Cl, 25%); found: 451.1279 (^35^Cl, 75%), 453.1246 (^37^Cl, 25%). Mp (dec.): 212 °C.

#### (*E*)-4-{[4-(4-(2,4-Diaminopyrimidin-5-yl)-2-methoxybut-3-yn-2-yl)phenyl]diazenyl}-2-methylbenzoic acid (**14**)

**S21c** (61 mg, 0.14 mmol) was reacted following general procedure E. The residue was purified by flash chromatography (DCM/MeOH/AcOH = 90:9:1, v/v) to afford compound **14** as an orange solid (22 mg, 51 μmol, 37%). *R*_*f*_: 0.79 (DCM/MeOH/AcOH = 90:9:1, v/v). ^1^H NMR (400 MHz, DMSO-*d*_6_) δ 8.04–7.92 (m, 4H), 7.84–7.71 (m, 4H), 6.62 (br s, 4H), 3.21 (s, 3H), 2.62 (s, 3H), 1.78 (s, 3H). ^13^C NMR (101 MHz, DMSO-*d*_6_) δ 168.2, 163.5, 161.5, 158.6, 153.2, 151.5, 146.9, 140.6, 133.0, 131.5, 127.2, 125.7, 122.8, 119.5, 93.8, 89.3, 81.8, 76.5, 52.1, 31.8, 21.3. HRMS (ESI^+^) *m*/*z* calcd for [M + H]^+^ (C_23_H_23_N_6_O_3_^+^): 431.1826, found: 431.1833. Mp (dec.): 240 °C.

#### (*E*)-2-Chloro-4-{[4-(4-(2,4-diaminopyrimidin-5-yl)-2-methoxybut-3-yn-2-yl)phenyl]diazenyl}benzoic acid (**15**)

**S22** (0.28 g, 0.80 mmol) was reacted following the general procedure C. The residue was purified by preparative HPLC to afford compound **15** as an orange solid (0.17 g, 0.37 mmol, 52%). *R*_*f*_: 0.58 (DCM/MeOH/AcOH = 90:9:1, v/v). ^1^H NMR (400 MHz, DMSO-*d*_6_) δ 8.02–7.92 (m, 5H), 7.91 (dd, *J* = 8.1, 1.9 Hz, 1H), 7.87–7.80 (m, 2H), 6.44 (br s, 4H), 3.22 (s, 3H), 1.79 (s, 3H). ^13^C NMR (101 MHz, DMSO-*d*_6_) δ 166.4, 163.5, 162.2, 159.8, 153.3, 151.2, 147.5, 134.2, 132.6, 131.8, 127.2, 123.5, 123.0, 121.7, 93.6, 89.0, 82.3, 76.4, 52.1, 31.7. HRMS (ESI^+^) *m*/*z* calcd for [M + H]^+^ (C_22_H_20_ClN_6_O_3_^+^): 451.1280 (^35^Cl, 75%), 453.1250 (^37^Cl, 25%); found: 451.1276 (^35^Cl, 75%), 453.1242 (^37^Cl, 25%). Mp (dec.): 245 °C.

### Photochemical Characterization

UV–vis absorption spectra were recorded on an Agilent 8453 UV–vis Spectrophotometer. Photochemical isomerization (photoswitching) was achieved by irradiation from the side in a fluorescence quartz cuvette (width = 1.0 cm) using a custom-built (Prizmatix/Mountain Photonics) multiwavelength fiber coupled LED system (FC6-LED-WL) including the following LEDs: 365A, 390B, 420Z, 445B, 535R, 630CA. A detailed description of the setup was published by us recently.^84^ Alternatively, the samples were irradiated at 340 nm using the LOT Quantum design setup with 300 W Xe-Arc lamp, using the Thorlabs FB340-10 Bandpass filter. A Quantum Northwest TC1 temperature controller was used to maintain the temperature at 25 °C during photochemical studies. To analyze the fatigue resistance of the compounds, repeated irradiation cycles were performed using alternating wavelengths. The decrease and increase of the π–π* transition band was followed by UV–vis spectroscopy (Agilent 8453) at 25 °C while stirring. To determine the thermal lifetime for the metastable *cis* isomer, samples were irradiated with the custom-built setup until photostationary state (PSS) was reached. Then, the cuvettes were placed in a JASCO V750 spectrophotometer with a PTC 424*S*/15 temperature controller, and the recovery of the π–π* transition was followed while stirring at 25 °C (for solutions in DMSO and eDHFR assay buffer) or 37 °C (for solutions in 10 mM Tris-HCl buffer pH 7.0). The data were fitted using a first-order exponential function in OriginPro 2018. For the PSD determination by ^1^H NMR spectroscopy, 0.5 mL of a 2 mM solution of the respective compound in DMSO-*d*_6_ was thermally equilibrated by briefly (∼10 s) heating the sample with a heat gun. Subsequently, the sample was irradiated in a glass NMR tube with a 365 nm hand-held lamp (Spectroline ENB-280C) or the LEDs of the custom-built setup. Detailed data are reported in the SI.

### *In Vitro* Pharmacological Characterization

#### Overexpression of *E. coli* DHFR (eDHFR) Protein

The pT7-SC1 plasmid containing the eDHFR genes were transformed into E. cloni EXPRESS BL21 (DE3) cells (Lucigen), and transformants were selected on LB agar plates supplemented with 100 μg/mL ampicillin after overnight growth at 37 °C. The resulting colonies were grown at 37 °C in 2xYT medium supplemented with 100 μg/mL ampicillin until the optical density at 600 nm (OD_600_) reached ∼0.8 (200 rpm shaking). The eDHFR expression was subsequently induced by addition of 0.5 mM IPTG (isopropylβ-d-1-thiogalactopyranoside), and the temperature was switched to 25 °C for overnight growth (200 rpm shaking). The next day the bacteria were harvested by centrifugation at 6000×*g* at 4 °C for 25 min, and the resulting pellets were frozen at 80 °C until further use.

#### Purification of eDHFR Protein

Bacterial pellets originating from 50 mL culture were resuspended in 30 mL lysis buffer (150 mM NaCl, 15 mM Tris-HCl pH 7.5, 1 mM MgCl2, 0.2 units/mL DNaseI, 10 μg/mL lysozyme) and incubated at 37 °C for 20 min. After further disruption of the bacteria by probe sonication, the crude lysate was clarified by centrifugation at 6000×*g* at 4 °C for 30 min. After lysis of the bacteria by probe sonication, the crude lysates were clarified by centrifugation at 6000×*g* for 20 min at 4 °C, and the supernatant was mixed with 200 μL of Ni-NTA resin (Qiagen) equilibrated in wash buffer. After 1 h, the resin was loaded into a column (Micro Bio Spin, Bio-Rad) and washed with ∼5 mL of the wash buffer. eDHFR was eluted with approximately ∼0.5 mL of wash buffer containing 500 mM ethylenediaminetetraacetic acid.

#### Preparation of Inhibitor Solutions

A 10 mM stock solution of the inhibitor in DMSO was divided into two parts. One half was thermally adapted with a heat gun for 1 min to obtain only the *trans* isomer. To reach the PSS, the second half of the stock solution was diluted to 100 μM and then irradiated with light for 15–45 min, in a 10 mm quartz cuvette under constant stirring.

#### eDHFR Inhibition Assay

The compounds were tested as eDHFR inhibitors via a colorimetric assay (Sigma-Aldrich, Catalog No. CS0340). Instead of the human DHFR protein provided in the kit, the in-house purified eDHFR was used. In a 3 mL cuvette, eDHFR (∼20 ng) was incubated with NADPH (60 μM, final) for 3 min, followed by an incubation with the inhibitor (from 10^–5^ to 10^–12^ M, final) for an additional 7 min. The obtained solution was used as the blank for UV–vis spectroscopy (Agilent 8453). Then, dihydrofolic acid (15 μM, final) was added to the blank, and the absorbance at 340 nm was monitored every 5 s for 300 s at 25 °C. The concentration of DMSO in the final volume was 1% v/v for all enzymatic reactions. The measurements were performed in triplicates. For the control enzymatic reactions, eDHFR was incubated with NADPH and DMSO (1% v/v, final). In one case (compound **1**), it was necessary to perform the inhibition assay under continuous irradiation with λ_1_ = 390 nm light because of the extremely short thermal half-life of the compound (*t*_1/2_ = 10 s). The related control enzymatic reaction was also performed under continuous irradiation with λ_1_ = 390 nm light. Linear regression was performed in Microsoft Excel to calculate ΔAbs/s. % eDHFR activity was calculated as the ratio between ΔAbs/s for the sample and ΔAbs/s for the control reaction. GraphPadPrism 5.0 (GraphPad Software, Inc., version 5.00) was used for the determination of the IC_50_ of each inhibitor. Nonlinear regression was used for data fitting. The statistical significance of the difference in activity between the isomers was checked with the extra sum-of-squares F-test. Detailed data are reported in the SI.

### Antibacterial Activity Assay

A culture of *E. coli* CS1562 was grown overnight in LB medium (10 g/L tryptone, 5 g/L yeast extract, 5 g/L NaCl) containing tetracycline (10 μg/mL) at 37 °C. The OD_600_ was measured, and the culture was diluted to 0.002 OD_600_ in LB medium. Series dilutions of the compounds were prepared in DMSO and diluted 25 times in sterile LB medium in a black sterile Eppendorf tube. For the irradiated samples, triplicates containing 100 μL of this mixture were pipetted into a transparent sterile 96-well plate and irradiated (λ = 355 nm, 30 min, plate irradiation setup). Subsequently, the dark samples were added to the 96-well plate (100 μL, triplicates). Lastly, the LB medium containing *E. coli* CS1562 at 0.002 OD was added to every well (100 μL). The cells were grown overnight at 37 °C in a plate reader (Synergy H1, Biotek), and the OD_600_ was measured every 10 min with a 10 s shaking step before each measurement. The OD_600_ curves were background-corrected by subtracting the value at *t* = 0. MIC values were determined at 24 h of incubation. Detailed data are reported in the SI.

### Molecular Modeling

#### General remarks

The calculations and analysis were performed either on a HP EliteDesk, with an Intel Core i7-6700 processor with four cores and an NVIDIA GeForce GTX 1060 3GB graphics card (Molecular docking) or on the Peregrine cluster at the University of Groningen (Molecular Dynamics). The substructure search in the Cambridge Structural Database (CSD) was performed on ConQuest (as of July 2021, ver 2.0.5, Cambridge Crystallographic Data Centre). The measurements were handled through Mercury (ver 4.3.1, Cambridge Crystallographic Data Centre) and Excel 2019 (Microsoft). The docking calculations and the MD simulations were carried out with Maestro (ver 12.4, Schrödinger Release 2020-2: Maestro, Schrödinger, LLC, New York, NY, 2020). Detailed data are reported in the SI.

#### Geometry Measurements from the CSD

All ring atoms were set as “any” to allow all possible heterocycles and substitutions, and the linker atoms were set as “acyclic”. CSD search parameters: 3D coordinates determined, *R* factor ≤0.10, only not disordered, no errors, not polymeric, no ions, only organic. 1706 structures were found.

#### Molecular Docking

The protein (PDB ID: 3DAU,^13^ Chain: A) was prepared through the Protein Preparation Wizard in Maestro, performing the assignment of bond orders, hydrogen addition, hydrogen bonds definition and optimization, removal of water molecules and ions, and restrained minimization with the OPLS3e force field.^85^ All water molecules were removed. LigPrep was used to prepare the ligands and to generate possible states at pH 7.0 ± 2.0 with Epik. The ligands were docked with Induced Fit Docking XP^43,86^ with the standard protocol. The ligand was picked to define the centroid of the receptor box, the option “enhance planarity of conjugated pi groups” was selected, and the side chains were trimmed based on their B-factor. All docking pose images were obtained with Pymol (The PyMOL Molecular Graphics System, ver 2.2.3 Schrödinger, LLC). The cocrystallized ligand was redocked with RMSD = 0.8 Å. When the ligand featured a chiral center (hypothesis 2–5), the calculations were performed on the *R* enantiomer.

#### MD Simulations

The crystal or docking structures were embedded in a orthorhombic box of ca. 11,000 TIP3P^87^ water molecules with 0.15 M NaCl, and the dimension of the box was ca. 100 × 100 × 100 Å. The net charge of the system was neutralized by addition of sodium ions to the solvent box. The total number of atoms was ca. 40,000 atoms. The simulations were performed with the Desmond molecular dynamics package,^88^ with default settings for bond constrains, van der Waals and electrostatic interactions cutoffs, and PME method^89^ for long-range electrostatic interactions. Each system was subjected to the following relaxation and equilibration protocol: 100 ps of Brownian dynamics at 10 K in an NVT ensemble with harmonic restraints (50 kcal/mol/A^2^) on the solutes’ heavy atoms, followed by 12 ps in an NVT ensemble (Berendsen thermostat)^90^ at 10 K and retaining harmonic restraints on the solutes’ heavy atoms, followed by 12 ps in an NPT ensemble (Berendsen thermostat and barostat) at 10 K and retaining harmonic restraints on the solutes’ heavy atoms, followed by 24 ps in an NPT ensemble (Berendsen thermostat and barostat) at 300 K and retaining harmonic restraints on the solutes’ heavy atoms, followed by 24 ps in an NPT ensemble (Berendsen thermostat and barostat) at 300 K without harmonic restraints on the solutes’ heavy atoms. The production simulations were run for 100 ns in an NPT ensemble (300 K, 1 bar, Martyna–Tobias–Klein barostat and Nose–Hoover thermostat),^91,92^ in three replicas.^93^ Coordinates were saved every 100 ps and analyzed in Maestro to compute the following properties over the trajectories: RMSD of the ligand heavy atoms after least-squares fitting to the protein heavy atoms, RMSF of ligands, SASA of ligands and selected residues, atom distances for the identification of salt bridges. The salt bridge cutoff is defined as a distance of 5 Å between the centroids of side-chain charged groups (carboxylate and guanidine).^94^ This less strict criterion allows to include different interaction geometries (standard salt bridges and N–O bridges).

## Supporting Information Available

Synthetic schemes, synthetic procedures for literature compounds, photophysical characterization of compounds **1**–**15** by UV–vis and ^1^H NMR spectroscopy (photoisomerization, fatigue studies, thermal *cis*-to-*trans* isomerization, PSD determination), protein and DNA sequence of the expressed eDHFR, inhibitory activities of compounds **1**–**15** against eDHFR, antibacterial activities of compounds **6**–**15** against *E. coli* CS1562 and bacterial growth curves, ring angles measurements from the CSD, molecular docking poses, analysis of MD simulations (RMSD, interaction distances, ring angles, SASA, RMSF), physicochemical descriptors, UHPLC-MS purity analysis of final compounds, and copies of ^1^H NMR, ^13^C NMR and HRMS data for all isolated compounds (PDF)
PDB files with the predicted binding mode of compounds **1**–**15** (ZIP)
Molecular formula strings and associated biological data (CSV)

## Abbreviations Used

CRY1: cryptochrome-1
DHFR: dihydrofolate reductase
DMTA: design–make–test–analyze
DMSTA: design–make–switch–test–analyze
GSH: glutathione
IFD: induced fit docking
LB: Luria broth
PLA: propargyl-linked antifolate
PMAT: *para*-methoxyazobenzene trimethoprim
PSD: photostationary state distribution
PSS: photostationary state
RMSD: root-mean-square deviation
RMSF: root-mean-square fluctuation
SASA: solvent accessible surface area
TCAT: tetra-*ortho*-chloroazobenzene trimethoprim
TFAT: tetra-*ortho*-fluoroazobenzene trimethoprim
TMP: trimethoprim

## References

[ref1] Velema W. A.; Szymanski W.; Feringa B. L. Photopharmacology: Beyond Proof of Principle. J. Am. Chem. Soc. 2014, 136 (6), 2178–2191. 10.1021/ja413063e. 24456115

[ref2] Broichhagen J.; Frank J. A.; Trauner D. A Roadmap to Success in Photopharmacology. Acc. Chem. Res. 2015, 48 (7), 1947–1960. 10.1021/acs.accounts.5b00129. 26103428

[ref3] Lerch M. M.; Hansen M. J.; van Dam G. M.; Szymanski W.; Feringa B. L. Emerging Targets in Photopharmacology. Angew. Chem., Int. Ed. 2016, 55 (37), 10978–10999. 10.1002/anie.201601931. 27376241

[ref4] Hüll K.; Morstein J.; Trauner D. In Vivo Photopharmacology. Chem. Rev. 2018, 118 (21), 10710–10747. 10.1021/acs.chemrev.8b00037. 29985590

[ref5] Beharry A. A.; Woolley G. A. Azobenzene Photoswitches for Biomolecules. Chem. Soc. Rev. 2011, 40 (8), 4422–4437. 10.1039/c1cs15023e. 21483974

[ref6] Fuchter M. J. On the Promise of Photopharmacology Using Photoswitches: A Medicinal Chemist’s Perspective. J. Med. Chem. 2020, 63 (20), 11436–11447. 10.1021/acs.jmedchem.0c00629. 32511922

[ref7] Velema W. A.; Van Der Berg J. P.; Hansen M. J.; Szymanski W.; Driessen A. J. M.; Feringa B. L. Optical Control of Antibacterial Activity. Nat. Chem. 2013, 5 (11), 924–928. 10.1038/nchem.1750. 24153369

[ref8] Wegener M.; Hansen M. J.; Driessen A. J. M.; Szymanski W.; Feringa B. L. Photocontrol of Antibacterial Activity: Shifting from UV to Red Light Activation. J. Am. Chem. Soc. 2017, 139 (49), 17979–17986. 10.1021/jacs.7b09281. 29136373 PMC5730949

[ref9] Velema W. A.; Hansen M. J.; Lerch M. M.; Driessen A. J. M.; Szymanski W.; Feringa B. L. Ciprofloxacin-Photoswitch Conjugates: A Facile Strategy for Photopharmacology. Bioconjugate Chem. 2015, 26 (12), 2592–2597. 10.1021/acs.bioconjchem.5b00591. 26574623

[ref10] Babii O.; Afonin S.; Berditsch M.; Reier S.; Mykhailiuk P. K.; Kubyshkin V. S.; Steinbrecher T.; Ulrich A. S.; Komarov I. V. Controlling Biological Activity with Light: Diarylethene-Containing Cyclic Peptidomimetics. Angew. Chem., Int. Ed. 2014, 53 (13), 3392–3395. 10.1002/anie.201310019. 24554486

[ref11] Li Z.; Wang Y.; Li M.; Zhang H.; Guo H.; Ya H.; Yin J. Synthesis and Properties of Dithienylethene-Functionalized Switchable Antibacterial Agents. Org. Biomol. Chem. 2018, 16 (38), 6988–6997. 10.1039/C8OB01824C. 30229787

[ref12] Welleman I. M.; Hoorens M. W. H.; Feringa B. L.; Boersma H. H.; Szymański W. Photoresponsive Molecular Tools for Emerging Applications of Light in Medicine. Chem. Sci. 2020, 11 (43), 11672–11691. 10.1039/D0SC04187D. 34094410 PMC8162950

[ref13] Bennett B. C.; Wan Q.; Ahmad M. F.; Langan P.; Dealwis C. G. X-Ray Structure of the Ternary MTX·NADPH Complex of the Anthrax Dihydrofolate Reductase: A Pharmacophore for Dual-Site Inhibitor Design. J. Struct. Biol. 2009, 166 (2), 162–171. 10.1016/j.jsb.2009.01.001. 19374017 PMC2738603

[ref14] Weissleder R.; Ntziachristos V. Shedding Light onto Live Molecular Targets. Nat. Med. 2003, 9 (1), 123–128. 10.1038/nm0103-123. 12514725

[ref15] Yun S. H.; Kwok S. J. J. Light in Diagnosis, Therapy and Surgery. Nat. Biomed. Eng. 2017, 1 (1), 8 10.1038/s41551-016-0008. PMC5476943 28649464

[ref16] Schoenberger M.; Damijonaitis A.; Zhang Z.; Nagel D.; Trauner D. Development of a New Photochromic Ion Channel Blocker via Azologization of Fomocaine. ACS Chem. Neurosci. 2014, 5 (7), 514–518. 10.1021/cn500070w. 24856540 PMC4102962

[ref17] Morstein J.; Awale M.; Reymond J. L.; Trauner D. Mapping the Azolog Space Enables the Optical Control of New Biological Targets. ACS Cent. Sci. 2019, 5 (4), 607–618. 10.1021/acscentsci.8b00881. 31041380 PMC6487453

[ref18] Kobauri P.; Szymanski W.; Cao F.; Thallmair S.; Marrink S. J.; Witte M. D.; Dekker F. J.; Feringa B. L. Biaryl Sulfonamides as Cisoid Azosteres for Photopharmacology. Chem. Commun. 2021, 57 (34), 4126–4129. 10.1039/D1CC00950H. 33908493

[ref19] Borowiak M.; Nahaboo W.; Reynders M.; Nekolla K.; Jalinot P.; Hasserodt J.; Rehberg M.; Delattre M.; Zahler S.; Vollmar A.; Trauner D.; Thorn-Seshold O. Photoswitchable Inhibitors of Microtubule Dynamics Optically Control Mitosis and Cell Death. Cell 2015, 162 (2), 403–411. 10.1016/j.cell.2015.06.049. 26165941

[ref20] Matera C.; Gomila A. M. J.; Camarero N.; Libergoli M.; Soler C.; Gorostiza P. Photoswitchable Antimetabolite for Targeted Photoactivated Chemotherapy. J. Am. Chem. Soc. 2018, 140 (46), 15764–15773. 10.1021/jacs.8b08249. 30346152

[ref21] Mashita T.; Kowada T.; Takahashi H.; Matsui T.; Mizukami S. Light-Wavelength-Based Quantitative Control of Dihydrofolate Reductase Activity by Using a Photochromic Isostere of an Inhibitor. ChemBioChem. 2019, 20 (11), 1382–1386. 10.1002/cbic.201800816. 30656808

[ref22] Kolarski D.; Miller S.; Oshima T.; Nagai Y.; Aoki Y.; Kobauri P.; Srivastava A.; Sugiyama A.; Amaike K.; Sato A.; Tama F.; Szymanski W.; Feringa B. L.; Itami K.; Hirota T. Photopharmacological Manipulation of Mammalian CRY1 for Regulation of the Circadian Clock. J. Am. Chem. Soc. 2021, 143 (4), 2078–2087. 10.1021/jacs.0c12280. 33464888 PMC7863067

[ref23] Hoorens M. W. H.; Ourailidou M. E.; Rodat T.; van der Wouden P. E.; Kobauri P.; Kriegs M.; Peifer C.; Feringa B. L.; Dekker F. J.; Szymanski W. Light-Controlled Inhibition of BRAFV600E Kinase. Eur. J. Med. Chem. 2019, 179, 133–146. 10.1016/j.ejmech.2019.06.042. 31252305

[ref24] Hu T.; Zheng G.; Xue D.; Zhao S.; Li F.; Zhou F.; Zhao F.; Xie L.; Tian C.; Hua T.; Zhao S.; Xu Y.; Zhong G.; Liu Z.-J.; Makriyannis A.; Stevens R. C.; Tao H. Rational Remodeling of Atypical Scaffolds for the Design of Photoswitchable Cannabinoid Receptor Tools. J. Med. Chem. 2021, 64 (18), 13752–13765. 10.1021/acs.jmedchem.1c01088. 34477367

[ref25] Westbrook J. D.; Burley S. K. How Structural Biologists and the Protein Data Bank Contributed to Recent FDA New Drug Approvals. Structure 2019, 27 (2), 211–217. 10.1016/j.str.2018.11.007. 30595456 PMC7325526

[ref26] Sabe V. T.; Ntombela T.; Jhamba L. A.; Maguire G. E. M.; Govender T.; Naicker T.; Kruger H. G. Current Trends in Computer Aided Drug Design and a Highlight of Drugs Discovered via Computational Techniques: A Review. Eur. J. Med. Chem. 2021, 224, 113705 10.1016/j.ejmech.2021.113705. 34303871

[ref27] Wang X.; Song K.; Li L.; Chen L. Structure-Based Drug Design Strategies and Challenges. Curr. Top. Med. Chem. 2018, 18 (12), 998–1006. 10.2174/1568026618666180813152921. 30101712

[ref28] Schehr M.; Ianes C.; Weisner J.; Heintze L.; Müller M. P.; Pichlo C.; Charl J.; Brunstein E.; Ewert J.; Lehr M.; Baumann U.; Rauh D.; Knippschild U.; Peifer C.; Herges R. 2-Azo-, 2-Diazocine-Thiazols and 2-Azo-Imidazoles as Photoswitchable Kinase Inhibitors: Limitations and Pitfalls of the Photoswitchable Inhibitor Approach. Photochem. Photobiol. Sci. 2019, 18 (6), 1398–1407. 10.1039/C9PP00010K. 30924488

[ref29] Hoorens M. W. H.; Ourailidou M. E.; Rodat T.; van der Wouden P. E.; Kobauri P.; Kriegs M.; Peifer C.; Feringa B. L.; Dekker F. J.; Szymanski W. Light-Controlled Inhibition of BRAFV600E Kinase. Eur. J. Med. Chem. 2019, 179, 133 10.1016/j.ejmech.2019.06.042. 31252305

[ref30] Xu Z.; Shi L.; Jiang D.; Cheng J.; Shao X.; Li Z. Azobenzene Modified Imidacloprid Derivatives as Photoswitchable Insecticides: Steering Molecular Activity in a Controllable Manner. Sci. Rep. 2015, 5 (13962), 1–8. 10.1038/srep13962. PMC4593031 26434681

[ref31] Duran-Corbera A.; Catena J.; Otero-Viñas M.; Llebaria A.; Rovira X. Photoswitchable Antagonists for a Precise Spatiotemporal Control of B2-Adrenoceptors. J. Med. Chem. 2020, 63 (15), 8458–8470. 10.1021/acs.jmedchem.0c00831. 32686936

[ref32] Cheng B.; Morstein J.; Ladefoged L. K.; Maesen J. B.; Schiøtt B.; Sinning S.; Trauner D. A Photoswitchable Inhibitor of the Human Serotonin Transporter. ACS Chem. Neurosci. 2020, 11 (9), 1231–1237. 10.1021/acschemneuro.9b00521. 32275382 PMC8022892

[ref33] Hinnah K.; Willems S.; Morstein J.; Heering J.; Hartrampf F. W. W.; Broichhagen J.; Leippe P.; Merk D.; Trauner D. Photohormones Enable Optical Control of the Peroxisome Proliferator-Activated Receptor γ(PPARγ). J. Med. Chem. 2020, 63 (19), 10908–10920. 10.1021/acs.jmedchem.0c00654. 32886507 PMC11684002

[ref34] Willems S.; Morstein J.; Hinnah K.; Trauner D.; Merk D. A Photohormone for Light-Dependent Control of PPARα in Live Cells. J. Med. Chem. 2021, 64 (14), 10393–10402. 10.1021/acs.jmedchem.1c00810. 34213899

[ref35] Plowright A. T.; Johnstone C.; Kihlberg J.; Pettersson J.; Robb G.; Thompson R. A. Hypothesis Driven Drug Design: Improving Quality and Effectiveness of the Design-Make-Test-Analyse Cycle. Drug Discovery Today 2012, 17 (1–2), 56–62. 10.1016/j.drudis.2011.09.012. 21963616

[ref36] Schnell J. R.; Dyson H. J.; Wright P. E. Structure, Dynamics, and Catalytic Function of Dihydrofolate Reductase. Annu. Rev. Biophys. Biomol. Struct. 2004, 33, 119–140. 10.1146/annurev.biophys.33.110502.133613. 15139807

[ref37] Koźmiński P.; Halik P. K.; Chesori R.; Gniazdowska E. Overview of Dual-Acting Drug Methotrexate in Different Neurological Diseases, Autoimmune Pathologies and Cancers. Int. J. Mol. Sci. 2020, 21 (10), 3483 10.3390/ijms21103483. PMC7279024 32423175

[ref38] Crellin E.; Mansfield K. E.; Leyrat C.; Nitsch D.; Douglas I. J.; Root A.; Williamson E.; Smeeth L.; Tomlinson L. A. Trimethoprim Use for Urinary Tract Infection and Risk of Adverse Outcomes in Older Patients: Cohort Study. BMJ 2018, 360, k341 10.1136/bmj.k341. 29438980 PMC5806507

[ref39] Butler M. S.; Paterson D. L. Antibiotics in the Clinical Pipeline in October 2019. J. Antibiot. (Tokyo). 2020, 73 (6), 329–364. 10.1038/s41429-020-0291-8. 32152527 PMC7223789

[ref40] Schneider P.; Hawser S.; Islam K. Iclaprim, a Novel Diaminopyrimidine with Potent Activity on Trimethoprim Sensitive and Resistant Bacteria. Bioorg. Med. Chem. Lett. 2003, 13 (23), 4217–4221. 10.1016/j.bmcl.2003.07.023. 14623005

[ref41] Ballister E. R.; Aonbangkhen C.; Mayo A. M.; Lampson M. A.; Chenoweth D. M. Localized Light-Induced Protein Dimerization in Living Cells Using a Photocaged Dimerizer. Nat. Commun. 2014, 5, 5475 10.1038/ncomms6475. 25400104 PMC4308733

[ref42] Lauxen A. I.; Kobauri P.; Wegener M.; Hansen M. J.; Galenkamp N. S.; Maglia G.; Szymanski W.; Feringa B. L.; Kuipers O. P. Mechanism of Resistance Development in E. Coli against TCAT, a Trimethoprim-Based Photoswitchable Antibiotic. Pharmaceuticals 2021, 14 (5), 392 10.3390/ph14050392. 33919397 PMC8143356

[ref43] Sherman W.; Day T.; Jacobson M. P.; Friesner R. A.; Farid R. Novel Procedure for Modeling Ligand/Receptor Induced Fit Effects. J. Med. Chem. 2006, 49 (2), 534–553. 10.1021/jm050540c. 16420040

[ref44] Bender B. J.; Gahbauer S.; Luttens A.; Lyu J.; Webb C. M.; Stein R. M.; Fink E. A.; Balius T. E.; Carlsson J.; Irwin J. J.; Shoichet B. K. A Practical Guide to Large-Scale Docking. Nat. Protoc. 2021, 16 (10), 4799–4832. 10.1038/s41596-021-00597-z. 34561691 PMC8522653

[ref45] Wang Z.; Sun H.; Yao X.; Li D.; Xu L.; Li Y.; Tian S.; Hou T. Comprehensive Evaluation of Ten Docking Programs on a Diverse Set of Protein-Ligand Complexes: The Prediction Accuracy of Sampling Power and Scoring Power. Phys. Chem. Chem. Phys. 2016, 18 (18), 12964–12975. 10.1039/C6CP01555G. 27108770

[ref46] Fischer A.; Smieško M.; Sellner M.; Lill M. A. Decision Making in Structure-Based Drug Discovery: Visual Inspection of Docking Results. J. Med. Chem. 2021, 64 (5), 2489–2500. 10.1021/acs.jmedchem.0c02227. 33617246

[ref47] De Vivo M.; Masetti M.; Bottegoni G.; Cavalli A. Role of Molecular Dynamics and Related Methods in Drug Discovery. J. Med. Chem. 2016, 59 (9), 4035–4061. 10.1021/acs.jmedchem.5b01684. 26807648

[ref48] Zhang D.; Lazim R. Application of Conventional Molecular Dynamics Simulation in Evaluating the Stability of Apomyoglobin in Urea Solution. Sci. Rep. 2017, 7 (44651), 44651 10.1038/srep44651. 28300210 PMC5353640

[ref49] Manna M. S.; Tamer Y. T.; Gaszek I.; Poulides N.; Ahmed A.; Wang X.; Toprak F. C. R.; Woodard D. N. R.; Koh A. Y.; Williams N. S.; Borek D.; Atilgan A. R.; Hulleman J. D.; Atilgan C.; Tambar U.; Toprak E. A Trimethoprim Derivative Impedes Antibiotic Resistance Evolution. Nat. Commun. 2021, 12, 2949 10.1038/s41467-021-23191-z. 34011959 PMC8134463

[ref50] Pelphrey P. M.; Popov V. M.; Joska T. M.; Beierlein J. M.; Bolstad E. S. D.; Fillingham Y. A.; Wright D. L.; Anderson A. C. Highly Efficient Ligands for Dihydrofolate Reductase from Cryptosporidium Hominis and Toxoplasma Gondii Inspired by Structural Analysis. J. Med. Chem. 2007, 50 (5), 940–950. 10.1021/jm061027h. 17269758

[ref51] Otzen T.; Wempe E. G.; Kunz B.; Bartels R.; Lehwark-Yvetot G.; Hänsel W.; Schaper K. J.; Seydel J. K. Folate-Synthesizing Enzyme System as Target for Development of Inhibitors and Inhibitor Combinations against Candida Albicans - Synthesis and Biological Activity of New 2,4-Diaminopyrimidines and 4′-Substituted 4-Aminodiphenyl Sulfones. J. Med. Chem. 2004, 47 (1), 240–253. 10.1021/jm030931w. 14695838

[ref52] Bartova K.; Čechova L.; Prochazkova E.; Socha O.; Janeba Z.; Dracnsky M. Influence of Intramolecular Charge Transfer and Nuclear Quantum Effects on Intramolecular Hydrogen Bonds in Azopyrimidines. J. Org. Chem. 2017, 82 (19), 10350–10359. 10.1021/acs.joc.7b01810. 28829606

[ref53] Østergaard H.; Tachibana C.; Winther J. R. Monitoring Disulfide Bond Formation in the Eukaryotic Cytosol. J. Cell Biol. 2004, 166 (3), 337–345. 10.1083/jcb.200402120. 15277542 PMC2172265

[ref54] Perola E.; Charifson P. S. Conformational Analysis of Drug-Like Molecules Bound to Proteins: An Extensive Study of Ligand Reorganization upon Binding. J. Med. Chem. 2004, 47 (10), 2499–2510. 10.1021/jm030563w. 15115393

[ref55] Lombardo M. N.; G-Dayanandan N.; Wright D. L.; Anderson A. C. Crystal Structures of Trimethoprim-Resistant DfrA1 Rationalize Potent Inhibition by Propargyl-Linked Antifolates. ACS Infect. Dis. 2016, 2 (2), 149–156. 10.1021/acsinfecdis.5b00129. 27624966 PMC5108240

[ref56] Gómez-Santacana X.; de Munnik S. M.; Vijayachandran P.; Da Costa Pereira D.; Bebelman J. P. M.; de Esch I. J. P.; Vischer H. F.; Wijtmans M.; Leurs R. Photoswitching the Efficacy of a Small-Molecule Ligand for a Peptidergic GPCR: From Antagonism to Agonism. Angew. Chem., Int. Ed. 2018, 57 (36), 11608–11612. 10.1002/anie.201804875. 29926530

[ref57] Szymański W.; Wu B.; Poloni C.; Janssen D. B.; Feringa B. L. Azobenzene Photoswitches for Staudinger-Bertozzi Ligation. Angew. Chem., Int. Ed. 2013, 52 (7), 2068–2072. 10.1002/anie.201208596. 23307784

[ref58] Szymanski W.; Ourailidou M. E.; Velema W. A.; Dekker F. J.; Feringa B. L. Light-Controlled Histone Deacetylase (HDAC) Inhibitors: Towards Photopharmacological Chemotherapy. Chem. - Eur. J. 2015, 21 (46), 16517–16524. 10.1002/chem.201502809. 26418117 PMC5221732

[ref59] Calbo J.; Weston C. E.; White A. J. P.; Rzepa H. S.; Contreras-García J.; Fuchter M. J. Tuning Azoheteroarene Photoswitch Performance through Heteroaryl Design. J. Am. Chem. Soc. 2017, 139 (3), 1261–1274. 10.1021/jacs.6b11626. 28009517

[ref60] Crespi S.; Simeth N. A.; König B. Heteroaryl Azo Dyes as Molecular Photoswitches. Nat. Rev. Chem. 2019, 3 (3), 133–146. 10.1038/s41570-019-0074-6.

[ref61] Mafy N. N.; Matsuo K.; Hiruma S.; Uehara R.; Tamaoki N. Photoswitchable CENP-E Inhibitor Enabling the Dynamic Control of Chromosome Movement and Mitotic Progression. J. Am. Chem. Soc. 2020, 142 (4), 1763–1767. 10.1021/jacs.9b12782. 31927956

[ref62] Volarić J.; Szymanski W.; Simeth N. A.; Feringa B. L. Molecular Photoswitches in Aqueous Environments. Chem. Soc. Rev. 2021, 50, 12377 10.1039/D0CS00547A. 34590636 PMC8591629

[ref63] Grabowski S. J. Hydrogen Bonding Strength—Measures Based on Geometric and Topological Parameters. J. Phys. Org. Chem. 2004, 17 (1), 18–31. 10.1002/poc.685.

[ref64] Laurence C.; Brameld K. A.; Graton J.; Le Questel J.-Y.; Renault E. The PKBHX Database: Toward a Better Understanding of Hydrogen-Bond Basicity for Medicinal Chemists. J. Med. Chem. 2009, 52 (14), 4073–4086. 10.1021/jm801331y. 19537797

[ref65] Guterres H.; Im W. Improving Protein-Ligand Docking Results with High-Throughput Molecular Dynamics Simulations. J. Chem. Inf. Model. 2020, 60 (4), 2189–2198. 10.1021/acs.jcim.0c00057. 32227880 PMC7534544

[ref66] Scocchera E.; Reeve S. M.; Keshipeddy S.; Lombardo M. N.; Hajian B.; Sochia A. E.; Alverson J. B.; Priestley N. D.; Anderson A. C.; Wright D. L. Charged Nonclassical Antifolates with Activity Against Gram-Positive and Gram-Negative Pathogens. ACS Med. Chem. Lett. 2016, 7 (7), 692–696. 10.1021/acsmedchemlett.6b00120. 27437079 PMC4948012

[ref67] Donthamsetti P.; Konrad D. B.; Hetzler B.; Fu Z.; Trauner D.; Isacoff E. Y. Selective Photoswitchable Allosteric Agonist of a G Protein-Coupled Receptor. J. Am. Chem. Soc. 2021, 143 (24), 8951–8956. 10.1021/jacs.1c02586. 34115935 PMC8227462

[ref68] Vomasta D.; Högner C.; Branda N. R.; König B. Regulation of Human Carbonic Anhydrase I (HCAI) Activity by Using a Photochromic Inhibitor. Angew. Chem., Int. Ed. 2008, 47 (40), 7644–7647. 10.1002/anie.200802242. 18767093

[ref69] Bissantz C.; Kuhn B.; Stahl M. A Medicinal Chemist’s Guide to Molecular Interactions. J. Med. Chem. 2010, 53 (14), 5061–5084. 10.1021/jm100112j. 20345171 PMC2905122

[ref70] Arkhipova V.; Fu H.; Hoorens M. W. H.; Trinco G.; Lameijer L. N.; Marin E.; Feringa B. L.; Poelarends G. J.; Szymanski W.; Slotboom D. J.; Guskov A. Structural Aspects of Photopharmacology: Insight into the Binding of Photoswitchable and Photocaged Inhibitors to the Glutamate Transporter Homologue. J. Am. Chem. Soc. 2021, 143 (3), 1513–1520. 10.1021/jacs.0c11336. 33449695 PMC7844824

[ref71] Pospich S.; Küllmer F.; Nasufović V.; Funk J.; Belyy A.; Bieling P.; Arndt H. D.; Raunser S. Cryo-EM Resolves Molecular Recognition Of An Optojasp Photoswitch Bound To Actin Filaments In Both Switch States. Angew. Chem., Int. Ed. 2021, 60 (16), 8678–8682. 10.1002/anie.202013193. PMC8048601 33449370

[ref72] Reynders M.; Chaikuad A.; Berger B.; Bauer K.; Koch P.; Laufer S.; Knapp S.; Trauner D. Controlling the Covalent Reactivity of a Kinase Inhibitor with Light. Angew. Chem., Int. Ed. 2021, 60, 20178–20183. 10.1002/anie.202103767. PMC9940781 34081840

[ref73] Erb W.; Hellal A.; Albini M.; Rouden J.; Blanchet J. An Easy Route to (Hetero)Arylboronic Acids. Chem. - Eur. J. 2014, 20 (22), 6608–6612. 10.1002/chem.201402487. 24737711

[ref74] Lavastre O.; Ollivier L.; Dixneuf P. H.; Sibandhit S. Sequential Catalytic Synthesis of Rod-like Conjugated Poly-Ynes. Tetrahedron 1996, 52 (15), 5495–5504. 10.1016/0040-4020(96)00240-2.

[ref75] Lavastre O.; Cabioch S.; Dixneuf P. H.; Vohlidal J. Selective and Efficient Access to Ortho, Meta and Para Ring-Substituted Phenylacetylene Derivatives R-[C≡C-C6H4](x)-Y (Y:H, NO2, CN, I, NH2). Tetrahedron 1997, 53 (22), 7595–7604. 10.1016/S0040-4020(97)00451-1.

[ref76] Tian Y. J.; Meijer E. W.; Wang F. Cooperative Self-Assembly of Platinum(II) Acetylide Complexes. Chem. Commun. 2013, 49 (80), 9197–9199. 10.1039/c3cc44997a. 23995042

[ref77] Vasilevsky S. F.; Klyatskaya S. V.; Elguero J. One-Pot Synthesis of Monosubstituted Aryl(Hetaryl)Acetylenes by Direct Introduction of the C≡CH Residue into Arenes and Hetarenes. Tetrahedron 2004, 60 (31), 6685–6688. 10.1016/j.tet.2004.05.093.

[ref78] Ferrazzano L.; Martelli G.; Fantoni T.; Daka A.; Corbisiero D.; Viola A.; Ricci A.; Cabri W.; Tolomelli A. Fast Heck-Cassar-Sonogashira (Hcs) Reactions in Green Solvents. Org. Lett. 2020, 22 (10), 3969–3973. 10.1021/acs.orglett.0c01269. 32342693 PMC8007125

[ref79] Chavannavar A. P.; Oliver A. G.; Ashfeld B. L. An Umpolung Approach toward N-Aryl Nitrone Construction: A Phosphine-Mediated Addition of 1,2-Dicarbonyls to Nitroso Electrophiles. Chem. Commun. 2014, 50 (74), 10853–10856. 10.1039/C4CC05044D. 25090627

[ref80] Sakai N.; Asama S.; Anai S.; Konakahara T. One-Pot Preparation of Azobenzenes from Nitrobenzenes by the Combination of an Indium-Catalyzed Reductive Coupling and a Subsequent Oxidation. Tetrahedron 2014, 70 (11), 2027–2033. 10.1016/j.tet.2014.01.048.

[ref81] Badjić J. D.; Kostić N. M. Behavior of Organic Compounds Confined in Monoliths of Sol-Gel Silica Glass. Effects of Guest-Host Hydrogen Bonding on Uptake, Release, and Isomerization of the Guest Compounds. J. Mater. Chem. 2001, 11 (2), 408–418. 10.1039/b005823h.

[ref82] McIntyre J.; Simpson J. C. E. Cinnolines and Other Heterocyclic Types in Relation to the Chemotherapy of Trypanosomiasis. Part IV. Synthesis of Azocinnoline Derivatives. J. Chem. Soc. 1952, 2606–2615. 10.1039/jr9520002606.

[ref83] Stricker L.; Fritz E. C.; Peterlechner M.; Doltsinis N. L.; Ravoo B. J. Arylazopyrazoles as Light-Responsive Molecular Switches in Cyclodextrin-Based Supramolecular Systems. J. Am. Chem. Soc. 2016, 138 (13), 4547–4554. 10.1021/jacs.6b00484. 26972671

[ref84] Lameijer L. N.; Budzak S.; Simeth N. A.; Hansen M. J.; Feringa B. L.; Jacquemin D.; Szymanski W. General Principles for the Design of Visible-Light-Responsive Photoswitches: Tetra-Ortho-Chloro-Azobenzenes. Angew. Chem., Int. Ed. 2020, 59 (48), 21663–21670. 10.1002/anie.202008700. PMC7756550 33462976

[ref85] Roos K.; Wu C.; Damm W.; Reboul M.; Stevenson J. M.; Lu C.; Dahlgren M. K.; Mondal S.; Chen W.; Wang L.; Abel R.; Friesner R. A.; Harder E. D. OPLS3e : Extending Force Field Coverage for Drug-Like Small Molecules. J. Chem. Theory Comput. 2019, 15, 1863–1874. 10.1021/acs.jctc.8b01026. 30768902

[ref86] Friesner R. A.; Murphy R. B.; Repasky M. P.; Frye L. L.; Greenwood J. R.; Halgren T. A.; Sanschagrin P. C.; Mainz D. T. Extra Precision Glide: Docking and Scoring Incorporating a Model of Hydrophobic Enclosure for Protein–Ligand Complexes. J. Med. Chem. 2006, 49 (21), 6177–6196. 10.1021/jm051256o. 17034125

[ref87] Jorgensen W. L.; Chandrasekhar J.; Madura J. D.; Impey R. W.; Klein M. L. Comparison of Simple Potential Functions for Simulating Liquid Water. J. Chem. Phys. 1983, 79 (2), 926–935. 10.1063/1.445869.

[ref88] Shaw D. E. Proceedings of the 2006 ACM/IEEE Conference on Supercomputing (SC ’06), November 11–17, 2006, Tampa, FL; Association for Computing Machinery: New York, 2006.

[ref89] Essmann U.; Perera L.; Berkowitz M. L.; Darden T.; Lee H.; Pedersen L. G. A Smooth Particle Mesh Ewald Method. J. Chem. Phys. 1995, 103 (19), 8577–8593. 10.1063/1.470117.

[ref90] Berendsen H. J. C.; Postma J. P. M.; Van Gunsteren W. F.; Dinola A.; Haak J. R. Molecular Dynamics with Coupling to an External Bath. J. Chem. Phys. 1984, 81 (8), 3684–3690. 10.1063/1.448118.

[ref91] Martyna G. J.; Tobias D. J.; Klein M. L. Constant Pressure Molecular Dynamics Algorithms. J. Chem. Phys. 1994, 101 (5), 4177–4189. 10.1063/1.467468.

[ref92] Evans D. J.; Holian B. L. The Nose–Hoover Thermostat. J. Chem. Phys. 1985, 83 (8), 4069–4074. 10.1063/1.449071.

[ref93] Knapp B.; Ospina L.; Deane C. M. Avoiding False Positive Conclusions in Molecular Simulation: The Importance of Replicas. J. Chem. Theory Comput. 2018, 14 (12), 6127–6138. 10.1021/acs.jctc.8b00391. 30354113

[ref94] Kumar S.; Nussinov R. Relationship between Ion Pair Geometries and Electrostatic. Biophys. J. 2002, 83 (3), 1595–1612. 10.1016/S0006-3495(02)73929-5. 12202384 PMC1302257

