# Supplementary material for: Hypothesis-Driven, Structure-Based Design in Photopharmacology: The Case of eDHFR Inhibitors
Source: J Med Chem. 2022 Mar 8;65(6):4798–817. doi: 10.1021/acs.jmedchem.1c01962 (PMC8958501; doi:10.1021/acs.jmedchem.1c01962)
Supplement: Supplementary file 1 — jm1c01962_si_001.pdf [file jm1c01962_si_001.pdf]

Supporting Information

Hypothesis-Driven, Structure-Based Design in Photopharmacology: the Case of eDHFR Inhibitors

Piermichele Kobauri,[a] Nicole S. Galenkamp,[b] Albert M. Schulte,[a] Jisk de Vries,[a] Nadja A. Simeth,[a,c] Giovanni Maglia,[b] Sebastian Thallmair,[d,e] Dušan Kolarski,[a,f] Wiktor Szymanski,*[a,f] Ben L. Feringa*[a]

[a] Stratingh Institute for Chemistry, University of Groningen, Nijenborgh 4, 9747 AG Groningen, The Netherlands

[b] Groningen Biomolecular Science & Biotechnology Institute, University of Groningen, Nijenborgh 4, 9747 AG Groningen, The Netherlands

[c] Current address: Institute for Organic and Biomolecular Chemistry, University of Goettingen, Tammannstr. 2, 37077 Göttingen, Germany

[d] Groningen Biomolecular Sciences and Biotechnology Institute & Zernike Institute for Advanced Materials, University of Groningen, Nijenborgh 7, 9747 AG Groningen, The Netherlands

[e] Current address: Frankfurt Institute for Advanced Studies, Ruth-Moufang-Straße 1, 60438 Frankfurt am Main, Germany

[f] Current address: DWI-Leibniz Institut für interaktive Materialien e.V., RWTH Aachen University, Forckenbeckstraße 50, 52074 Aachen, Germany

[g] Department of Radiology, Medical Imaging Center, University of Groningen, University Medical Center Groningen, Hanzeplein 1, 9713 GZ Groningen, The Netherlands

* Corresponding authors: w.szymanski@umcg.nl, b.l.feringa@rug.nl

Table of contents

S1. Synthesis and characterization 3

S1.1. Synthetic schemes 3

S1.1.1. First hypothesis 3

S1.1.2. Second hypothesis 4

S1.1.3. Third hypothesis 4

S1.1.4. Fourth hypothesis 5

S1.1.5. Fifth hypothesis 5

S1.2. Synthetic procedures 7

S2. Photochemical and thermal isomerization studied by UV-vis and NMR spectroscopies 9

S2.1. First hypothesis 9

S2.2. Second hypothesis 16

S2.3. Third hypothesis 18

S2.4. Fourth hypothesis 21

S2.5. Fifth hypothesis 24

S2.6. Overviews of thermal half-lives and PSDs 30

S3. *In vitro* pharmacological characterization 32

S3.1. Protein and DNA sequences 32

S3.2. eDHFR inhibition assay 33
S4. Antibacterial assay and bacterial growth curves 41
S5. Molecular modeling 44
S5.1. Geometry measurements from the CSD 44
S5.2. Molecular docking and MD simulations 44
S5.2.1. Reference compounds 46
S5.2.2. First hypothesis 51
S5.2.3. Second hypothesis 54
S5.2.4. Third hypothesis 58
S5.2.5. Fourth hypothesis 60
S5.2.6. Fifth hypothesis 65
S5.3. Physicochemical descriptors 74
S6. UHPLC-MS traces of compounds tested for biological activity 77
S7. NMR and HRMS data 92
S7.1. First hypothesis 93
S7.2. Second hypothesis 115
S7.3. Third hypothesis 129
S7.4. Fourth hypothesis 145
S7.5. Fifth hypothesis 165
S8. References 203

S1. Synthesis and characterization

S1.1. Synthetic schemes

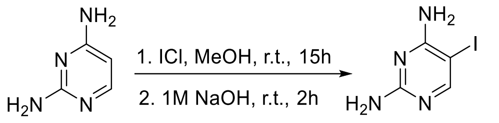

S1 56%

S1.1.1. <u>First hypothesis</u>

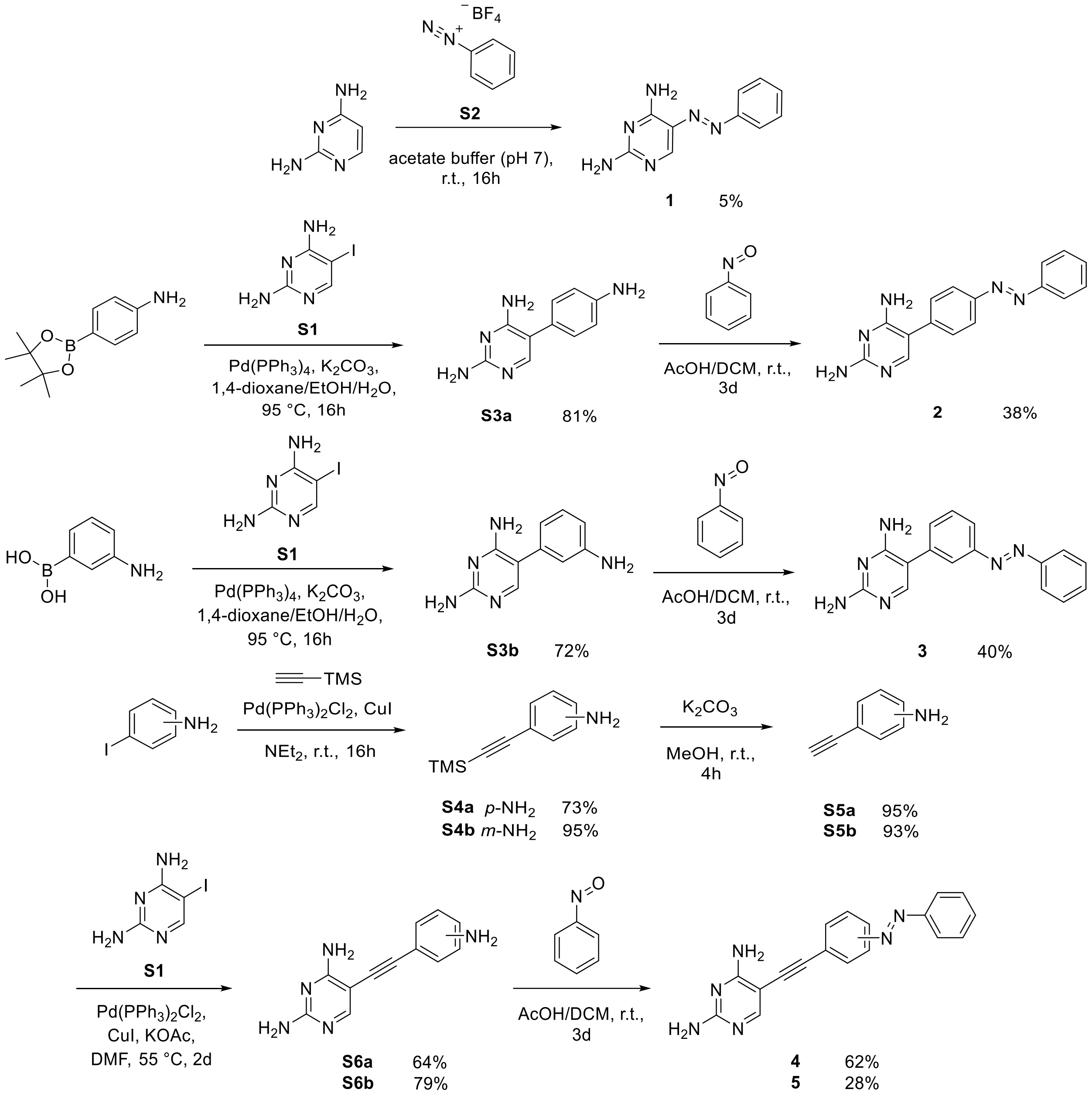

Scheme S1. Synthesis of compounds **1-5**.

S1.1.2. Second hypothesis

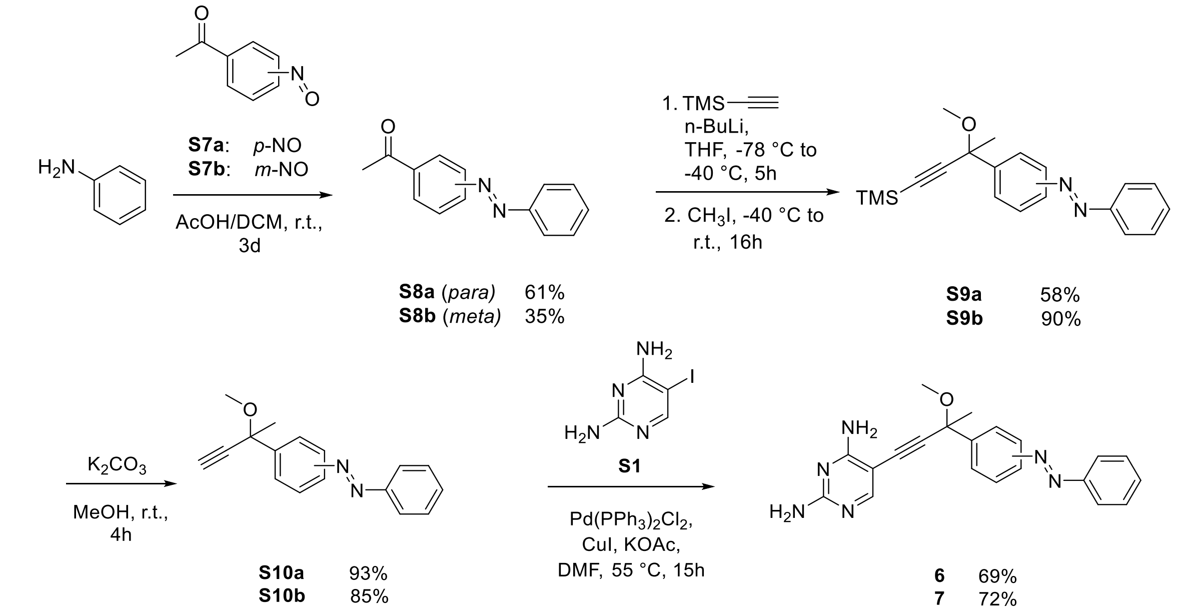

Scheme S2. Synthesis of compounds **6** and **7**.

S1.1.3. Third hypothesis

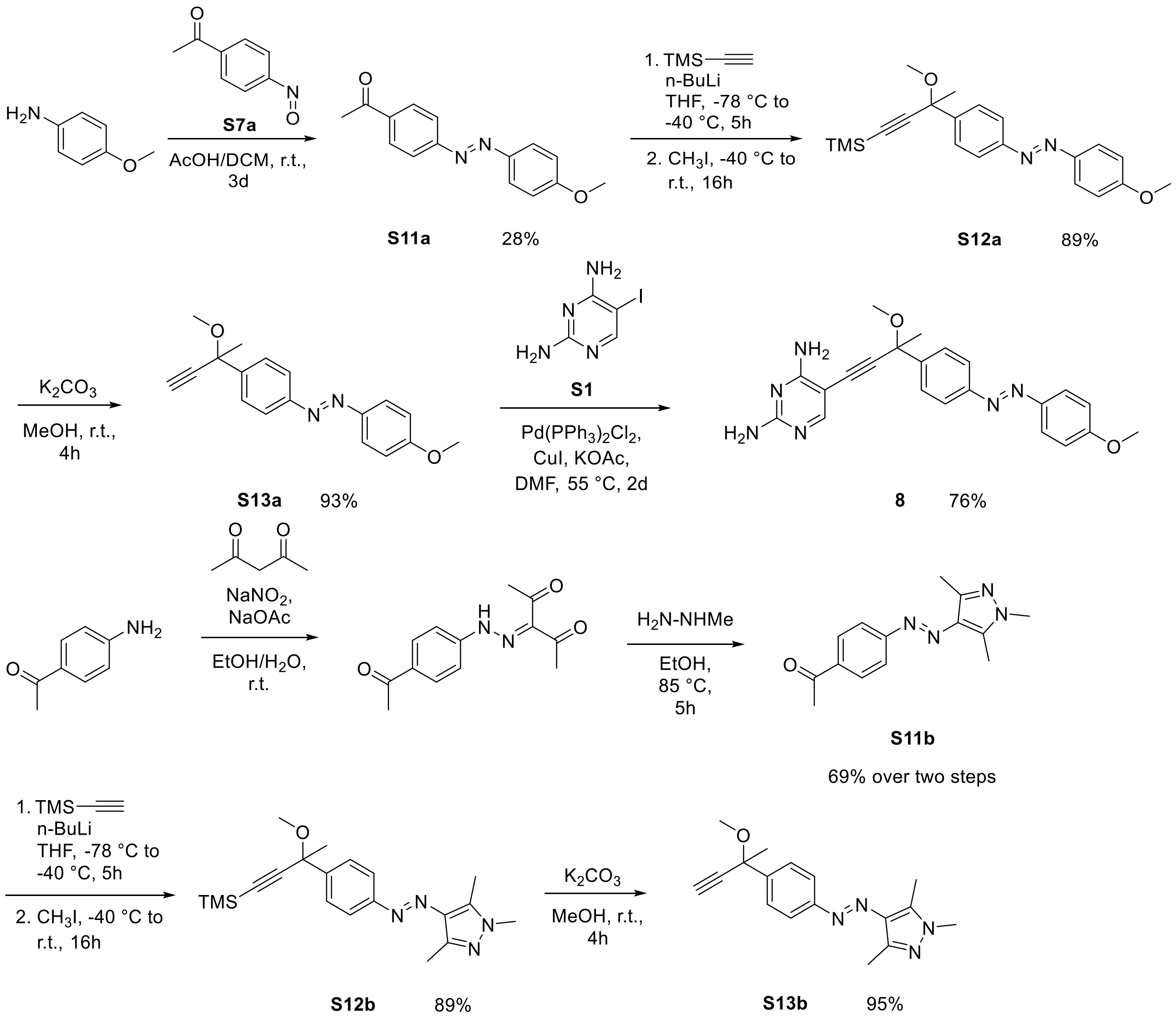

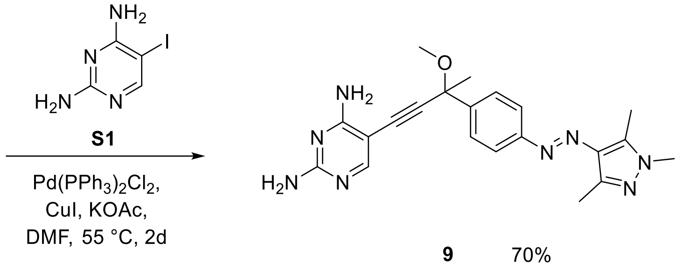

Scheme S3. Synthesis of compounds **8** and **9**.

S1.1.4. Fourth hypothesis

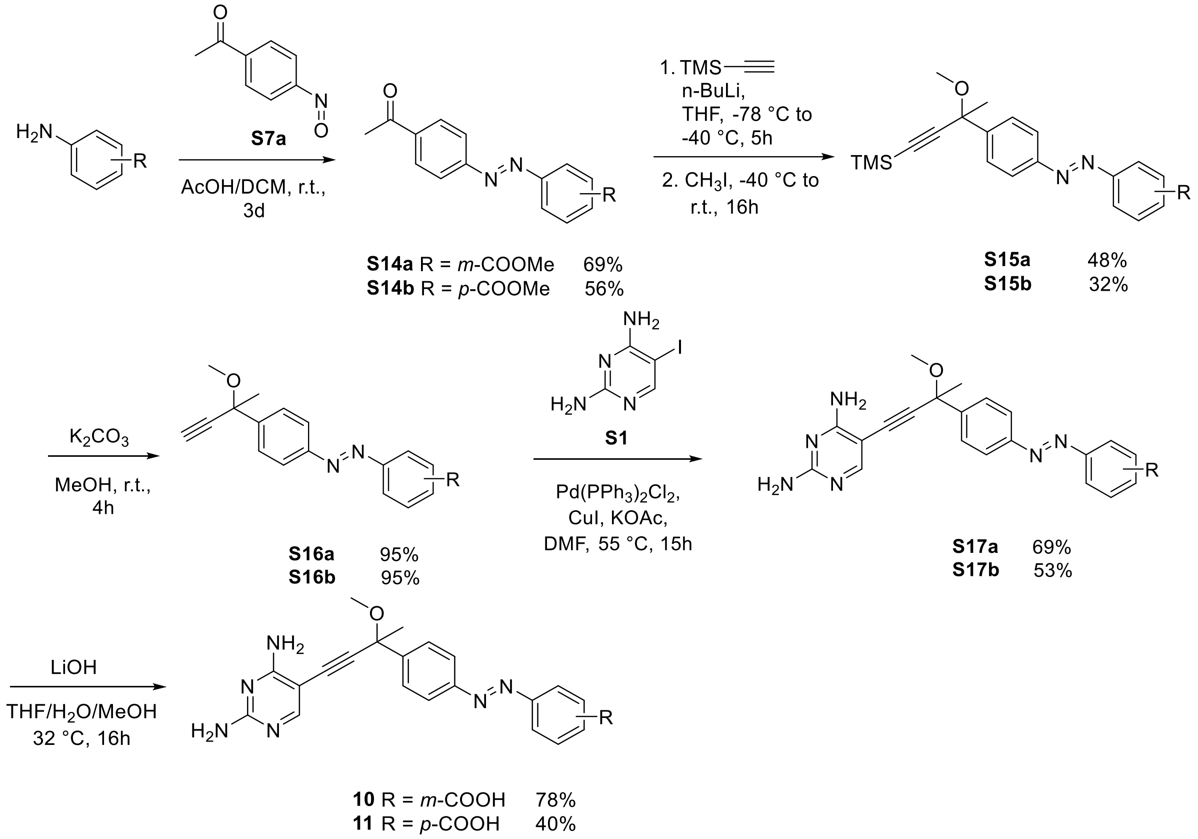

Scheme S4. Synthesis of compounds **10** and **11**.

S1.1.5. Fifth hypothesis

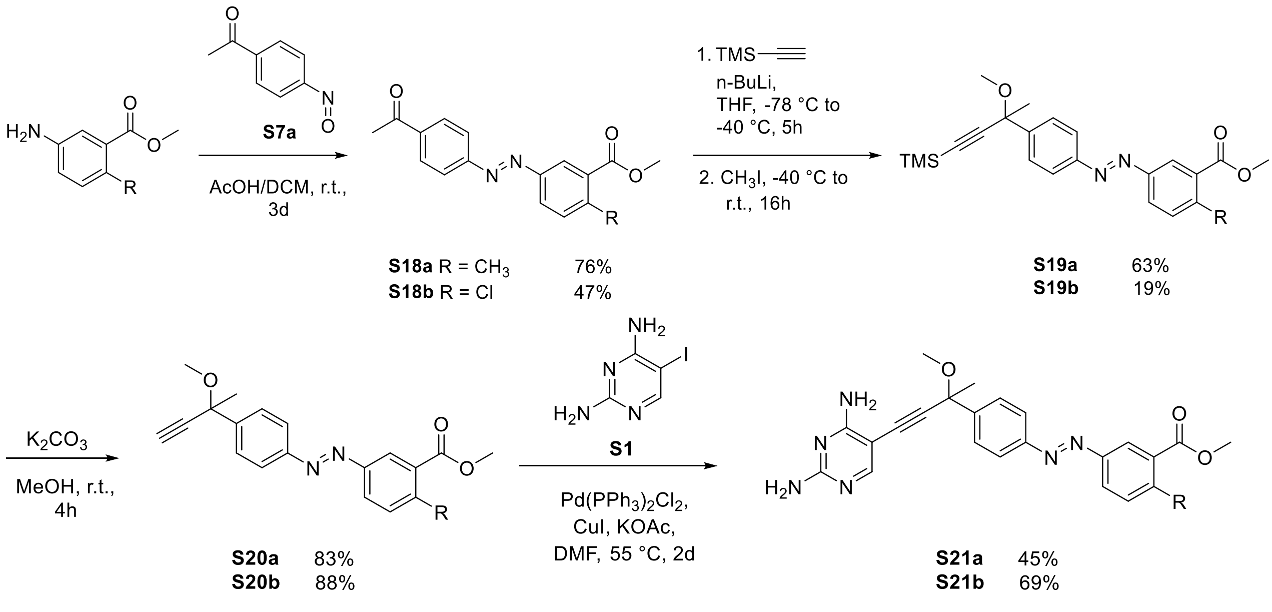

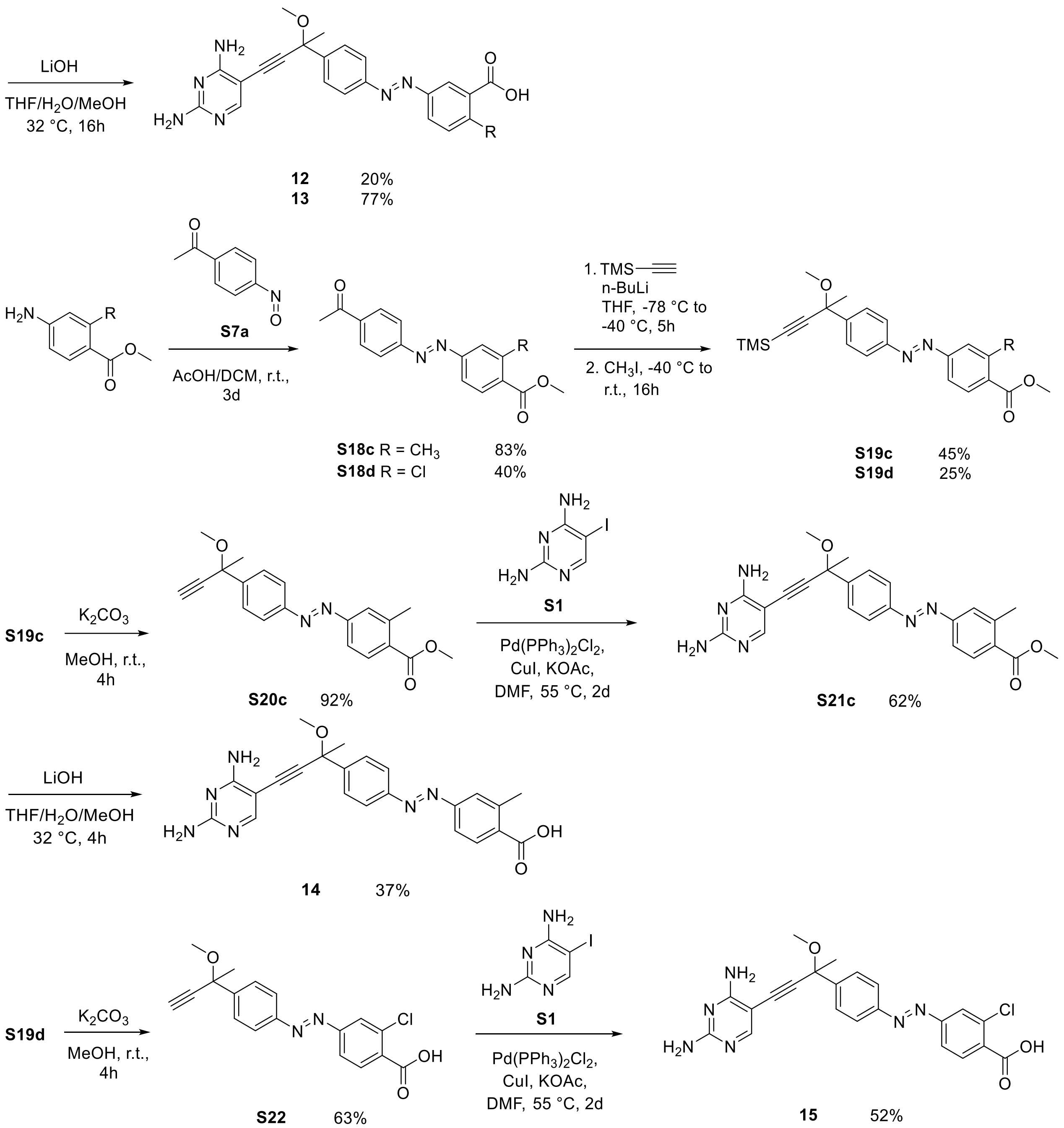

Scheme S5. Synthesis of compounds **12-15**.

S1.2. Synthetic procedures

5-Iodopyrimidine-2,4-diamine (S1)

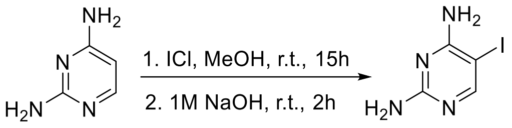

S1 56%

Prepared following a literature procedure.[1] In a flame-dried, round-bottom flask, 2,4-diaminopyrimidine (1.0 g, 9.1 mmol) was dissolved in MeOH (30 mL). ICl (1.5 mL, 29 mmol) was added dropwise, and the mixture was stirred at room temperature for 15 h. MeOH was removed under reduced pressure, and the obtained viscous oil was stirred in Et_2O (40 mL) for 45 min. The resulting precipitate was filtered off and thoroughly washed with Et_2O. Subsequently, the solid was suspended in 1.0 M aq. NaOH (100 mL) and the mixture stirred at r.t. for 2 h. The solid was filtered, washed with water (2 x 10 mL), and dried to afford **S1** as a brown solid (1.2 g, 5.1 mmol, 56 %). An analytical sample was prepared by recrystallization from MeCN to give **S1** as colorless crystals. R_f: 0.60 (DCM/MeOH = 9:1, v/v). ^{1}H NMR (400 MHz, DMSO-d_6) δ = 7.90 (s, 1H), 6.35 (br s, 2H), 6.06 (br s, 2H); spectrum in agreement with literature data.[1] HRMS (ESI$^+$) *m/z* calc. for $[M+H]^+$ ($C_4H_6IN_4^+$): 236.9632, found: 236.9628. M.p. (dec.): 197 °C (lit. 212–214 °C).[1]

Benzenediazonium tetrafluoroborate (S2)

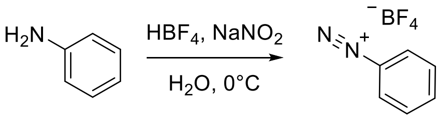

S2

Prepared following a literature procedure.[2] To a solution of aniline (1.8 g, 20 mmol) in ethanol at r.t. was added HBF_4 (48 % in water, 12 mL), and the reaction mixture was stirred for 2 min. The mixture was cooled to 0 °C and aqueous solution *tert*-butyl nitrite was added dropwise. The mixture was stirred at 0 °C for 15 min and then 1 h at r.t. Diethyl ether was added to the reaction mixture and the resulting solids were filtered, washed with diethyl ether (3 x 20 mL) and dried under vacuum to give S6 (0.78 g, 4.1 mmol, 21 %). The grey solid (crude yield = 21 %) was used directly for the next step without purification.

4-[(Trimethylsilyl)ethynyl]aniline (S4a)

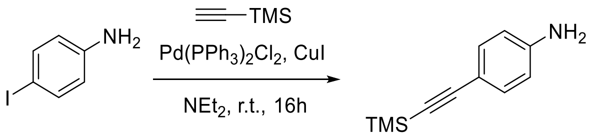

S4a 73%

Prepared following a literature procedure.[3] In a flame-dried, 3-necked round-bottom flask, under N_2 atmosphere, TMS-acetylene (0.54 g, 5.5 mmol) was added to a solution of 4-iodoaniline (1.0 g, 4.6 mmol) in deoxygenated diethylamine (20 mL) with $PdCl_2(PPh_3)_2$ (64 mg, 90 µmol) and CuI (6.0 mg, 32 µmol). After stirring for 16 h at room temperature, the solvent was evaporated and the residue was extracted with Et_2O (40 mL), washed with brine (2 x 20 mL), dried over $MgSO_4$ and the solvent was

removed under reduced pressure. The residue was purified by flash chromatography (Pentane/EtOAc = 7:3, v/v) to afford **S4a** as a brown solid (0.64 g, 3.4 mmol, 73 %). R_f: 0.68 (Pentane/EtOAc = 7:3, v/v). ^{1}H NMR (400 MHz, $CDCl_3$) δ 7.27 (AA'BB', 2H), 6.55 (AA'BB', 2H), 3.79 (br s, 2H), 0.24 (s, 9H); spectrum in agreement with literature data.[4] HRMS (ESI$^+$) *m/z* calc. for $[M+H]^+$ ($C_{11}H_{16}NSi^+$): 190.1047, found: 190.1044. M.p.: 87–89 °C (lit. 95–96 °C).[4]

3-[(Trimethylsilyl)ethynyl]aniline (S4b)

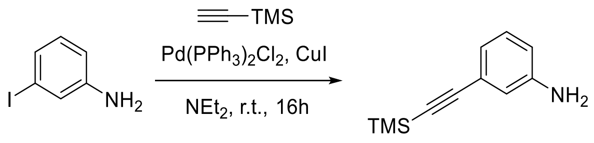

S4b 95%

Prepared following a literature procedure.[5] In a flame-dried, 3-necked round-bottom flask, under N_2 atmosphere, TMS-acetylene (1.6 g, 16 mmol) was added to a solution of 3-iodoaniline (3.0 g, 14 mmol) in deoxygenated diethylamine (60 mL) with $PdCl_2(PPh_3)_2$ (0.19 g, 0.27 mmol) and CuI (19 mg, 0.10 mmol). After stirring for 16 h at room temperature, the solvent was evaporated and the residue was extracted with Et_2O (80 mL), washed with brine (2 x 40 mL), dried over $MgSO_4$ and the solvent was removed under reduced pressure. The crude product was purified by flash chromatography (Pentane/EtOAc = 9:1, v/v) to afford **S4b** as a yellow oil (2.5 g, 13 mmol, 95 %). R_f: 0.67 (Pentane/EtOAc = 7:3, v/v). ^{1}H NMR (400 MHz, $CDCl_3$) δ 7.08 (app t, 1H), 6.88 (d, J = 8.0 Hz, 1H), 6.79 (s, 1H), 6.61 (t, J = 8.0 Hz, 1H), 3.65 (br s, 2H), 0.26 (s, 9H); spectrum in agreement with literature data.[5] HRMS (ESI$^+$) *m/z* calc. for $[M+H]^+$ ($C_{11}H_{16}NSi^+$): 190.1047, found: 190.1048.

S2. Photochemical and thermal isomerization studied by UV-vis and NMR spectroscopies

S2.1. First hypothesis

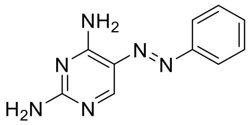

1

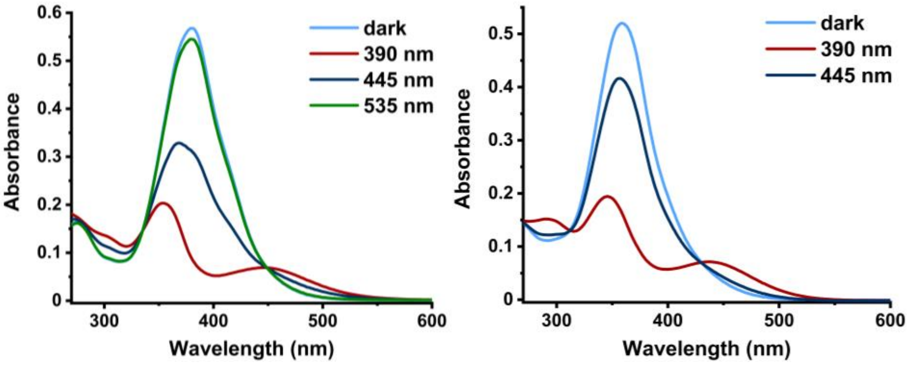

Figure S1. Photoisomerization of **1** in (left) DMSO (20 µM) and (right) eDHFR assay buffer with 1 % DMSO (20 µM).

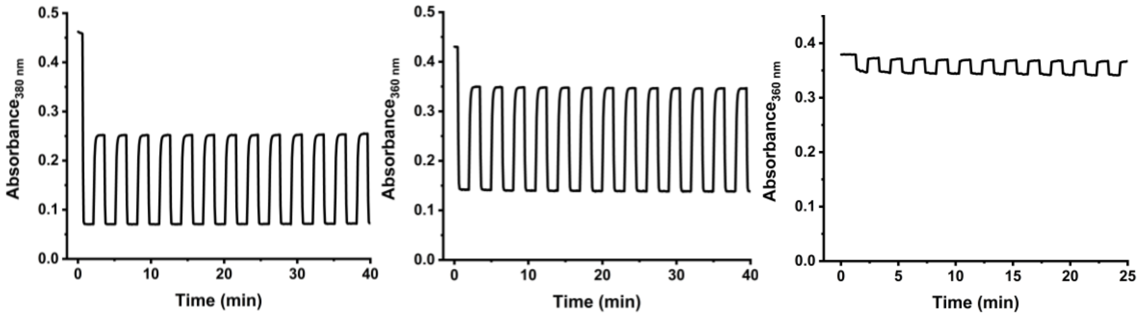

Figure S2. Fatigue studies of **1**. Left: in DMSO (20 µM), using alternating 390 and 445 nm light. Center: in eDHFR assay buffer with 1 % DMSO (20 µM), using alternating 365 and 445 nm light. Right: in eDHFR assay buffer with 1 % DMSO (20 µM) + 10 mM GSH, using alternating 365 and 445 nm light.

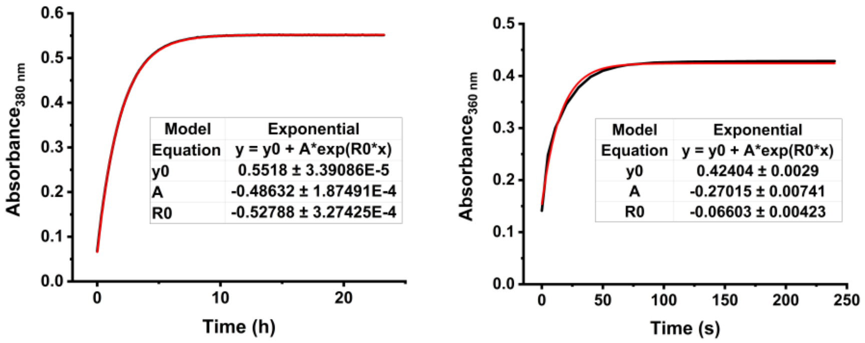

Figure S3. Thermal *cis*-to-*trans* isomerization of **1** in (left) DMSO (25 °C, 20 µM) with $t_{1/2}$ = 1.3 h, and in (right) eDHFR assay buffer with 1 % DMSO (25 °C, 20 µM), with $t_{1/2}$ = 10 s.

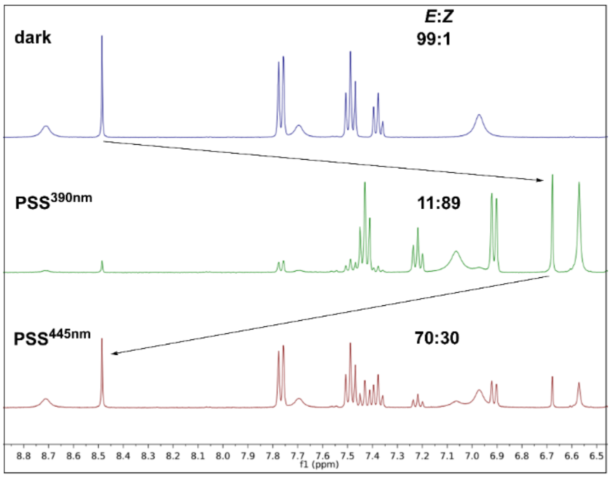

Figure S4. Partial ^{1}H NMR spectra for compound **1** before and after irradiation with 390 and 445 nm light (~2 mM in DMSO-d_6).

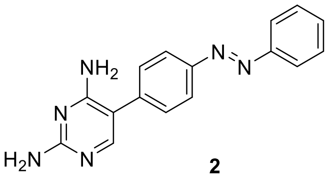

2

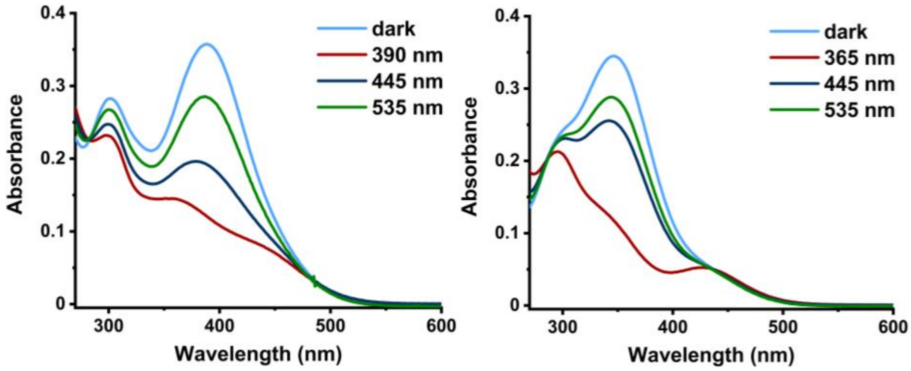

Figure S5. Photoisomerization of **2** in (left) DMSO (20 µM) and (right) eDHFR assay buffer with 1 % DMSO (20 µM).

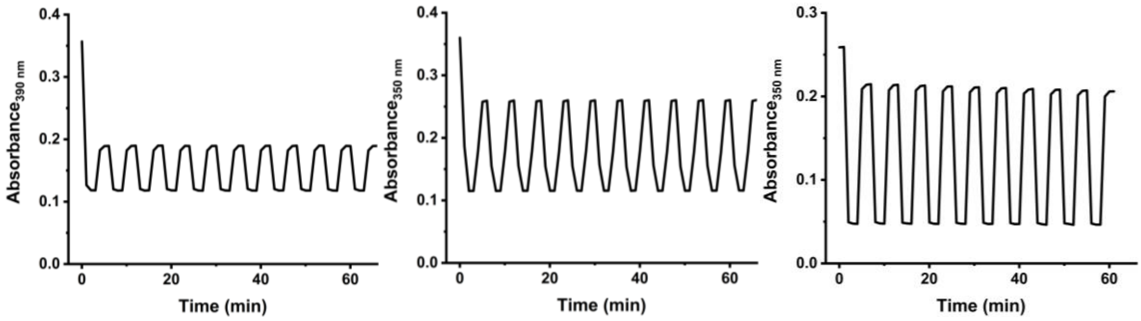

Figure S6. Fatigue studies of **2**. Left: in DMSO (20 µM), using alternating 390 and 445 nm light. Center: in eDHFR assay buffer with 1 % DMSO (20 µM), using alternating 365 and 445 nm light. Right: in eDHFR assay buffer with 1 % DMSO (20 µM) + 10 mM GSH, using alternating 365 and 445 nm light.

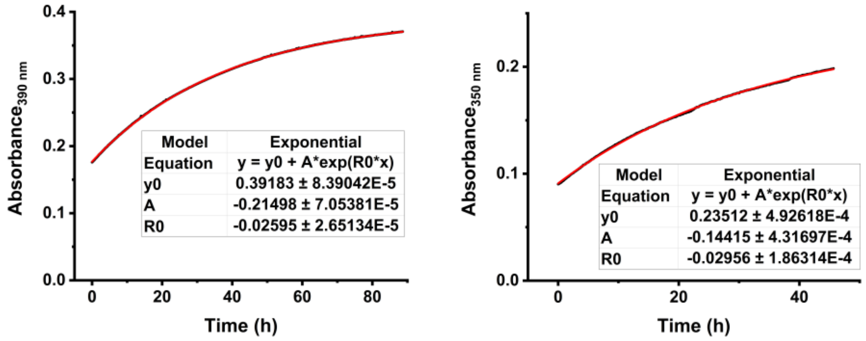

Figure S7. Thermal *cis*-to-*trans* isomerization of **2** in (left) DMSO (25 °C, 20 µM) with $t_{1/2}$ = 27 h, and in (right) eDHFR assay buffer with 1 % DMSO (25 °C, 20 µM), with $t_{1/2}$ = 23 h.

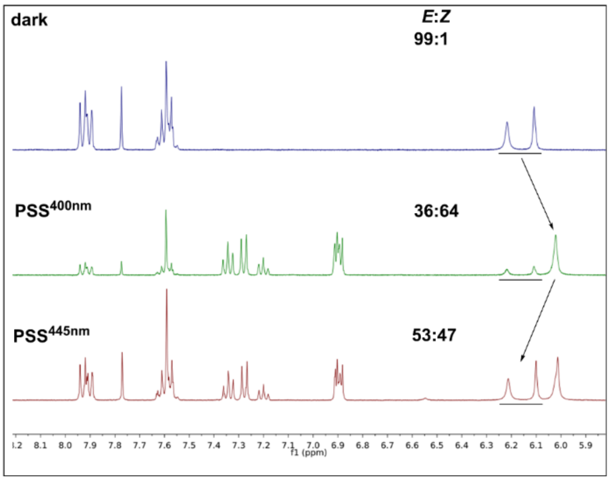

Figure S8. Partial ^{1}H NMR spectra for compound **2** before and after irradiation with 400 and 445 nm light (~2 mM in DMSO-d_6).

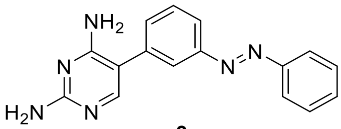

3

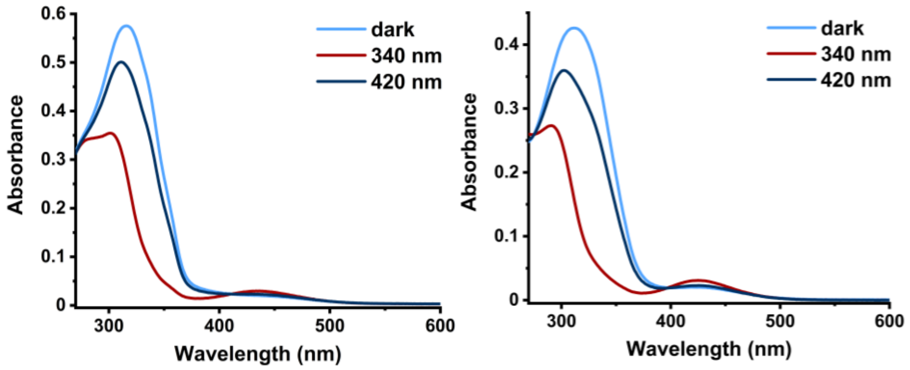

Figure S9. Photoisomerization of **3** in (left) DMSO (20 µM) and (right) eDHFR assay buffer with 1 % DMSO (20 µM).

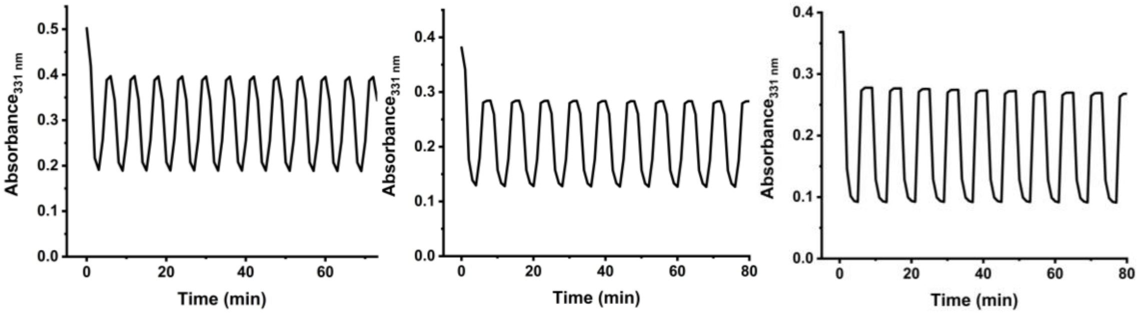

Figure S10. Fatigue studies of **3** using alternating 365 and 420 nm light. Left: in DMSO (20 µM). Center: in eDHFR assay buffer with 1 % DMSO (20 µM). Right: in eDHFR assay buffer with 1 % DMSO (20 µM) + 10 mM GSH.

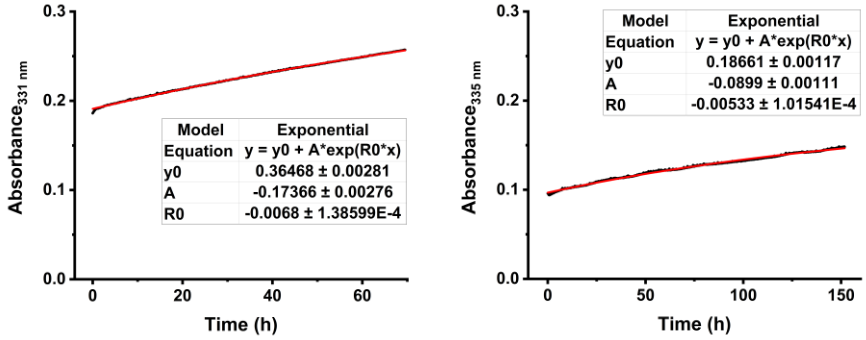

Figure S11. Thermal *cis*-to-*trans* isomerization of **3** in (left) DMSO (25 °C, 20 µM) with $t_{1/2}$ > 24 h, and in (right) eDHFR assay buffer with 1 % DMSO (25 °C, 20 µM), with $t_{1/2}$ > 24 h.

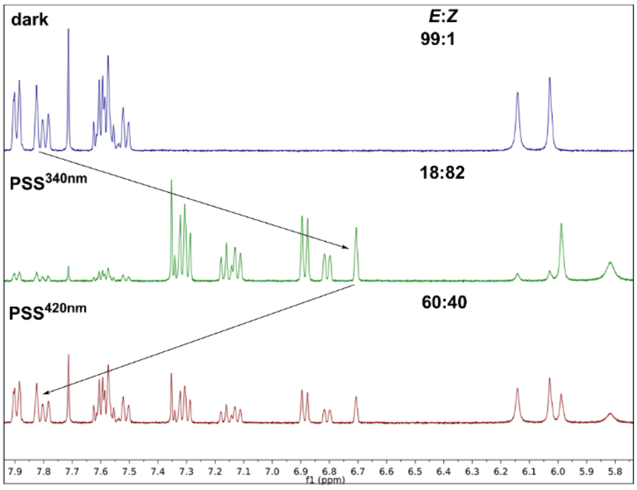

Figure S12. Partial ^{1}H NMR spectra for compound **3** before and after irradiation with 340 and 420 nm light (~2 mM in DMSO-*d*$_6$).

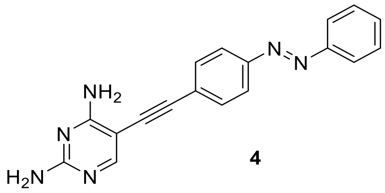

4

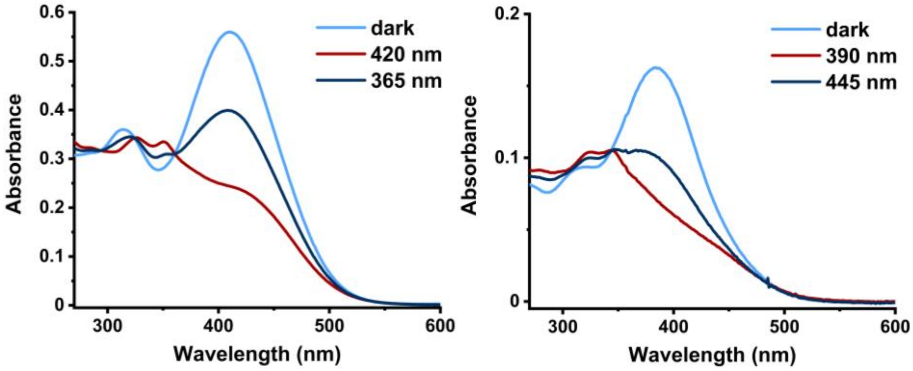

Figure S13. Photoisomerization of **4** in (left) DMSO (20 µM) and (right) eDHFR assay buffer with 50 % DMSO (5 µM).

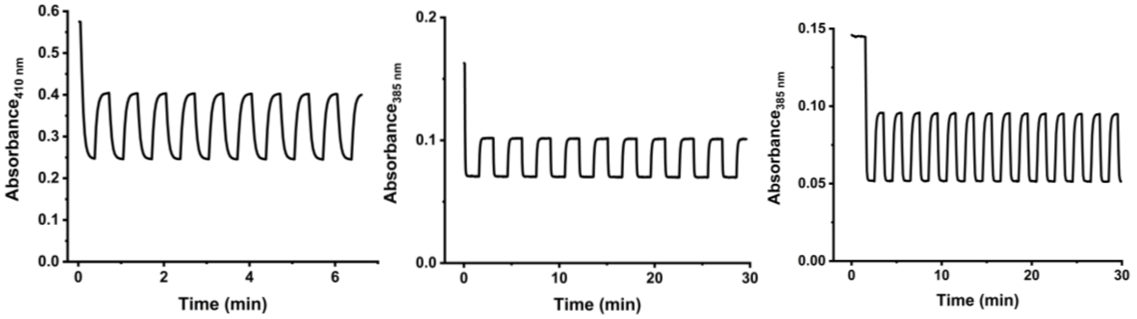

Figure S14. Fatigue studies of **4**. Left: in DMSO (20 µM), using alternating 420 and 365 nm light. Center: in eDHFR assay buffer with 50 % DMSO (5 µM), using alternating 390 and 445 nm light. Right: in eDHFR assay buffer with 50 % DMSO (5 µM) + 10 mM GSH, using alternating 390 and 445 nm light.

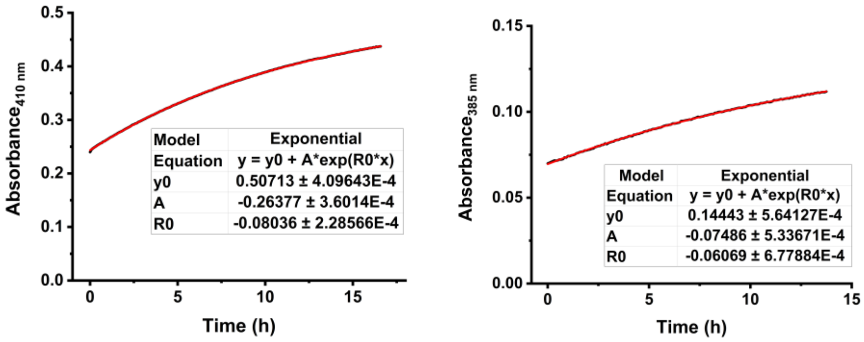

Figure S15. Thermal *cis*-to-*trans* isomerization of **4** in (left) DMSO (25 °C, 20 µM) with $t_{1/2}$ = 8.6 h, and in (right) eDHFR assay buffer with 50 % DMSO (25 °C, 5 µM), with $t_{1/2}$ = 11 h.

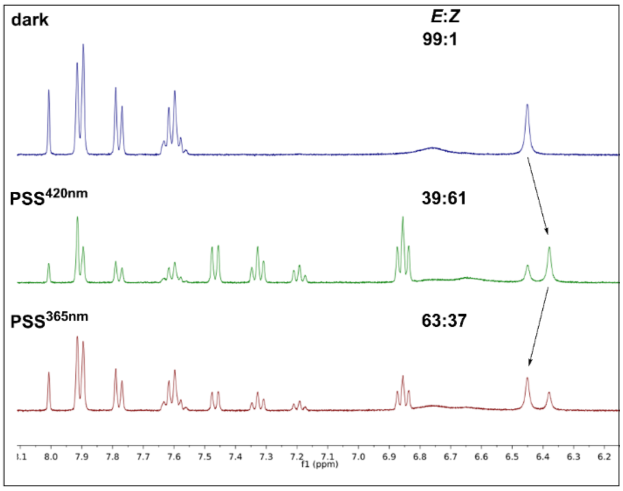

Figure S16. Partial ^{1}H NMR spectra for compound **4** before and after irradiation with 420 and 365 nm light (~2 mM in DMSO-d_6).

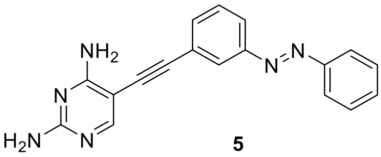

5

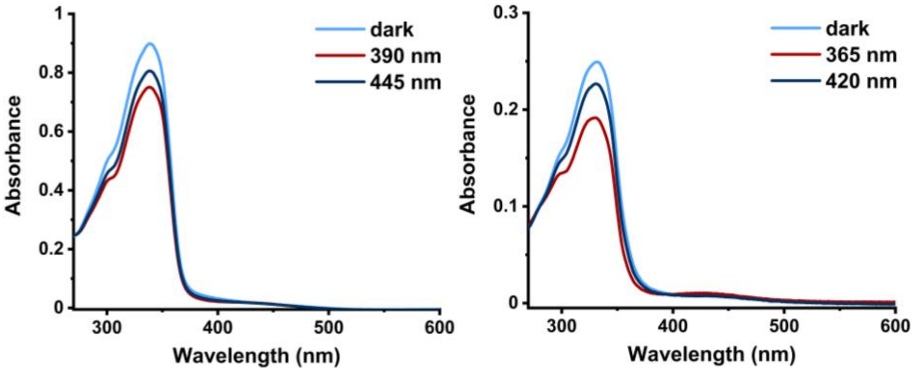

Figure S17. Photoisomerization of **5** in (left) DMSO (20 µM) and (right) eDHFR assay buffer with 50 % DMSO (5 µM).

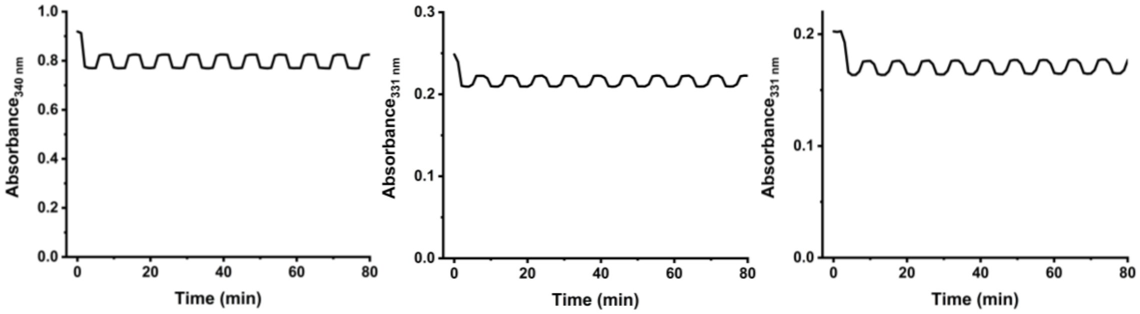

Figure S18. Fatigue studies of **5** using alternating 390 and 445 nm light. Left: in DMSO (20 µM). Center: in eDHFR assay buffer with 50 % DMSO (5 µM). Right: in eDHFR assay buffer with 50 % DMSO (5 µM) + 10 mM GSH.

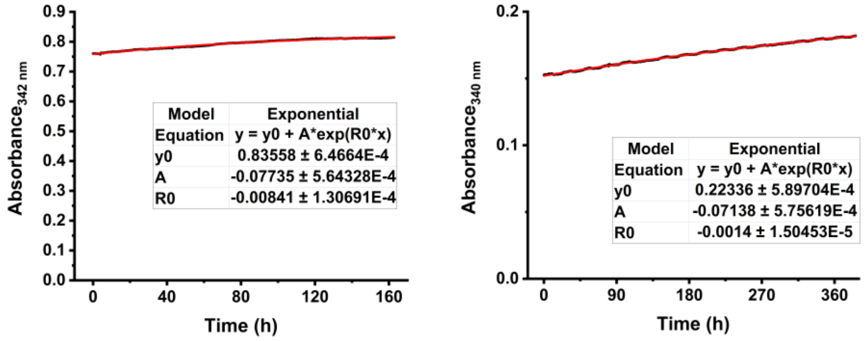

Figure S19. Thermal *cis*-to-*trans* isomerization of **5** in (left) DMSO (25 °C, 20 µM) with $t_{1/2}$ > 24 h, and in (right) eDHFR assay buffer with 50 % DMSO (25 °C, 5 µM), with $t_{1/2}$ > 24 h.

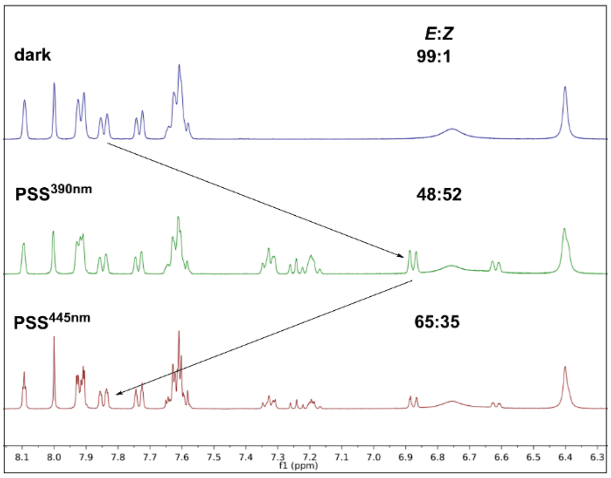

Figure S20. Partial ^{1}H NMR spectra for compound **5** before and after irradiation with 390 and 445 nm light (~2 mM in DMSO-d_6).

S2.2. Second hypothesis

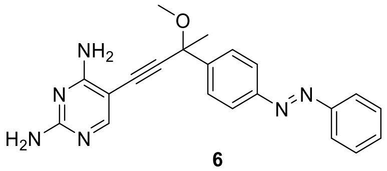

6

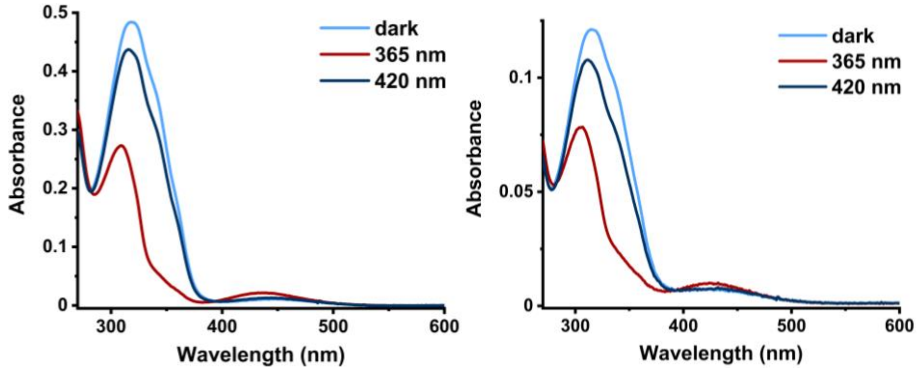

Figure S21. Photoisomerization of **6** in (left) DMSO (20 µM) and (right) eDHFR assay buffer with 20 % DMSO (5 µM).

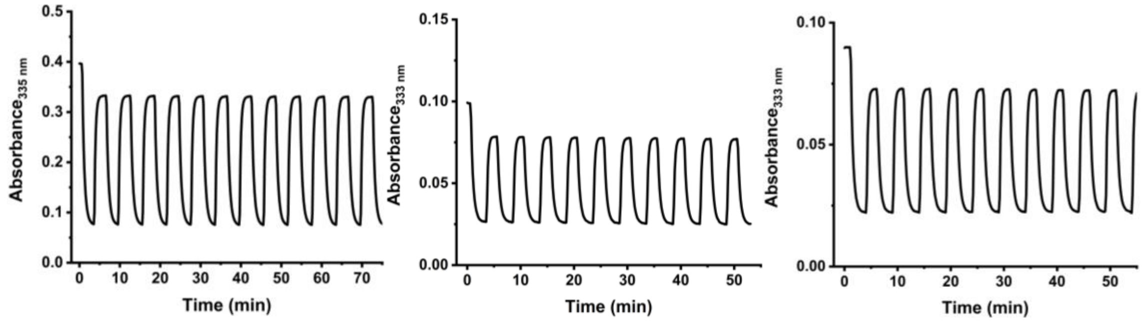

Figure S22. Fatigue studies of **6** using alternating 365 and 420 nm light. Left: in DMSO (20 µM). Center: in eDHFR assay buffer with 20 % DMSO (5 µM). Right: in eDHFR assay buffer with 20 % DMSO (5 µM) + 10 mM GSH.

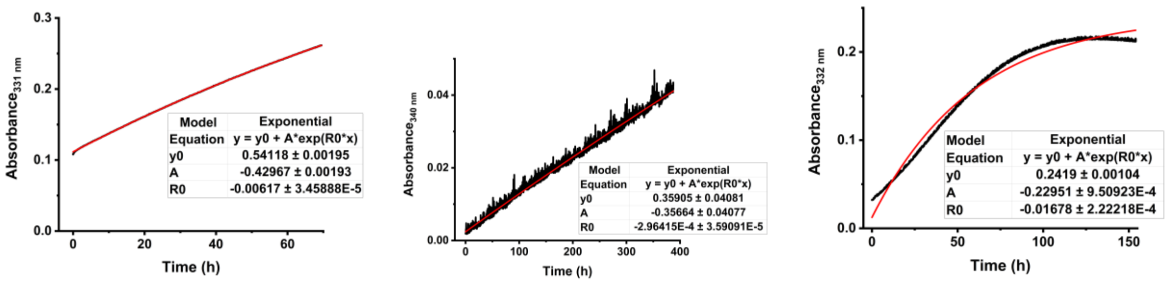

Figure S23. Thermal *cis*-to-*trans* isomerization of **6** in (left) DMSO (25 °C, 20 µM) with $t_{1/2}$ > 24 h, in (center) eDHFR assay buffer with 20 % DMSO (25 °C, 5 µM), with $t_{1/2}$ > 24 h, and in (right) Tris buffer with 20 % DMSO (37 °C, 5 µM), with $t_{1/2}$ > 24 h.

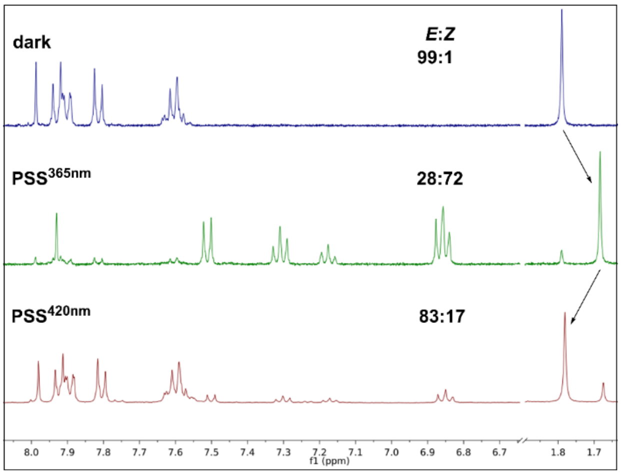

Figure S24. Partial ^1H NMR spectra for compound **6** before and after irradiation with 365 and 420 nm light (~2 mM in DMSO-d_6).

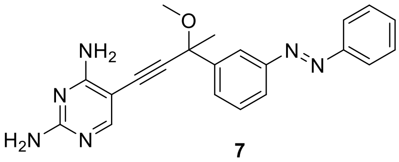

7

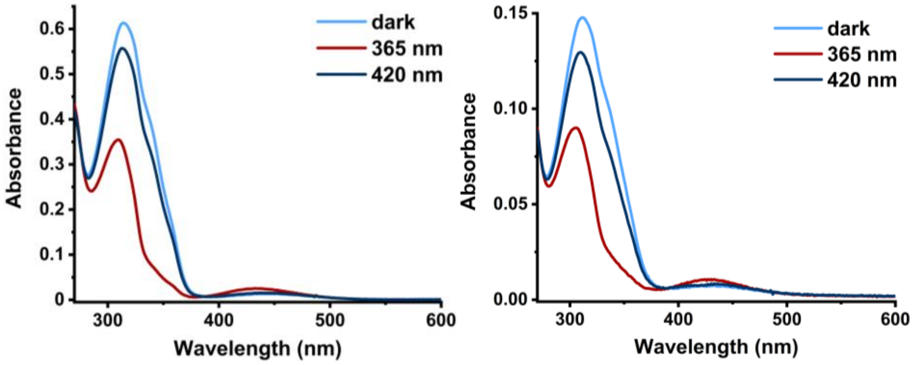

Figure S25. Photoisomerization of **7** in (left) DMSO (20 µM) and (right) eDHFR assay buffer with 20 % DMSO (5 µM).

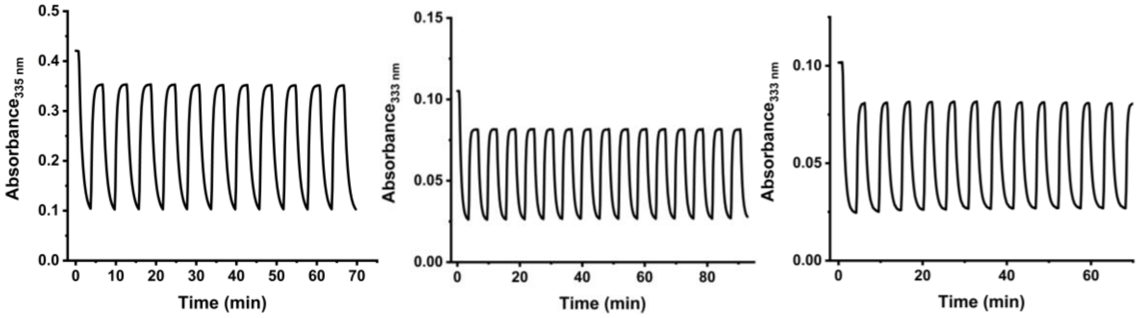

Figure S26. Fatigue studies of **7** using alternating 365 and 420 nm light. Left: in DMSO (20 µM). Center: in eDHFR assay buffer with 20 % DMSO (5 µM). Right: in eDHFR assay buffer with 20 % DMSO (5 µM) + 10 mM GSH.

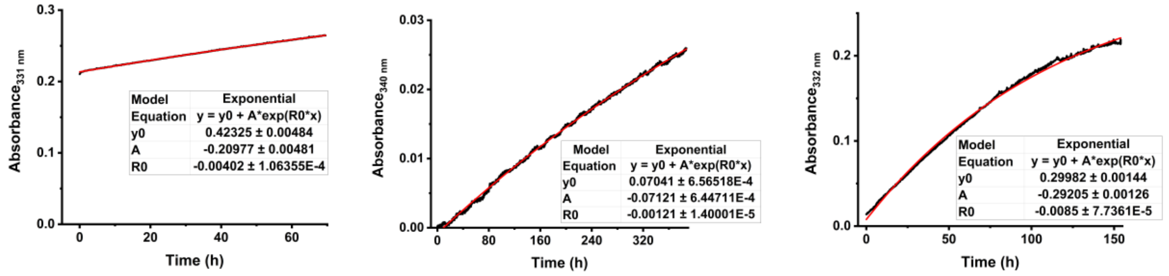

Figure S27. Thermal *cis*-to-*trans* isomerization of **7** in (left) DMSO (25 °C, 20 µM) with $t_{1/2}$ > 24 h, in (center) eDHFR assay buffer with 20 % DMSO (25 °C, 5 µM), with $t_{1/2}$ > 24 h, and in (right) Tris buffer with 20 % DMSO (37 °C, 5 µM), with $t_{1/2}$ > 24 h.

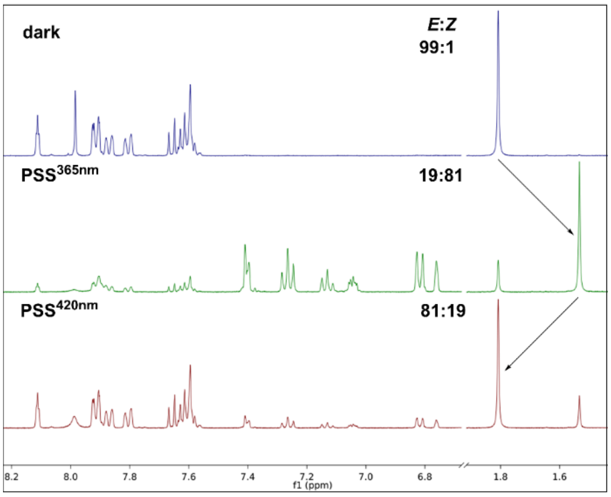

Figure S28. Partial ^{1}H NMR spectra for compound **7** before and after irradiation with 365 and 420 nm light (~2 mM in DMSO-d_6).

S2.3. Third hypothesis

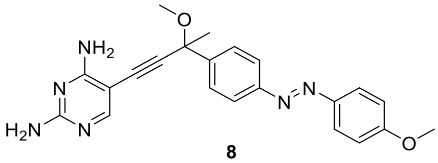

8

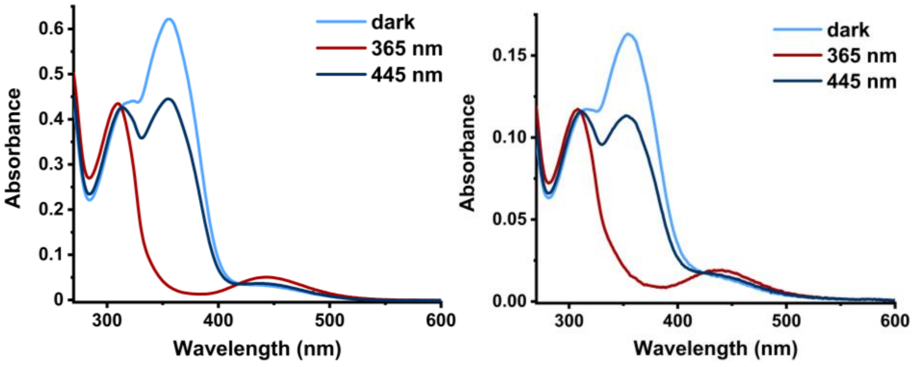

Figure S29. Photoisomerization of **8** in (left) DMSO (20 µM) and (right) eDHFR assay buffer with 50 % DMSO (5 µM).

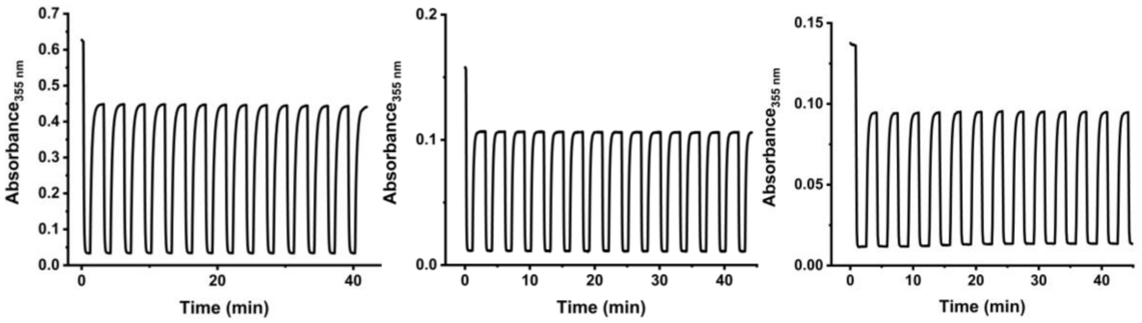

Figure S30. Fatigue studies of **8** using alternating 365 and 445 nm light. Left: in DMSO (20 µM). Center: in eDHFR assay buffer with 50 % DMSO (5 µM). Right: in eDHFR assay buffer with 50 % DMSO (5 µM) + 10 mM GSH.

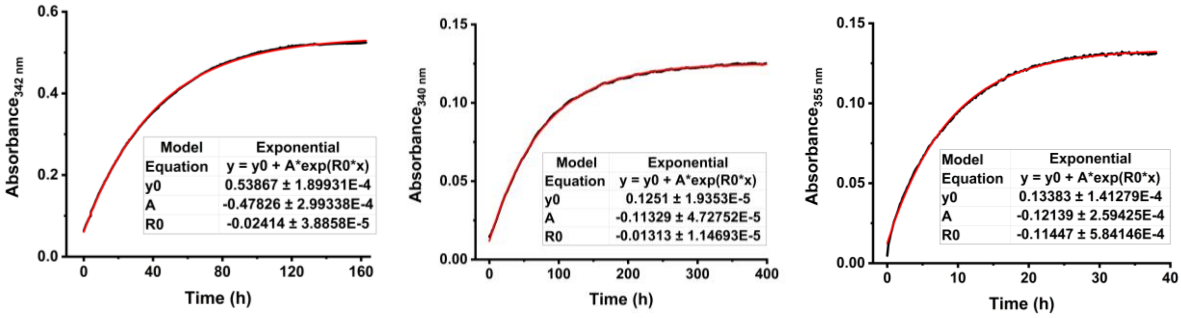

Figure S31. Thermal *cis*-to-*trans* isomerization of **8** in (left) DMSO (25 °C, 20 µM) with $t_{1/2}$ > 24 h, in (center) eDHFR assay buffer with 50 % DMSO (25 °C, 5 µM), with $t_{1/2}$ > 24 h, and in (right) Tris buffer with 50 % DMSO (37 °C, 5 µM), with $t_{1/2}$ = 6 h.

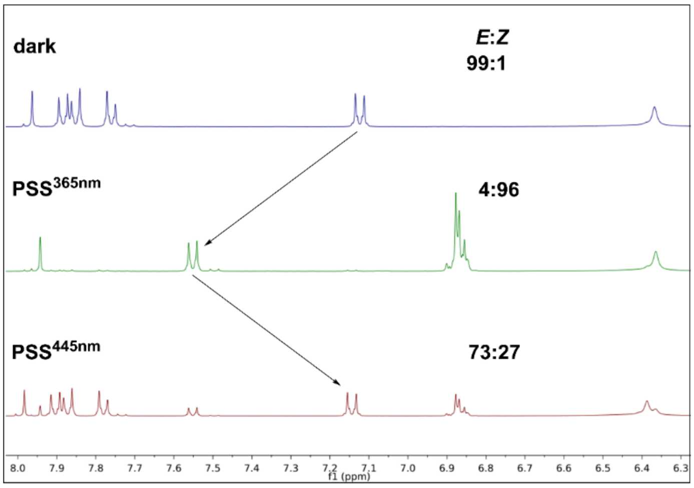

Figure S32. Partial ^{1}H NMR spectra for compound **8** before and after irradiation with 365 and 445 nm light (~2 mM in DMSO-d_6).

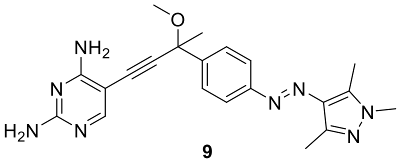

9

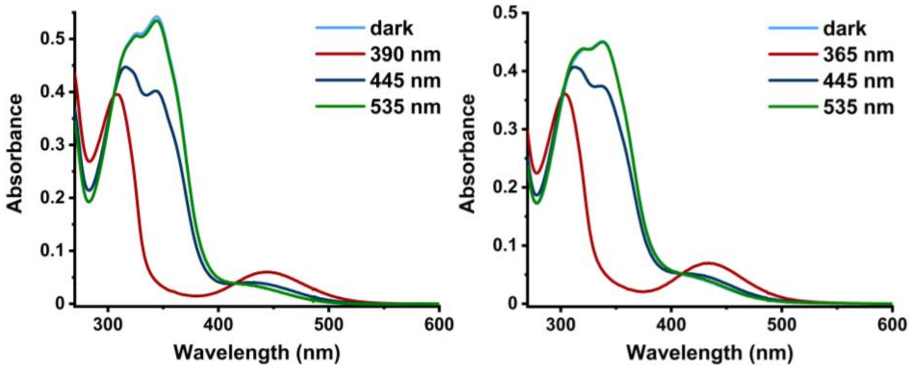

Figure S33. Photoisomerization of **9** in (left) DMSO (20 µM) and (right) eDHFR assay buffer with 1 % DMSO (20 µM).

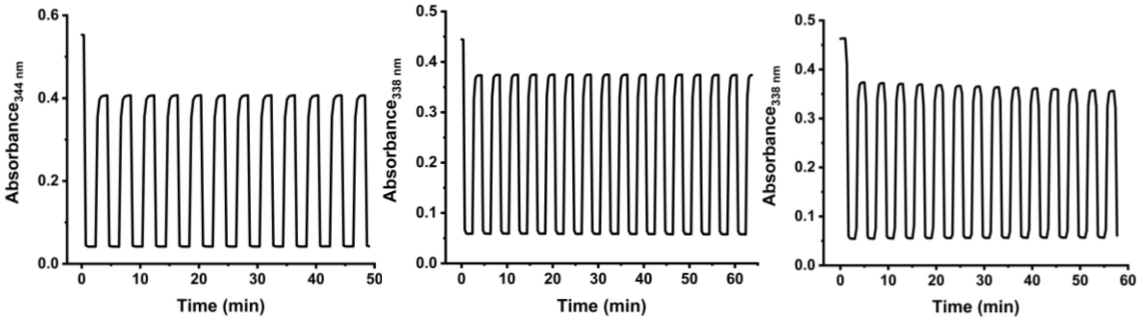

Figure S34. Fatigue studies of **9** using alternating 365 and 445 nm light. Left: in DMSO (20 µM). Center: in eDHFR assay buffer with 1 % DMSO (20 µM). Right: in eDHFR assay buffer with 1 % DMSO (20 µM) + 10 mM GSH.

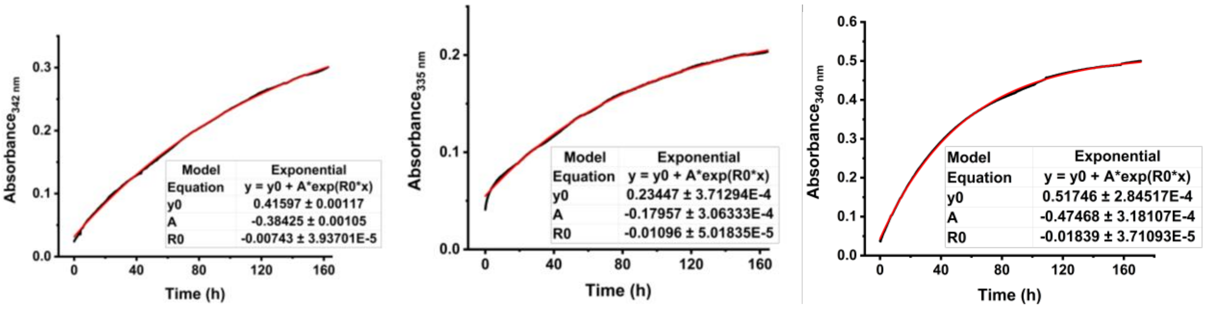

Figure S35. Thermal *cis*-to-*trans* isomerization of **9** in (left) DMSO (25 °C, 20 µM) with $t_{1/2}$ > 24 h, in (center) eDHFR assay buffer with 1 % DMSO (25 °C, 20 µM), with $t_{1/2}$ > 24 h, and in (right) Tris buffer with 1 % DMSO (37 °C, 20 µM), with $t_{1/2}$ > 24 h.

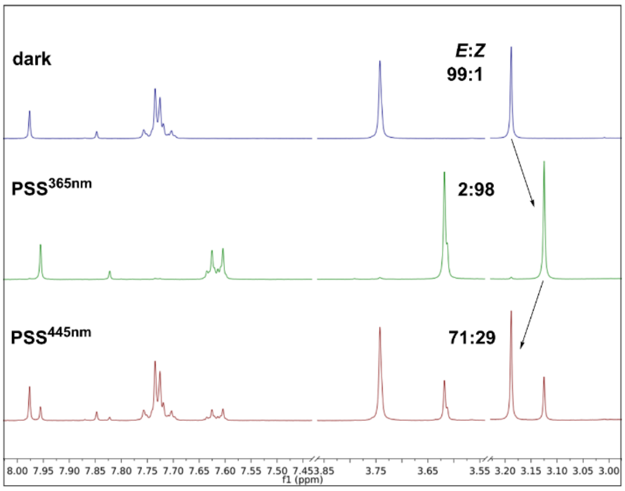

Figure S36. Partial ^{1}H NMR spectra for compound **9** before and after irradiation with 365 and 445 nm light (~2 mM in DMSO-d6).

S2.4. Fourth hypothesis

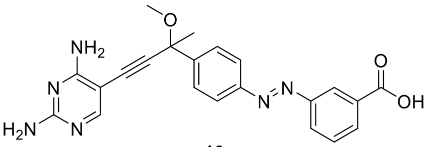

10

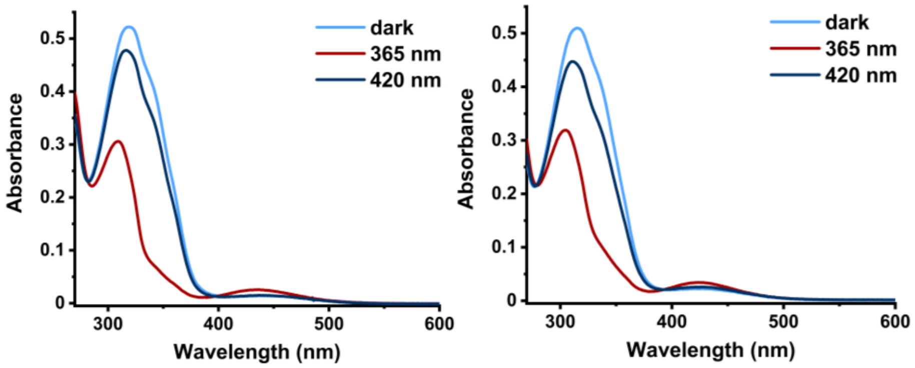

Figure S37. Photoisomerization of **10** in (left) DMSO (20 µM) and (right) eDHFR assay buffer with 1 % DMSO (20 µM).

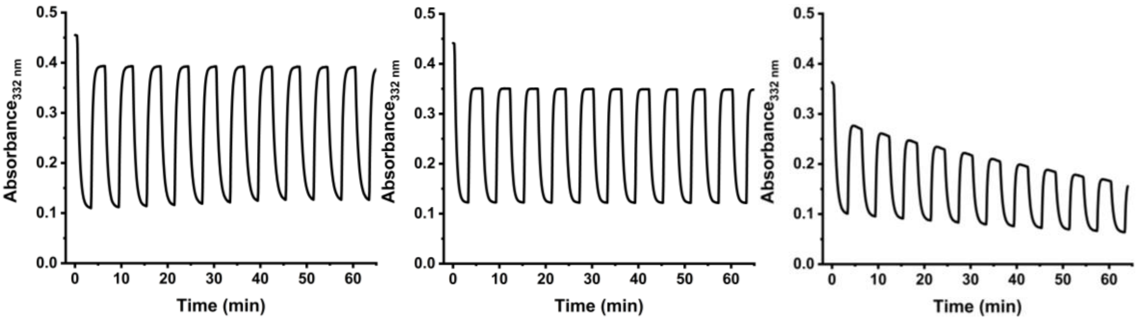

Figure S38. Fatigue studies of **10** using alternating 365 and 420 nm light. Left: in DMSO (20 µM). Center: in eDHFR assay buffer with 1 % DMSO (20 µM). Right: in eDHFR assay buffer with 1 % DMSO (20 µM) + 10 mM GSH.

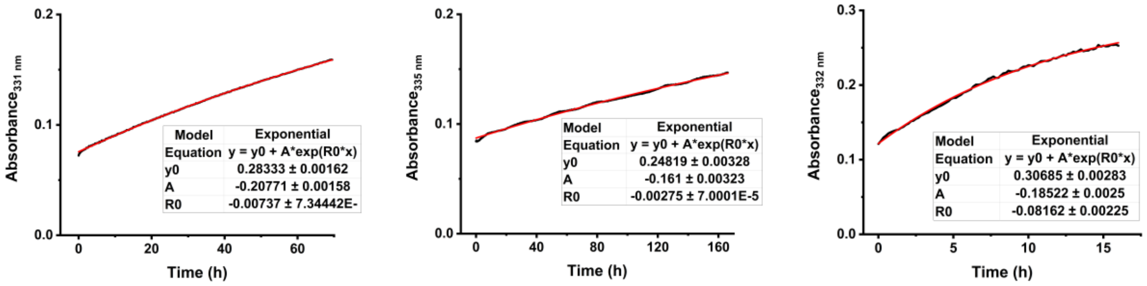

Figure S39. Thermal *cis*-to-*trans* isomerization of **10** in (left) DMSO (25 °C, 20 µM) with $t_{1/2}$ > 24 h, in (center) eDHFR assay buffer with 1 % DMSO (25 °C, 20 µM), with $t_{1/2}$ > 24 h, and in (right) Tris buffer with 1 % DMSO (37 °C, 20 µM), with $t_{1/2}$ = 8.5 h.

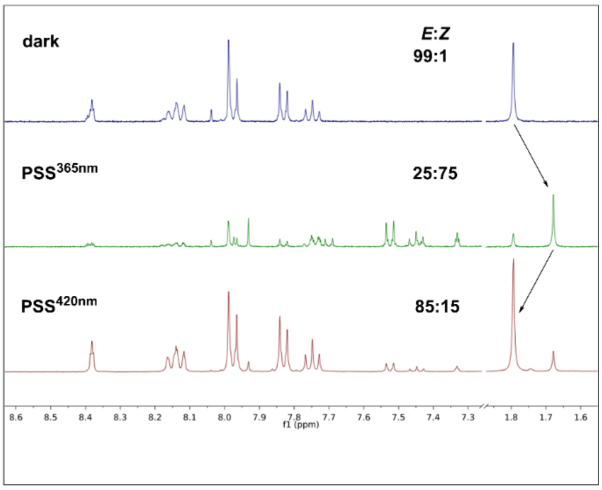

Figure S40. Partial ^{1}H NMR spectra for compound **10** before and after irradiation with 365 and 420 nm light (~2 mM in DMSO-d_6).

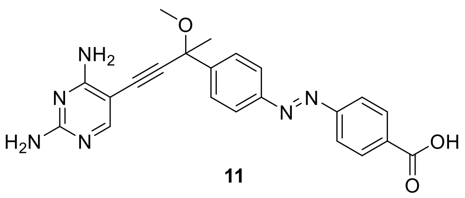

11

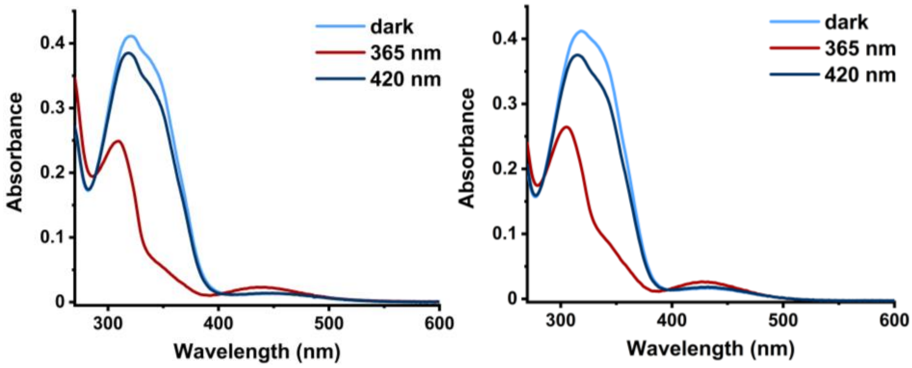

Figure S41. Photoisomerization of **11** in (left) DMSO (20 µM) and (right) eDHFR assay buffer with 5 % DMSO (20 µM).

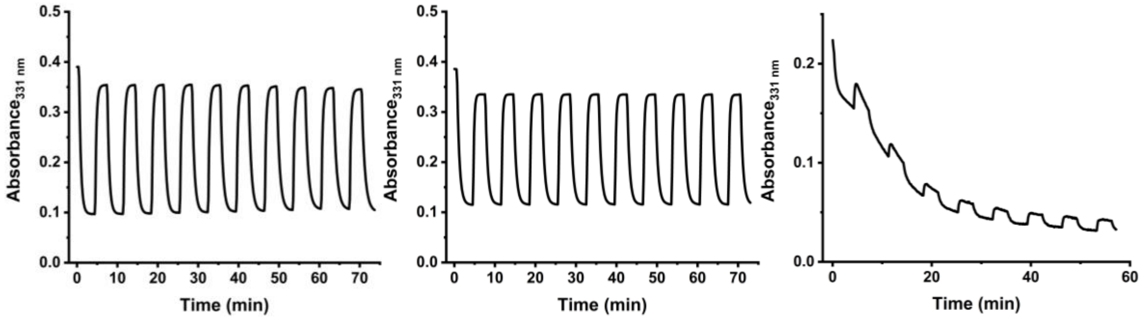

Figure S42. Fatigue studies of **11** using alternating 365 and 420 nm light. Left: in DMSO (20 µM). Center: in eDHFR assay buffer with 5 % DMSO (20 µM). Right: in eDHFR assay buffer with 5 % DMSO (20 µM) + 10 mM GSH.

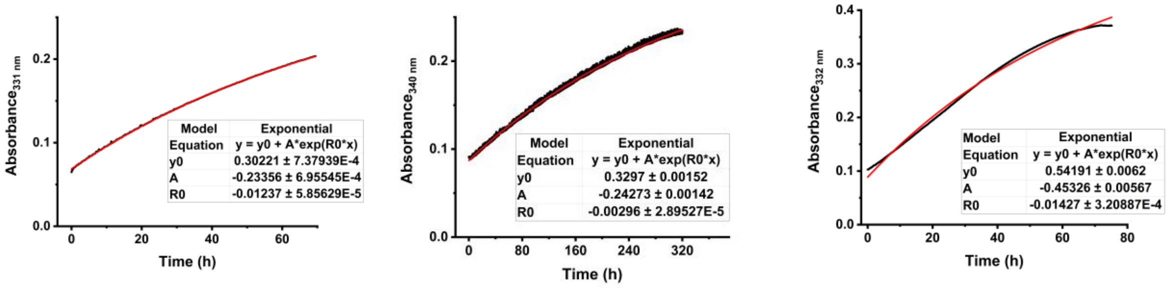

Figure S43. Thermal *cis*-to-*trans* isomerization of **11** in (left) DMSO (25 °C, 20 µM) with $t_{1/2}$ > 24 h, in (center) eDHFR assay buffer with 5 % DMSO (25 °C, 20 µM), with $t_{1/2}$ > 24 h, and in (right) Tris buffer with 5 % DMSO (37 °C, 20 µM), with $t_{1/2}$ > 24 h.

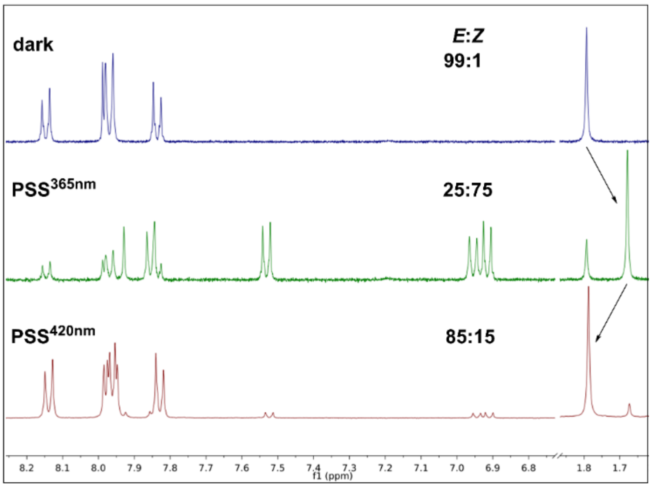

Figure S44. Partial ^1H NMR spectra for compound **11** before and after irradiation with 365 and 420 nm light (~2 mM in DMSO-d_6).

S2.5. Fifth hypothesis

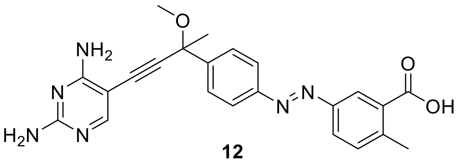

12

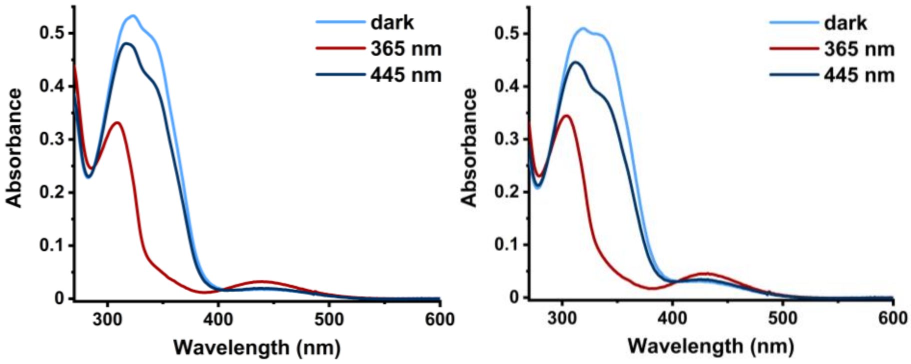

Figure S45. Photoisomerization of **12** in (left) DMSO (20 µM) and (right) eDHFR assay buffer with 1 % DMSO (20 µM).

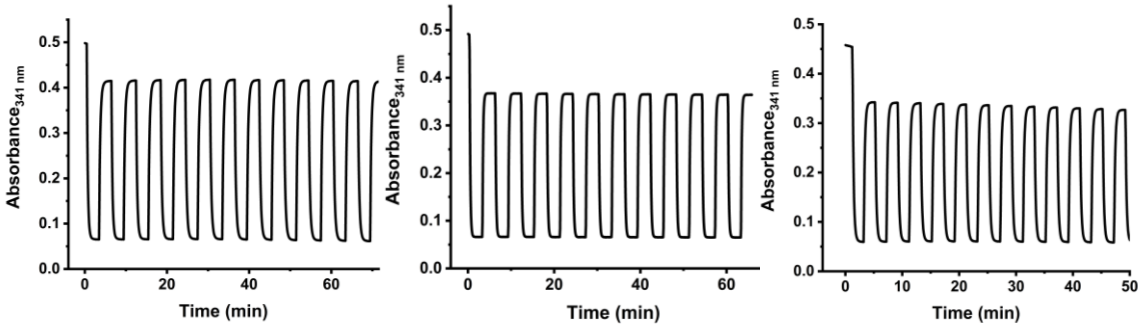

Figure S46. Fatigue studies of **12** using alternating 365 and 420 nm light. Left: in DMSO (20 µM). Center: in eDHFR assay buffer with 1 % DMSO (20 µM). Right: in eDHFR assay buffer with 1 % DMSO (20 µM) + 10 mM GSH.

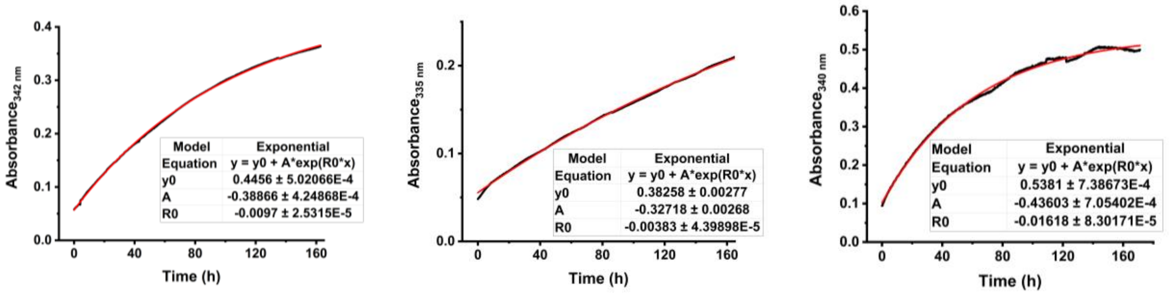

Figure S47. Thermal *cis*-to-*trans* isomerization of **12** in (left) DMSO (25 °C, 20 µM) with $t_{1/2}$ > 24 h, in (center) eDHFR assay buffer with 1 % DMSO (25 °C, 20 µM), with $t_{1/2}$ > 24 h, and in (right) Tris buffer with 1 % DMSO (37 °C, 20 µM), with $t_{1/2}$ > 24 h.

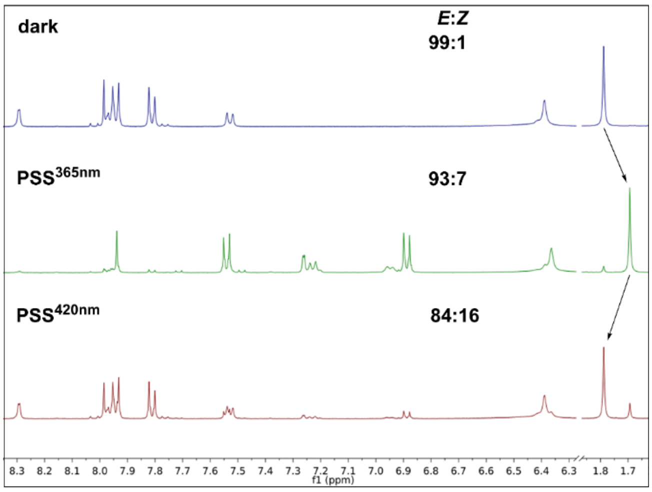

Figure S48. Partial ^{1}H NMR spectra for compound **12** before and after irradiation with 365 and 420 nm light (~2 mM in DMSO-d_6).

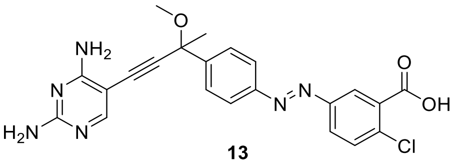

13

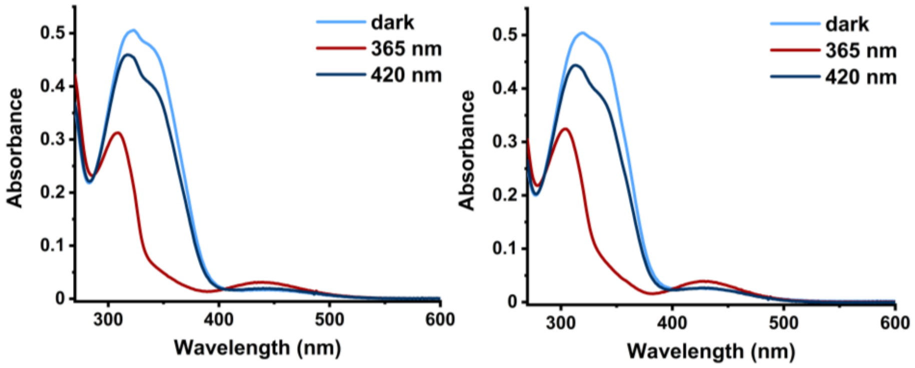

Figure S49. Photoisomerization of **13** in (left) DMSO (20 µM) and (right) eDHFR assay buffer with 1 % DMSO (20 µM).

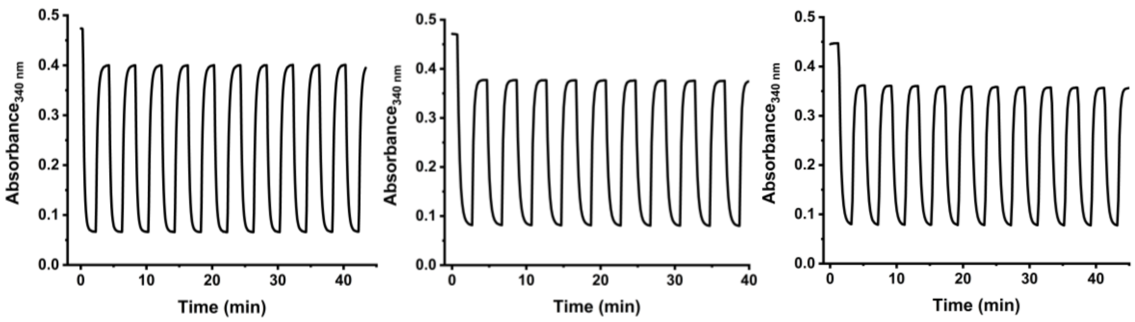

Figure S50. Fatigue studies of **13** using alternating 365 and 420 nm light. Left: in DMSO (20 µM). Center: in eDHFR assay buffer with 1 % DMSO (20 µM). Right: in eDHFR assay buffer with 1 % DMSO (20 µM) + 10 mM GSH.

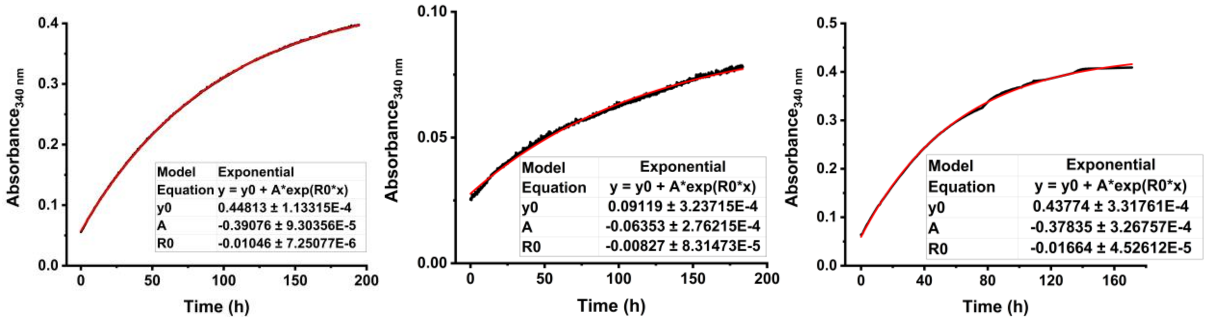

Figure S51. Thermal *cis*-to-*trans* isomerization of **13** in (left) DMSO (25 °C, 20 µM) with $t_{1/2}$ > 24 h, in (center) eDHFR assay buffer with 1 % DMSO (25 °C, 20 µM), with $t_{1/2}$ > 24 h, and in (right) Tris buffer with 1 % DMSO (37 °C, 20 µM), with $t_{1/2}$ > 24 h.

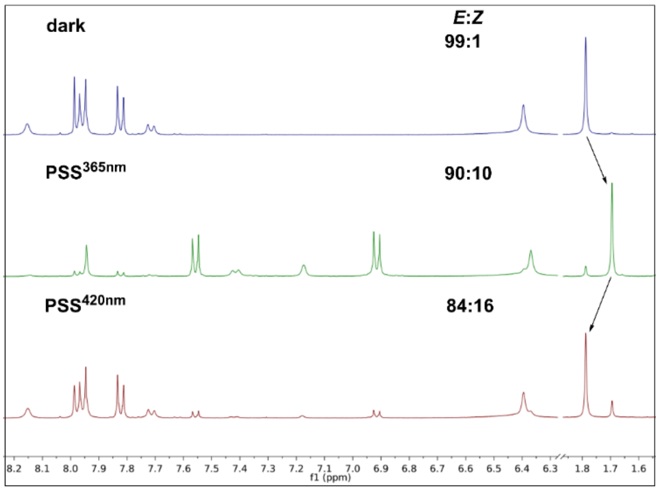

Figure S52. Partial ^{1}H NMR spectra for compound **13** before and after irradiation with 365 and 420 nm light (~2 mM in DMSO-d_6).

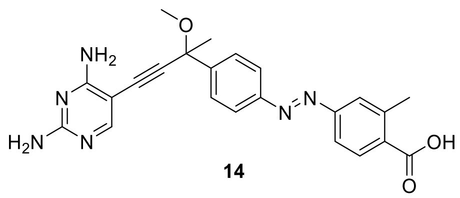

14

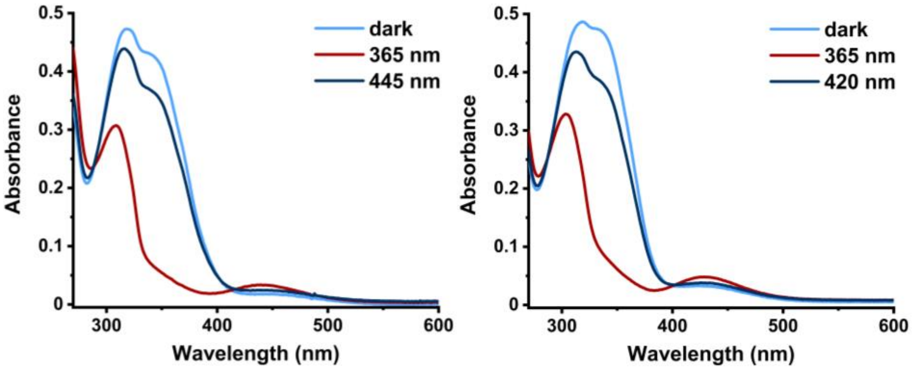

Figure S53. Photoisomerization of **14** in (left) DMSO (20 µM) and (right) eDHFR assay buffer with 1 % DMSO (20 µM).

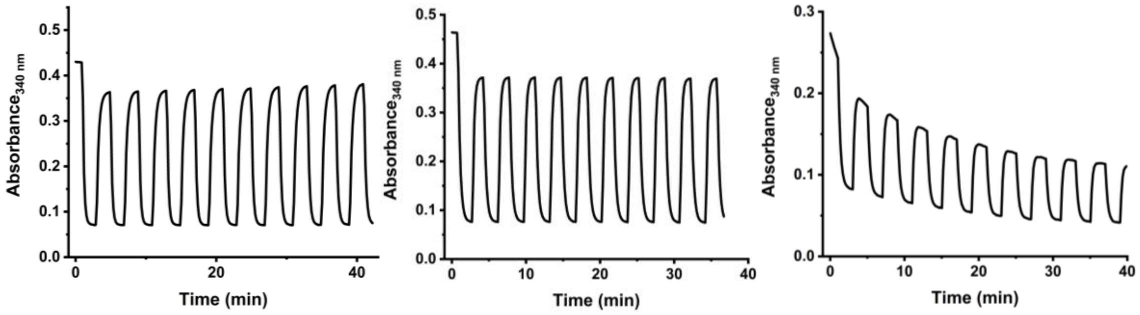

Figure S54. Fatigue studies of **14** using alternating 365 and 445 nm light. Left: in DMSO (20 µM). Center: in eDHFR assay buffer with 1 % DMSO (20 µM). Right: in eDHFR assay buffer with 1 % DMSO (20 µM) + 10 mM GSH.

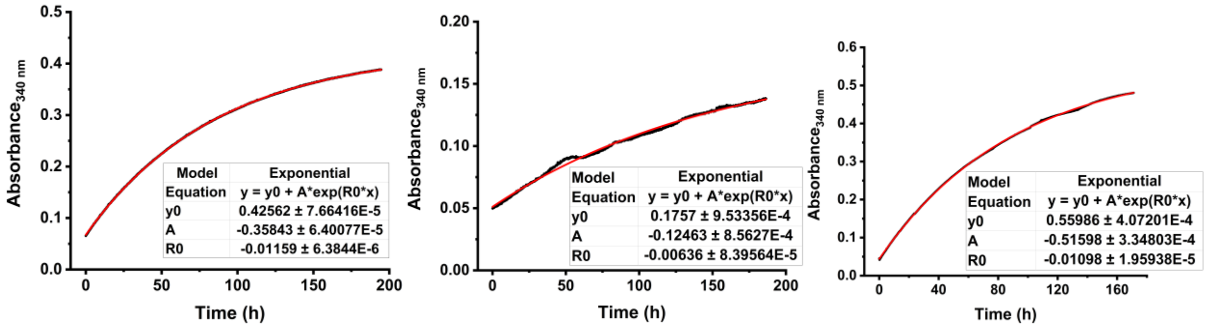

Figure S55. Thermal *cis*-to-*trans* isomerization of **14** in (left) DMSO (25 °C, 20 µM) with $t_{1/2}$ > 24 h, in (center) eDHFR assay buffer with 1 % DMSO (25 °C, 20 µM), with $t_{1/2}$ > 24 h, and in (right) Tris buffer with 1 % DMSO (37 °C, 20 µM), with $t_{1/2}$ > 24 h.

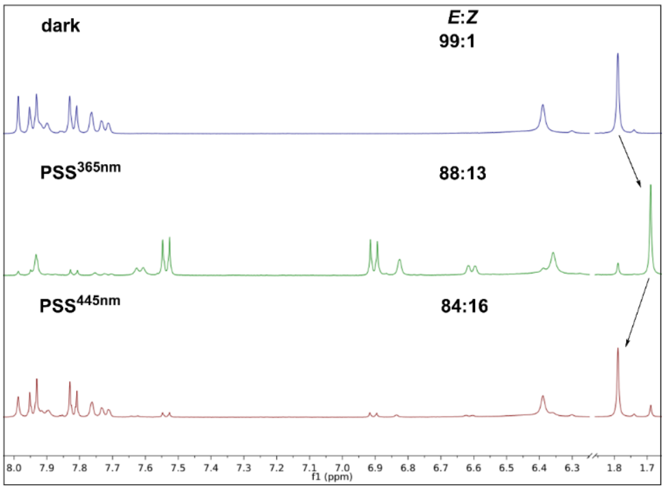

Figure S56. Partial ^{1}H NMR spectra for compound **14** before and after irradiation with 365 and 420 nm light (~2 mM in DMSO-d_6).

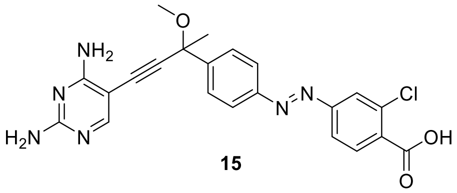

15

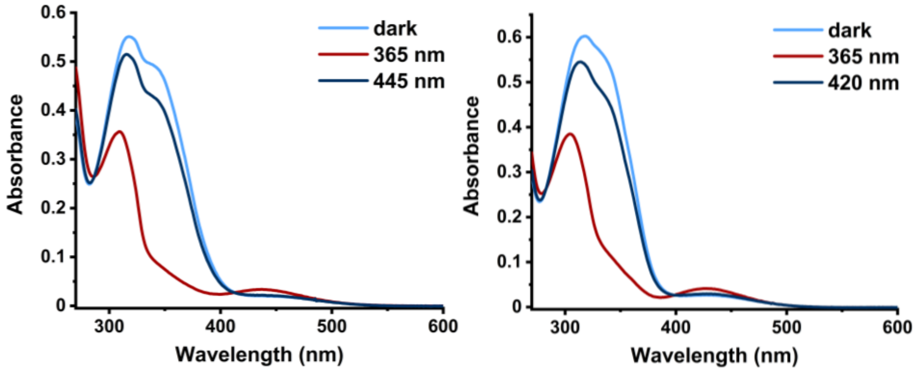

Figure S57. Photoisomerization of **15** in (left) DMSO (20 µM) and (right) eDHFR assay buffer with 1 % DMSO (20 µM).

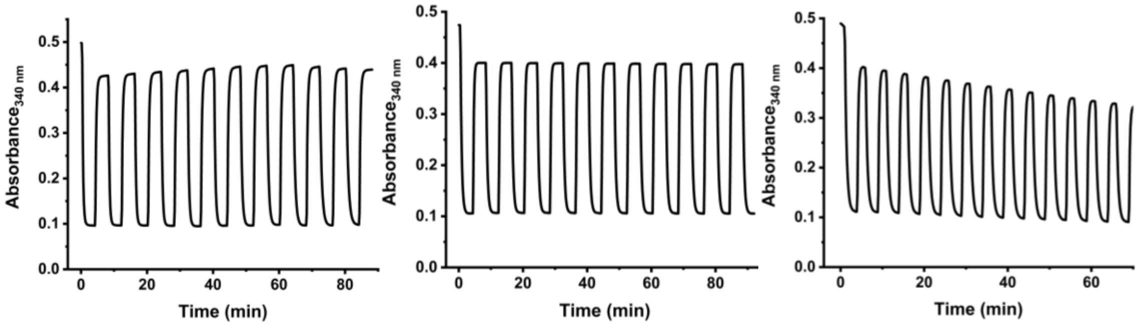

Figure S58. Fatigue studies of **15** using alternating 365 and 420 nm light. Left: in DMSO (20 µM). Center: in eDHFR assay buffer with 1 % DMSO (20 µM). Right: in eDHFR assay buffer with 1 % DMSO (20 µM) + 10 mM GSH.

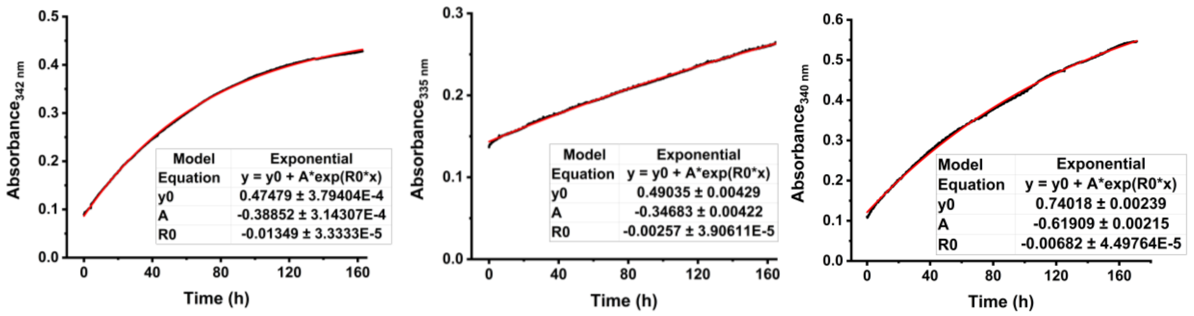

Figure S59. Thermal *cis*-to-*trans* isomerization of **15** in (left) DMSO (25 °C, 20 µM) with $t_{1/2}$ > 24 h, in (center) eDHFR assay buffer with 1 % DMSO (25 °C, 20 µM), with $t_{1/2}$ > 24 h, and in (right) Tris buffer with 1 % DMSO (37 °C, 20 µM), with $t_{1/2}$ > 24 h.

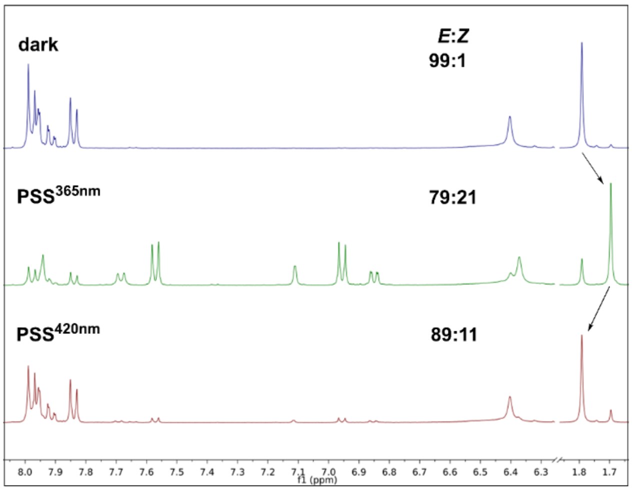

Figure S60. Partial ^{1}H NMR spectra for compound **15** before and after irradiation with 365 and 420 nm light (~2 mM in DMSO-d_6).

S2.6. Overviews of thermal half-lives and PSDs

Table S1. Overview of the thermal lifetimes in DMSO (20 µM), DHFR assay buffer at 25 °C and Tris buffer at 37 °C (both with different concentrations and DMSO %).

Compound	$t_{1/2}$ DMSO (25 °C)	$t_{1/2}$ Buffer (25 °C)	$t_{1/2}$ Buffer (37 °C)	Buffer conditions
1	1.3 h	10 s	n.d.	20 µM, 1 % DMSO
2	27 h	23 h	n.d.	20 µM, 1 % DMSO
3	> 24 h	> 24 h	n.d.	20 µM, 1 % DMSO
4	8.6 h	11 h	n.d.	5 µM, 50 % DMSO
5	> 24 h	> 24 h	n.d.	5 µM, 50 % DMSO
6	> 24 h	> 24 h	> 24 h	5 µM, 20 % DMSO
7	> 24 h	> 24 h	> 24 h	5 µM, 20 % DMSO
8	> 24 h	> 24 h	6 h	5 µM, 50 % DMSO
9	> 24 h	> 24 h	> 24 h	20 µM, 1 % DMSO
10	> 24 h	> 24 h	8.5 h	20 µM, 1 % DMSO
11	> 24 h	> 24 h	> 24 h	20 µM, 5 % DMSO
12	> 24 h	> 24 h	> 24 h	20 µM, 1 % DMSO
13	> 24 h	> 24 h	> 24 h	20 µM, 1 % DMSO
14	> 24 h	> 24 h	> 24 h	20 µM, 1 % DMSO
15	> 24 h	> 24 h	> 24 h	20 µM, 1 % DMSO

Table S2. Overview of the PSDs in DMSO-d_6 (~2 mM).

Compound	λ_1 (nm)	% cis at λ_1	λ_2 (nm)	% cis at λ_2
1	390	89%	445	30%
2	400	64%	445	47%
3	340	82%	420	40%
4	420	61%	365	37%
5	390	52%	445	35%
6	365	72%	420	17%
7	365	81%	420	19%
8	365	96%	445	27%
9	365	98%	445	29%
10	365	75%	420	15%
11	365	69%	420	10%
12	365	93%	420	16%
13	365	90%	420	16%
14	365	88%	445	16%
15	365	79%	420	11%

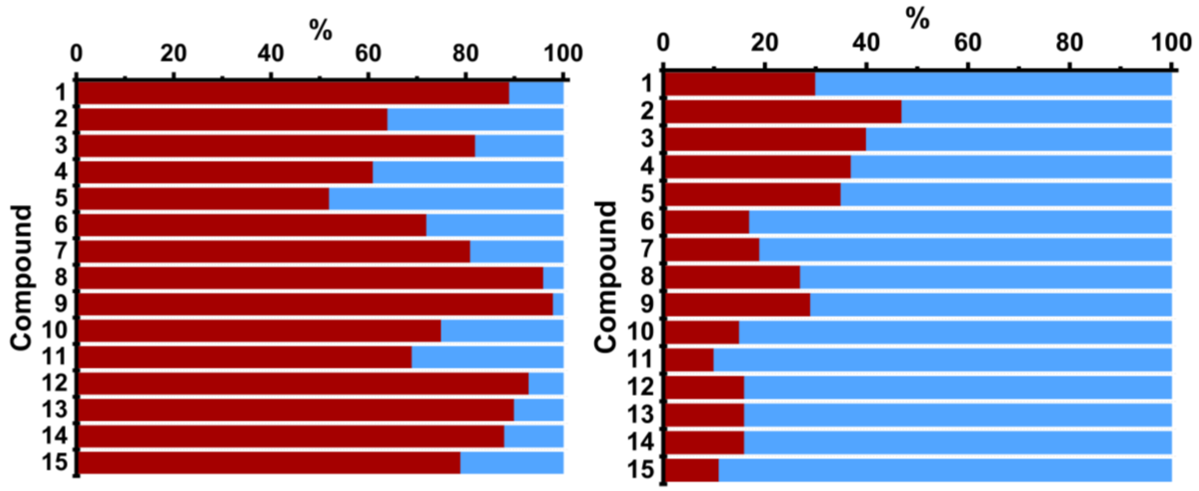

Figure S61. Overview of the PSDs in DMSO-d_6, upon irradiation (left) with light of λ_1 and (right) with light of λ_2.

S3. *In vitro* pharmacological characterization

S3.1. Protein and DNA sequences

>WT-DHFR-His(C85A, C152S)[6] (Protein sequence)

MAHHHHHHGSAMISLIAALAVDRVIGMENAMPWNLPADLAWFKRNTLDKPVIMGRHTWESIGRPLPG
RKNIILSSQPGTDDRVTWVKSVDEAIAAAGDVPEIMVIGGGRVYEQFLPKAQKLYLTHIDAEVEGDTHF
PDYEPDDWESVFSEFHDADAQNSHSYSFEILERR

>WT-DHFR-His(C85A, C152S)[6] (DNA sequence)

ATGGCTCACCACCACCACCACCACGGTTCGGCTATGATTTCTCTGATTGCGGCACTGGCTGTCG
ATCGTGTTATTGGTATGGAAAACGCTATGCCGTGGAATCTGCCGGCTGATCTGGCGTGGTTTAAA
CGTAACACCCTGGACAAGCCGGTCATTATGGGCCGCCATACGTGGGAAAGCATCGGTCGTCCG
CTGCCGGGTCGCAAAAATATTATCCTGAGCAGCCAGCCGGGCACCGATGACCGTGTGACGTGG
GTTAAGAGCGTCGATGAAGCAATTGCGGCGGCAGGCGACGTGCCGGAAATTATGGTTATCGGC
GGTGGCCGCGTTTATGAACAGTTCCTGCCGAAAGCCCAAAAGCTGTACCTGACCCATATCGATG
CAGAAGTCGAAGGTGATACGCACTTTCCGGACTATGAACCGGATGACTGGGAAAGTGTGTTCTC
CGAATTTCACGACGCCGACGCTCAGAACAGCCACTCATACTCATTCGAAATCCTGGAACGCCGT
TGATAAAAGCTT

S3.2. eDHFR inhibition assay

Table S3. Overview of IC_{50} values as determined with the DHFR assay. * = Significant differences in activity between the isomers (see Figures **S64-68**).

Compound		IC_{50}	Compound		IC_{50}
TMP		1.0 ± 0.2 nM	2,4-diaminopyrimidine		0.39 ± 0.15 mM
1	dark	> 10 µM	10*	dark*	24 ± 4.0 nM
	light	> 10 µM		light*	57 ± 12 nM
2	dark	> 10 µM	11	dark	58 ± 13 nM
	light	> 10 µM		light	70 ± 39 nM
3	dark	280 ± 60 nM	12*	dark*	13 ± 2.8 nM
	light	710 ± 560 nM		light*	71 ± 18 nM
4	dark	> 10 µM	13	dark	28 ± 4.8 nM
	light	> 10 µM		light	29 ± 8.6 nM
5	dark	5.3 ± 5.3 µM	14*	dark*	77 ± 26 nM
	light	2.6 ± 2.6 µM		light*	37 ± 14 nM
6*	dark*	370 ± 140 nM	15*	dark*	52 ± 11 nM
	light*	160 ± 62 nM		light*	27 ± 3.7 nM
7	dark	190 ± 40 nM	PMAT[7]	dark	2.0 ± 0.61 nM
	light	250 ± 140 nM		light	2.5 ± 0.61 nM
8	dark	330 ± 190 nM	TFAT[7]	dark	1.9 ± 0.61 nM
	light	200 ± 77 nM		light	1.7 ± 0.32 nM
9	dark	220 ± 60 nM	TCAT[7]	dark	3.8 ± 1.4 nM
	light	640 ± 510 nM		light	3.3 ± 2.1 nM

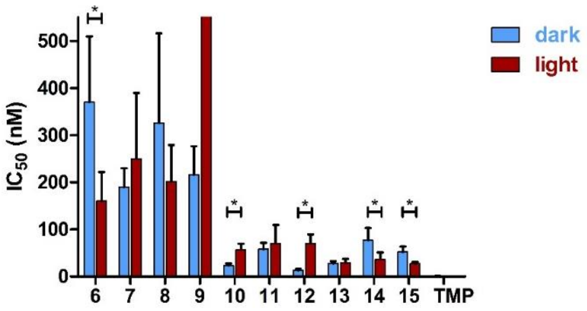

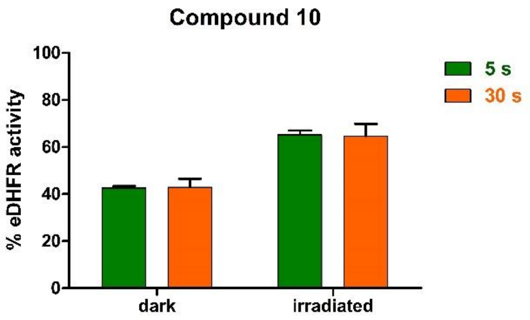

Figure S62. (left) Overview of IC_{50} values as determined with the eDHFR assay for compounds **6-15**. (right) Evaluation of possible assay interference: eDHFR inhibition of compound **10** at a fixed concentration (30 nM) with 5 s and 30 s recording time intervals.

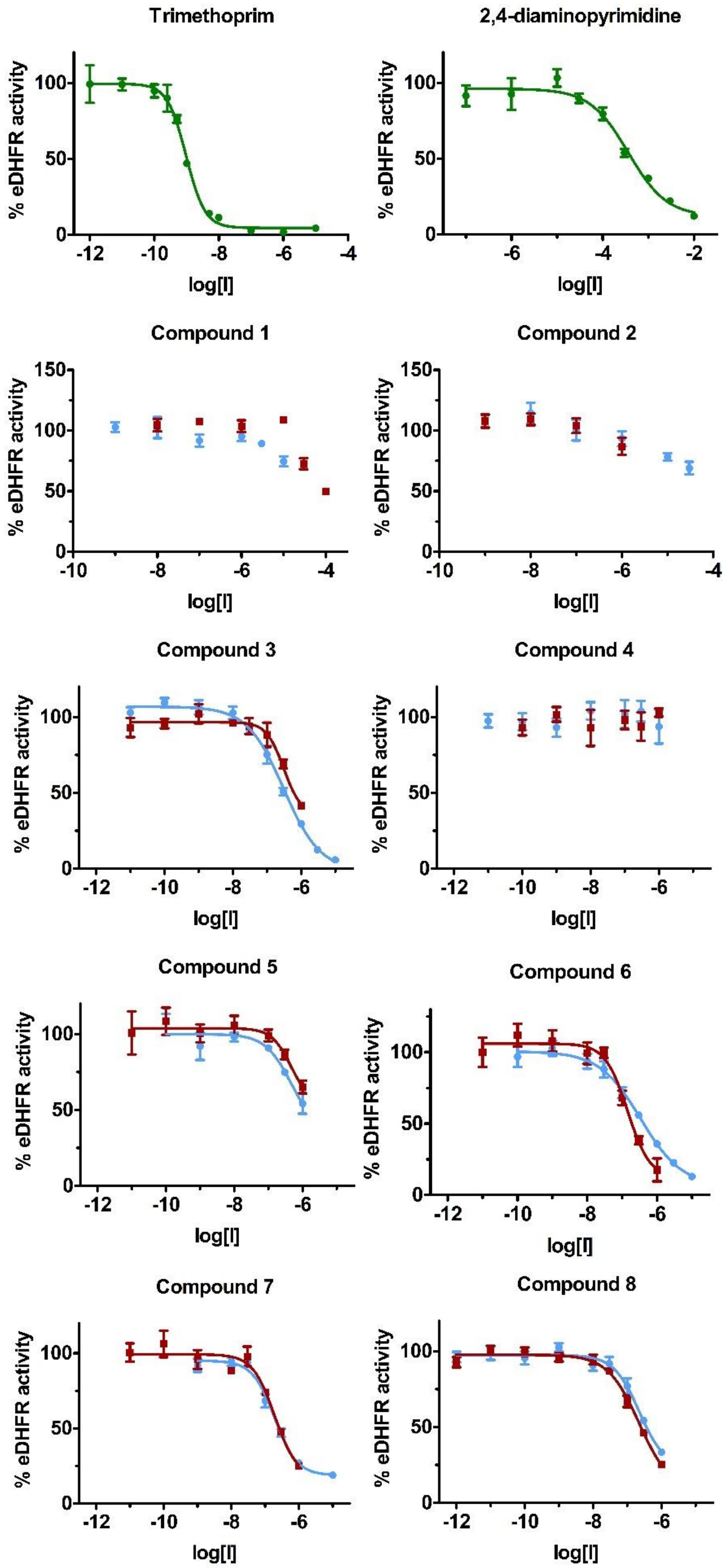
Trimethoprim
2,4-diaminopyrimidine
Compound 1
Compound 2
Compound 3
Compound 4
Compound 5
Compound 6
Compound 7
Compound 8
% eDHFR activity
log[I]
0
50
100
150
-12
-10
-8
-6
-4
-2

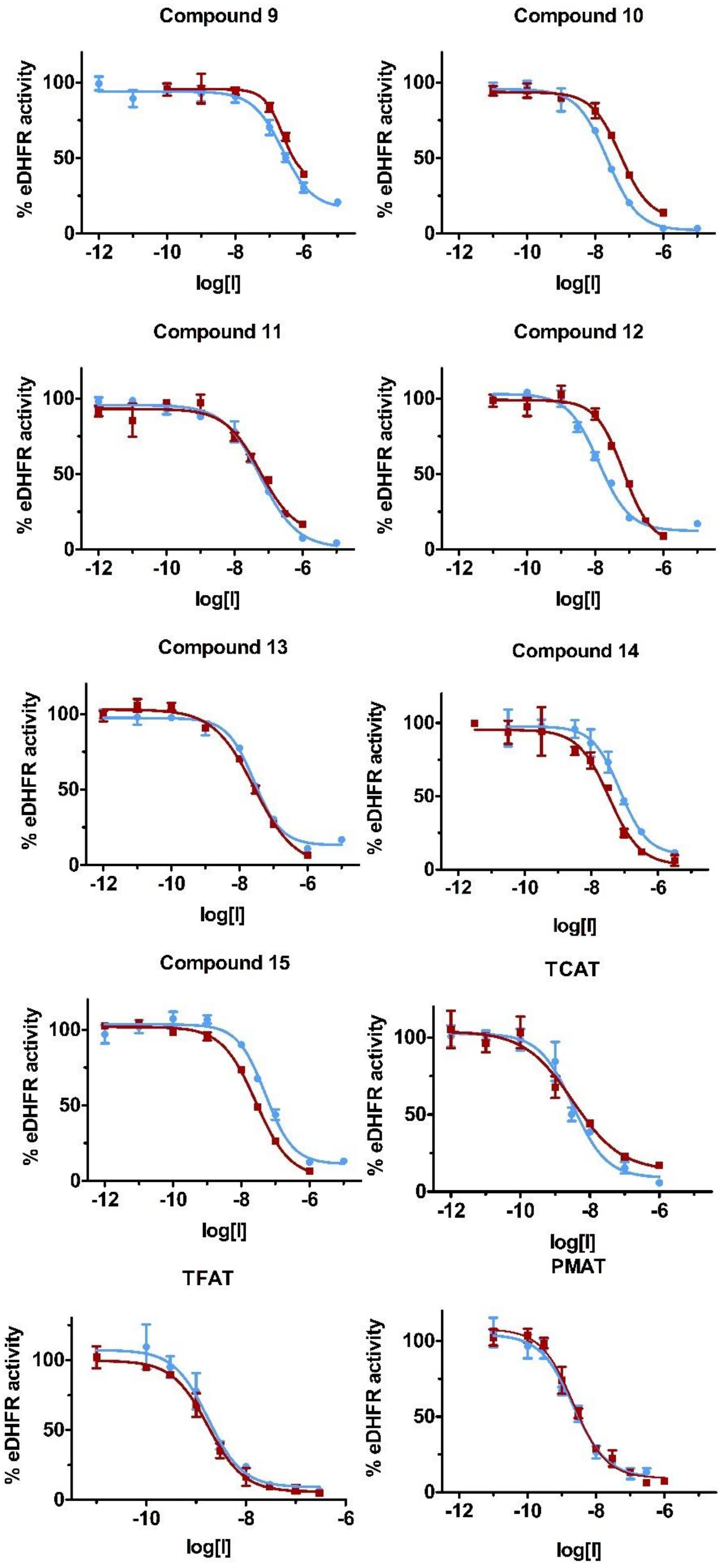

Figure S63. Dose-response curves for the tested eDHFR inhibitors.

	Nonlin fit	A dark Y	B irradiated Y	C Global (shared) Y
1	Comparison of Fits			
2	Null hypothesis			LOGIC50 same for all data sets
3	Alternative hypothesis			LOGIC50 different for each data set
4	P value			0.0126
5	Conclusion (alpha = 0.05)			Reject null hypothesis
6	Preferred model			LOGIC50 different for each data set
7	F (DFn, DFd)			6.777 (1,43)
8				
9	LOGIC50 different for each data set			
10	Best-fit values			
11	BOTTOM	6.194	11.76	
12	TOP	100.5	106.0	
13	LOGIC50	-6.469	-6.845	
14	HILLSLOPE	-0.7336	-1.352	
15	IC50	3.399e-007	1.428e-007	
16	Span	94.28	94.23	
17	Std. Error			
18	BOTTOM	4.703	8.068	
19	TOP	1.884	2.334	
20	LOGIC50	0.08291	0.08751	
21	HILLSLOPE	0.08541	0.2916	
22	Span	5.686	8.908	
23	95% Confidence Intervals			
24	BOTTOM	-3.538 to 15.92	-5.075 to 28.59	
25	TOP	96.57 to 104.4	101.1 to 110.9	
26	LOGIC50	-6.640 to -6.297	-7.028 to -6.663	
27	HILLSLOPE	-0.9103 to -0.5569	-1.960 to -0.7437	
28	IC50	2.290e-007 to 5.045e-007	9.382e-008 to 2.175e-007	
29	Span	82.51 to 106.0	75.65 to 112.8	
30	Goodness of Fit			
31	Degrees of Freedom	23	20	
32	R²	0.9851	0.9583	
33	Absolute Sum of Squares	420.2	1124	
34	Sy.x	4.274	7.495	
35				
36	LOGIC50 same for all data sets			
37	Best-fit values			
38	BOTTOM	12.01	-10.04	
39	TOP	101.1	106.8	
40	LOGIC50	-6.613	-6.613	-6.613
41	HILLSLOPE	-0.7998	-0.9510	
42	IC50	2.436e-007	2.436e-007	2.436e-007
43	Span	89.09	116.8	
44	Std. Error			
45	BOTTOM	4.969	9.919	
46	TOP	2.731	2.131	
47	LOGIC50	0.08816	0.08816	0.08816
48	HILLSLOPE	0.1311	0.1344	
49	Span	6.554	10.84	
50	95% Confidence Intervals			
51	BOTTOM	1.986 to 22.03	-30.05 to 9.964	
52	TOP	95.59 to 106.6	102.5 to 111.1	
53	LOGIC50	-6.791 to -6.436	-6.791 to -6.436	-6.791 to -6.436
54	HILLSLOPE	-1.064 to -0.5354	-1.222 to -0.6800	
55	IC50	1.617e-007 to 3.668e-007	1.617e-007 to 3.668e-007	1.617e-007 to 3.668e-007
56	Span	75.87 to 102.3	94.95 to 138.7	
57	Goodness of Fit			
58	Degrees of Freedom			44
59	R²	0.9827	0.9517	0.9693
60	Absolute Sum of Squares	486.2	1301	1787
61	Sy.x			6.373
62	Constraints			
63	LOGIC50	LOGIC50 is shared	LOGIC50 is shared	
64	Number of points			
65	Analyzed	27	24	

Figure S64. Results of the extra sum-of-squares F test for dark/irr **compound 6**.

Nonlin fit	A	B	C
	dark	irradiated	Global (shared)
	Y	Y	Y
1 Comparison of Fits			
2 Null hypothesis			LOGIC50 same for all data sets
3 Alternative hypothesis			LOGIC50 different for each data set
4 P value			P<0.0001
5 Conclusion (alpha = 0.05)			Reject null hypothesis
6 Preferred model			LOGIC50 different for each data set
7 F (DFn, DFd)			48.05 (1,37)
8			
9 LOGIC50 different for each data set			
10 Best-fit values			
11 BOTTOM	2.036	9.452	
12 TOP	95.64	93.63	
13 LOGIC50	-7.635	-7.253	
14 HILLSLOPE	-0.9674	-1.027	
15 IC50	2.319e-008	5.590e-008	
16 Span	93.60	84.18	
17 Std. Error			
18 BOTTOM	1.635	2.738	
19 TOP	1.338	1.092	
20 LOGIC50	0.03586	0.04525	
21 HILLSLOPE	0.07982	0.09728	
22 Span	2.261	3.148	
23 95% Confidence Intervals			
24 BOTTOM	-1.374 to 5.447	3.674 to 15.23	
25 TOP	92.85 to 98.43	91.33 to 95.93	
26 LOGIC50	-7.710 to -7.560	-7.348 to -7.157	
27 HILLSLOPE	-1.134 to -0.8009	-1.233 to -0.8221	
28 IC50	1.952e-008 to 2.755e-008	4.487e-008 to 6.964e-008	
29 Span	88.89 to 98.32	77.54 to 90.82	
30 Goodness of Fit			
31 Degrees of Freedom	20	17	
32 R²	0.9930	0.9911	
33 Absolute Sum of Squares	241.2	157.6	
34 Sy.x	3.472	3.045	
35			
36 LOGIC50 same for all data sets			
37 Best-fit values			
38 BOTTOM	-1.863	18.20	
39 TOP	93.72	95.39	
40 LOGIC50	-7.480	-7.480	-7.480
41 HILLSLOPE	-0.9011	-1.132	
42 IC50	3.309e-008	3.309e-008	3.309e-008
43 Span	95.58	77.18	
44 Std. Error			
45 BOTTOM	2.361	2.984	
46 TOP	1.842	1.721	
47 LOGIC50	0.04094	0.04094	0.04094
48 HILLSLOPE	0.1037	0.1642	
49 Span	3.371	3.848	
50 95% Confidence Intervals			
51 BOTTOM	-6.644 to 2.919	12.16 to 24.25	
52 TOP	89.99 to 97.45	91.90 to 98.87	
53 LOGIC50	-7.563 to -7.397	-7.563 to -7.397	-7.563 to -7.397
54 HILLSLOPE	-1.111 to -0.6911	-1.464 to -0.7992	
55 IC50	2.734e-008 to 4.005e-008	2.734e-008 to 4.005e-008	2.734e-008 to 4.005e-008
56 Span	88.76 to 102.4	69.39 to 84.98	
57 Goodness of Fit			
58 Degrees of Freedom			38
59 R²	0.9866	0.9743	0.9833
60 Absolute Sum of Squares	459.9	456.8	916.7
61 Sy.x			4.912
62 Constraints			
63 LOGIC50	LOGIC50 is shared	LOGIC50 is shared	
64 Number of points			
65 Analyzed	24	21	

Figure S65. Results of the extra sum-of-squares F test for dark/irr **compound 10**.

	Nonlin fit	A	B	C
		dark	irradiated	Global (shared)
		Y	Y	Y
1	Comparison of Fits			
2	Null hypothesis			LOGIC50 same for all data sets
3	Alternative hypothesis			LOGIC50 different for each data set
4	P value			P<0.0001
5	Conclusion (alpha = 0.05)			Reject null hypothesis
6	Preferred model			LOGIC50 different for each data set
7	F (DFn, DFd)			111.6 (1,43)
8				
9	LOGIC50 different for each data set			
10	Best-fit values			
11	BOTTOM	12.29	3.863	
12	TOP	103.1	99.02	
13	LOGIC50	-7.897	-7.167	
14	HILLSLOPE	-0.9554	-1.075	
15	IC50	1.269e-008	6.810e-008	
16	Span	90.80	95.16	
17	Std. Error			
18	BOTTOM	1.892	4.012	
19	TOP	1.803	1.508	
20	LOGIC50	0.04669	0.05583	
21	HILLSLOPE	0.08630	0.1195	
22	Span	2.808	4.578	
23	95% Confidence Intervals			
24	BOTTOM	8.372 to 16.20	-4.505 to 12.23	
25	TOP	99.35 to 106.8	95.87 to 102.2	
26	LOGIC50	-7.993 to -7.800	-7.283 to -7.050	
27	HILLSLOPE	-1.134 to -0.7768	-1.324 to -0.8260	
28	IC50	1.016e-008 to 1.585e-008	5.208e-008 to 8.905e-008	
29	Span	84.99 to 96.61	85.61 to 104.7	
30	Goodness of Fit			
31	Degrees of Freedom	23	20	
32	R²	0.9877	0.9872	
33	Absolute Sum of Squares	436.5	377.7	
34	Sy.x	4.356	4.345	
35				
36	LOGIC50 same for all data sets			
37	Best-fit values			
38	BOTTOM	3.498	17.56	
39	TOP	97.96	102.1	
40	LOGIC50	-7.509	-7.509	-7.509
41	HILLSLOPE	-0.8029	-1.228	
42	IC50	3.096e-008	3.096e-008	3.096e-008
43	Span	94.46	84.58	
44	Std. Error			
45	BOTTOM	3.963	4.396	
46	TOP	3.234	2.817	
47	LOGIC50	0.06511	0.06511	0.06511
48	HILLSLOPE	0.1422	0.2658	
49	Span	6.025	5.781	
50	95% Confidence Intervals			
51	BOTTOM	-4.495 to 11.49	8.694 to 26.43	
52	TOP	91.44 to 104.5	96.46 to 107.8	
53	LOGIC50	-7.641 to -7.378	-7.641 to -7.378	-7.641 to -7.378
54	HILLSLOPE	-1.090 to -0.5161	-1.764 to -0.6920	
55	IC50	2.288e-008 to 4.189e-008	2.288e-008 to 4.189e-008	2.288e-008 to 4.189e-008
56	Span	82.31 to 106.6	72.92 to 96.24	
57	Goodness of Fit			
58	Degrees of Freedom			44
59	R²	0.9538	0.9562	0.9552
60	Absolute Sum of Squares	1637	1290	2928
61	Sy.x			8.157
62	Constraints			
63	LOGIC50	LOGIC50 is shared	LOGIC50 is shared	
64	Number of points			
65	Analyzed	27	24	

Figure S66. Results of the extra sum-of-squares F test for dark/irr **compound 12**.

	Nonlin fit	A	B	C
		dark	irradiated	Global (shared)
		Y	Y	Y
1	Comparison of Fits			
2	Null hypothesis			LOGIC50 same for all data sets
3	Alternative hypothesis			LOGIC50 different for each data set
4	P value			0.0039
5	Conclusion (alpha = 0.05)			Reject null hypothesis
6	Preferred model			LOGIC50 different for each data set
7	F (DFn, DFd)			9.308 (1,43)
8				
9	LOGIC50 different for each data set			
10	Best-fit values			
11	BOTTOM	10.09	3.118	
12	TOP	97.67	95.39	
13	LOGIC50	-7.138	-7.468	
14	HILLSLOPE	-1.048	-0.9319	
15	IC50	7.270e-008	3.404e-008	
16	Span	87.58	92.27	
17	Std. Error			
18	BOTTOM	4.160	4.286	
19	TOP	2.304	2.383	
20	LOGIC50	0.07229	0.08142	
21	HILLSLOPE	0.1589	0.1381	
22	Span	5.145	5.251	
23	95% Confidence Intervals			
24	BOTTOM	1.413 to 18.77	-5.750 to 11.99	
25	TOP	92.86 to 102.5	90.45 to 100.3	
26	LOGIC50	-7.289 to -6.988	-7.636 to -7.300	
27	HILLSLOPE	-1.379 to -0.7164	-1.217 to -0.6462	
28	IC50	5.137e-008 to 1.029e-007	2.310e-008 to 5.017e-008	
29	Span	76.85 to 98.31	81.40 to 103.1	
30	Goodness of Fit			
31	Degrees of Freedom	20	23	
32	R²	0.9704	0.9676	
33	Absolute Sum of Squares	752.9	1093	
34	Sy.x	6.136	6.894	
35				
36	LOGIC50 same for all data sets			
37	Best-fit values			
38	BOTTOM	15.18	-3.797	
39	TOP	99.44	94.34	
40	LOGIC50	-7.293	-7.293	-7.293
41	HILLSLOPE	-1.083	-0.8196	
42	IC50	5.096e-008	5.096e-008	5.096e-008
43	Span	84.26	98.14	
44	Std. Error			
45	BOTTOM	4.021	4.503	
46	TOP	2.694	2.487	
47	LOGIC50	0.06256	0.06256	0.06256
48	HILLSLOPE	0.1912	0.1201	
49	Span	5.477	5.894	
50	95% Confidence Intervals			
51	BOTTOM	7.072 to 23.29	-12.88 to 5.285	
52	TOP	94.01 to 104.9	89.33 to 99.36	
53	LOGIC50	-7.419 to -7.167	-7.419 to -7.167	-7.419 to -7.167
54	HILLSLOPE	-1.468 to -0.6971	-1.062 to -0.5774	
55	IC50	3.811e-008 to 6.815e-008	3.811e-008 to 6.815e-008	3.811e-008 to 6.815e-008
56	Span	73.21 to 95.30	86.25 to 110.0	
57	Goodness of Fit			
58	Degrees of Freedom			44
59	R²	0.9634	0.9610	0.9624
60	Absolute Sum of Squares	931.7	1314	2246
61	Sy.x			7.144
62	Constraints			
63	LOGIC50	LOGIC50 is shared	LOGIC50 is shared	
64	Number of points			
65	Analyzed	24	27	

Figure S67. Results of the extra sum-of-squares F test for dark/irr **compound 14**.

Nonlin fit	A<br>dark<br>Y	B<br>irradiated<br>Y	C<br>Global (shared)<br>Y
1 Comparison of Fits			
2 Null hypothesis			LOGIC50 same for all data sets
3 Alternative hypothesis			LOGIC50 different for each data set
4 P value			0.0003
5 Conclusion (alpha = 0.05)			Reject null hypothesis
6 Preferred model			LOGIC50 different for each data set
7 F (DFn, DFd)			15.10 (1,43)
8			
9 LOGIC50 different for each data set			
10 Best-fit values			
11 BOTTOM	11.24	2.324	
12 TOP	103.6	101.9	
13 LOGIC50	-7.293	-7.571	
14 HILLSLOPE	-1.065	-0.8722	
15 IC50	5.093e-008	2.685e-008	
16 Span	92.33	99.60	
17 Std. Error			
18 BOTTOM	2.299	1.872	
19 TOP	1.408	0.7299	
20 LOGIC50	0.04645	0.02895	
21 HILLSLOPE	0.1156	0.04933	
22 Span	2.822	2.151	
23 95% Confidence Intervals			
24 BOTTOM	6.479 to 15.99	-1.580 to 6.228	
25 TOP	100.7 to 106.5	100.4 to 103.5	
26 LOGIC50	-7.389 to -7.197	-7.632 to -7.511	
27 HILLSLOPE	-1.304 to -0.8256	-0.9751 to -0.7693	
28 IC50	4.082e-008 to 6.355e-008	2.336e-008 to 3.085e-008	
29 Span	86.49 to 98.17	95.12 to 104.1	
30 Goodness of Fit			
31 Degrees of Freedom	23	20	
32 R²	0.9863	0.9968	
33 Absolute Sum of Squares	503.1	96.36	
34 Sy.x	4.677	2.195	
35			
36 LOGIC50 same for all data sets			
37 Best-fit values			
38 BOTTOM	12.71	-11.42	
39 TOP	104.1	102.6	
40 LOGIC50	-7.348	-7.348	-7.348
41 HILLSLOPE	-1.091	-0.6509	
42 IC50	4.489e-008	4.489e-008	4.489e-008
43 Span	91.37	114.0	
44 Std. Error			
45 BOTTOM	1.999	3.632	
46 TOP	1.283	1.600	
47 LOGIC50	0.03819	0.03819	0.03819
48 HILLSLOPE	0.1074	0.05904	
49 Span	2.507	4.642	
50 95% Confidence Intervals			
51 BOTTOM	8.677 to 16.74	-18.74 to -4.090	
52 TOP	101.5 to 106.7	99.36 to 105.8	
53 LOGIC50	-7.425 to -7.271	-7.425 to -7.271	-7.425 to -7.271
54 HILLSLOPE	-1.308 to -0.8745	-0.7699 to -0.5318	
55 IC50	3.760e-008 to 5.361e-008	3.760e-008 to 5.361e-008	3.760e-008 to 5.361e-008
56 Span	86.31 to 96.42	104.6 to 123.4	
57 Goodness of Fit			
58 Degrees of Freedom			44
59 R²	0.9854	0.9909	0.9879
60 Absolute Sum of Squares	533.9	276.0	809.9
61 Sy.x			4.290
62 Constraints			
63 LOGIC50	LOGIC50 is shared	LOGIC50 is shared	
64 Number of points			
65 Analyzed	27	24	

Figure S68. Results of the extra sum-of-squares F test for dark/irr **compound 15**.

S4. Antibacterial assay and bacterial growth curves

Table S4. Overview of MIC values as determined with the antibacterial activity assay. * = The concentration range was limited by solubility in LB medium.

Compound		MIC (µM)
TMP		1.3
6	dark	> 40*
	light	20
7	dark	20*
	light	20*
8	dark	> 80*
	light	> 80*
9	dark	80
	light	80
10	dark	20
	light	20
11	dark	40
	light	40
12	dark	20
	light	20
13	dark	40
	light	80
14	dark	20
	light	40
15	dark	40
	light	20

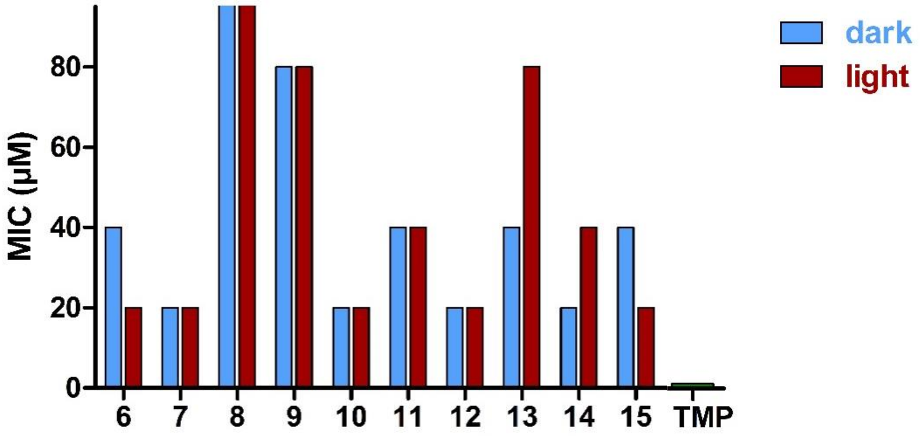

Figure S69. Overview of MIC values as determined with antibacterial activity assay for compounds **6**-**15**.

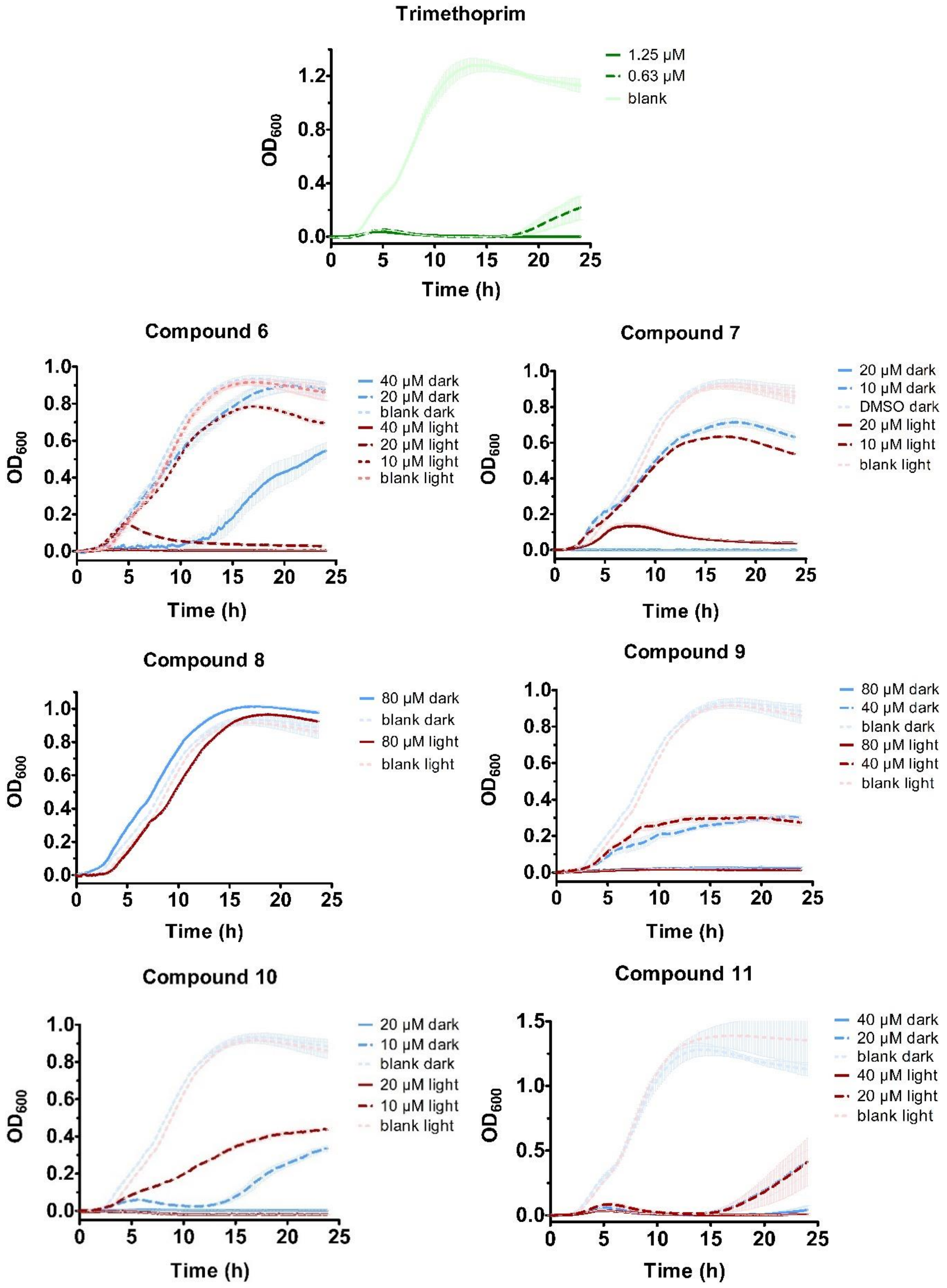
Trimethoprim
1.25 µM
0.63 µM
blank
OD_{600}
Time (h)
Compound 6
40 µM dark
20 µM dark
blank dark
40 µM light
20 µM light
10 µM light
blank light
Compound 7
20 µM dark
10 µM dark
DMSO dark
20 µM light
10 µM light
blank light
Compound 8
80 µM dark
blank dark
80 µM light
blank light
Compound 9
80 µM dark
40 µM dark
blank dark
80 µM light
40 µM light
blank light
Compound 10
20 µM dark
10 µM dark
blank dark
20 µM light
10 µM light
blank light
Compound 11
40 µM dark
20 µM dark
blank dark
40 µM light
20 µM light
blank light

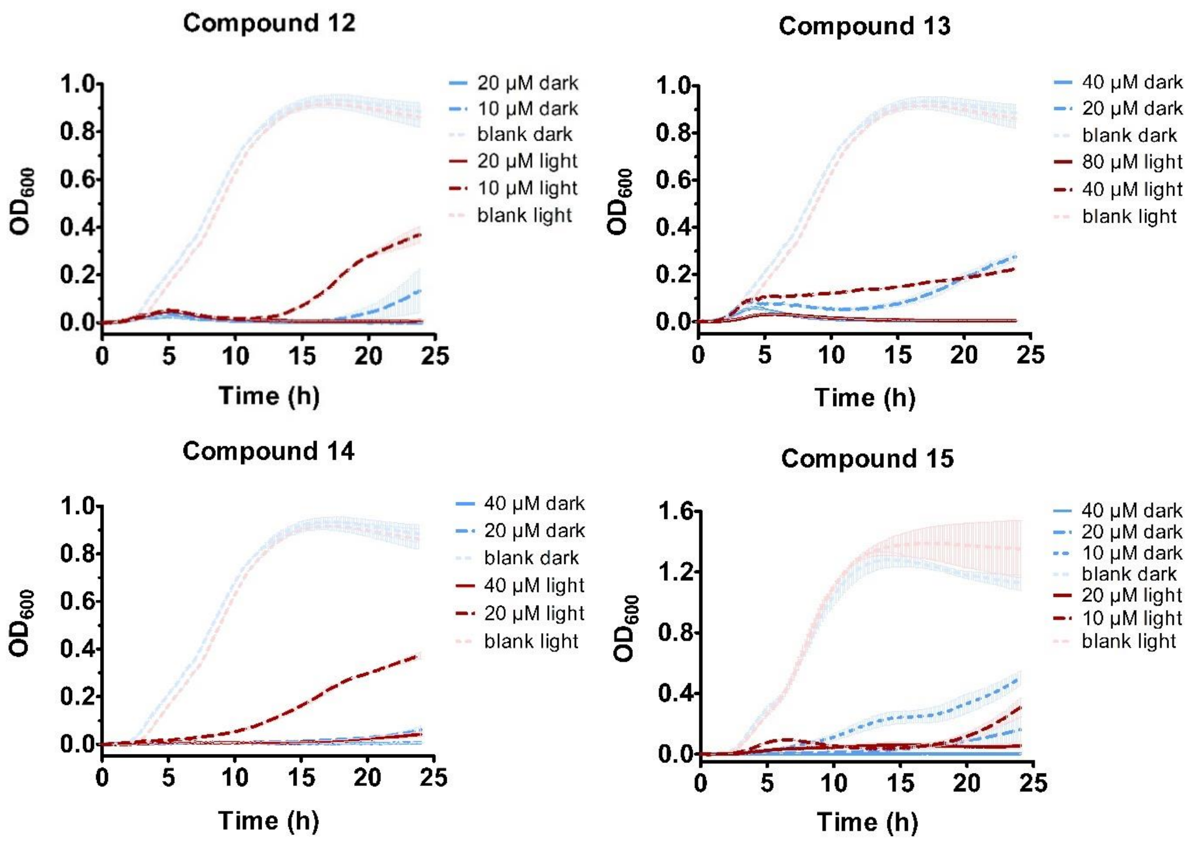

Figure S70. Bacterial growth curves for compounds **6**-**15**.

S5. Molecular modeling

S5.1. Geometry measurements from the CSD

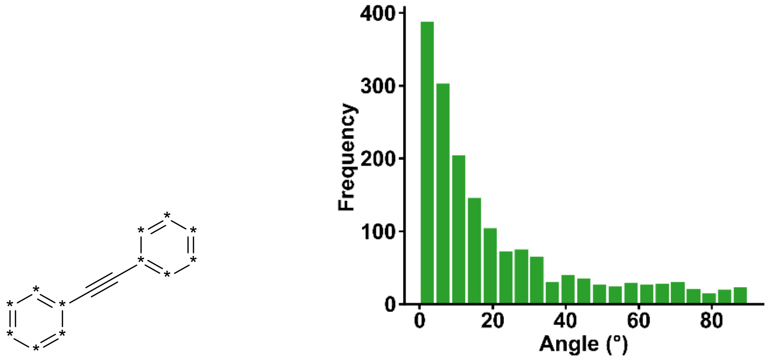

Figure S71. Distribution of ring angles of two generic 6-membered rings linked by a triple bond in the CSD (1706 hits).

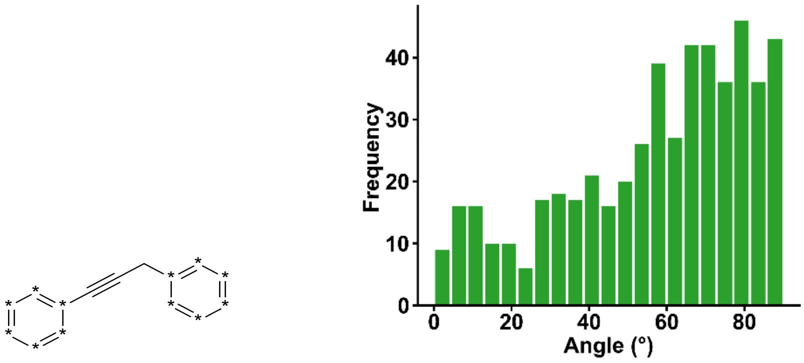

Figure S72. Distribution of ring angles of two generic 6-membered rings linked by a propargyl linker in the CSD (513 hits).

S5.2. Molecular docking and MD simulations

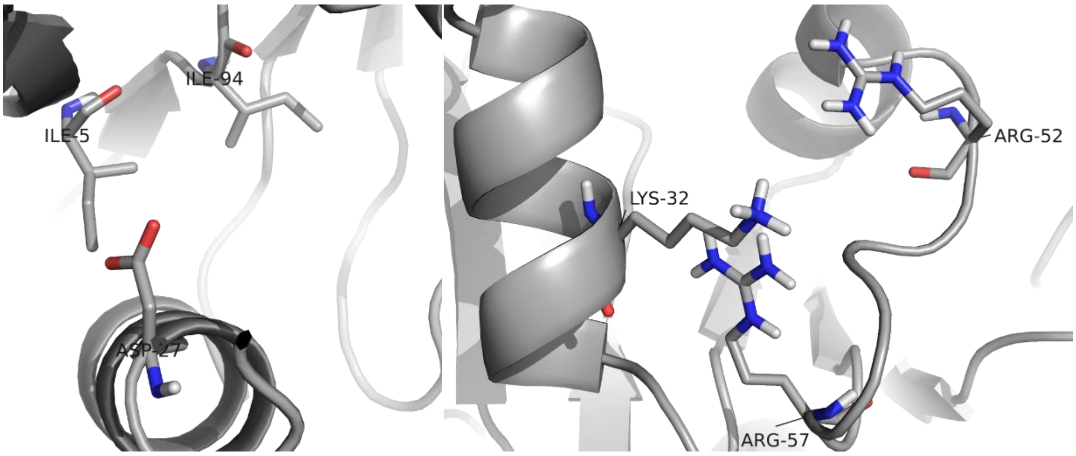

Figure S73. Key residues for buried hydrogen bonds (left, Asp27, Ile5, Ile94) and for salt bridges (right, Arg57, Arg52, Lys32).

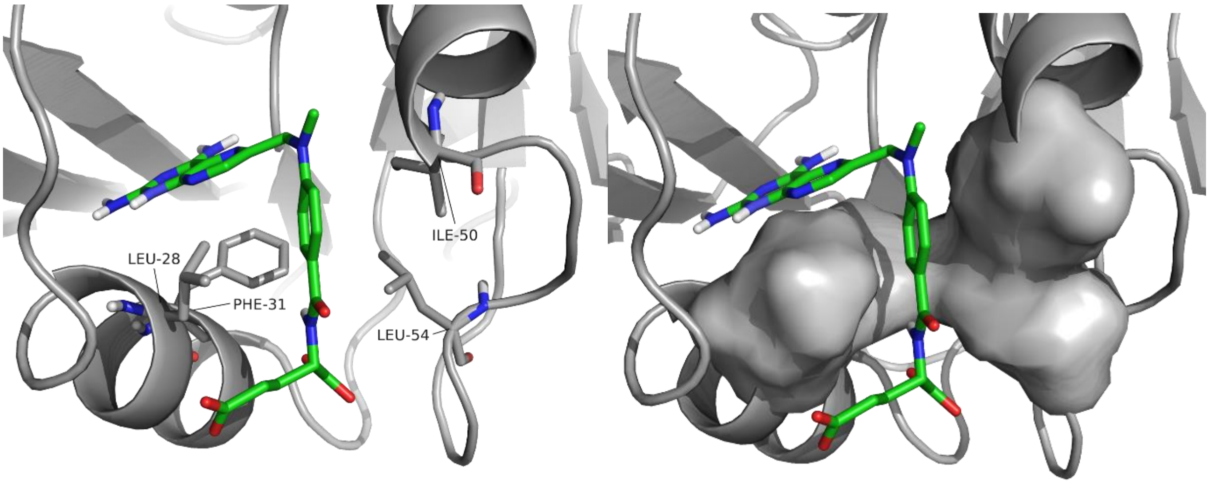

Figure S74. Residues of the lipophilic pocket (Phe31, Leu28, Leu54, Ile50).

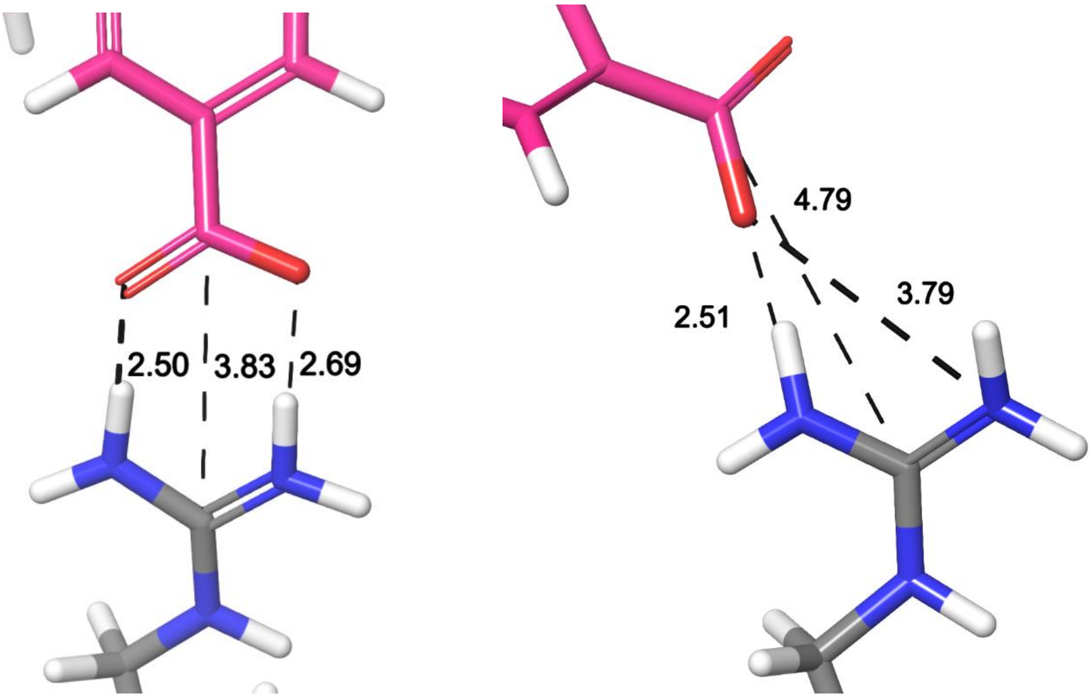

Figure S75. Spatial arrangement and distance measurements for (left) a standard salt bridge and (right) an N-O bridge.

S5.2.1. Reference compounds

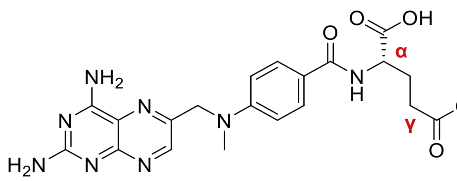

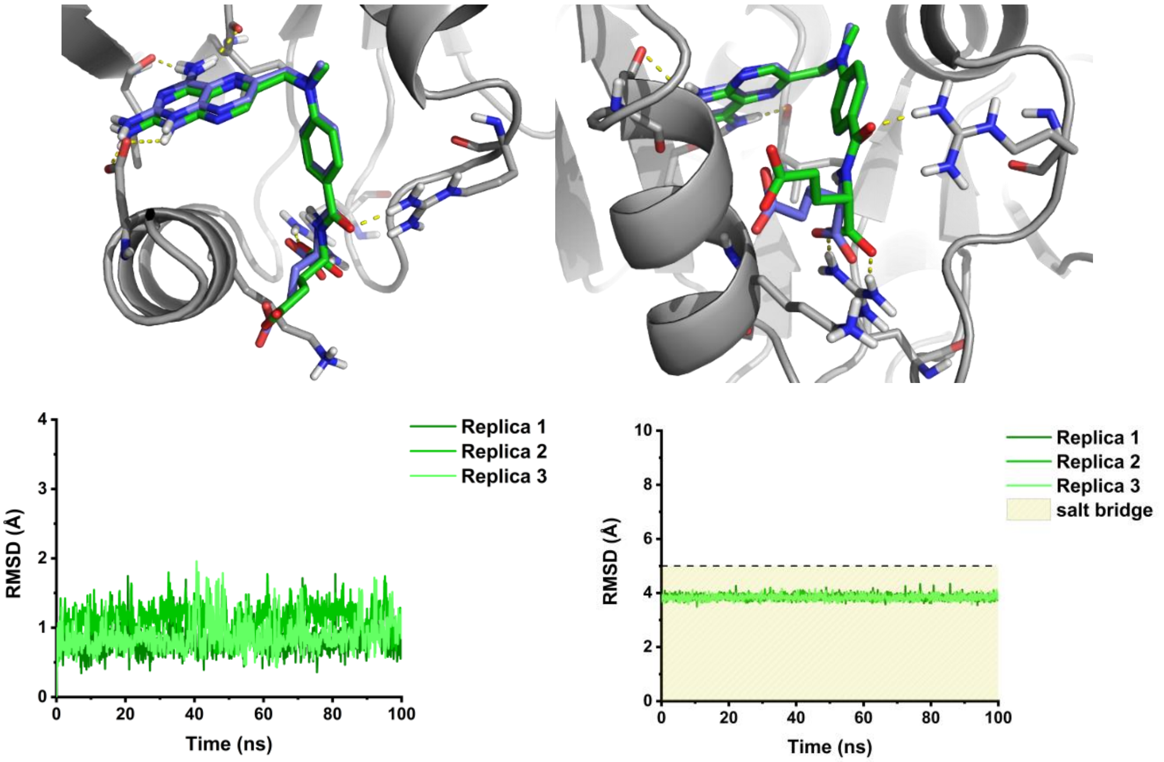

Figure S76. (top) Redocking of the co-crystallized ligand methotrexate. The co-crystallized ligand is depicted in green, while the docking pose is in purple. (bottom left) RMSD of methotrexate throughout three replicas of 100 ns MD simulations. (bottom right) Distance between Arg57 and carboxylates of methotrexate throughout three replicas of 100 ns MD simulations.

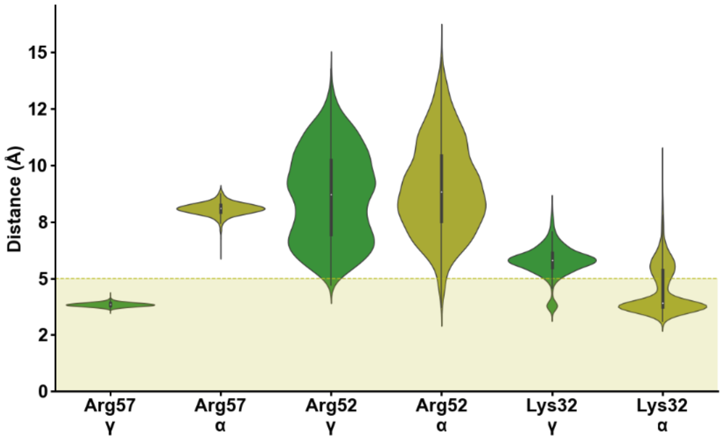

Figure S77. Violin plots of the distance between γ- and α-carboxylate of methotrexate and selected residues throughout three replicas of 100 ns MD simulations.

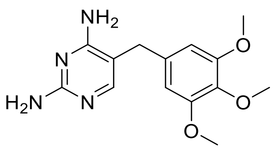

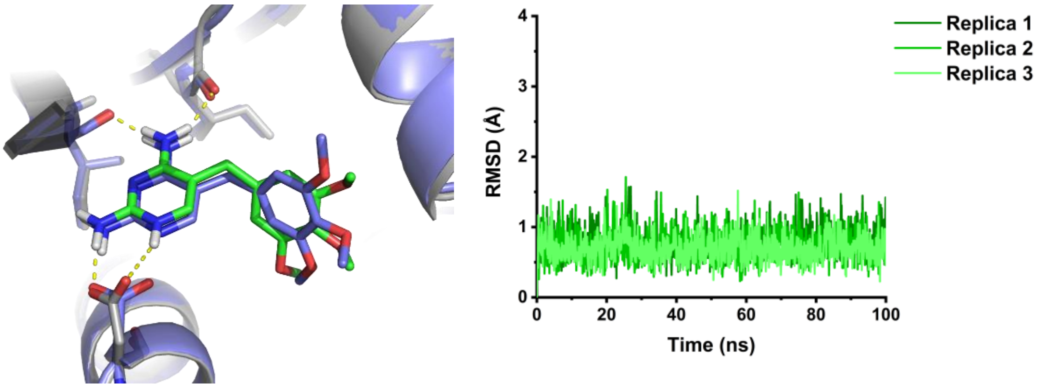

Figure S78. (left) Docking pose of TMP (green), superposed with the complex TMP-eDHFR (violet, PDB ID: 6XG5). (right) RMSD of TMP throughout three replicas of 100 ns MD simulations.

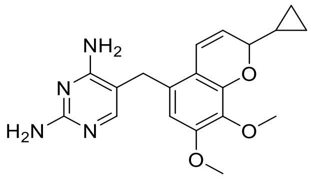

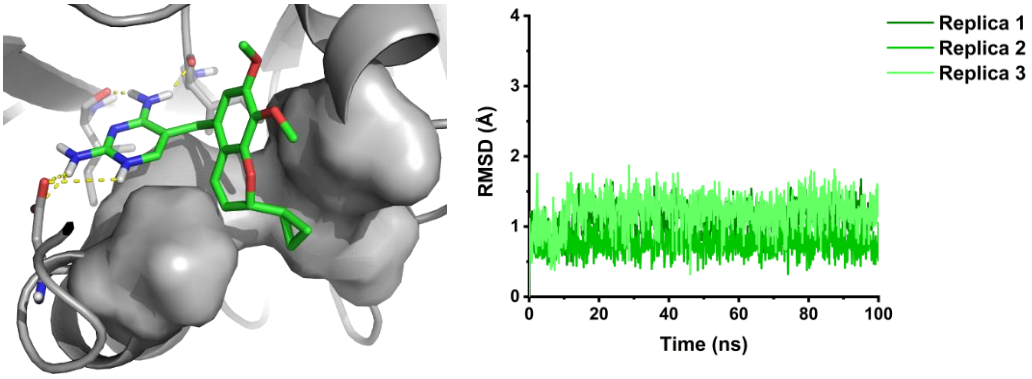

Figure S79. (left) Docking pose of iclaprim (green). (right) RMSD of iclaprim throughout three replicas of 100 ns MD simulations.

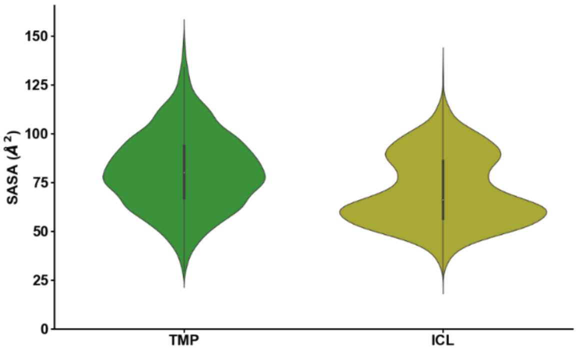

Figure S80. Violin plots of the SASA of the lipophilic pocket for TMP and iclaprim throughout three replicas of 100 ns MD simulations.

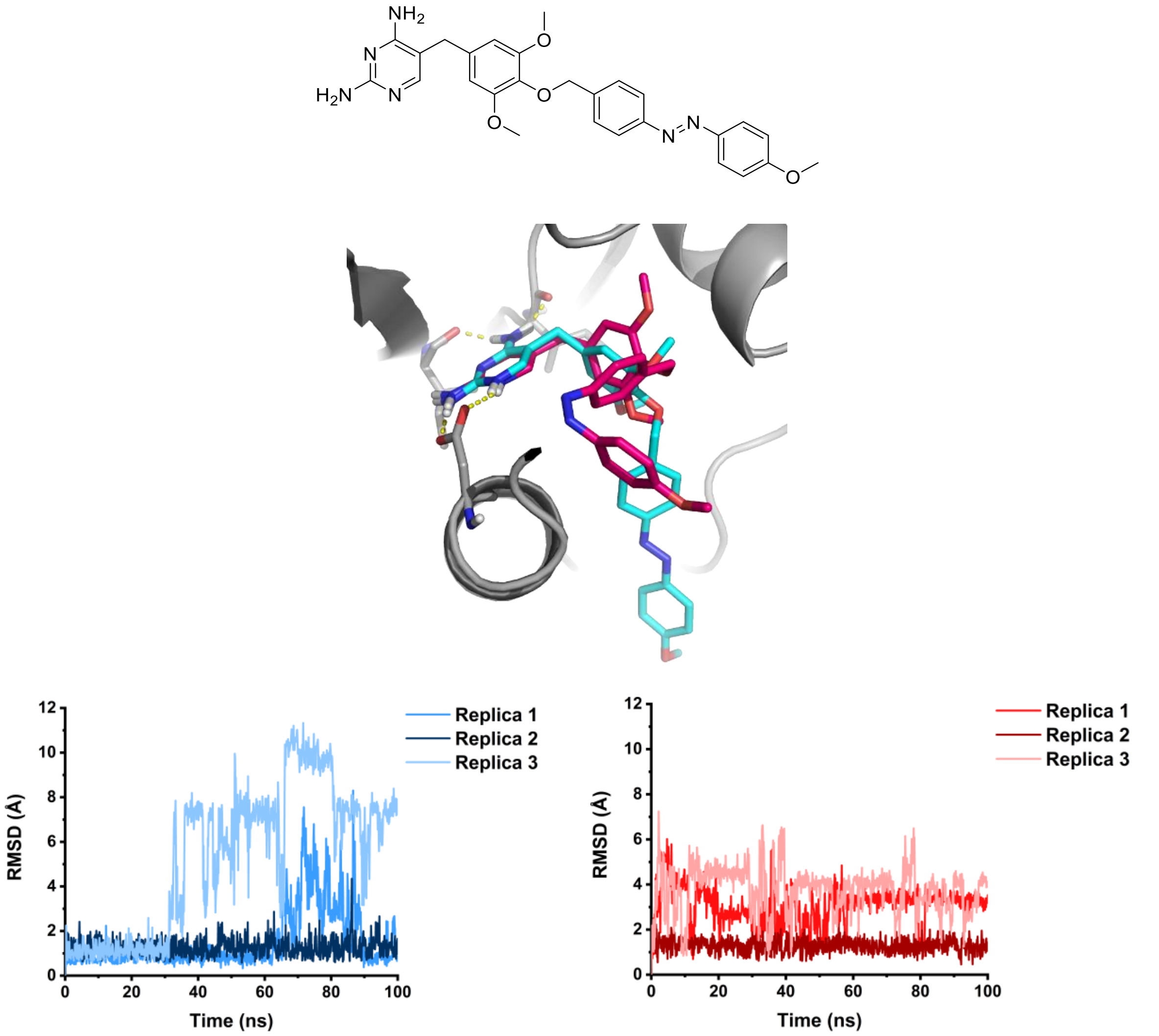

Figure S81. (top) Docking poses of *trans*-**PMAT** (cyan) and *cis*-**PMAT** (red). (bottom) RMSD of (left) *trans*-**PMAT** and (right) *cis*-**PMAT** throughout three replicas of 100 ns MD simulations.

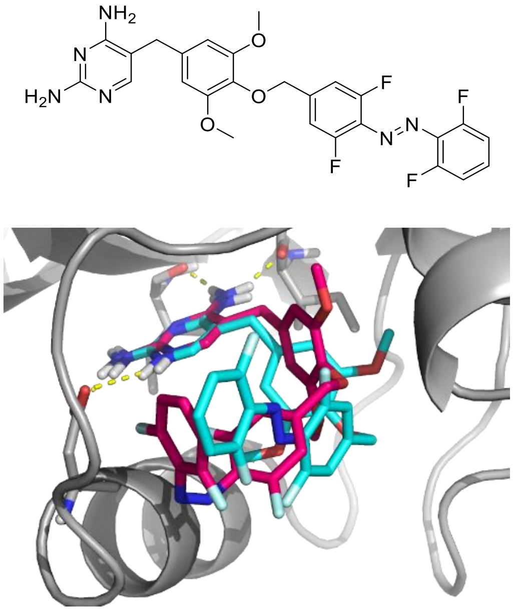

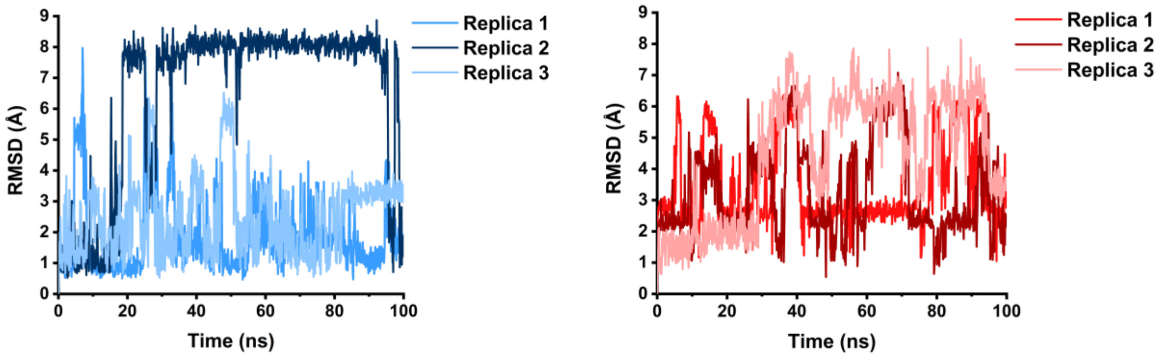

Figure S82. (top) Docking poses of *trans*-**TFAT** (cyan) and *cis*-**TFAT** (red). (bottom) RMSD of (left) *trans*-**TFAT** and (right) *cis*-**TFAT** throughout three replicas of 100 ns MD simulations.

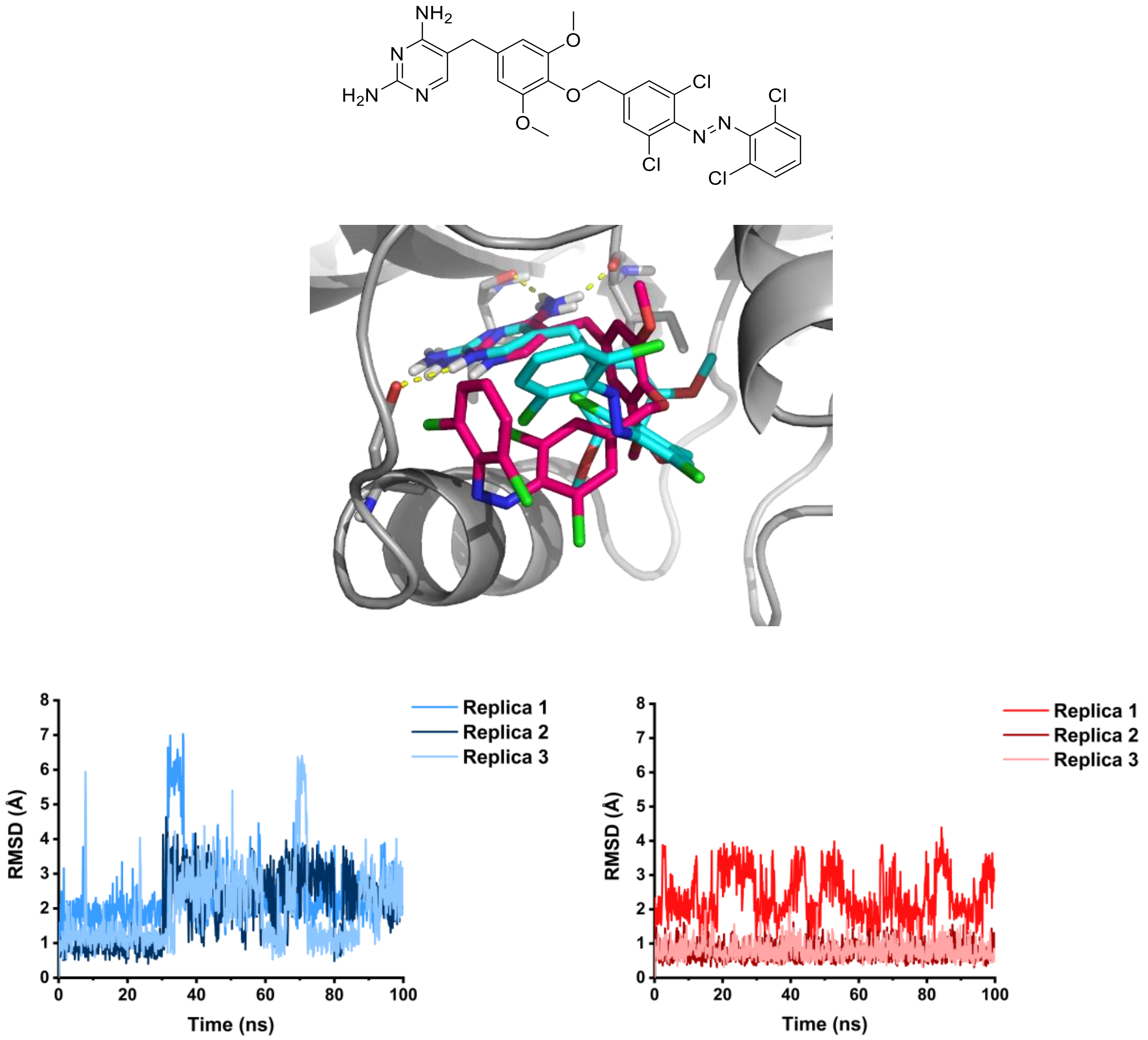

Figure S83. (top) Docking poses of *trans*-**TCAT** (cyan) and *cis*-**TCAT** (red). (bottom) RMSD of (left) *trans*-**TCAT** and (right) *cis*-**TCAT** throughout three replicas of 100 ns MD simulations.

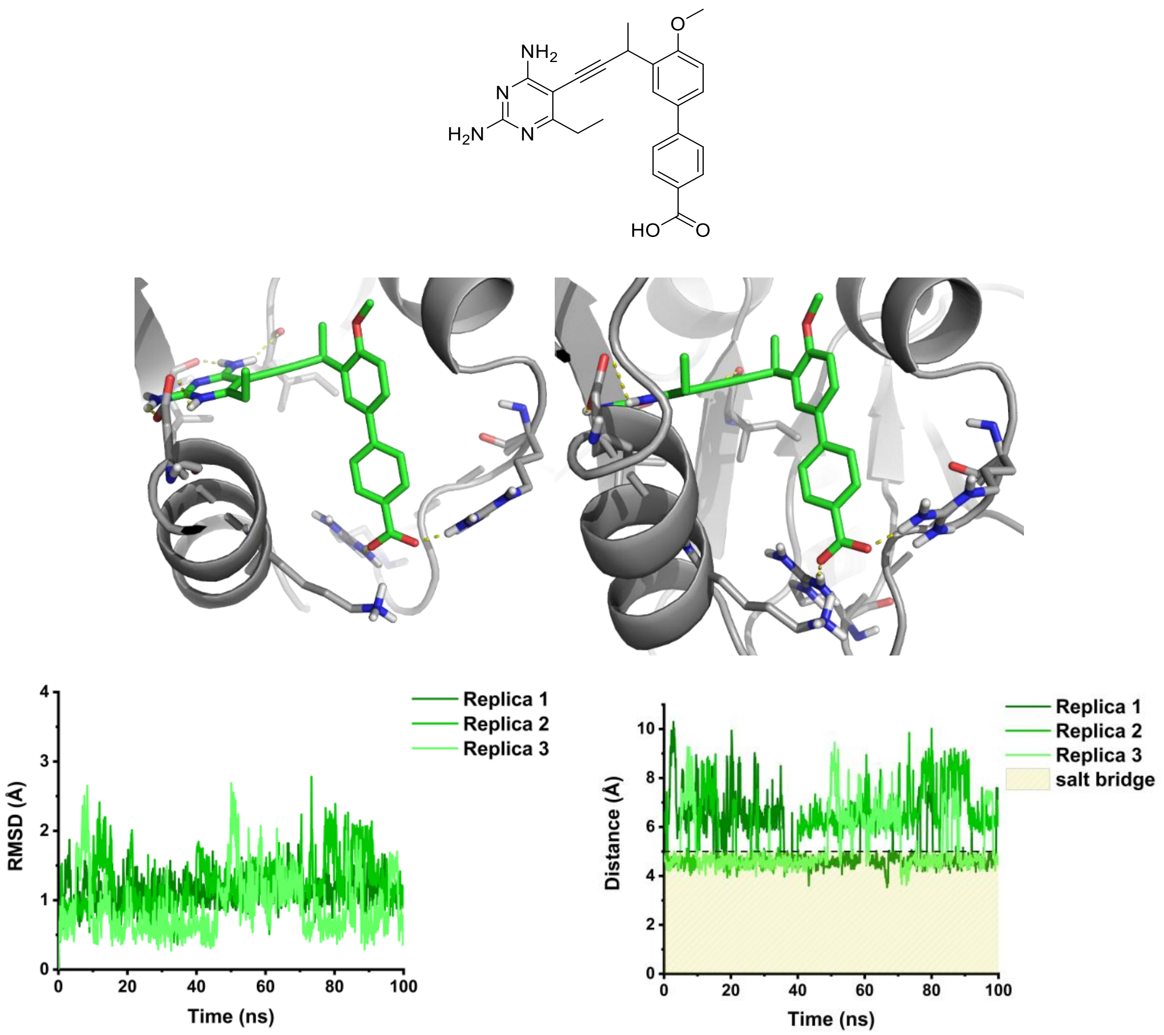

Figure S84. (top) Docking pose of **PLA-COOH** (green). (bottom left) RMSD of **PLA-COOH** throughout three replicas of 100 ns MD simulations. (bottom right) Distance between Arg57 and the carboxylic acid of **PLA-COOH** throughout three replicas of 100 ns MD simulations.

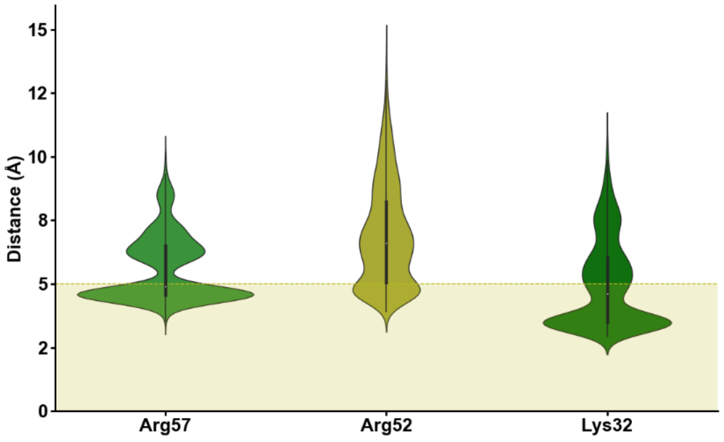

Figure S85. Violin plots of the distance between the carboxylic acid of **PLA-COOH** and selected residues throughout three replicas of 100 ns MD simulations.

S5.2.2. First hypothesis

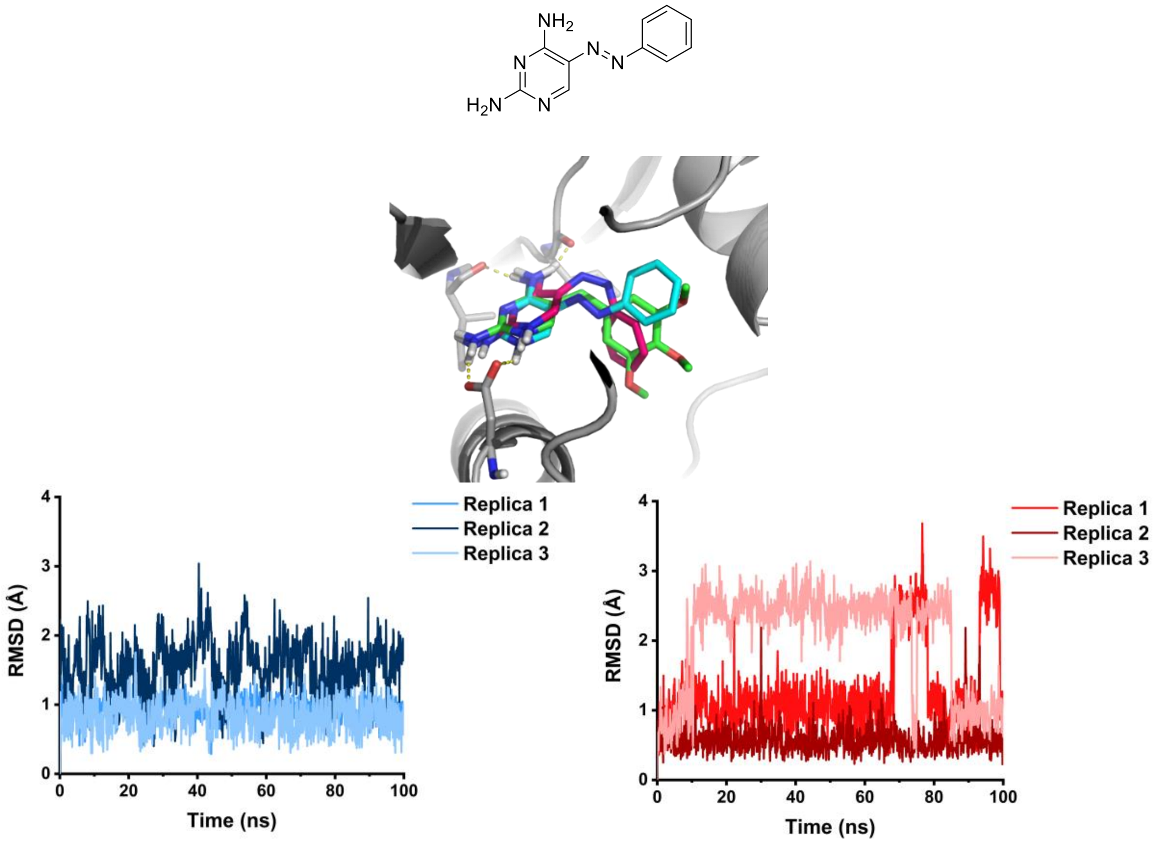

Figure S86. (top) Docking poses of *trans*-**1** (cyan) and *cis*-**1** (red), superimposed with the docking pose of TMP (green). (bottom) RMSD of (left) *trans*-**1** and (right) *cis*-**1** throughout three replicas of 100 ns MD simulations.

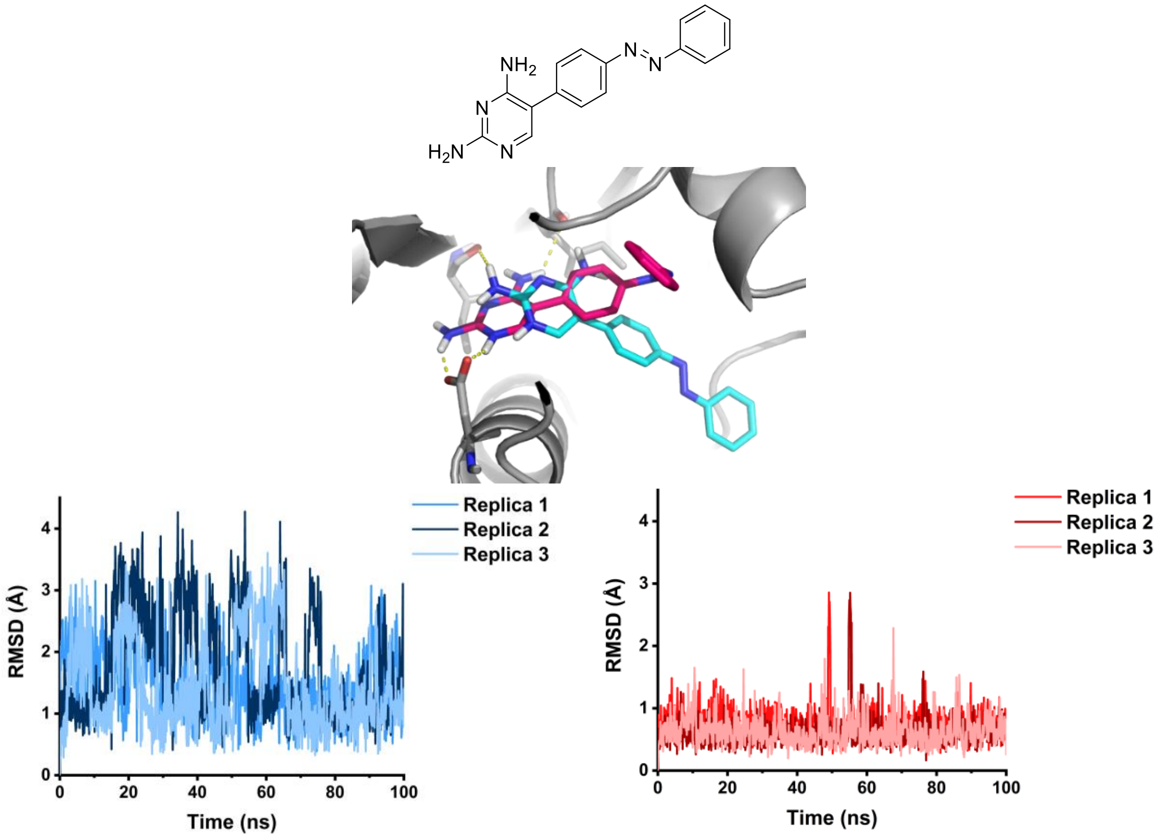

Figure S87. (top) Docking poses of *trans*-**2** (cyan) and *cis*-**2** (red). (bottom) RMSD of (left) *trans*-**2** and (right) *cis*-**2** throughout three replicas of 100 ns MD simulations.

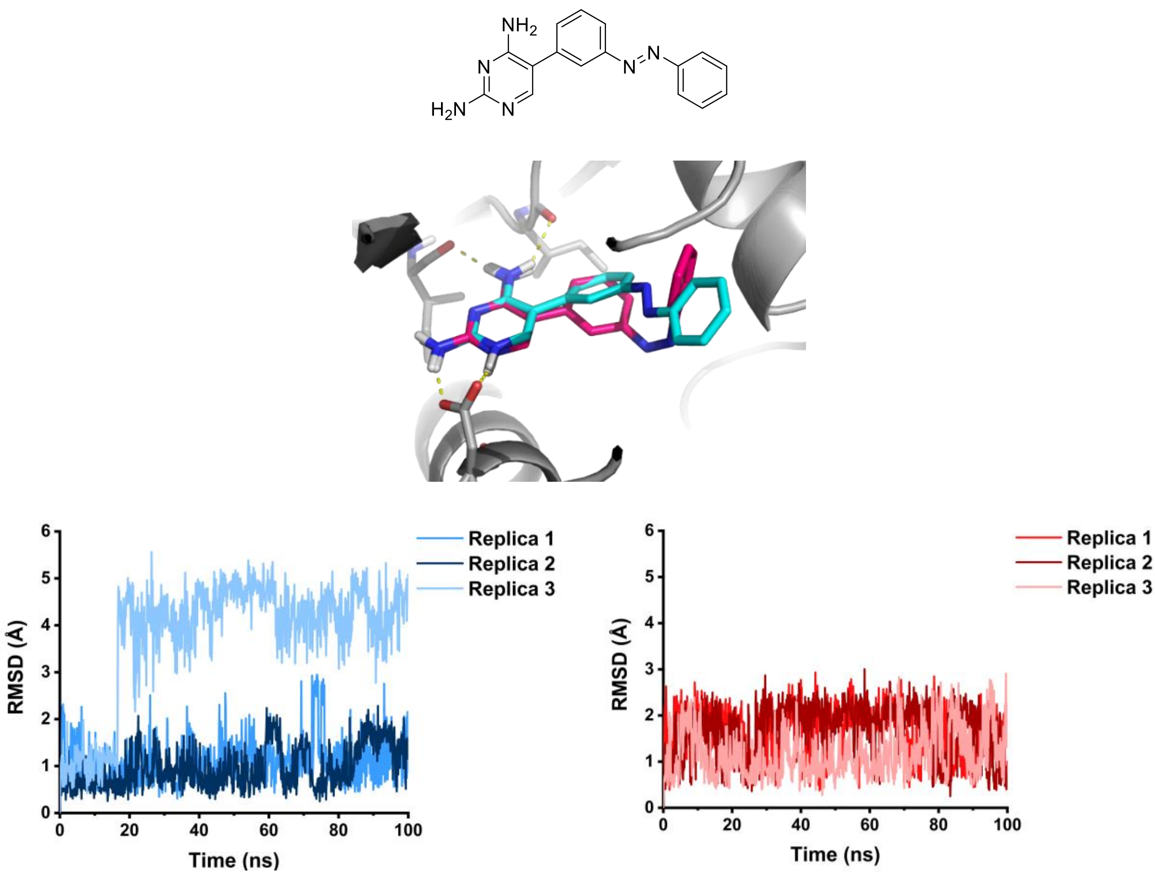

Figure S88. (top) Docking poses of *trans*-**3** (cyan) and *cis*-**3** (red). (bottom) RMSD of (left) *trans*-**3** and (right) *cis*-**3** throughout three replicas of 100 ns MD simulations.

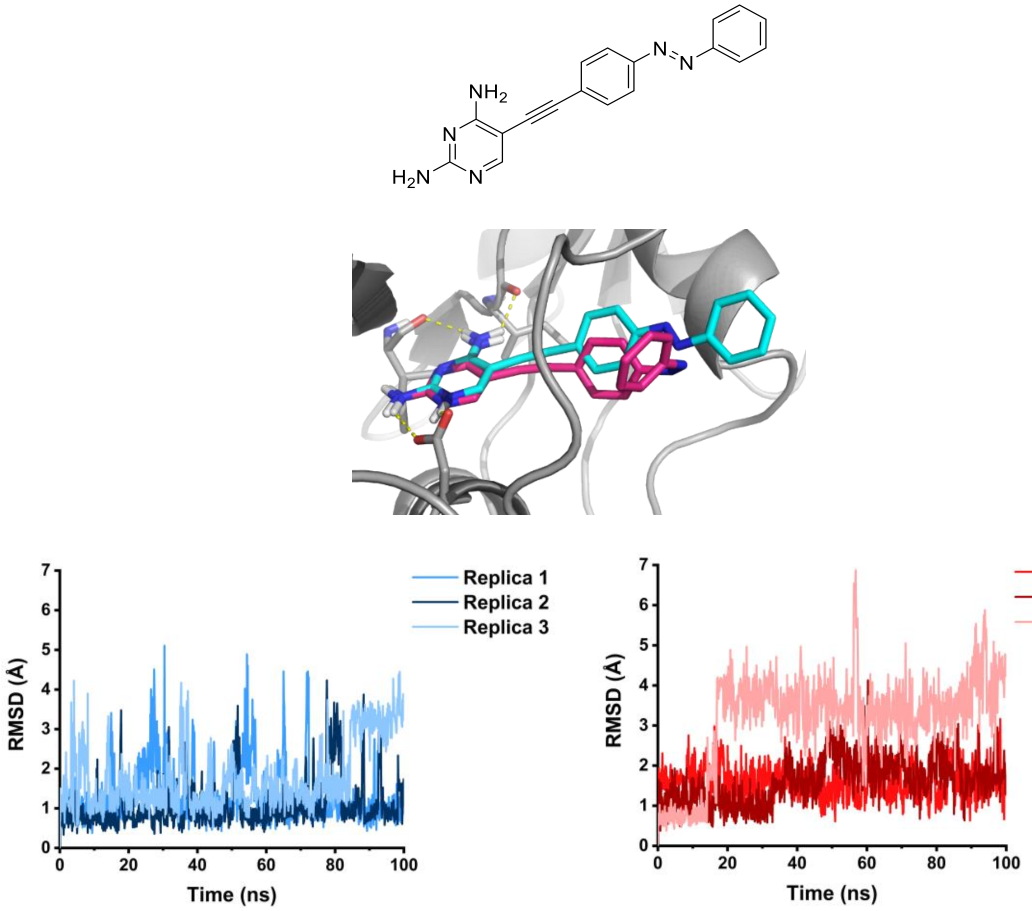

Figure S89. (top) Docking poses of *trans*-**4** (cyan) and *cis*-**4** (red). (bottom) RMSD of (left) *trans*-**4** and (right) *cis*-**4** throughout three replicas of 100 ns MD simulations.

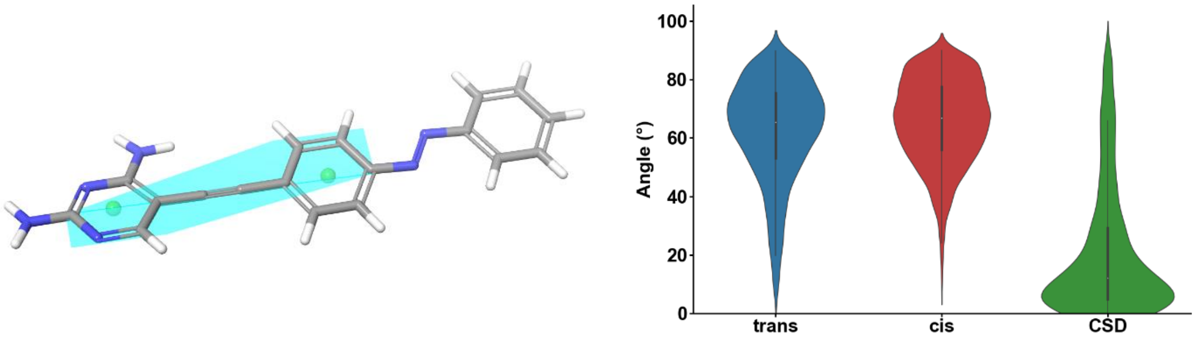

Figure S90. Distributions of ring angles between pyrimidine and the inner ring of azobenzene for *trans*-**4** (blue) and *cis*-**4** (red) throughout three replicas of 100 ns MD simulations, compared to the CSD distribution of ring angles between two generic rings linked by a triple bond (green).

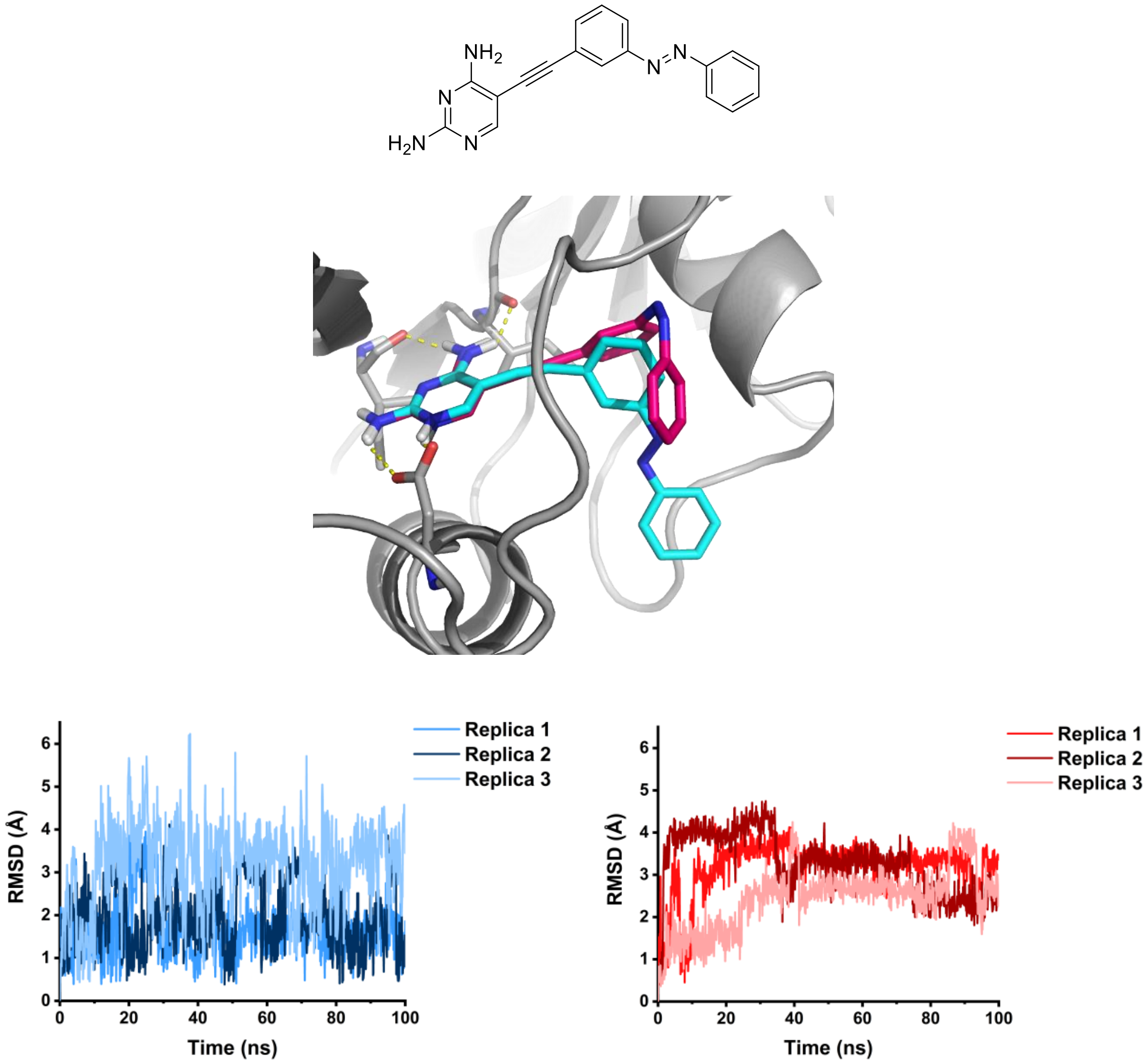

Figure S91. (top) Docking poses of *trans*-**5** (cyan) and *cis*-**5** (red). (bottom) RMSD of (left) *trans*-**5** and (right) *cis*-**5** throughout three replicas of 100 ns MD simulations.

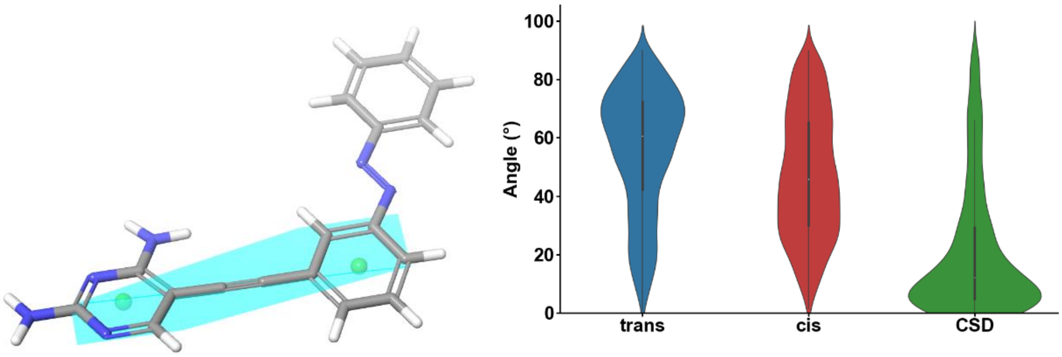

Figure S92. Distributions of ring angles between pyrimidine and the inner ring of azobenzene for *trans*-**5** (blue) and *cis*-**5** (red) throughout three replicas of 100 ns MD simulations, compared to the CSD distribution of ring angles between two generic rings linked by a triple bond (green).

S5.2.3. <u>Second hypothesis</u>

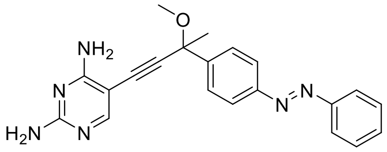

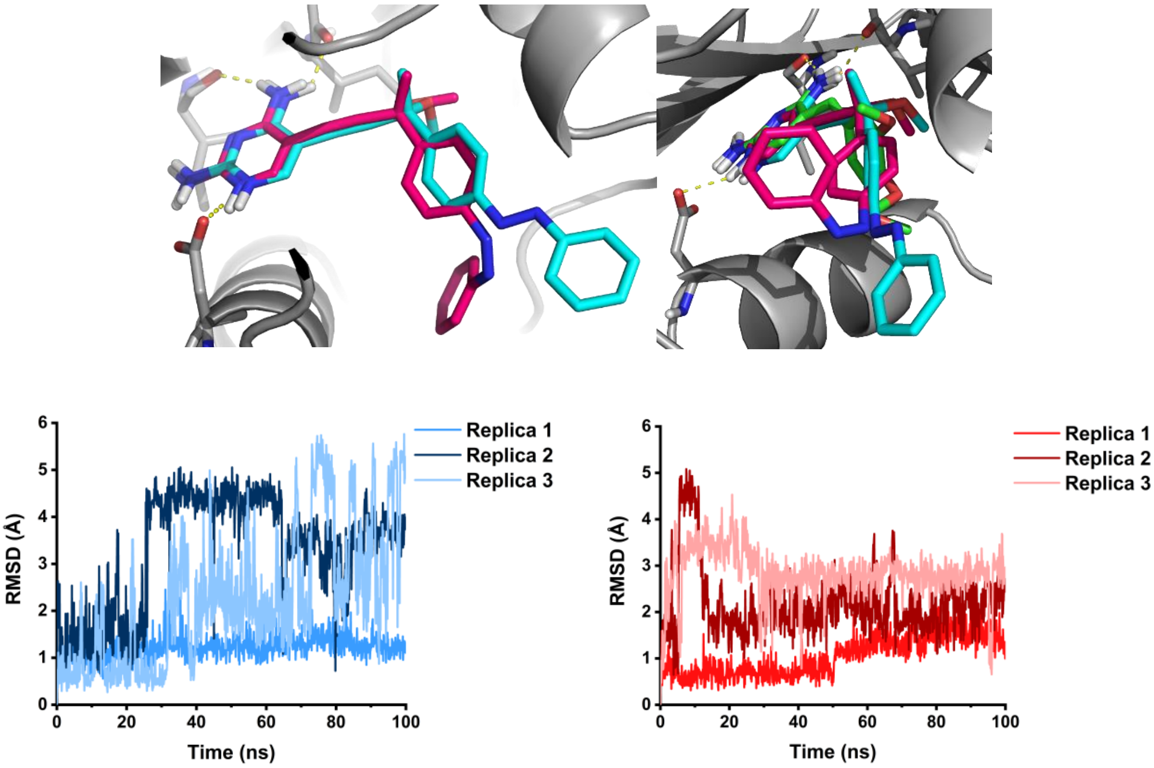

Figure S93. (top) Docking poses of *trans*-**6** (cyan) and *cis*-**6** (red), superimposed with TMP (green). (bottom) RMSD of (left) *trans*-**6** and (right) *cis*-**6** throughout three replicas of 100 ns MD simulations.

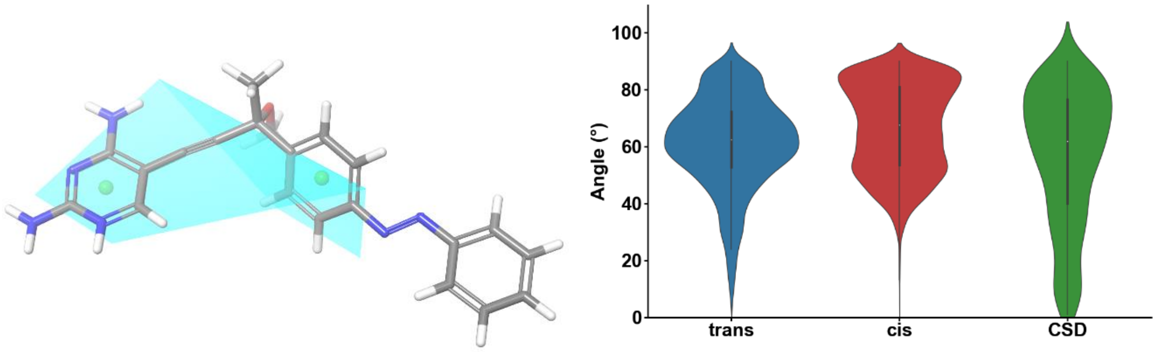

Figure S94. Distributions of ring angles between pyrimidine and the inner ring of azobenzene for *trans*-**6** (blue) and *cis*-**6** (red) throughout three replicas of 100 ns MD simulations, compared to the CSD distribution of ring angles between two generic rings linked by a triple bond (green).

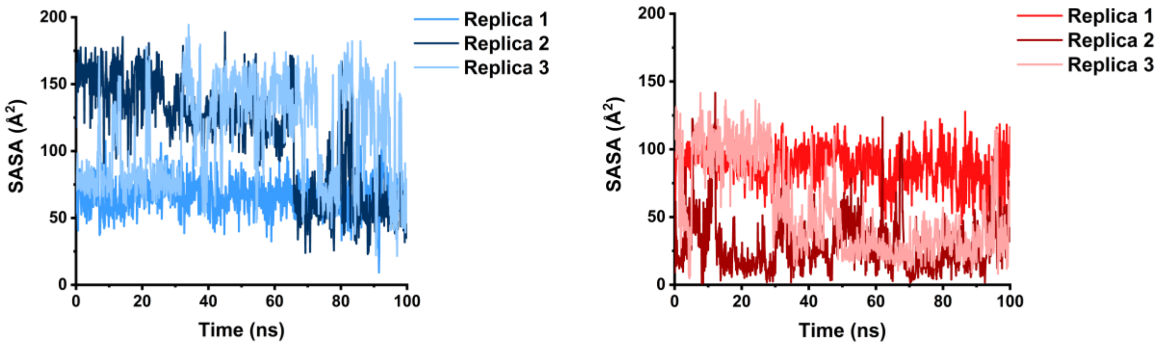

Figure S95. SASA of the outer benzene ring for (left) *trans*-**6** and (right) *cis*-**6** throughout three replicas of 100 ns MD simulations.

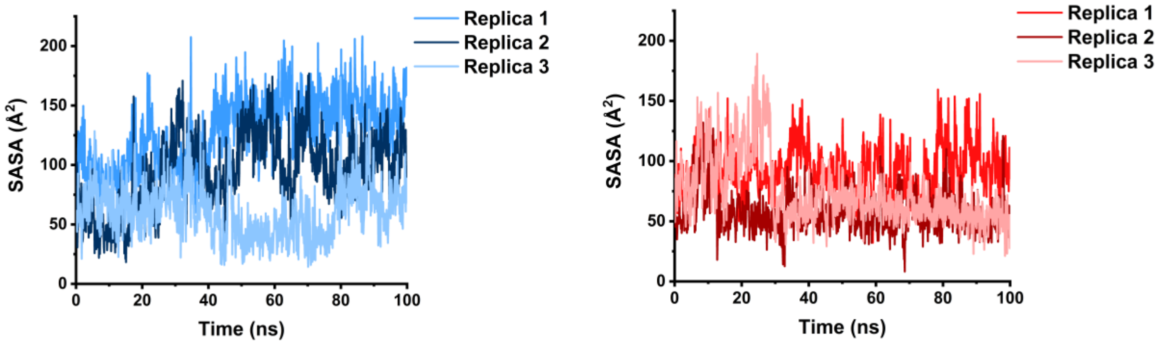

Figure S96. SASA of the lipophilic pocket for (left) *trans*-**6** and (right) *cis*-**6** throughout three replicas of 100 ns MD simulations.

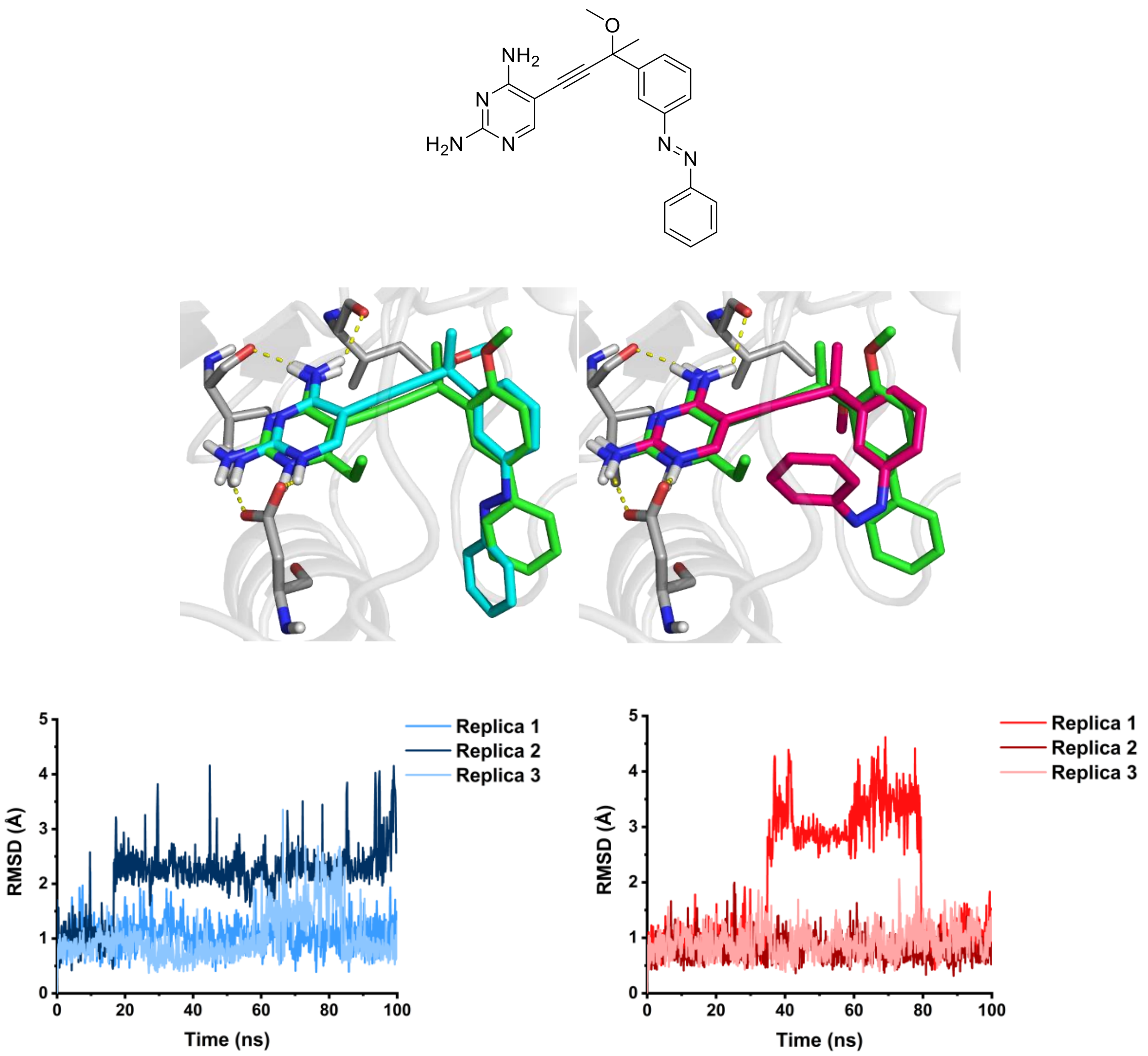

Figure S97. (top) Docking poses of *trans*-**7** (cyan) and *cis*-**7** (red), superimposed with **PLA** (green). (bottom) RMSD of (left) *trans*-**7** and (right) *cis*-**7** throughout three replicas of 100 ns MD simulations.

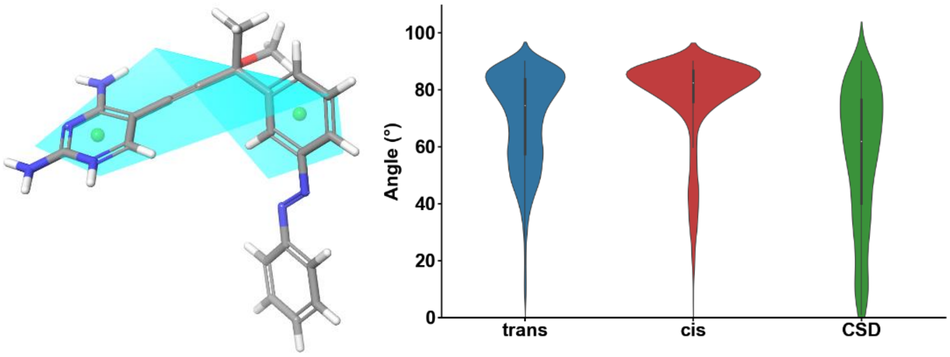

Figure S98. Distributions of ring angles between pyrimidine and the inner ring of azobenzene for *trans*-**7** (blue) and *cis*-**7** (red) throughout three replicas of 100 ns MD simulations, compared to the CSD distribution of ring angles between two generic rings linked by a triple bond (green).

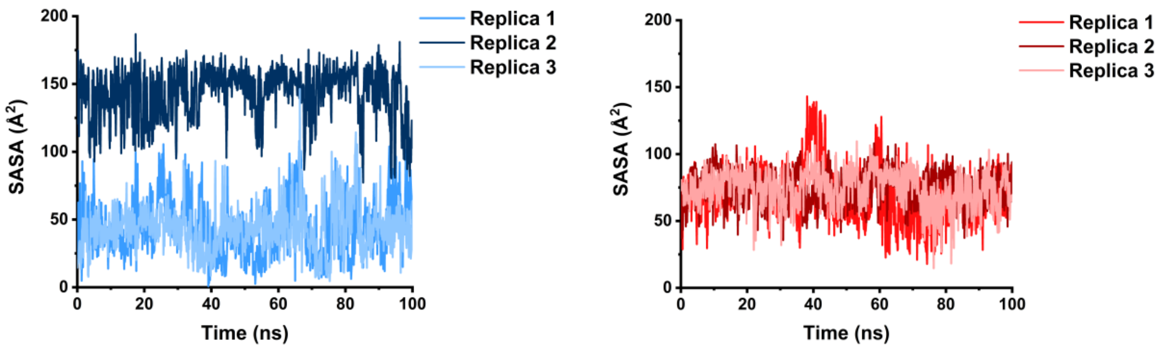

Figure S99. SASA of the outer benzene ring for (left) *trans*-**7** and (right) *cis*-**7** throughout three replicas of 100 ns MD simulations.

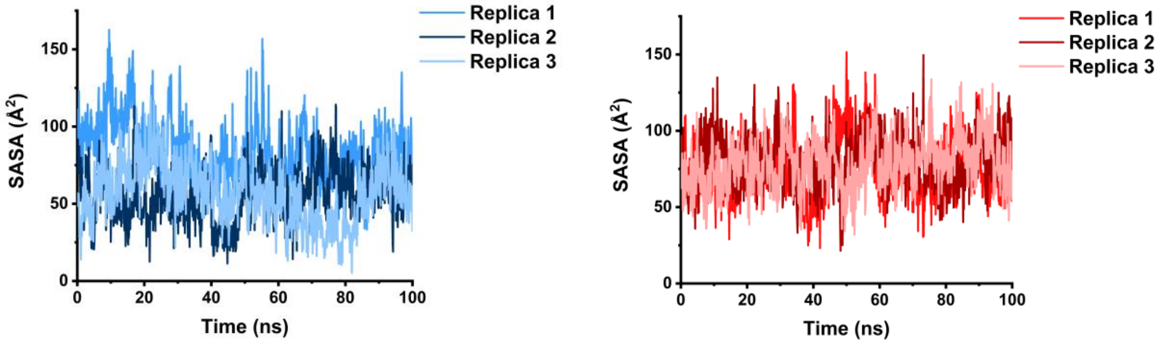

Figure S100. SASA of the lipophilic pocket for (left) *trans*-**7** and (right) *cis*-**7** throughout three replicas of 100 ns MD simulations.

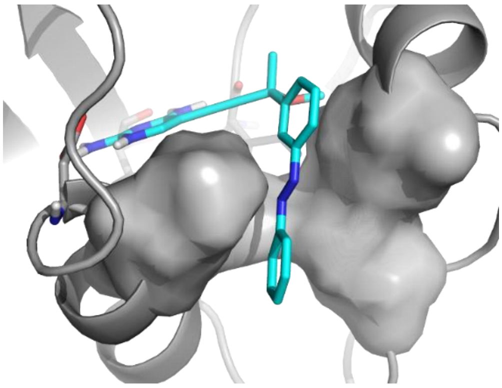

Figure S101. Docking pose of *trans*-**7** (cyan, hydrophobic subpocket as gray surface).

S5.2.4. Third hypothesis

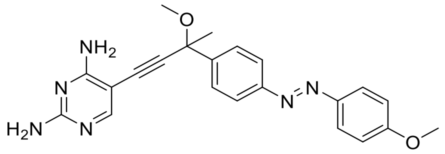

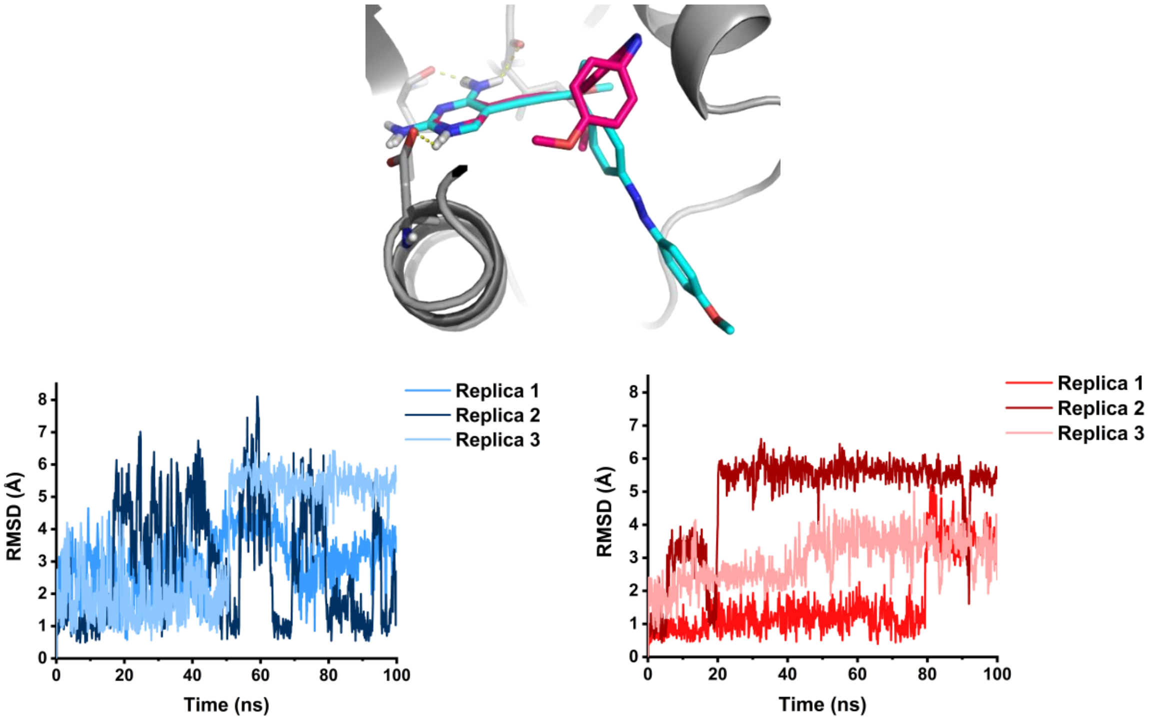

Figure S102. (top) Docking poses of *trans*-**8** (cyan) and *cis*-**8** (red). (bottom) RMSD of (left) *trans*-**8** and (right) *cis*-**8** throughout three replicas of 100 ns MD simulations.

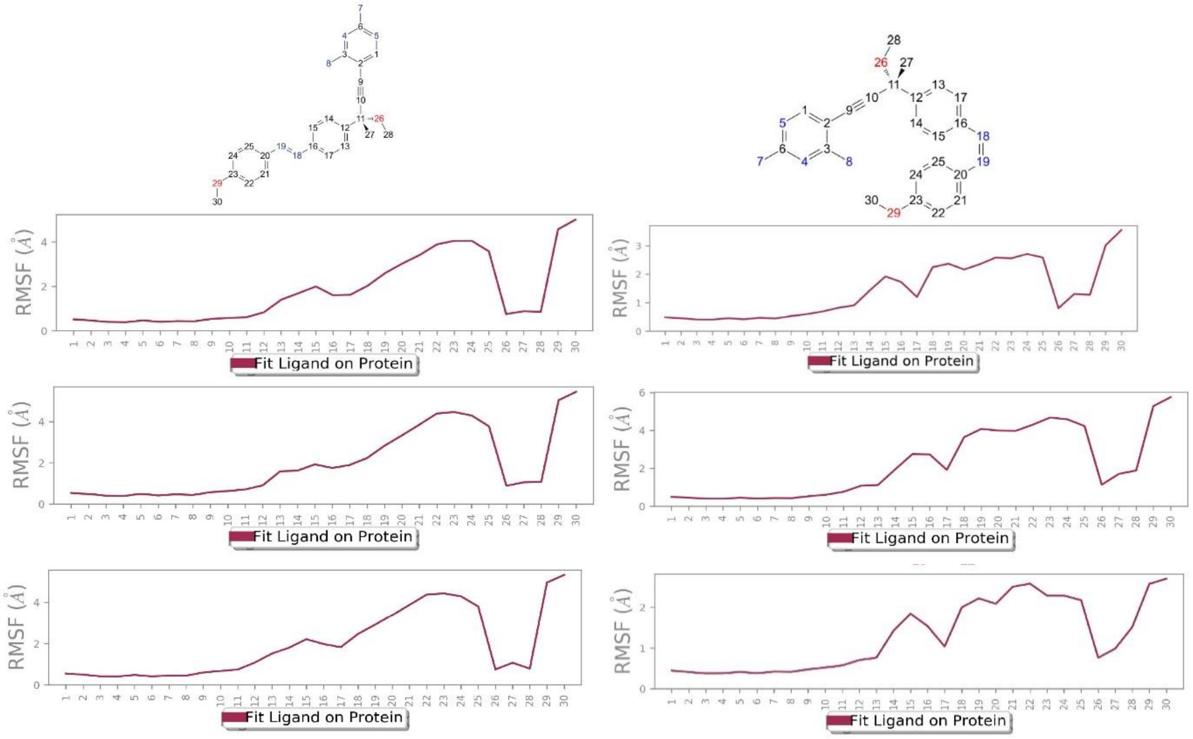

Figure S103. RMSF of (left) *trans*-**8** and (right) *cis*-**8** throughout three replicas of 100 ns MD simulations. Note the high RMSF value for the oxygen atom of the methoxy substituent (atom 29). Taken from the Simulation Interactions Diagram Report generated by Schrödinger.

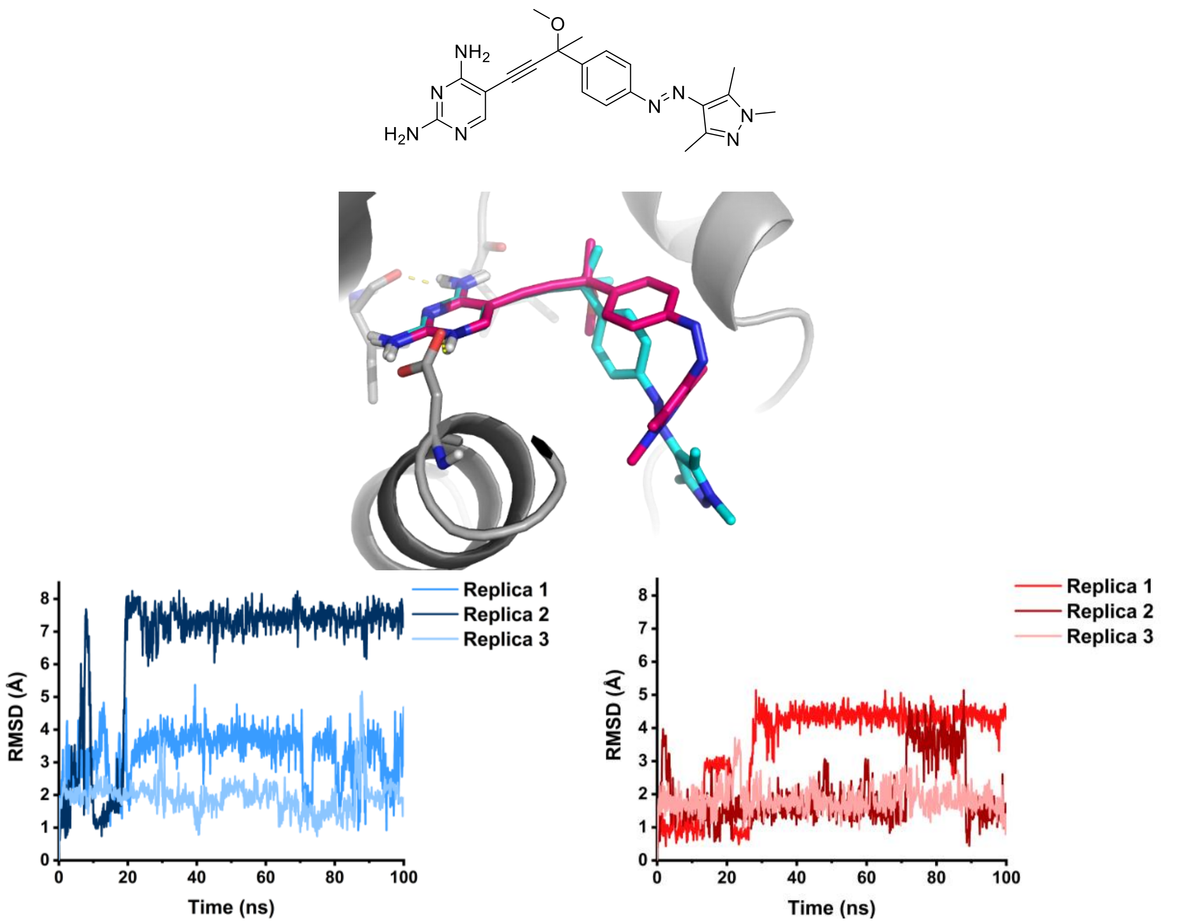

Figure S104. (top) Docking poses of *trans*-**9** (cyan) and *cis*-**9** (red). (bottom) RMSD of (left) *trans*-**9** and (right) *cis*-**9** throughout three replicas of 100 ns MD simulations.

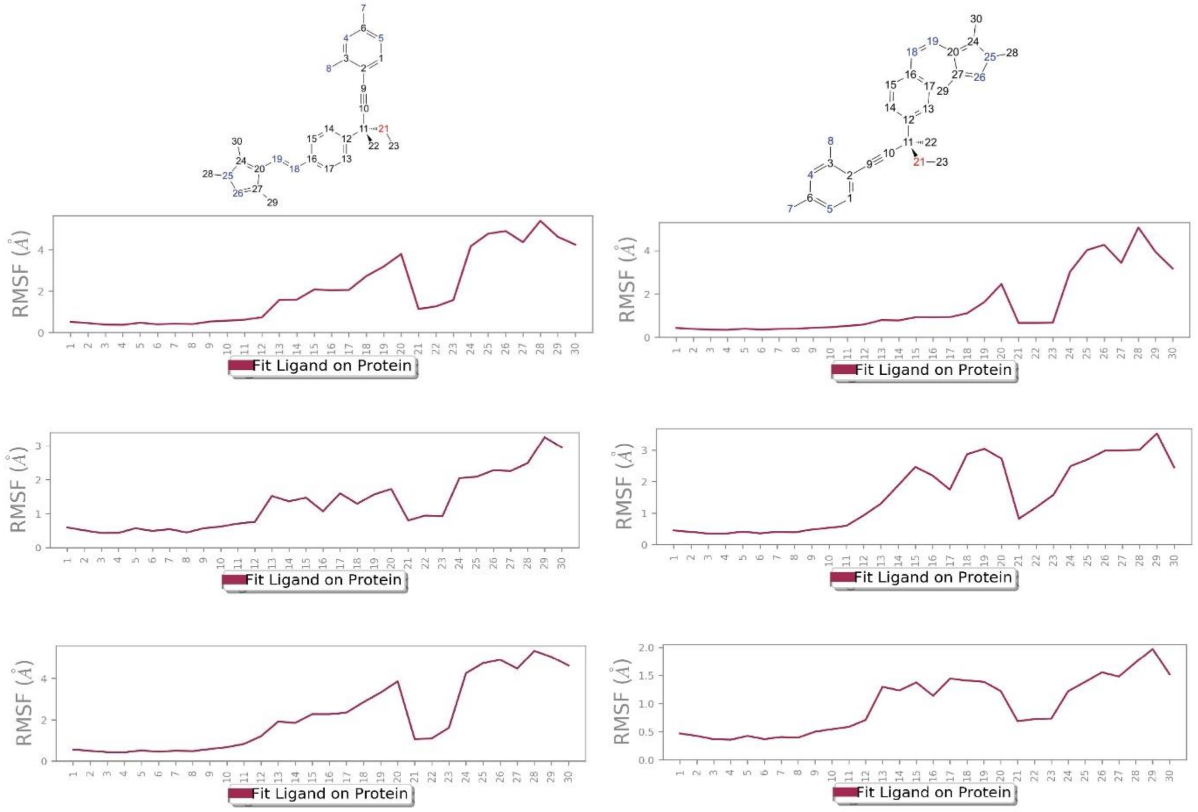

Figure S105. RMSF of (left) *trans*-**9** and (right) *cis*-**9** throughout three replicas of 100 ns MD simulations. Note the high RMSF value for the unsubstituted nitrogen atom of the pyrazole ring (atom 26). Taken from the Simulation Interactions Diagram Report generated by Schrödinger.

S5.2.5. Fourth hypothesis

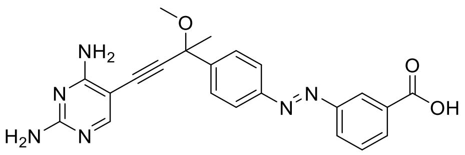

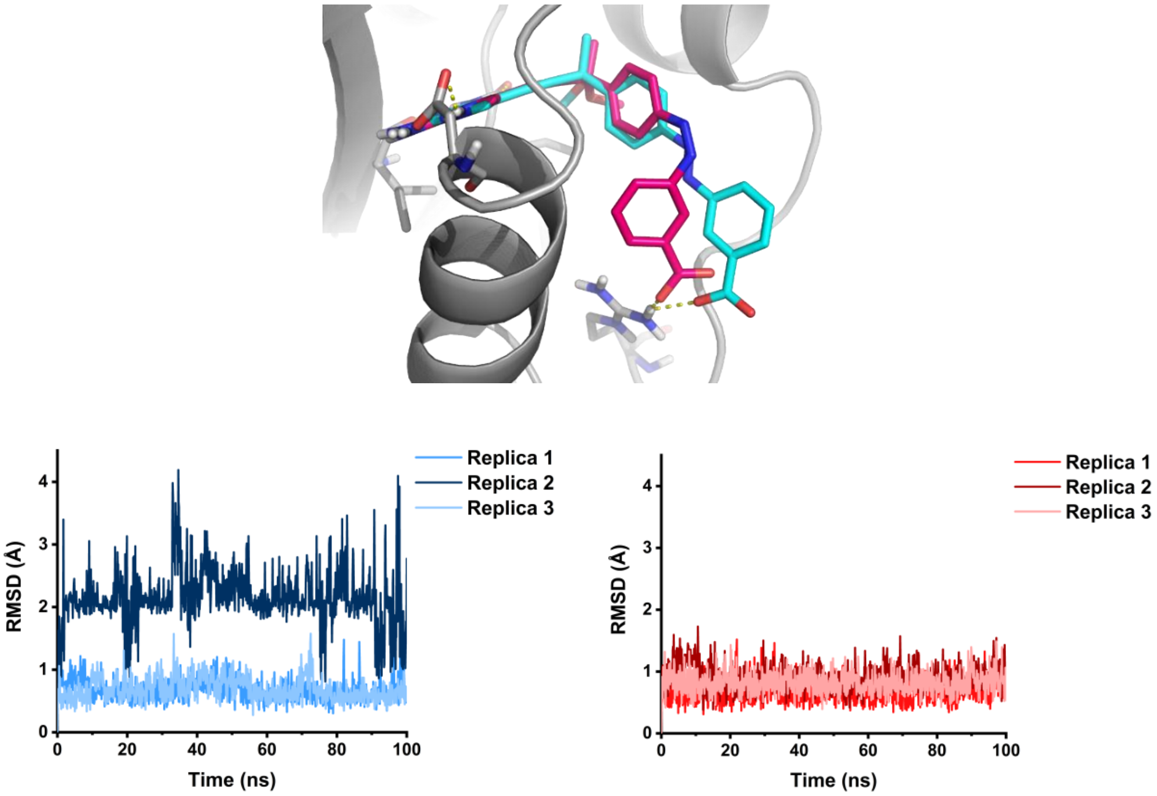

Figure S106. (top) Docking poses of *trans*-**10** (cyan) and *cis*-**10** (red). (bottom) RMSD of (left) *trans*-**10** and (right) *cis*-**10** throughout three replicas of 100 ns MD simulations.

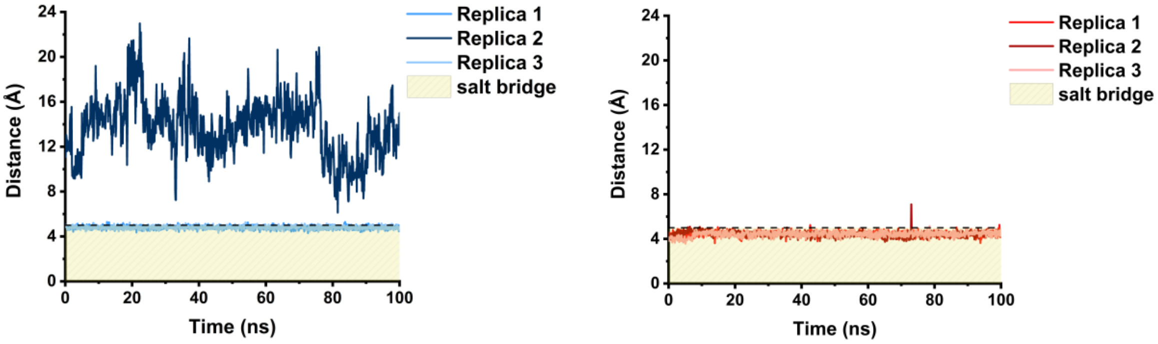

Figure S107. Distance between carboxylate and Arg57 for (left) *trans*-**10** and (right) *cis*-**10** throughout three replicas of 100 ns MD simulations.

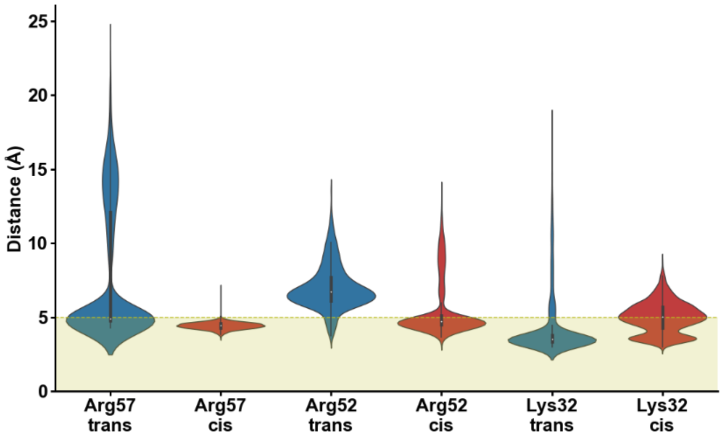

Figure S108. Violin plots of the distance between carboxylate and selected residues for *trans*- and *cis*-**10** throughout three replicas of 100 ns MD simulations.

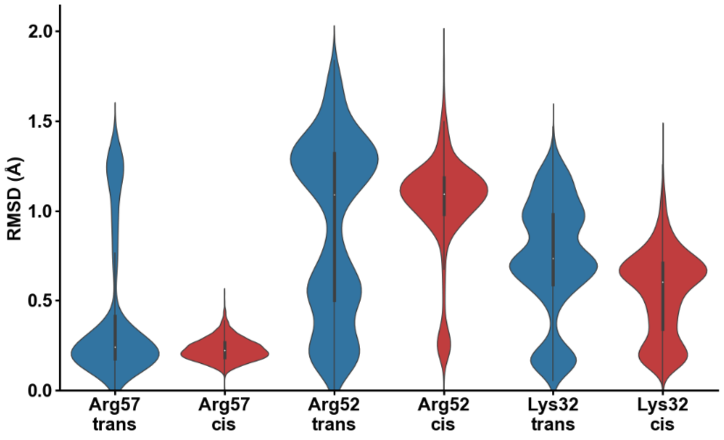

Figure S109. Violin plots of the RMSD of selected residues for *trans*- and *cis*-**10** throughout three replicas of 100 ns MD simulations.

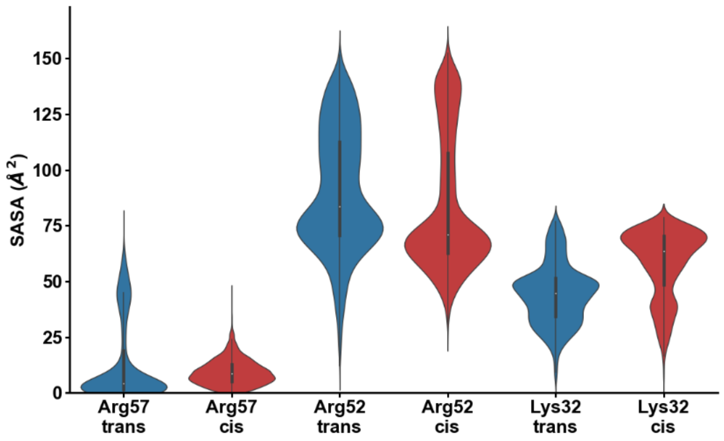

Figure S110. Violin plots of the SASA of selected residues for *trans*- and *cis*-**10** throughout three replicas of 100 ns MD simulations.

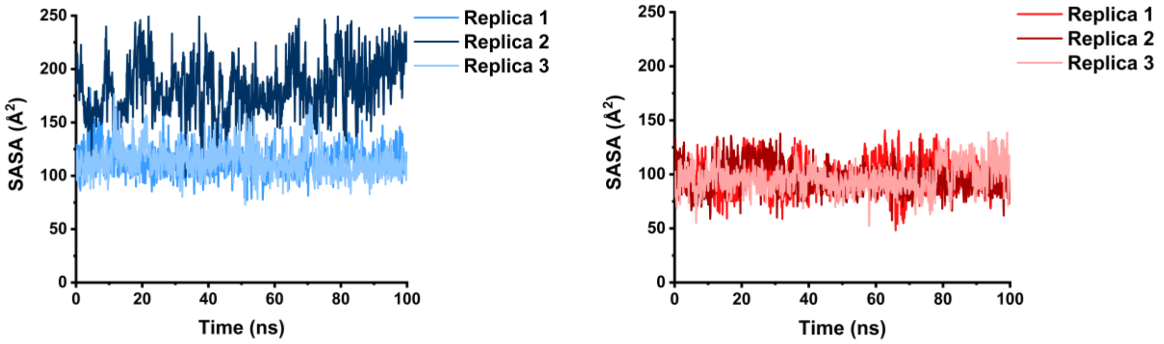

Figure S111. SASA of (left) *trans*-**10** and (right) *cis*-**10** throughout three replicas of 100 ns MD simulations.

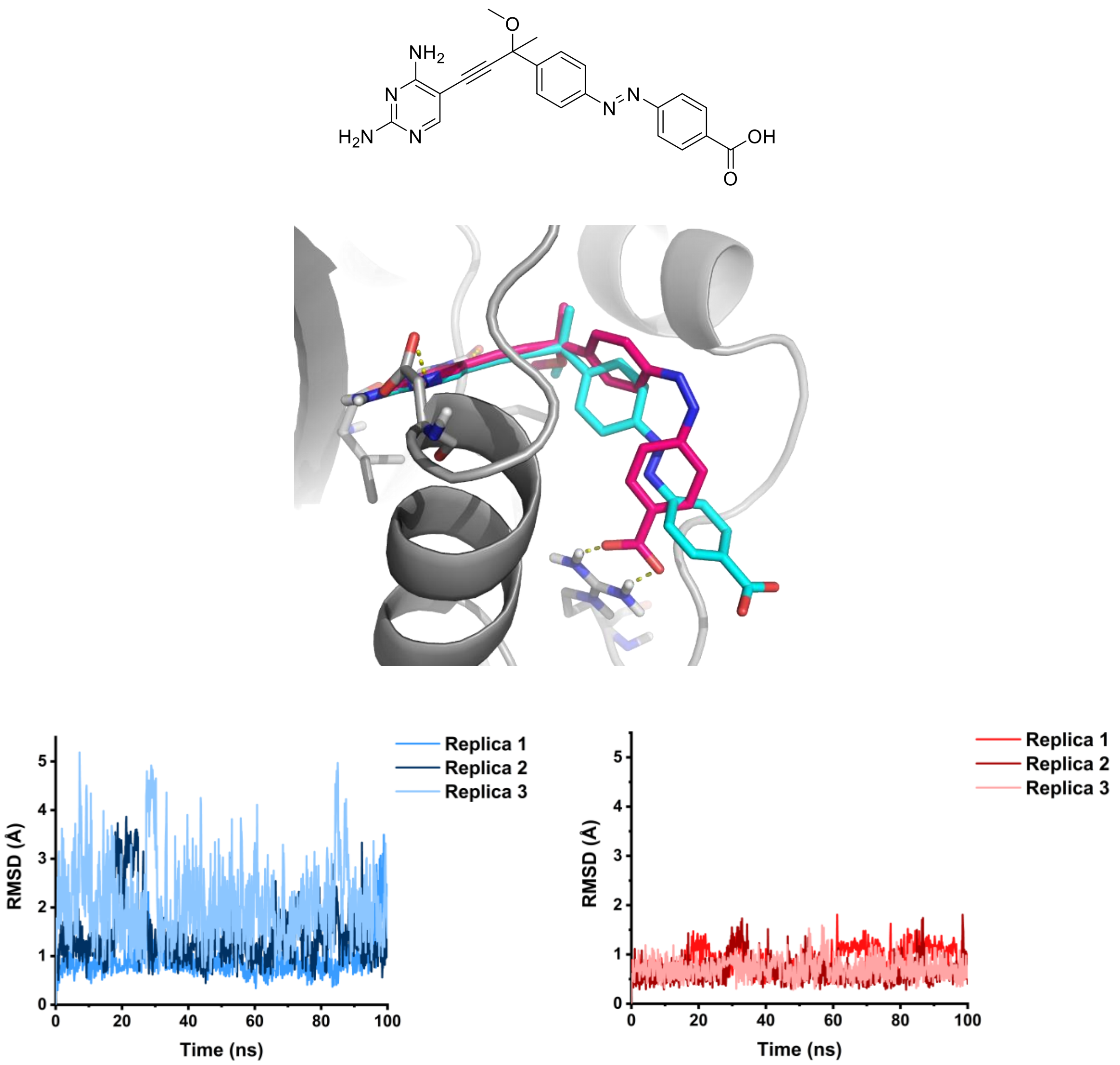

Figure S112. (top) Docking poses of *trans*-**11** (cyan) and *cis*-**11** (red). (bottom) RMSD of (left) *trans*-**11** and (right) *cis*-**11** throughout three replicas of 100 ns MD simulations.

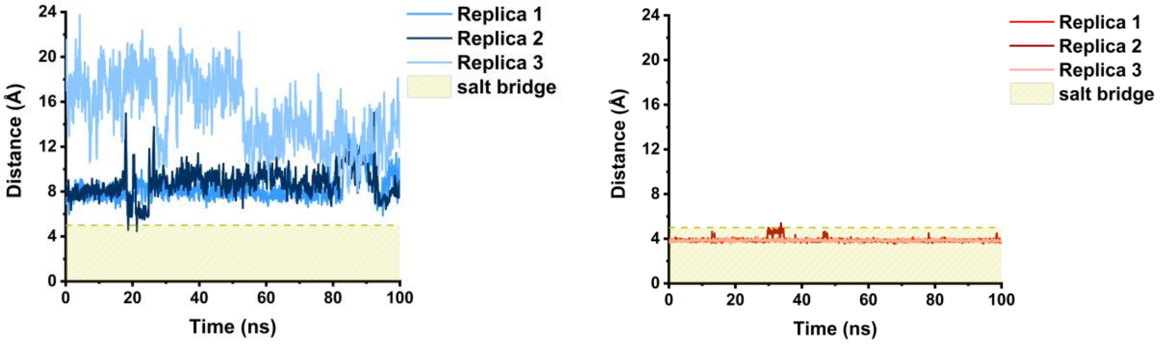

Figure S113. Distance between carboxylate and Arg57 for (left) *trans*-**11** and (right) *cis*-**11** throughout three replicas of 100 ns MD simulations.

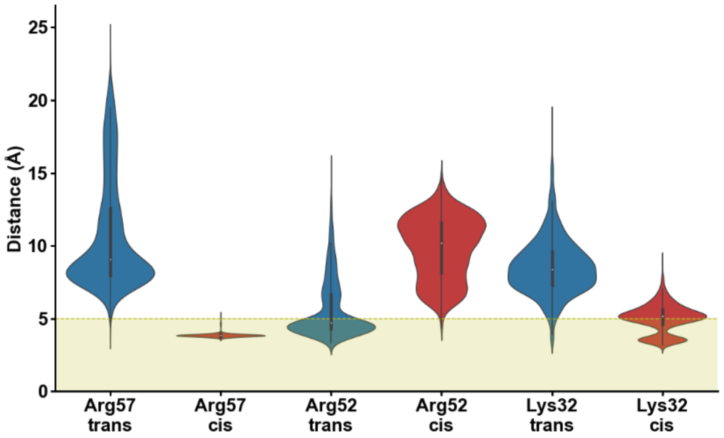

Figure S114. Violin plots of the distance between carboxylate and selected residues for *trans*- and *cis*-**11** throughout three replicas of 100 ns MD simulations.

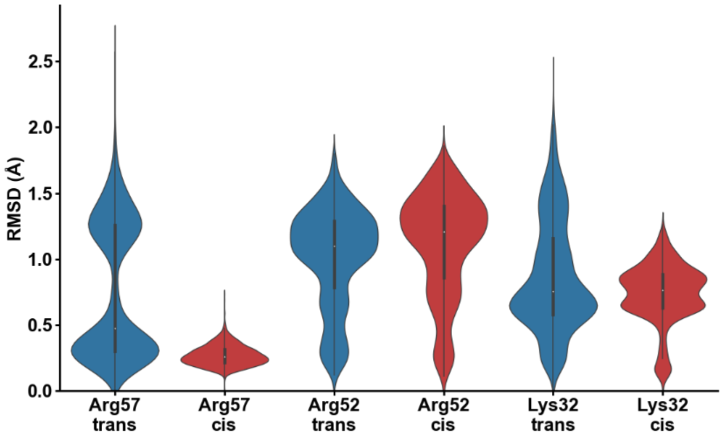

Figure S115. Violin plots of the RMSD of selected residues for *trans*- and *cis*-**11** throughout three replicas of 100 ns MD simulations.

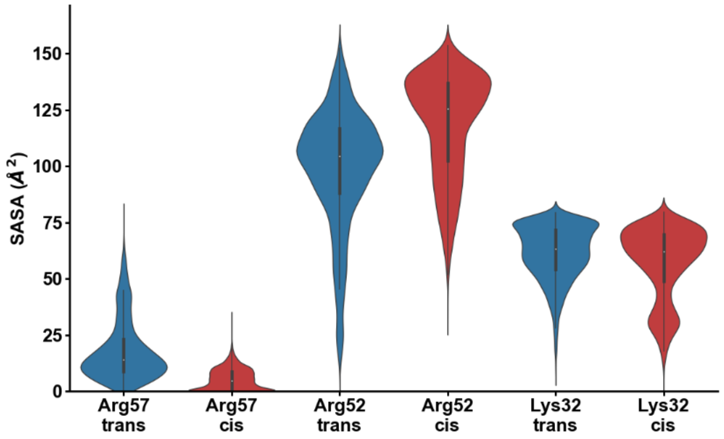

Figure S116. Violin plots of the SASA of selected residues for *trans*- and *cis*-**11** throughout three replicas of 100 ns MD simulations.

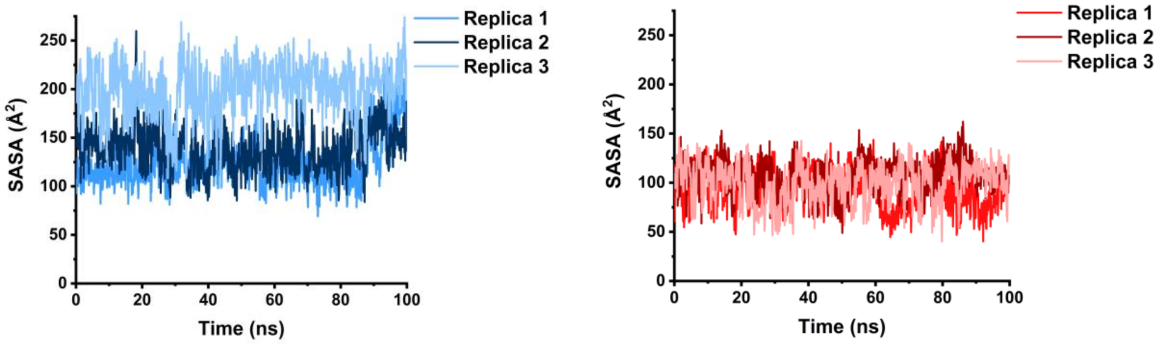

Figure S117. SASA of (left) *trans*-**11** and (right) *cis*-**11** throughout three replicas of 100 ns MD simulations.

S5.2.6. Fifth hypothesis

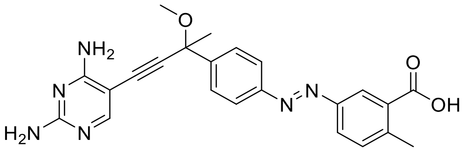

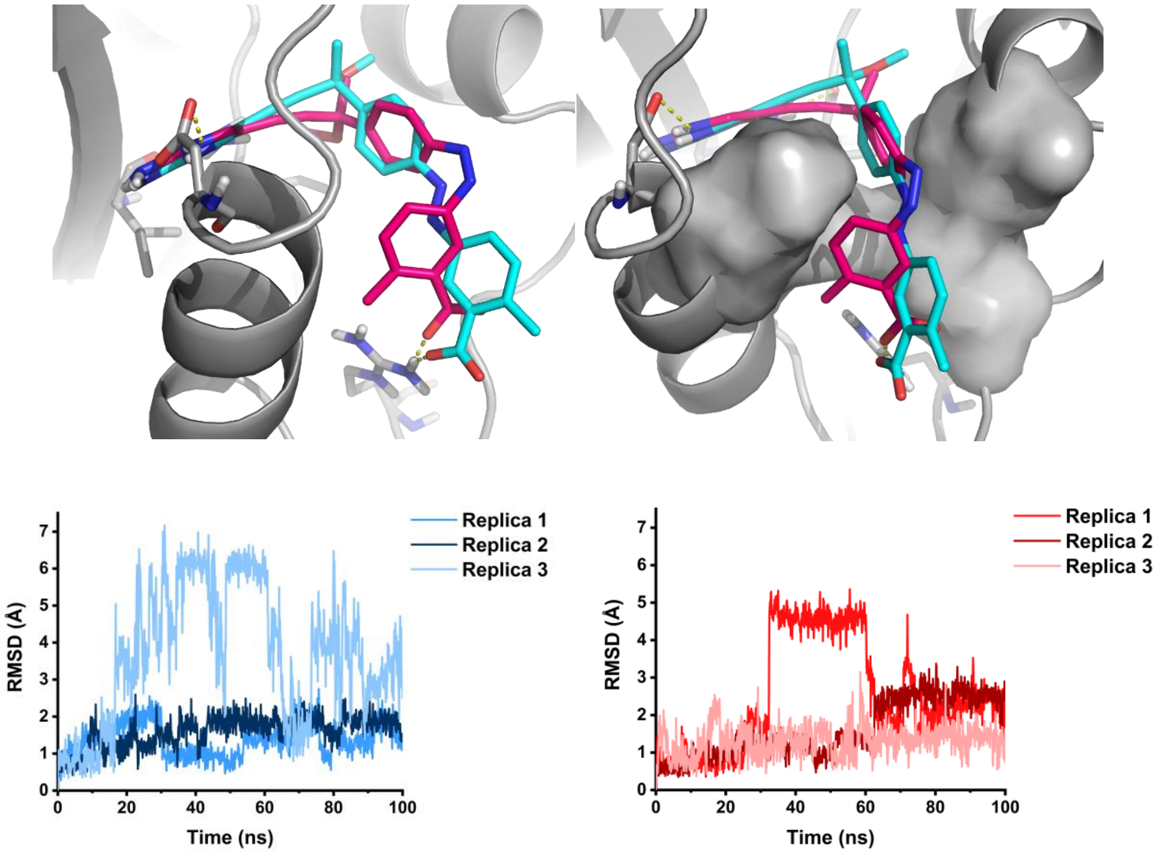

Figure S118. (top) Docking poses of *trans*-**12** (cyan) and *cis*-**12** (red). The hydrophobic subpocket is depicted as a gray surface. (bottom) RMSD of (left) *trans*-**12** and (right) *cis*-**12** throughout three replicas of 100 ns MD simulations.

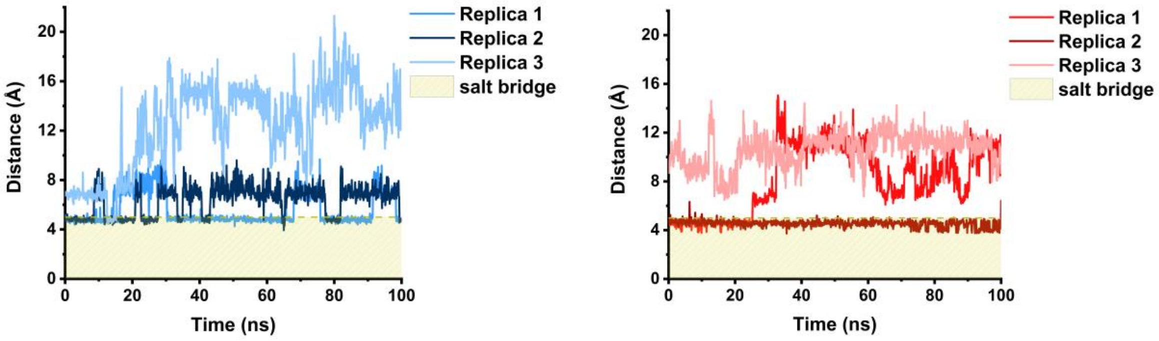

Figure S119. Distance between carboxylate and Arg57 for (left) *trans*-**12** and (right) *cis*-**12** throughout three replicas of 100 ns MD simulations.

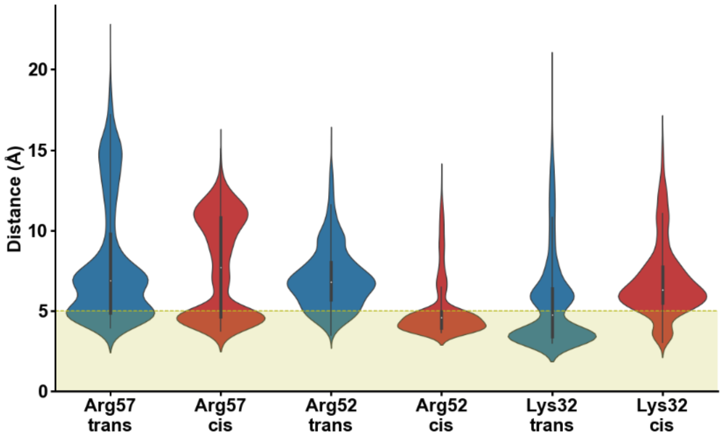

Figure S120. Violin plots of the distance between carboxylate and selected residues for *trans*- and *cis*-**12** throughout three replicas of 100 ns MD simulations.

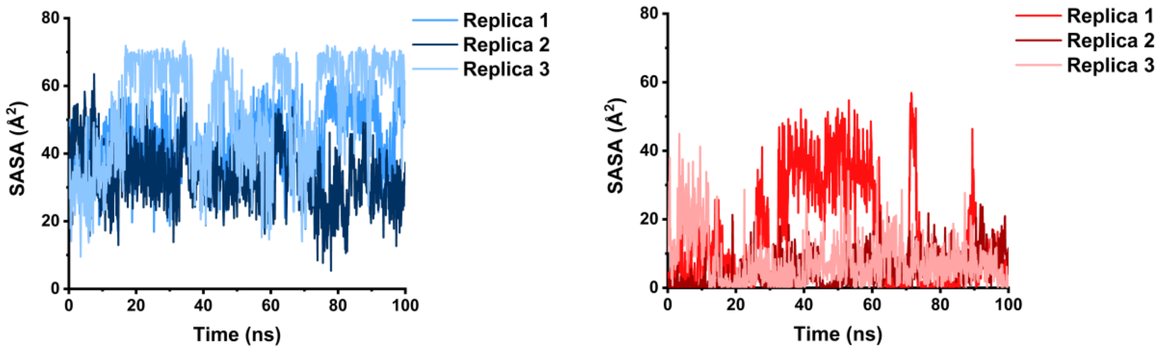

Figure S121. SASA of the methyl group on the outer ring for (left) *trans*-**12** and (right) *cis*-**12** throughout three replicas of 100 ns MD simulations.

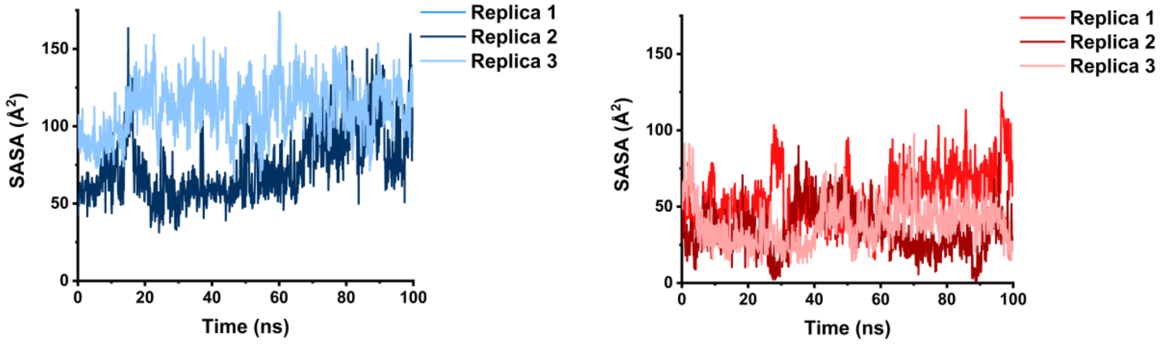

Figure S122. SASA of the lipophilic pocket for (left) *trans*-**12** and (right) *cis*-**12** throughout three replicas of 100 ns MD simulations.

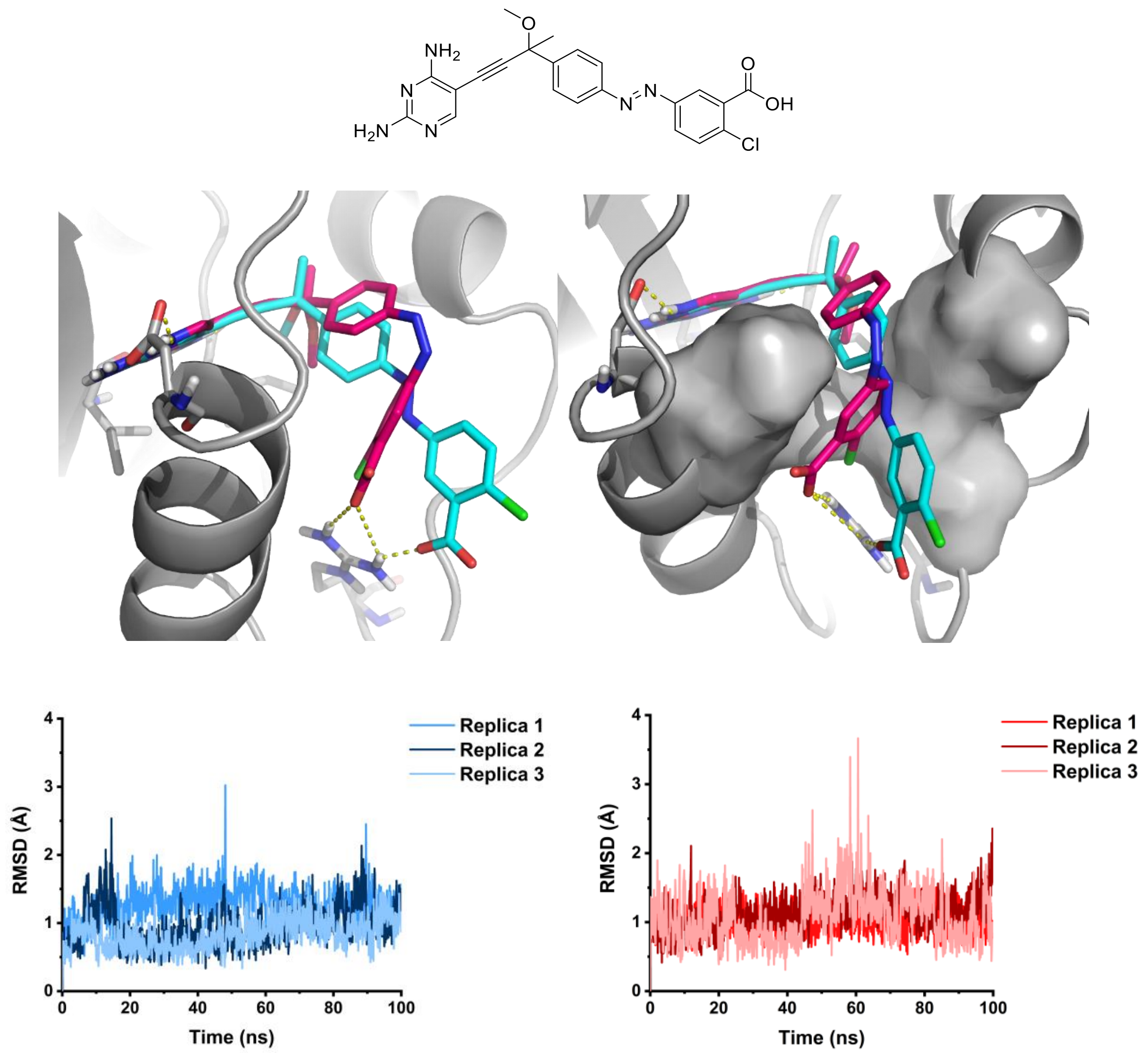

Figure S123. (top) Docking poses of *trans*-**13** (cyan) and *cis*-**13** (red). The hydrophobic subpocket is depicted as a gray surface. (bottom) RMSD of (left) *trans*-**13** and (right) *cis*-**13** throughout three replicas of 100 ns MD simulations.

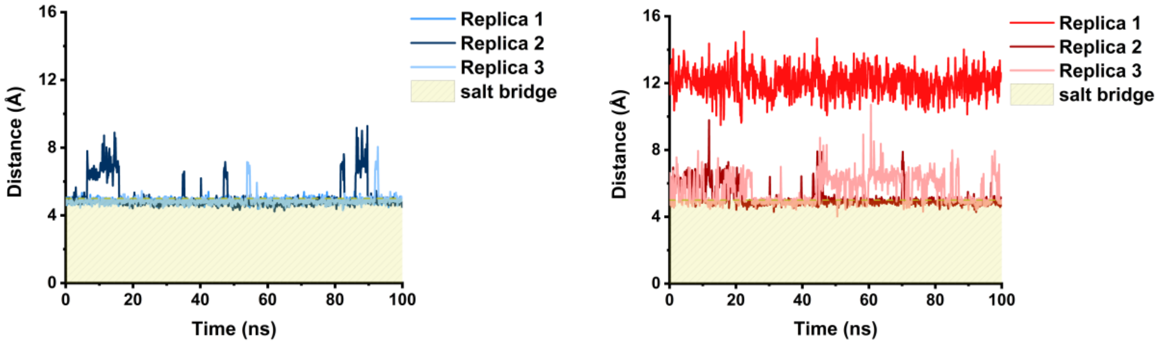

Figure S124. Distance between carboxylate and Arg57 for (left) *trans*-**13** and (right) *cis*-**13** throughout three replicas of 100 ns MD simulations.

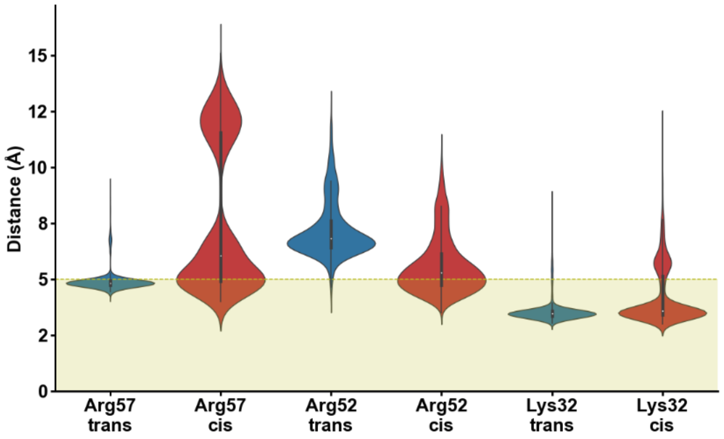

Figure S125. Violin plots of the distance between carboxylate and selected residues for *trans*- and *cis*-**13** throughout three replicas of 100 ns MD simulations.

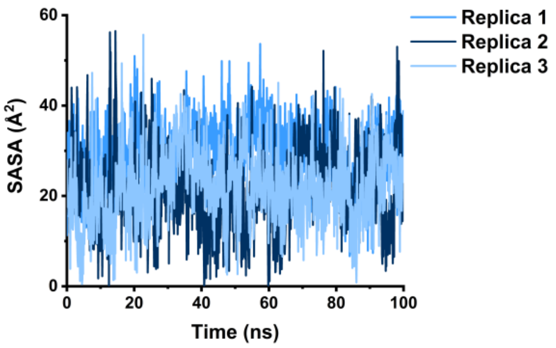

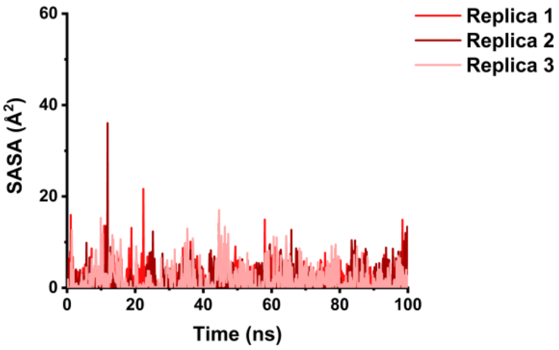

Figure S126. SASA of the chlorine substituent on the outer ring for (left) *trans*-**13** and (right) *cis*-**13** throughout three replicas of 100 ns MD simulations.

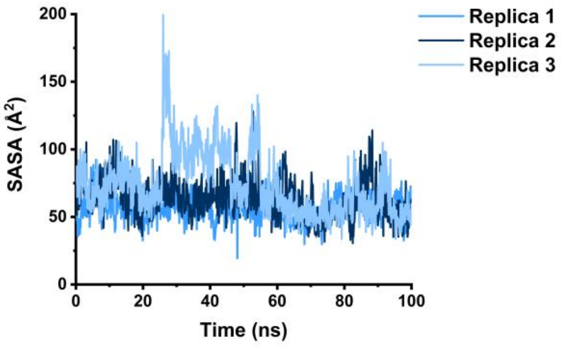

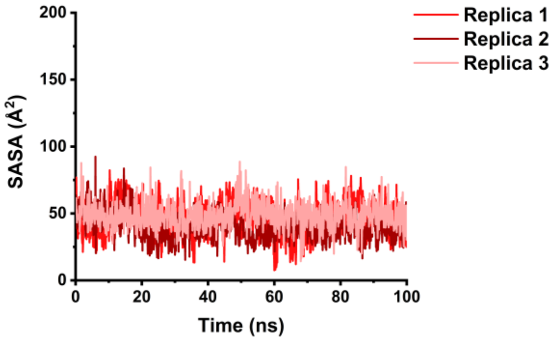

Figure S127. SASA of the lipophilic pocket for (left) *trans*-**13** and (right) *cis*-**13** throughout three replicas of 100 ns MD simulations.

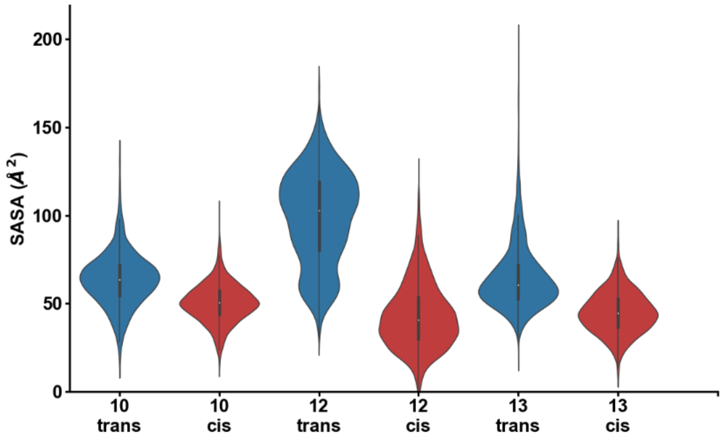

Figure S128. Violin plots of the SASA of the lipophilic pocket for inhibitors with a *m*-COOH substituent throughout three replicas of 100 ns MD simulations.

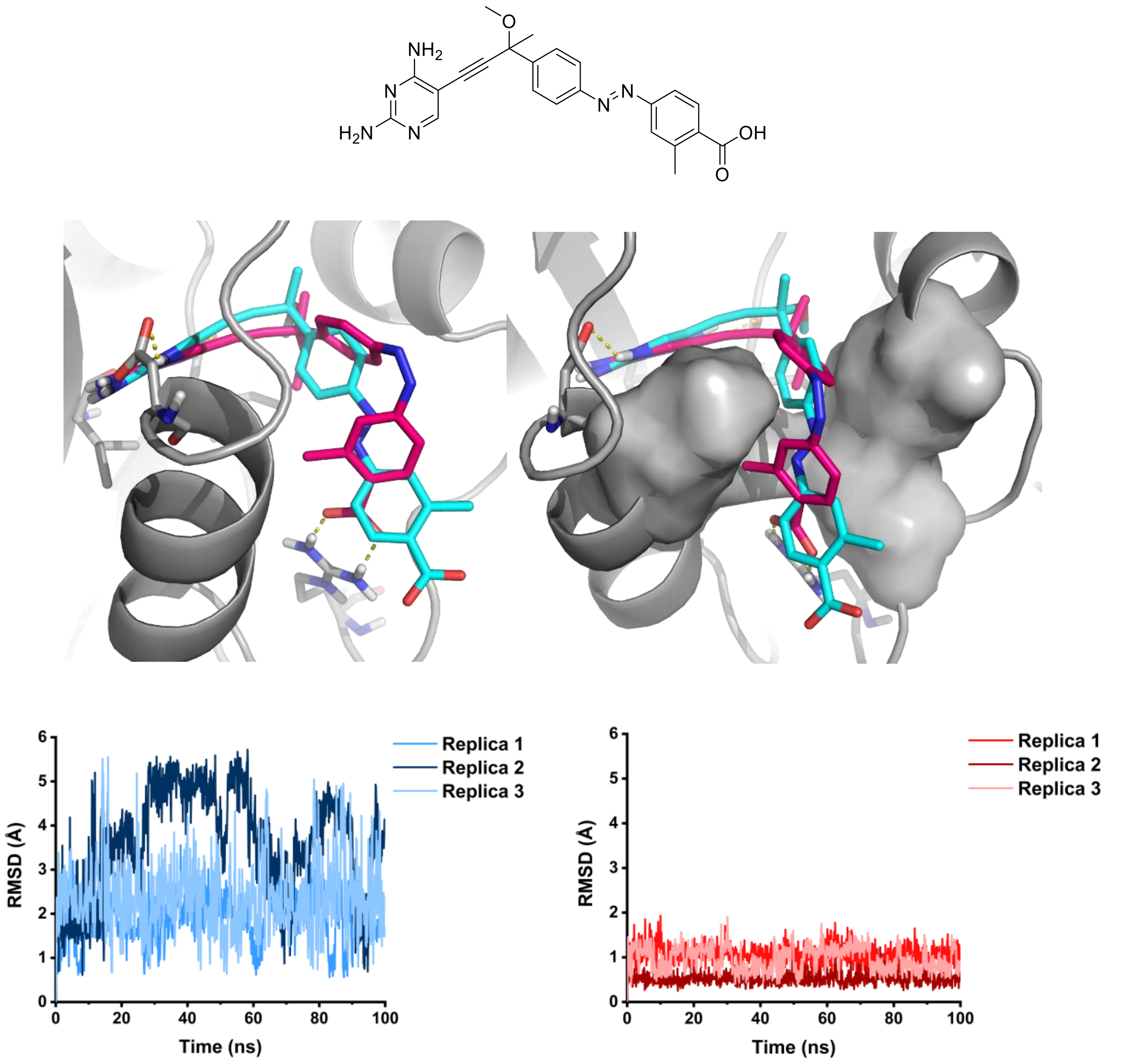

Figure S129. (top) Docking poses of *trans*-**14** (cyan) and *cis*-**14** (red). The hydrophobic subpocket is depicted as a gray surface. (bottom) RMSD of (left) *trans*-**14** and (right) *cis*-**14** throughout three replicas of 100 ns MD simulations.

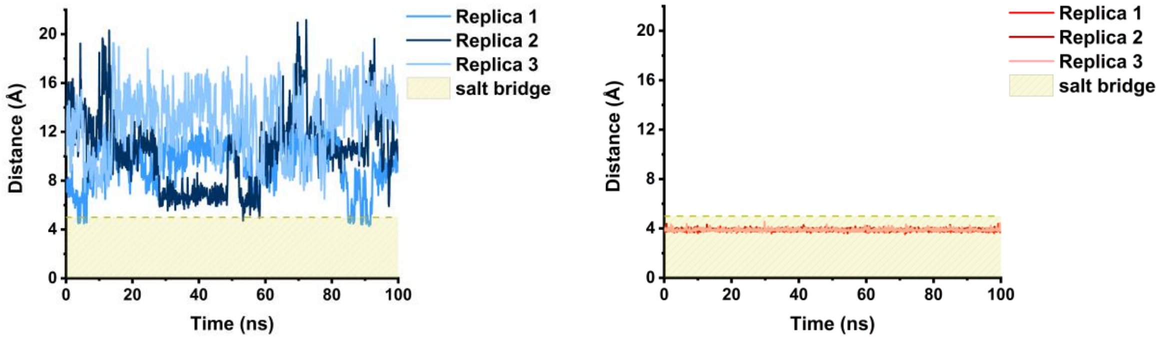

Figure S130. Distance between carboxylate and Arg57 for (left) *trans*-**14** and (right) *cis*-**14** throughout three replicas of 100 ns MD simulations.

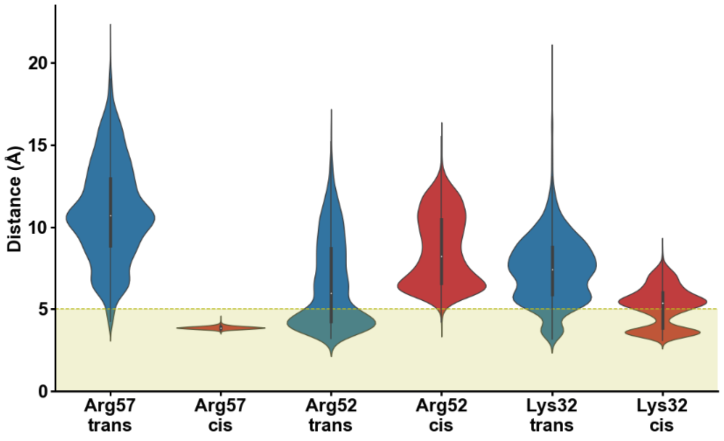

Figure S131. Violin plots of the distance between carboxylate and selected residues for *trans*- and *cis*-**14** throughout three replicas of 100 ns MD simulations.

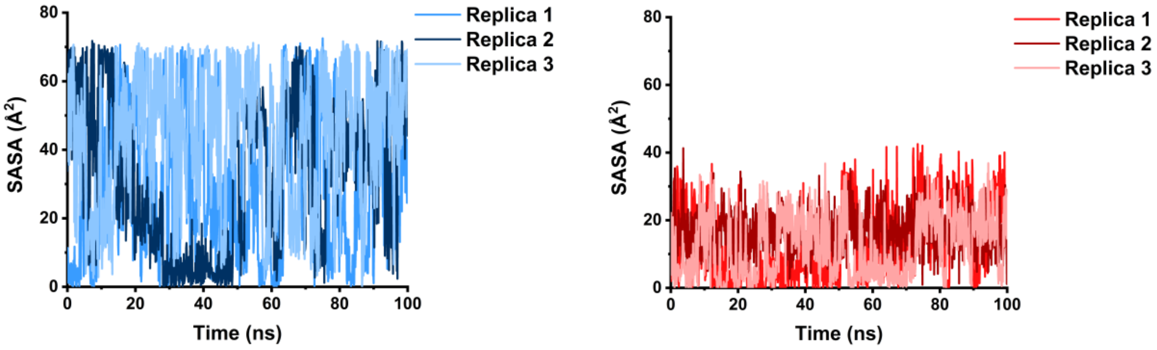

Figure S132. SASA of the methyl group on the outer ring for (left) *trans*-**14** and (right) *cis*-**14** throughout three replicas of 100 ns MD simulations.

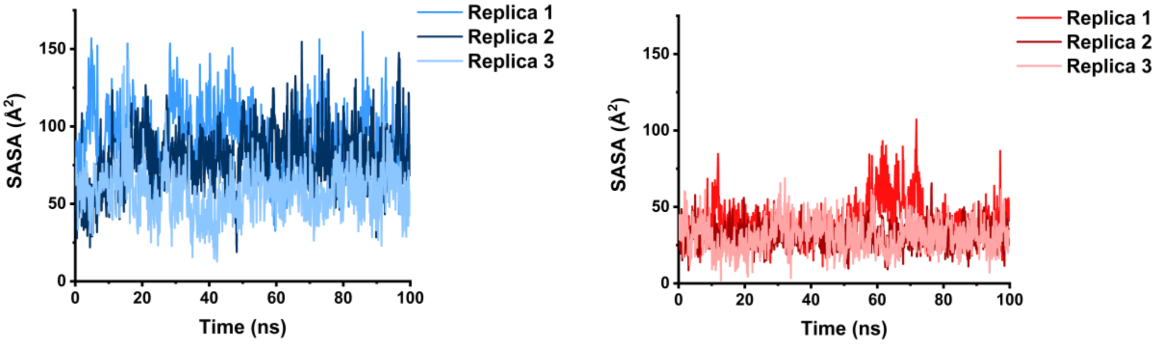

Figure S133. SASA of the lipophilic pocket for (left) *trans*-**14** and (right) *cis*-**14** throughout three replicas of 100 ns MD simulations.

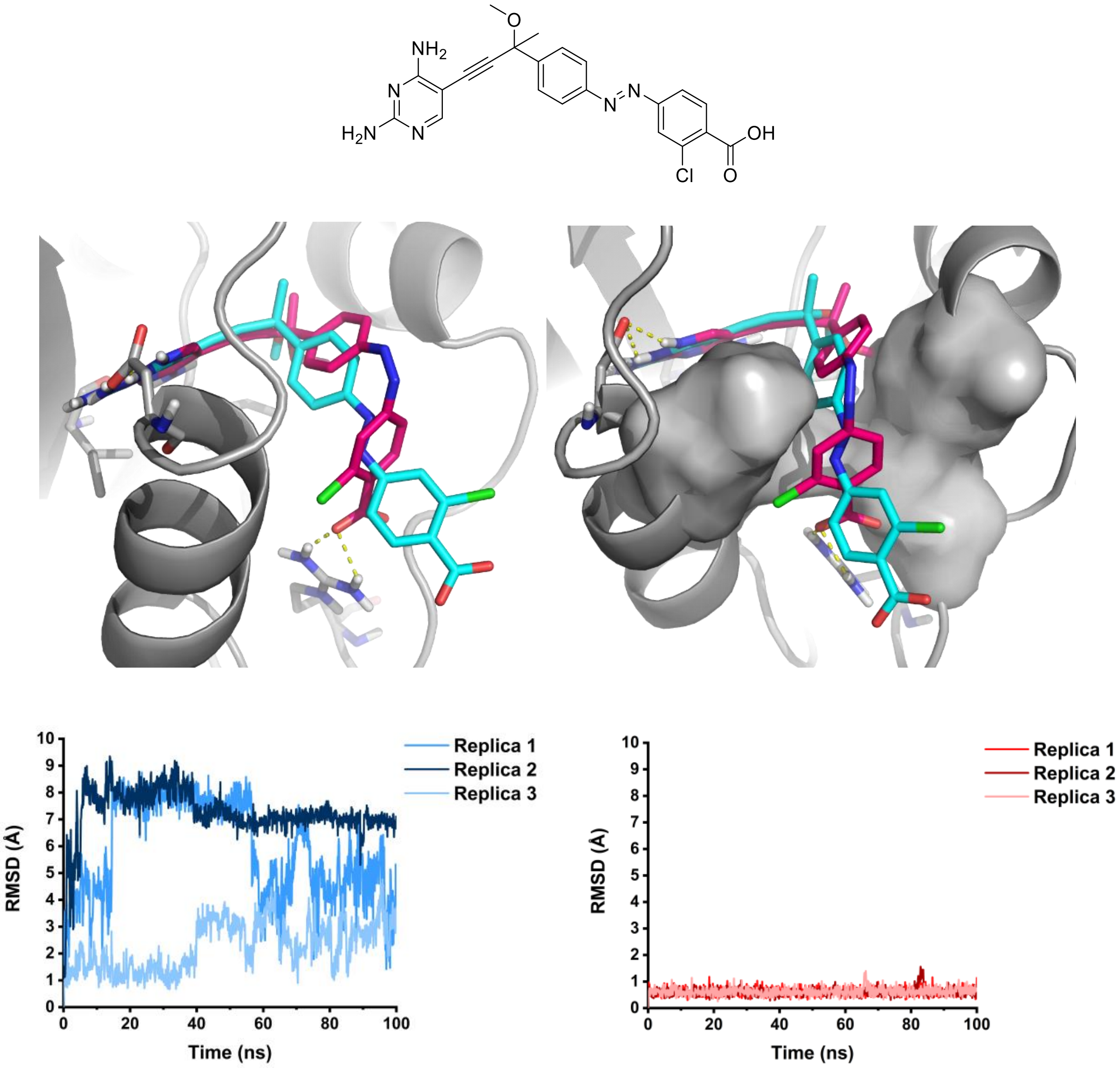

Figure S134. (top) Docking poses of *trans*-**15** (cyan) and *cis*-**15** (red). The hydrophobic subpocket is depicted as a gray surface. (bottom) RMSD of (left) *trans*-**15** and (right) *cis*-**15** throughout three replicas of 100 ns MD simulations.

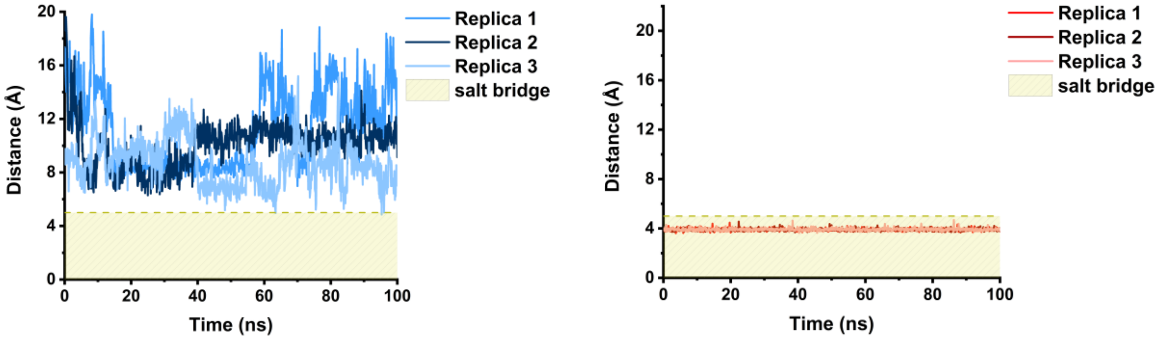

Figure S135. Distance between carboxylate and Arg57 for (left) *trans*-**15** and (right) *cis*-**15** throughout three replicas of 100 ns MD simulations.

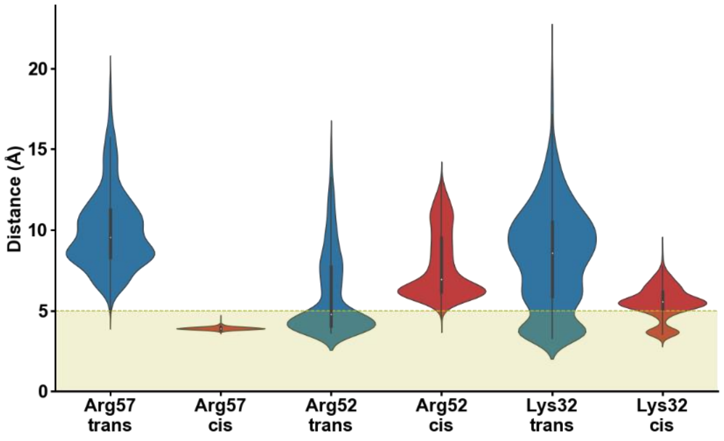

Figure S136. Violin plots of the distance between carboxylate and selected residues for *trans*- and *cis*-**15** throughout three replicas of 100 ns MD simulations.

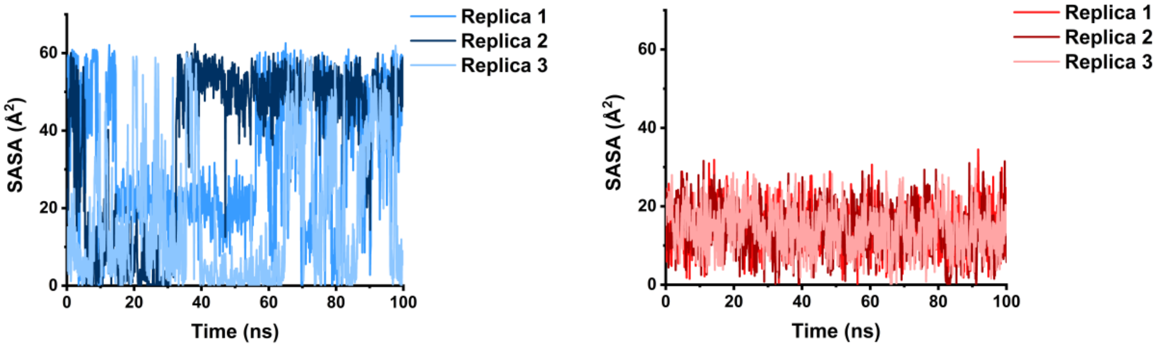

Figure S137. SASA of the chlorine substituent on the outer ring for (left) *trans*-**15** and (right) *cis*-**15** throughout three replicas of 100 ns MD simulations.

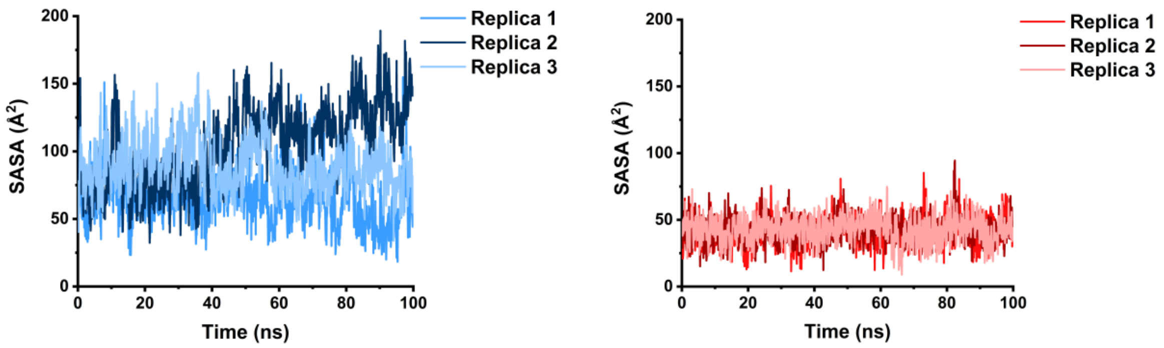

Figure S138. SASA of the lipophilic pocket for (left) *trans*-**15** and (right) *cis*-**15** throughout three replicas of 100 ns MD simulations.

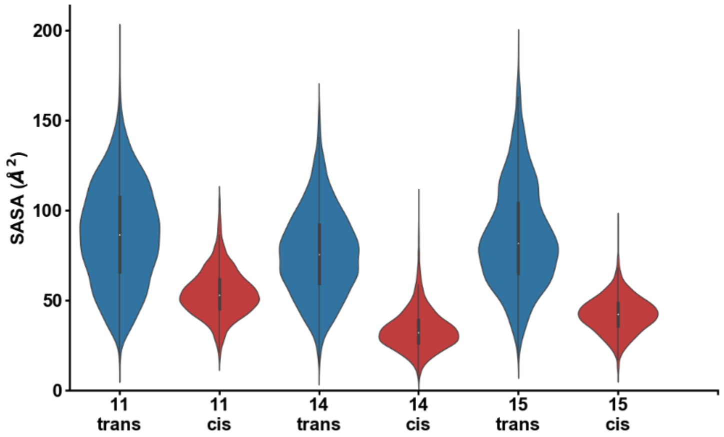

Figure S139. Violin plots of the SASA of the lipophilic pocket for inhibitors with a *p*-COOH substituent throughout three replicas of 100 ns MD simulations.

S5.3. Physicochemical descriptors

The data were retrieved from the ChEMBL database (as of August 2021) with two different target queries: “E. coli” for antibacterial activity (Target ID: CHEMBL354) and “eDHFR” for enzyme inhibition activity (Target ID: CHEMBL1809). The definitions of active subsets were adapted from published criteria.[8] The data were handled with KNIME and the properties were calculated with its RDKit node.

Table S5. Filters used to define antibacterial and biochemical compounds as active.[8]

Dataset	Standard Type	Standard Units	Standard Value
E. coli	‘MIC’	‘nM’	<= 25’000
E. coli	‘MIC’	‘ug.ml-1’ or ‘ug ml-1’	<= 8
E. coli	‘MIC50’	‘nM’	<= 25’000
E. coli	‘MIC50’	‘ug.ml-1’ or ‘ug ml-1’	<= 8
E. coli	‘MIC95’	‘uM’	<= 100
E. coli	‘MIC95’	‘ug.ml-1’ or ‘ug ml-1’	<= 32
eDHFR	‘Inhibition’	‘%’	70 <= x <= 100
eDHFR	‘IC50’	‘nM’	<= 25’000
eDHFR	‘IC50’	‘ug.ml-1’ or ‘ug ml-1’	<= 8
eDHFR	‘IC90’	‘nM’	<= 100’000
eDHFR	‘IC90’	‘ug.ml-1’ or ‘ug ml-1’	<= 32
eDHFR	‘Kd’	‘nM’	<= 25’000
eDHFR	‘Ki’	‘nM’	<= 25’000

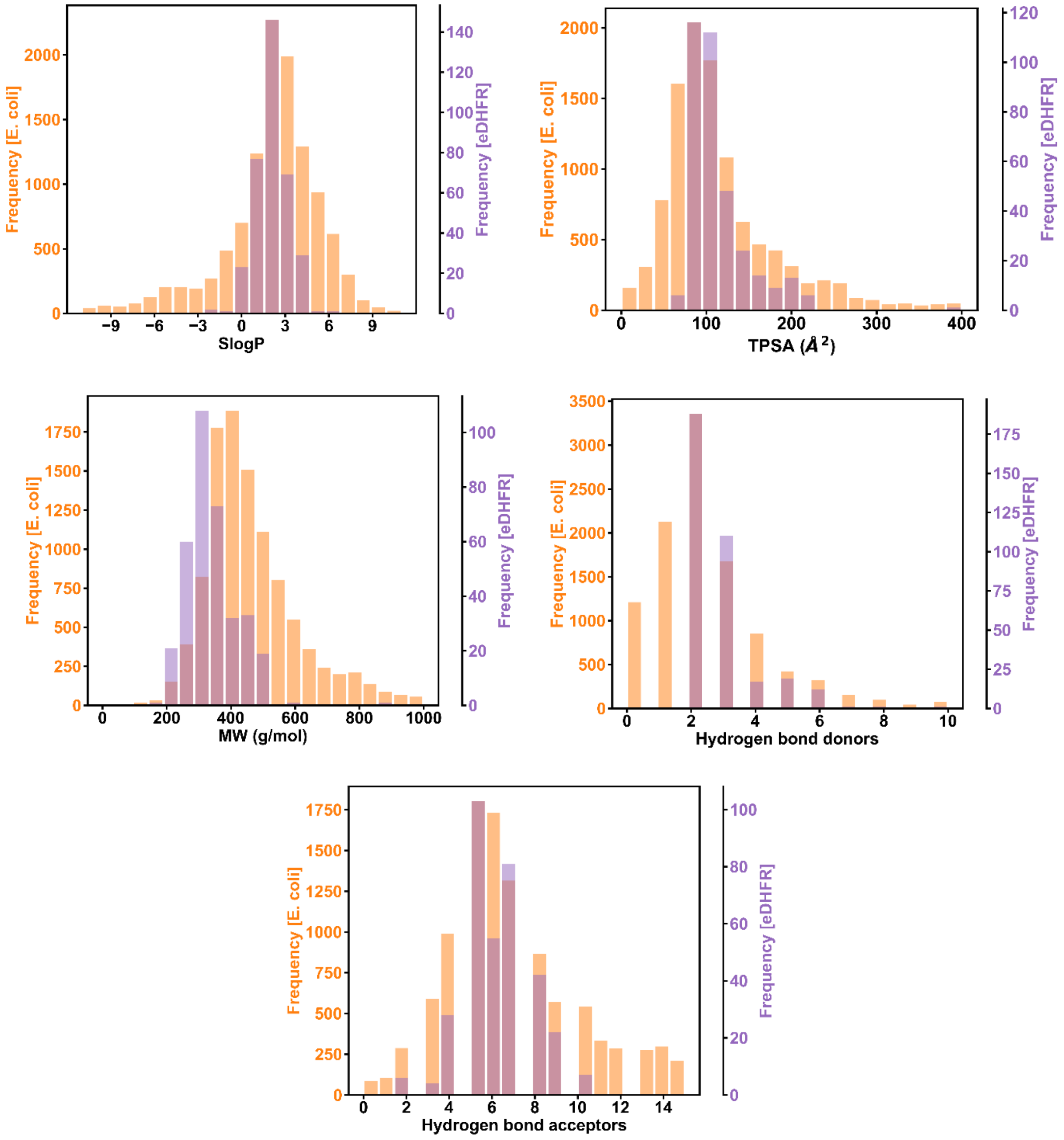

Figure S140. Distributions of RDKit descriptors calculated for compounds with antibacterial activity against *E.coli* (orange) and eDHFR inhibition activity (purple).

Table S6. RDKit descriptors calculated for the eDHFR inhibitors considered in this study.

**Compound**	**SlogP**	**TPSA**	**AMW**	**HBD**	**HBA**
TMP	0.68	106.76	291.33	2	6
Iclaprim	1.85	106.76	355.42	2	6
PMAT	4.67	140.71	501.57	2	9
TFAT	5.22	131.48	543.5	2	8
TCAT	7.28	131.48	609.32	2	8
PLA	1.82	128.43	416.48	2	6
1	1.48	103.79	215.24	2	5
2	3.14	103.79	291.34	2	5
3	3.14	103.79	291.34	2	5
4	2.88	103.79	315.36	2	5
5	2.88	103.79	315.36	2	5
6	3.39	113.02	373.44	2	6
7	3.39	113.02	373.44	2	6
8	3.4	122.25	403.47	2	7
9	2.74	130.84	405.49	2	8
10	1.75	153.15	416.44	2	8
11	1.75	153.15	416.44	2	8
12	2.06	153.15	430.47	2	8
13	2.41	153.15	450.89	2	8
14	2.06	153.15	430.47	2	8
15	2.41	153.15	450.89	2	8

S6. UHPLC-MS traces of compounds tested for biological activity

Compound 1

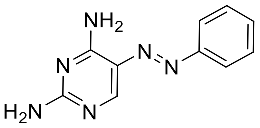

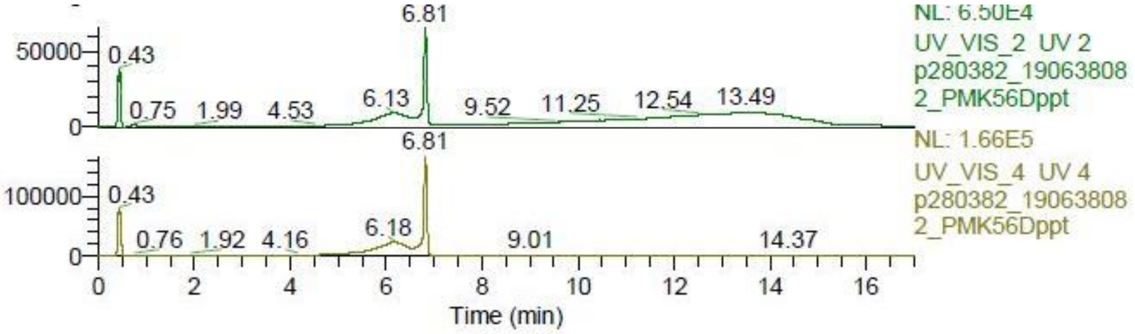

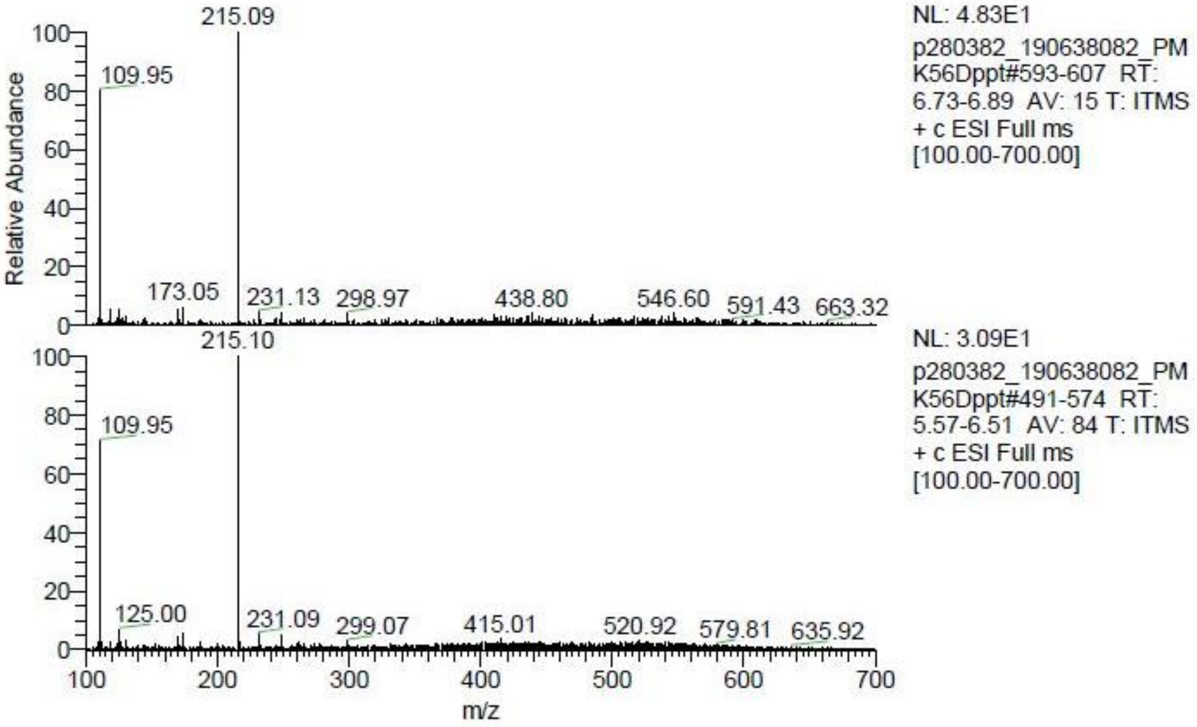

Compound 2

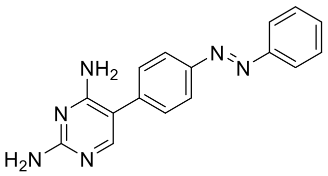
NH2
N
N
N
N
H2N

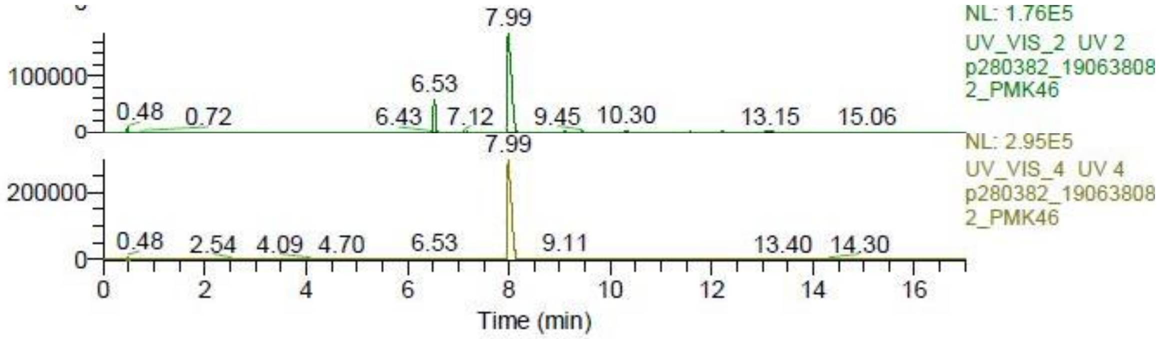
7.99
NL: 1.76E5
UV_VIS_2 UV 2
p280382_19063808
2_PMK46
100000
0
0.48 0.72
6.53
6.43 7.12
9.45 10.30
13.15 15.06
7.99
NL: 2.95E5
UV_VIS_4 UV 4
p280382_19063808
2_PMK46
200000
0
0.48 2.54 4.09 4.70 6.53 9.11 13.40 14.30
0 2 4 6 8 10 12 14 16
Time (min)

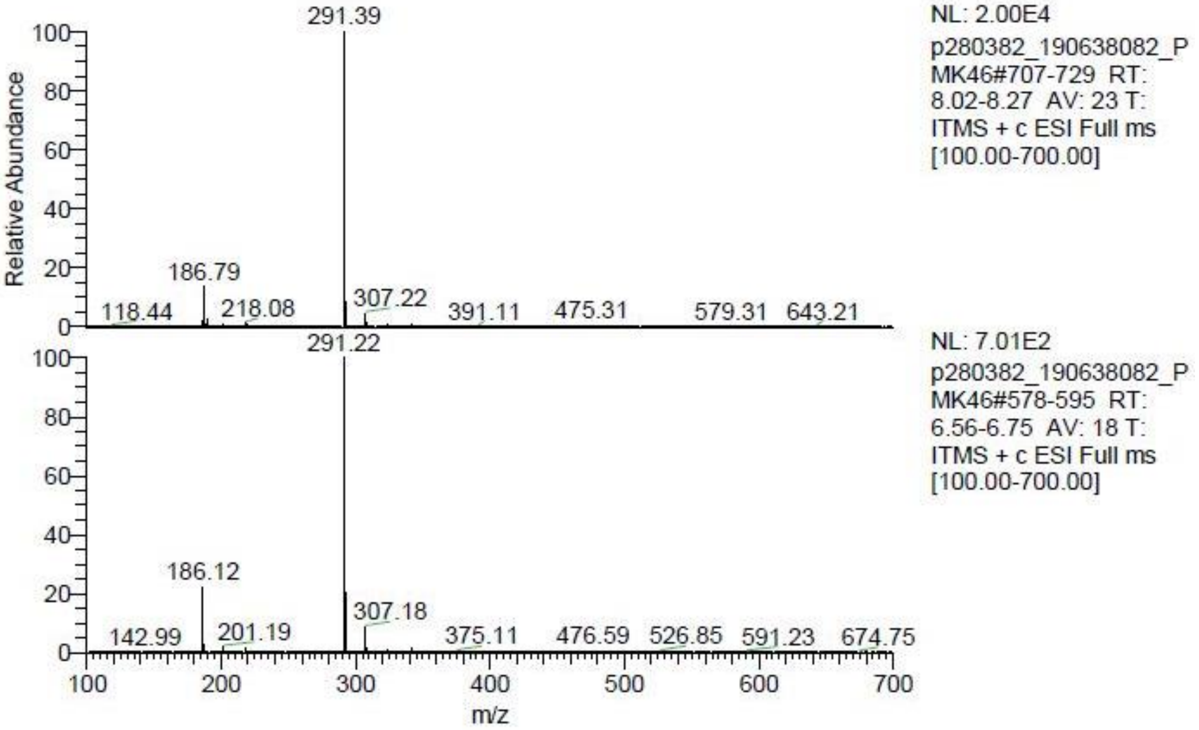
291.39
NL: 2.00E4
p280382_190638082_P
MK46#707-729 RT:
8.02-8.27 AV: 23 T:
ITMS + c ESI Full ms
[100.00-700.00]
Relative Abundance
100
80
60
40
20
0
186.79
118.44 218.08 307.22 391.11 475.31 579.31 643.21
291.22
NL: 7.01E2
p280382_190638082_P
MK46#578-595 RT:
6.56-6.75 AV: 18 T:
ITMS + c ESI Full ms
[100.00-700.00]
186.12
142.99 201.19 307.18 375.11 476.59 526.85 591.23 674.75
100 200 300 400 500 600 700
m/z

Compound 3

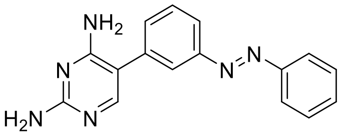

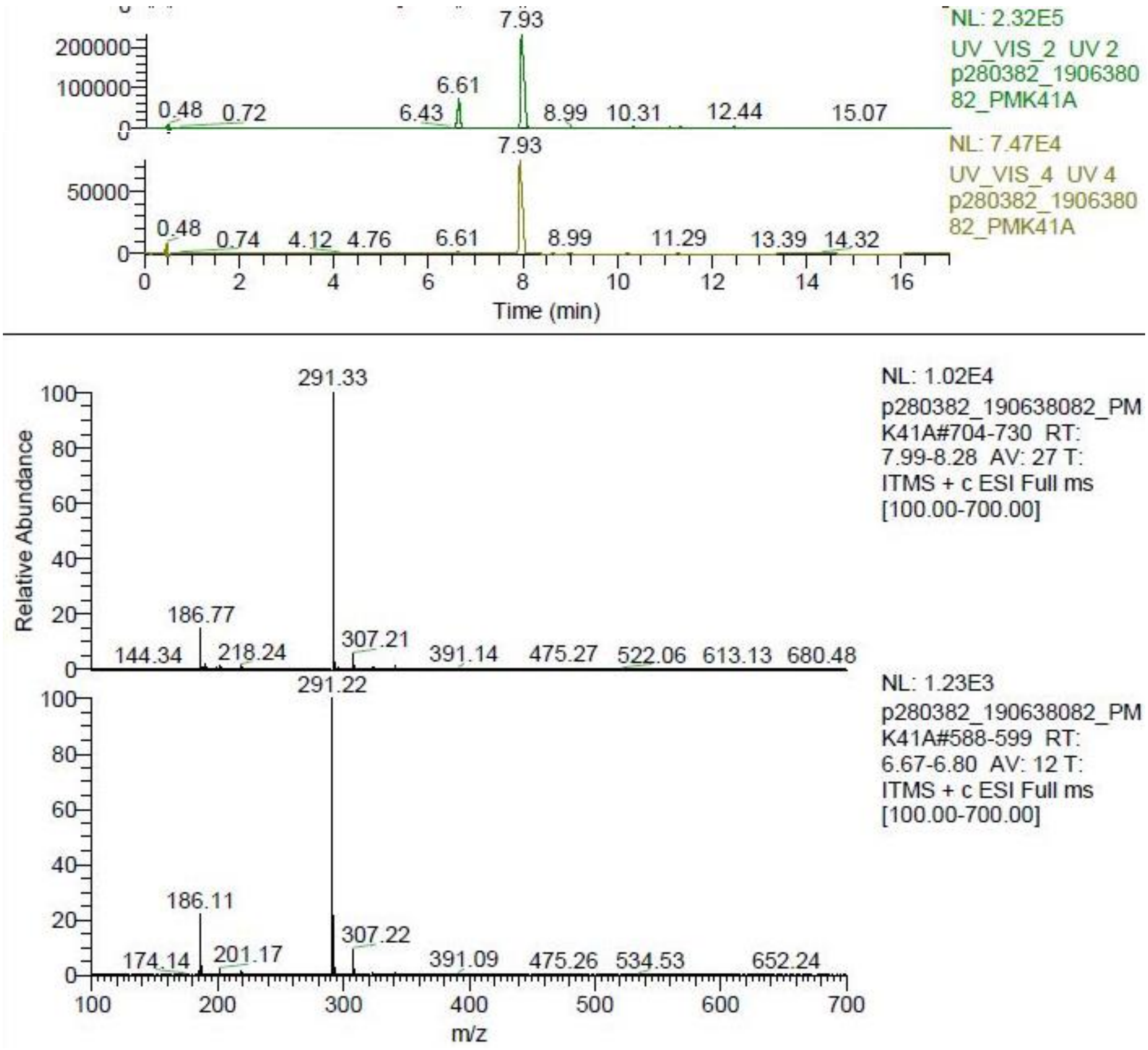

7.93
NL: 2.32E5
UV_VIS_2 UV 2
p280382_1906380
82_PMK41A
200000
100000
0
0.48
0.72
6.43
6.61
8.99
10.31
12.44
15.07
7.93
NL: 7.47E4
UV_VIS_4 UV 4
p280382_1906380
82_PMK41A
50000
0.48
0.74
4.12
4.76
6.61
8.99
11.29
13.39
14.32
0 2 4 6 8 10 12 14 16
Time (min)
291.33
NL: 1.02E4
p280382_190638082_PM
K41A#704-730 RT:
7.99-8.28 AV: 27 T:
ITMS + c ESI Full ms
[100.00-700.00]
Relative Abundance
186.77
144.34
218.24
307.21
391.14
475.27
522.06
613.13
680.48
291.22
NL: 1.23E3
p280382_190638082_PM
K41A#588-599 RT:
6.67-6.80 AV: 12 T:
ITMS + c ESI Full ms
[100.00-700.00]
186.11
307.22
174.14
201.17
391.09
475.26
534.53
652.24
100 200 300 400 500 600 700
m/z

Compound 4

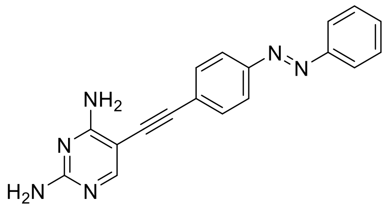
NH2
N
N
H2N
N
N

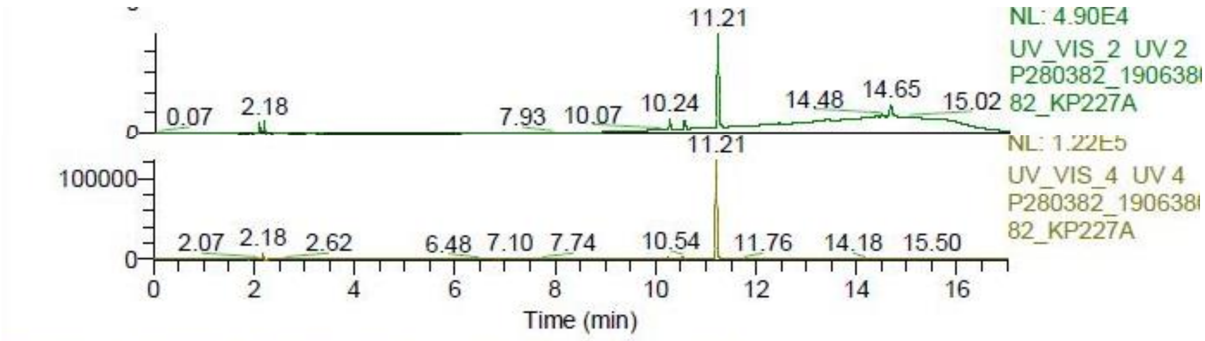
11.21
NL: 4.90E4
UV_VIS_2 UV 2
P280382_190638082_KP227A
0.07
2.18
7.93
10.07
10.24
14.48
14.65
15.02
11.21
NL: 1.22E5
UV_VIS_4 UV 4
P280382_190638082_KP227A
100000
2.07
2.18
2.62
6.48
7.10
7.74
10.54
11.76
14.18
15.50
0
2
4
6
8
10
12
14
16
Time (min)

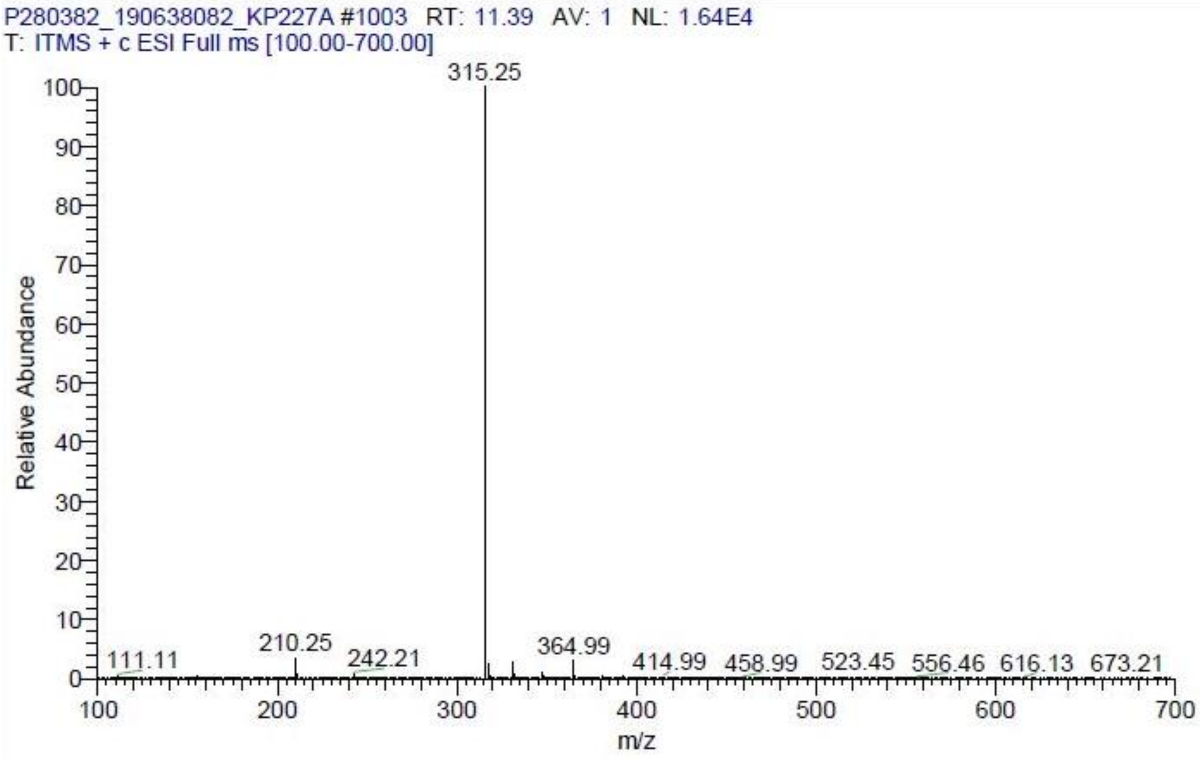
P280382_190638082_KP227A #1003 RT: 11.39 AV: 1 NL: 1.64E4
T: ITMS + c ESI Full ms [100.00-700.00]
315.25
Relative Abundance
111.11
210.25
242.21
364.99
414.99
458.99
523.45
556.46
616.13
673.21
m/z

Compound 5

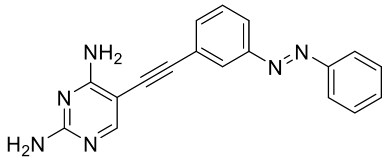
NH2
N
H2N
N
N
N

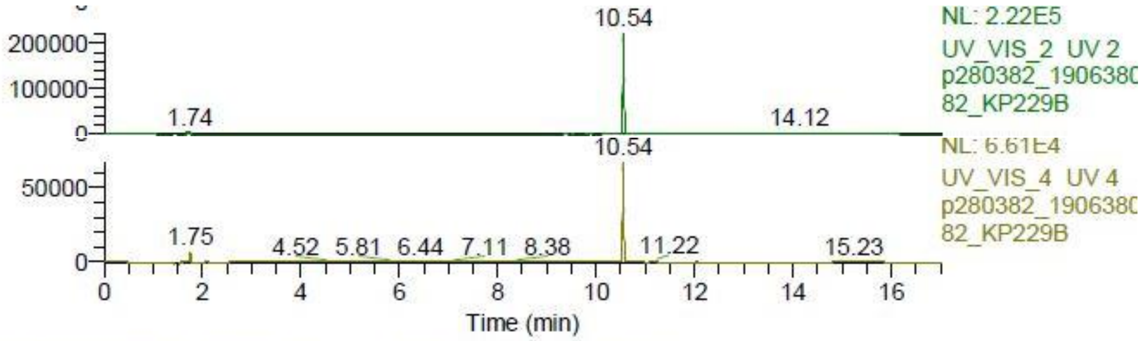
10.54
NL: 2.22E5
UV_VIS_2 UV 2
p280382_1906380
82_KP229B
200000
100000
0
1.74
14.12
10.54
NL: 6.61E4
UV_VIS_4 UV 4
p280382_1906380
82_KP229B
50000
0
1.75
4.52
5.81
6.44
7.11
8.38
11.22
15.23
0
2
4
6
8
10
12
14
16
Time (min)

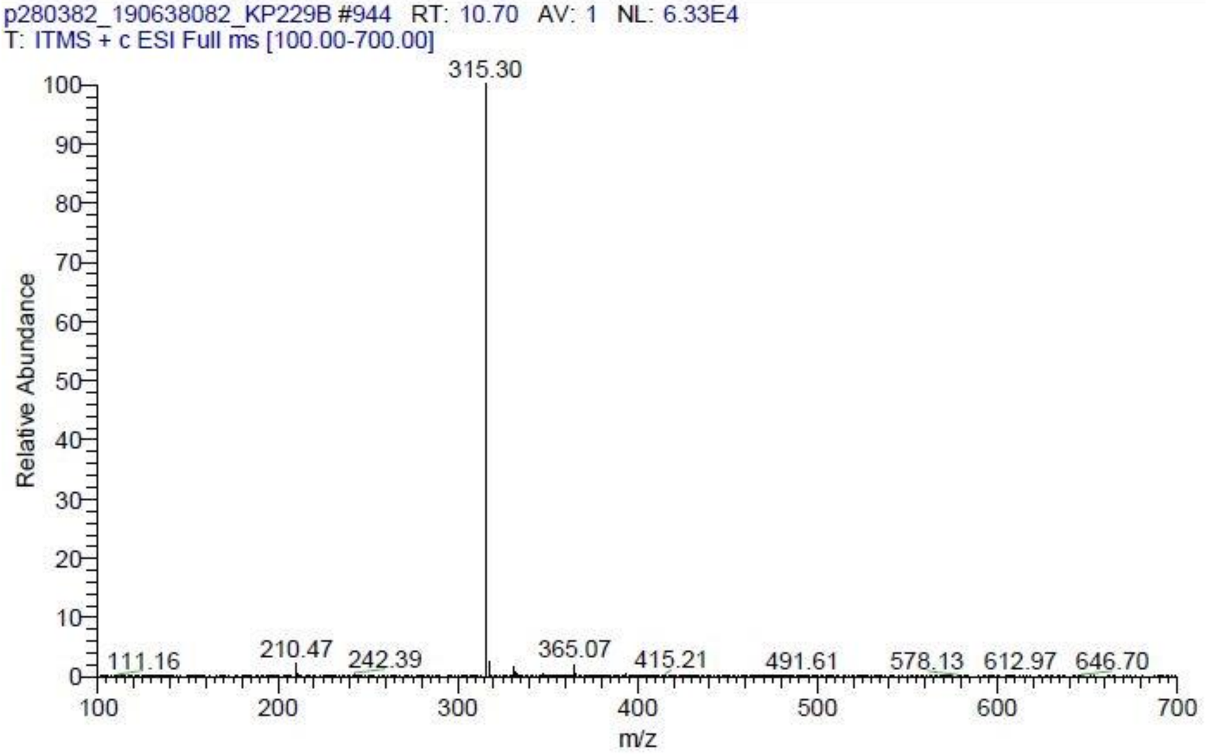
p280382_190638082_KP229B #944 RT: 10.70 AV: 1 NL: 6.33E4
T: ITMS + c ESI Full ms [100.00-700.00]
315.30
Relative Abundance
100
90
80
70
60
50
40
30
20
10
0
111.16
210.47
242.39
365.07
415.21
491.61
578.13
612.97
646.70
100
200
300
400
500
600
700
m/z

Compound 6

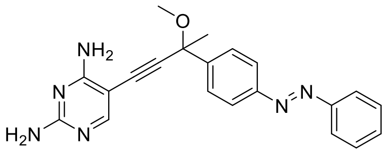

O
NH2
N
N
H2N
N
N
N

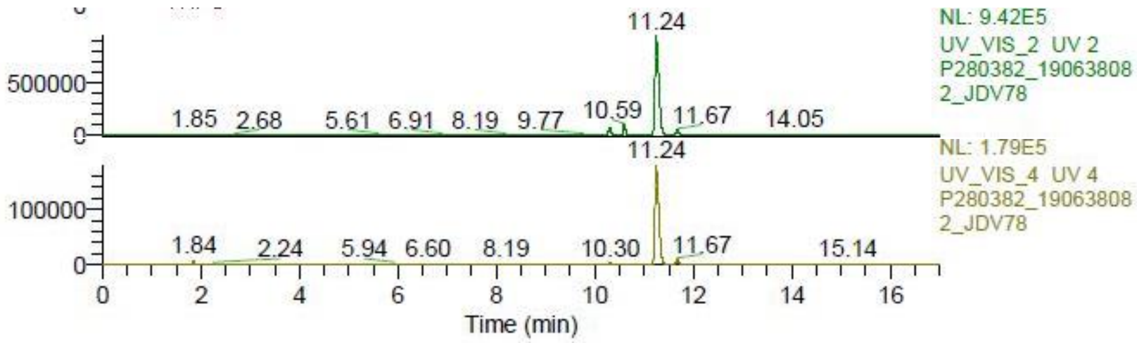

11.24
NL: 9.42E5
UV_VIS_2 UV 2
P280382_19063808
2_JDV78
500000
0
1.85
2.68
5.61
6.91
8.19
9.77
10.59
11.67
14.05
11.24
NL: 1.79E5
UV_VIS_4 UV 4
P280382_19063808
2_JDV78
100000
0
1.84
2.24
5.94
6.60
8.19
10.30
11.67
15.14
0 2 4 6 8 10 12 14 16
Time (min)

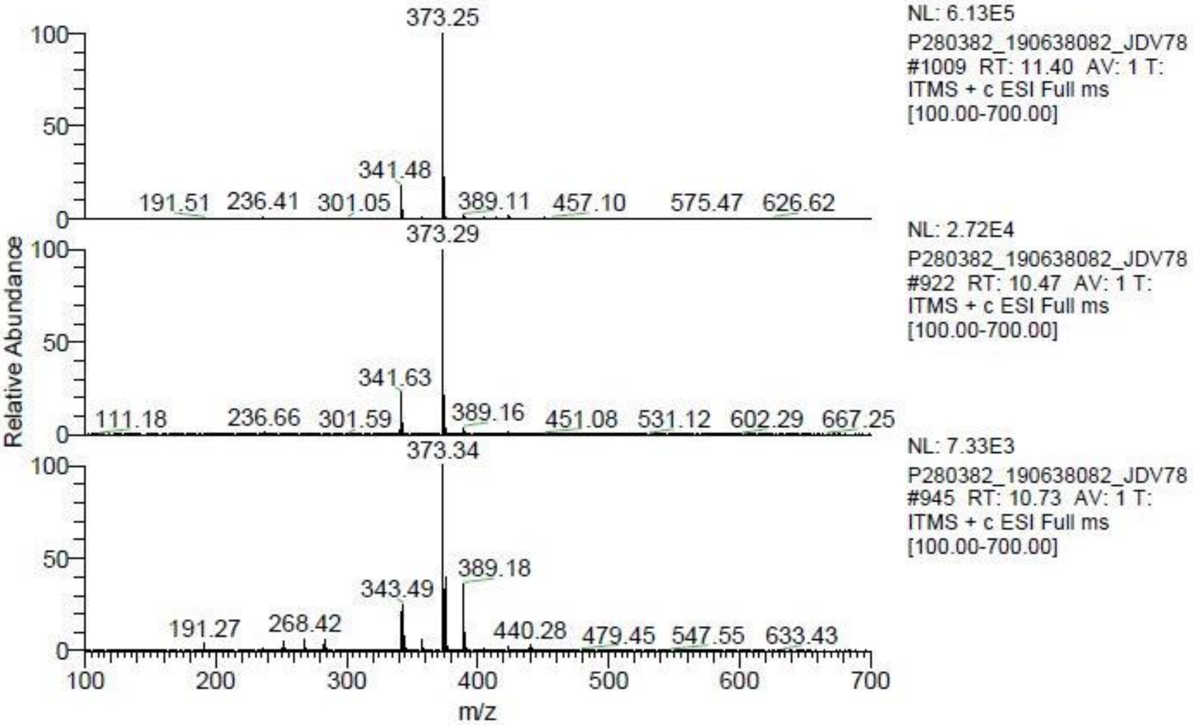

373.25
NL: 6.13E5
P280382_190638082_JDV78
#1009 RT: 11.40 AV: 1 T:
ITMS + c ESI Full ms
[100.00-700.00]
100
50
0
341.48
191.51
236.41
301.05
389.11
457.10
575.47
626.62
373.29
NL: 2.72E4
P280382_190638082_JDV78
#922 RT: 10.47 AV: 1 T:
ITMS + c ESI Full ms
[100.00-700.00]
Relative Abundance
341.63
111.18
236.66
301.59
389.16
451.08
531.12
602.29
667.25
373.34
NL: 7.33E3
P280382_190638082_JDV78
#945 RT: 10.73 AV: 1 T:
ITMS + c ESI Full ms
[100.00-700.00]
389.18
343.49
191.27
268.42
440.28
479.45
547.55
633.43
100 200 300 400 500 600 700
m/z

Compound 7

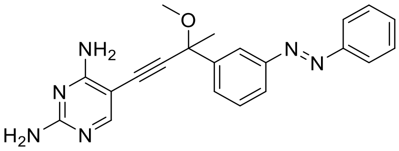

NH2
N
N
H2N
O
N
N

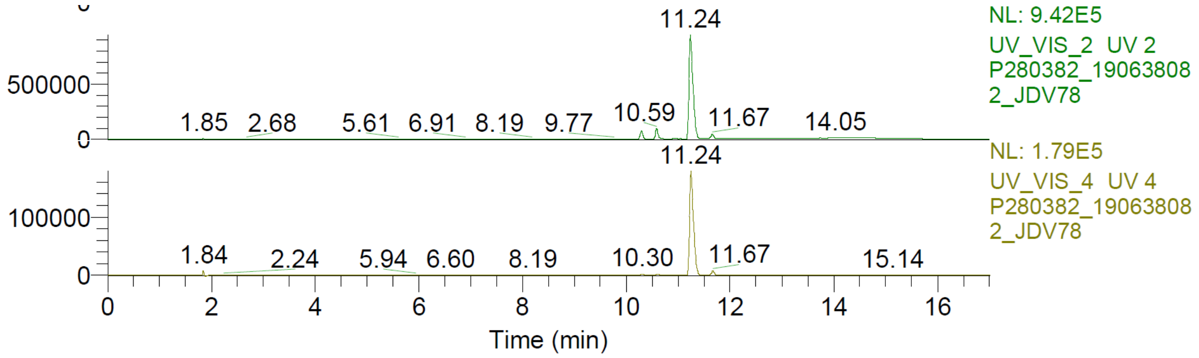

11.24
NL: 9.42E5
UV_VIS_2 UV 2
P280382_19063808
2_JDV78
500000
0
1.85 2.68 5.61 6.91 8.19 9.77 10.59 11.67 14.05
11.24
NL: 1.79E5
UV_VIS_4 UV 4
P280382_19063808
2_JDV78
100000
0
1.84 2.24 5.94 6.60 8.19 10.30 11.67 15.14
0 2 4 6 8 10 12 14 16
Time (min)

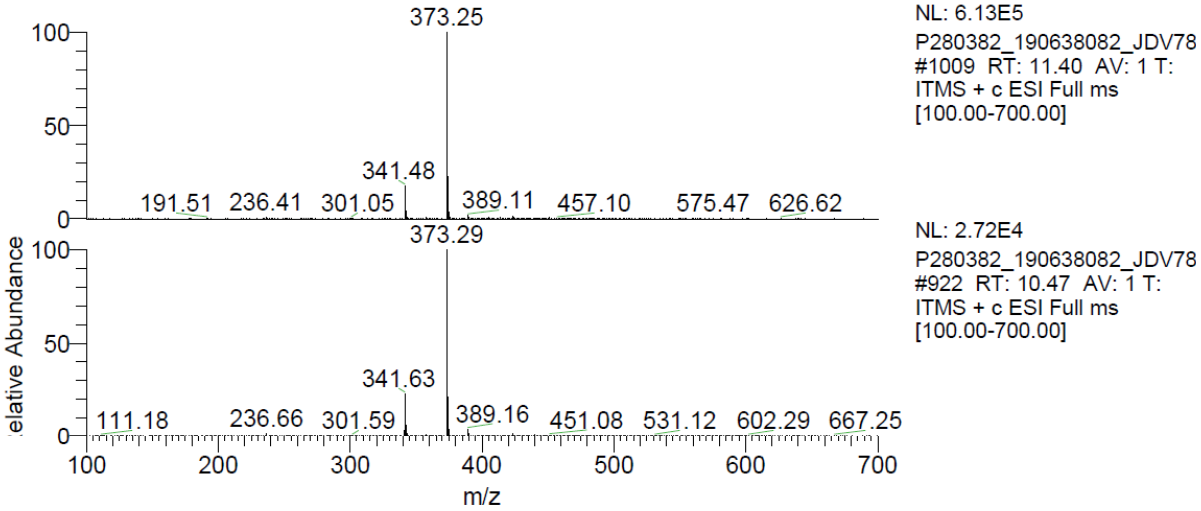

373.25
NL: 6.13E5
P280382_190638082_JDV78
#1009 RT: 11.40 AV: 1 T:
ITMS + c ESI Full ms
[100.00-700.00]
100
50
0
341.48
191.51 236.41 301.05 389.11 457.10 575.47 626.62
373.29
NL: 2.72E4
P280382_190638082_JDV78
#922 RT: 10.47 AV: 1 T:
ITMS + c ESI Full ms
[100.00-700.00]
elative Abundance
341.63
111.18 236.66 301.59 389.16 451.08 531.12 602.29 667.25
100 200 300 400 500 600 700
m/z

Compound 8

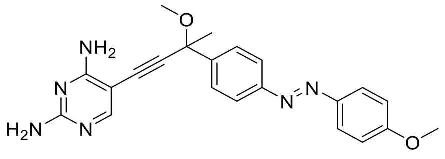

NH2
N
H2N
N
O
N
N
O

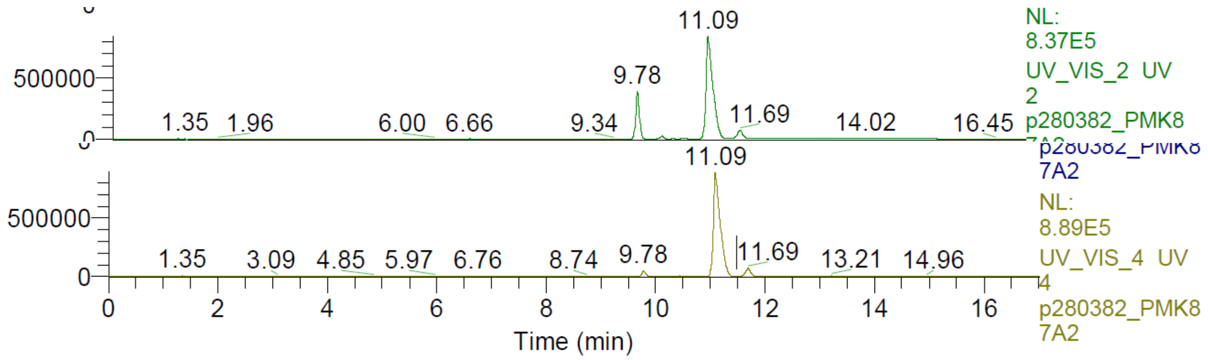

11.09
9.78
1.35
1.96
6.00
6.66
9.34
11.69
14.02
16.45
500000
NL: 8.37E5
UV_VIS_2 UV 2
p280382_PMK87A2
11.09
1.35
3.09
4.85
5.97
6.76
8.74
9.78
11.69
13.21
14.96
500000
0
NL: 8.89E5
UV_VIS_4 UV 4
p280382_PMK87A2
0 2 4 6 8 10 12 14 16
Time (min)

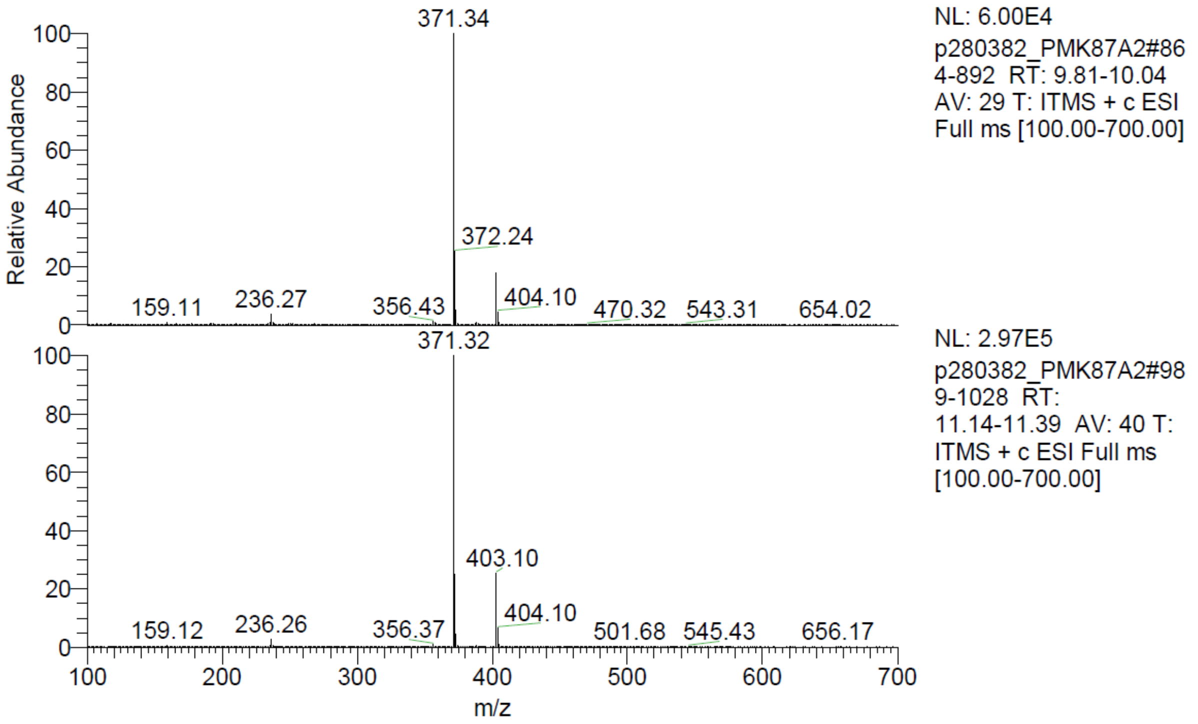

371.34
372.24
159.11
236.27
356.43
404.10
470.32
543.31
654.02
NL: 6.00E4
p280382_PMK87A2#864-892 RT: 9.81-10.04
AV: 29 T: ITMS + c ESI Full ms [100.00-700.00]
Relative Abundance
371.32
403.10
159.12
236.26
356.37
404.10
501.68
545.43
656.17
NL: 2.97E5
p280382_PMK87A2#989-1028 RT: 11.14-11.39 AV: 40 T: ITMS + c ESI Full ms [100.00-700.00]
100 200 300 400 500 600 700
m/z

Compound 9

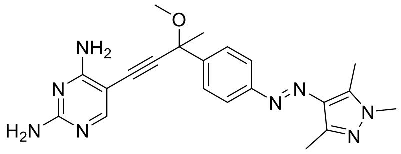
NH2
N
N
H2N
O
N
N
N
N

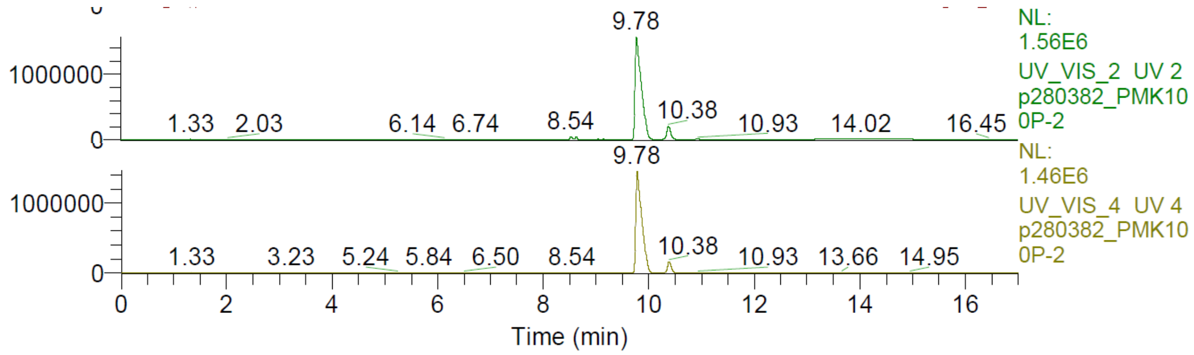
9.78
1000000
0
1.33
2.03
6.14
6.74
8.54
10.38
10.93
14.02
16.45
NL:
1.56E6
UV_VIS_2 UV 2
p280382_PMK100P-2
9.78
3.23
5.24
5.84
6.50
13.66
14.95
NL:
1.46E6
UV_VIS_4 UV 4
p280382_PMK100P-2
Time (min)

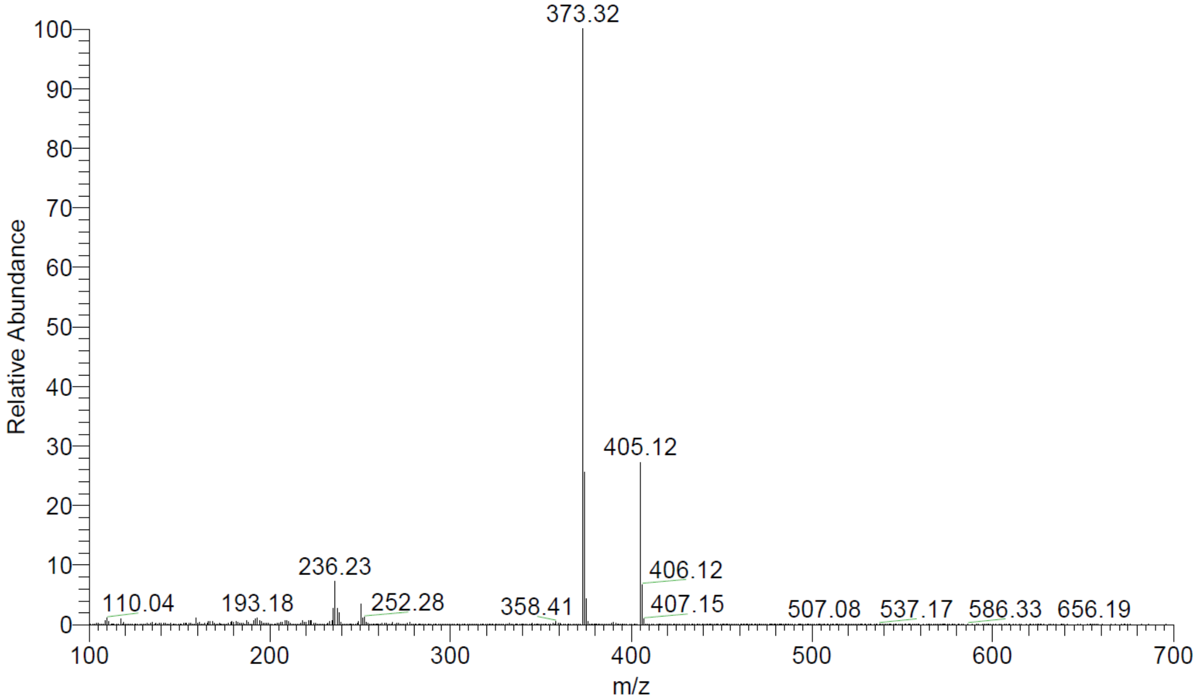
373.32
405.12
236.23
406.12
110.04
193.18
252.28
358.41
407.15
507.08
537.17
586.33
656.19
Relative Abundance
m/z

Compound 10

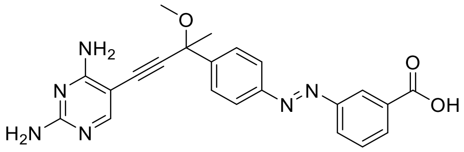
NH2
H2N
N
N
O
N
N
O
OH

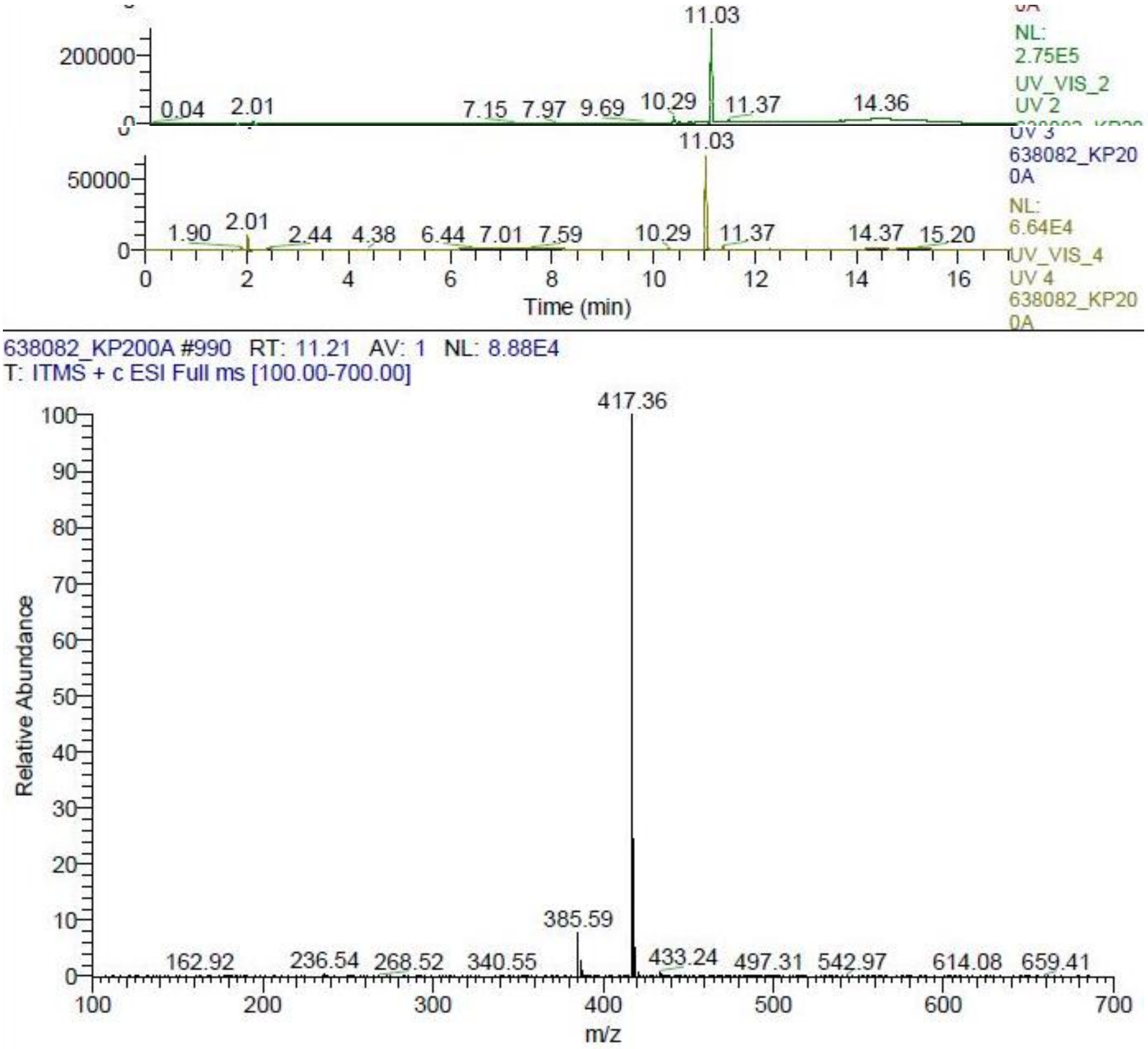
11.03
NL:
2.75E5
UV_VIS_2
UV 2
200000
0.04
2.01
7.15
7.97
9.69
10.29
11.37
14.36
UV 3
638082_KP200A
NL:
6.64E4
UV_VIS_4
UV 4
638082_KP200A
50000
1.90
2.44
4.38
6.44
7.01
7.59
14.37
15.20
Time (min)
638082_KP200A #990 RT: 11.21 AV: 1 NL: 8.88E4
T: ITMS + c ESI Full ms [100.00-700.00]
417.36
385.59
162.92
236.54
268.52
340.55
433.24
497.31
542.97
614.08
659.41
Relative Abundance
m/z

Compound 11

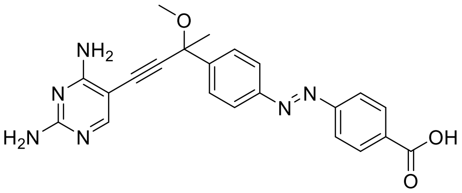

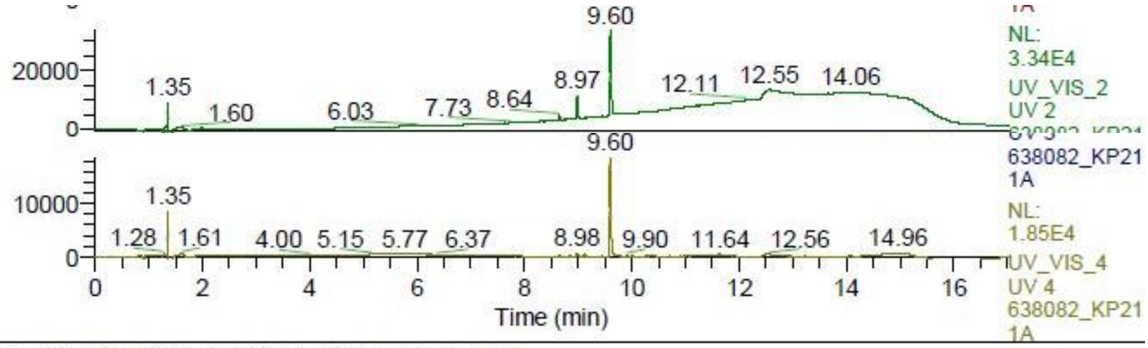

9.60
20000
1.35
1.60
6.03
7.73
8.64
8.97
12.11
12.55
14.06
NL:
3.34E4
UV_VIS_2
UV 2
9.60
10000
1.35
1.28
1.61
4.00
5.15
5.77
6.37
8.98
9.90
11.64
12.56
14.96
638082_KP21
1A
NL:
1.85E4
UV_VIS_4
UV 4
638082_KP21
1A
Time (min)

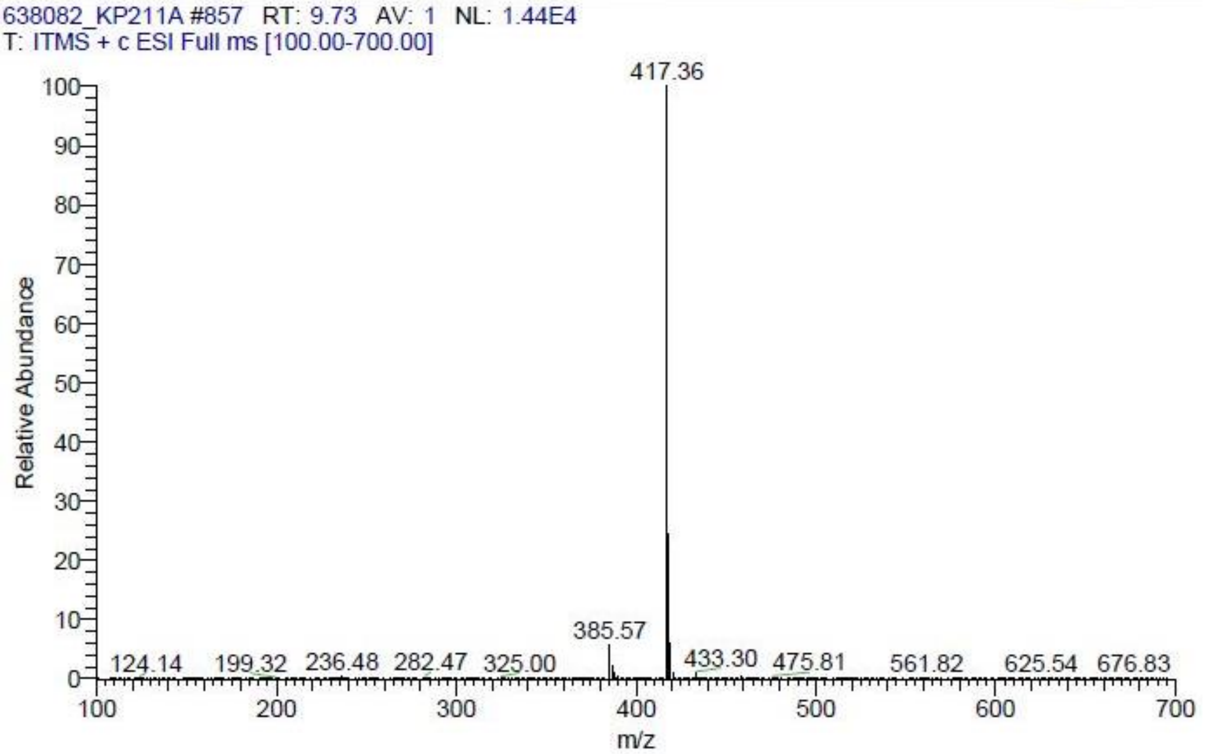

638082_KP211A #857 RT: 9.73 AV: 1 NL: 1.44E4
T: ITMS + c ESI Full ms [100.00-700.00]
417.36
385.57
124.14
199.32
236.48
282.47
325.00
433.30
475.81
561.82
625.54
676.83
Relative Abundance
m/z

Compound 12

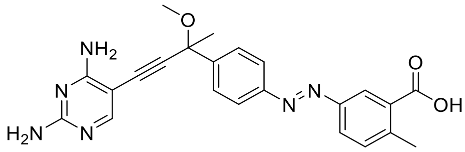

NH2
N
H2N
N
O
N
N
O
OH

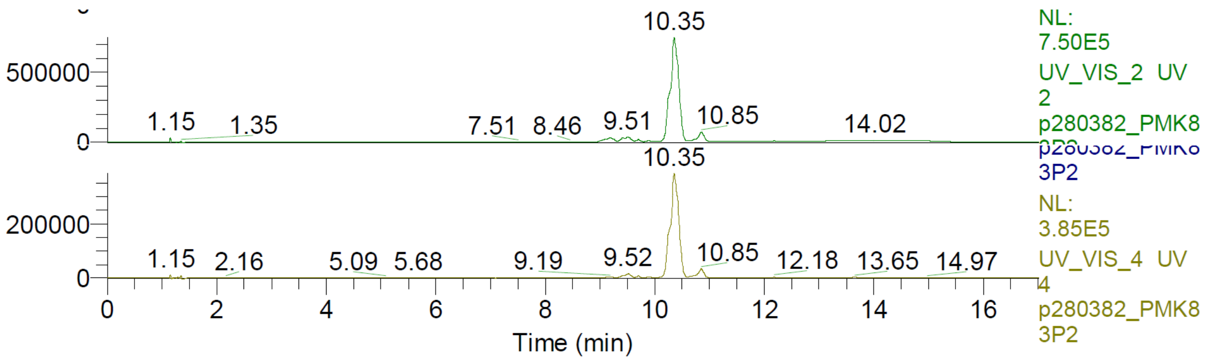

10.35
NL:
7.50E5
UV_VIS_2 UV
2
p280382_PMK8
500000
0
1.15
1.35
7.51
8.46
9.51
10.85
14.02
3P2
NL:
3.85E5
UV_VIS_4 UV
4
p280382_PMK8
3P2
200000
1.15
2.16
5.09
5.68
9.19
9.52
10.85
12.18
13.65
14.97
0
2
4
6
8
10
12
14
16
Time (min)

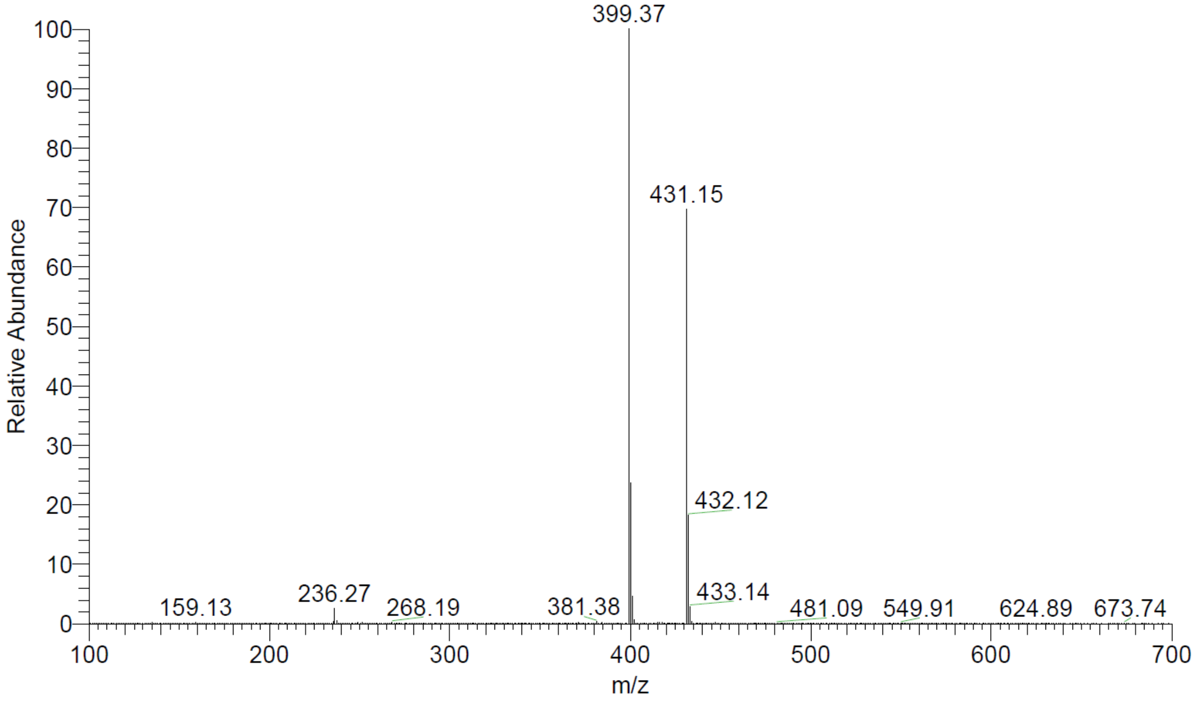

399.37
431.15
432.12
433.14
159.13
236.27
268.19
381.38
481.09
549.91
624.89
673.74
Relative Abundance
100
90
80
70
60
50
40
30
20
10
0
100
200
300
400
500
600
700
m/z

Compound 13

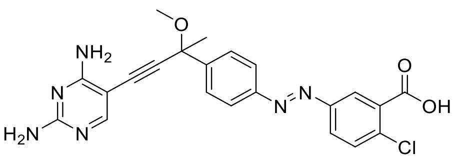
NH2
N
H2N
N
O
N
N
O
OH
Cl

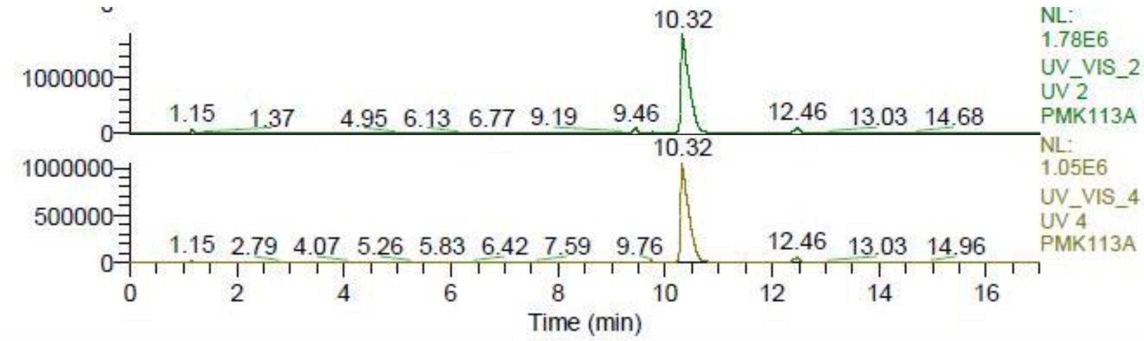
10.32
NL:
1.78E6
UV_VIS_2
UV 2
PMK113A
1000000
0
1.15 1.37 4.95 6.13 6.77 9.19 9.46
12.46 13.03 14.68
10.32
NL:
1.05E6
UV_VIS_4
UV 4
PMK113A
1000000
500000
0
1.15 2.79 4.07 5.26 5.83 6.42 7.59 9.76
12.46 13.03 14.96
0 2 4 6 8 10 12 14 16
Time (min)

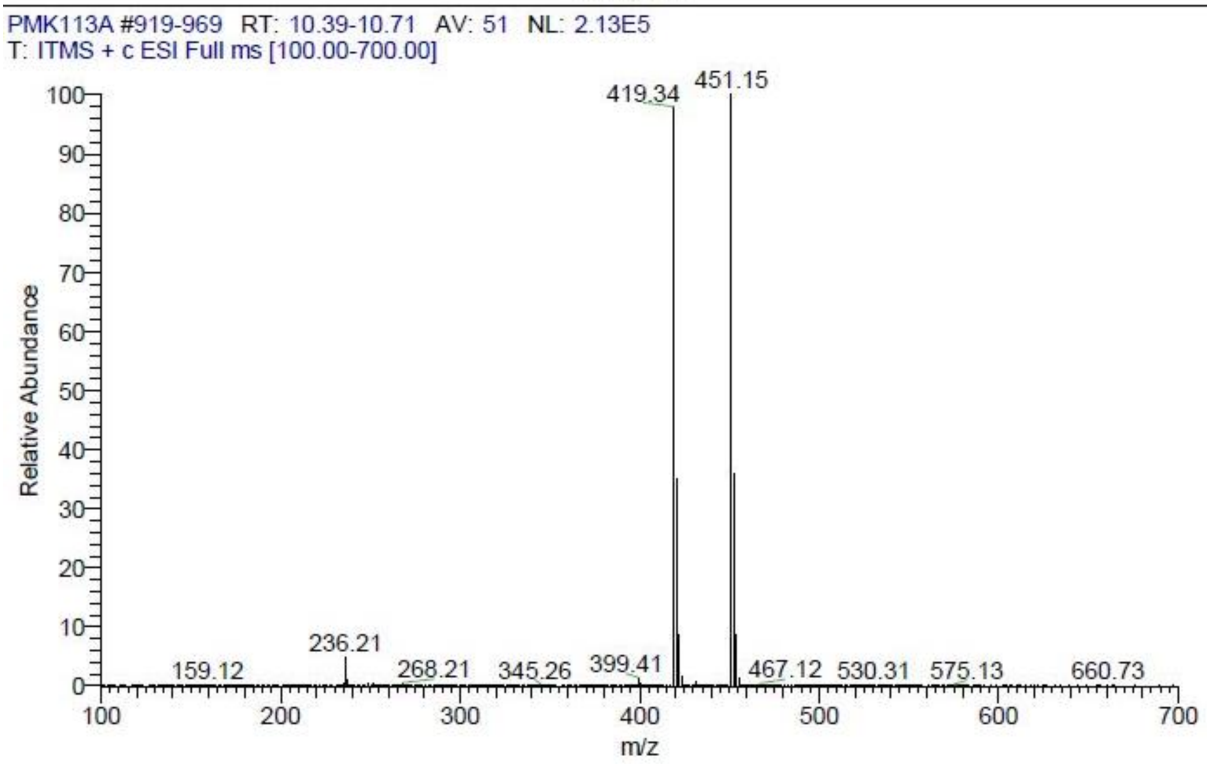
PMK113A #919-969 RT: 10.39-10.71 AV: 51 NL: 2.13E5
T: ITMS + c ESI Full ms [100.00-700.00]
419.34
451.15
236.21
159.12 268.21 345.26 399.41
467.12 530.31 575.13 660.73
Relative Abundance
100 90 80 70 60 50 40 30 20 10 0
100 200 300 400 500 600 700
m/z

Compound 14

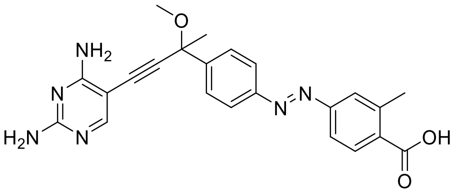

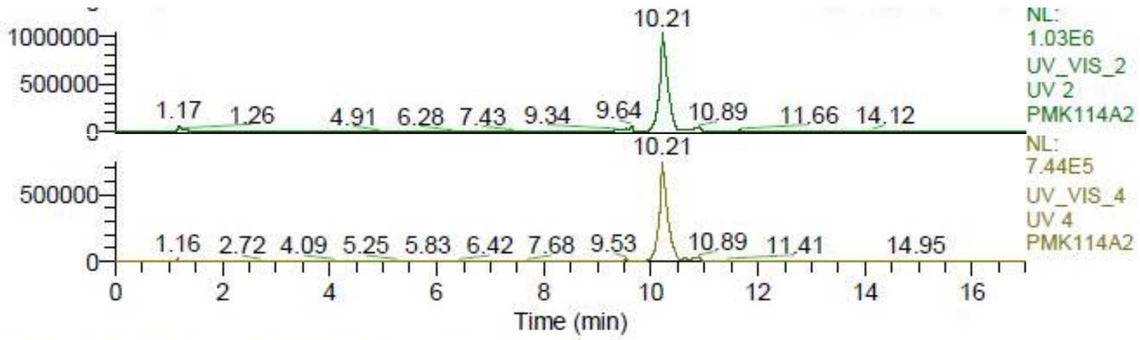
10.21
NL: 1.03E6
UV_VIS_2
UV 2
PMK114A2
1.17 1.26 4.91 6.28 7.43 9.34 9.64 10.89 11.66 14.12
NL: 7.44E5
UV_VIS_4
UV 4
PMK114A2
1.16 2.72 4.09 5.25 5.83 6.42 7.68 9.53 10.89 11.41 14.95
Time (min)

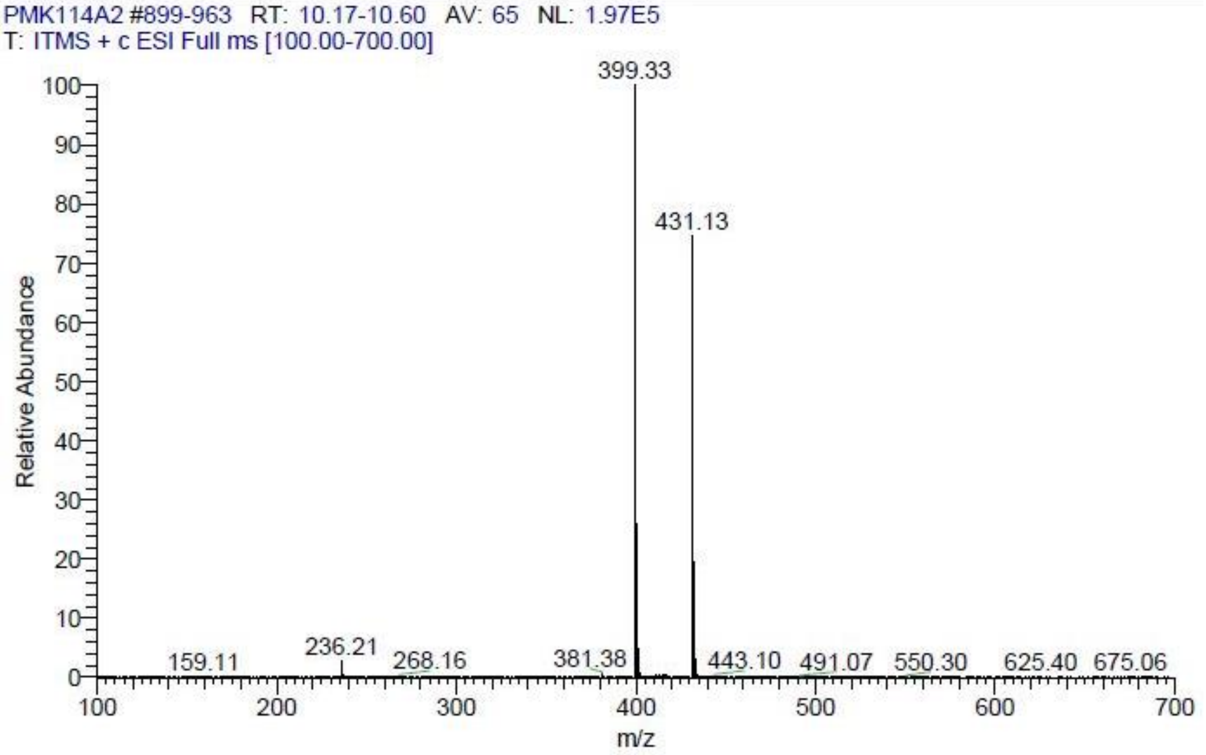
PMK114A2 #899-963 RT: 10.17-10.60 AV: 65 NL: 1.97E5
T: ITMS + c ESI Full ms [100.00-700.00]
399.33
431.13
159.11 236.21 268.16 381.38 443.10 491.07 550.30 625.40 675.06
Relative Abundance
m/z

Compound 15

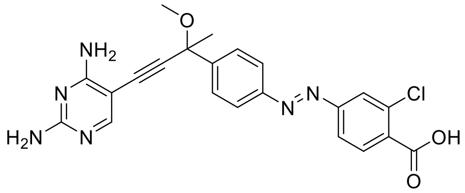

O
NH2
N
H2N
N
N
N
Cl
OH
O

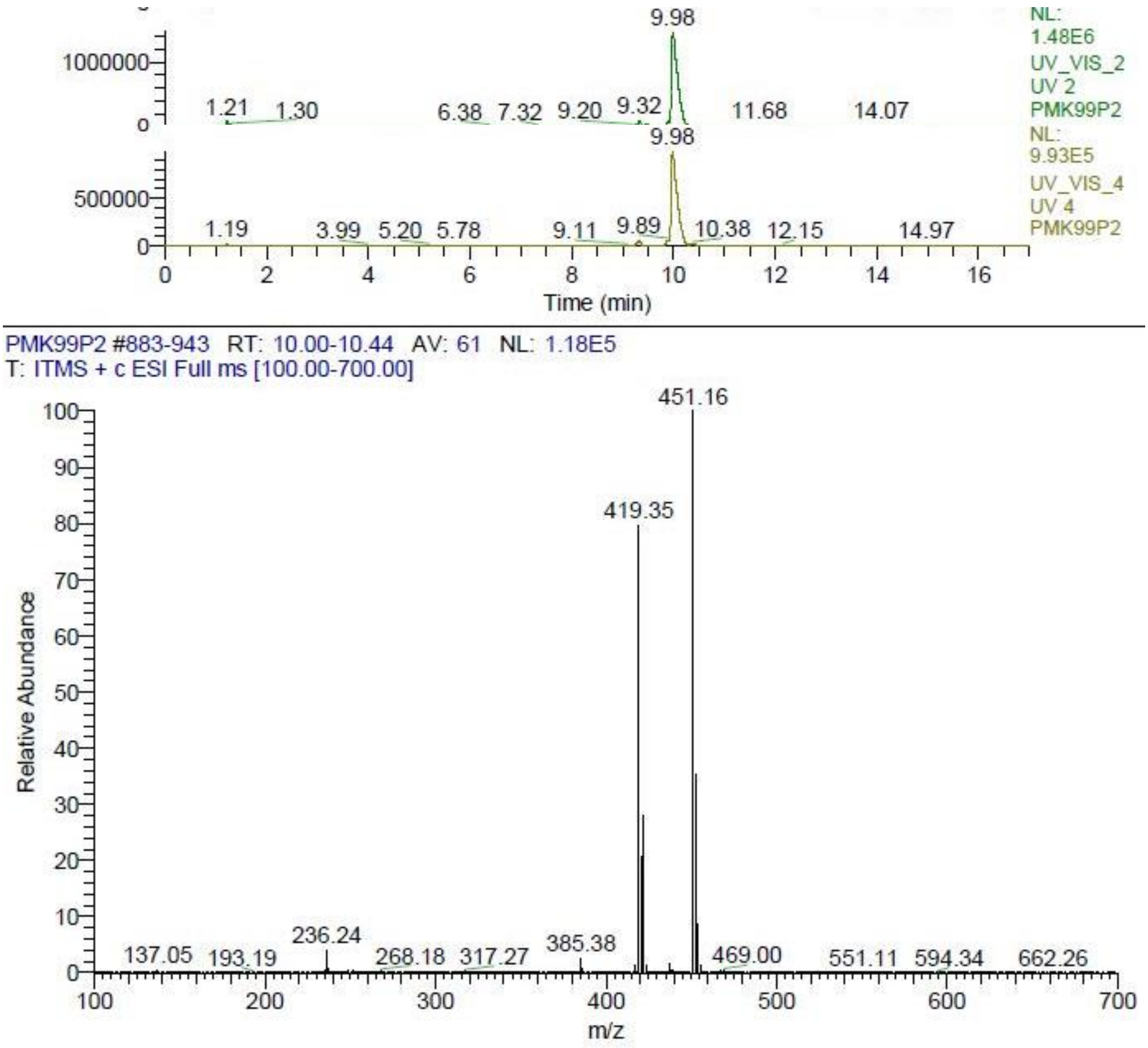

9.98
NL:
1.48E6
UV_VIS_2
UV 2
PMK99P2
1000000
0
1.21
1.30
6.38
7.32
9.20
9.32
11.68
14.07
9.98
NL:
9.93E5
UV_VIS_4
UV 4
PMK99P2
500000
1.19
3.99
5.20
5.78
9.11
9.89
10.38
12.15
14.97
0 2 4 6 8 10 12 14 16
Time (min)
PMK99P2 #883-943 RT: 10.00-10.44 AV: 61 NL: 1.18E5
T: ITMS + c ESI Full ms [100.00-700.00]
451.16
419.35
236.24
137.05
193.19
268.18
317.27
385.38
469.00
551.11
594.34
662.26
Relative Abundance
0 10 20 30 40 50 60 70 80 90 100
100 200 300 400 500 600 700
m/z

S7. NMR and HRMS data

Compound S1 (^{1}H NMR, 400 MHz, DMSO-d_6)

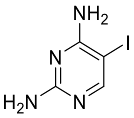

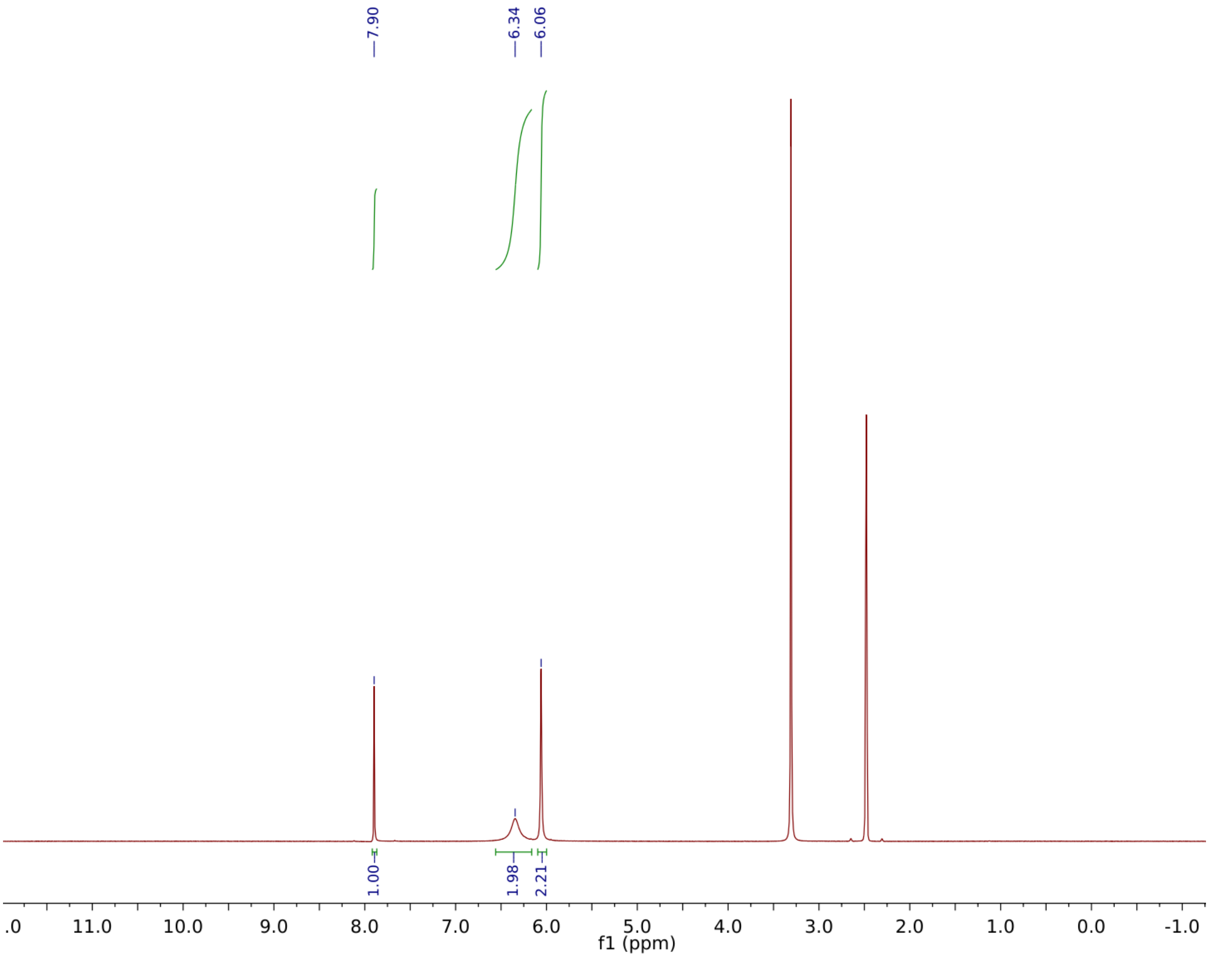

S7.1. First hypothesis

Compound 1 (^{1}H NMR, 400 MHz, DMSO-d_6)

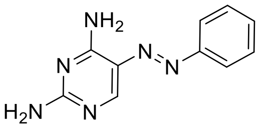

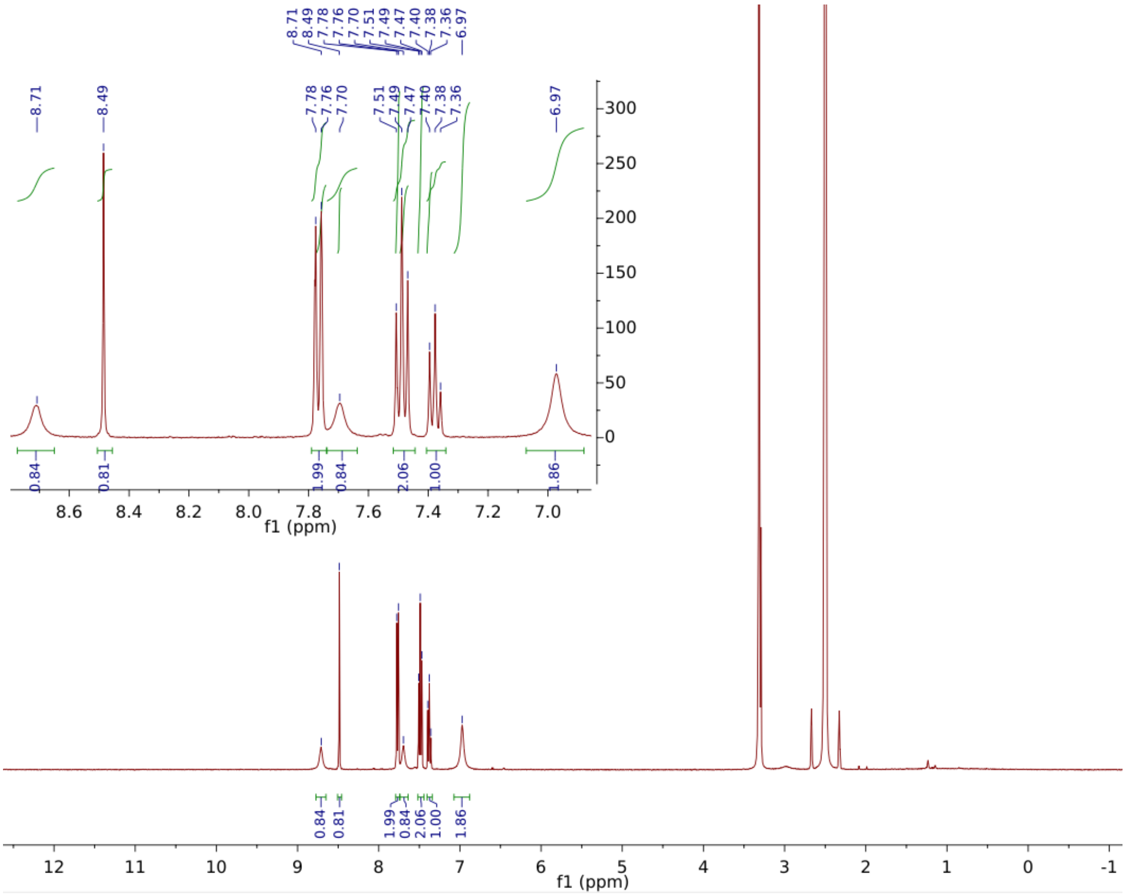

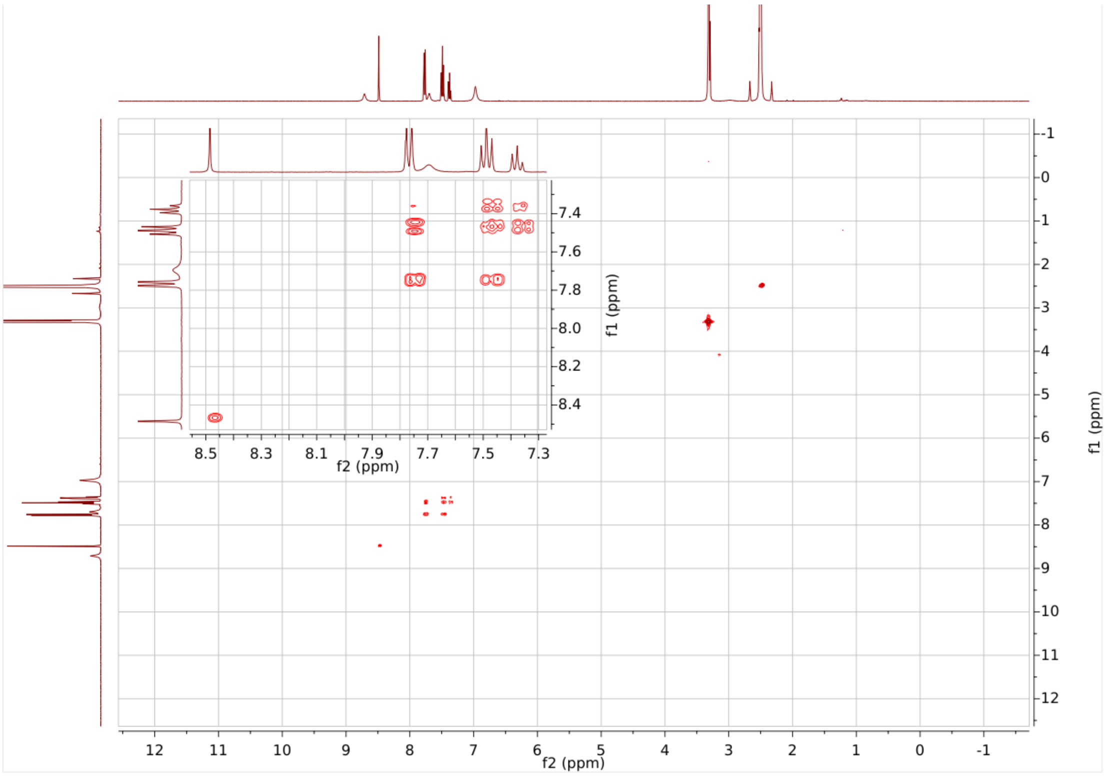

Compound 1 (^{13}C NMR, 101 MHz, DMSO-d_6)

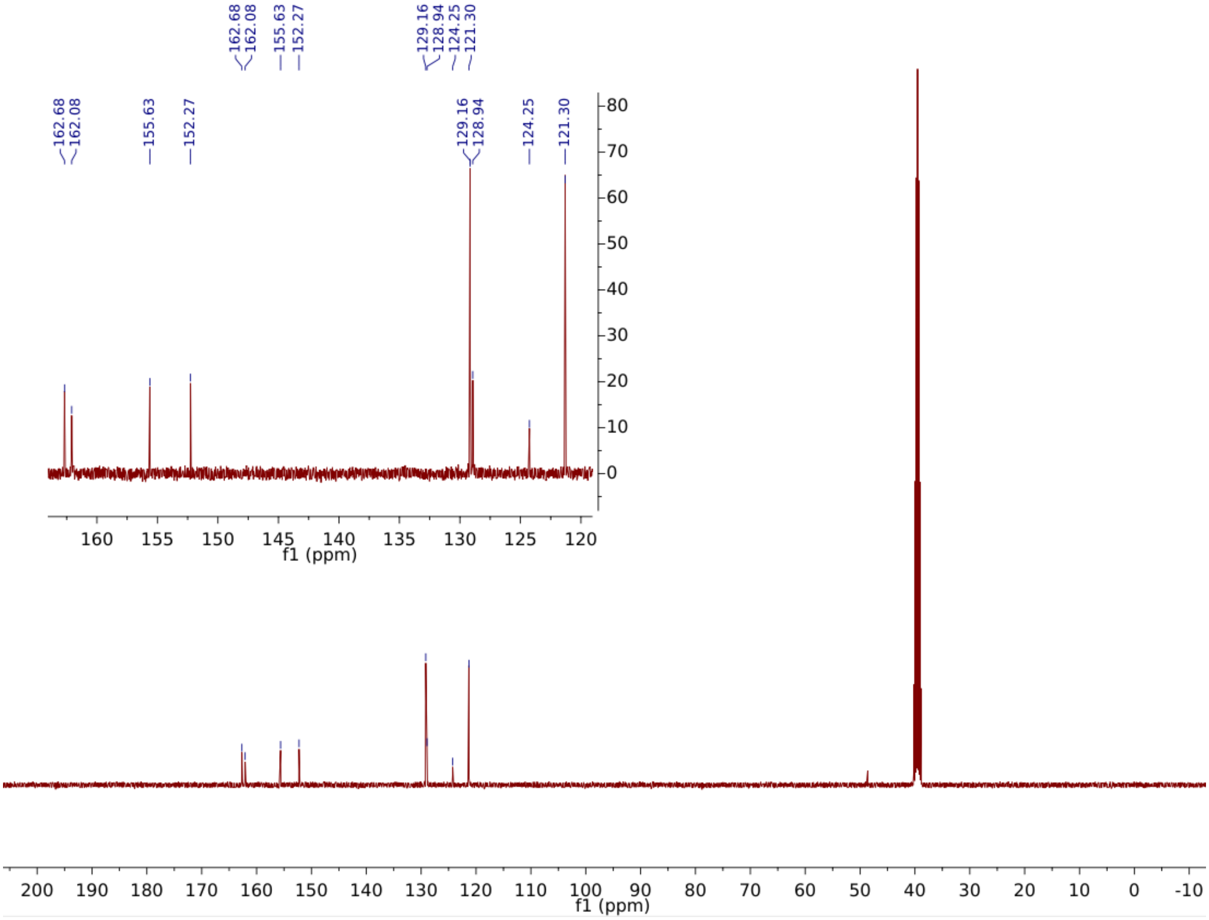

Compound 1 (HRMS)

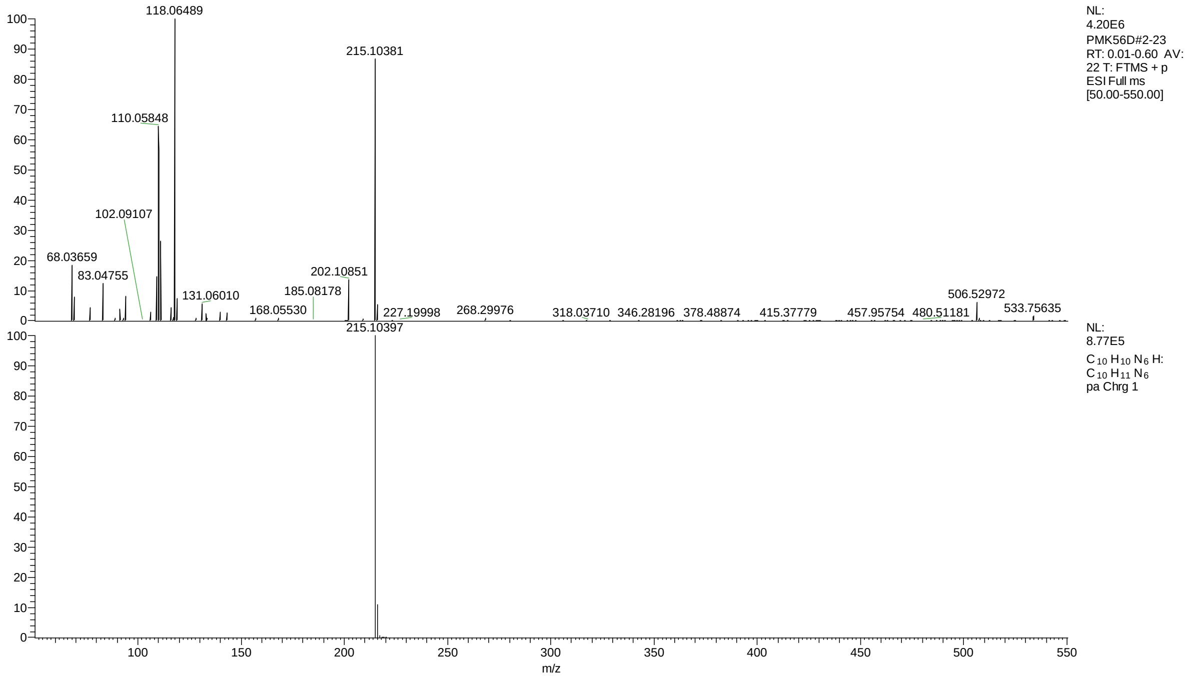

Compound S3a (^{1}H NMR, 400 MHz, DMSO-d_6)

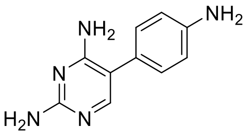

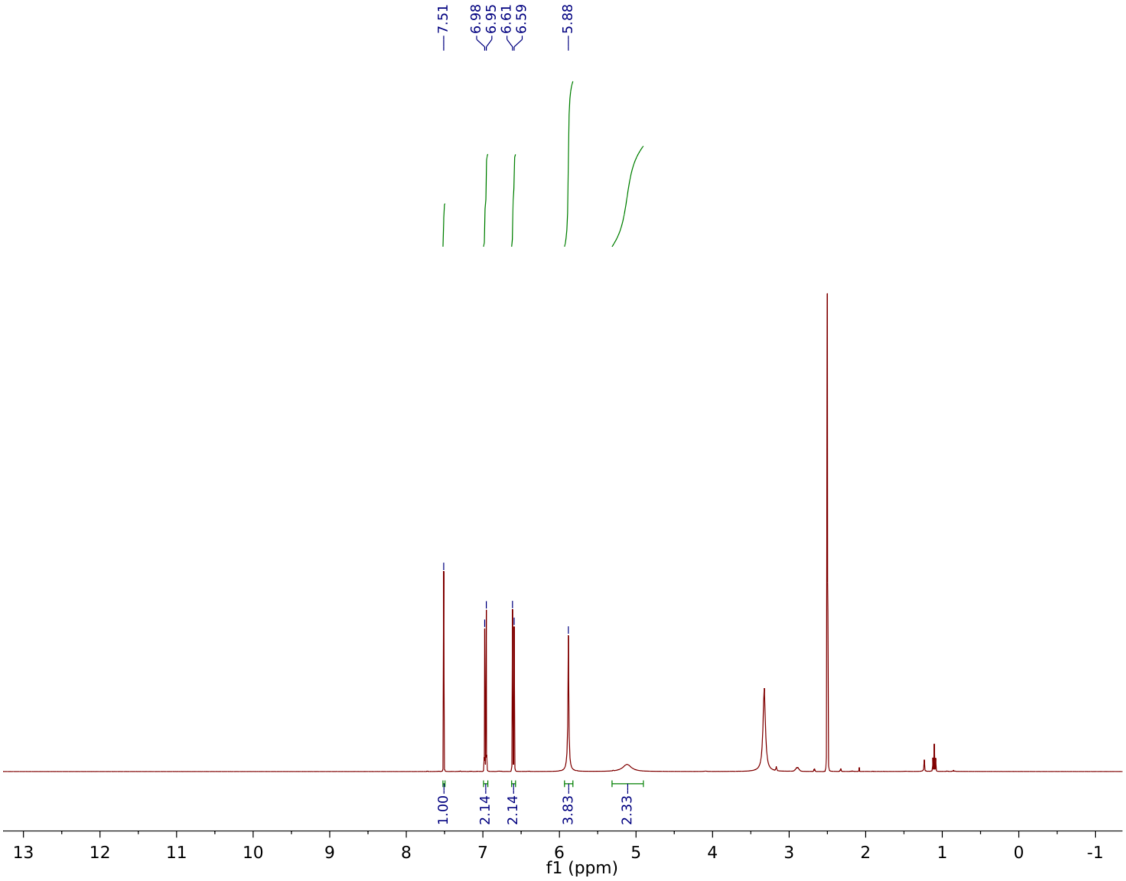

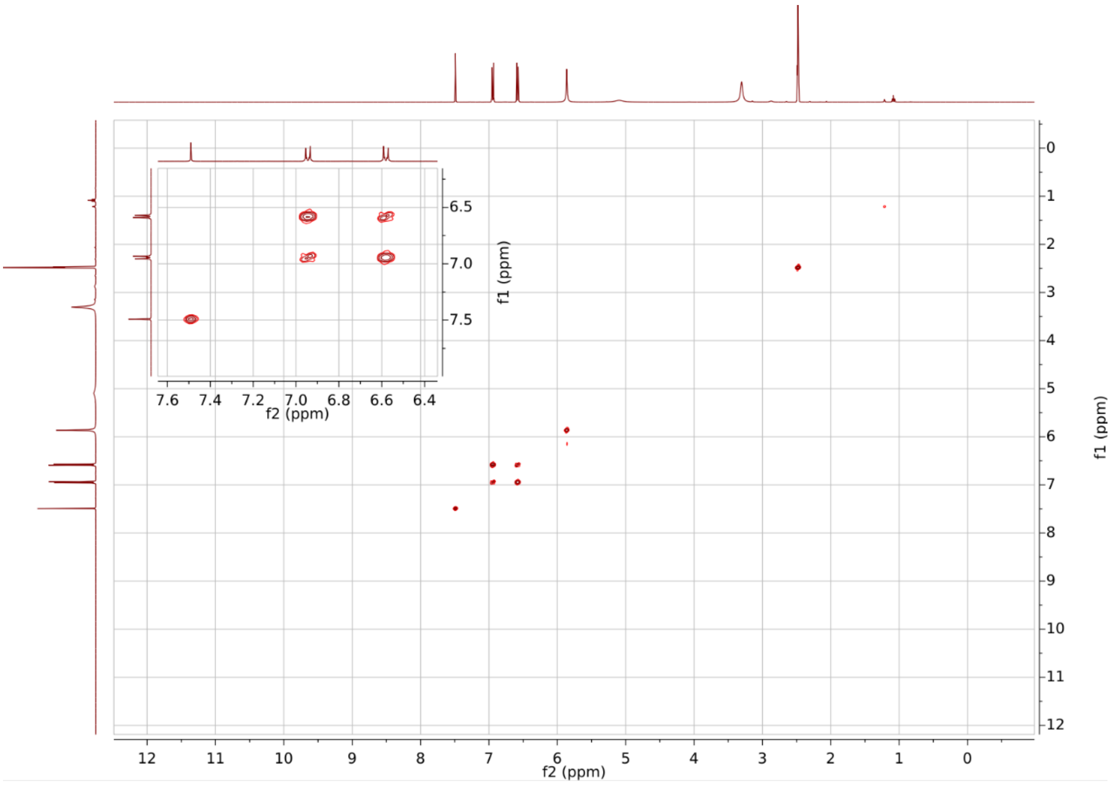

Compound S3a (^{13}C NMR, 101 MHz, DMSO-d_6)

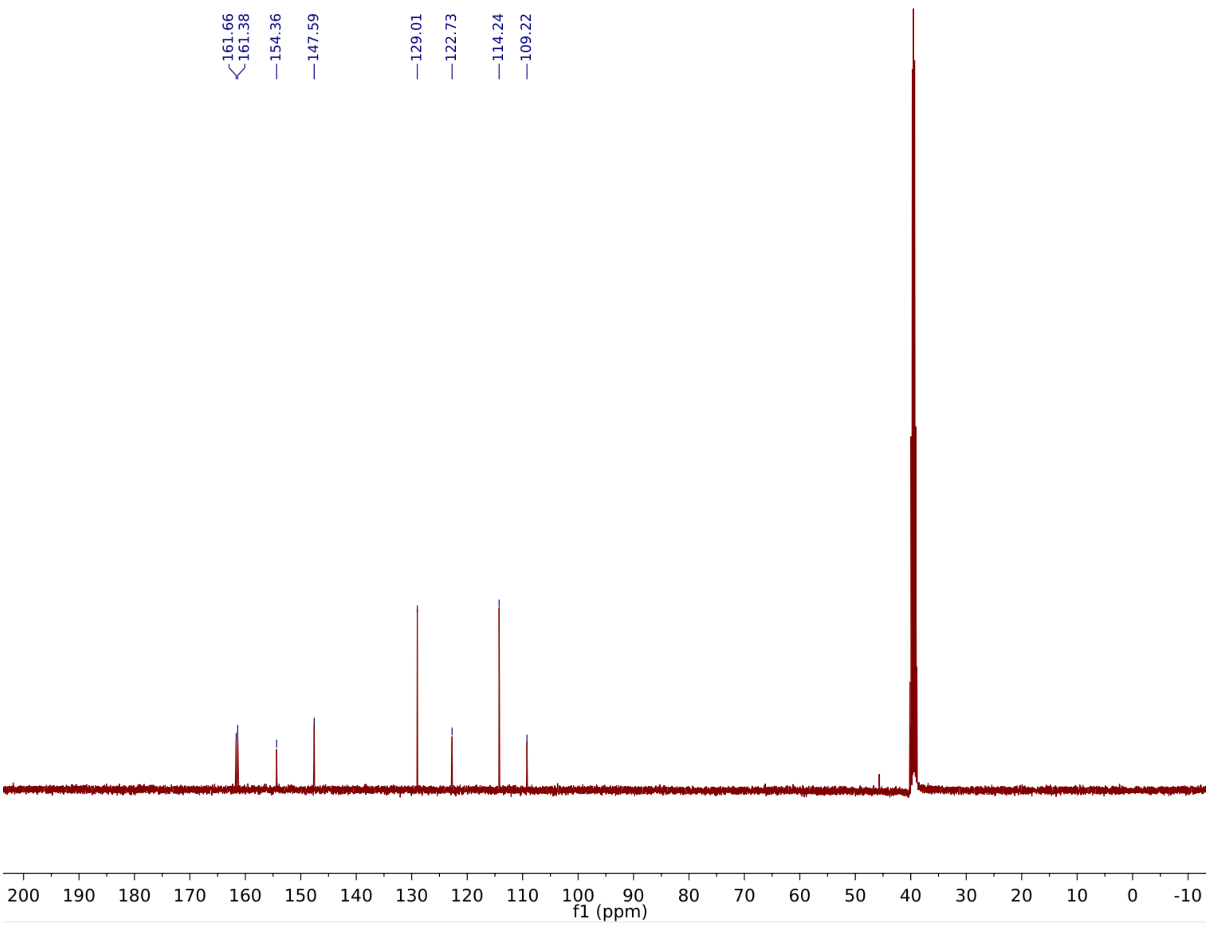

Compound S3b (^{1}H NMR, 400 MHz, DMSO-d_6)

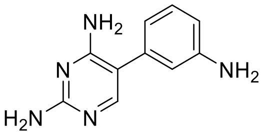

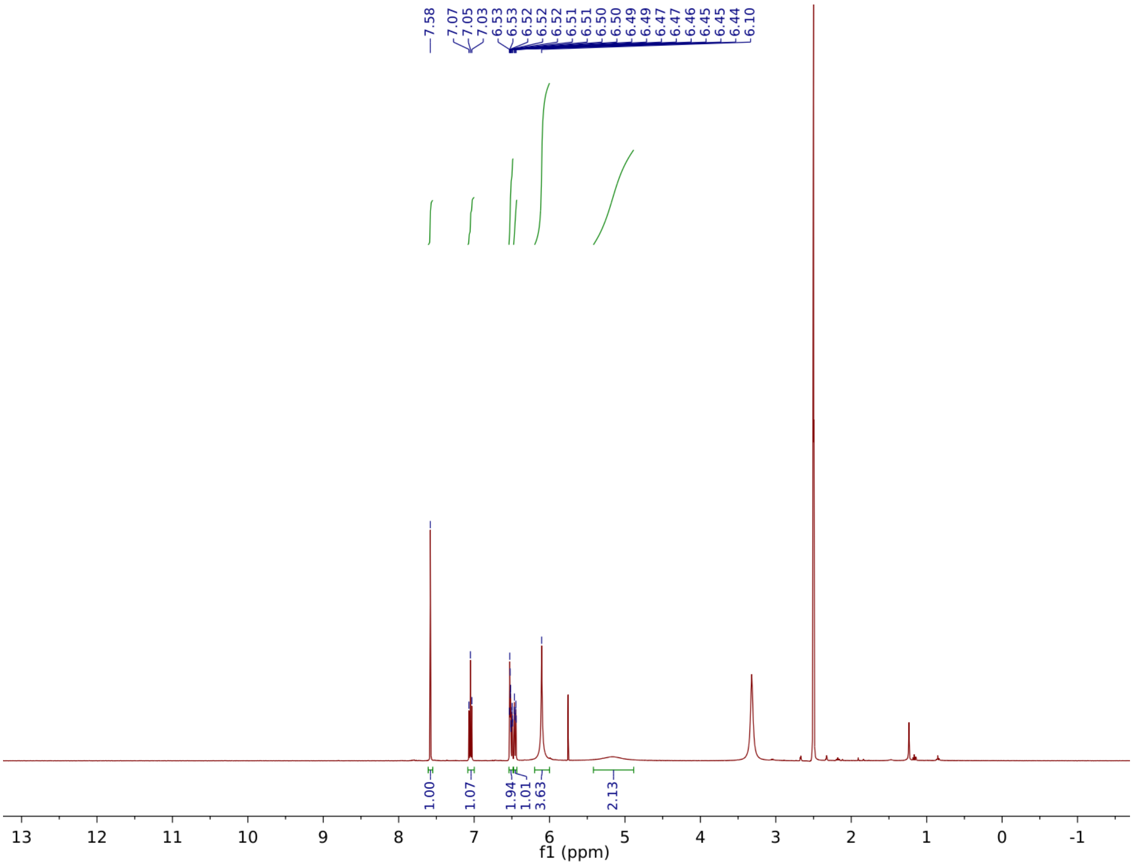

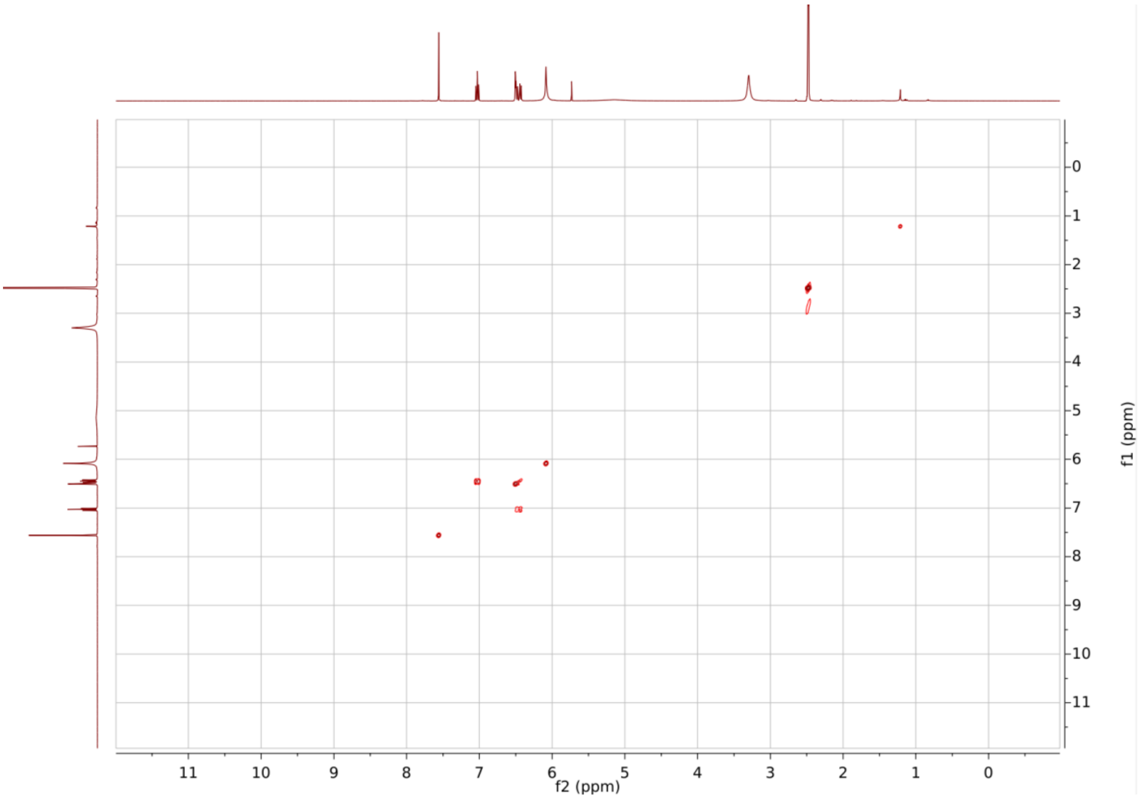

Compound S3b (^{13}C NMR, 101 MHz, DMSO-d_6)

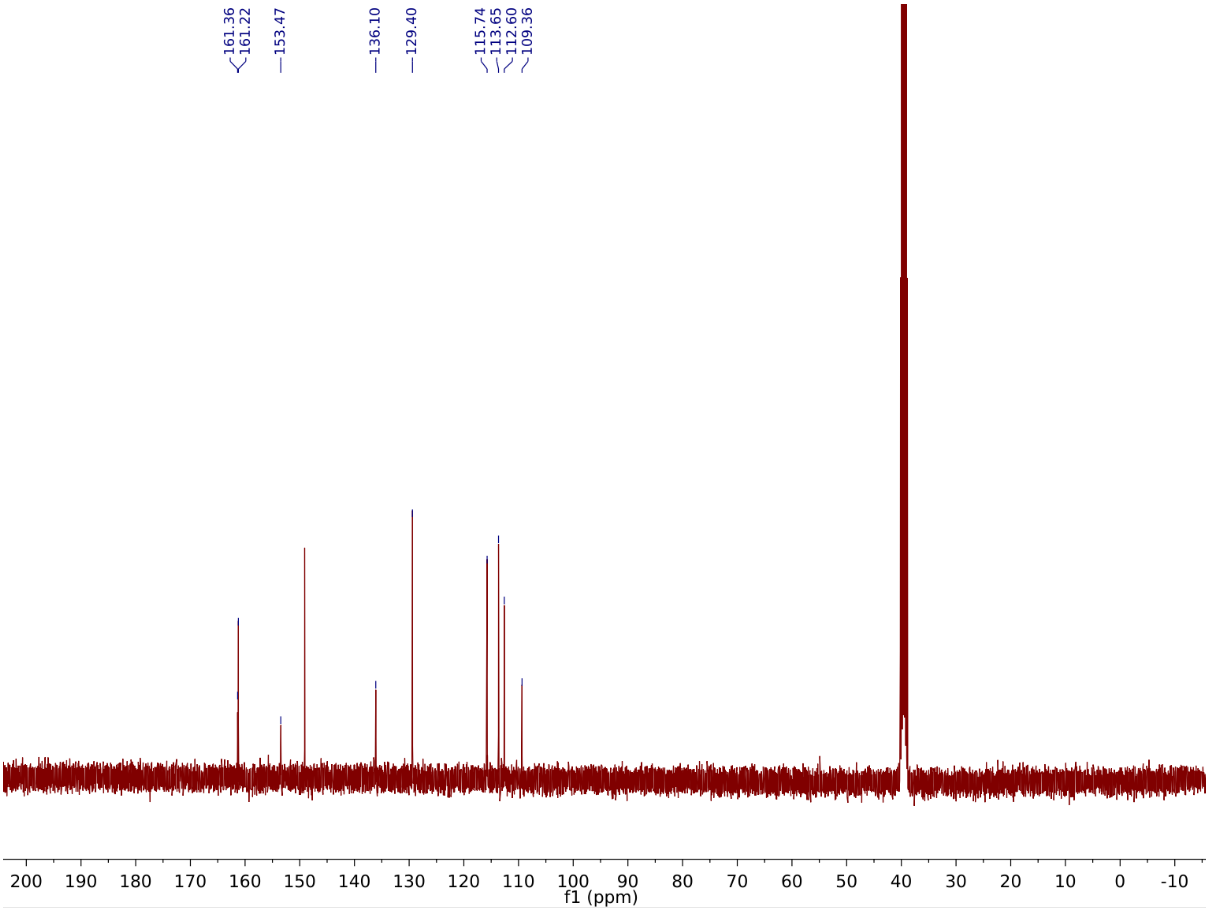

Compound 2 (^{1}H NMR, 400 MHz, DMSO-d_6)

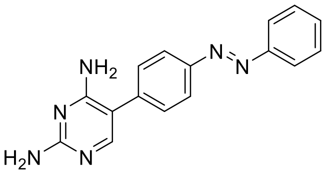

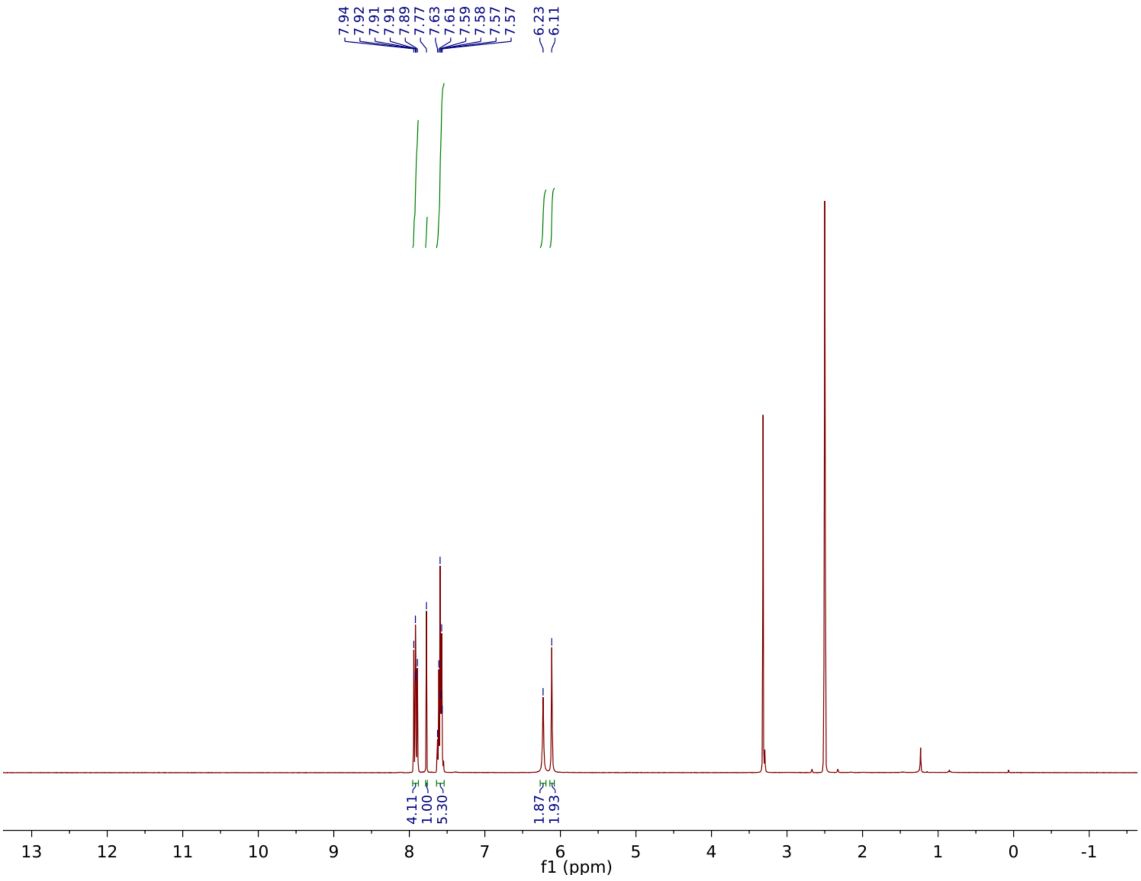

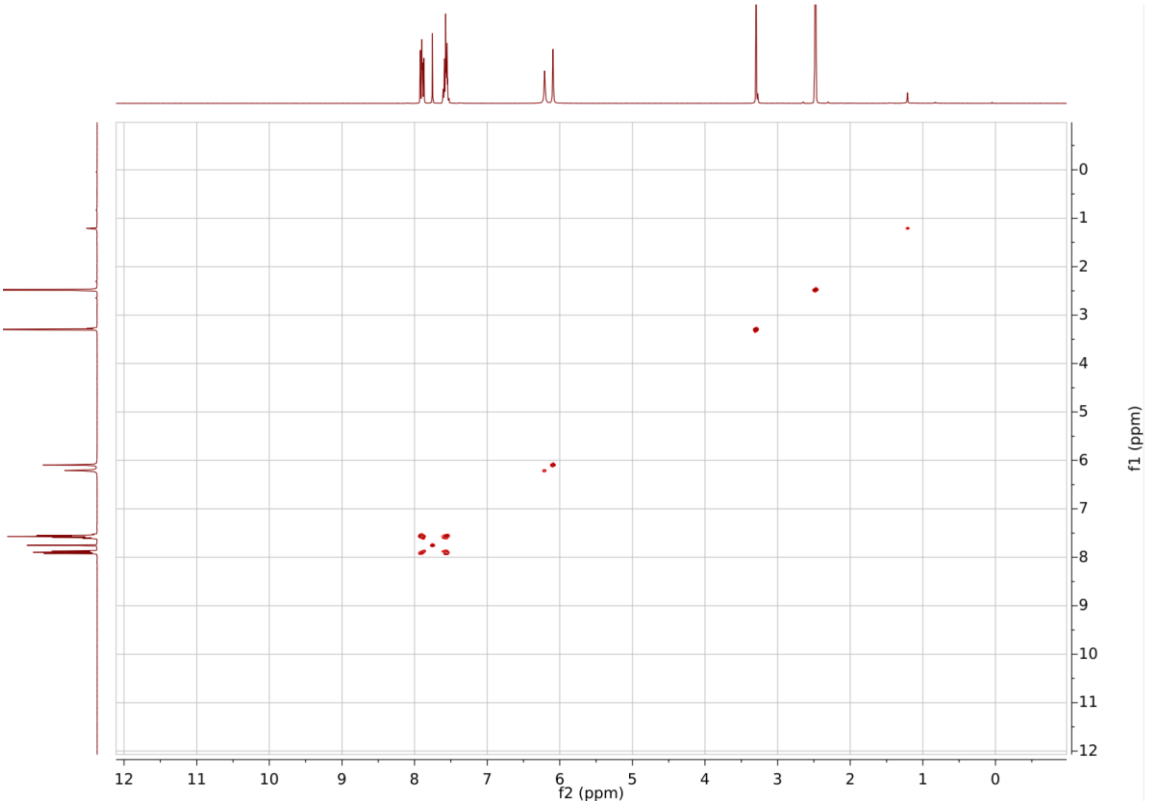

Compound 2 (^{13}C NMR, 101 MHz, DMSO-d_6)

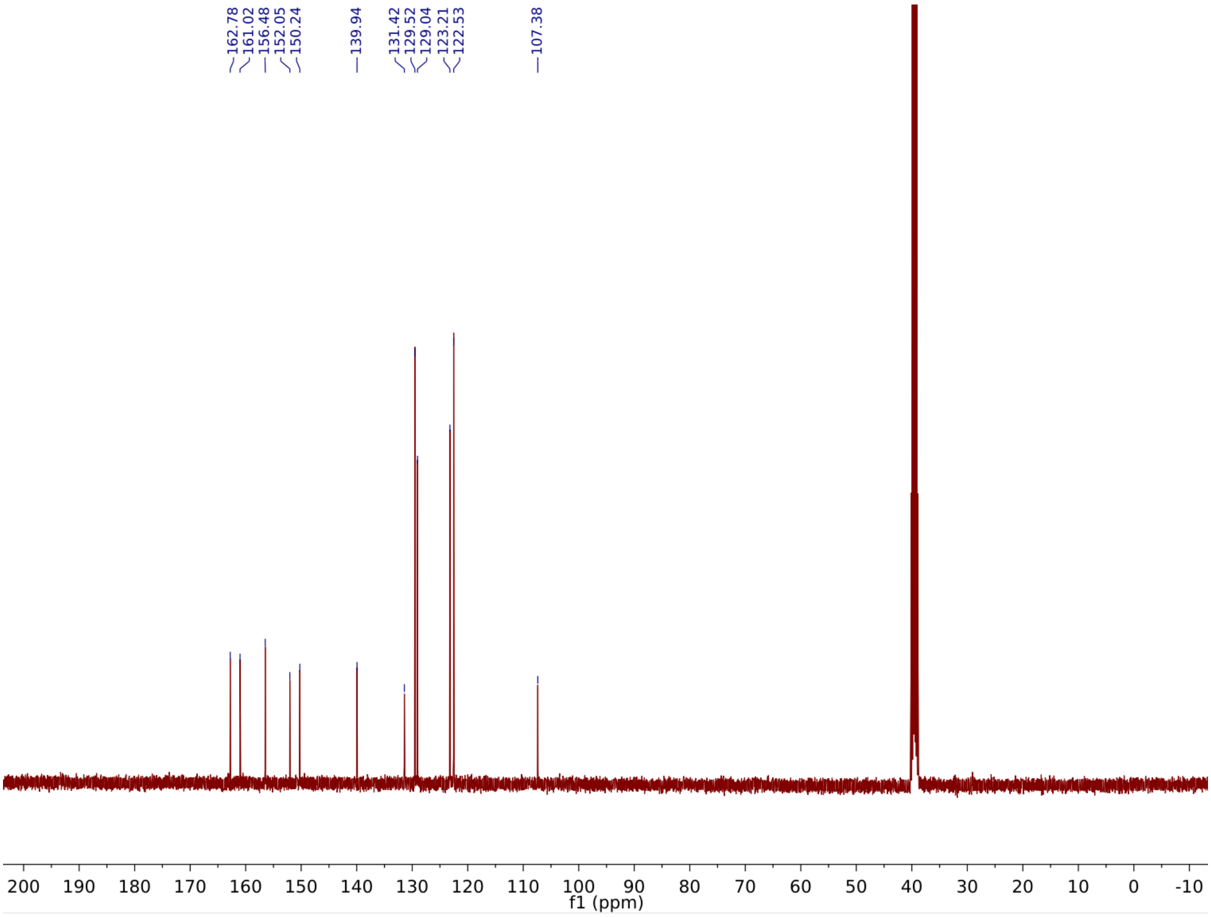

Compound 2 (HRMS)

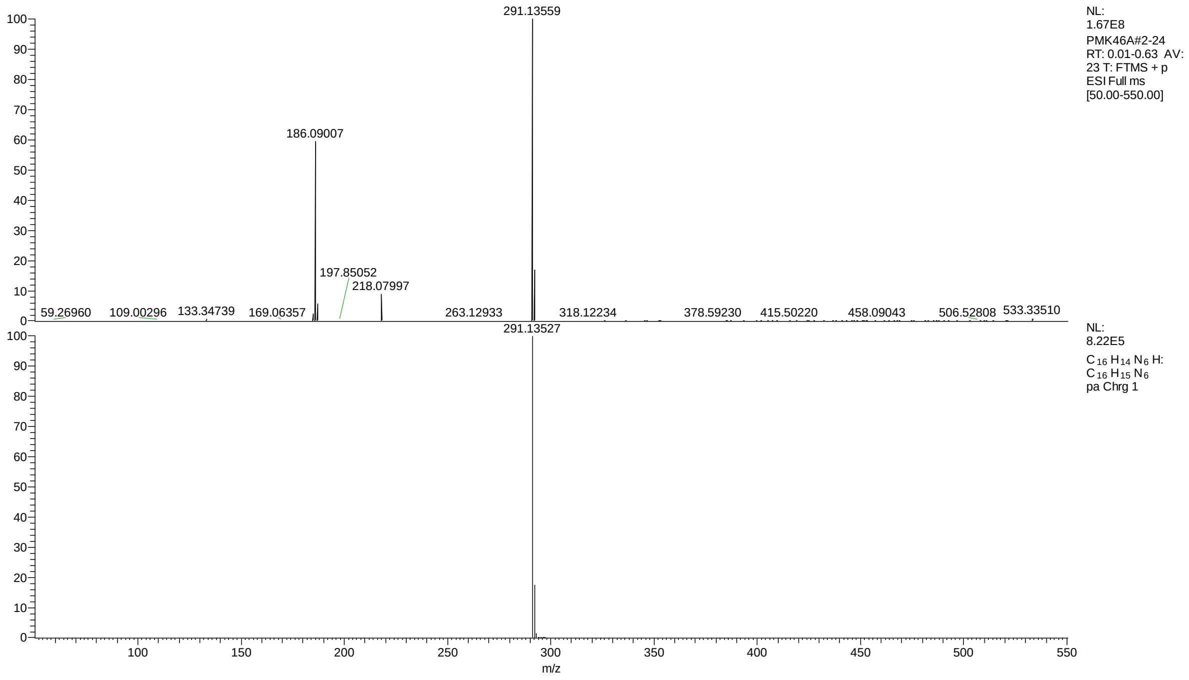

Compound 3 (^{1}H NMR, 400 MHz, DMSO-d_6)

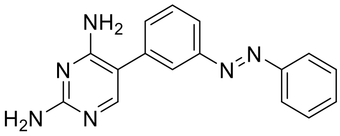

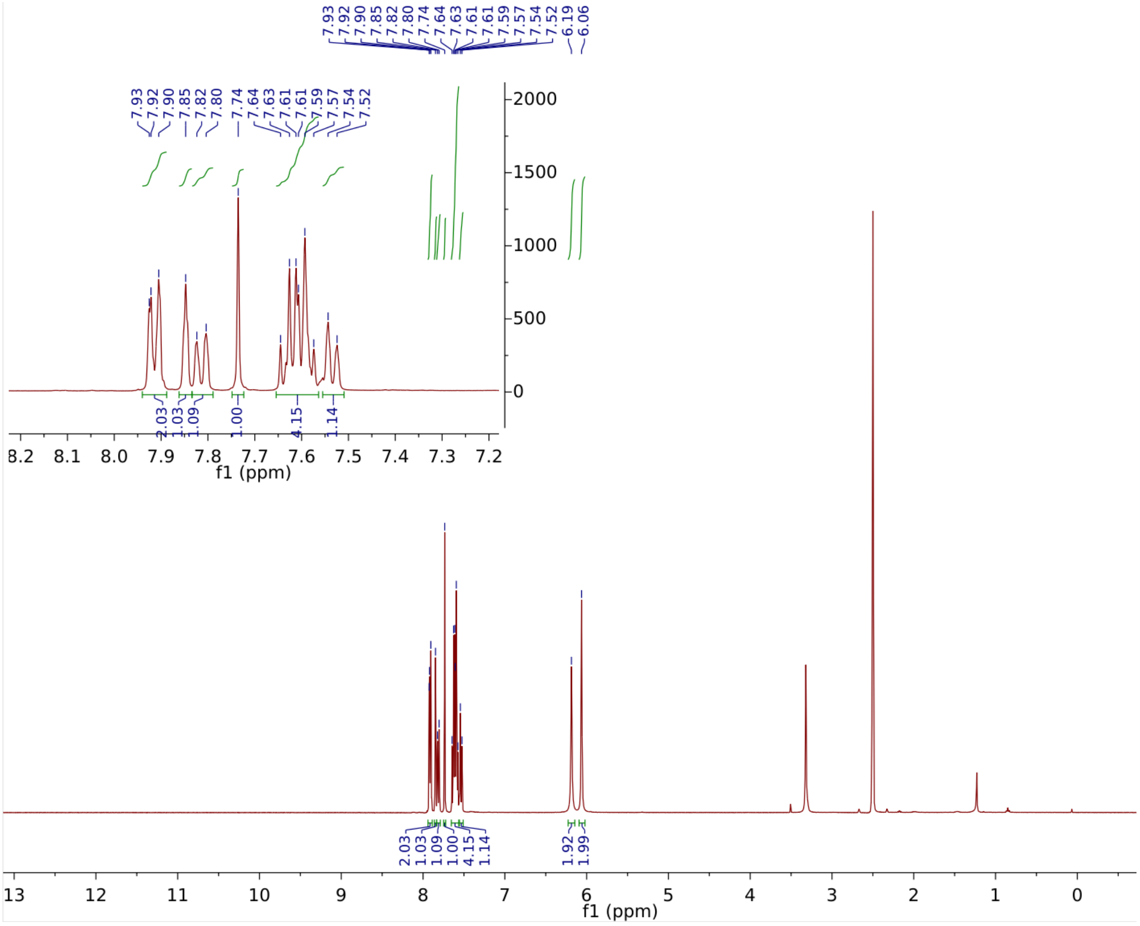

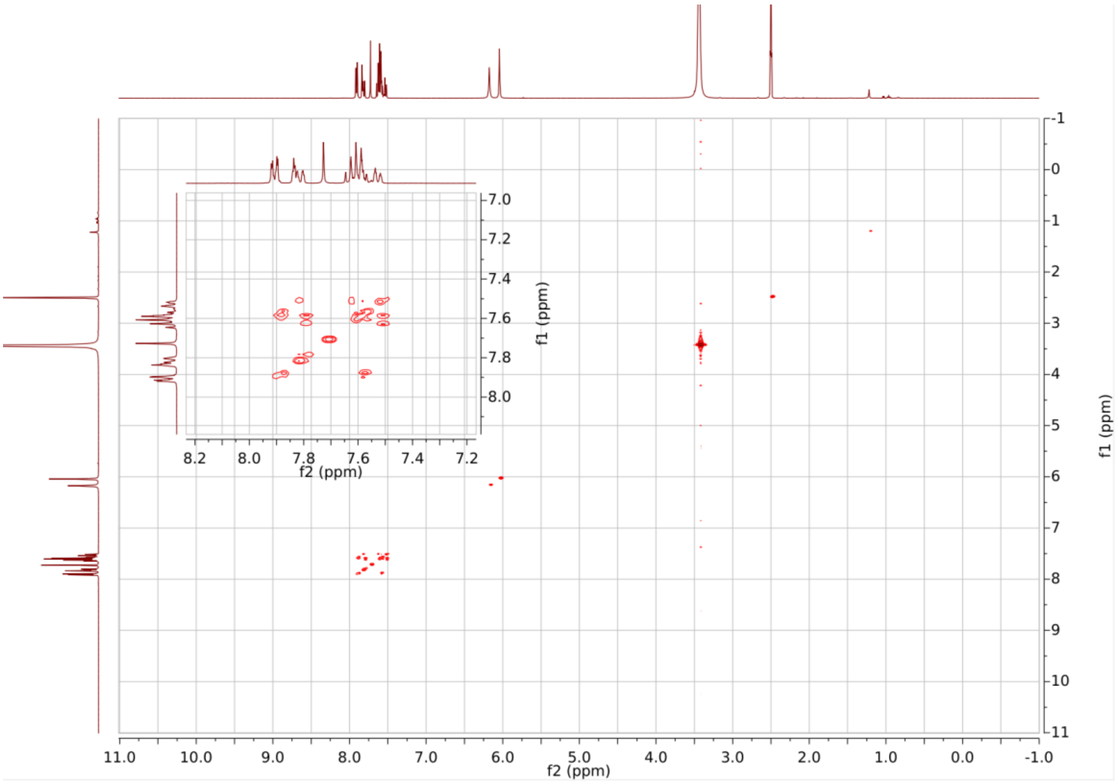

Compound 3 (^{13}C NMR, 101 MHz, DMSO-d_6)

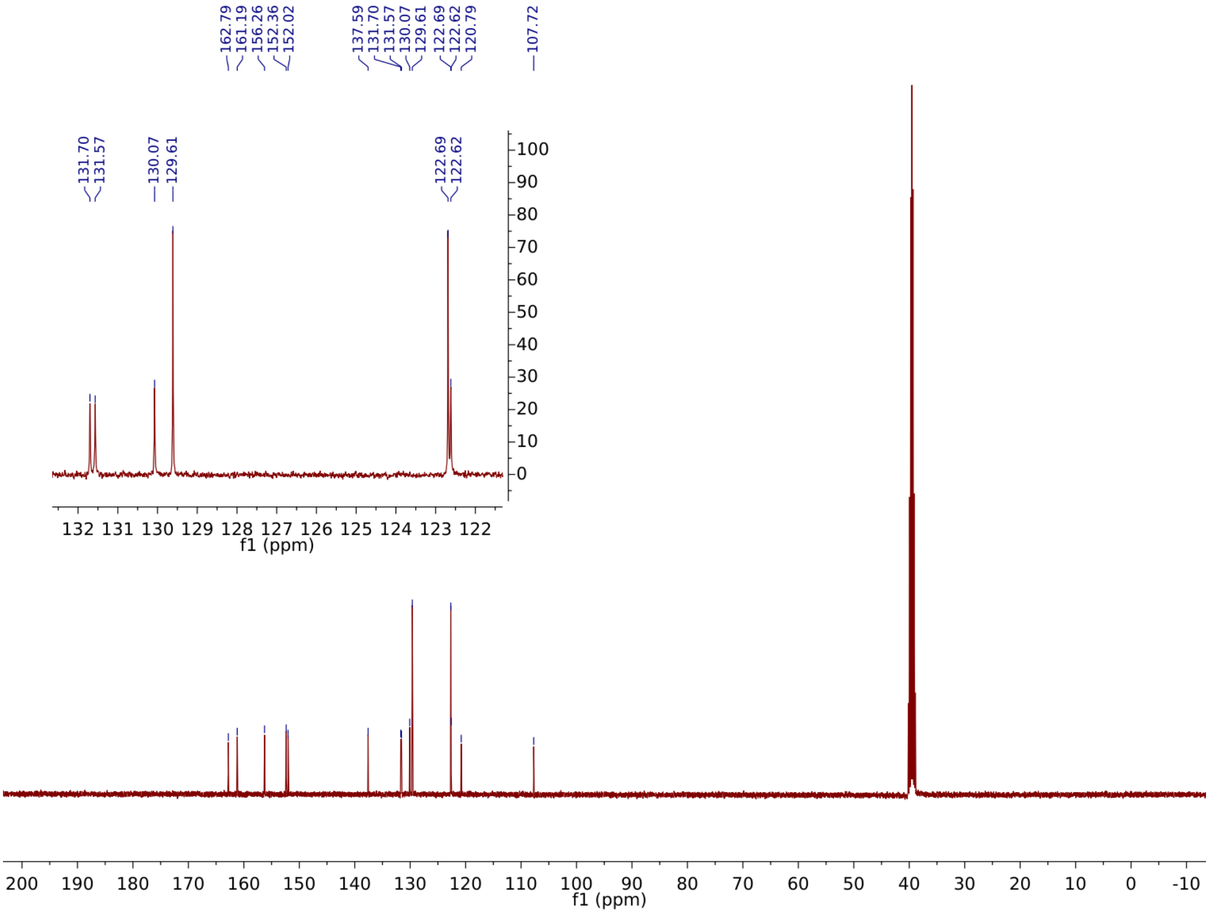

Compound 3 (HRMS)

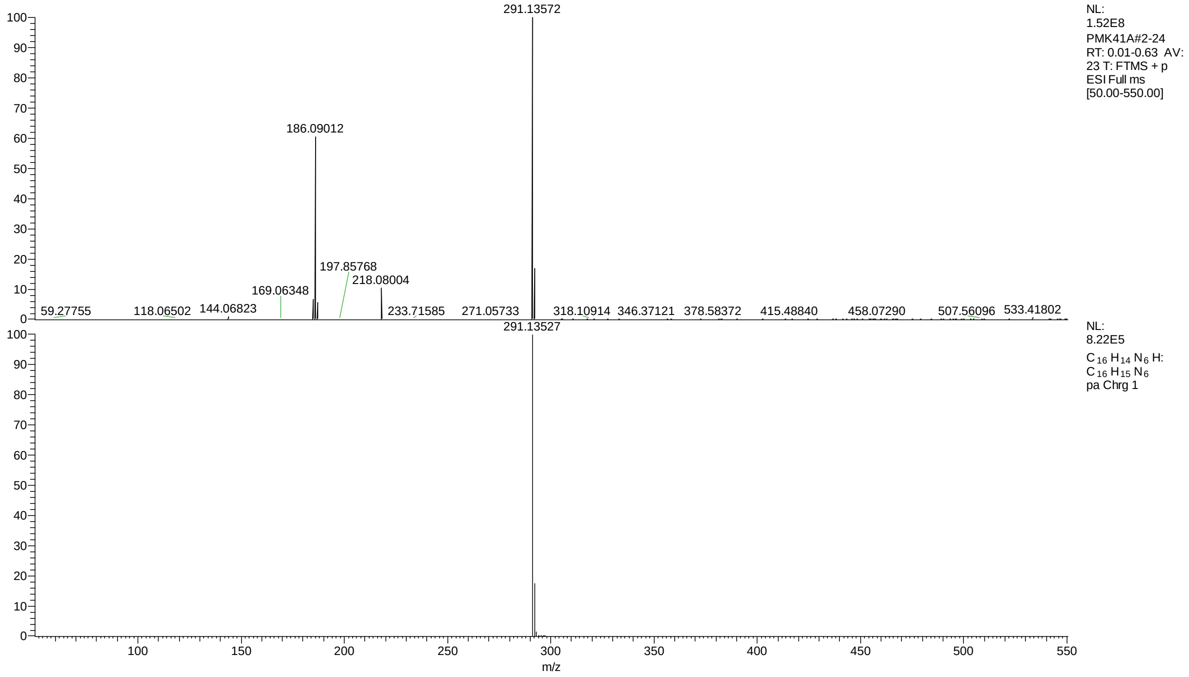

Compound S4a (^{1}H NMR, 400 MHz, $CDCl_3$)

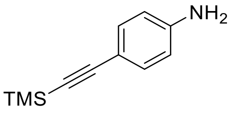

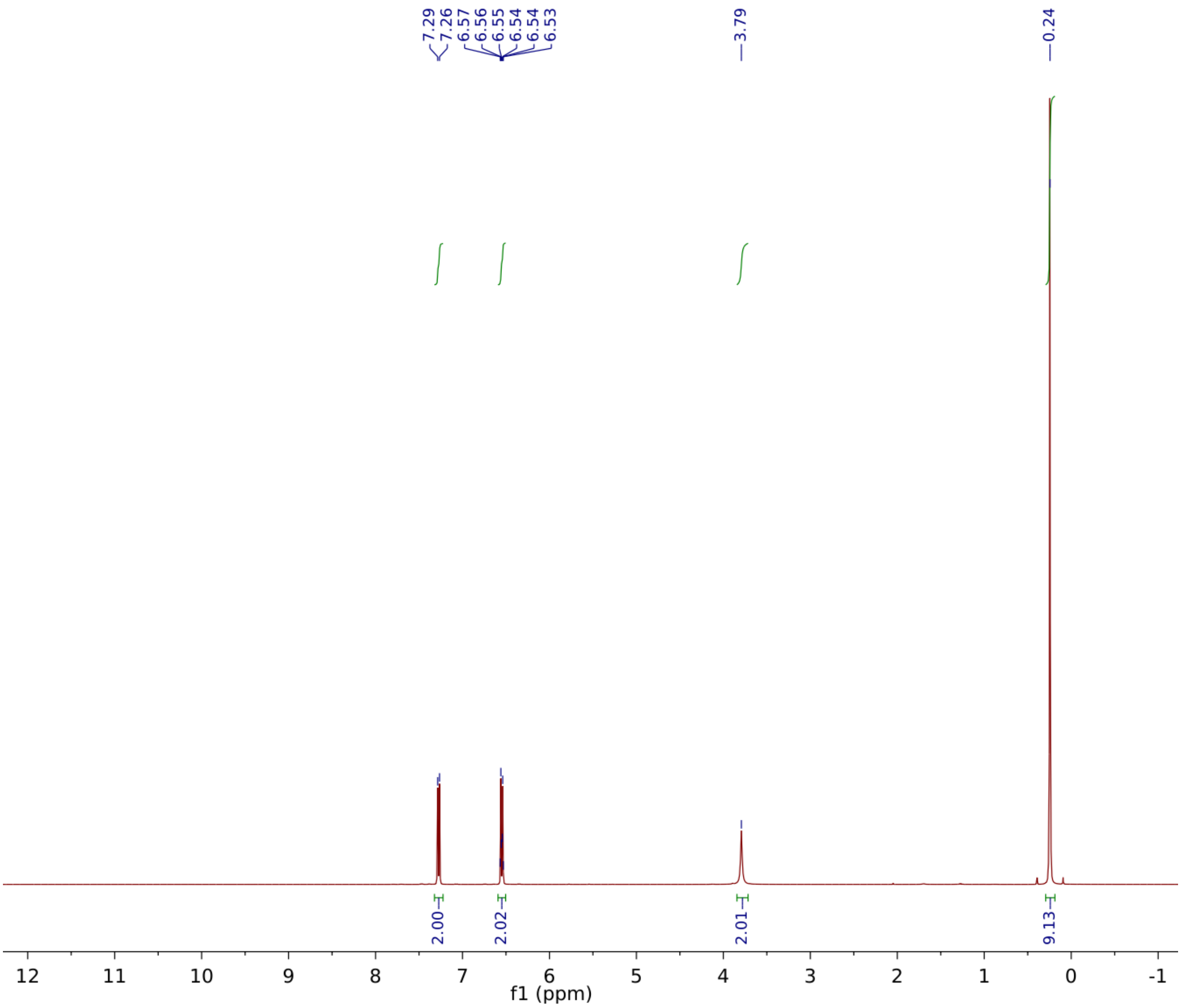

Compound S4b (^{1}H NMR, 400 MHz, $CDCl_3$)

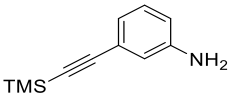

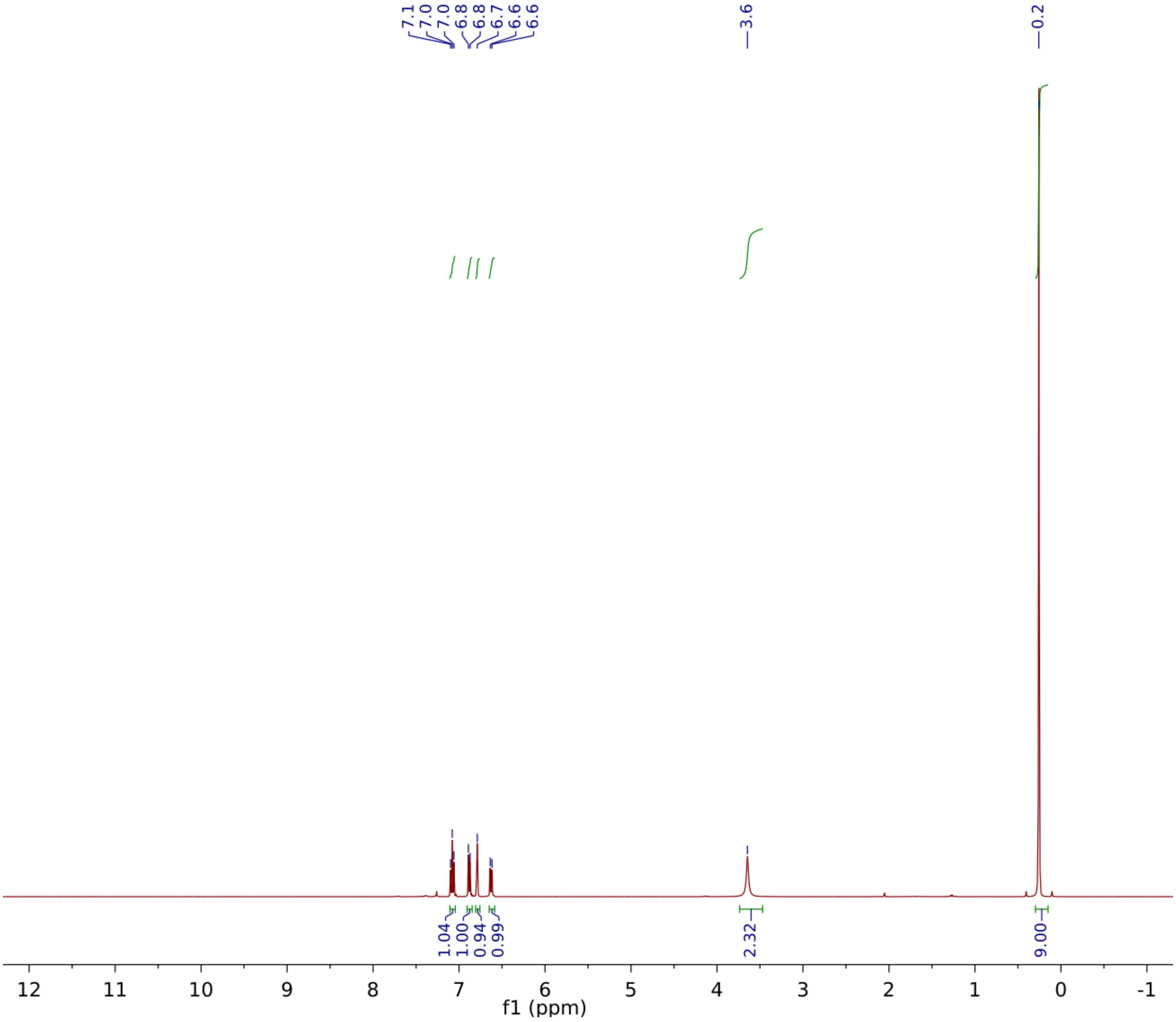

Compound S5a (^{1}H NMR, 400 MHz, $CDCl_3$)

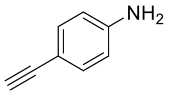

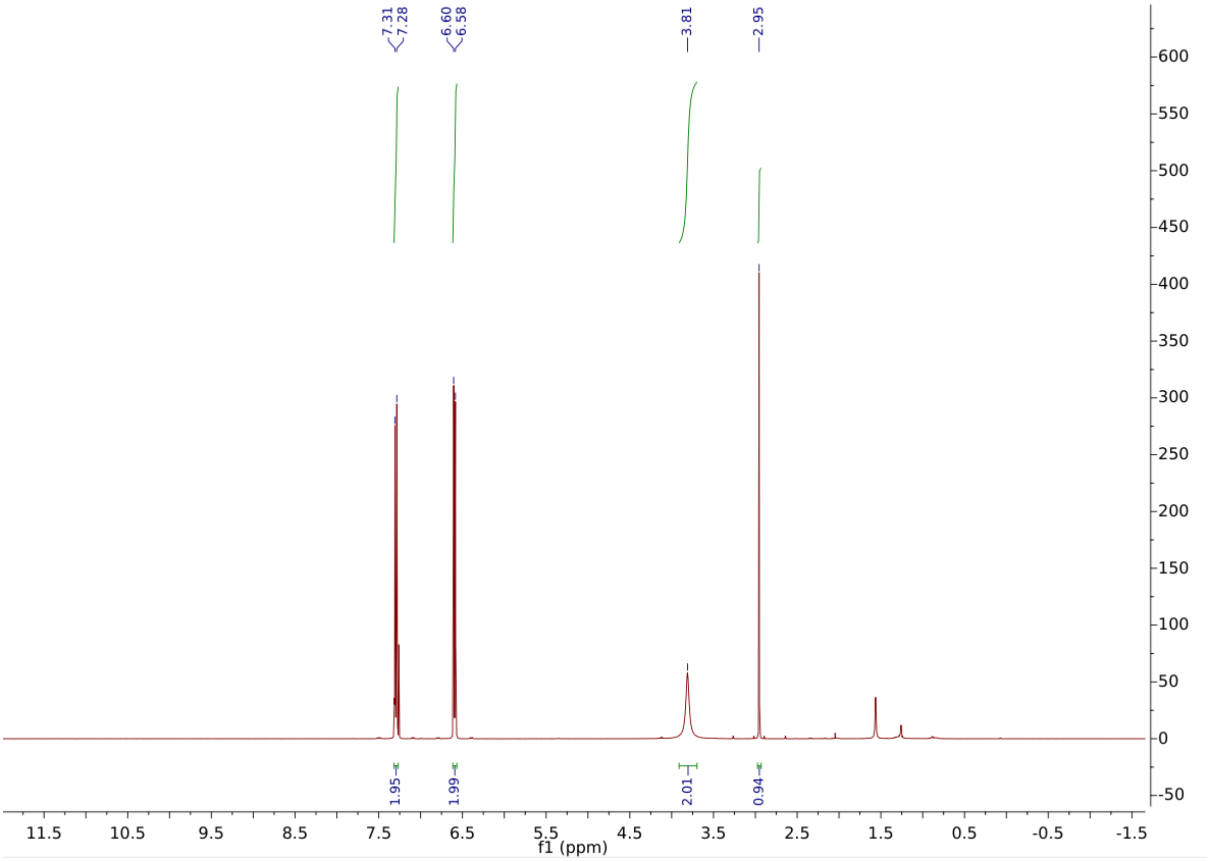

Compound S5b (^{1}H NMR, 400 MHz, $CDCl_3$)

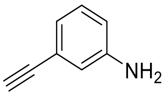

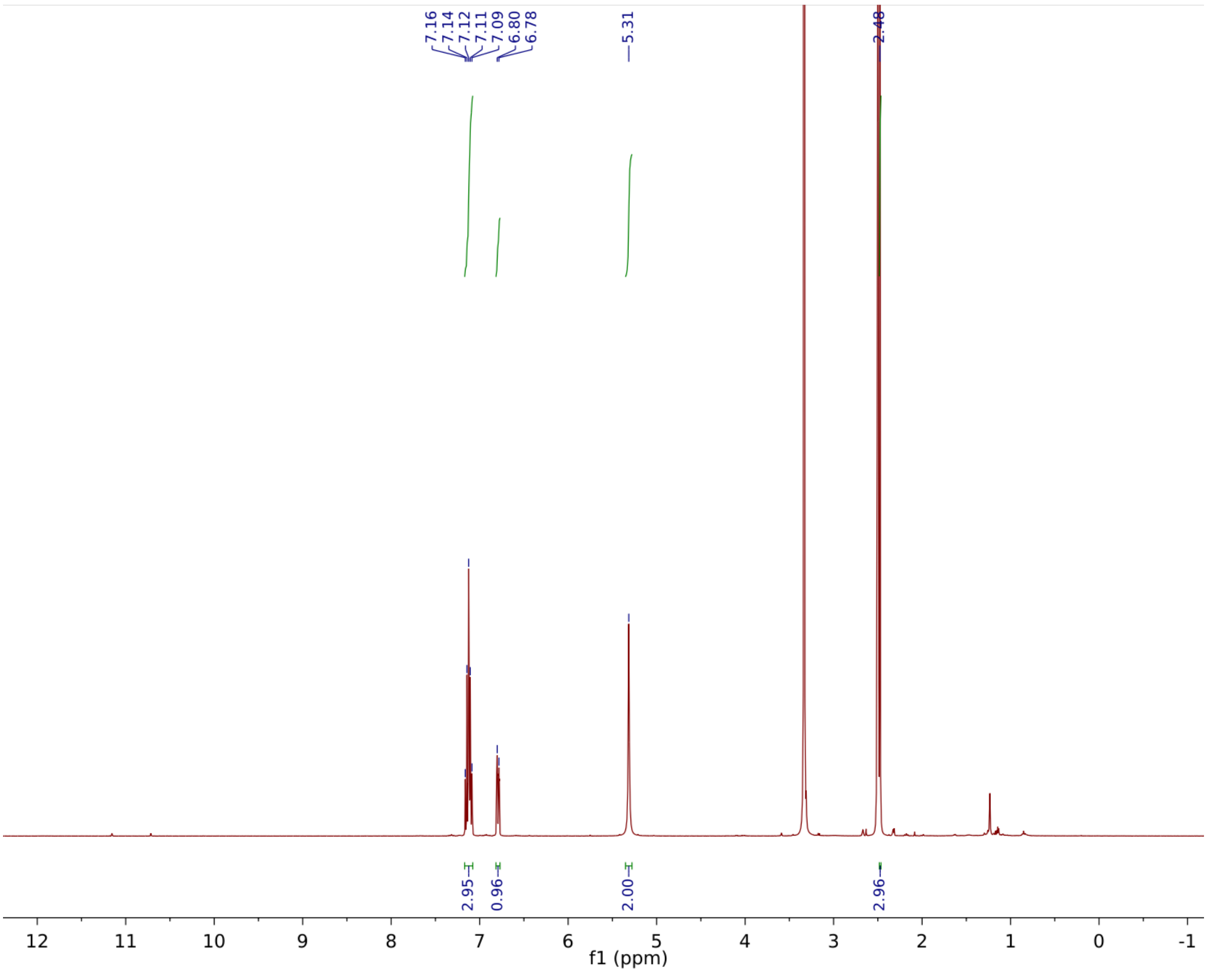

Compound S6a (^{1}H NMR, 400 MHz, DMSO-d_6)

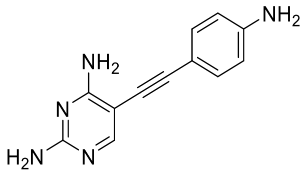

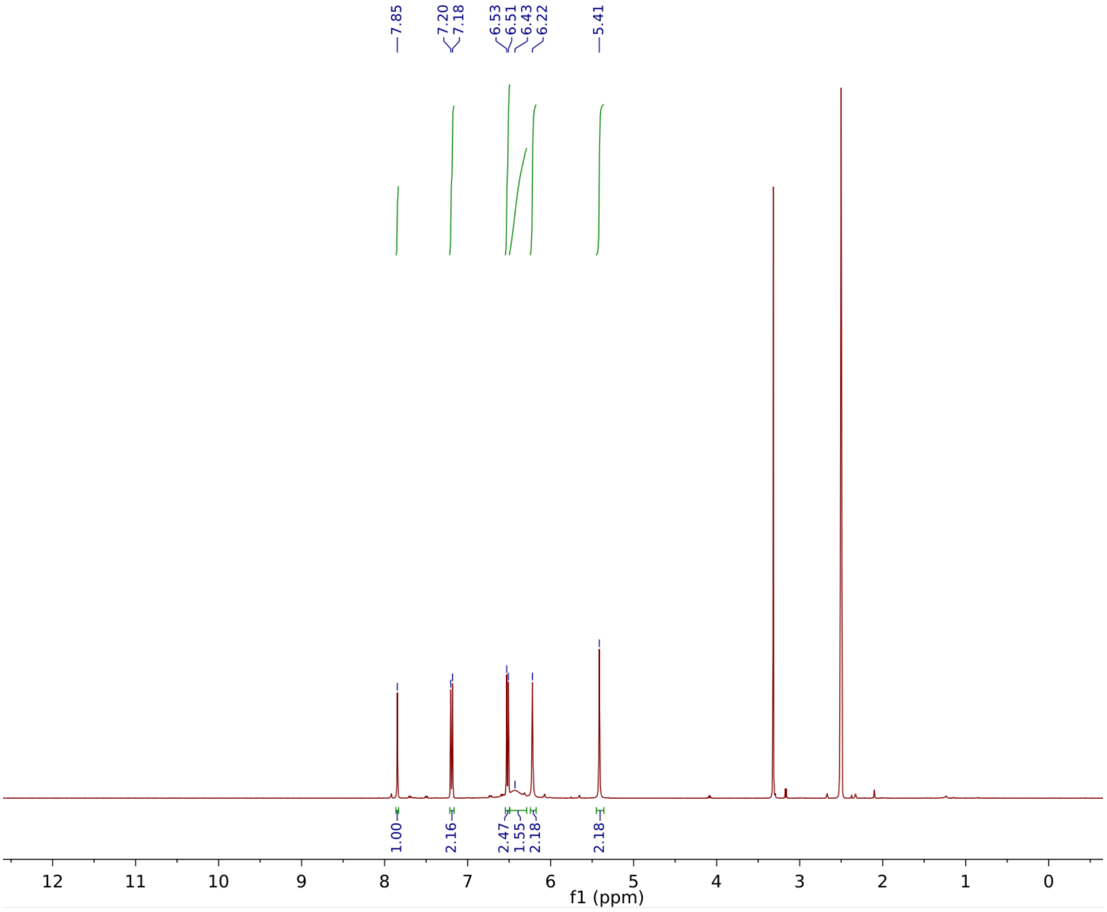

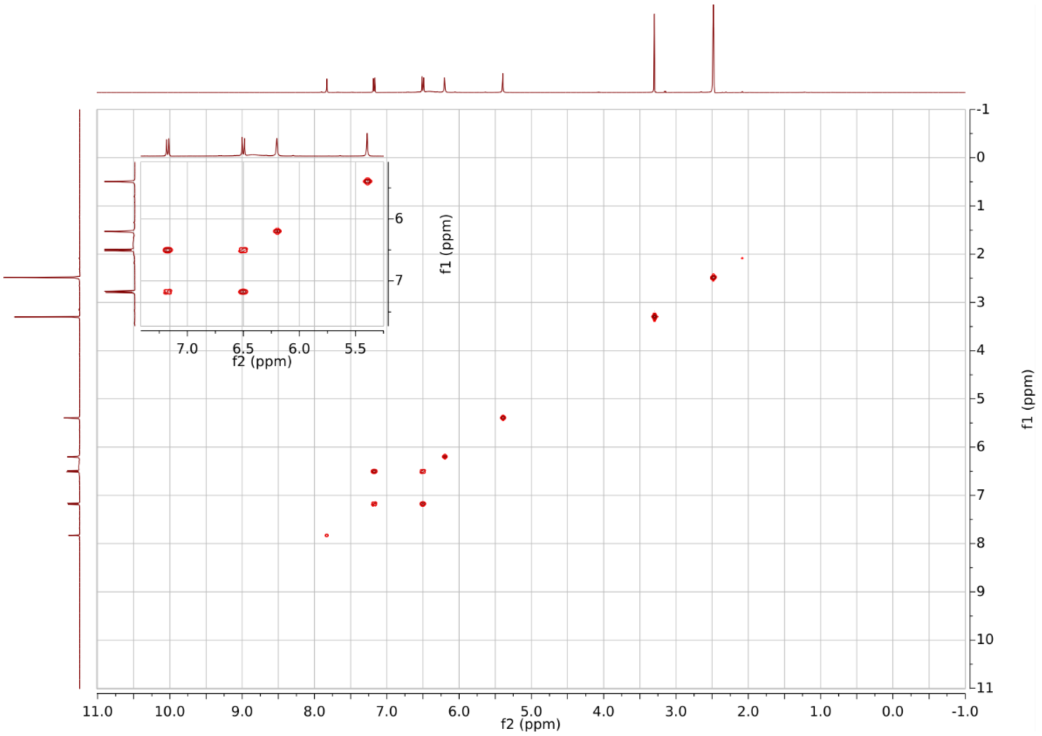

Compound S6a (^{13}C NMR, 101 MHz, DMSO-d_6)

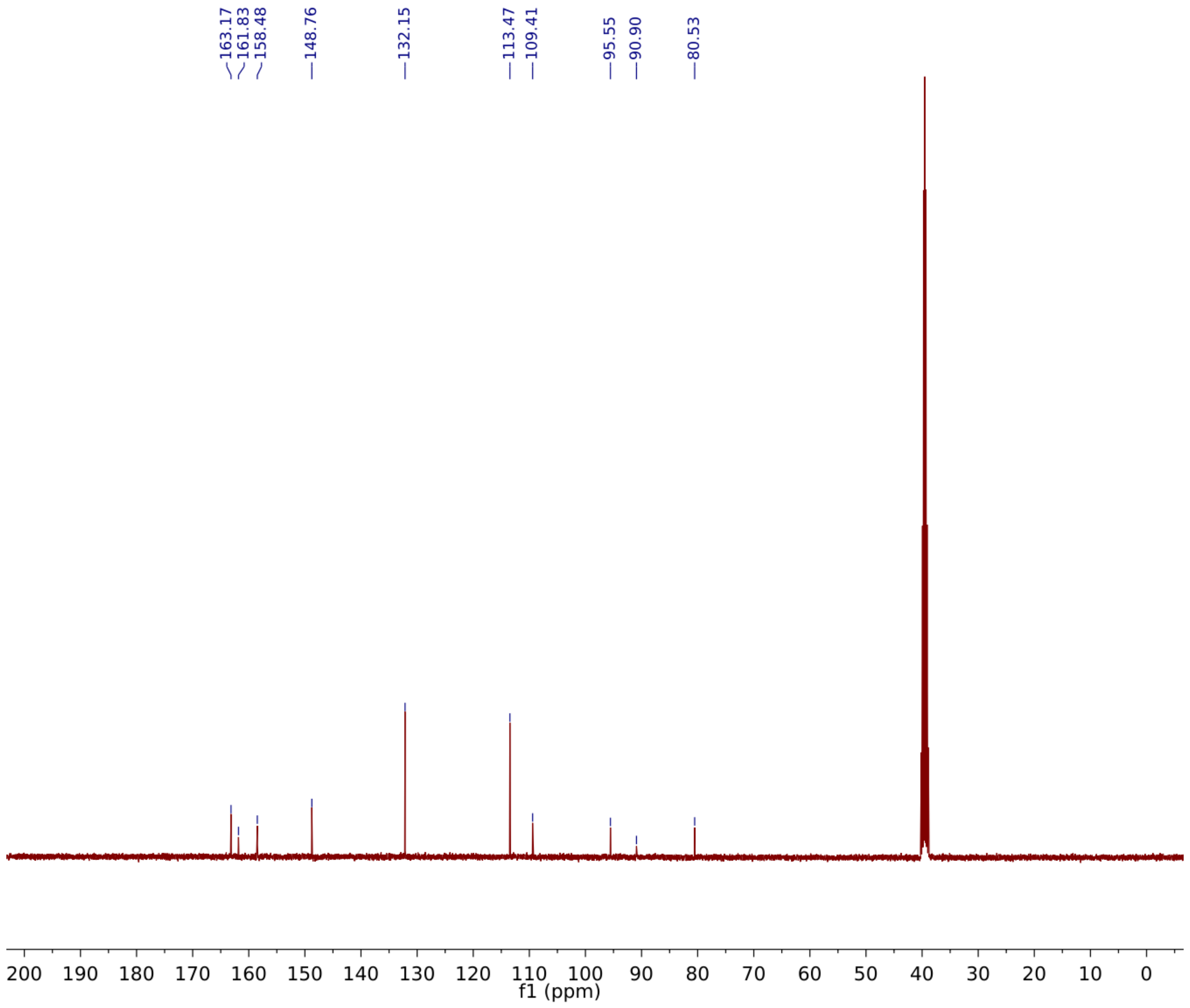

Compound S6b (^{1}H NMR, 400 MHz, DMSO-d_6)

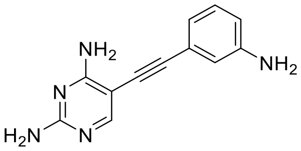

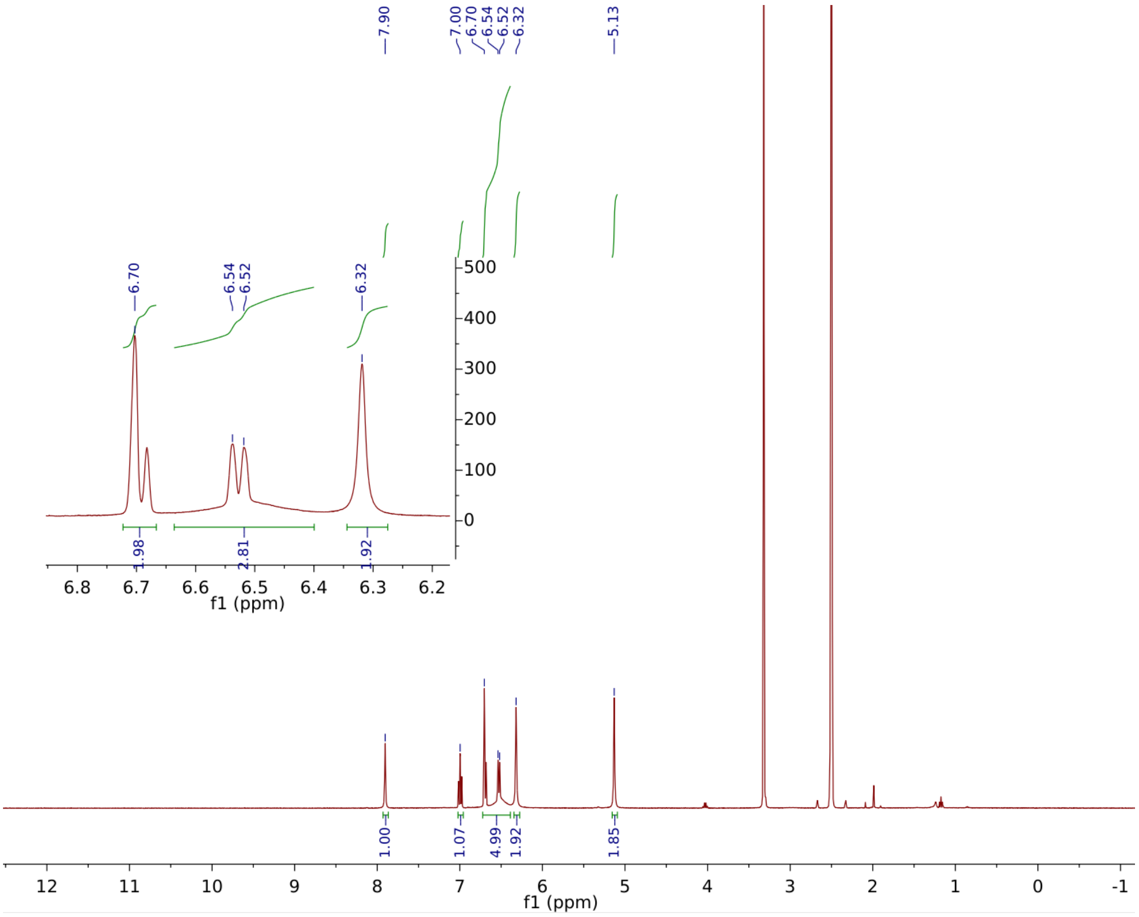

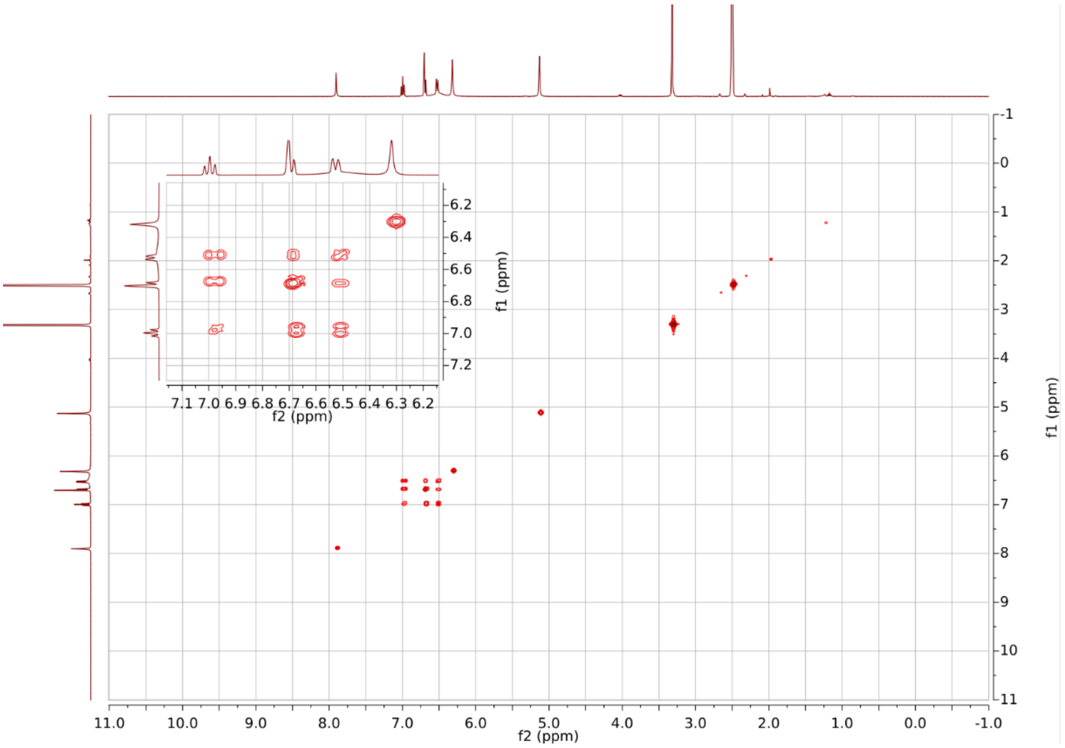

Compound S6b (^{13}C NMR, 101 MHz, DMSO-d_6)

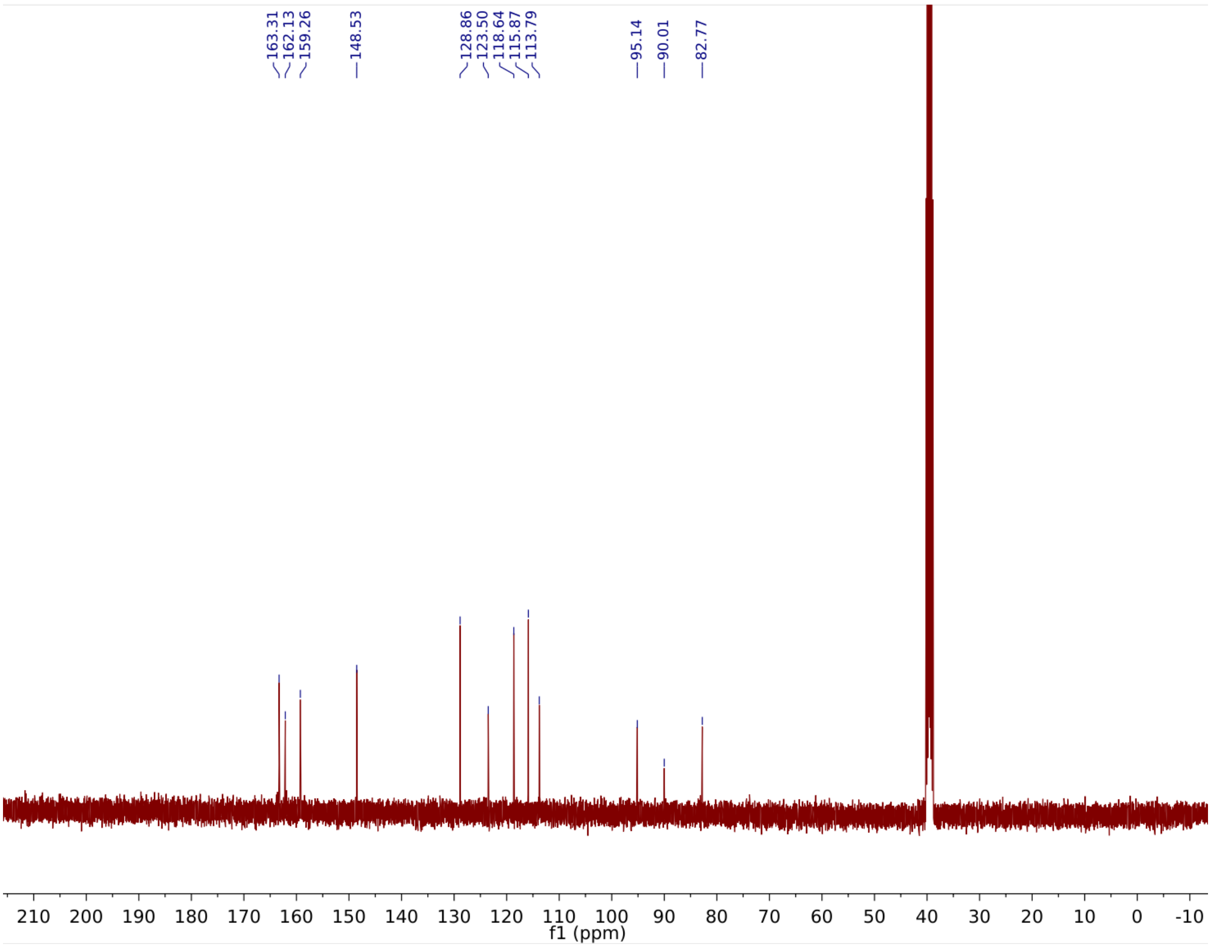
163.31
162.13
159.26
148.53
128.86
123.50
118.64
115.87
113.79
95.14
90.01
82.77
210 200 190 180 170 160 150 140 130 120 110 100 90 80 70 60 50 40 30 20 10 0 -10
f1 (ppm)

Compound 4 (^{1}H NMR, 400 MHz, DMSO-d_6)

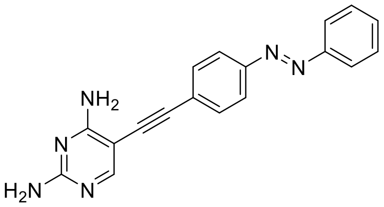

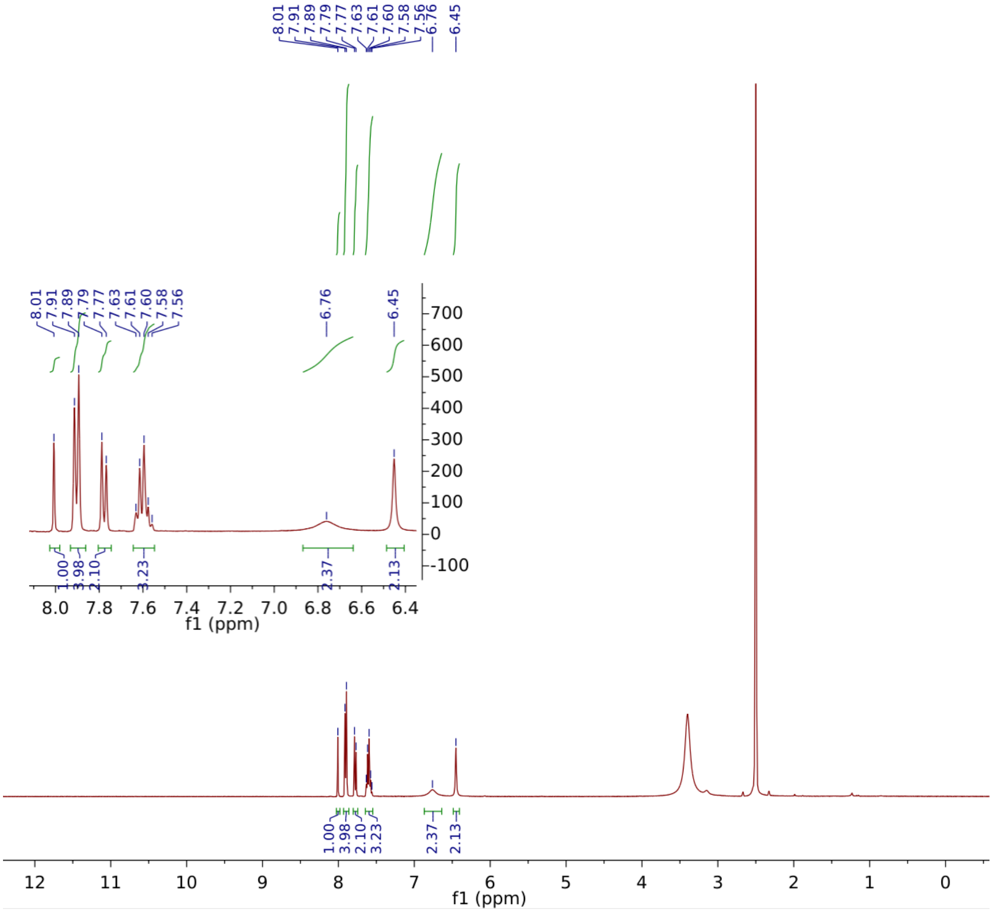

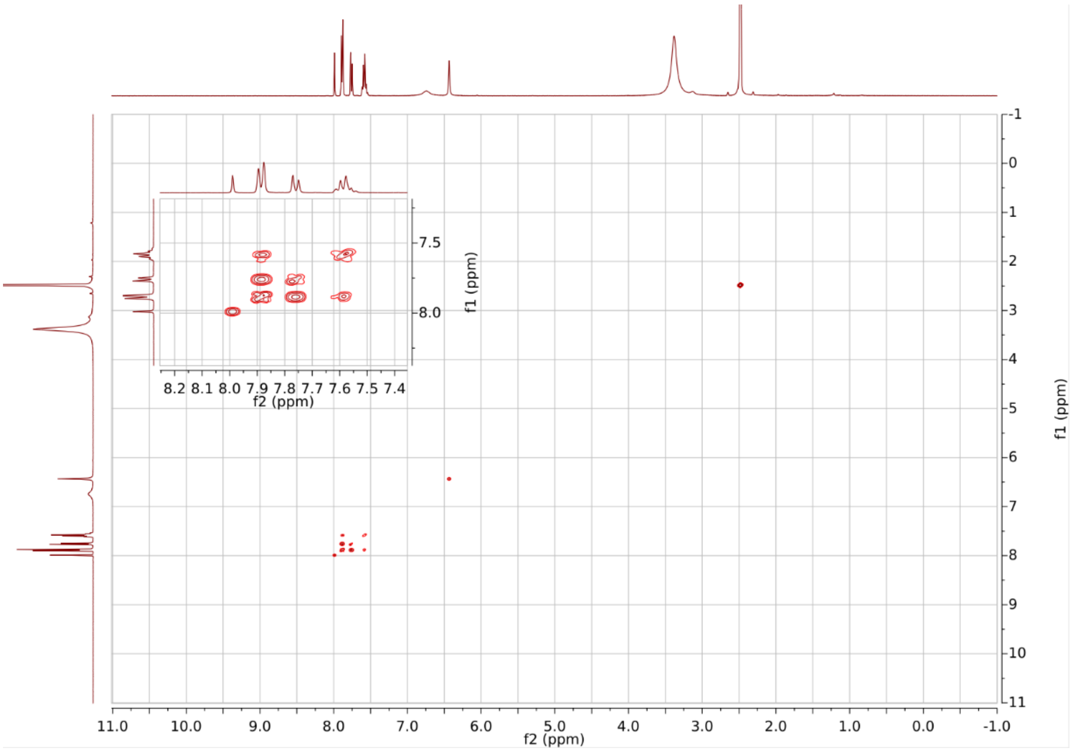

Compound 4 (^{13}C NMR, 101 MHz, DMSO-d6)

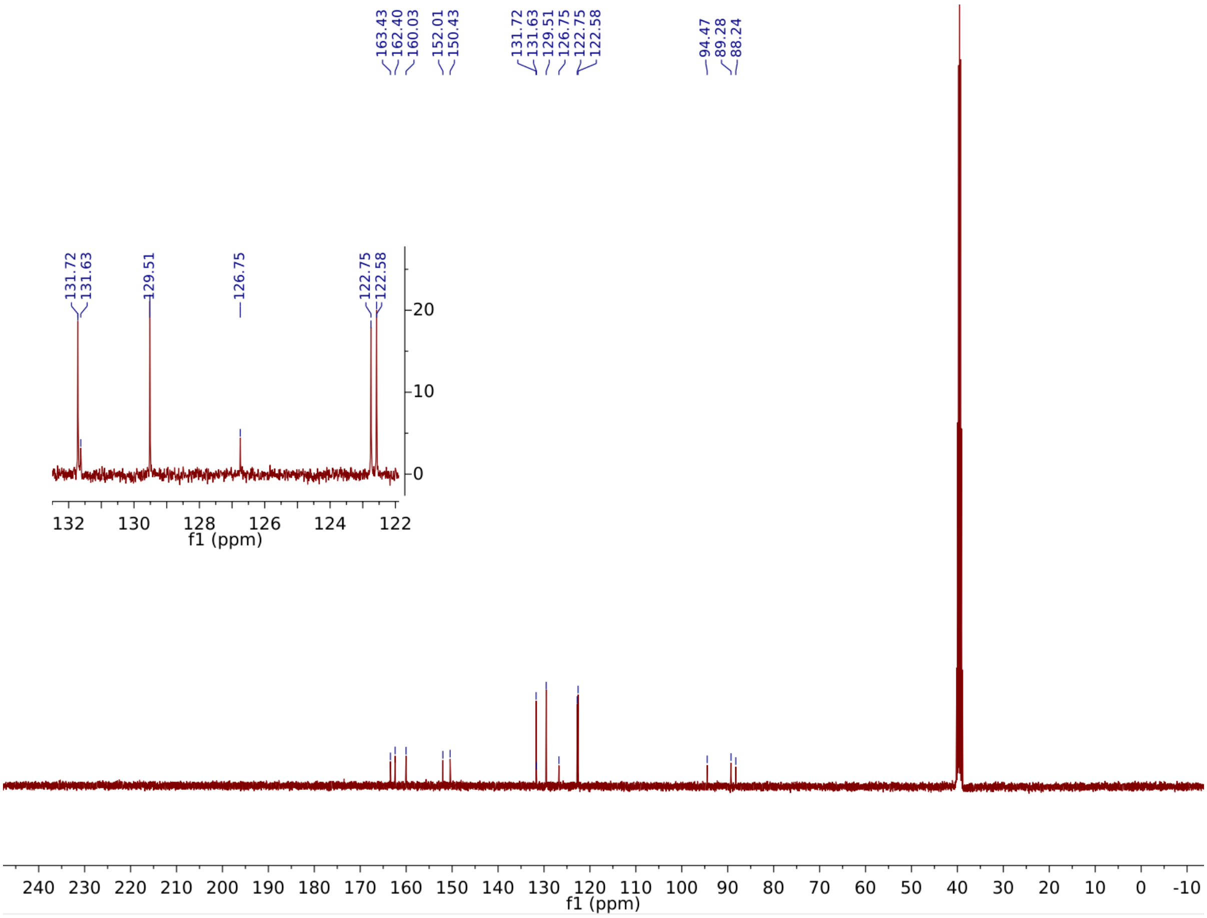

Compound 4 (HRMS)

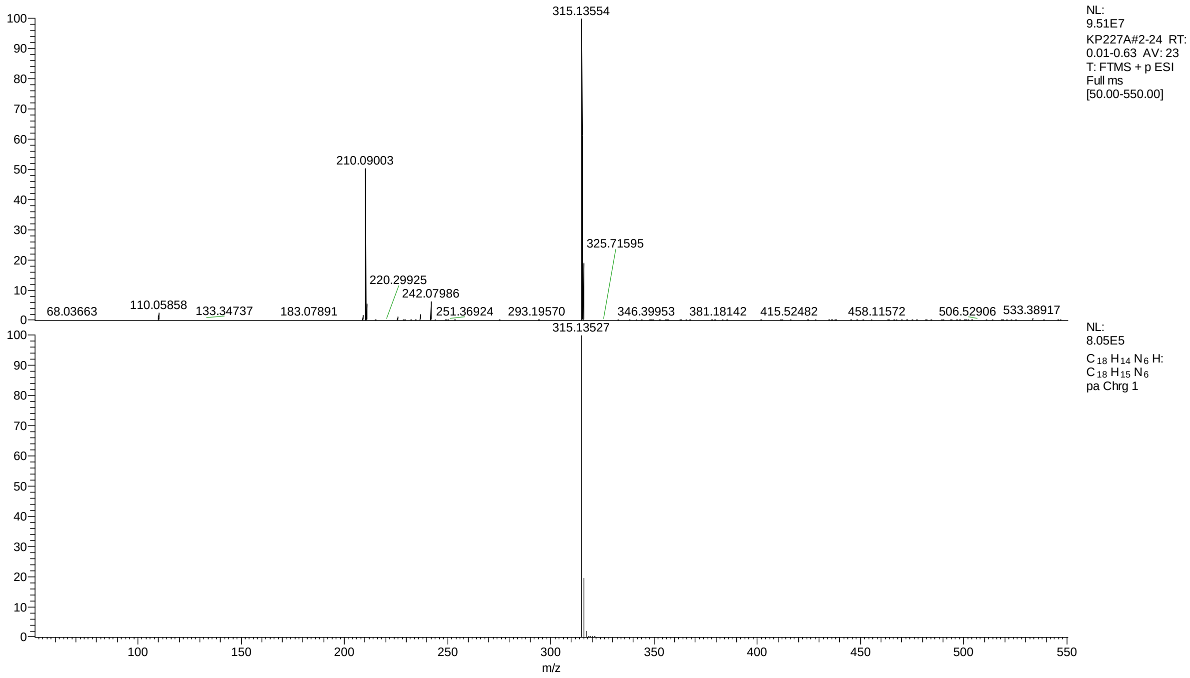

Compound 5 (^{1}H NMR, 400 MHz, DMSO-d_6)

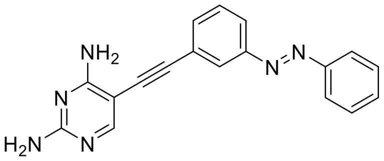

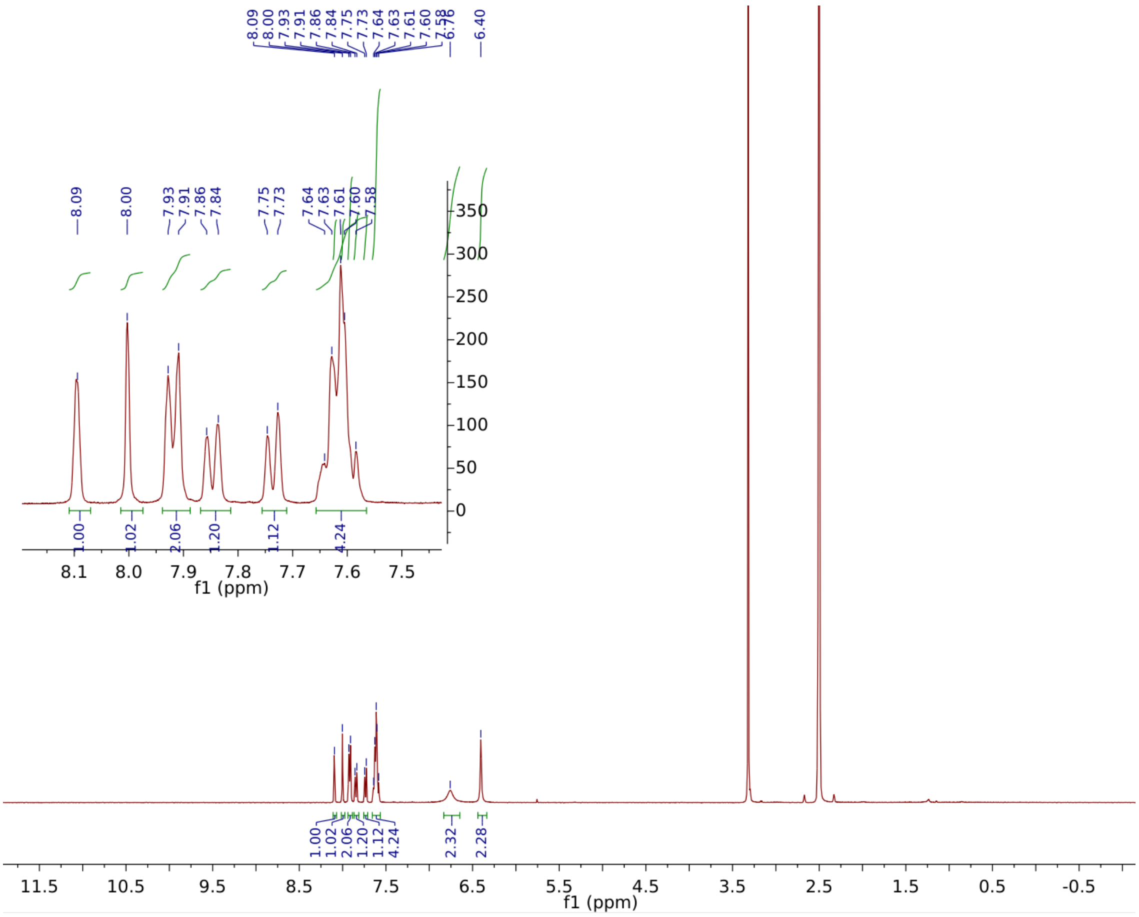

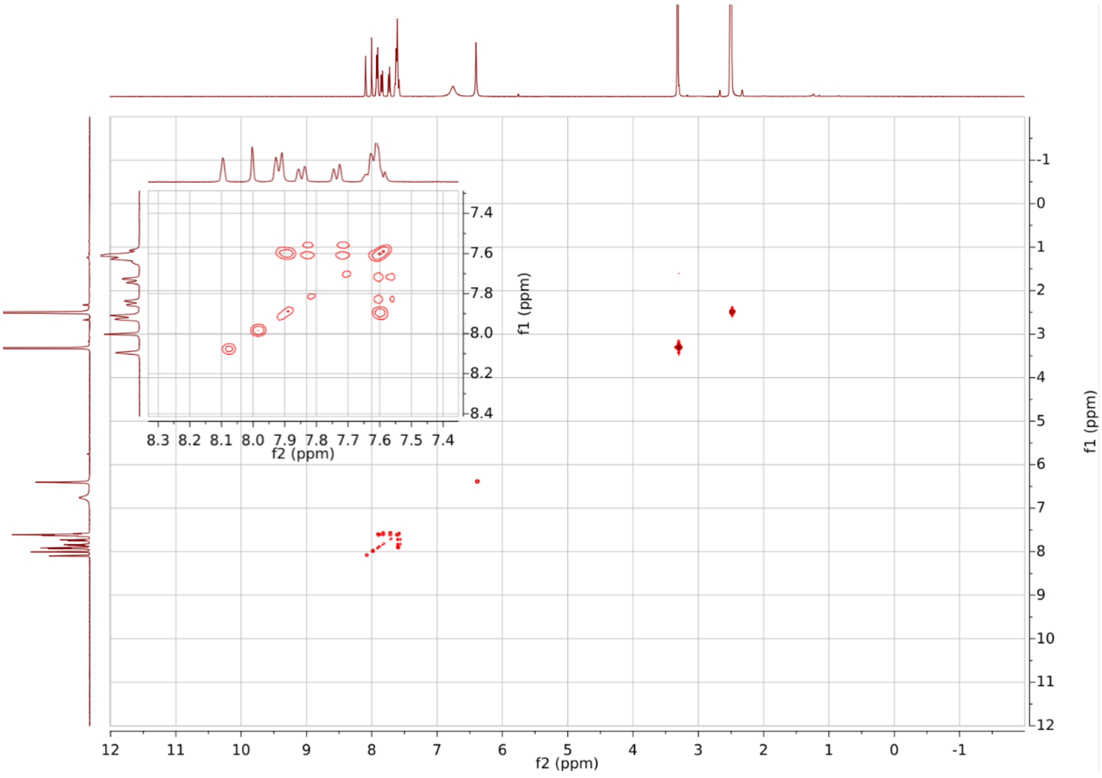

Compound 5 (^{13}C NMR, 101 MHz, DMSO-d_6)

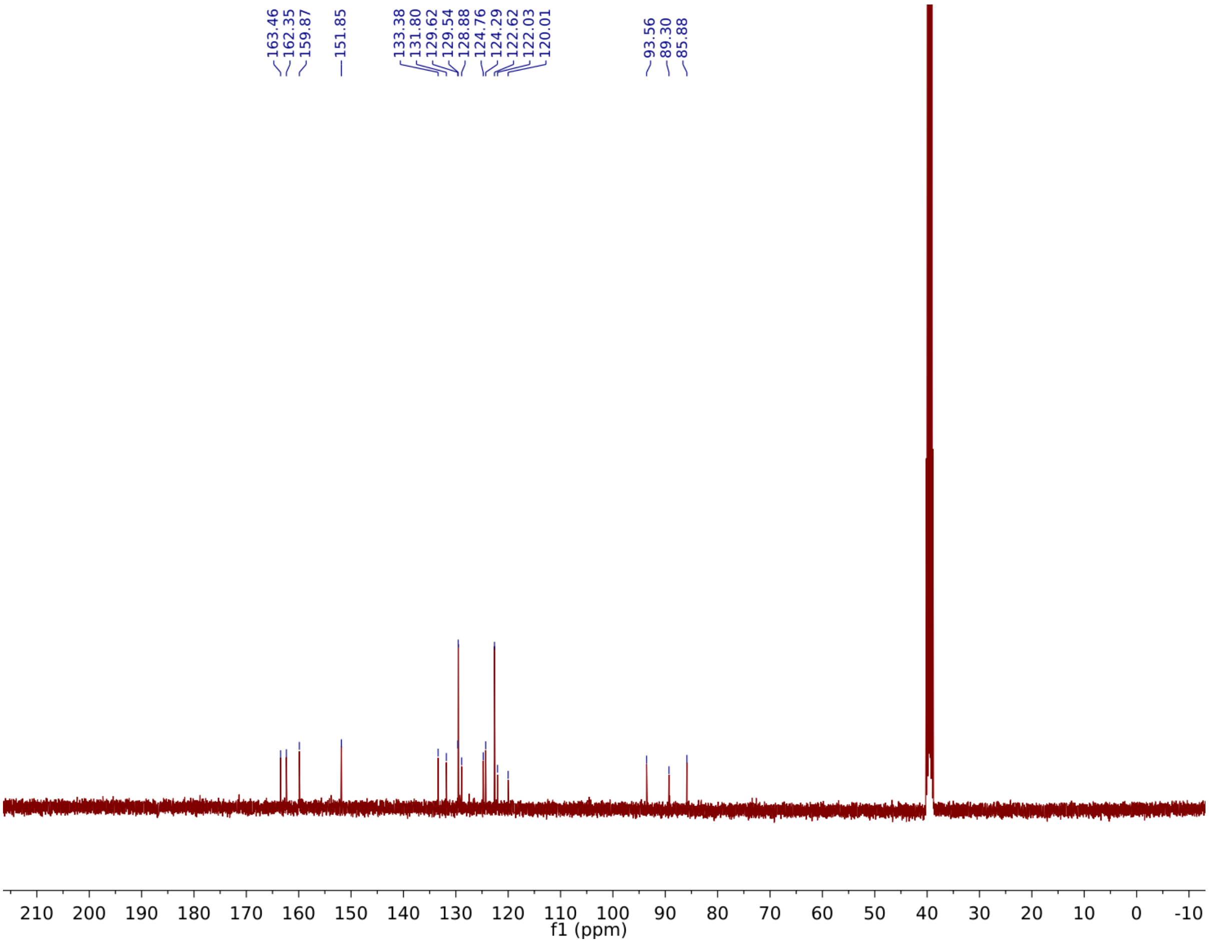

Compound 5 (HRMS)

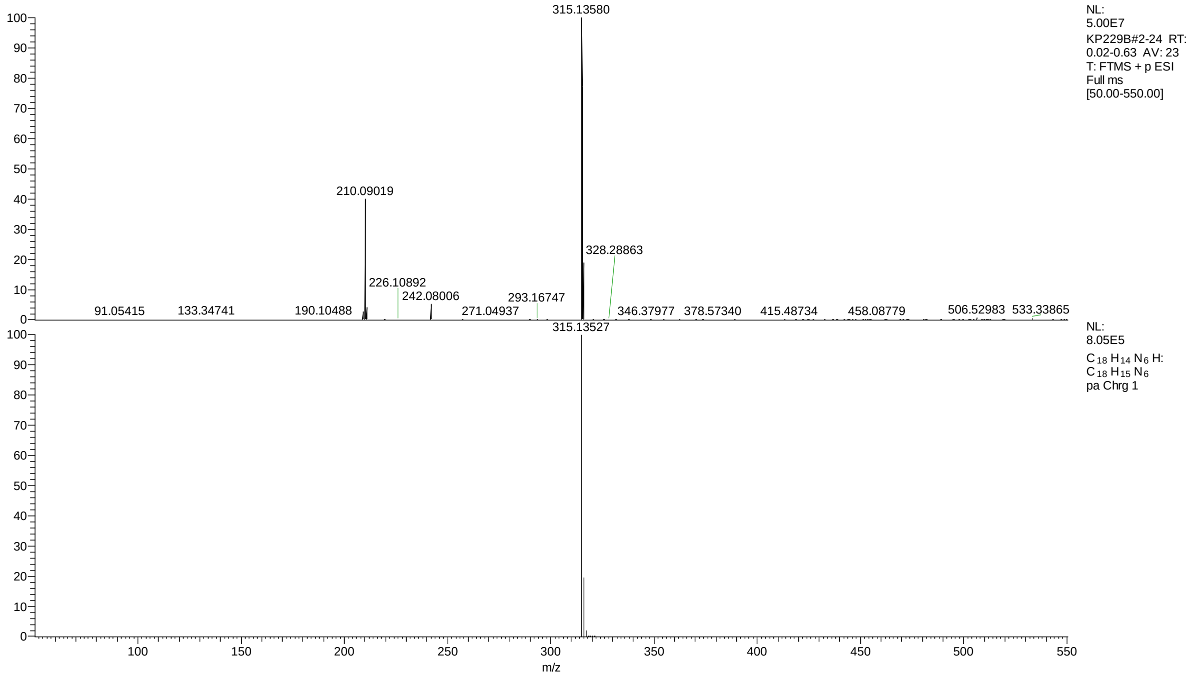

S7.2. Second hypothesis

Compound S8a (^{1}H NMR, 400 MHz, $CDCl_3$)

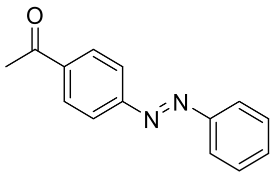

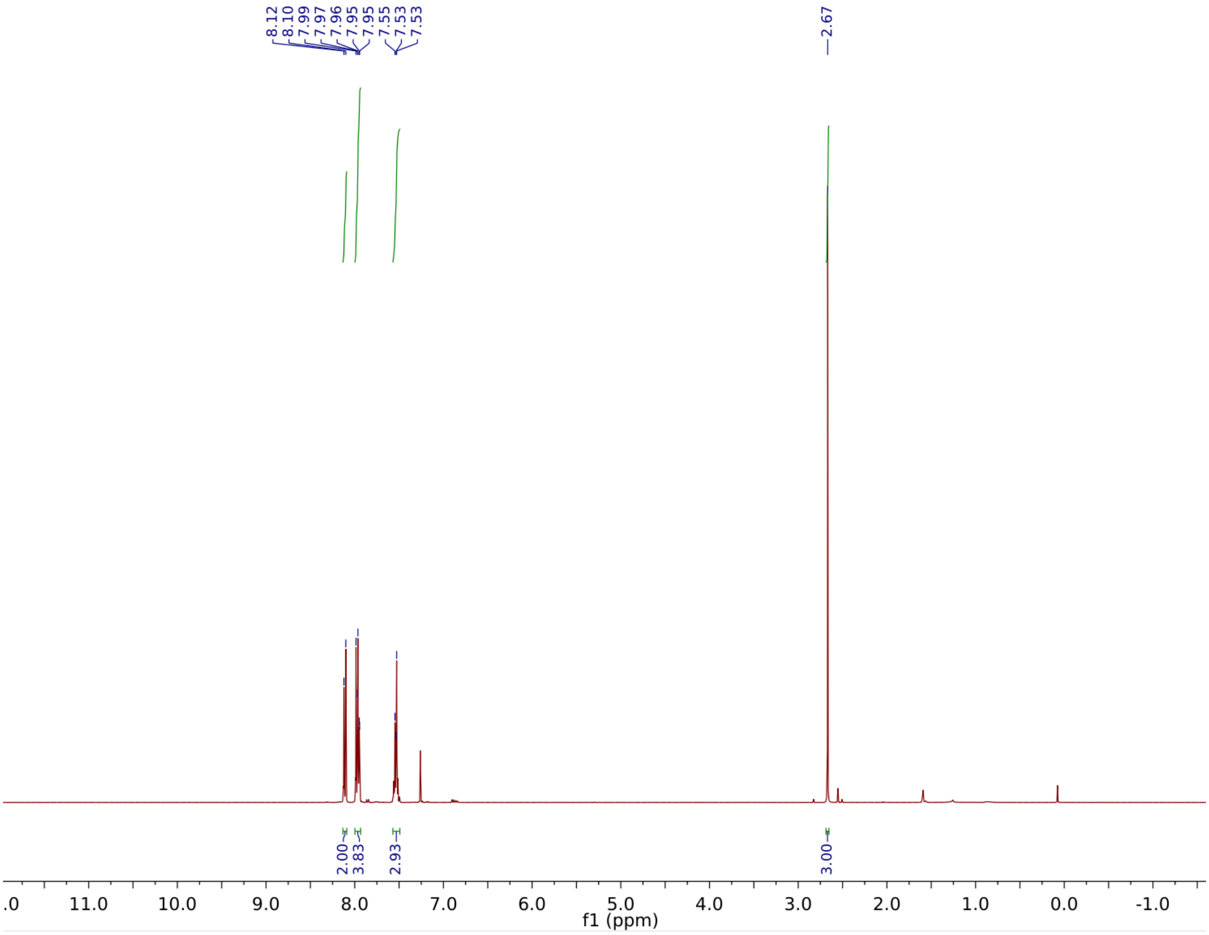

Compound S8b (^{1}H NMR, 400 MHz, $CDCl_3$)

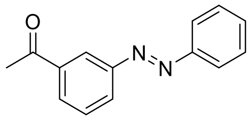

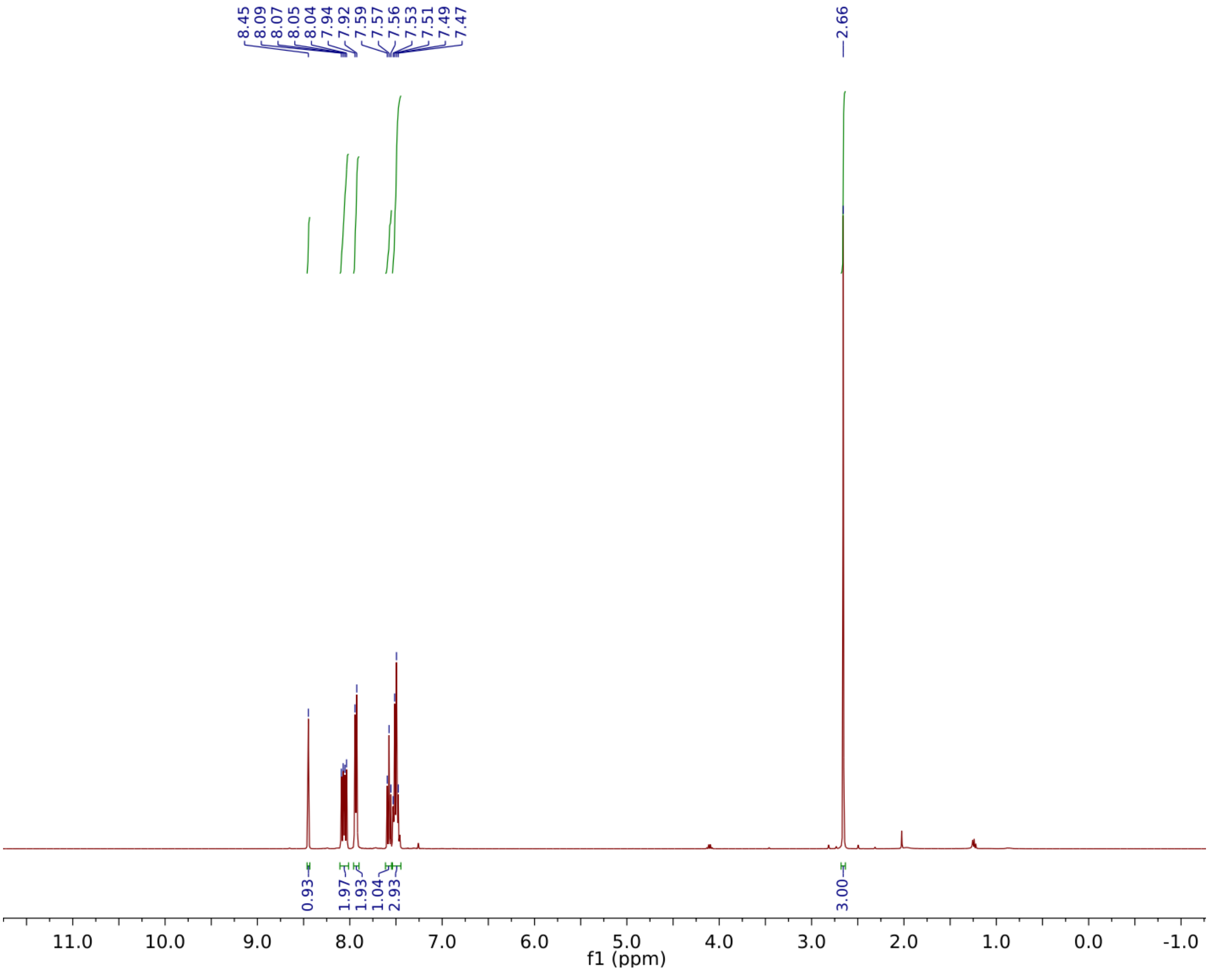

Compound S9a (^{1}H NMR, 400 MHz, DMSO-*d6*)

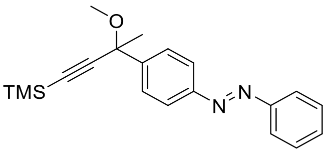

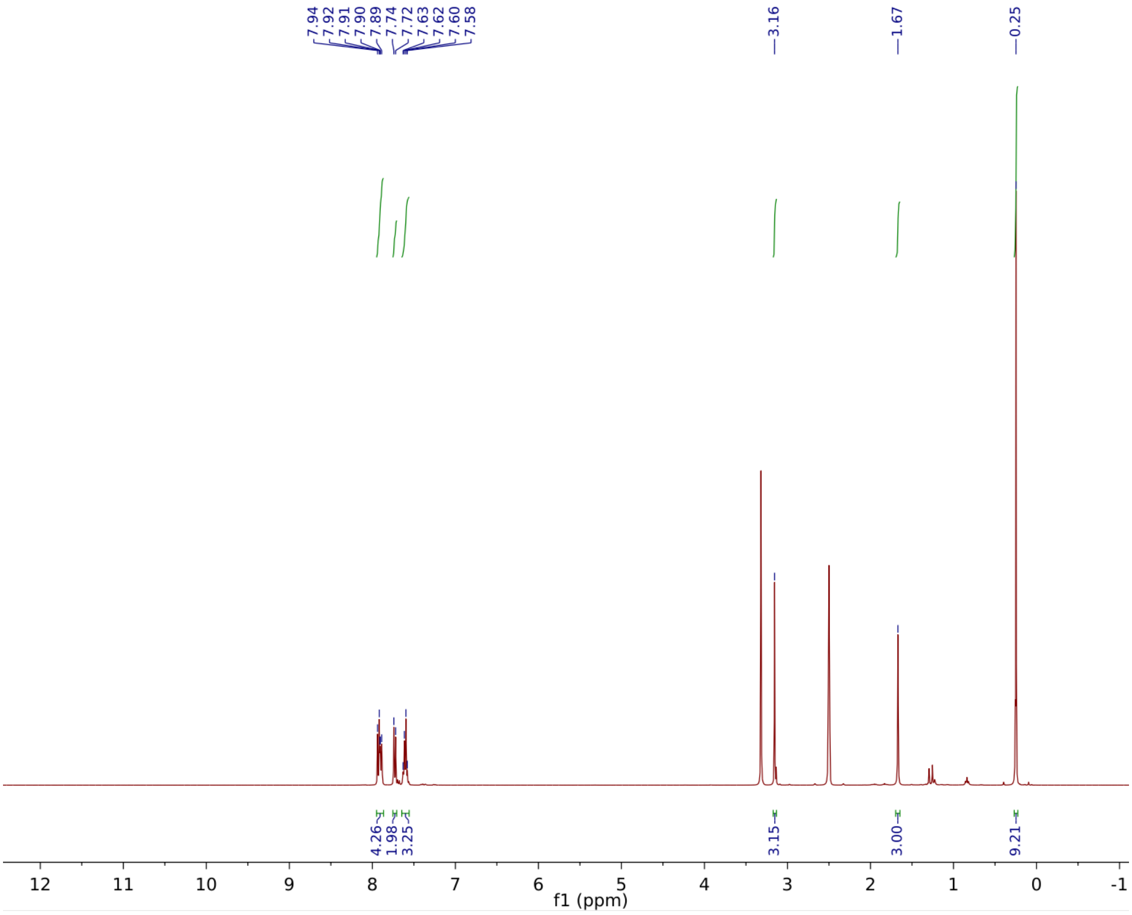

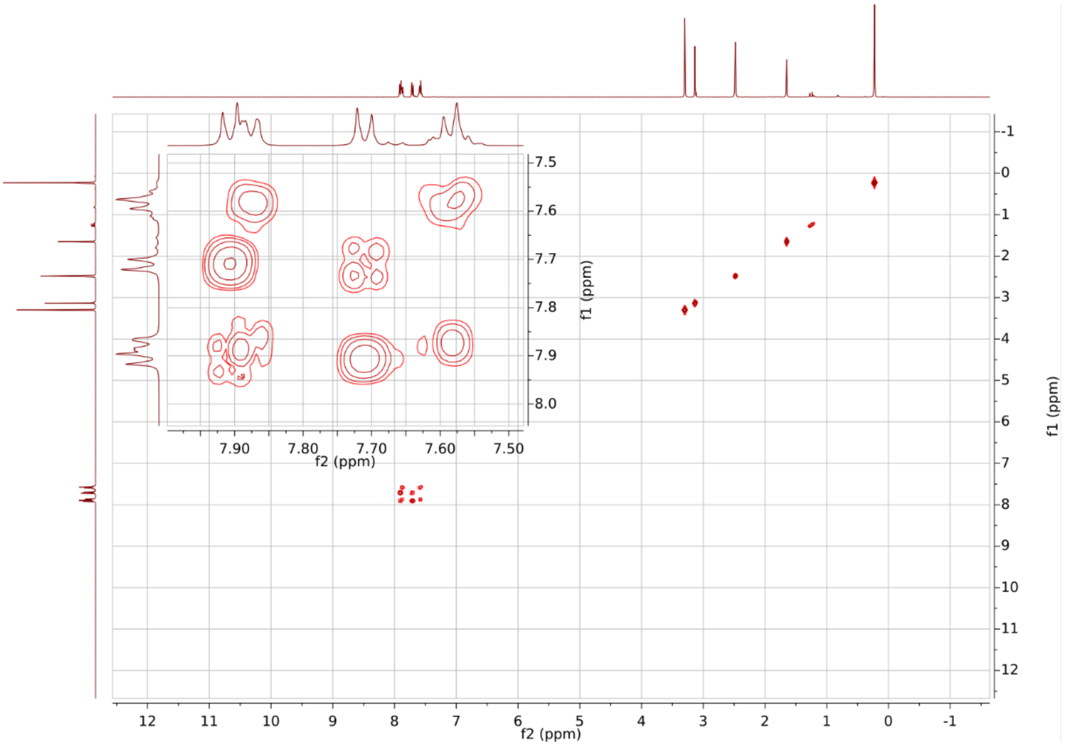

Compound S9a (^{13}C NMR, 101 MHz, DMSO-*d6*)

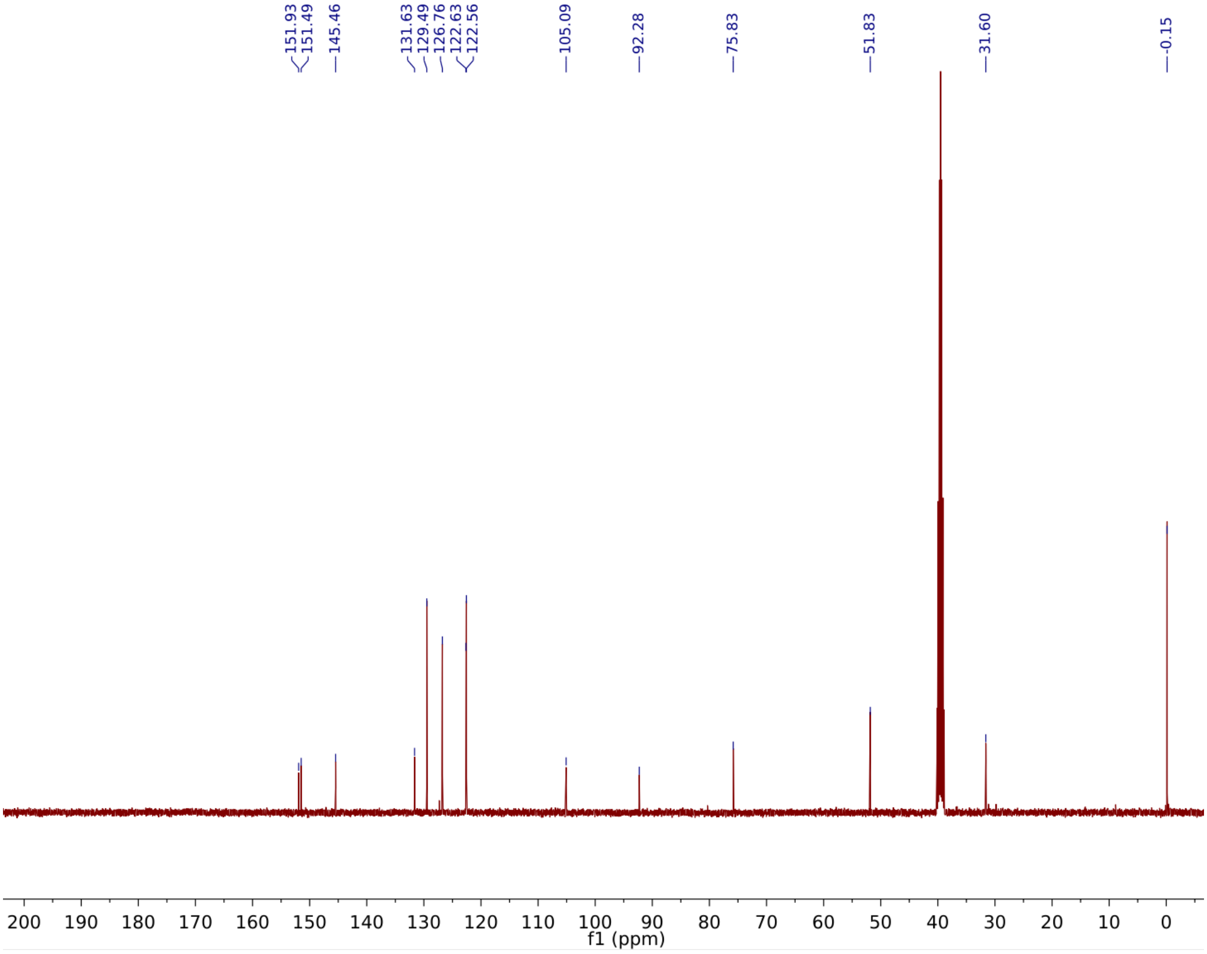

Compound S9b (^{1}H NMR, 400 MHz, $CDCl_3$)

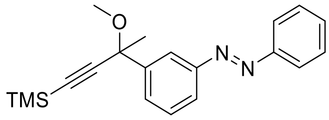

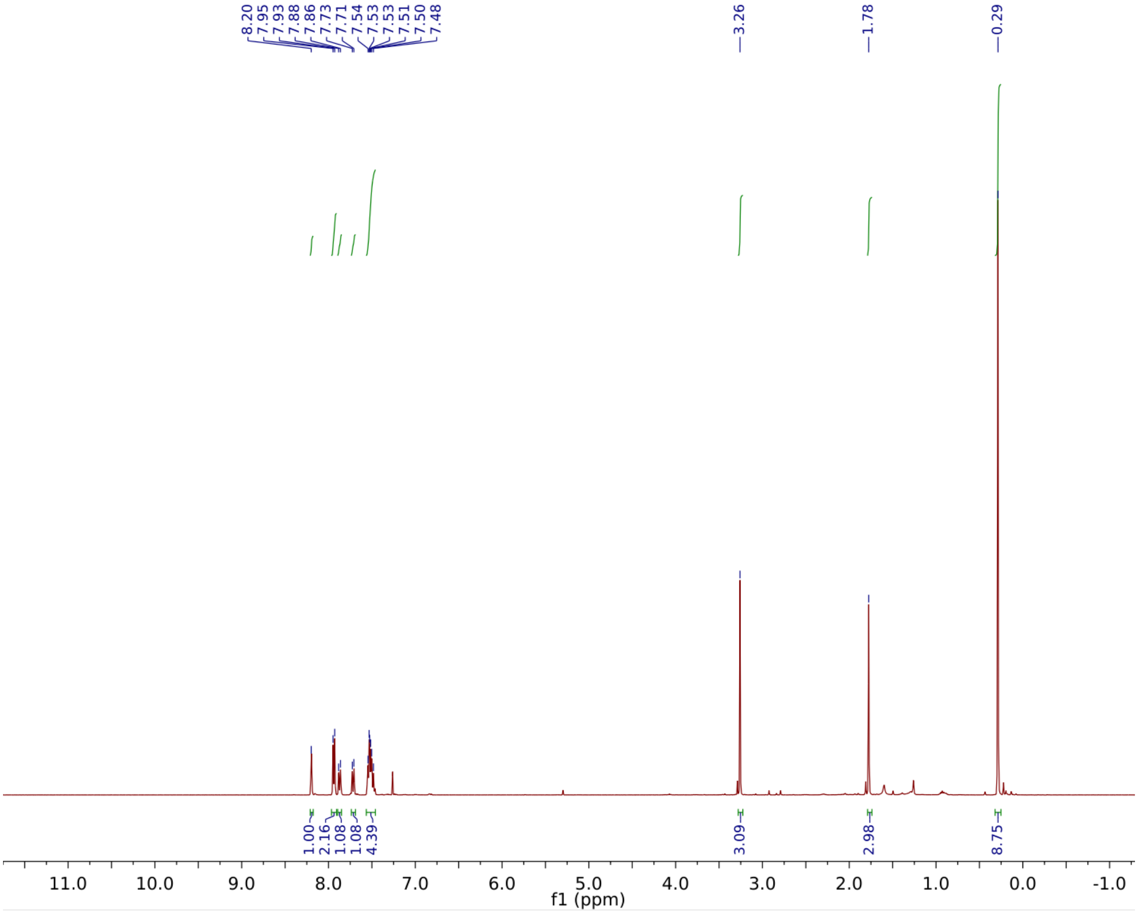

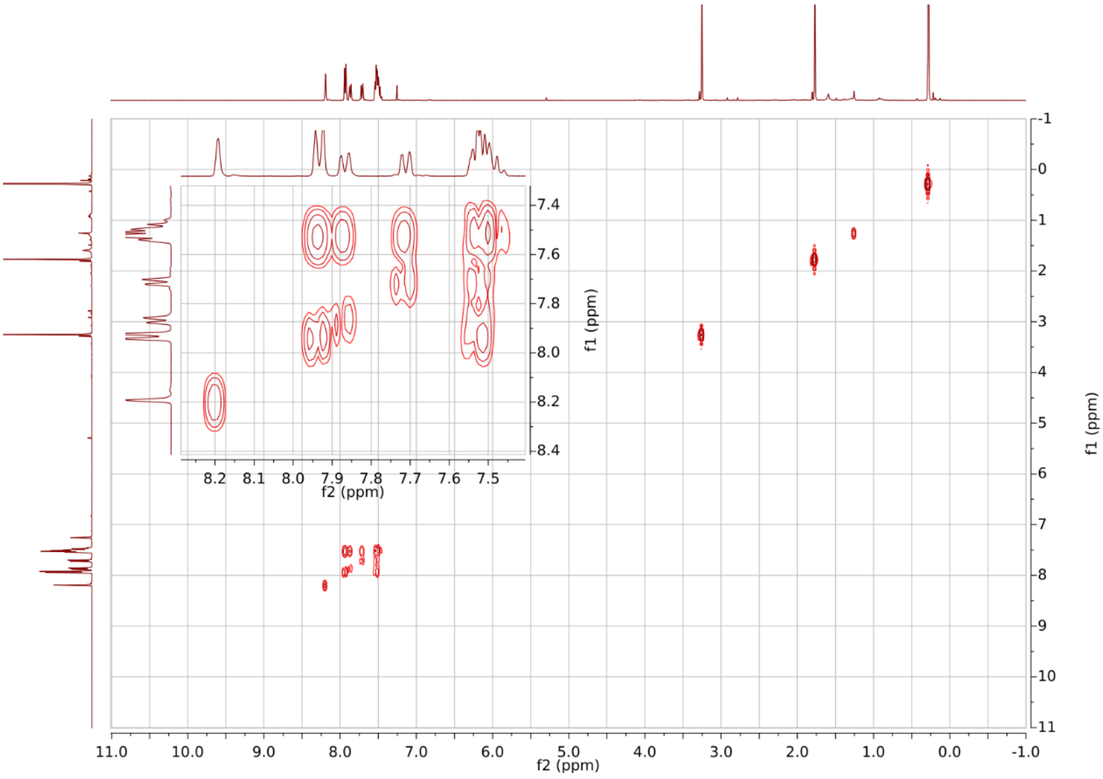

Compound S9b (^{13}C NMR, 101 MHz, $CDCl_3$)

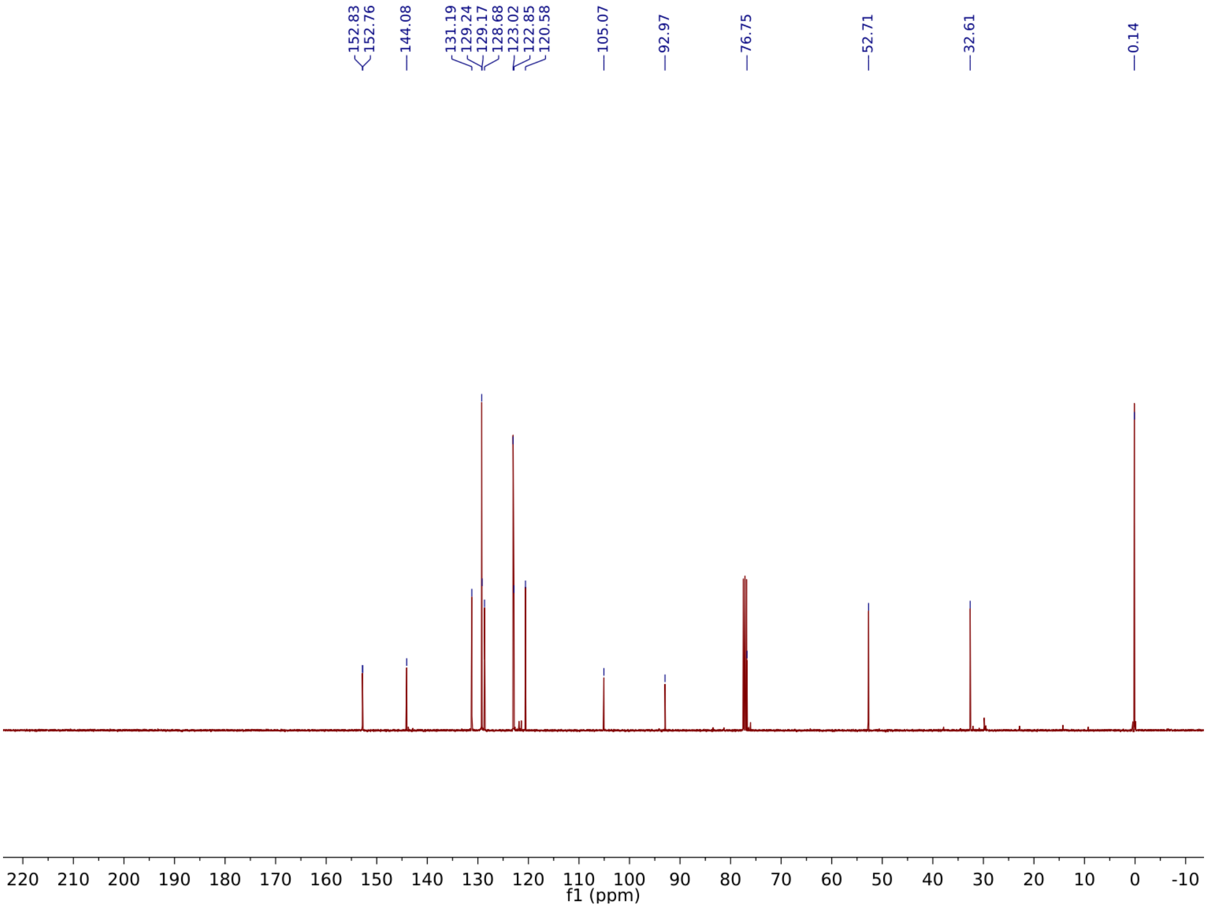

Compound S10a (^{1}H NMR, 400 MHz, DMSO-d_6)

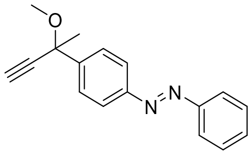

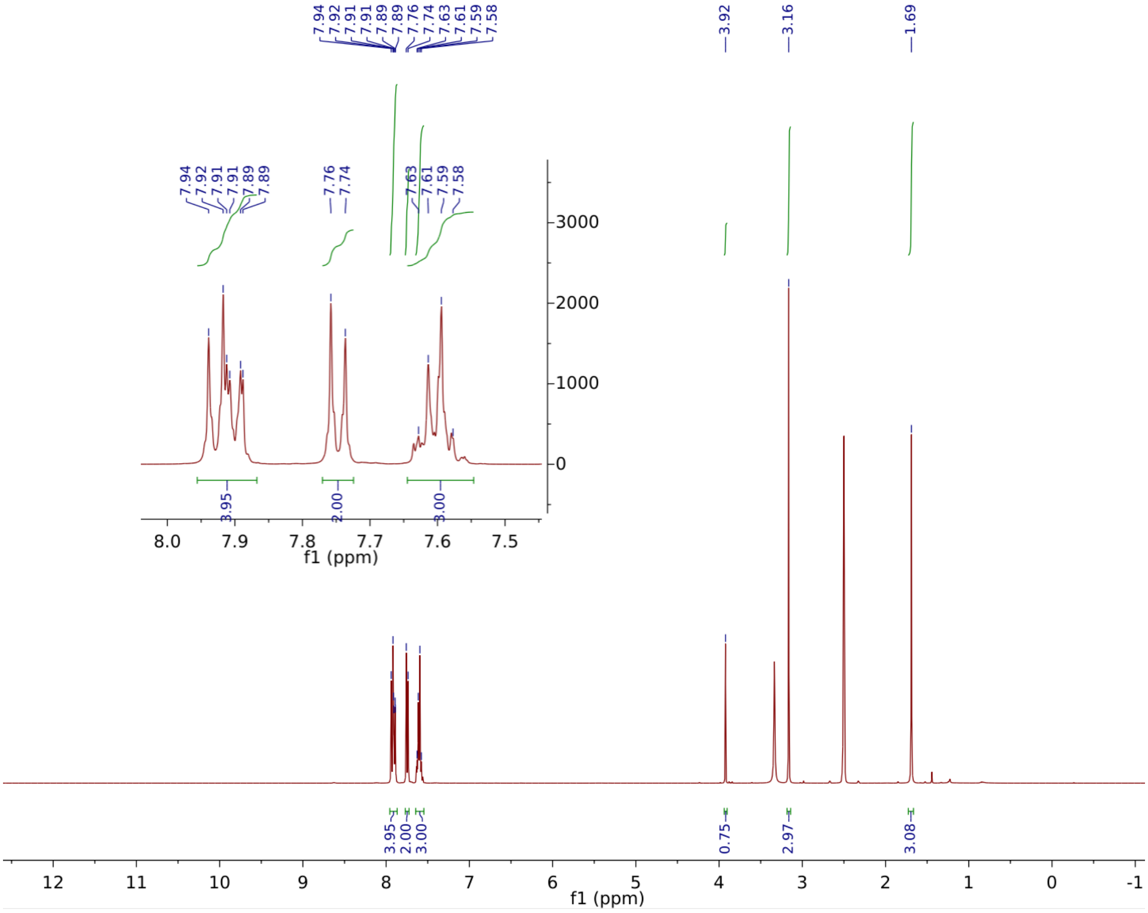

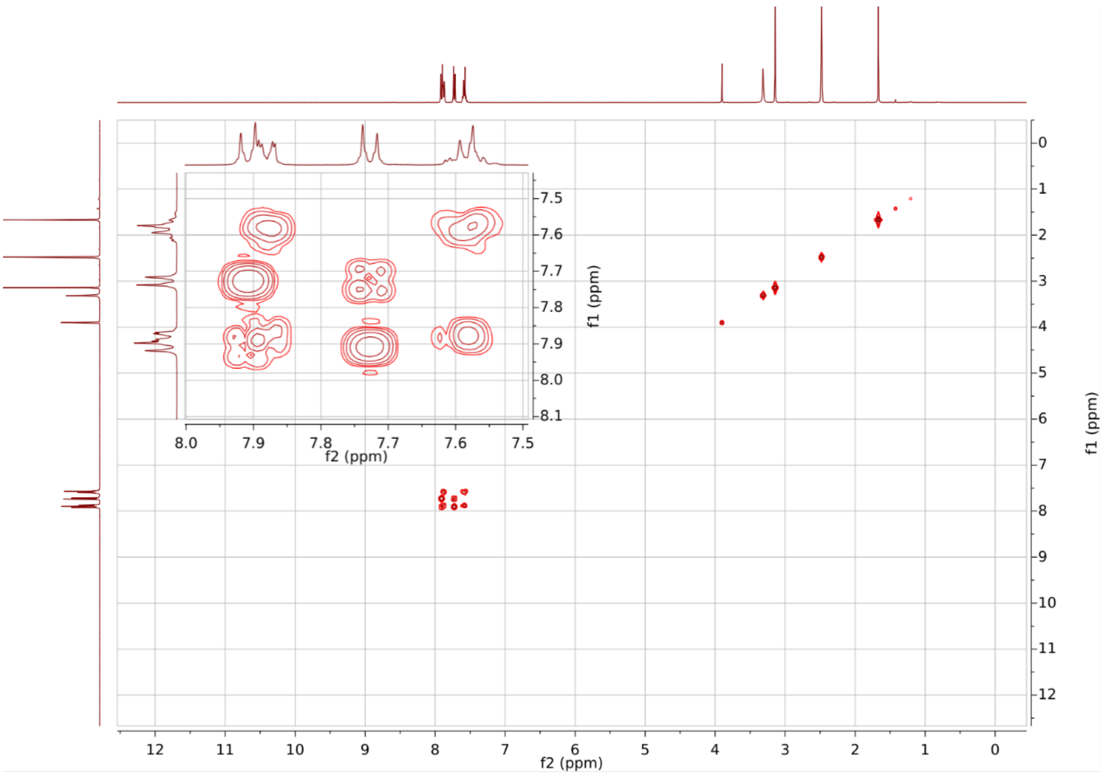

Compound S10a (^{13}C NMR, 101 MHz, DMSO-d_6)

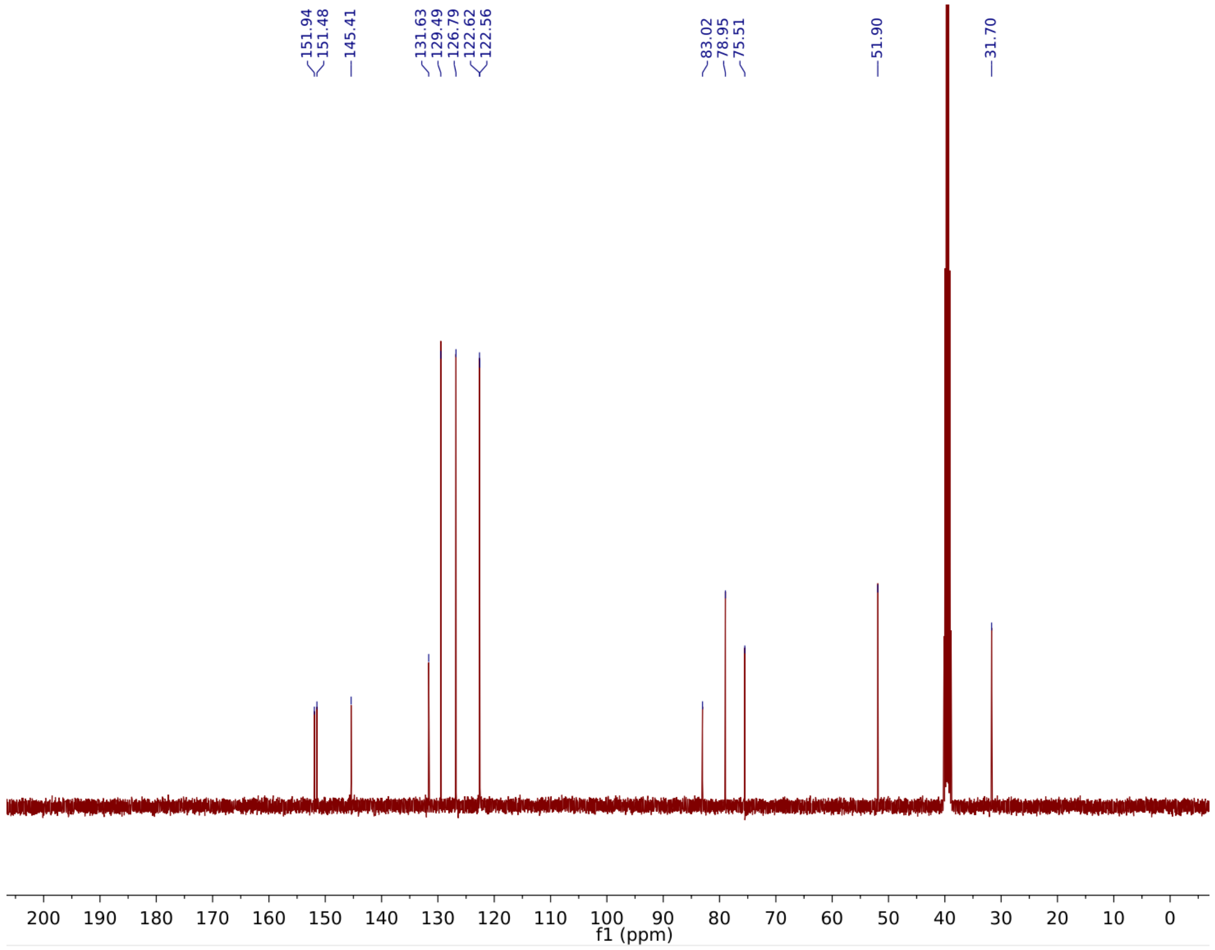

Compound S10b (^1H NMR, 400 MHz, $CDCl_3$)

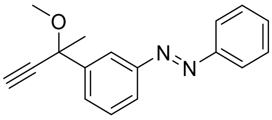

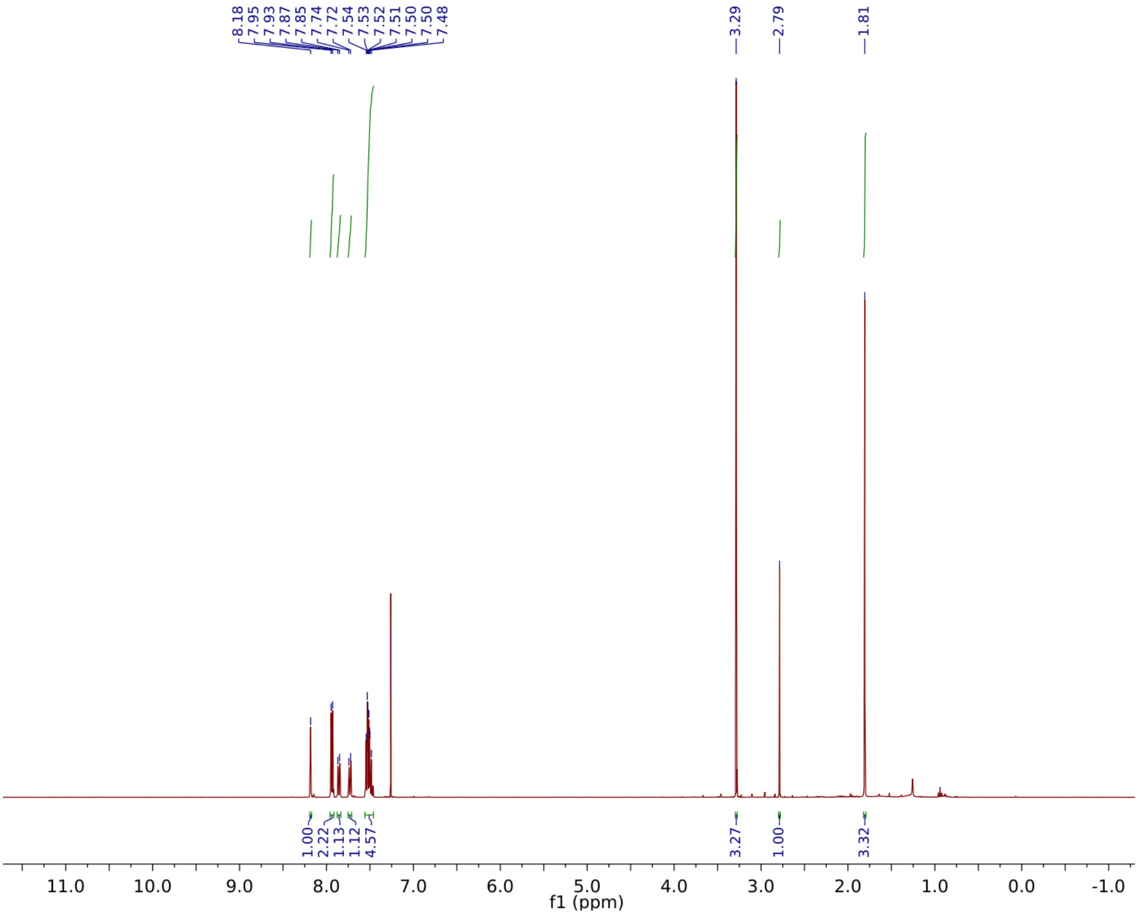

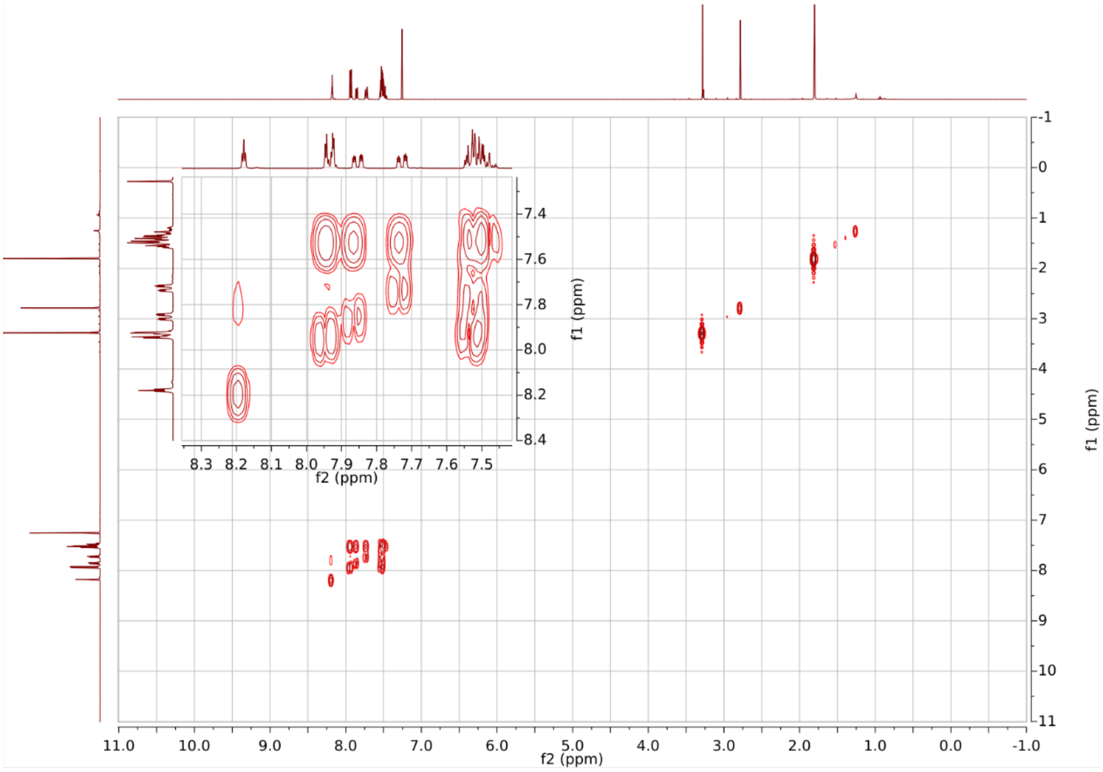

Compound S10b (^{13}C NMR, 101 MHz, $CDCl_3$)

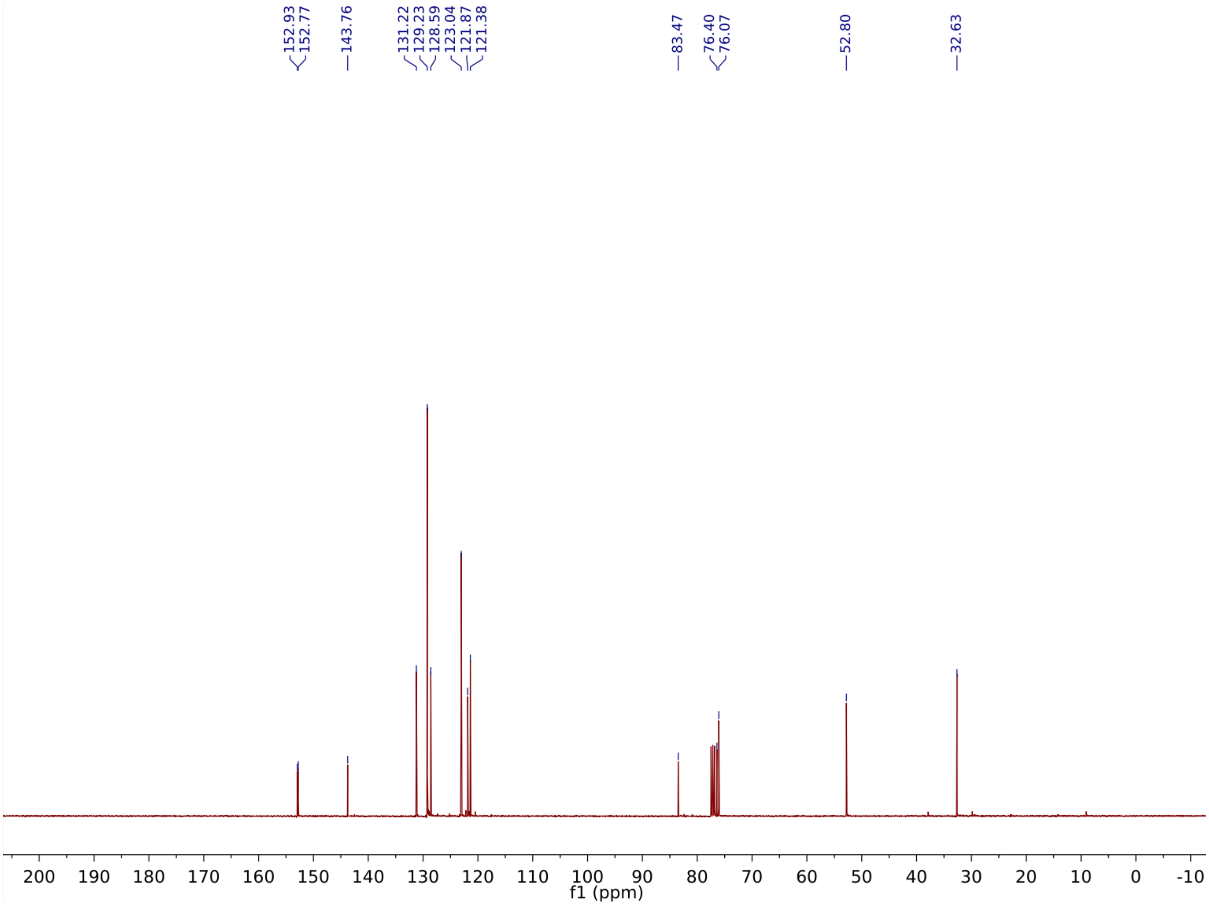

Compound 6 (^{1}H NMR, 400 MHz, DMSO-d_6)

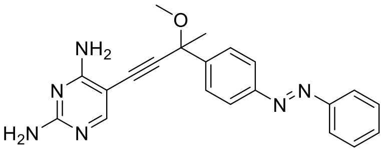

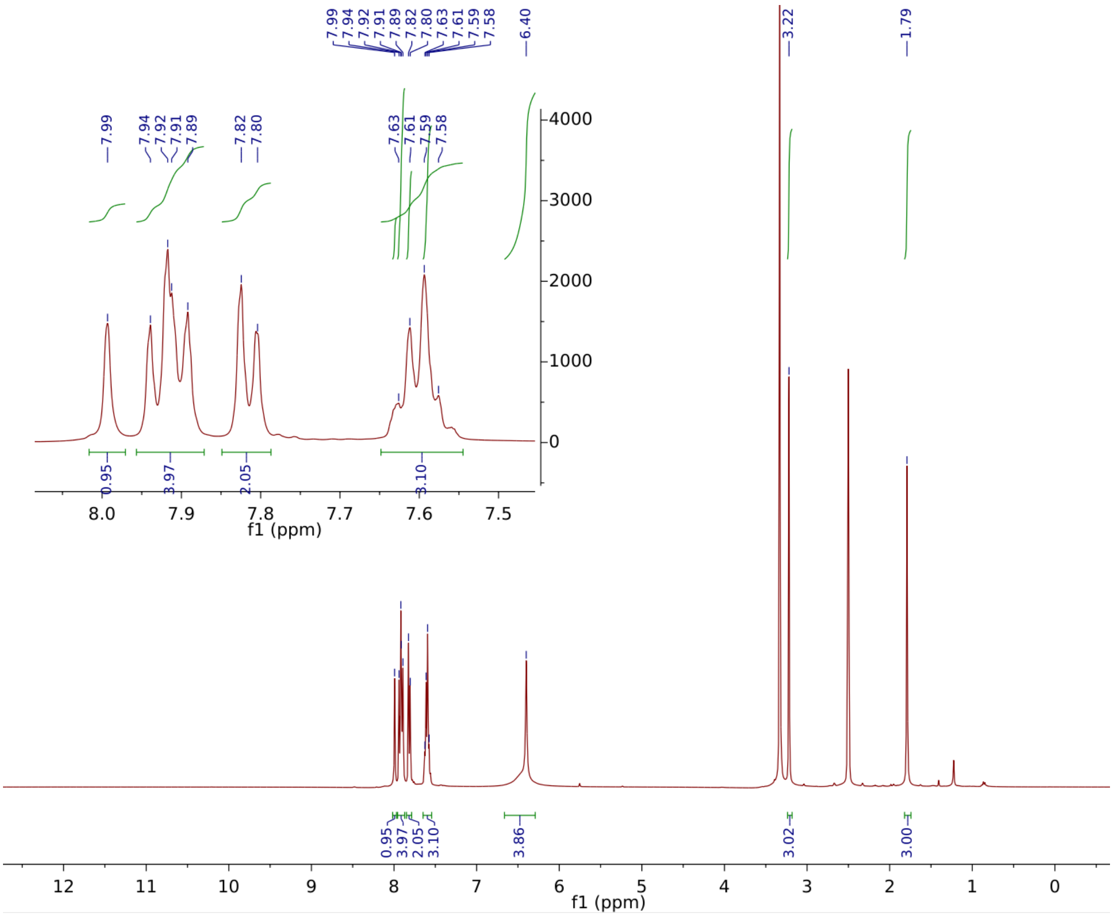

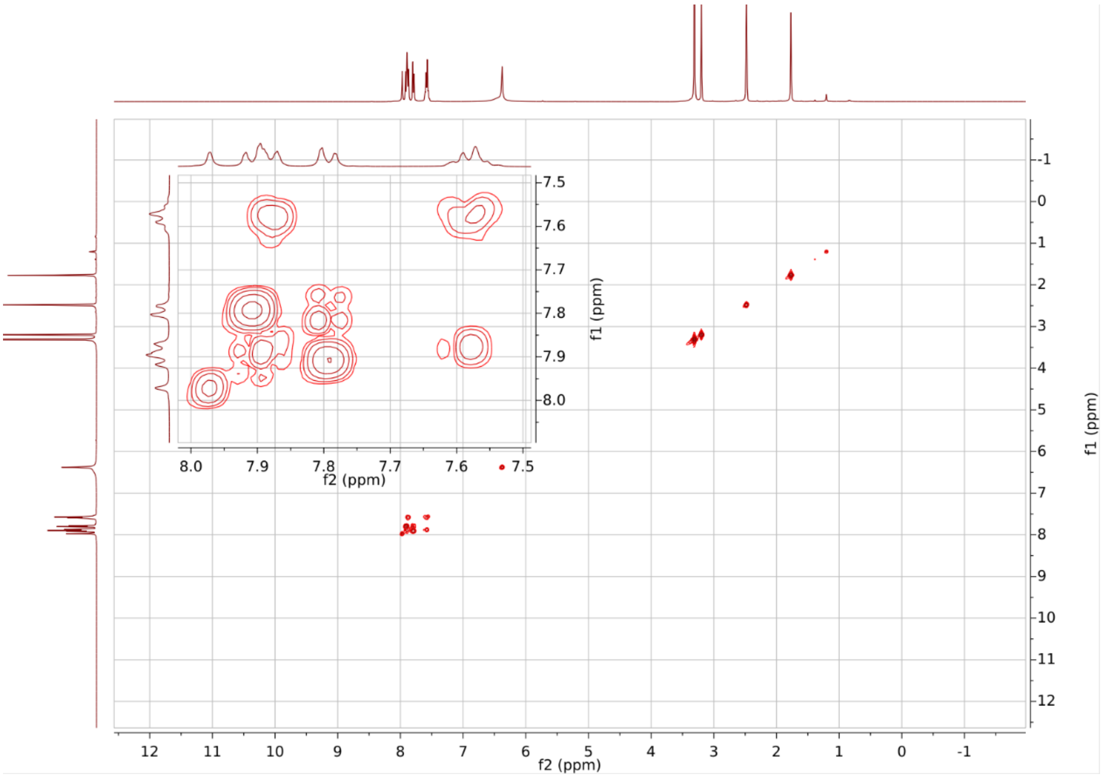

Compound 6 (^{13}C NMR, 101 MHz, DMSO-d_6)

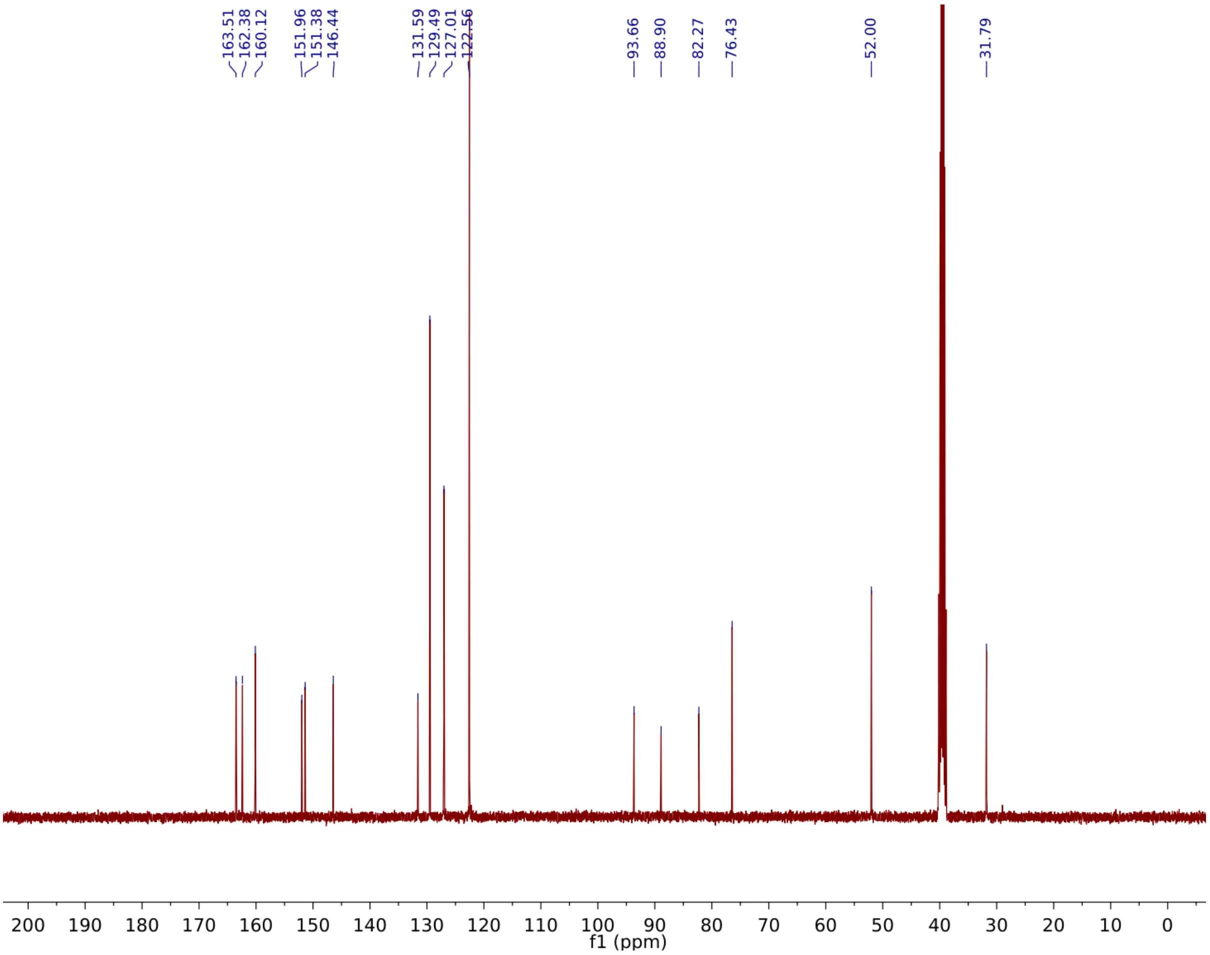

Compound 6 (HRMS)

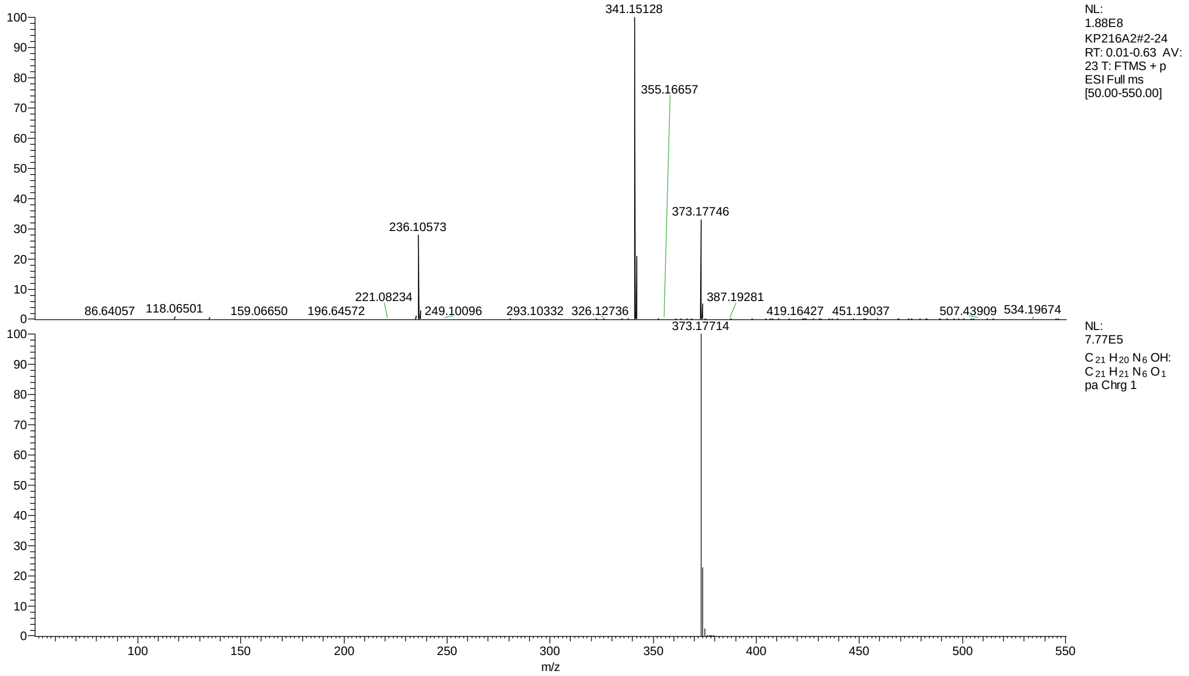

Compound 7 (^{1}H NMR, 400 MHz, DMSO-d_6)

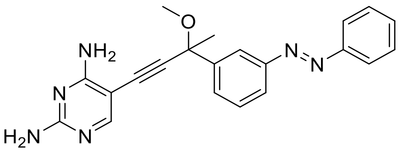

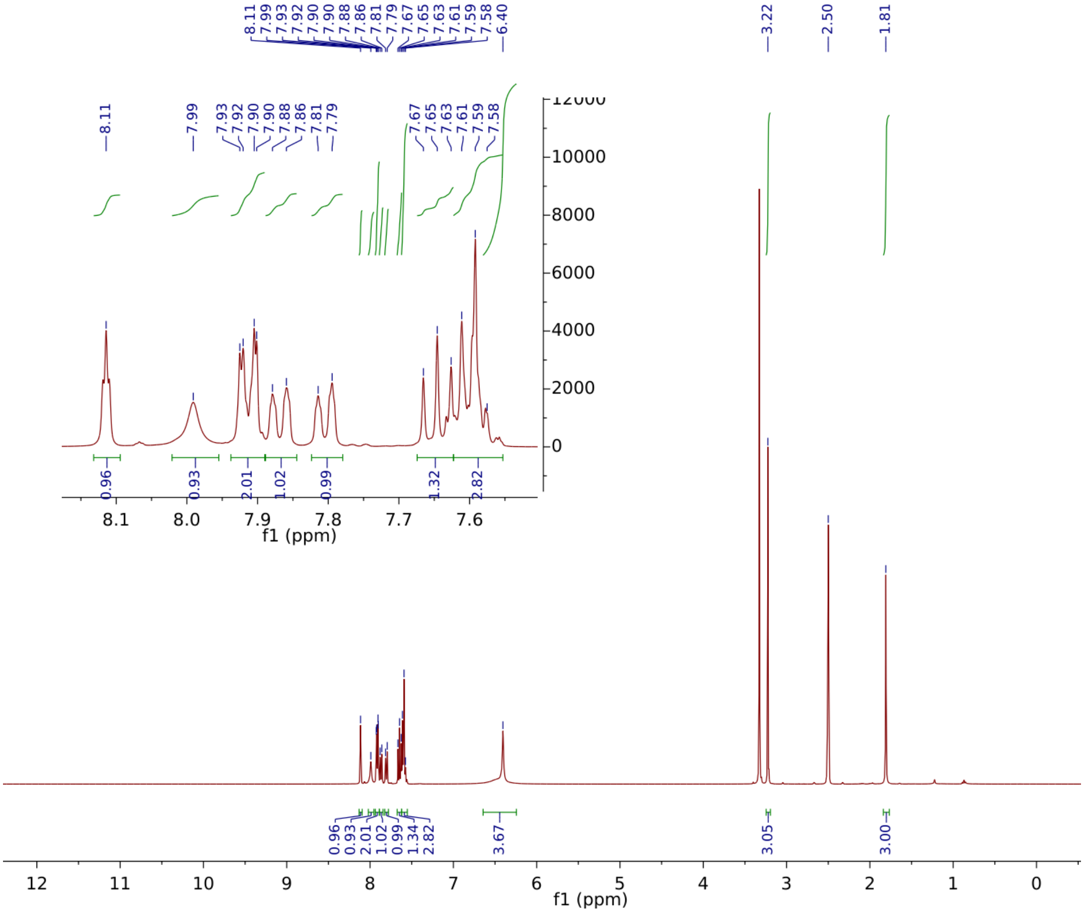

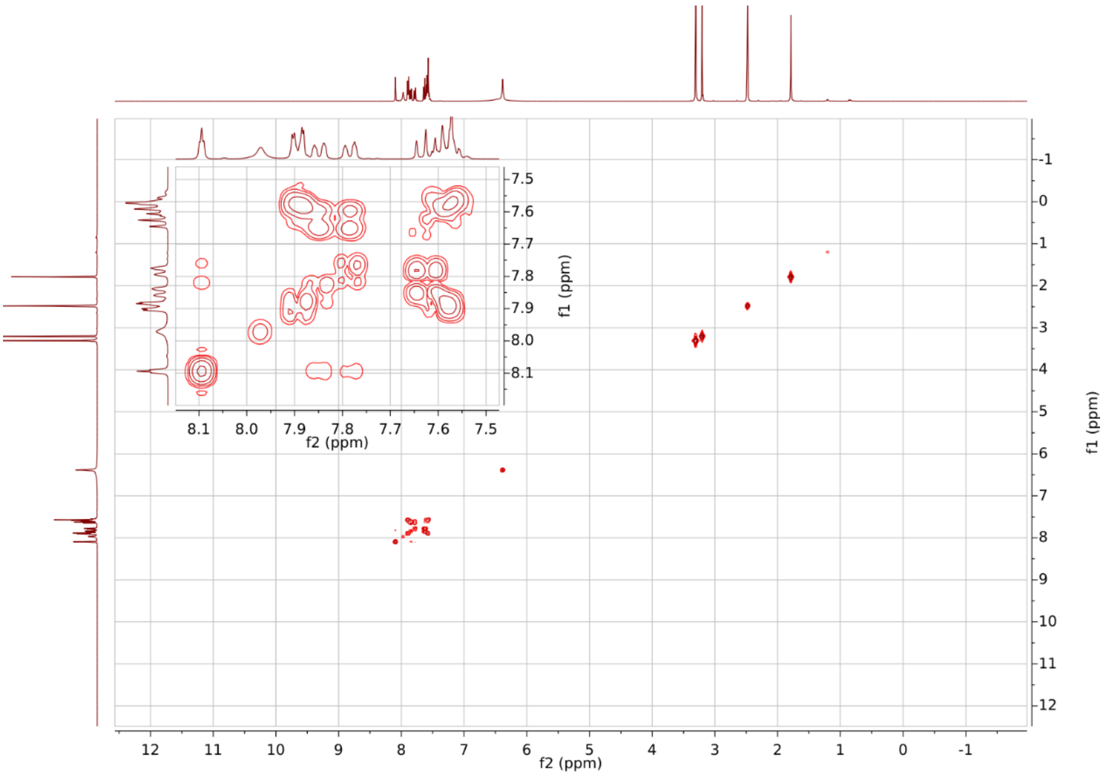

Compound 7 (^{13}C NMR, 101 MHz, DMSO-d_6)

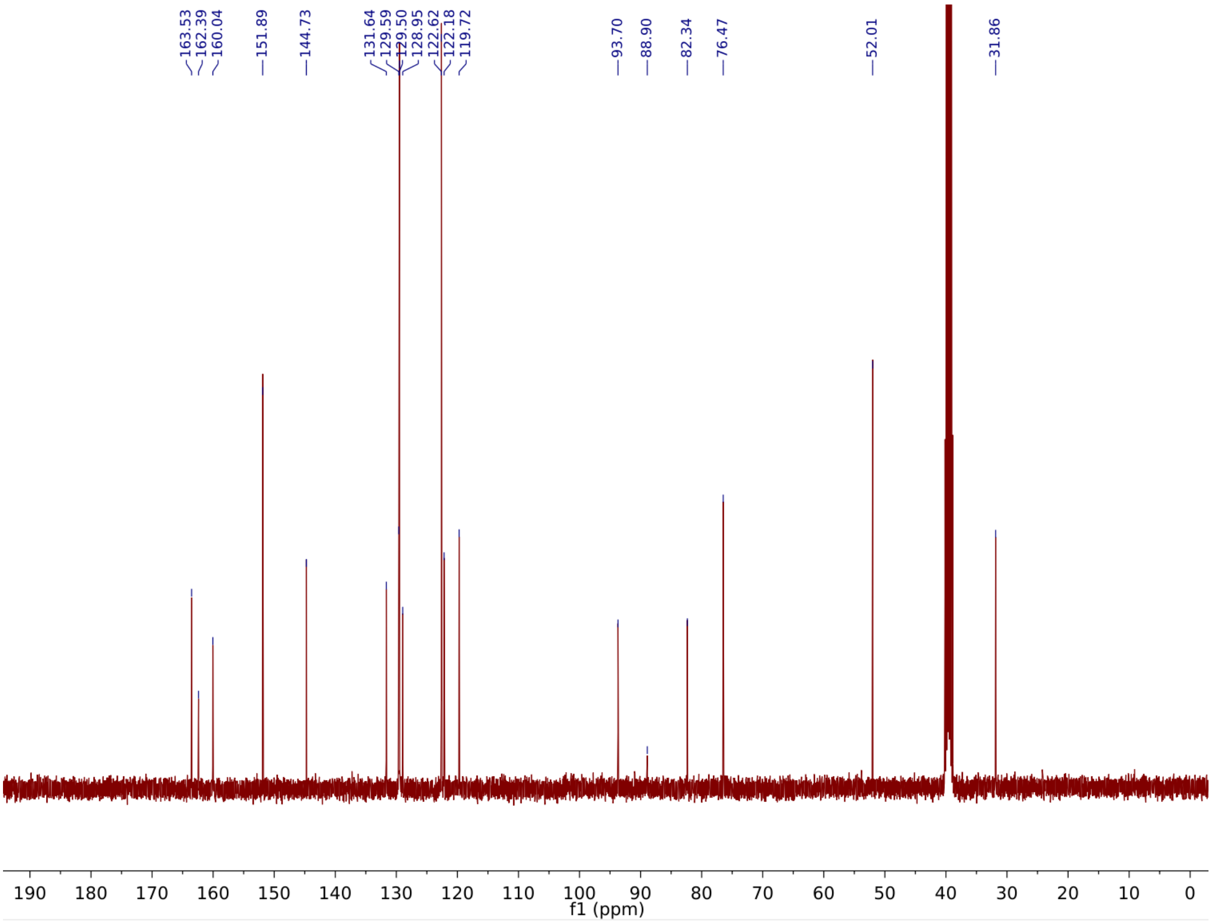

Compound 7 (HRMS)

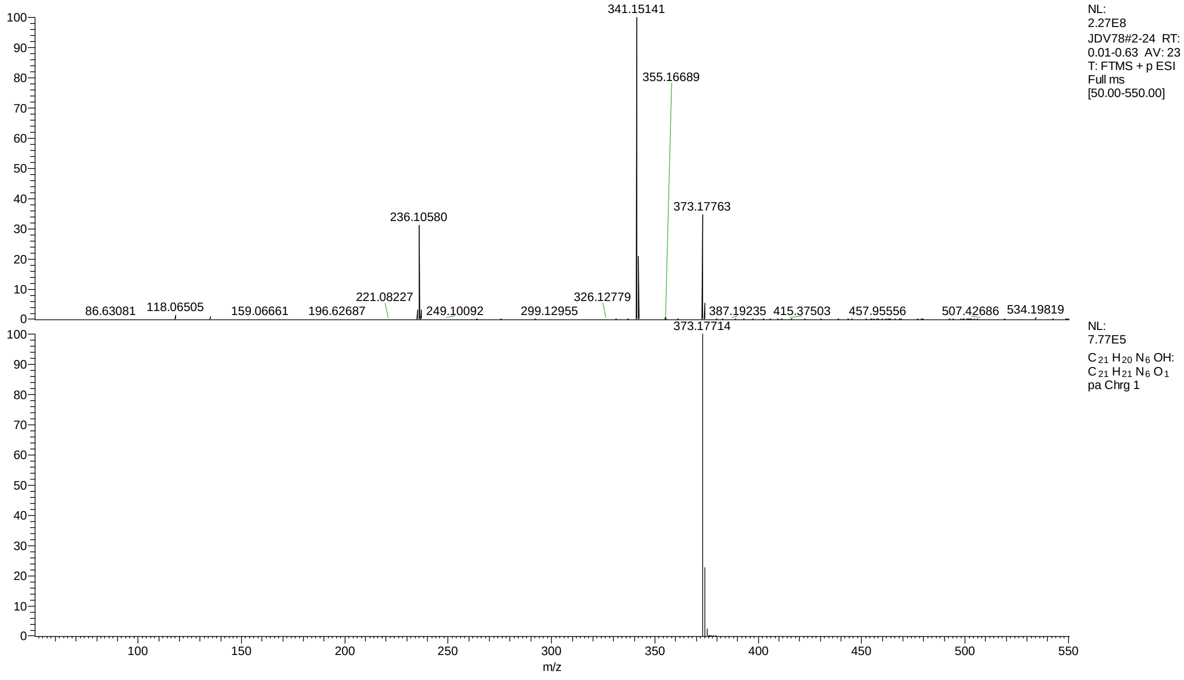

S7.3. Third hypothesis

Compound S11a (^{1}H NMR, 400 MHz, $CDCl_3$)

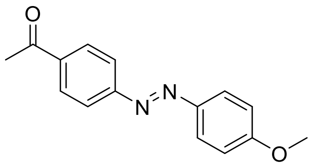

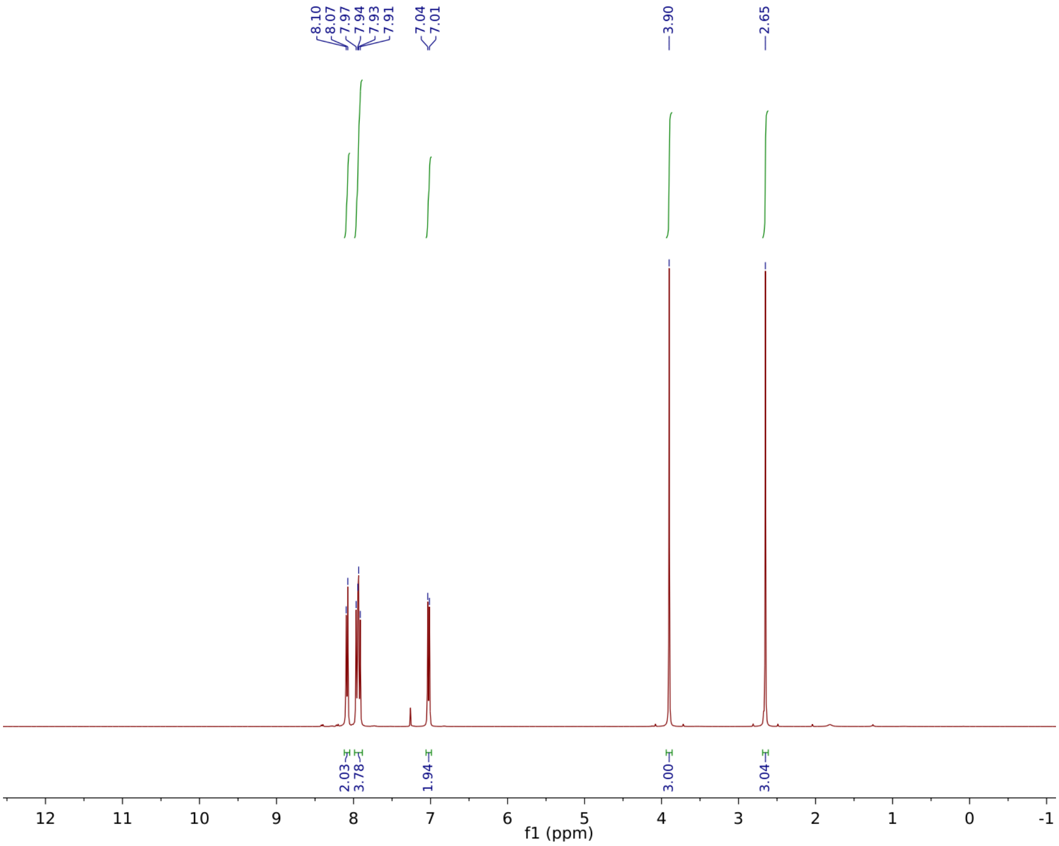

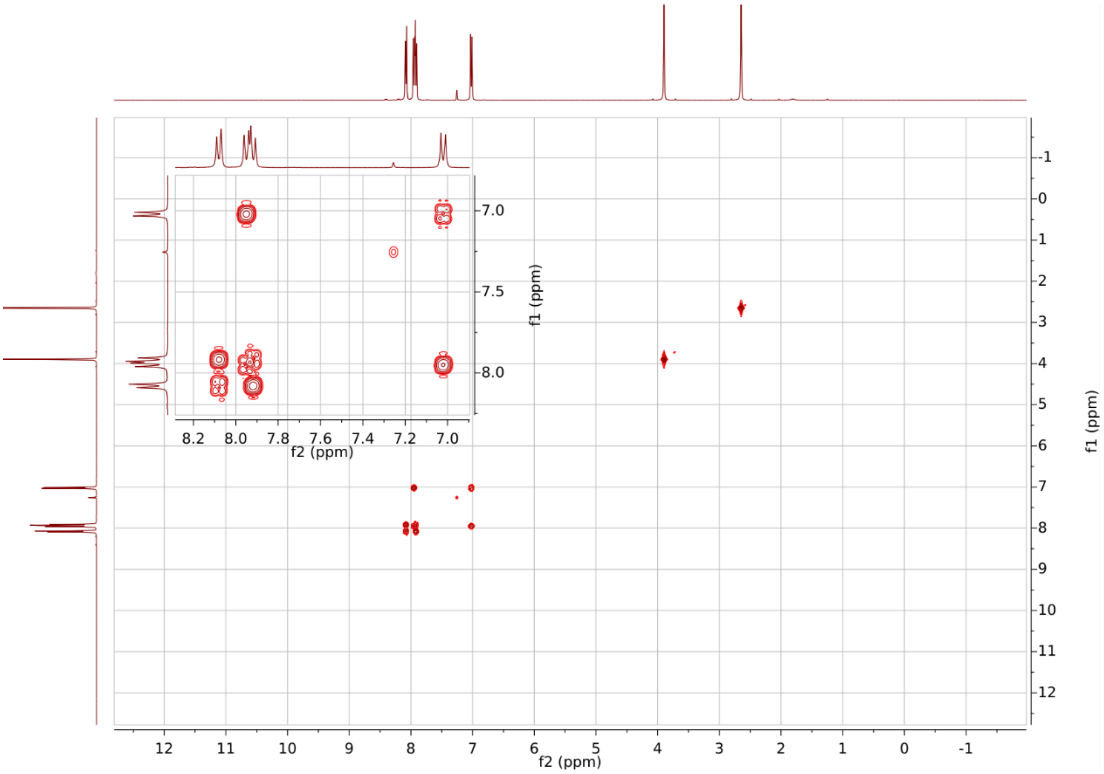

Compound S11a (^{13}C NMR, 101 MHz, $CDCl_3$)

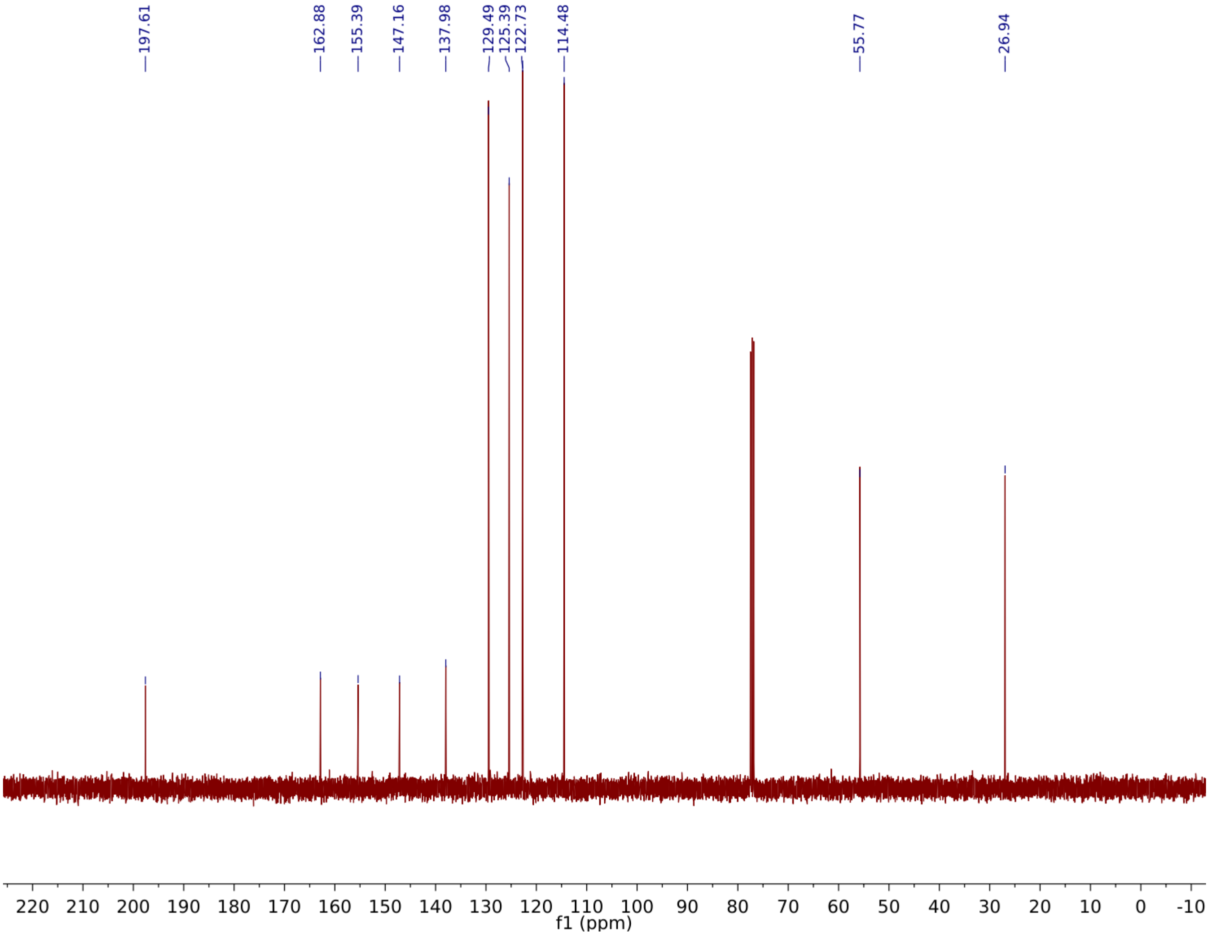

Compound S11b (^{1}H NMR, 400 MHz, DMSO-d_6)

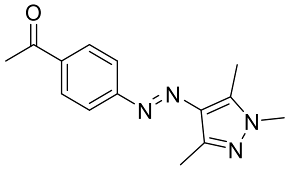

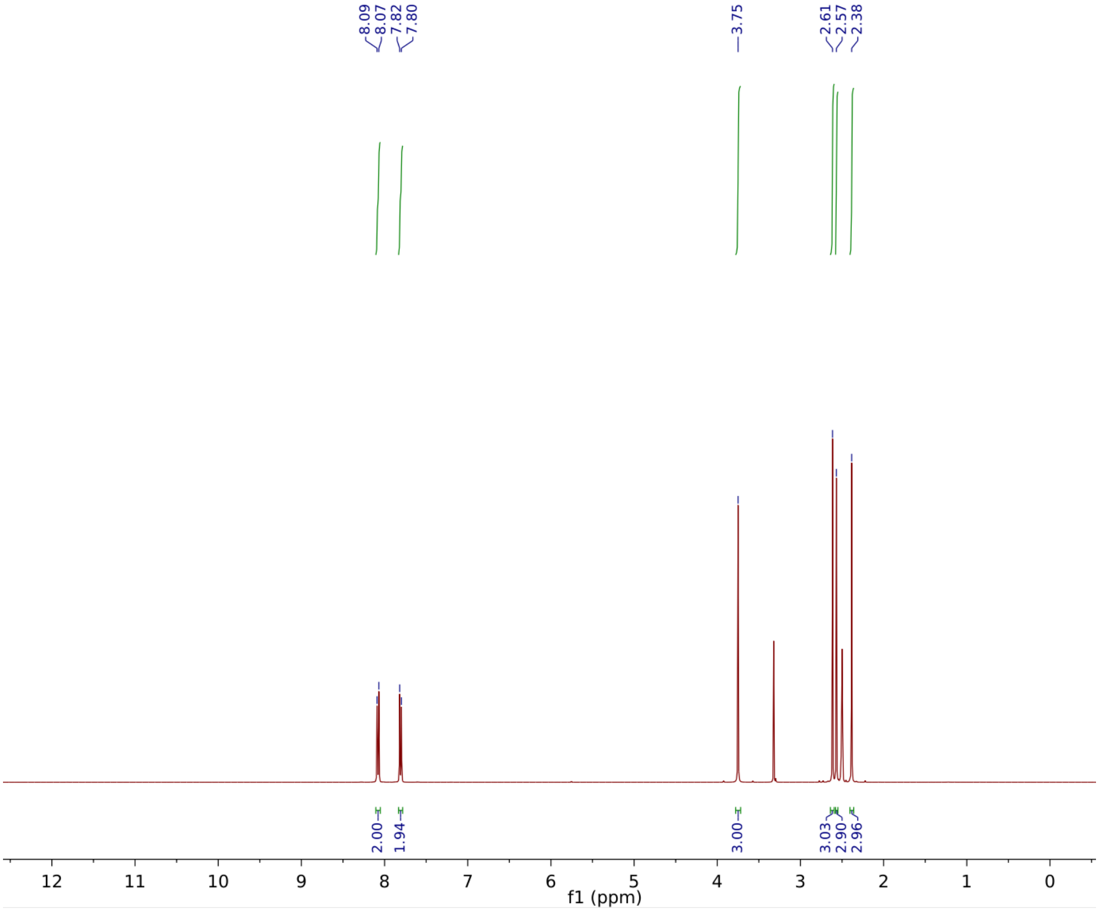

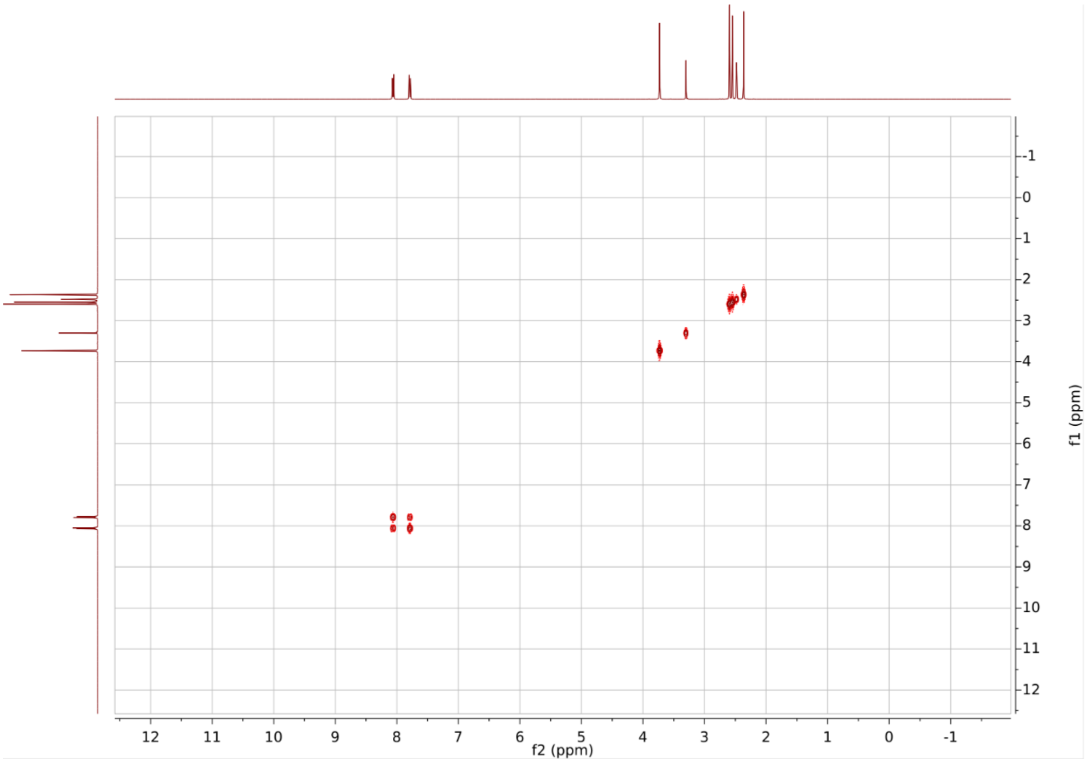

Compound S11b (^{13}C NMR, 101 MHz, DMSO-d_6)

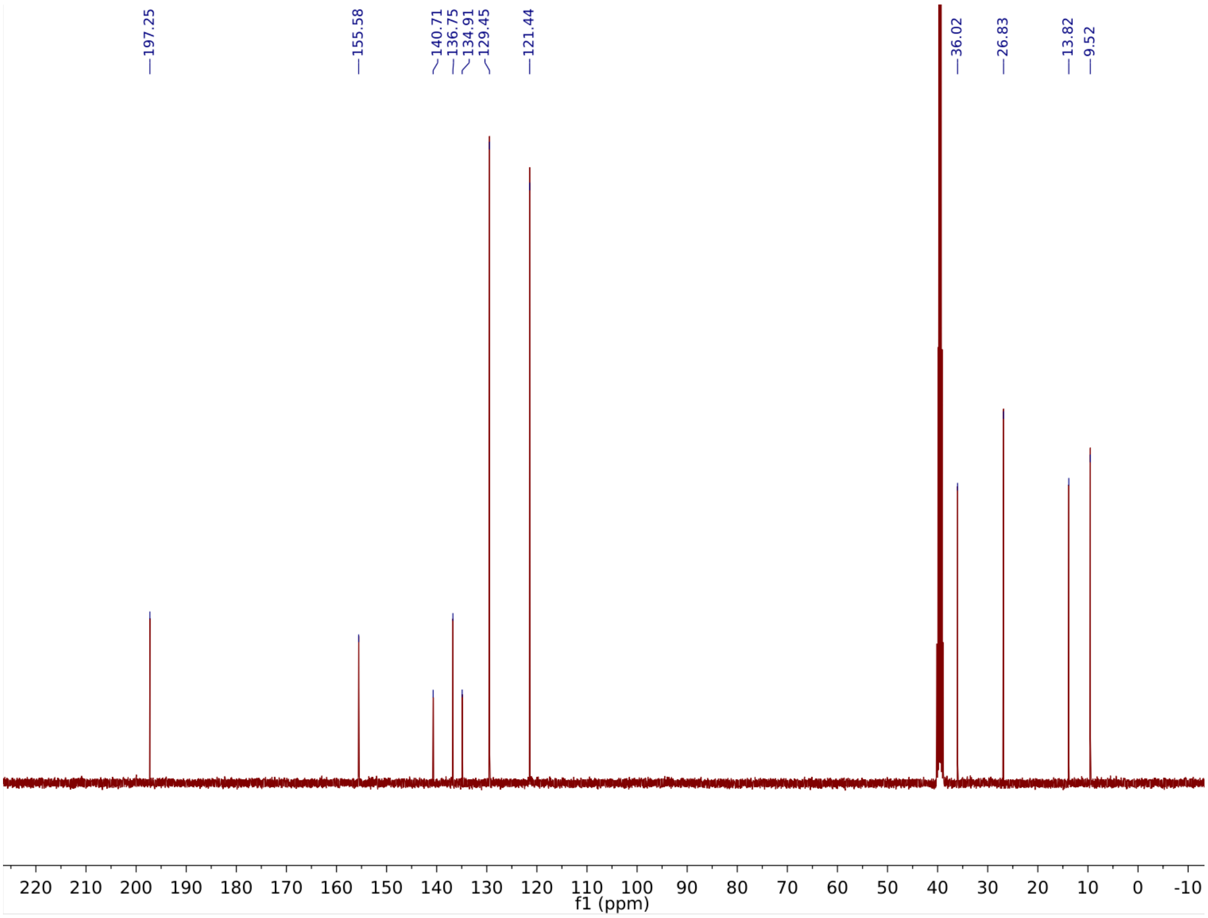

Compound S12a (^{1}H NMR, 400 MHz, $CDCl_3$)

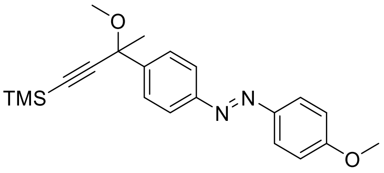

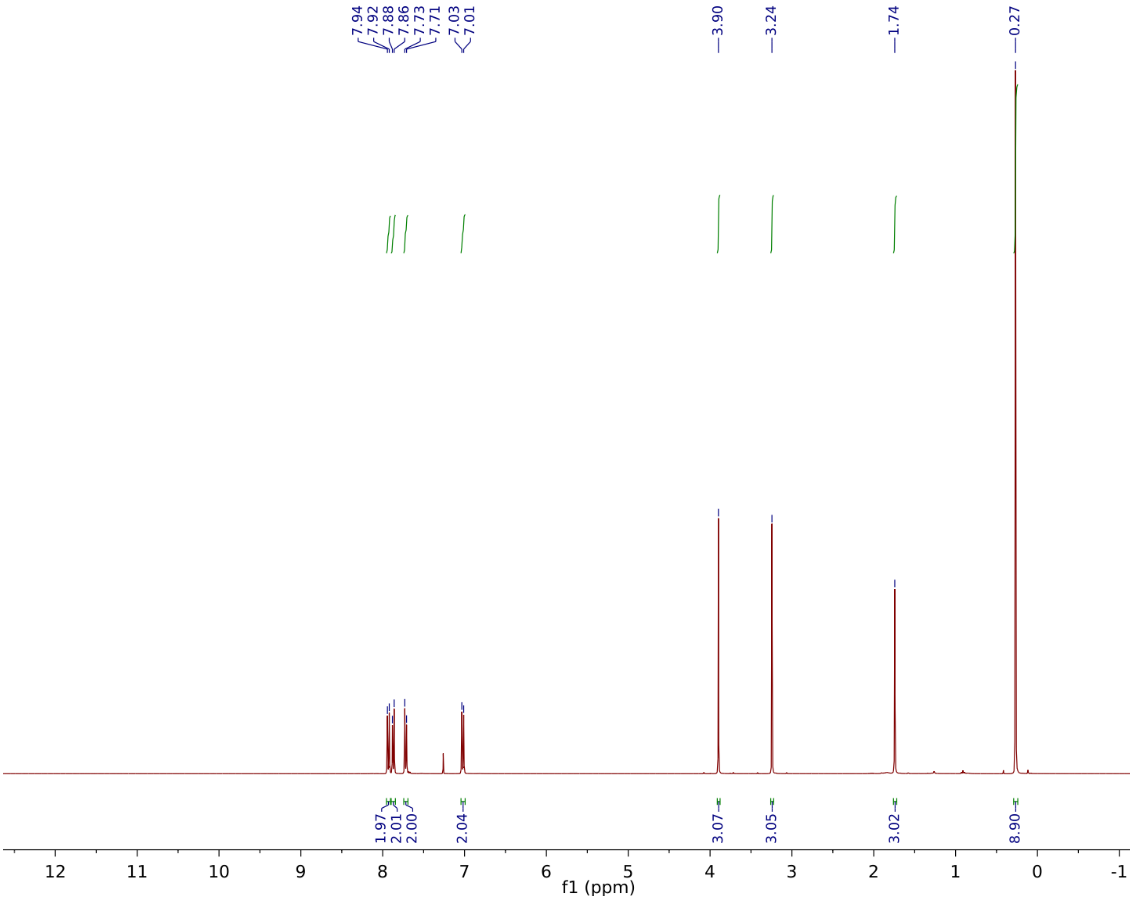

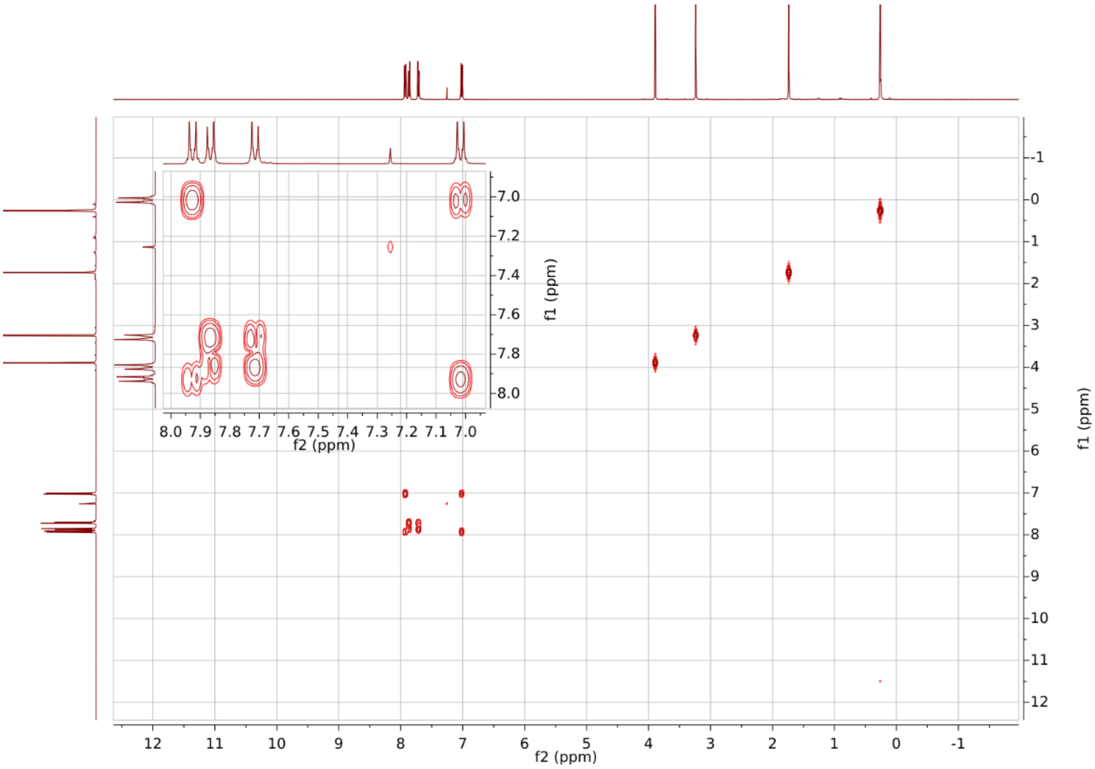

Compound S12a (^{13}C NMR, 101 MHz, $CDCl_3$)

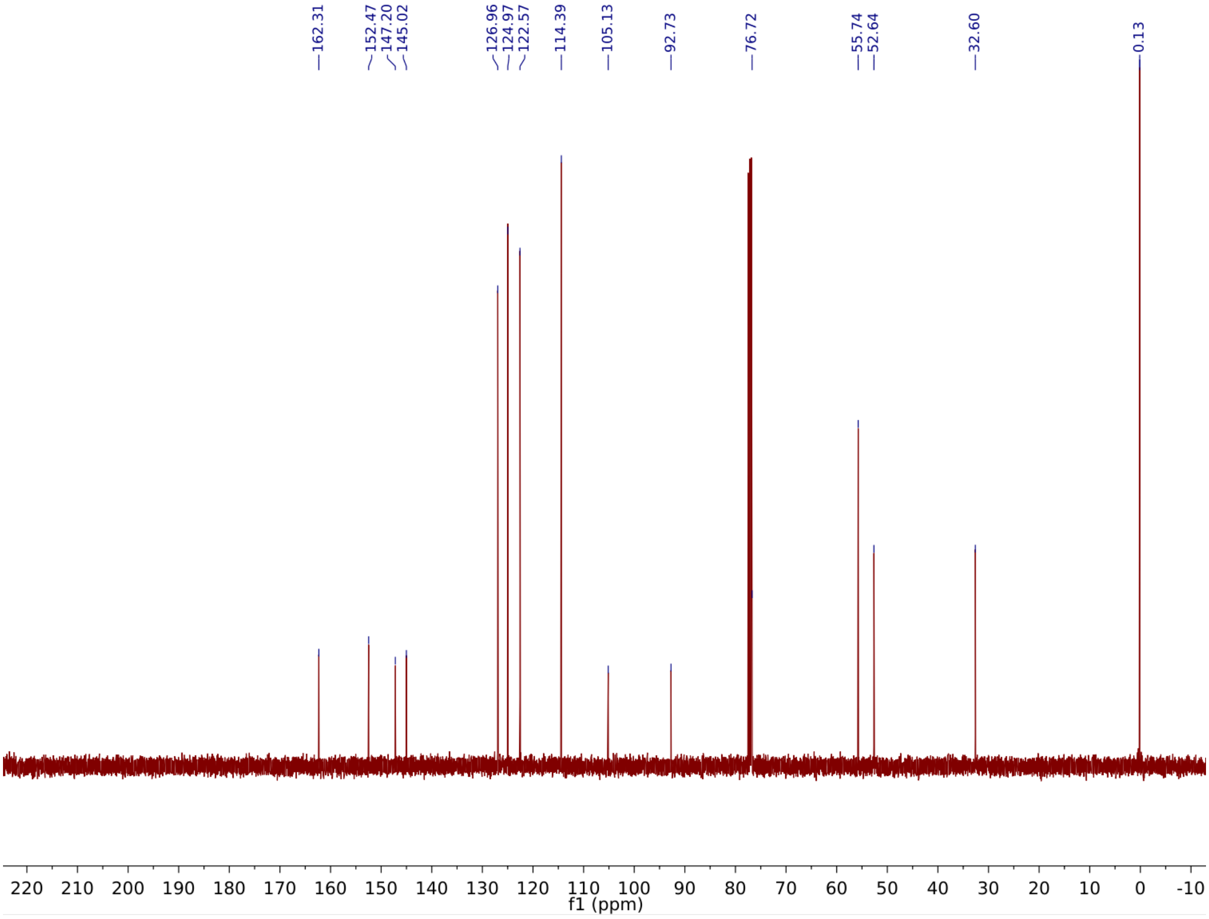

Compound S12b (^{1}H NMR, 400 MHz, $CDCl_3$)

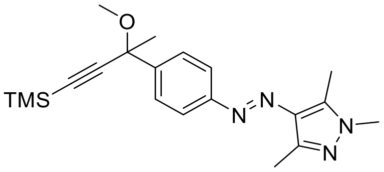

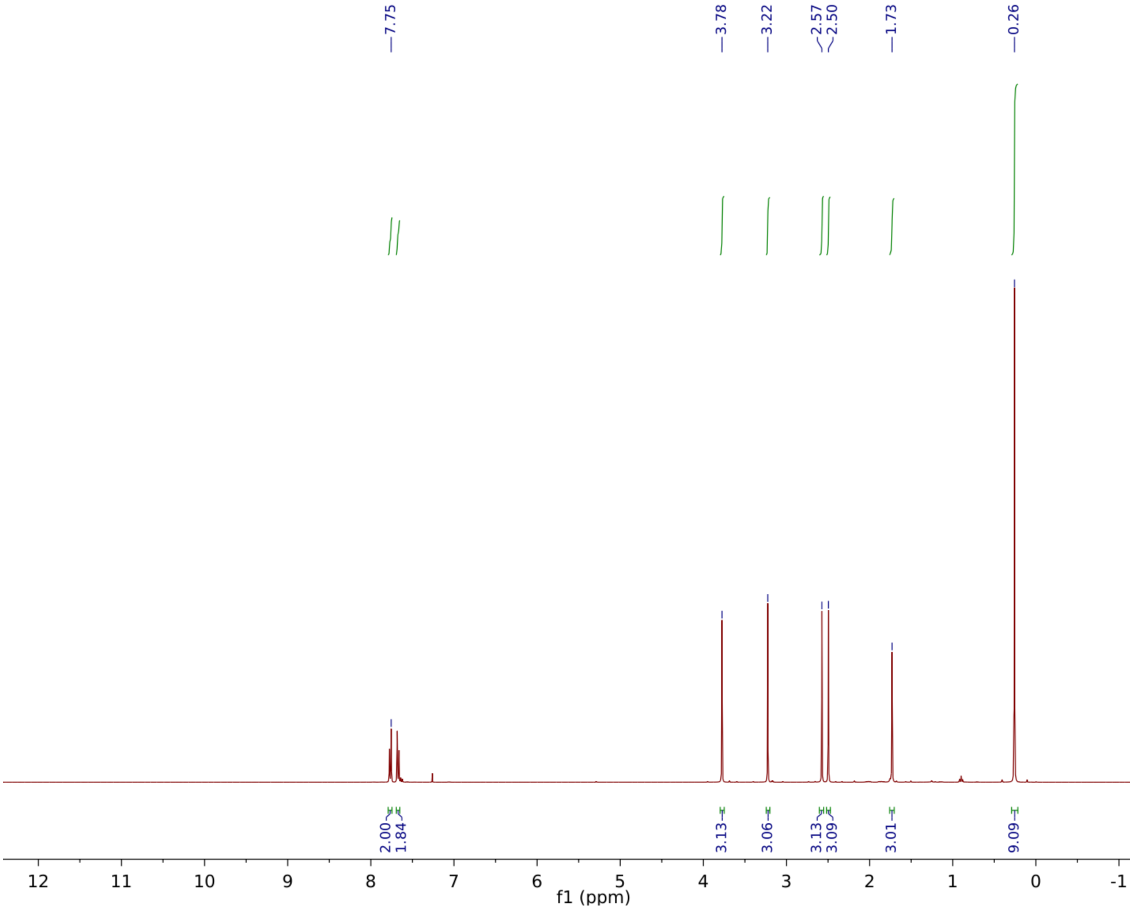

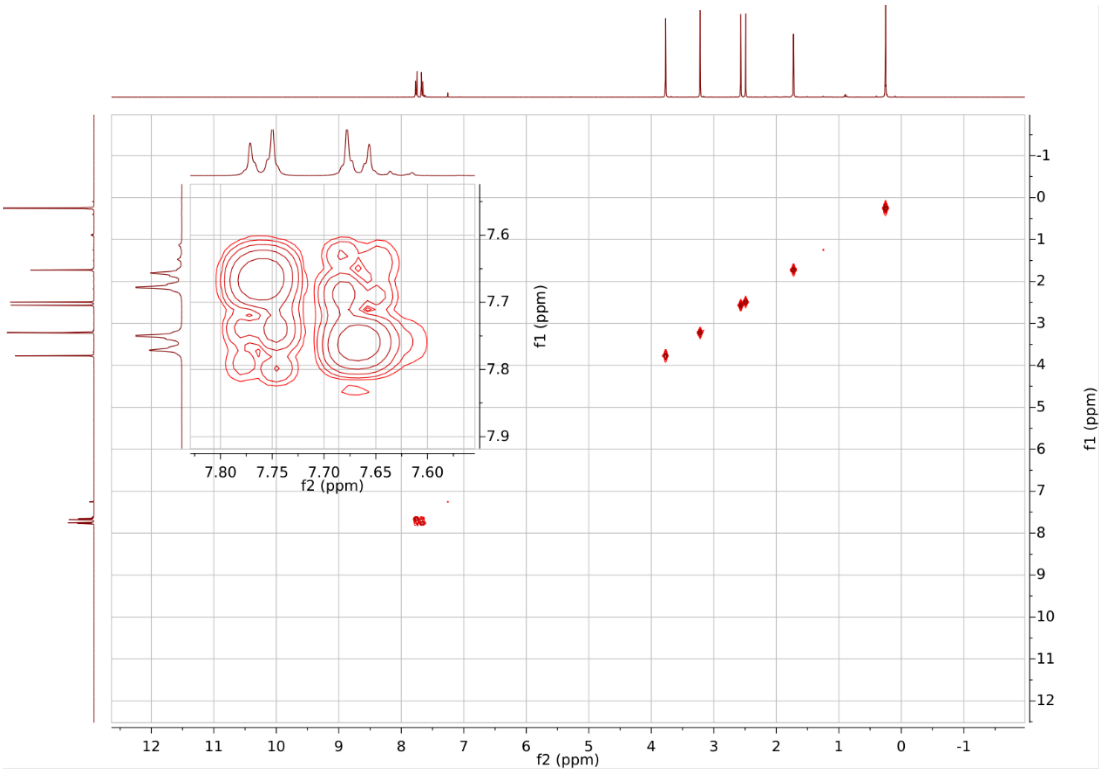

Compound S12b (^{13}C NMR, 101 MHz, $CDCl_3$)

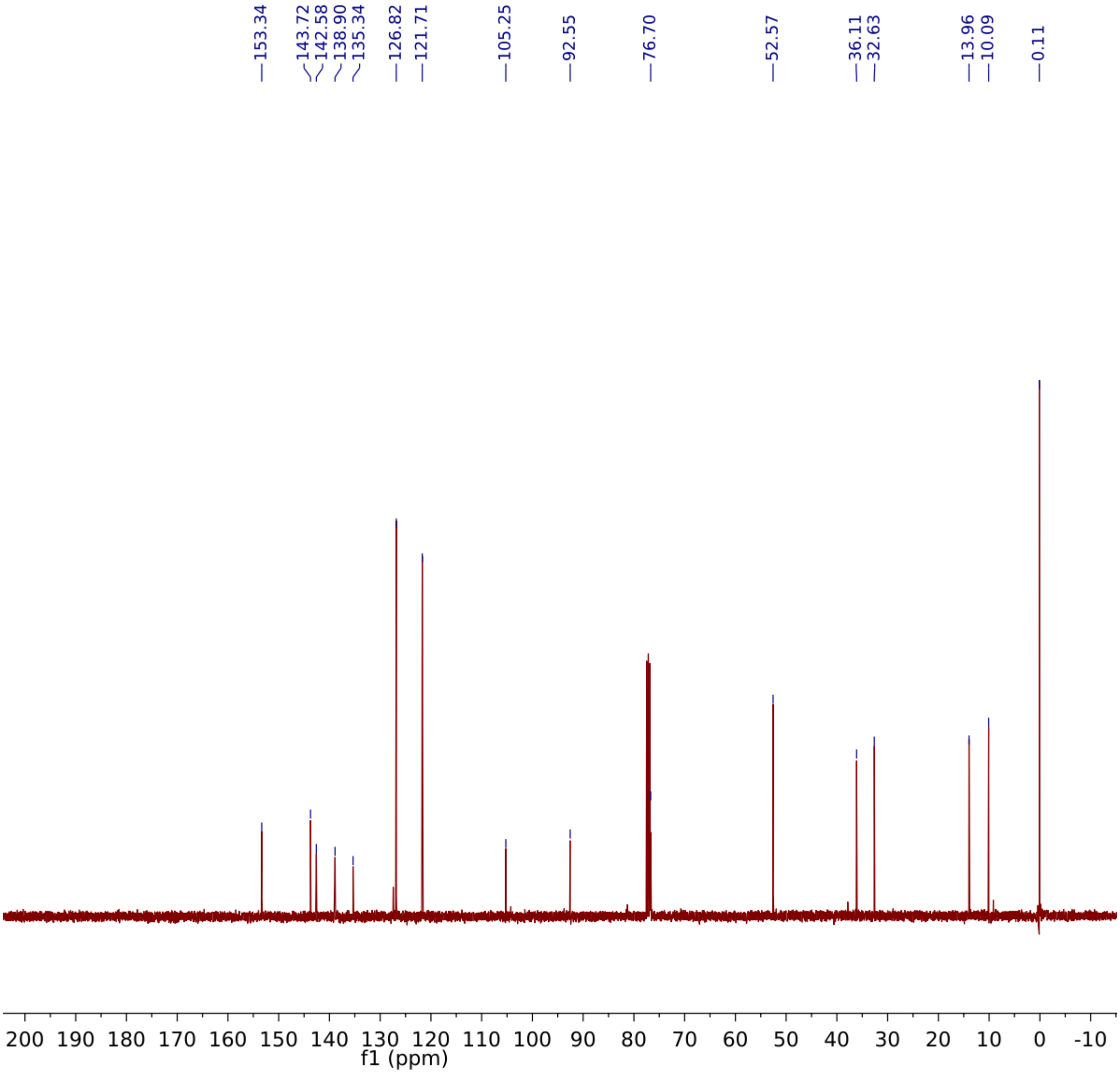

Compound S13a (^{1}H NMR, 400 MHz, $CDCl_3$)

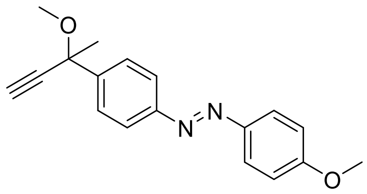

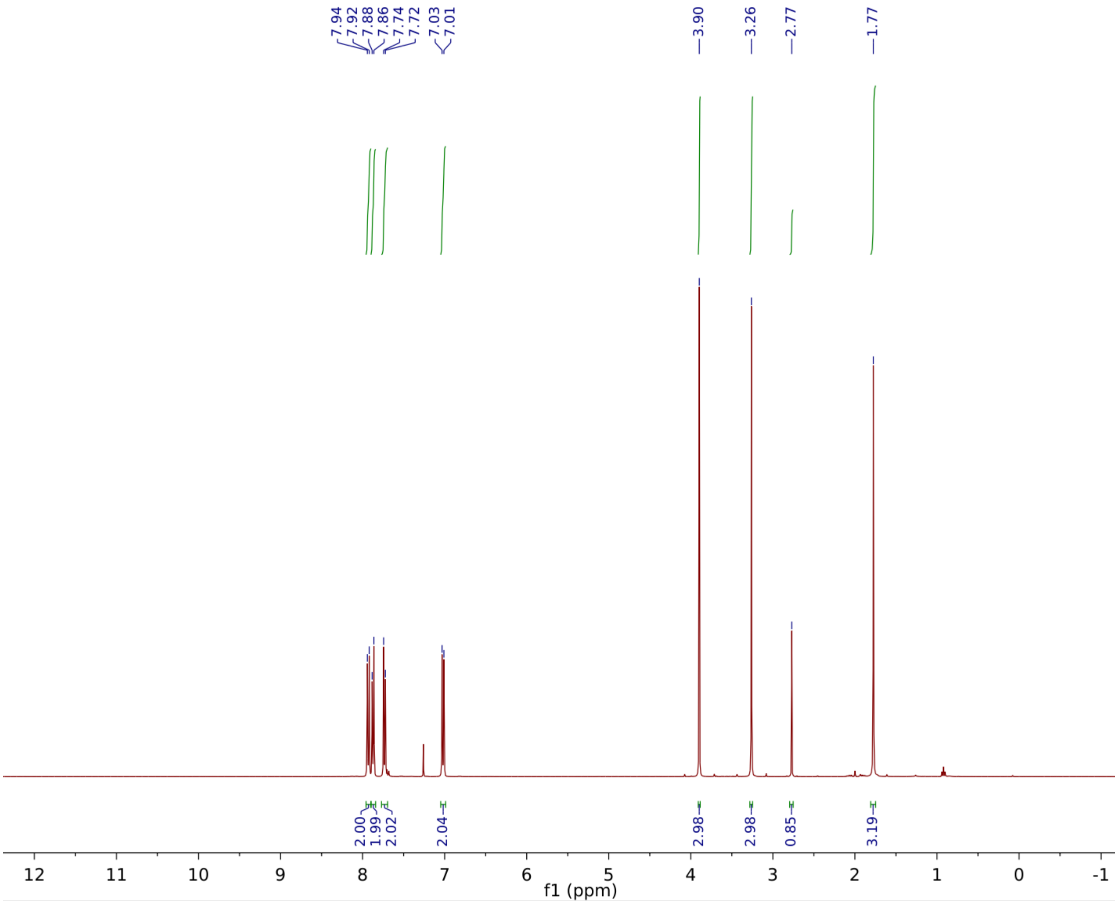

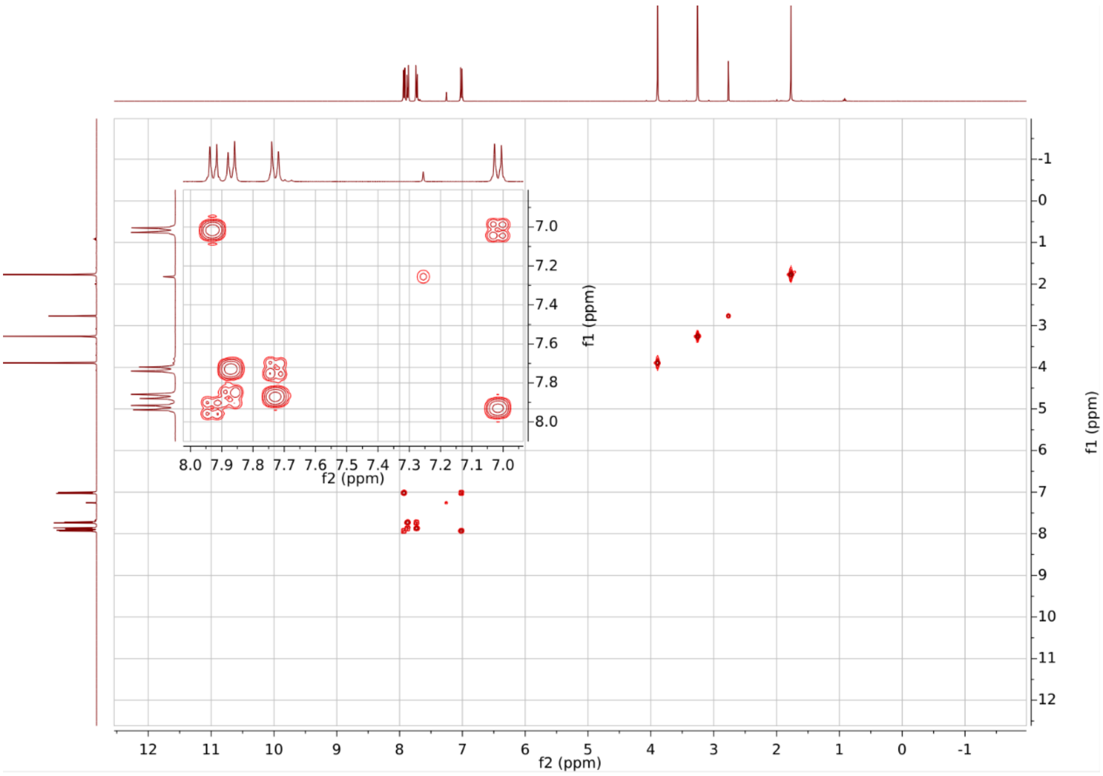

Compound S13a (^{13}C NMR, 101 MHz, $CDCl_3$)

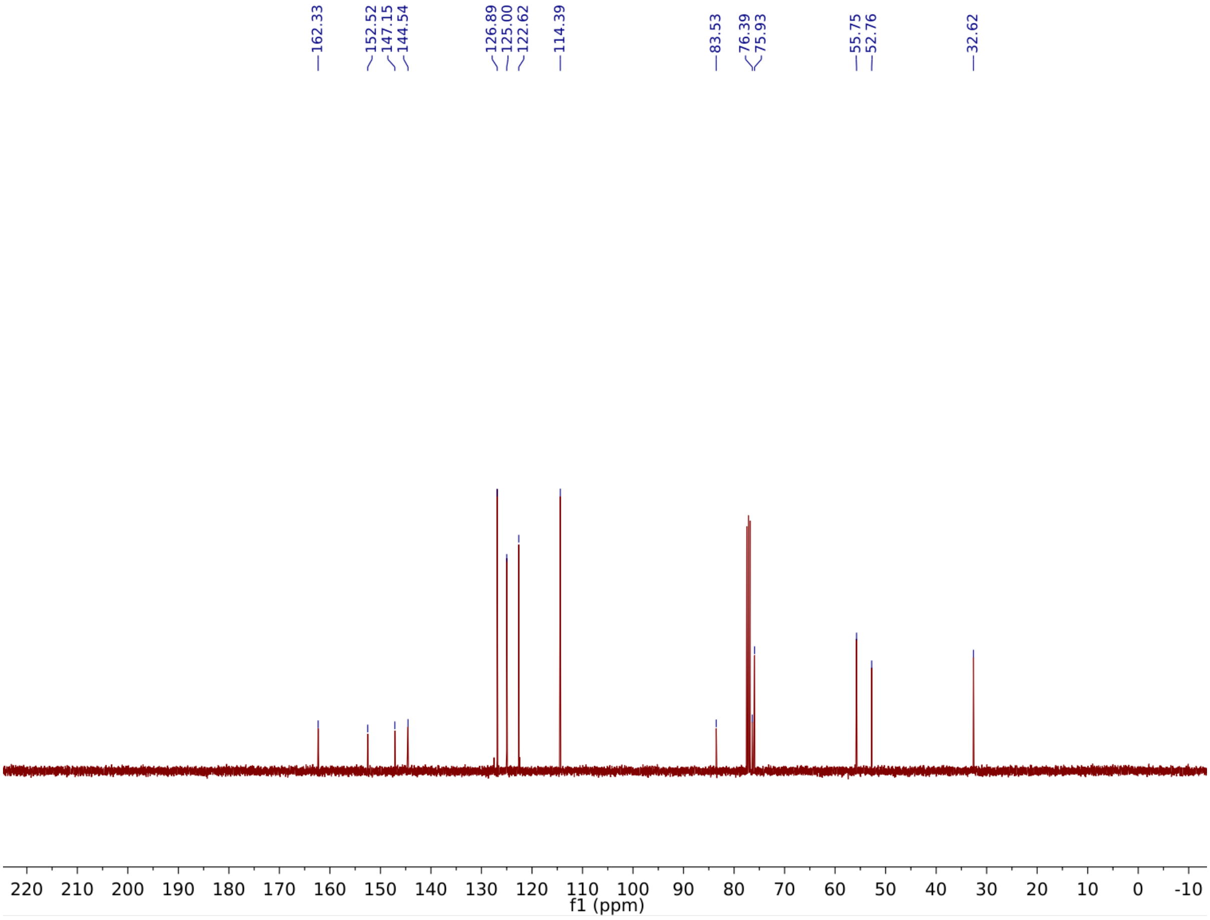

Compound S13b (^{1}H NMR, 400 MHz, $CDCl_3$)

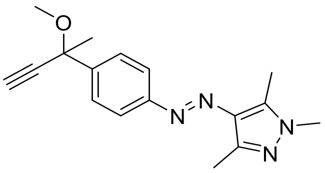

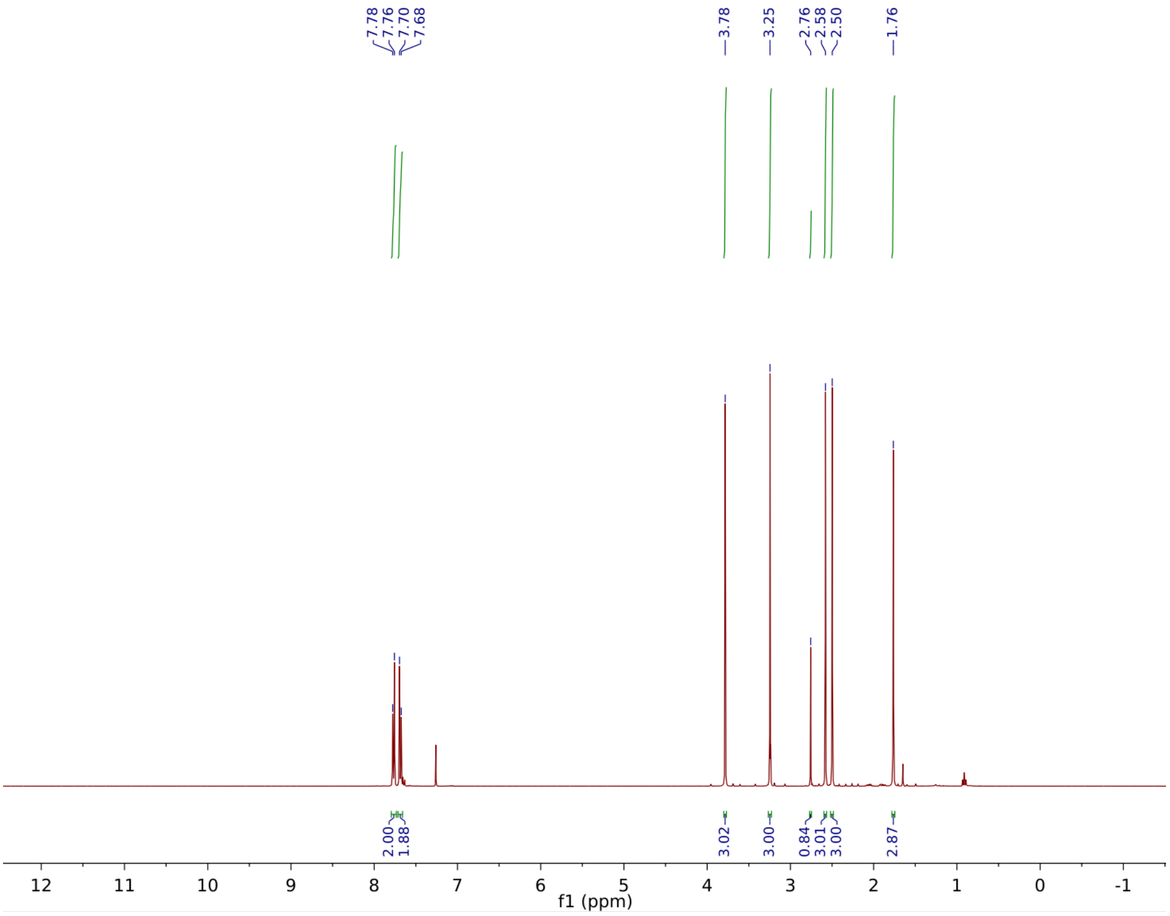

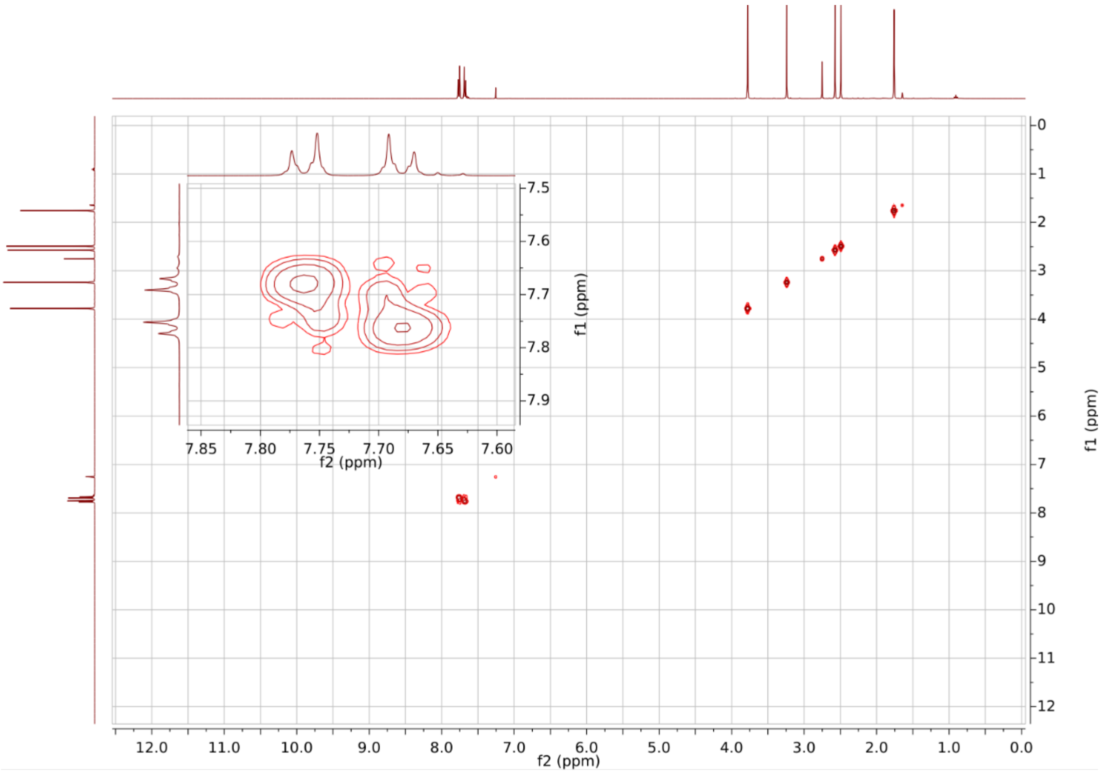

Compound S13b (^{13}C NMR, 101 MHz, $CDCl_3$)

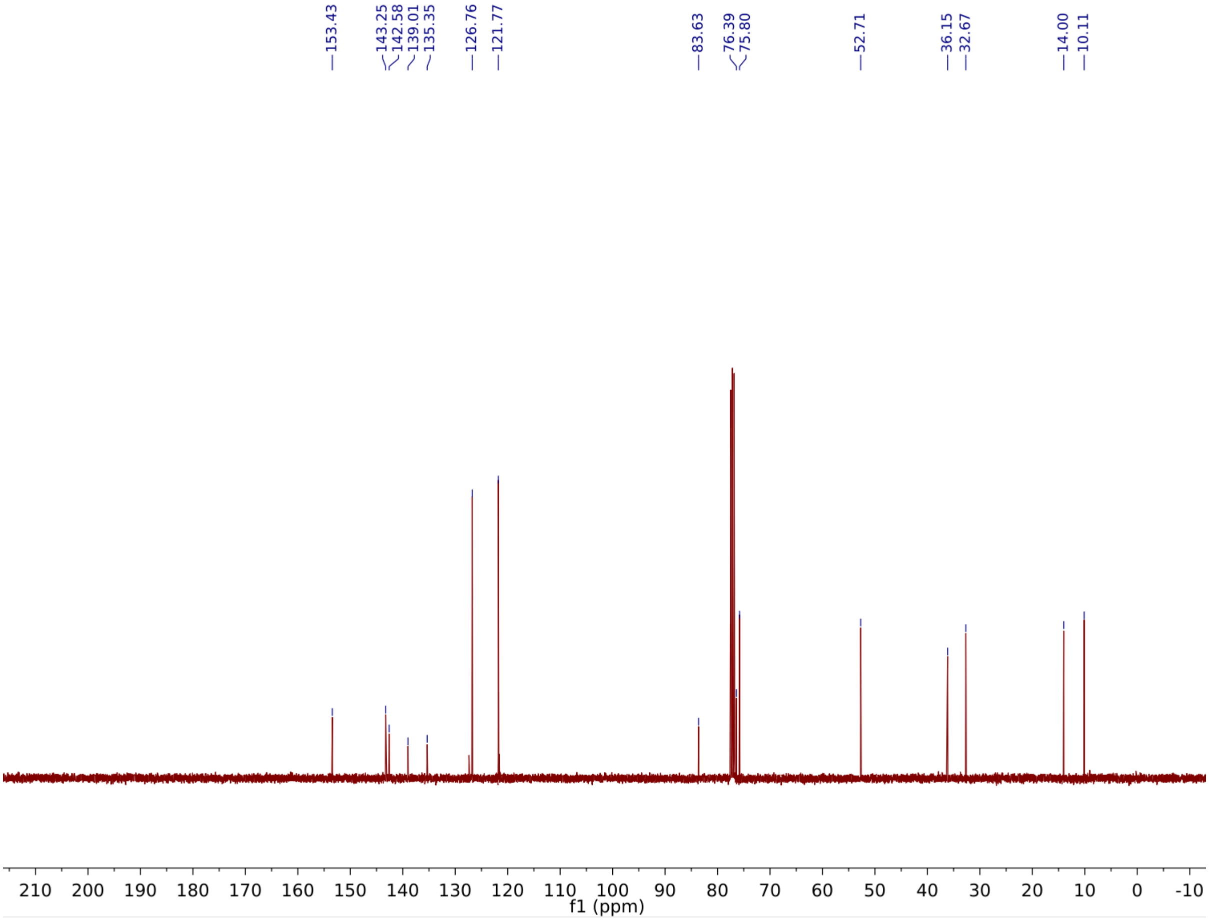

Compound 8 (^{1}H NMR, 400 MHz, DMSO-d_6)

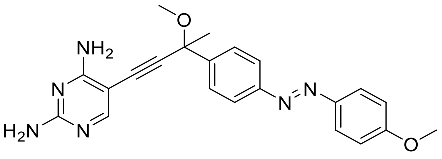

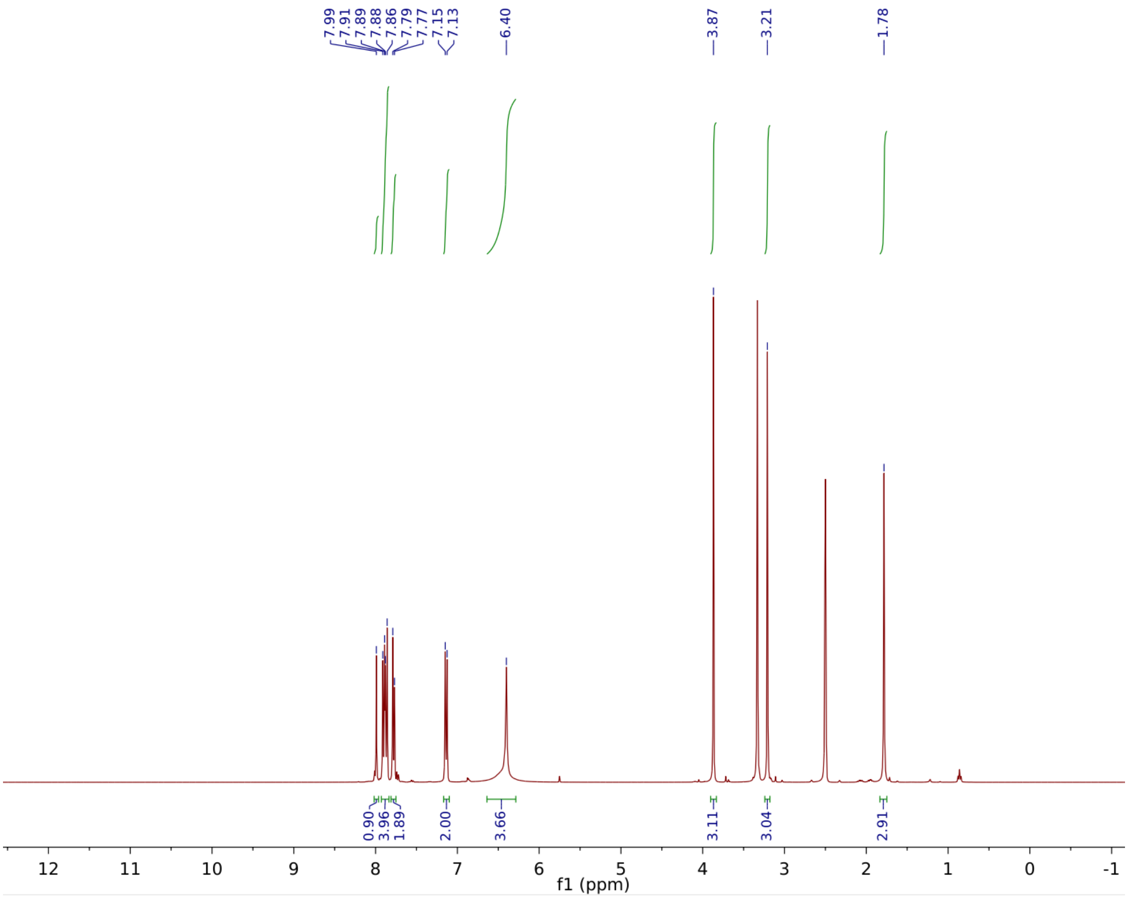

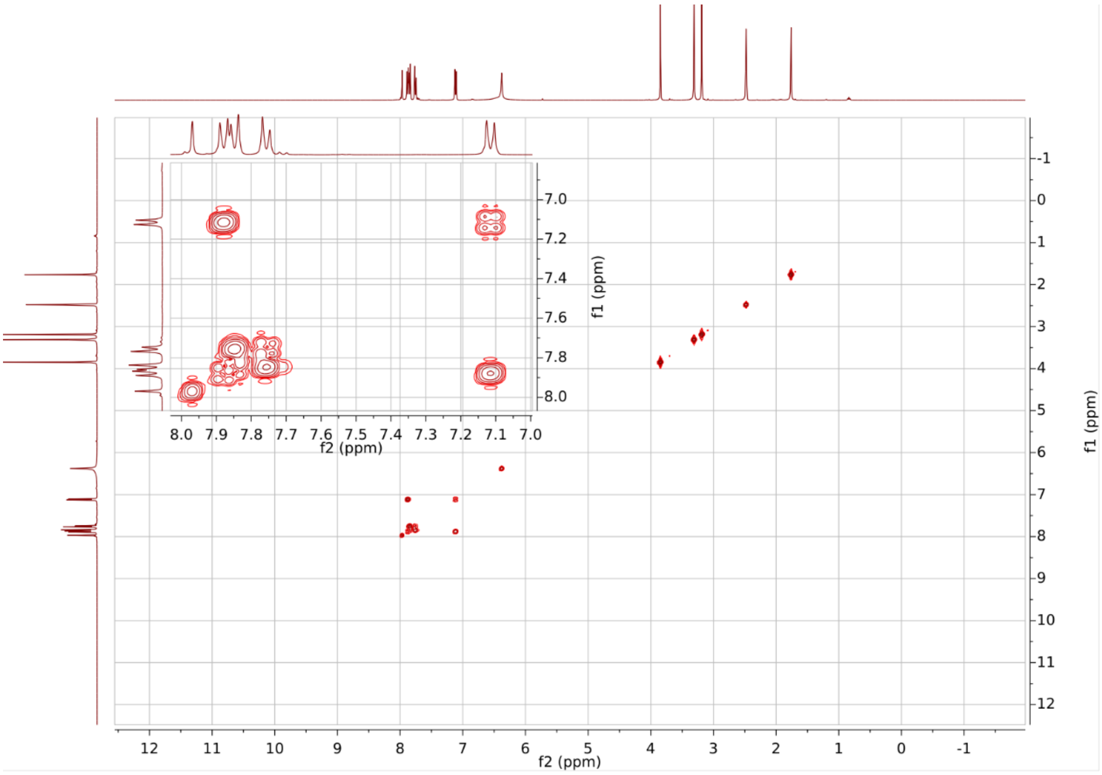

Compound 8 (^{13}C NMR, 101 MHz, DMSO-d_6)

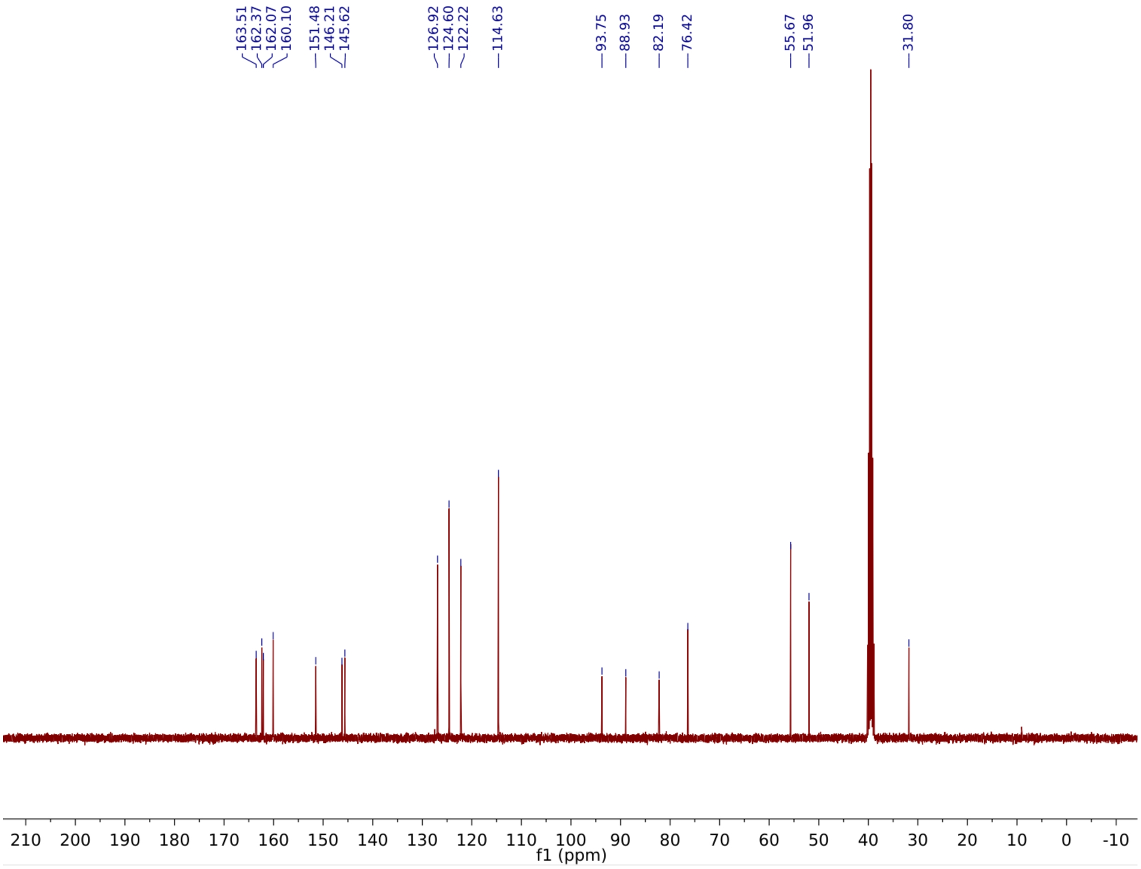

Compound 8 (HRMS)

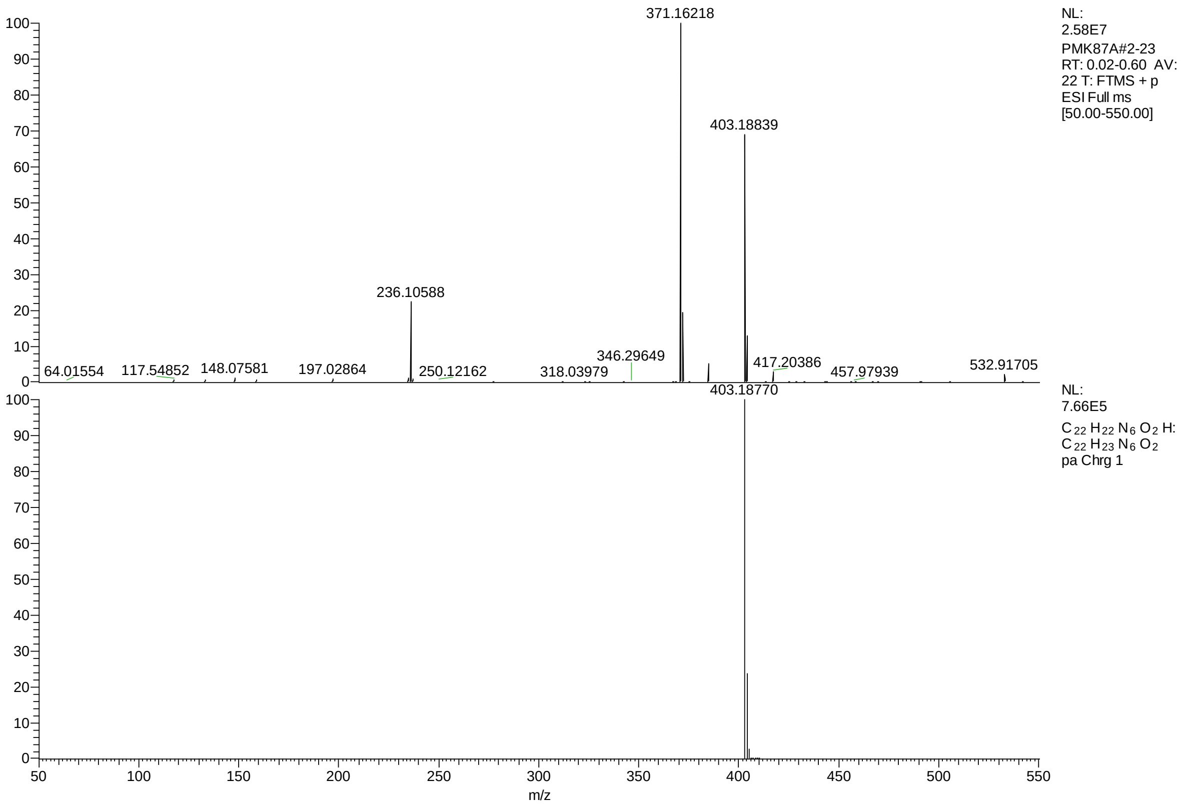

Compound 9 (^{1}H NMR, 400 MHz, $CDCl_3$)

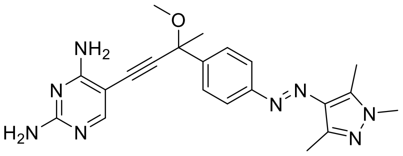

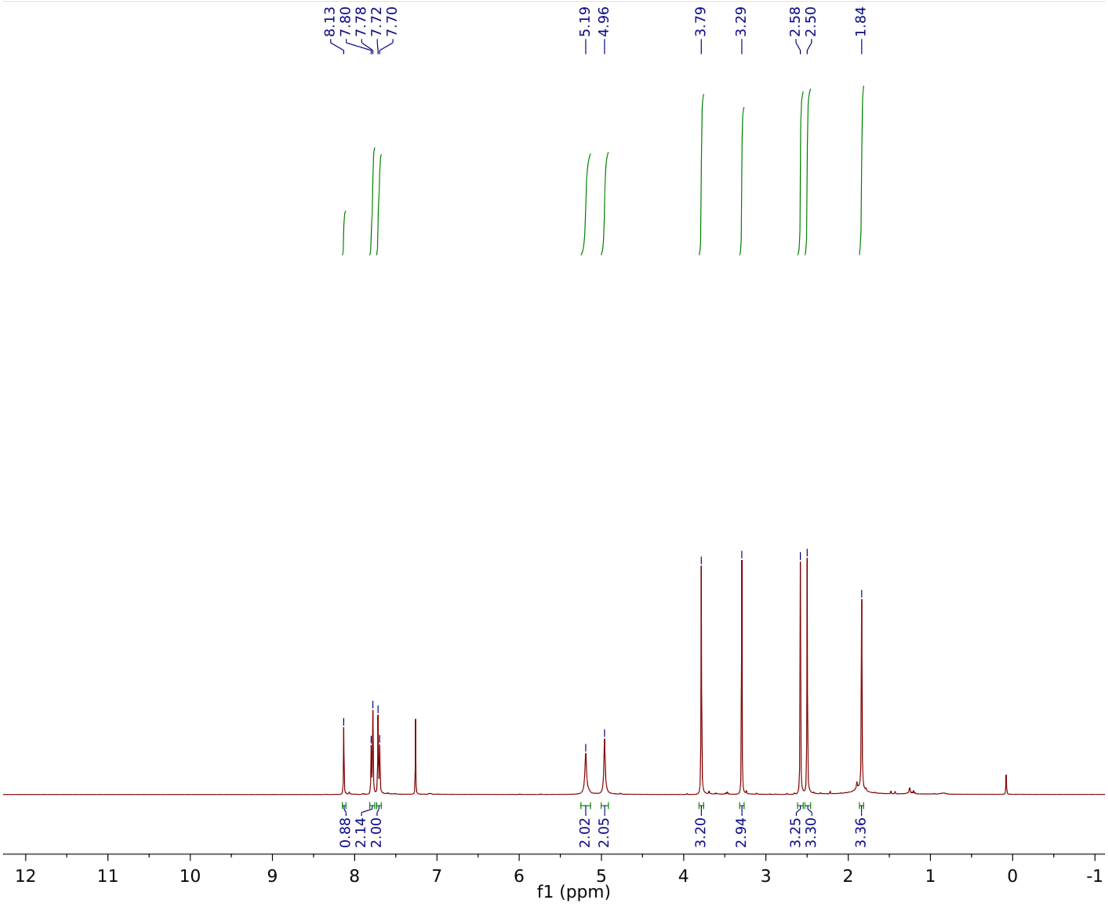

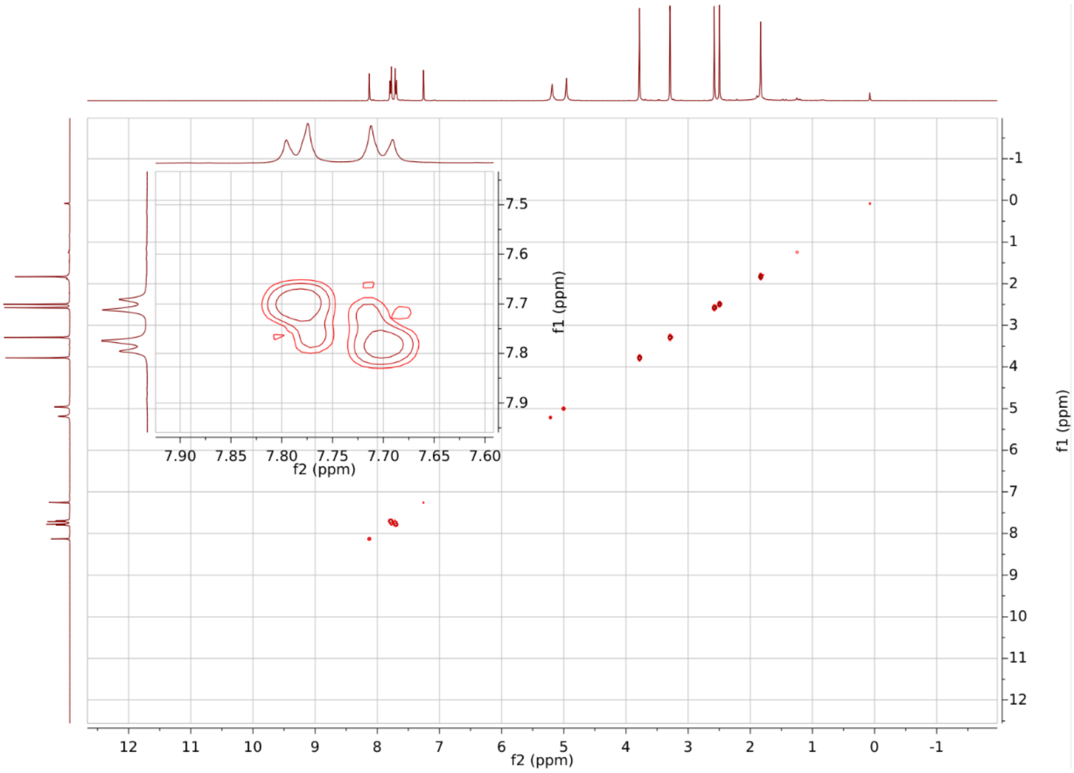

Compound 9 (^{13}C NMR, 101 MHz, $CDCl_3$)

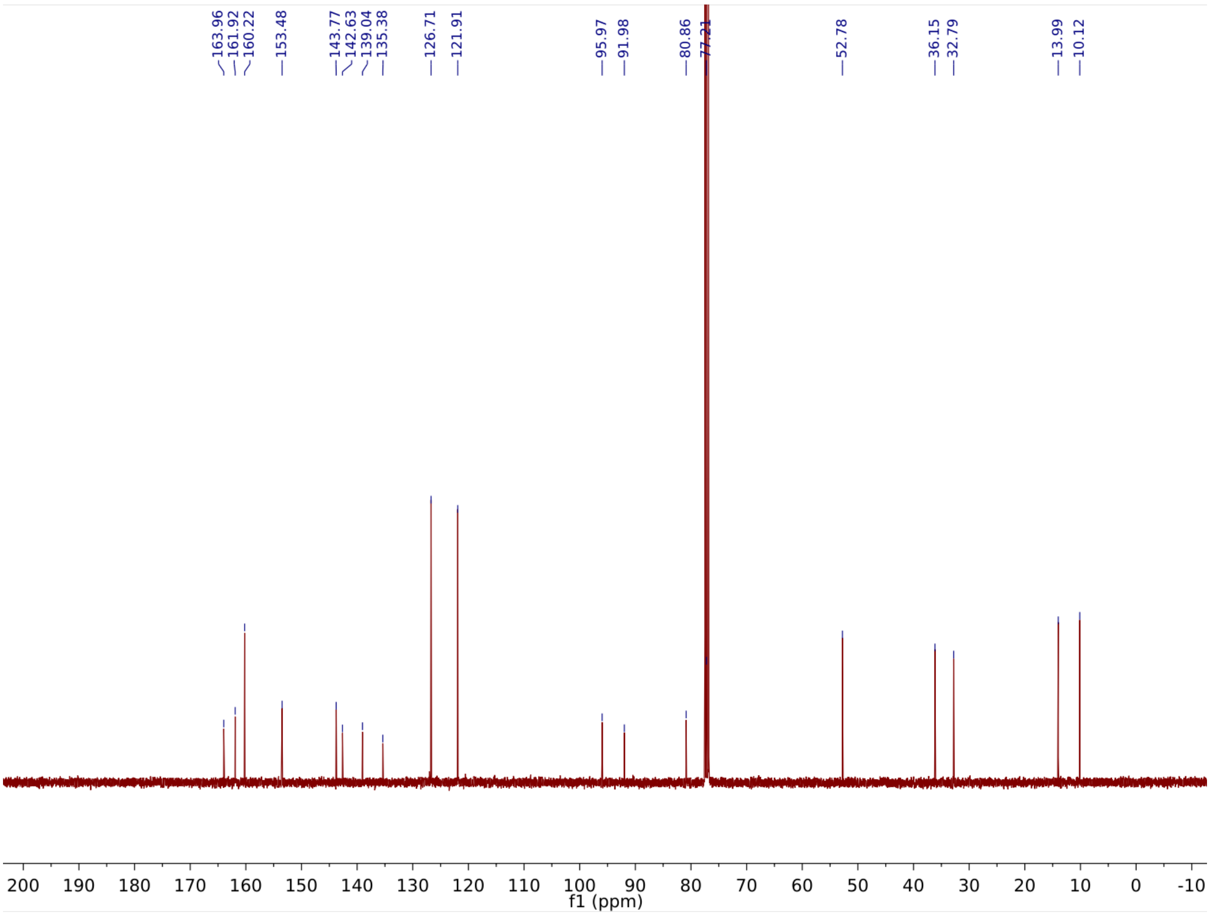

Compound 9 (HRMS)

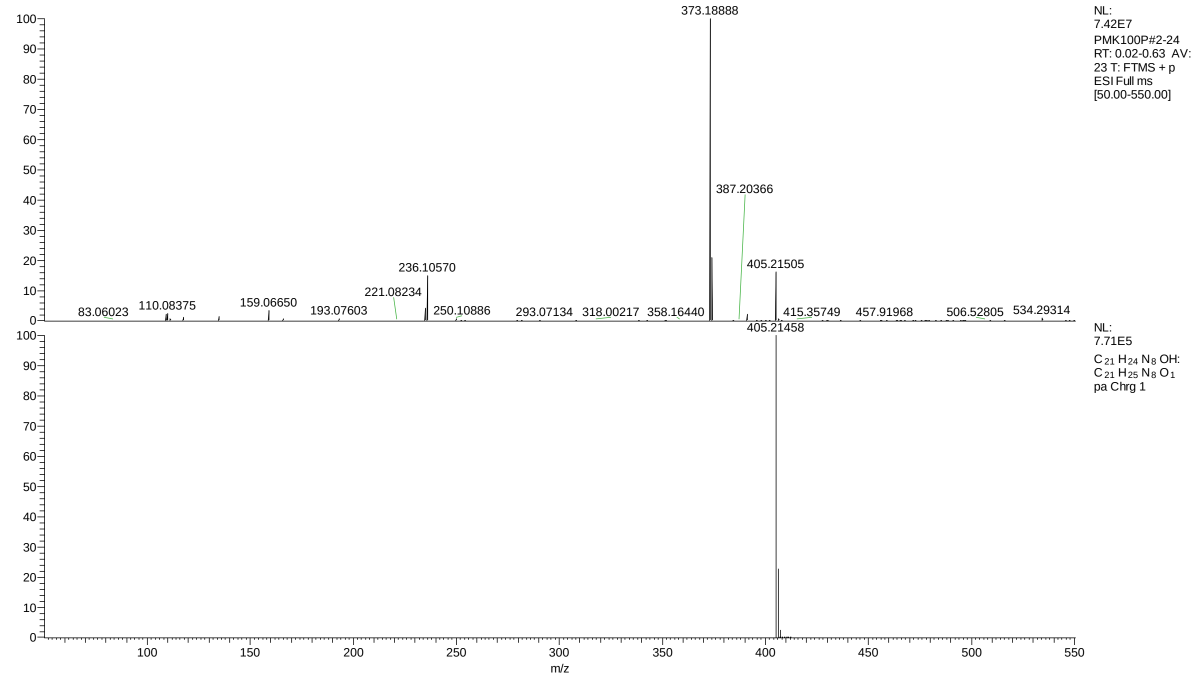

S7.4. Fourth hypothesis

Compound S14a (^{1}H NMR, 400 MHz, $CDCl_3$)

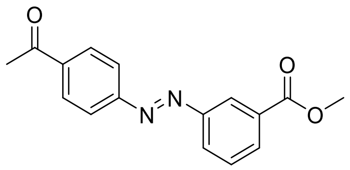

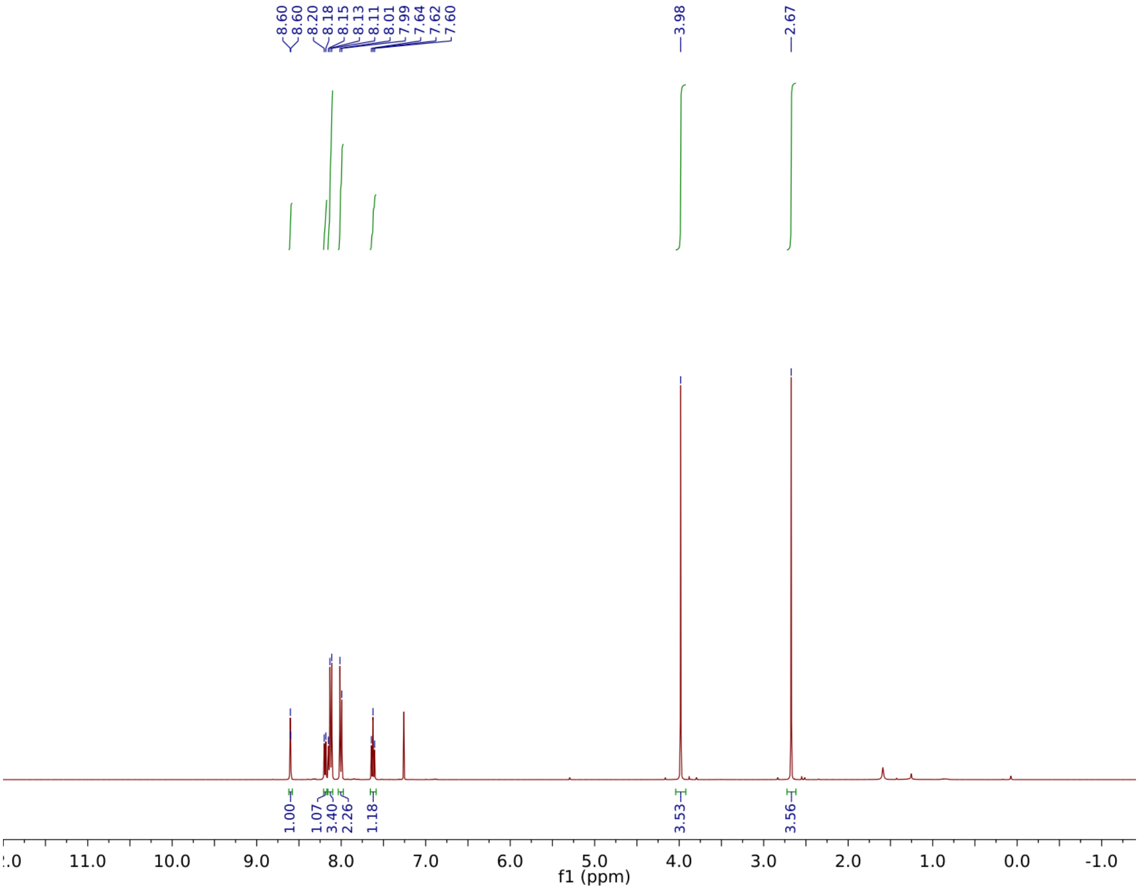

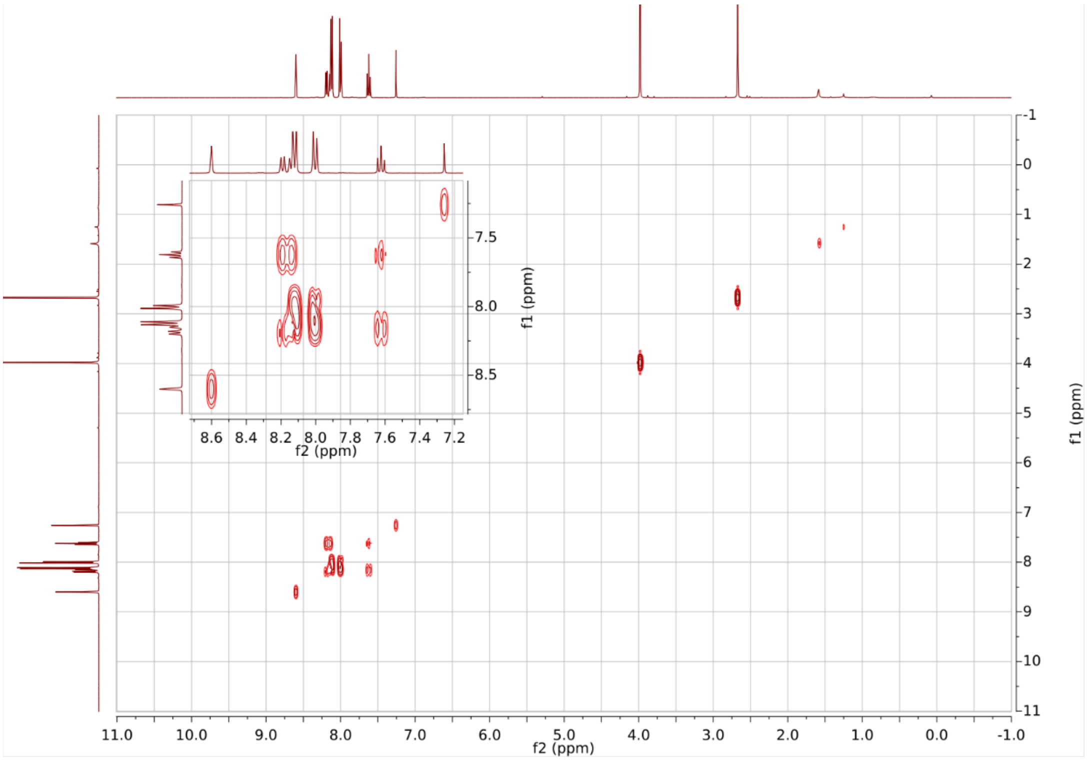

Compound S14a (^{13}C NMR, 101 MHz, $CDCl_3$)

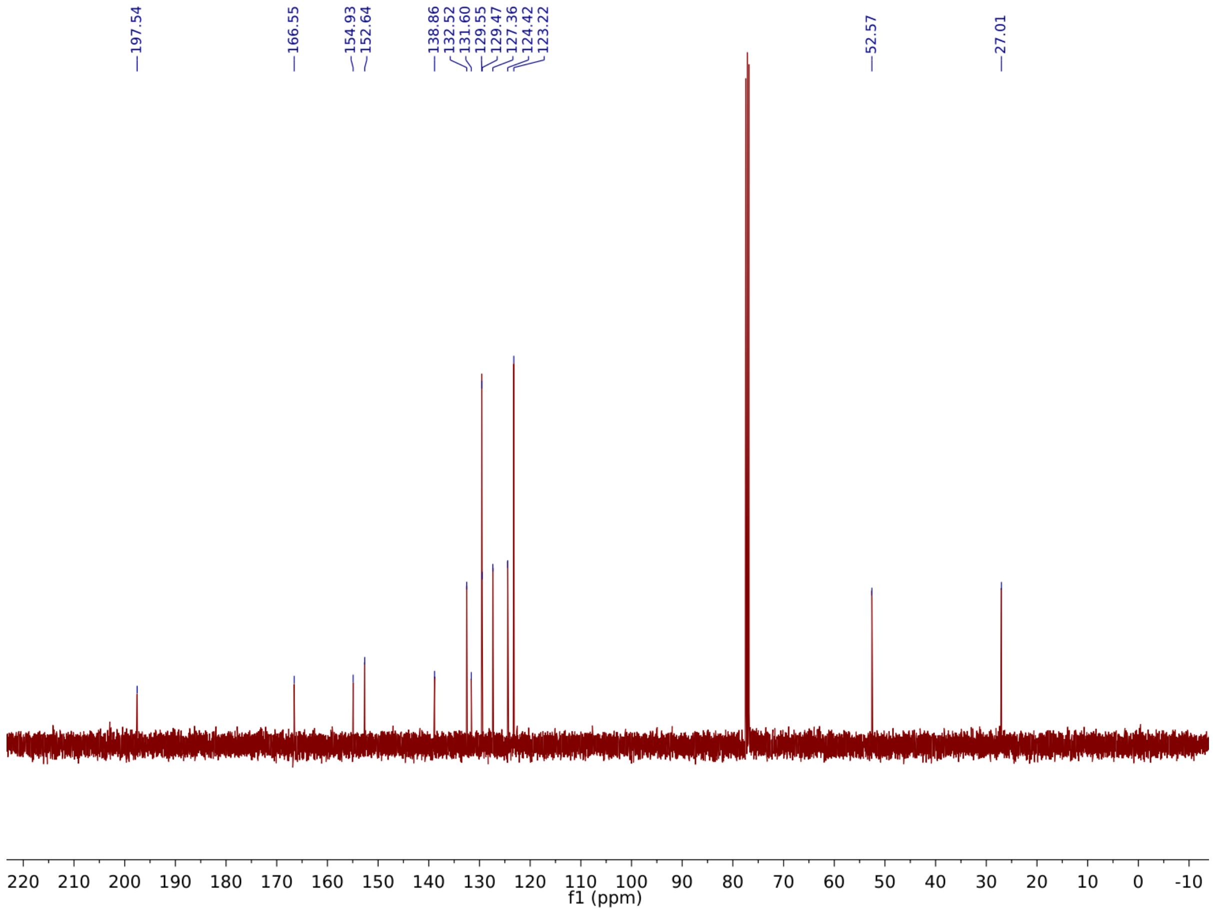

Compound S14b (¹H NMR, 400 MHz, $CDCl_3$)

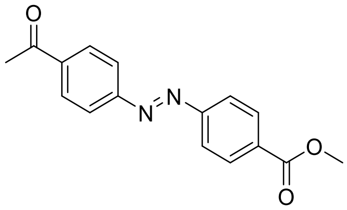

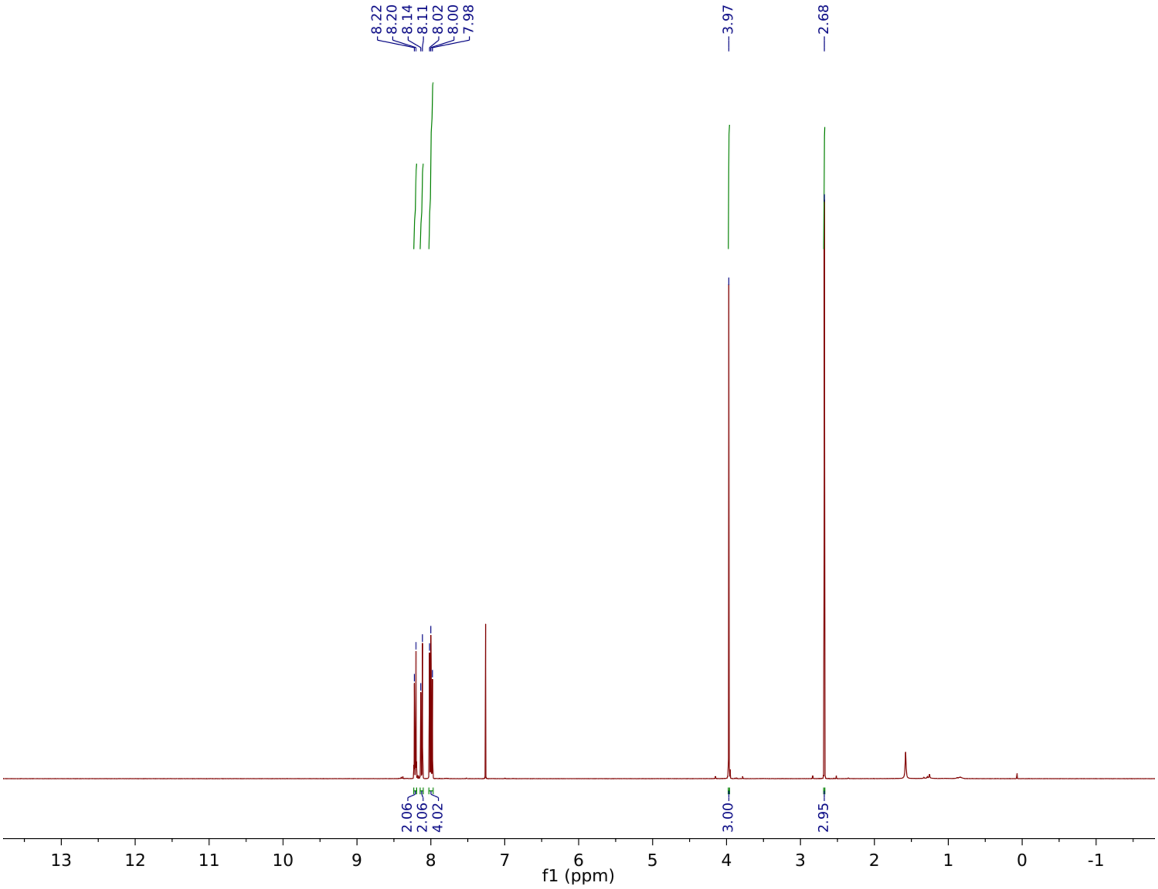

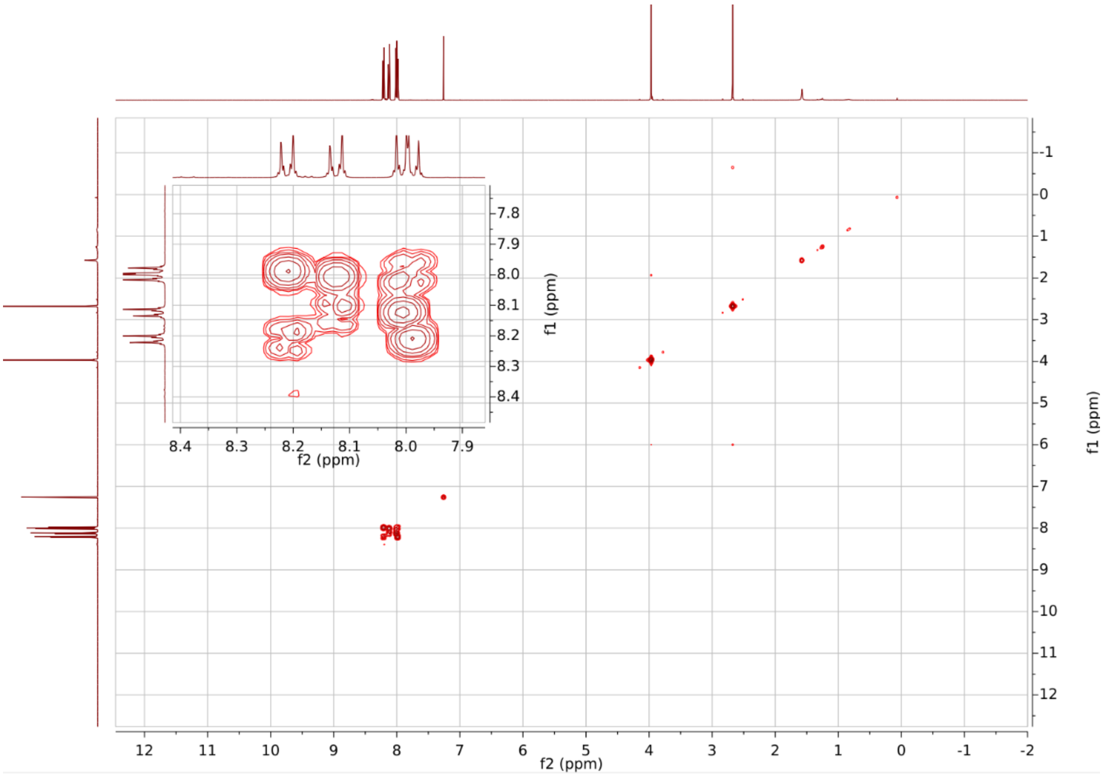

Compound S14b (^{13}C NMR, 101 MHz, $CDCl_3$)

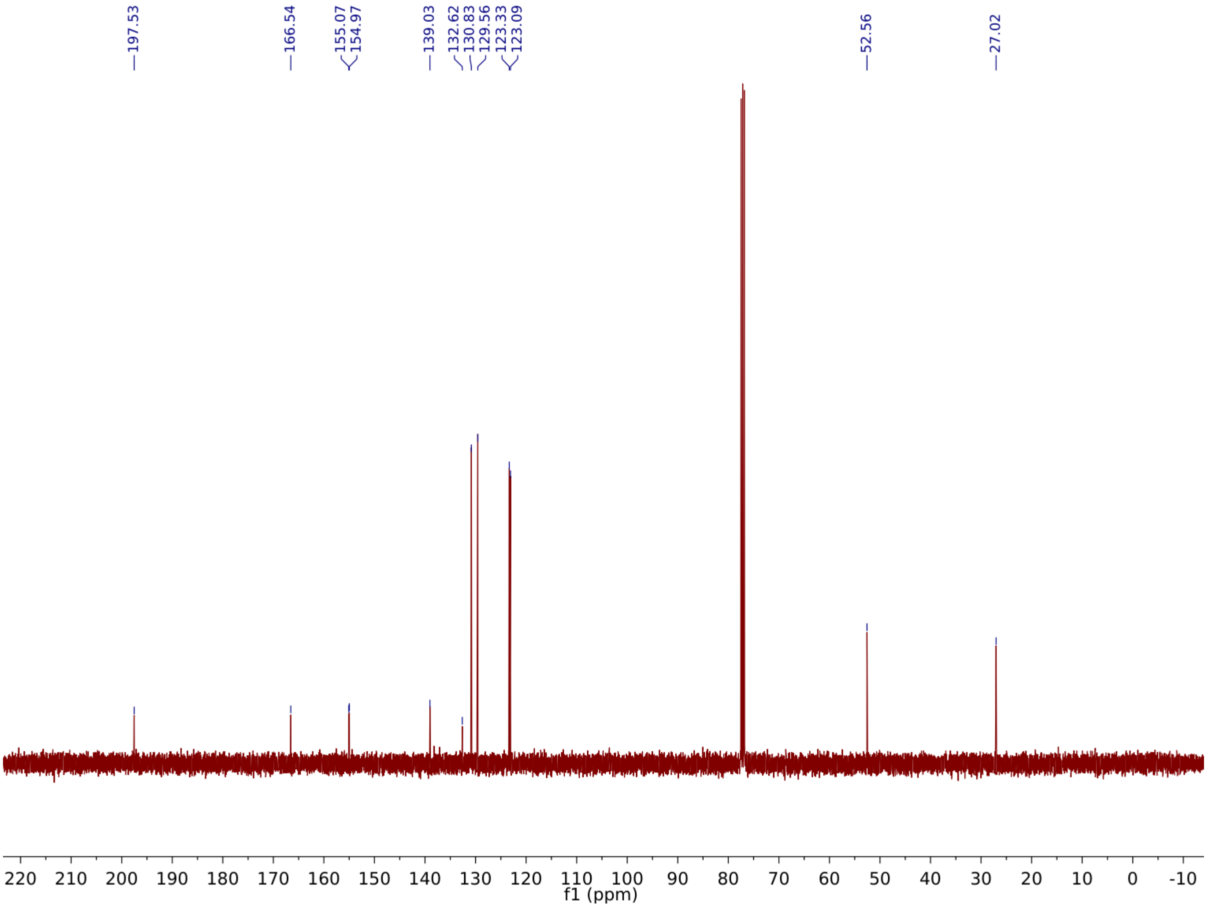

Compound S15a (^{1}H NMR, 400 MHz, DMSO-d_6)

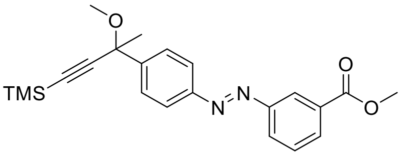

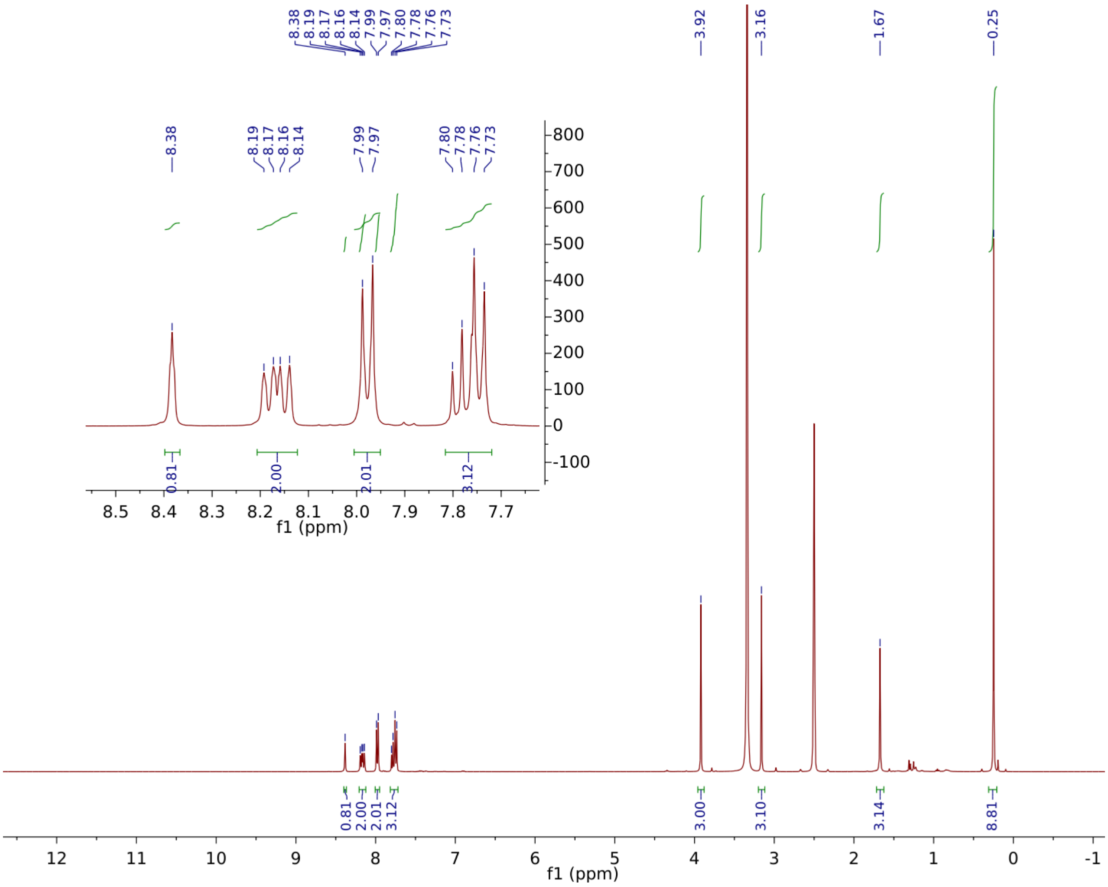

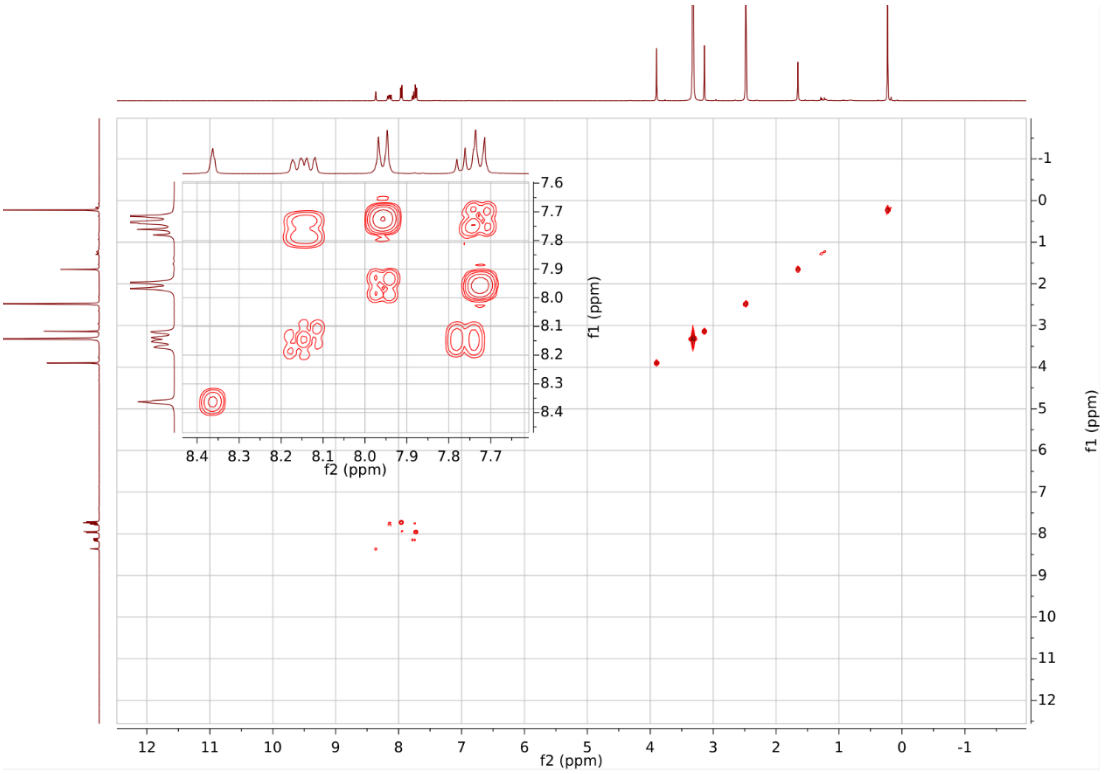

Compound S15a (^{13}C NMR, 101 MHz, DMSO-d_6)

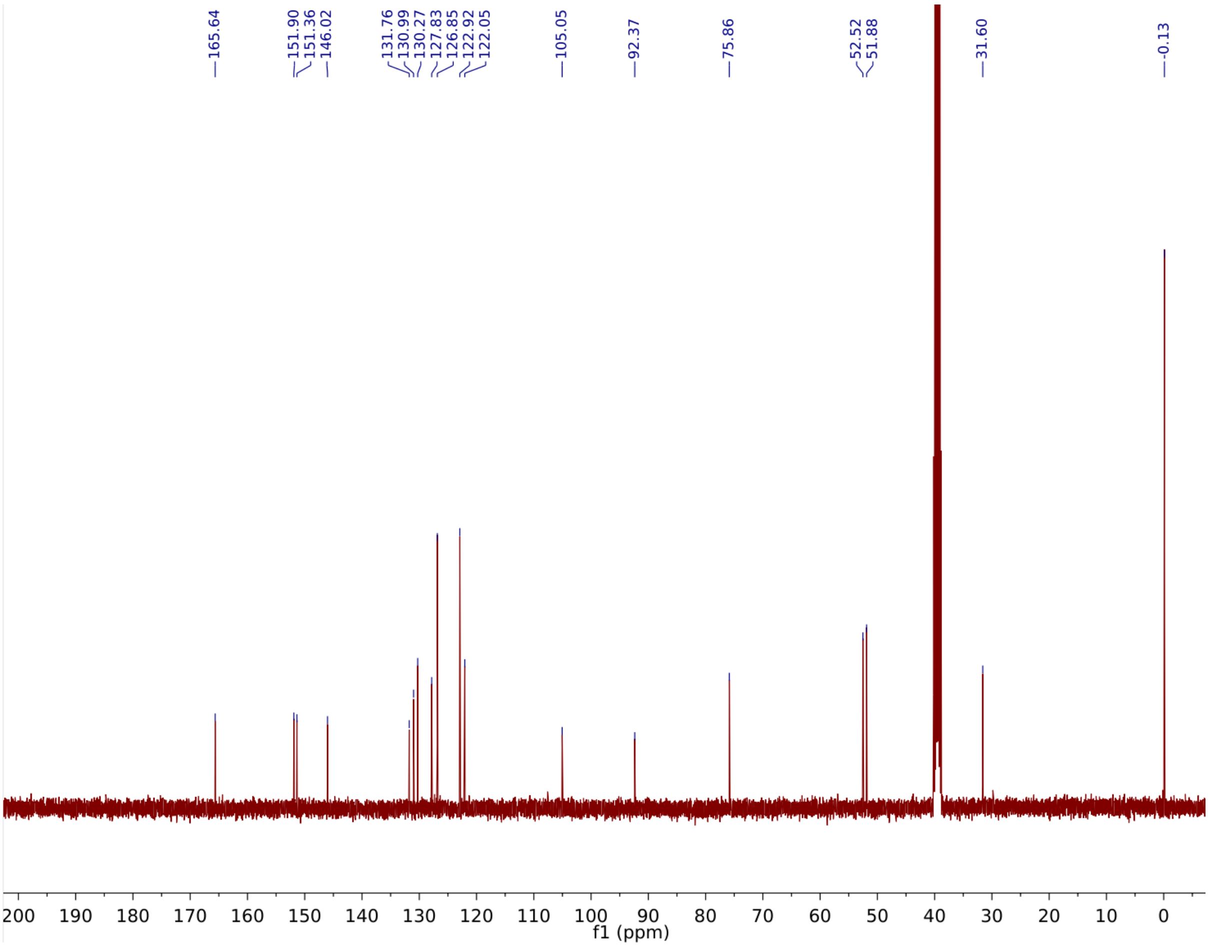

Compound S15b (^{1}H NMR, 400 MHz, $CDCl_3$)

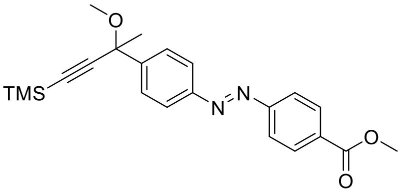

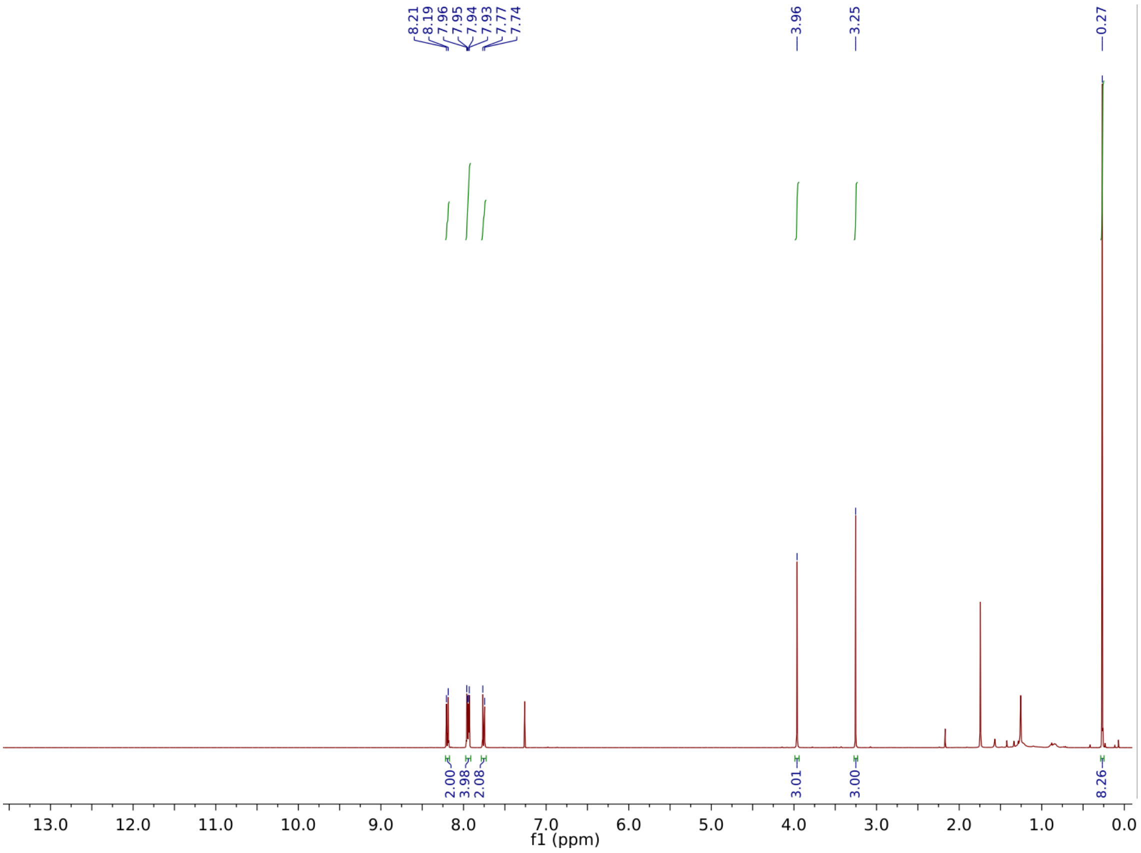

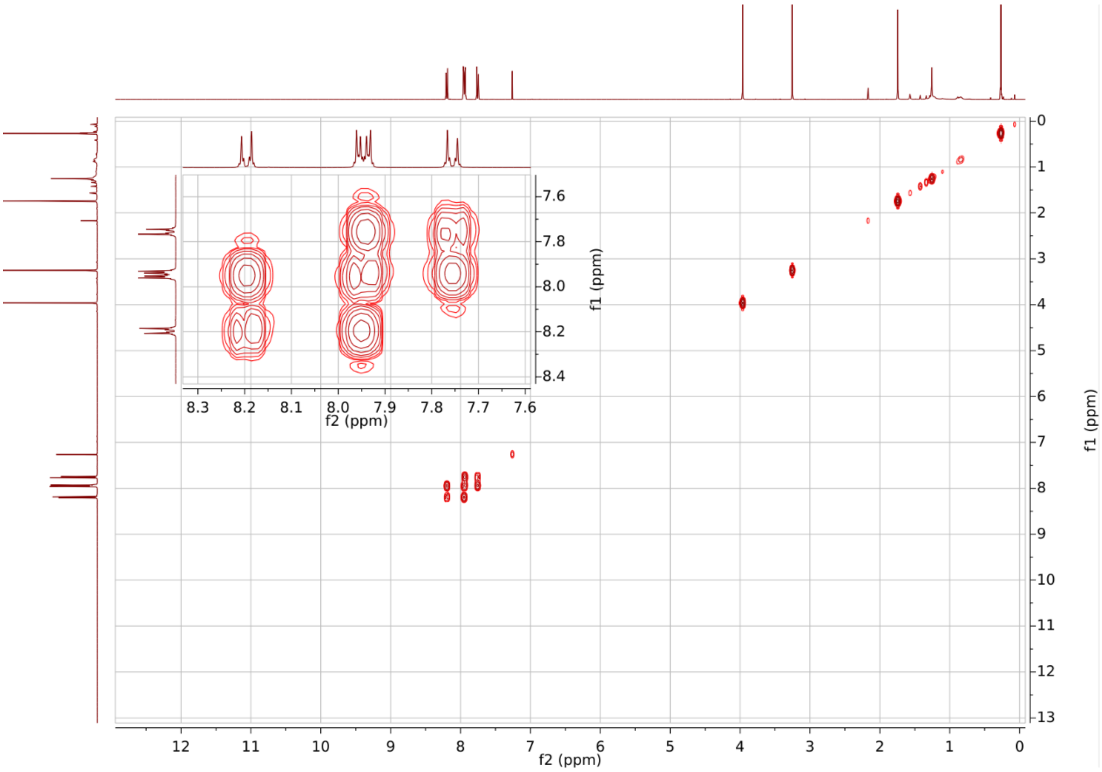

Compound S15b (^{13}C NMR, 101 MHz, $CDCl_3$)

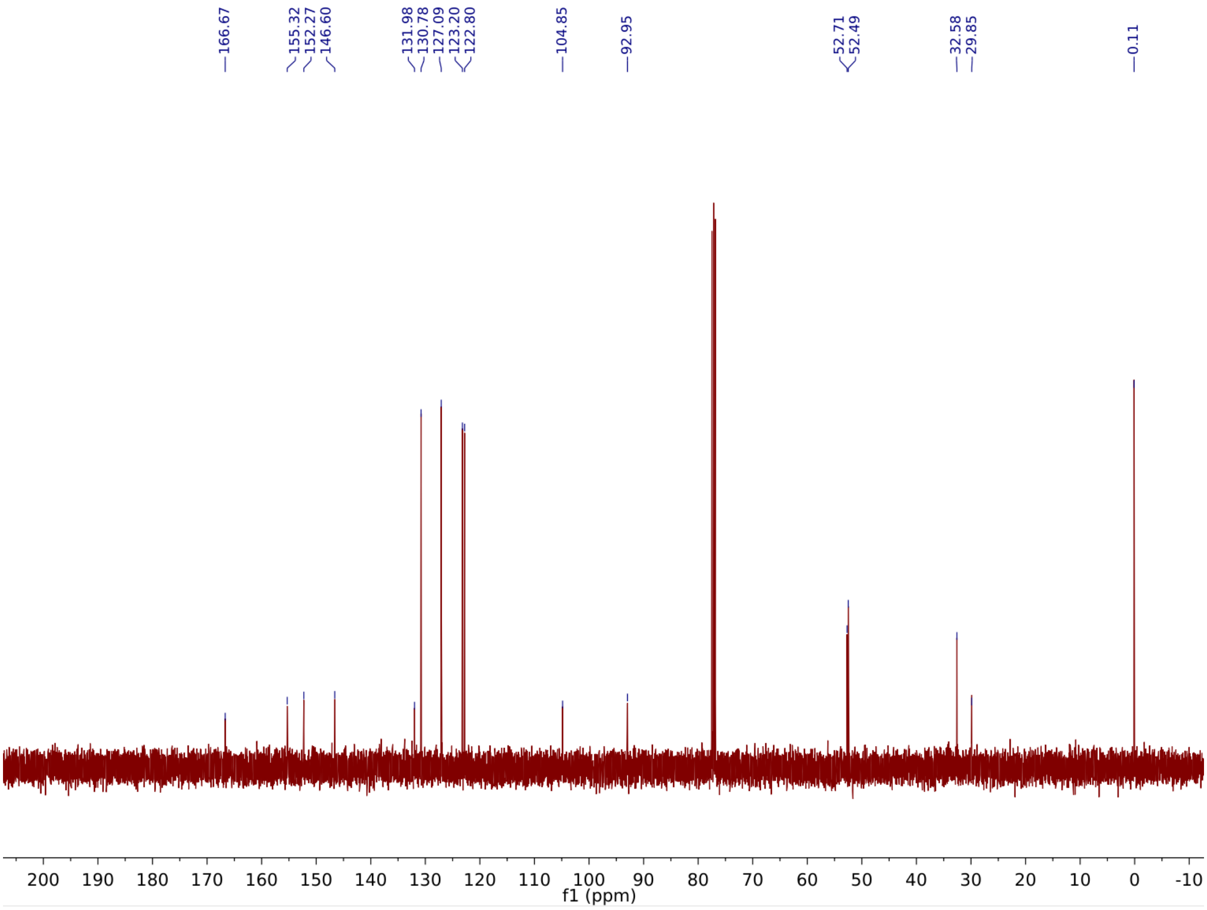

Compound S16a (^{1}H NMR, 400 MHz, $CDCl_3$)

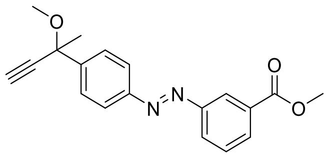

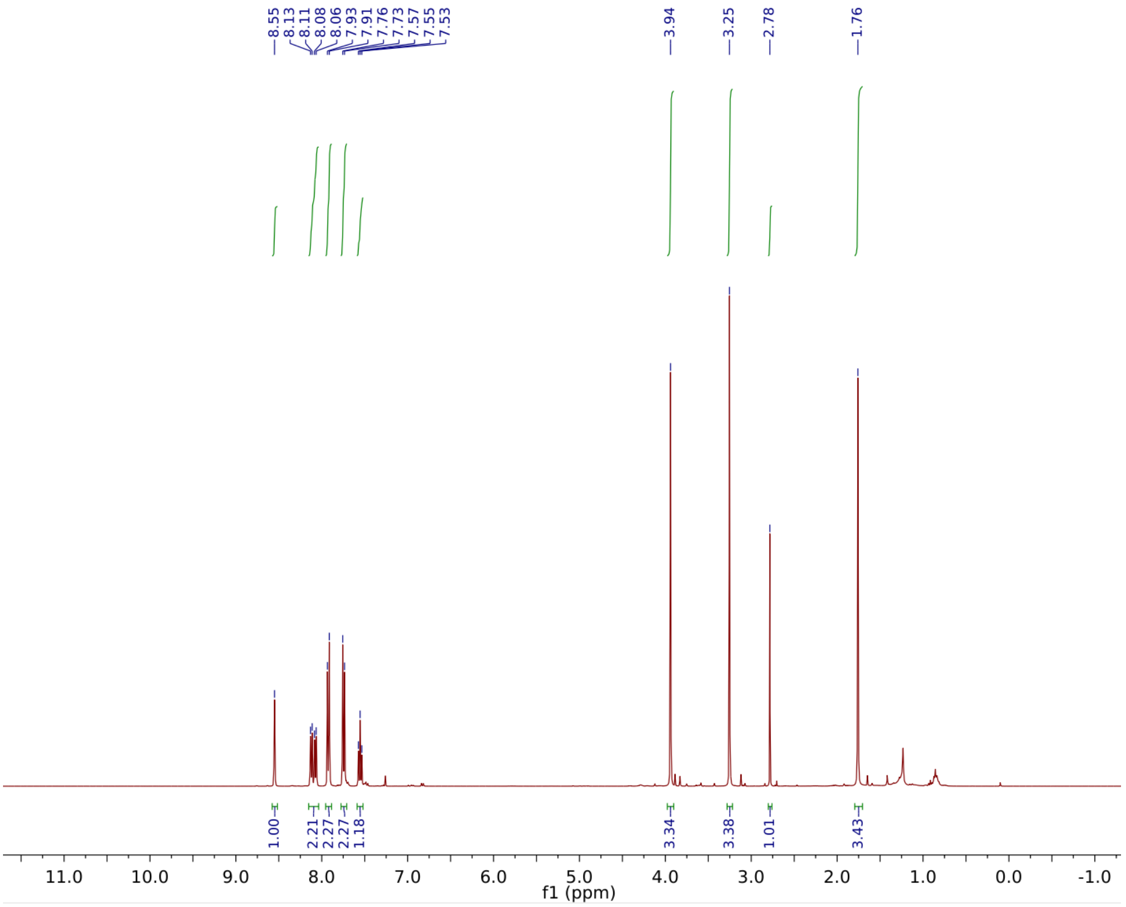

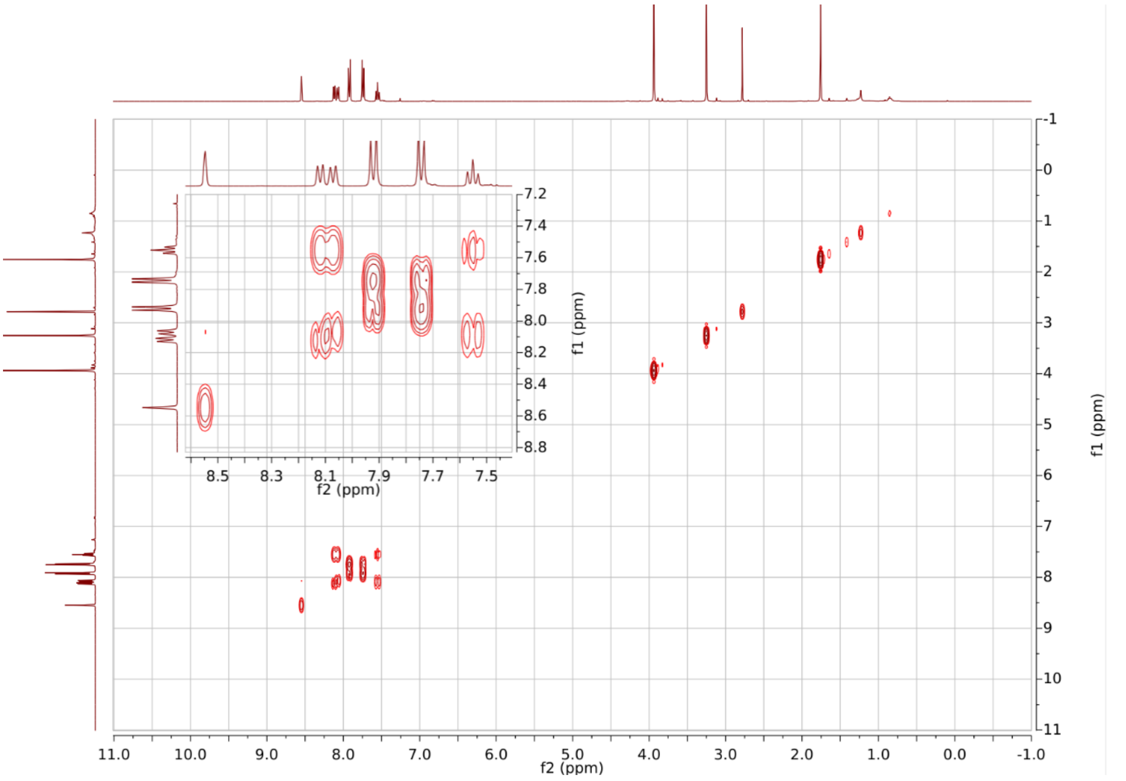

Compound S16a (^{13}C NMR, 101 MHz, $CDCl_3$)

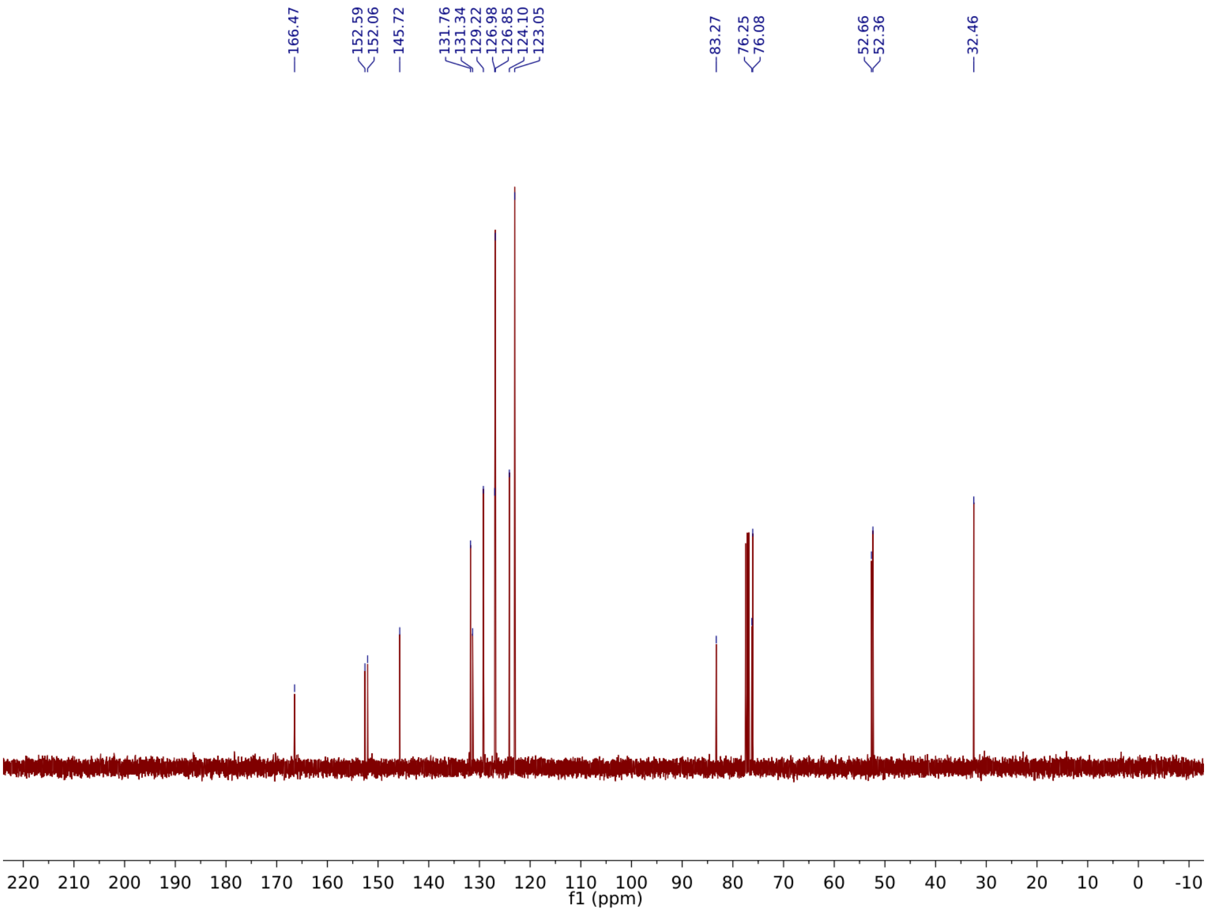

Compound S16b (^{1}H NMR, 400 MHz, $CDCl_3$)

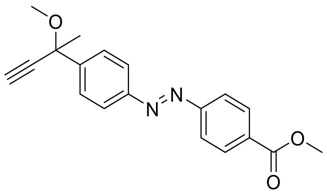

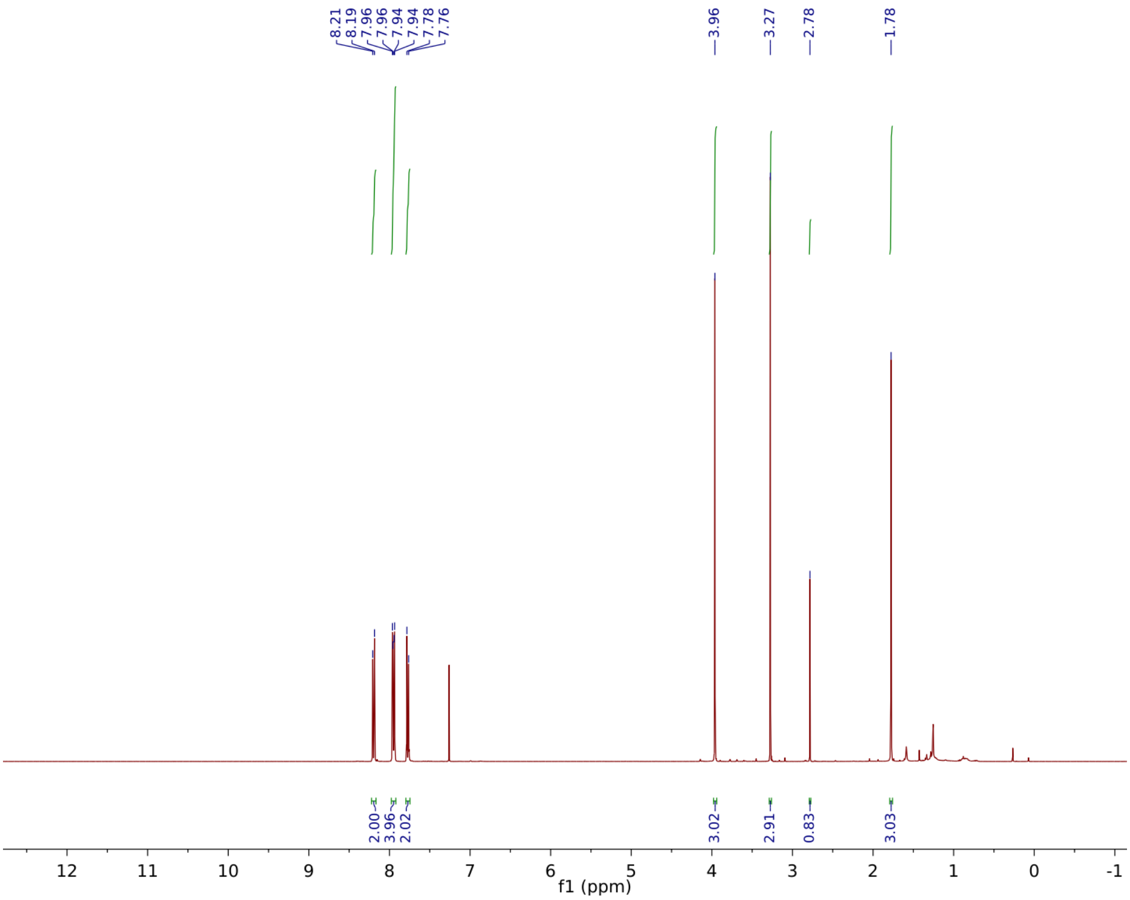

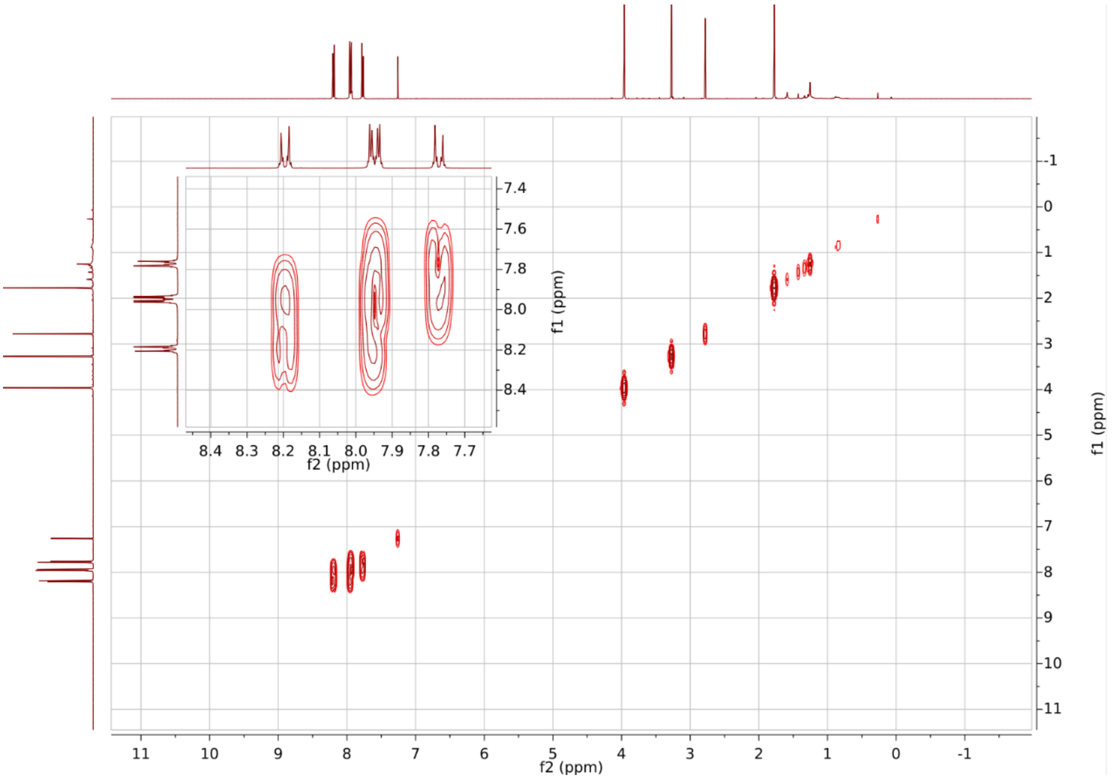

Compound S16b (^{13}C NMR, 101 MHz, $CDCl_3$)

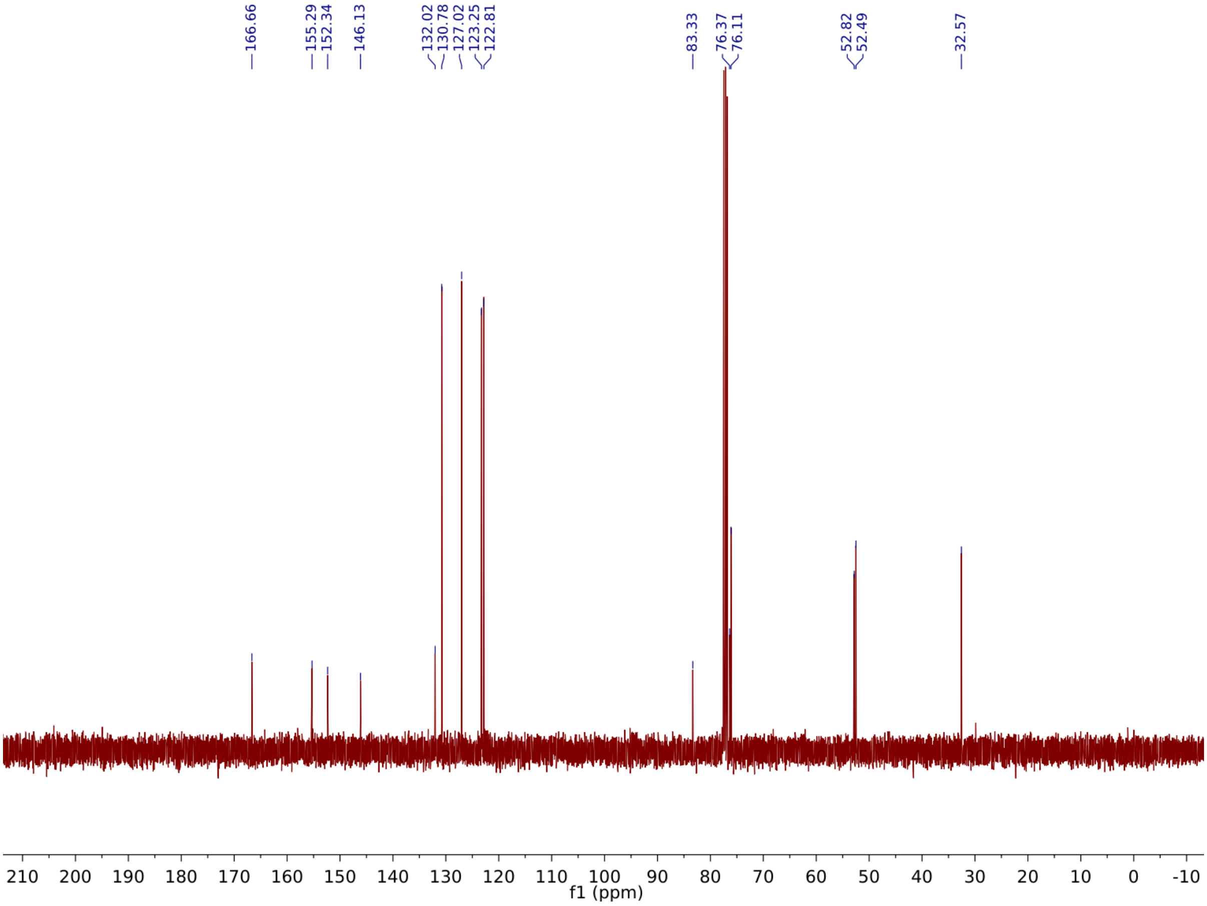

Compound S17a (^{1}H NMR, 400 MHz, DMSO-d_6)

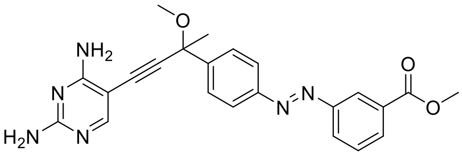

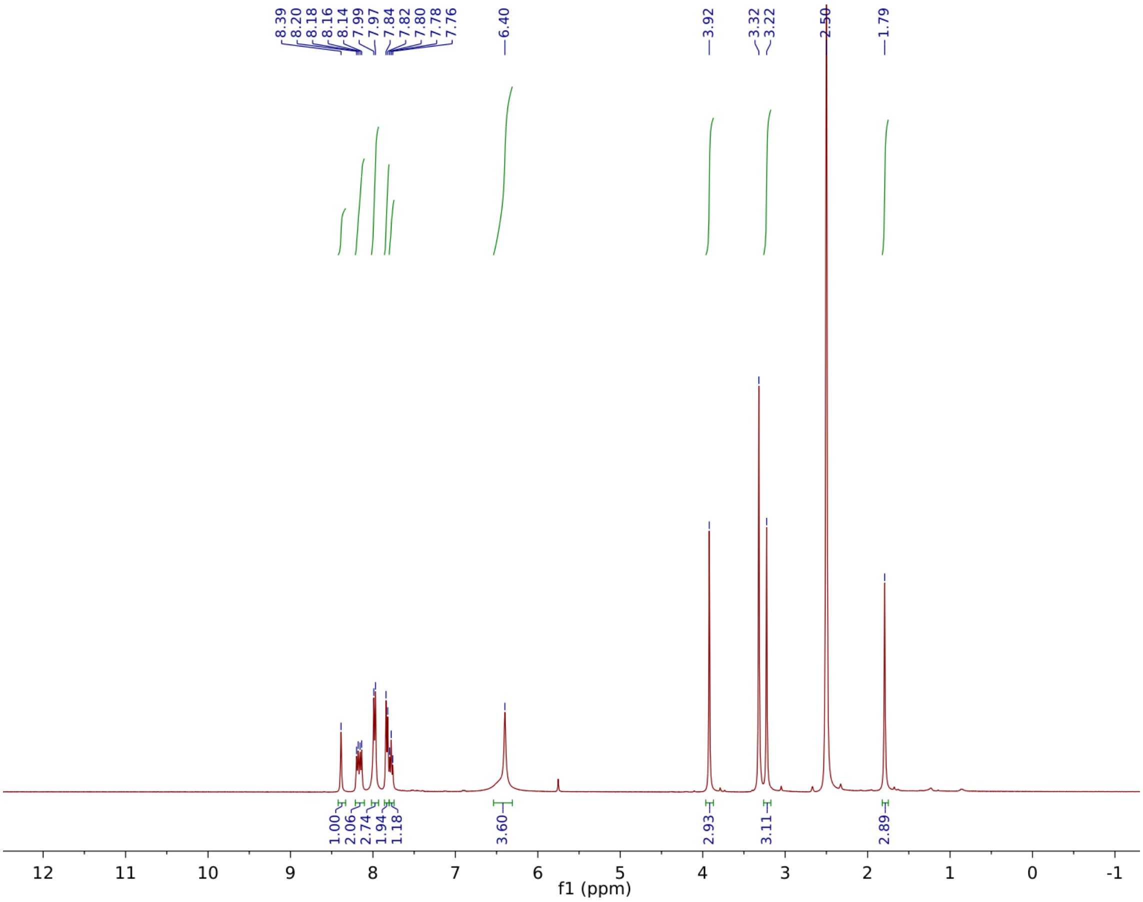

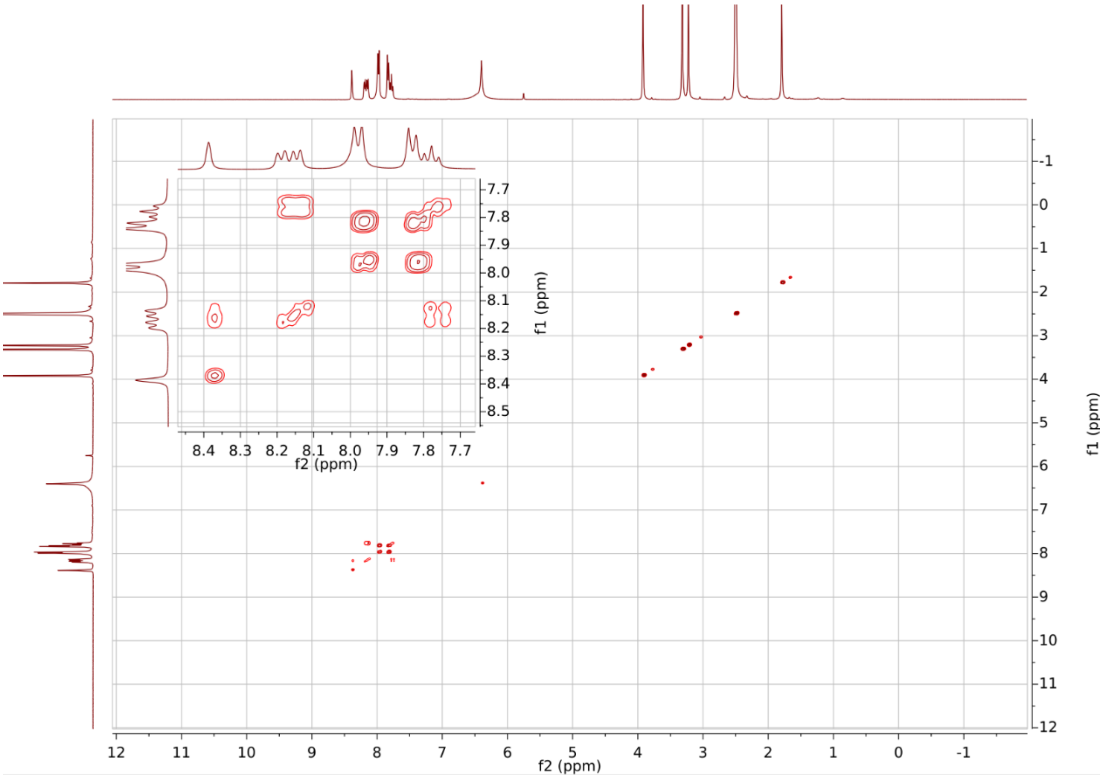

Compound S17a (^{13}C NMR, 101 MHz, DMSO-d_6)

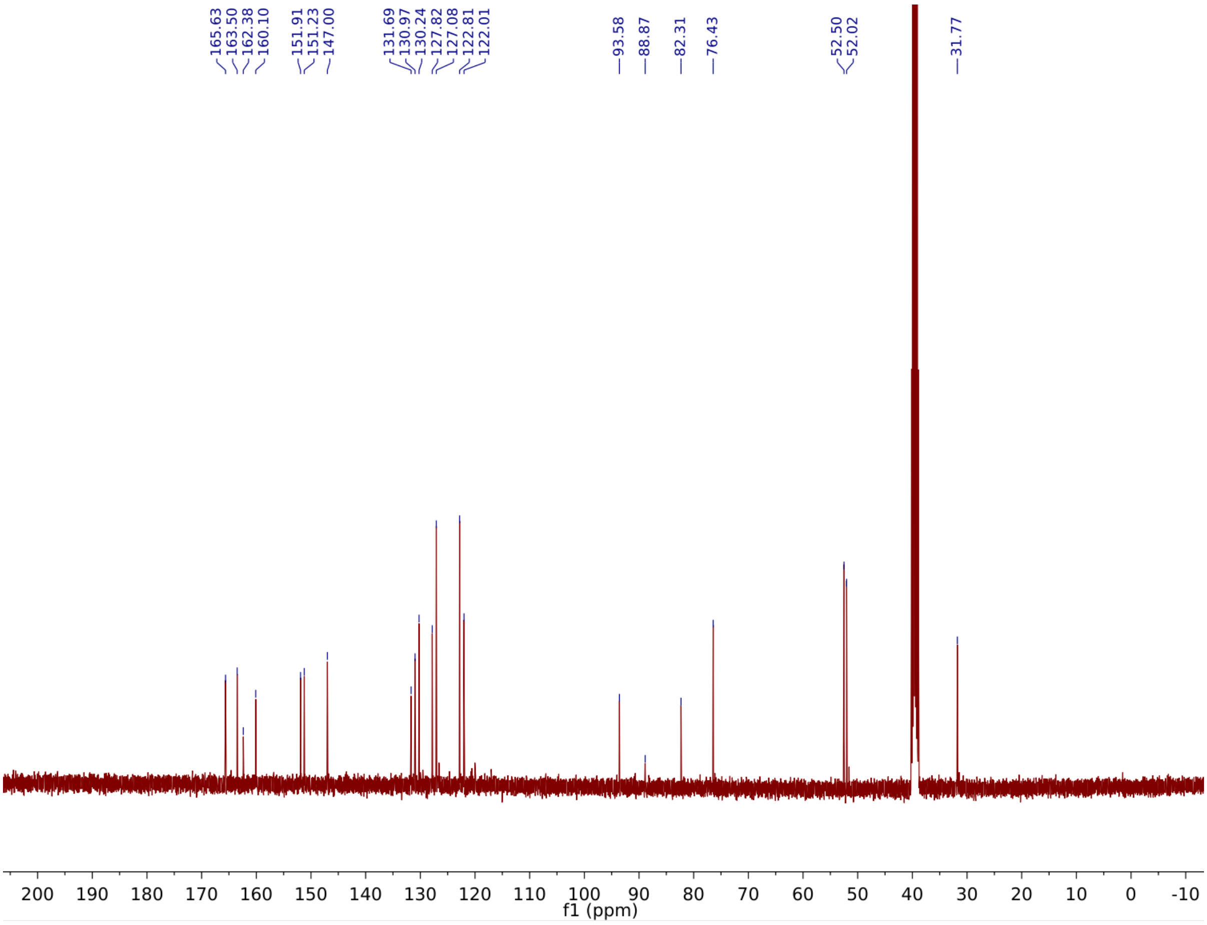

Compound S17b (^{1}H NMR, 400 MHz, DMSO-d_6)

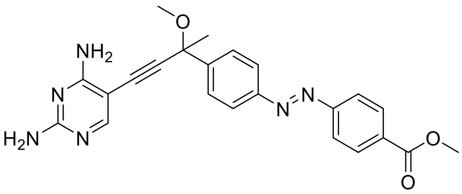

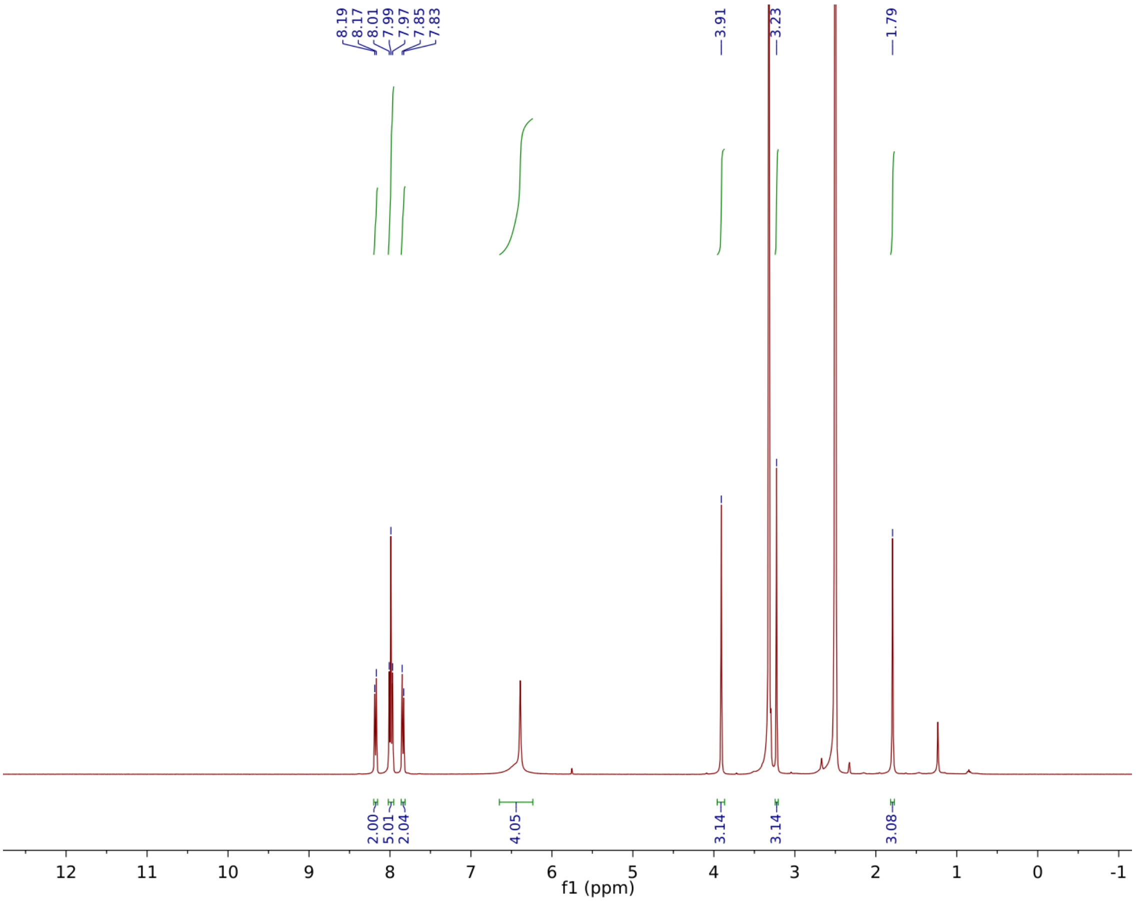

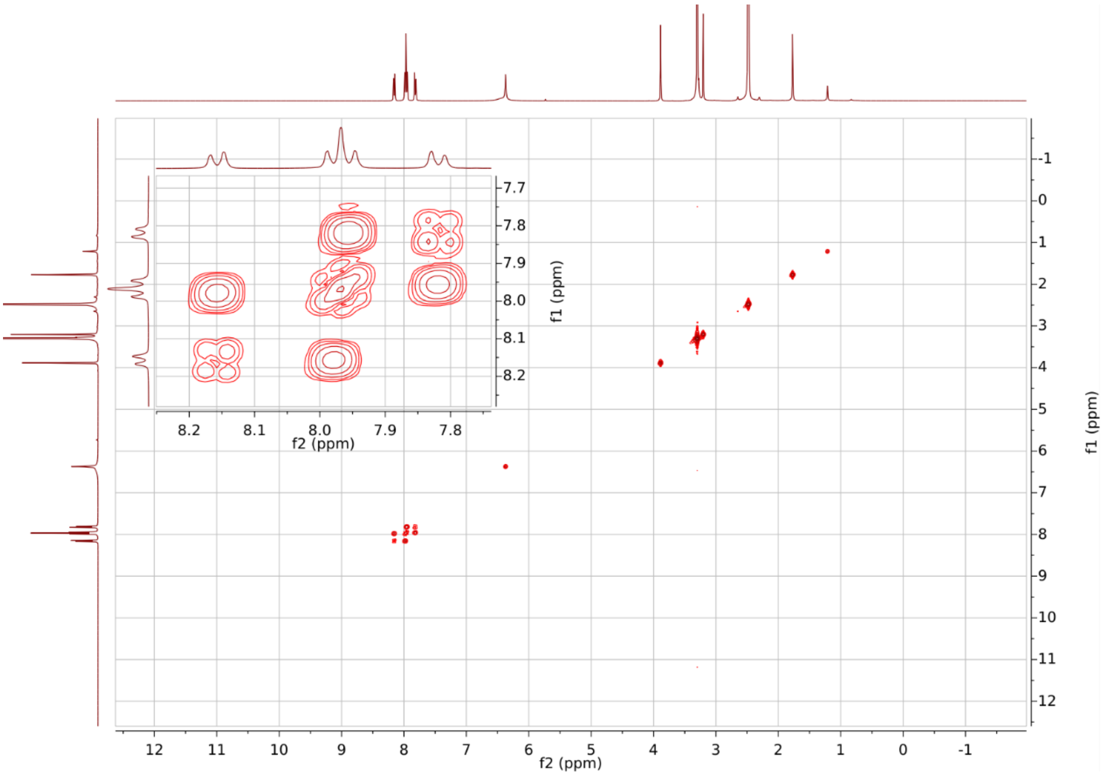

Compound S17b (^{13}C NMR, 101 MHz, DMSO-d_6)

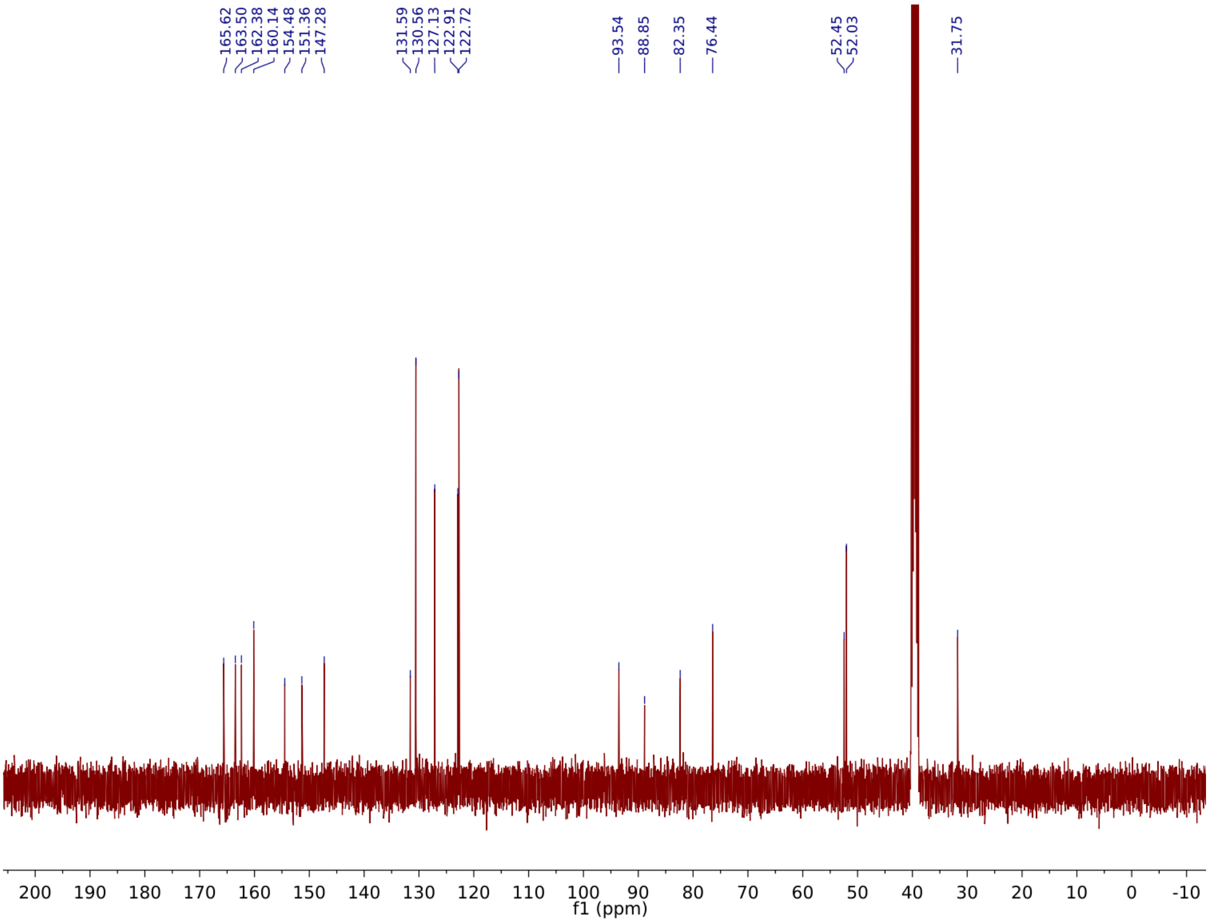

Compound 10 (^{1}H NMR, 400 MHz, DMSO-d_6)

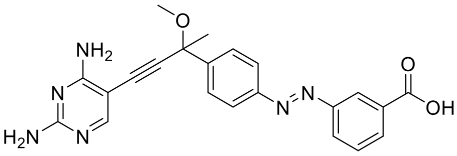

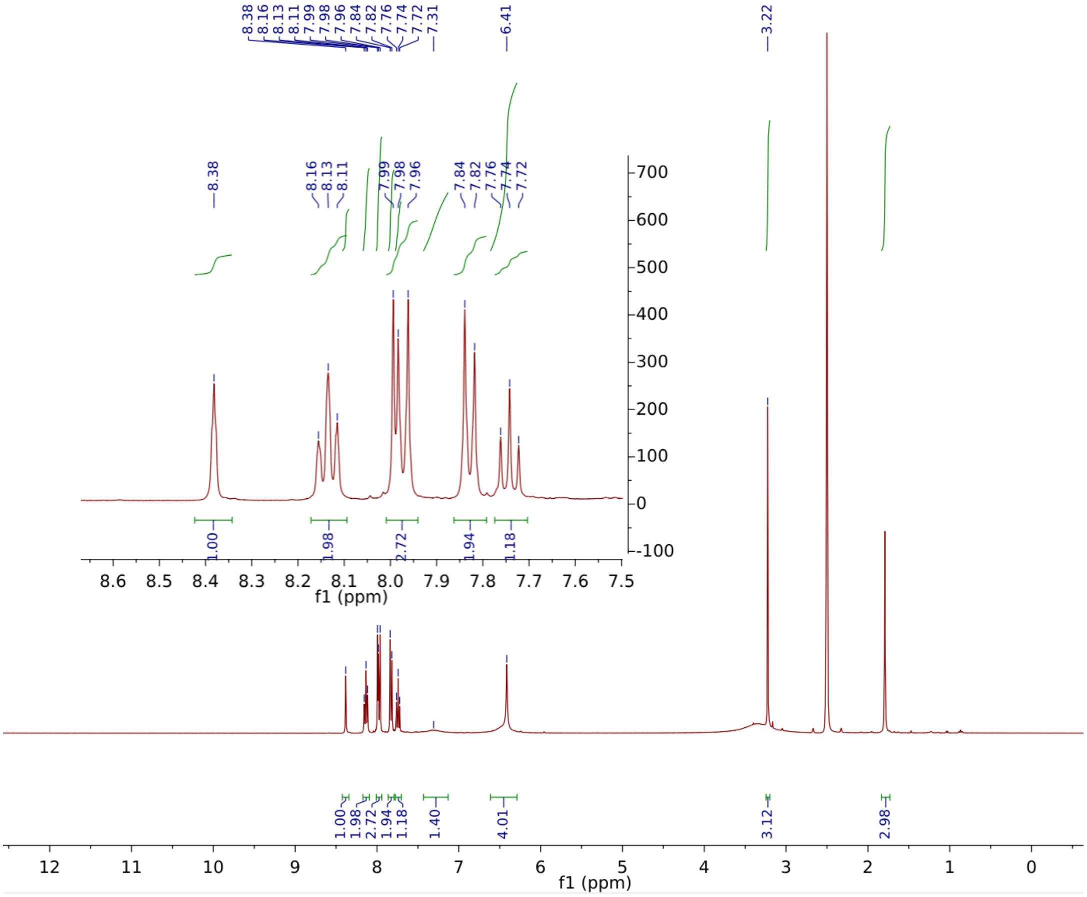

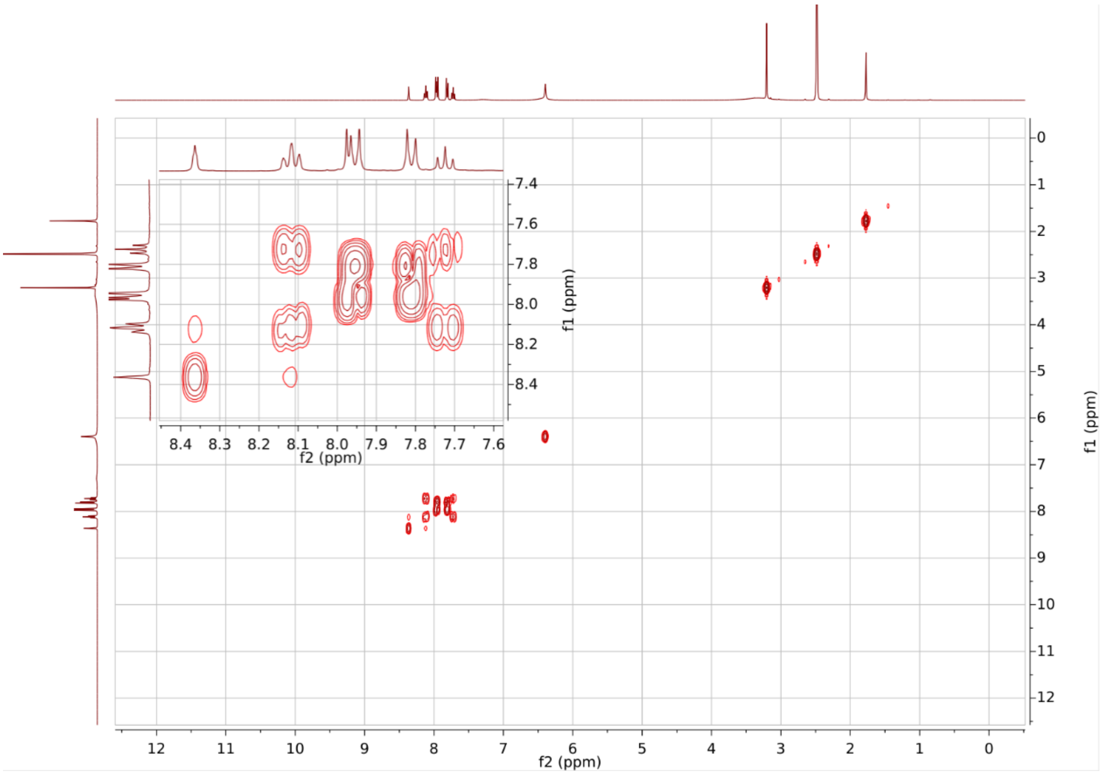

Compound 10 (^{13}C NMR, 101 MHz, DMSO-d_6)

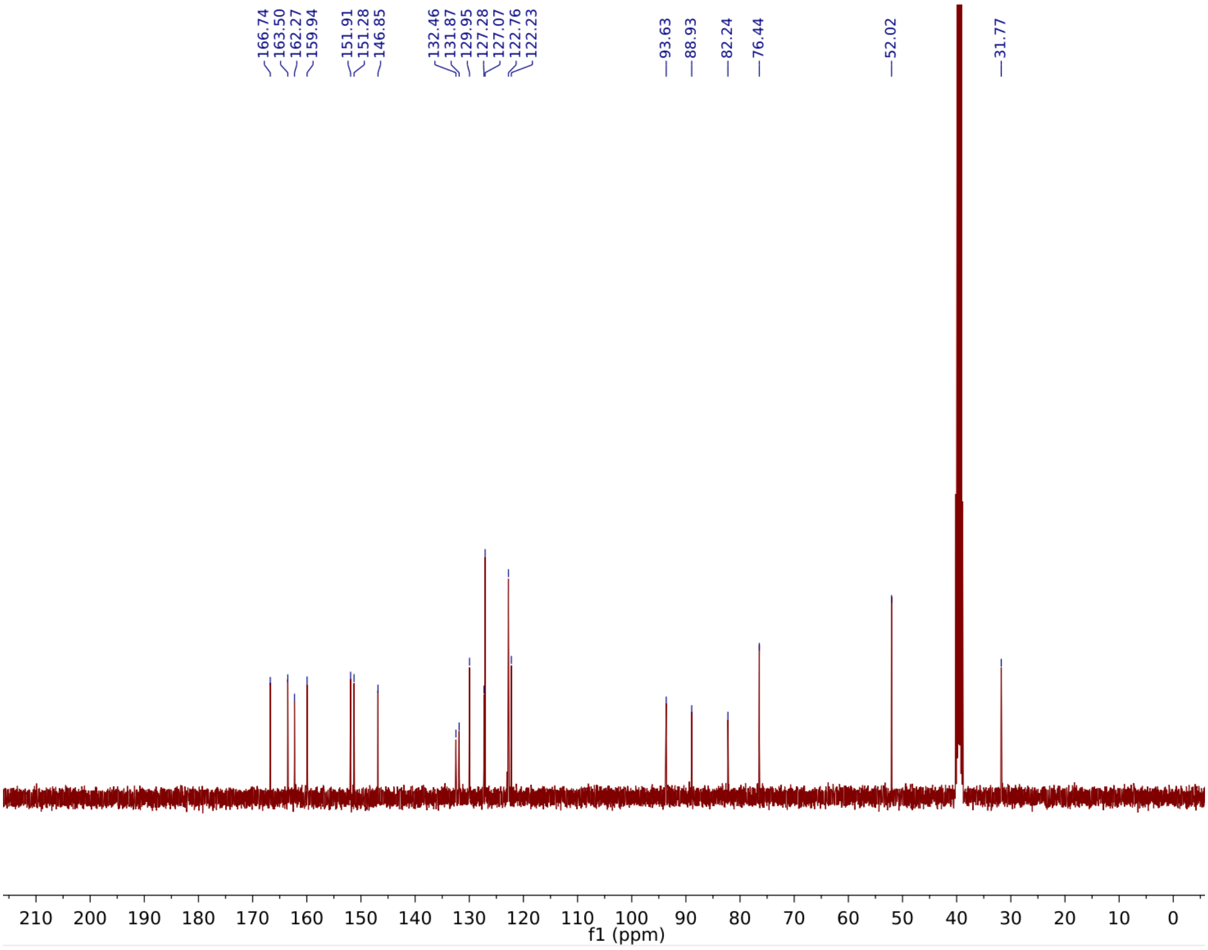

Compound 10 (HRMS)

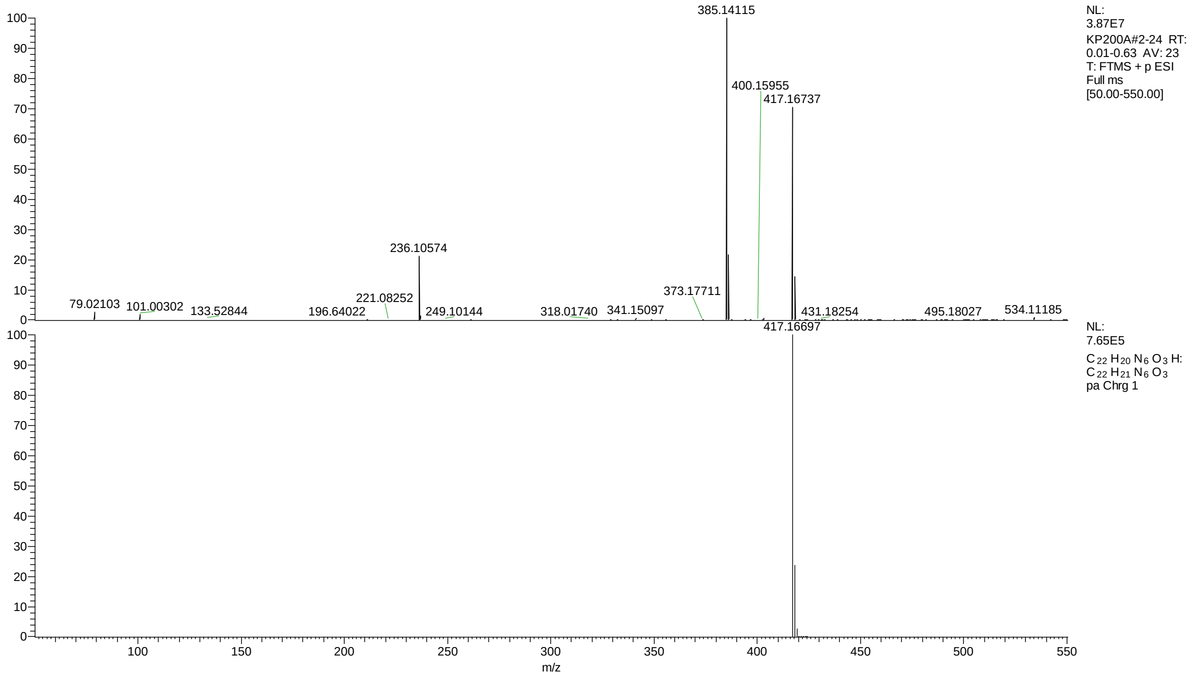

Compound 11 (^{1}H NMR, 400 MHz, DMSO-d_6)

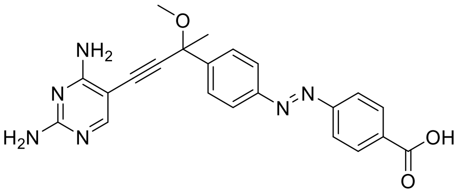

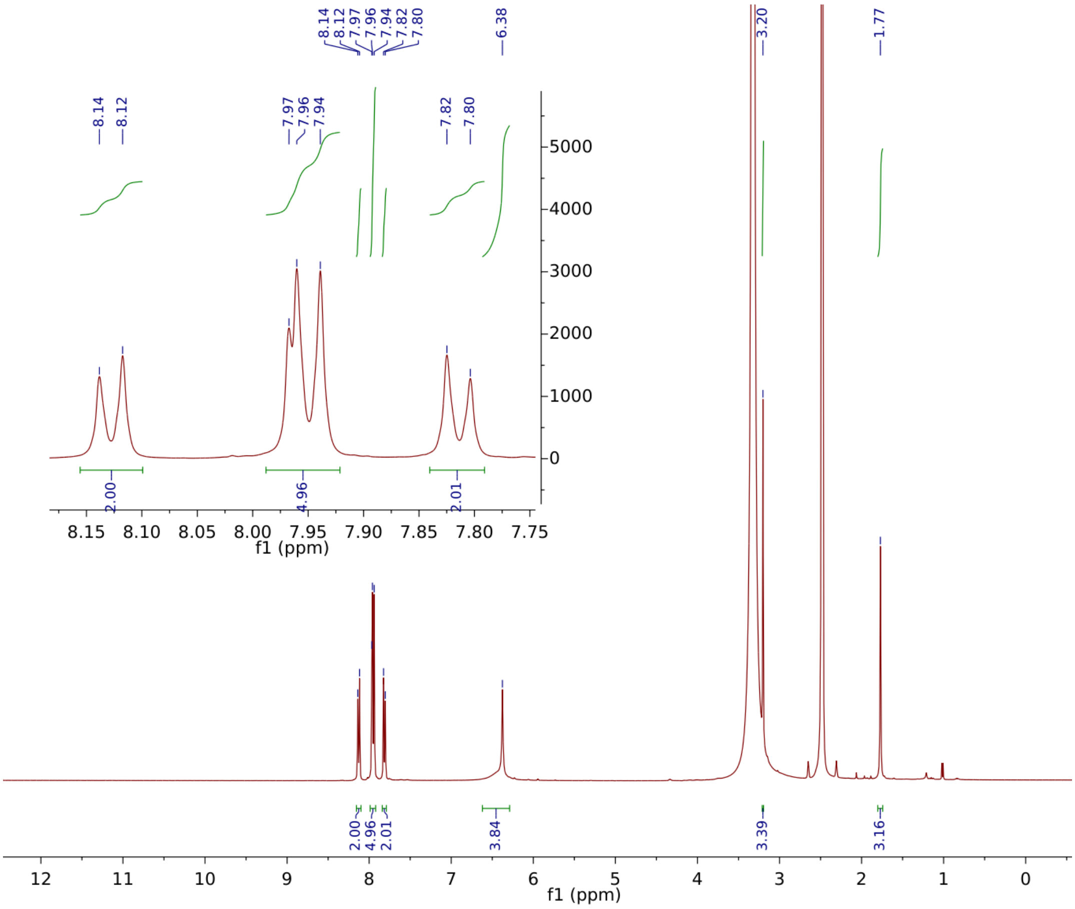

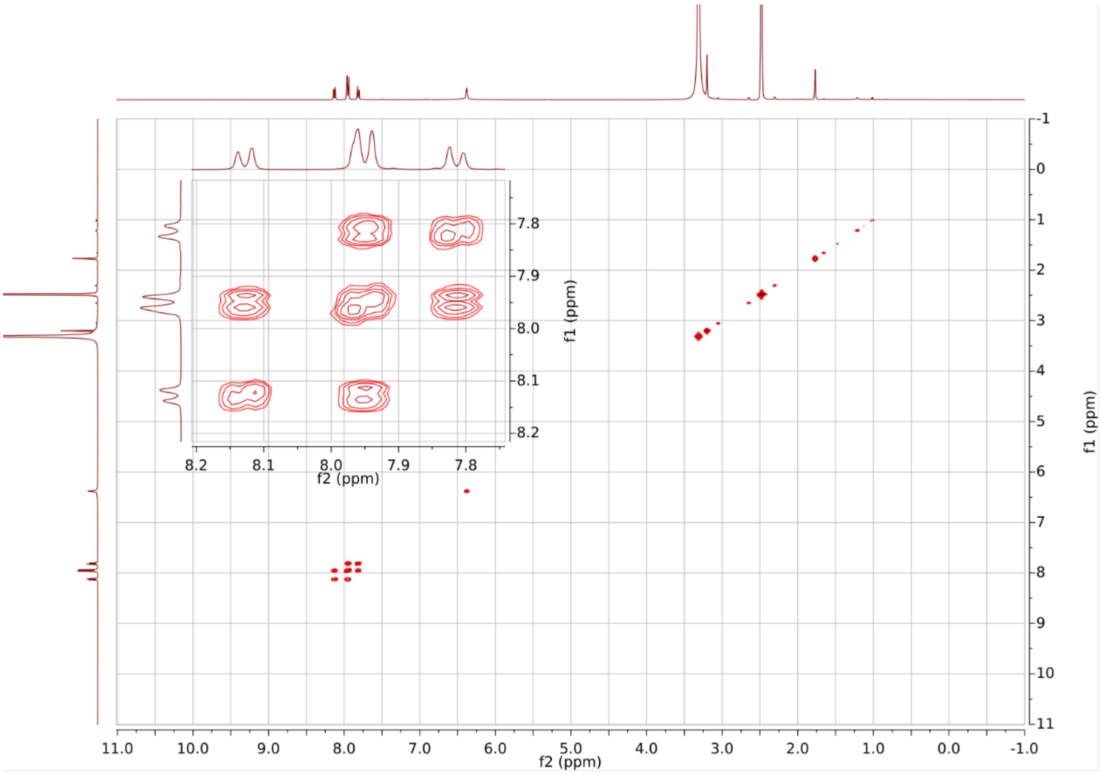

Compound 11 (^{13}C NMR, 101 MHz, DMSO-d_6)

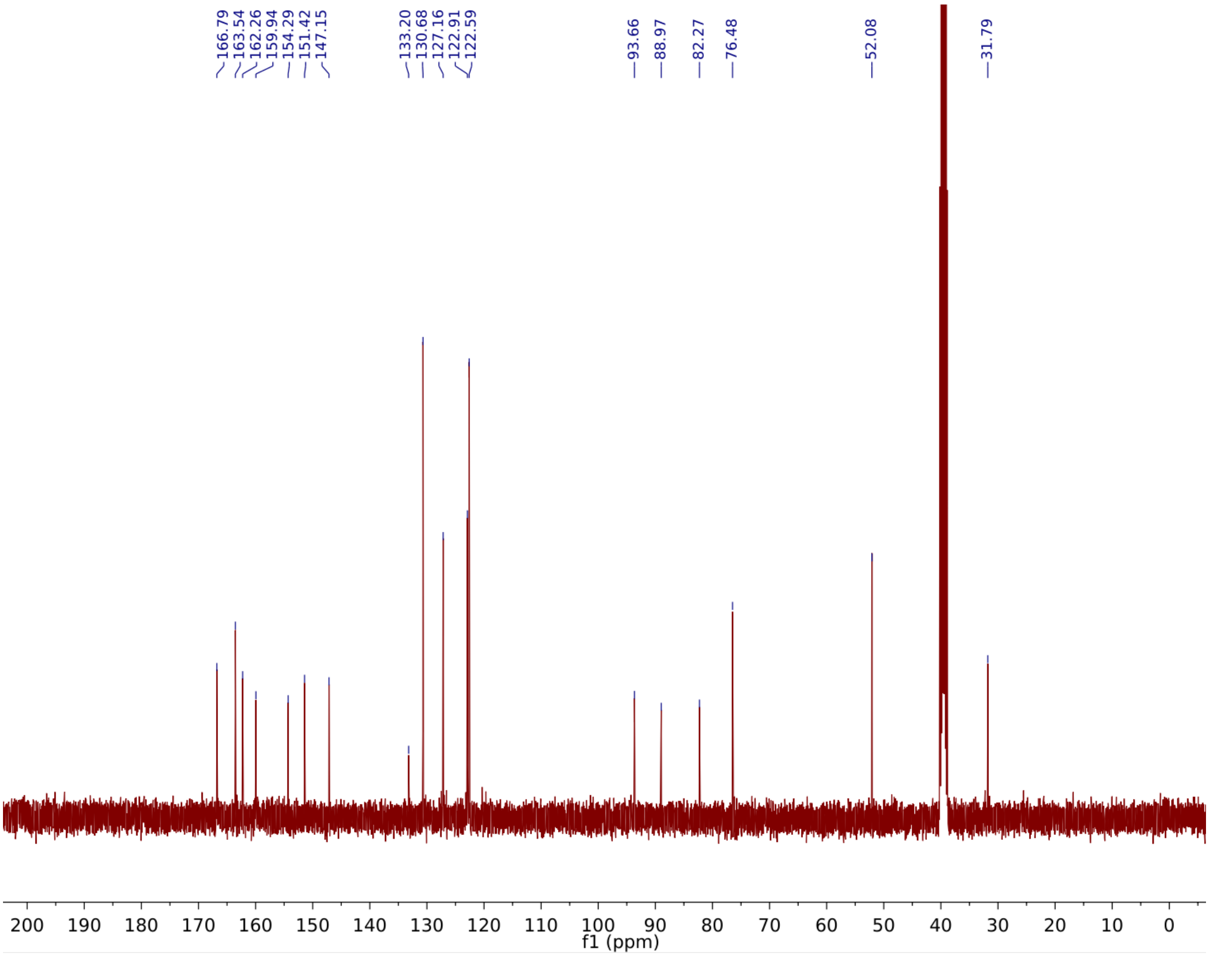

Compound 11 (HRMS)

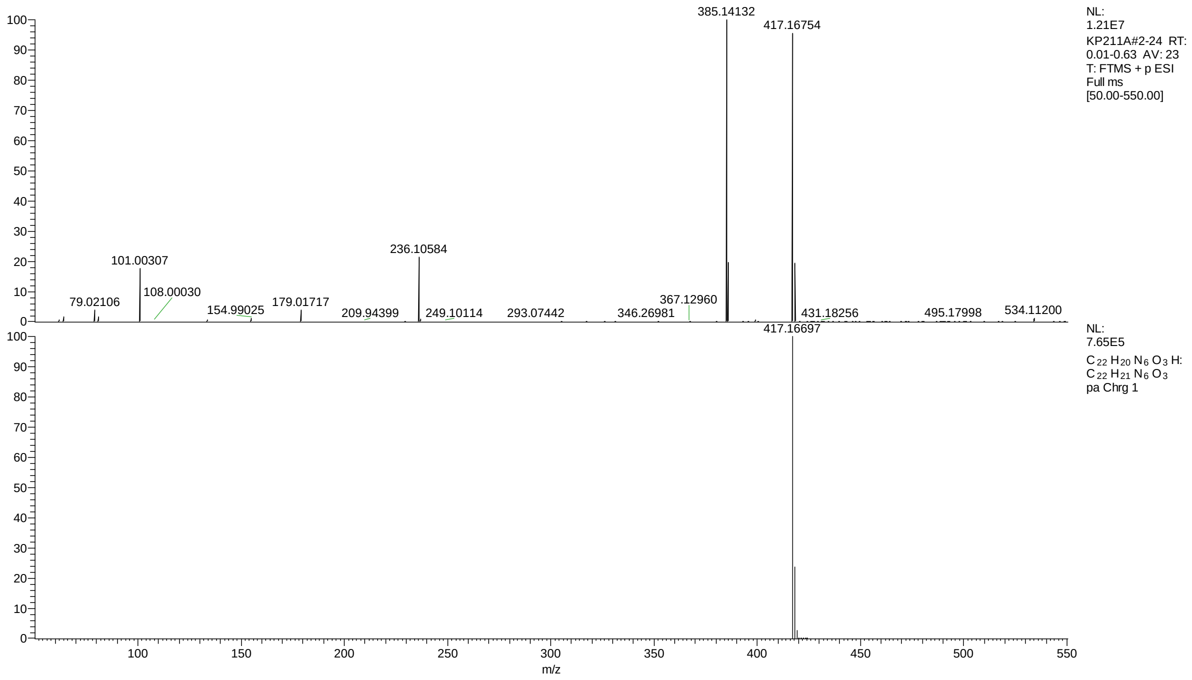

S7.5. Fifth hypothesis

Compound S18a (^{1}H NMR, 400 MHz, $CDCl_3$)

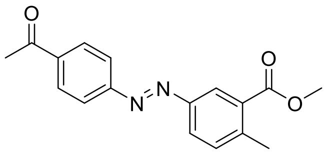

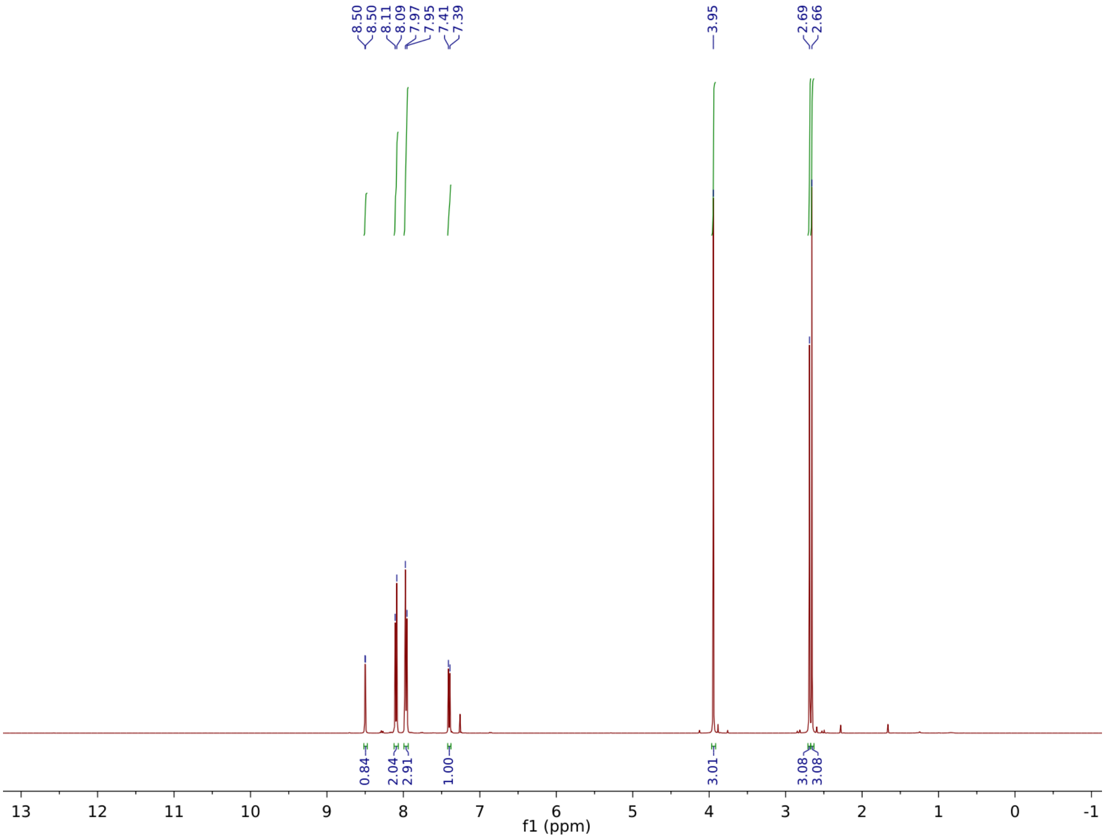

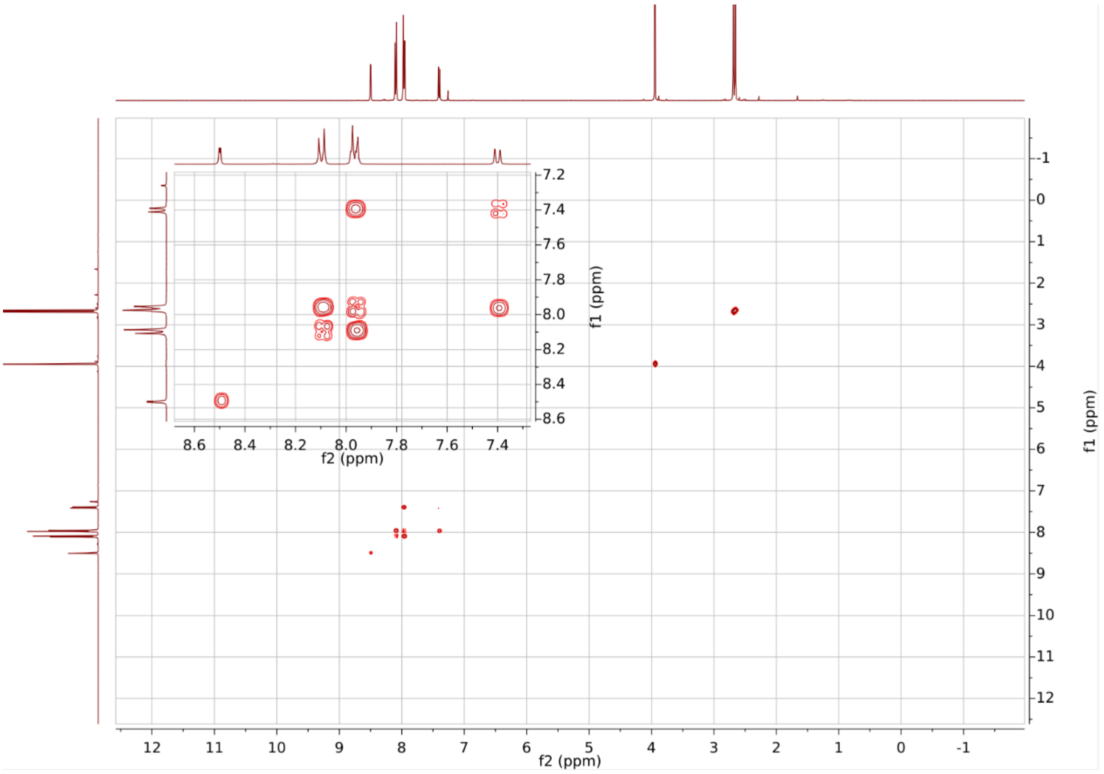

Compound S18a (^{13}C NMR, 101 MHz, $CDCl_3$)

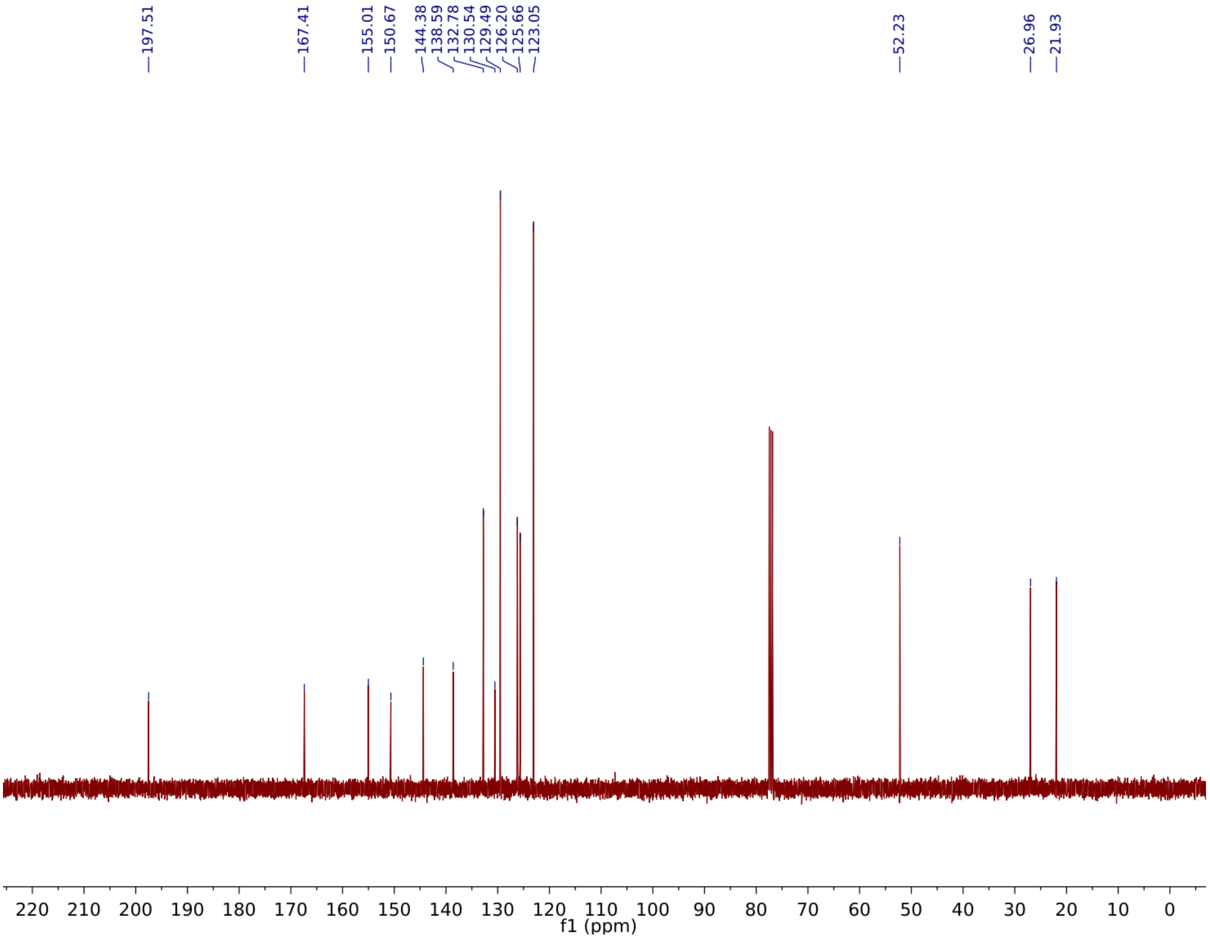

Compound S18b (^{1}H NMR, 400 MHz, $CDCl_3$)

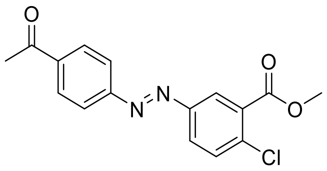

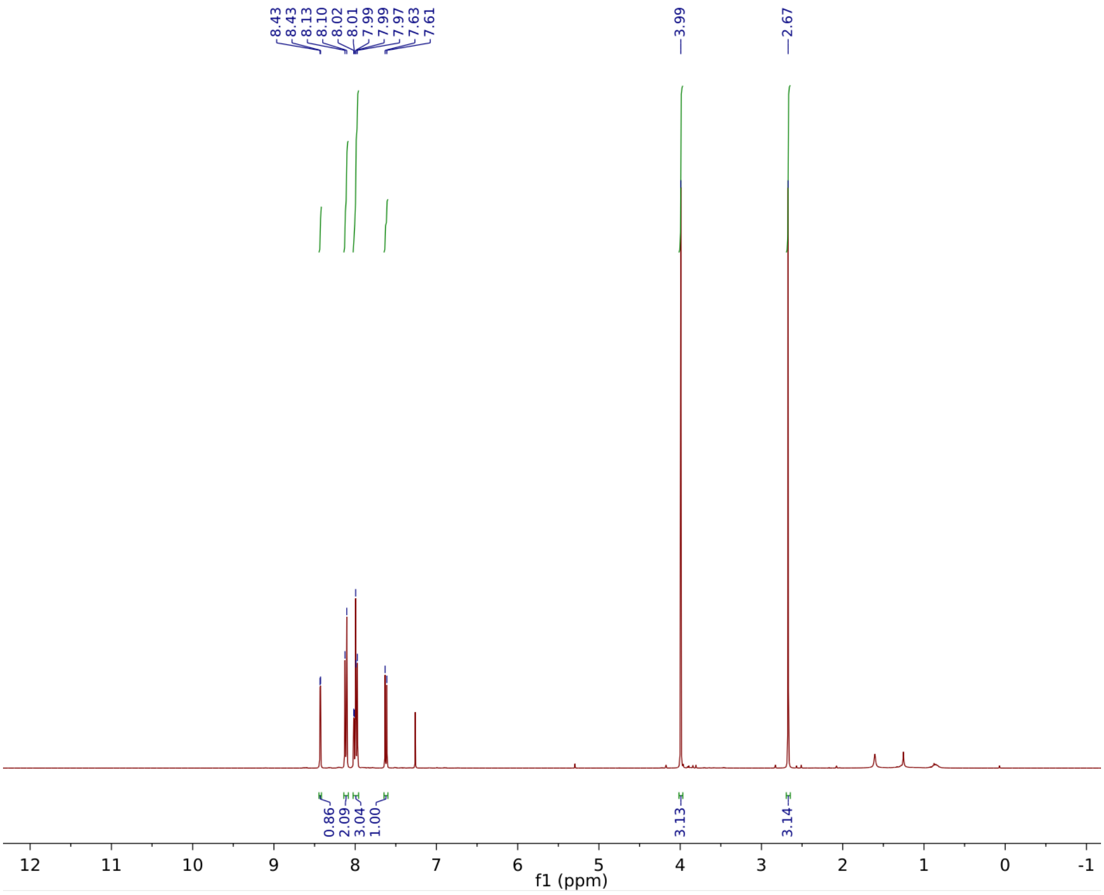

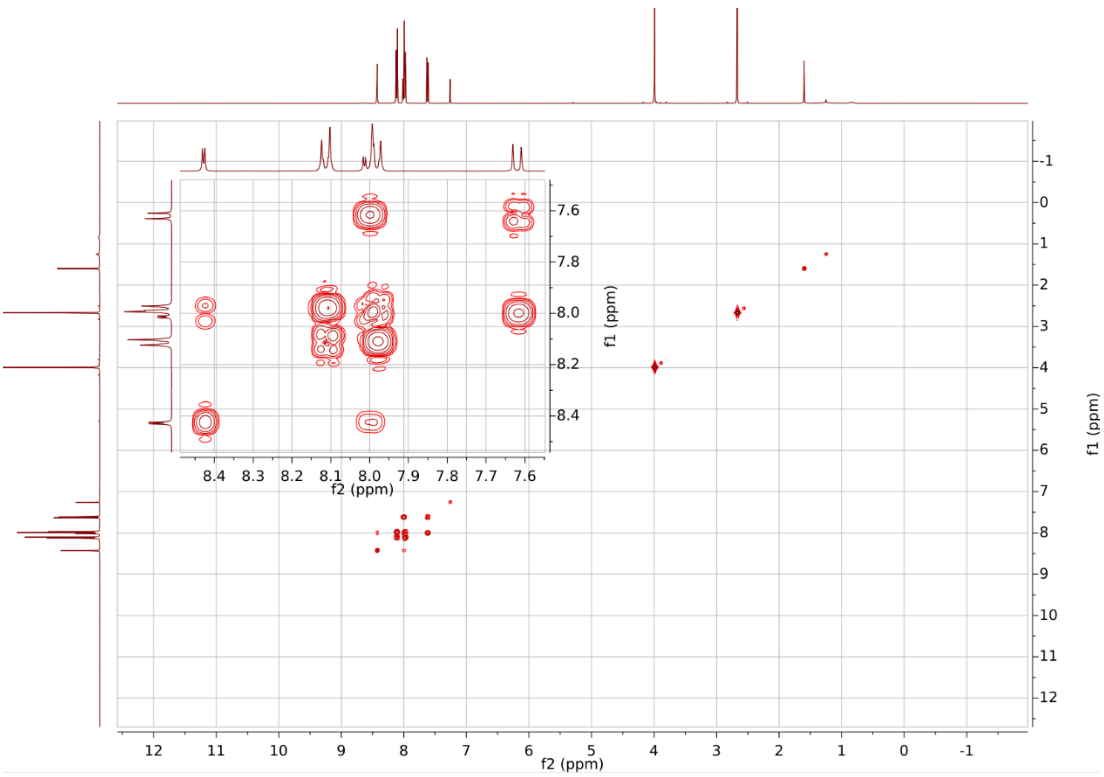

Compound S18b (^{13}C NMR, 101 MHz, $CDCl_3$)

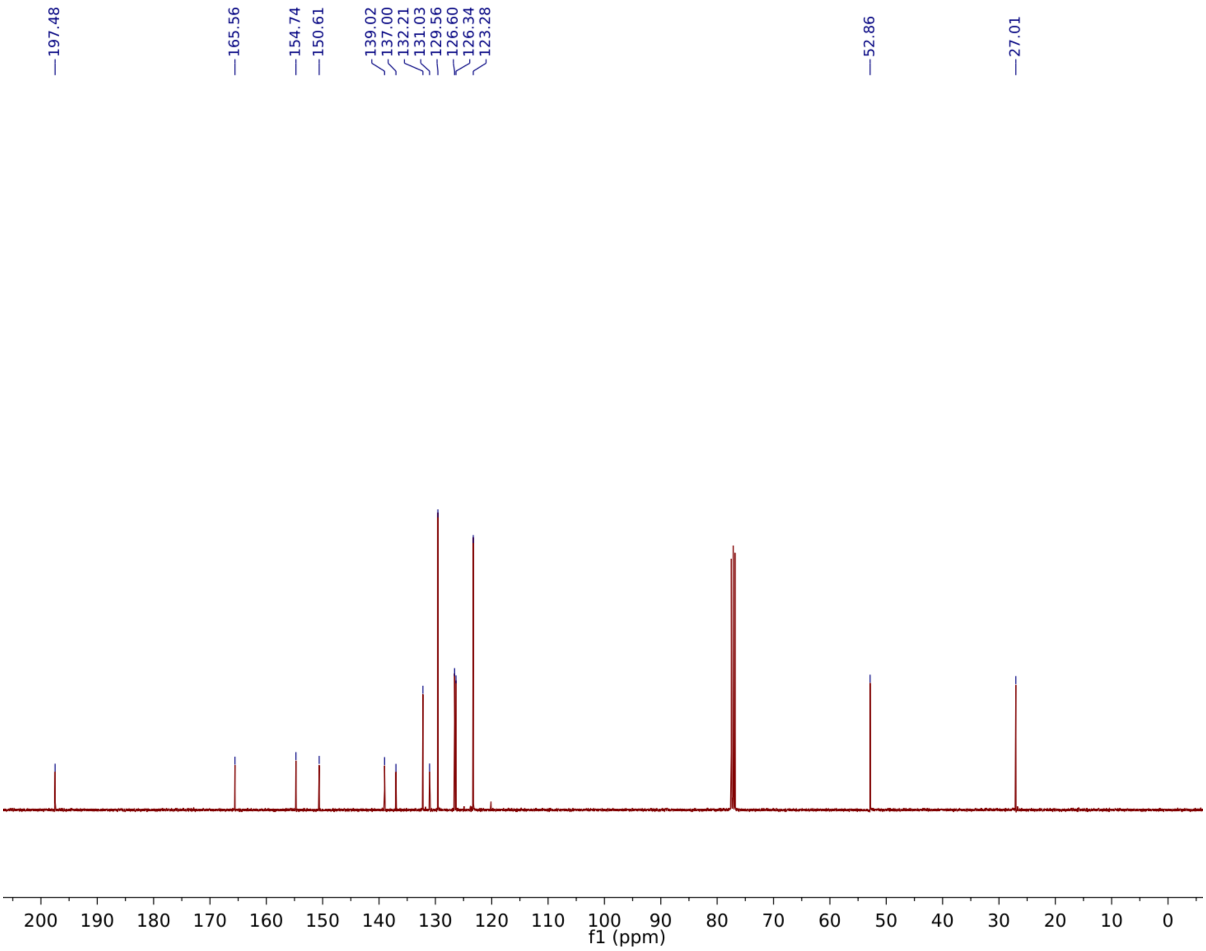

Compound S18c (^{1}H NMR, 400 MHz, $CDCl_3$)

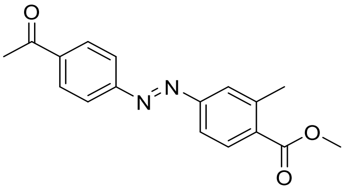

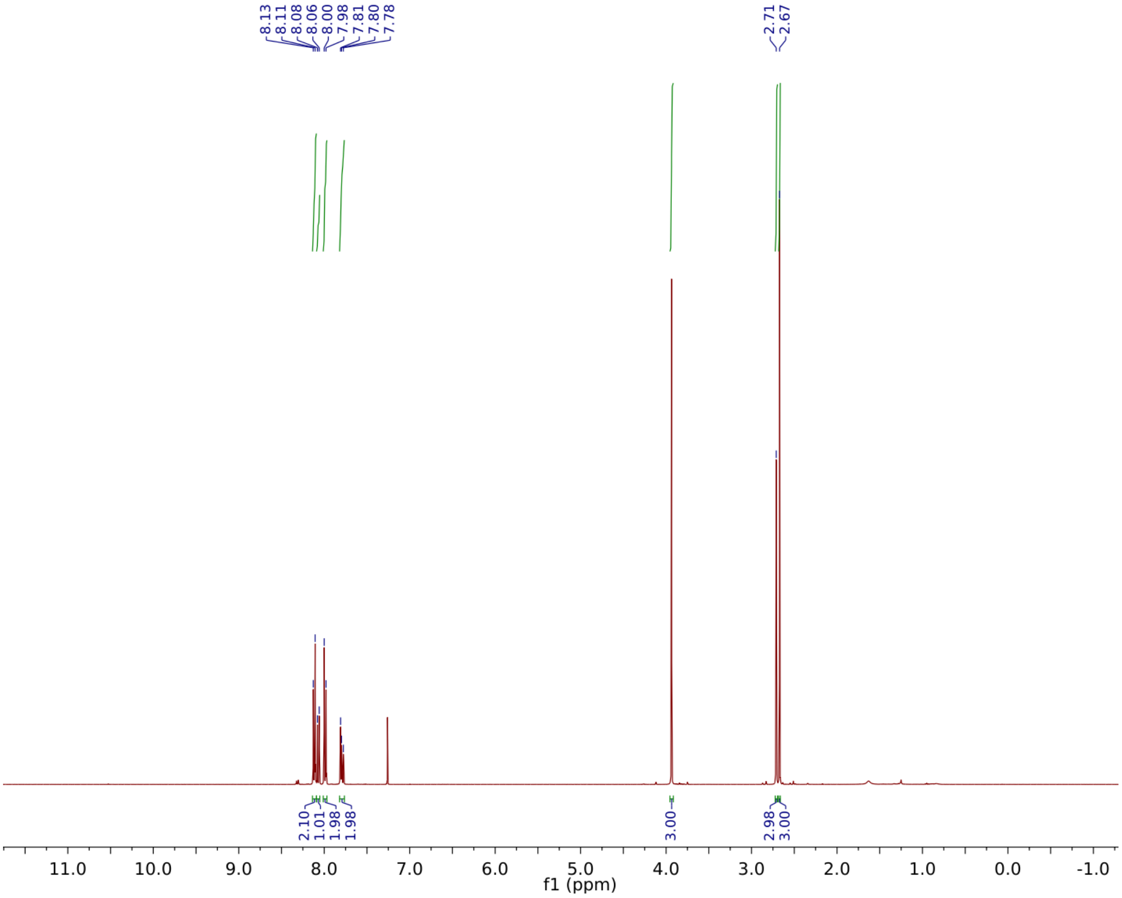

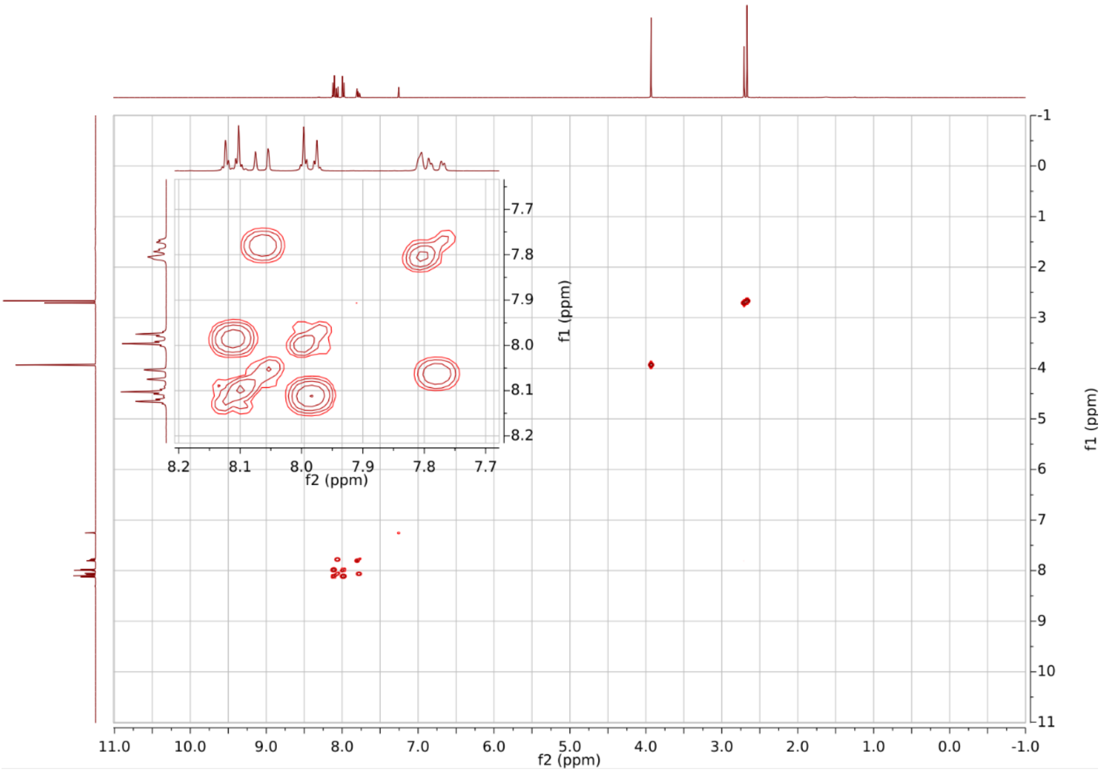

Compound S18c (^{13}C NMR, 101 MHz, $CDCl_3$)

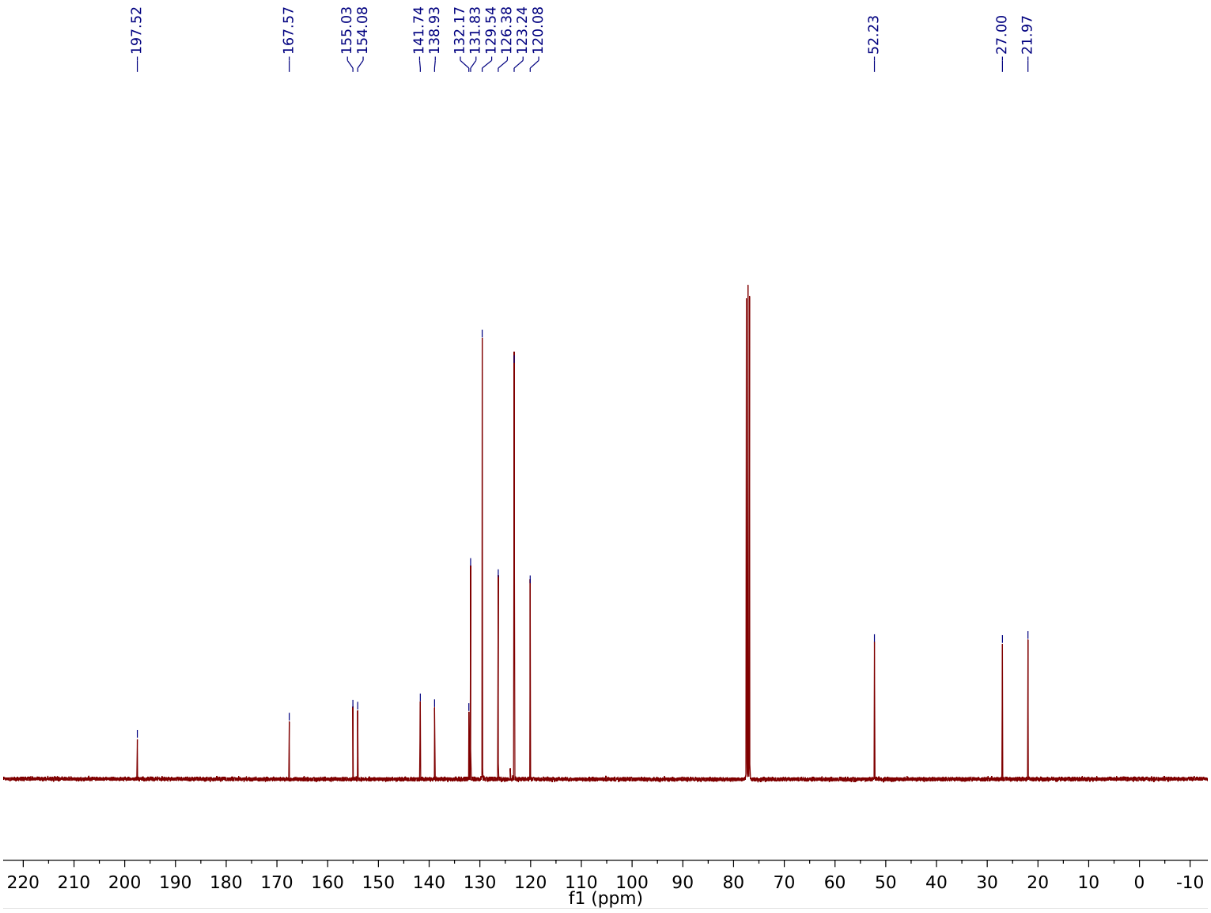

Compound S18d (^{1}H NMR, 400 MHz, $CDCl_3$)

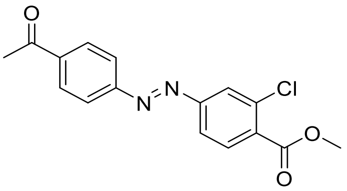

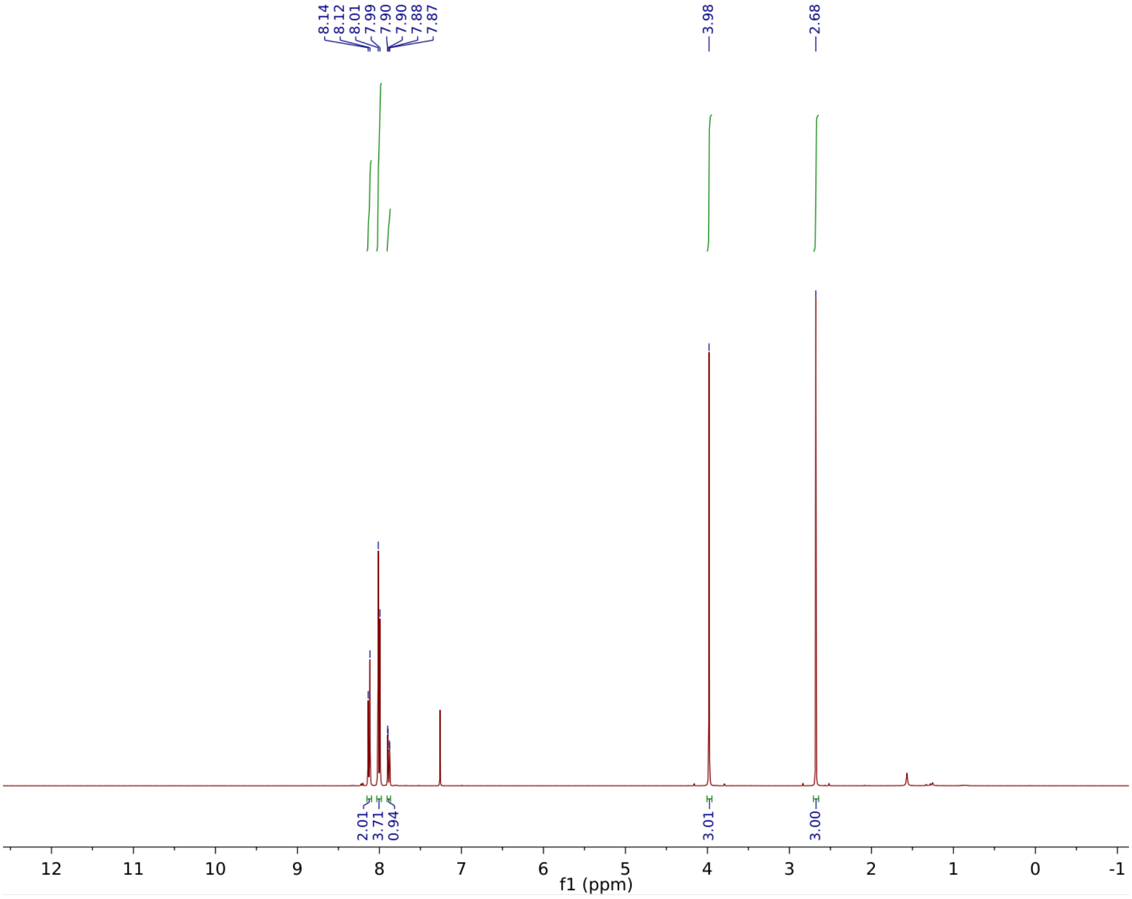

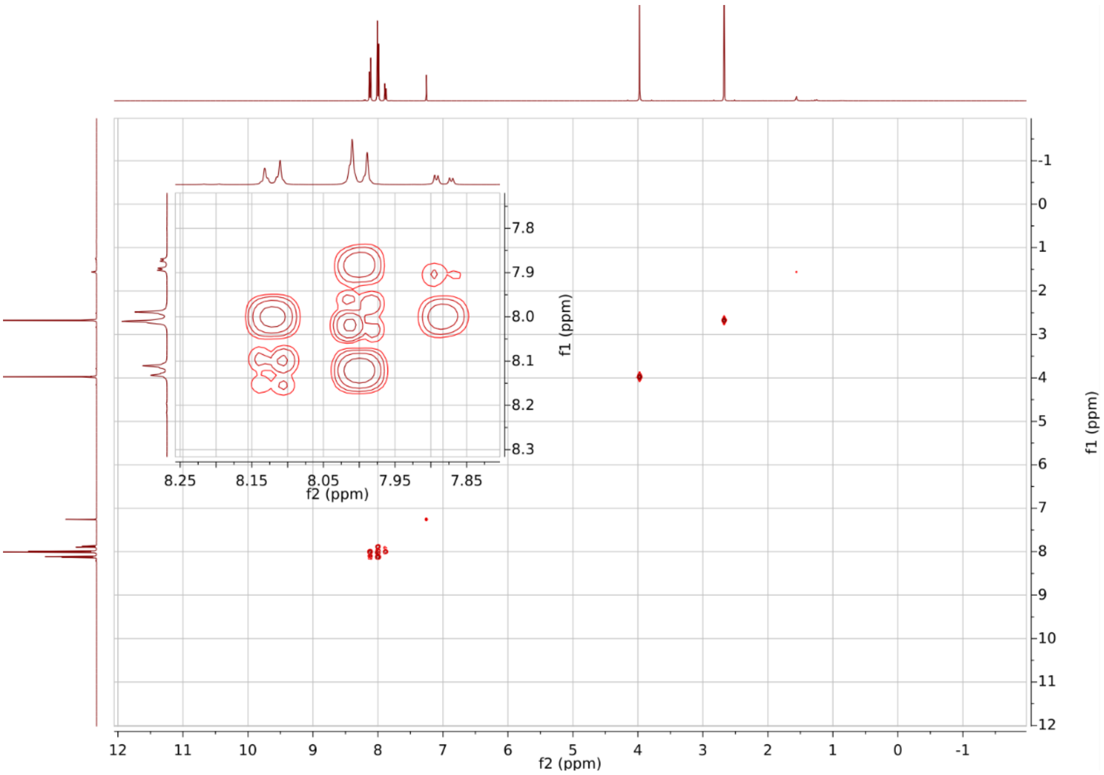

Compound S18d (^{13}C NMR, 101 MHz, $CDCl_3$)

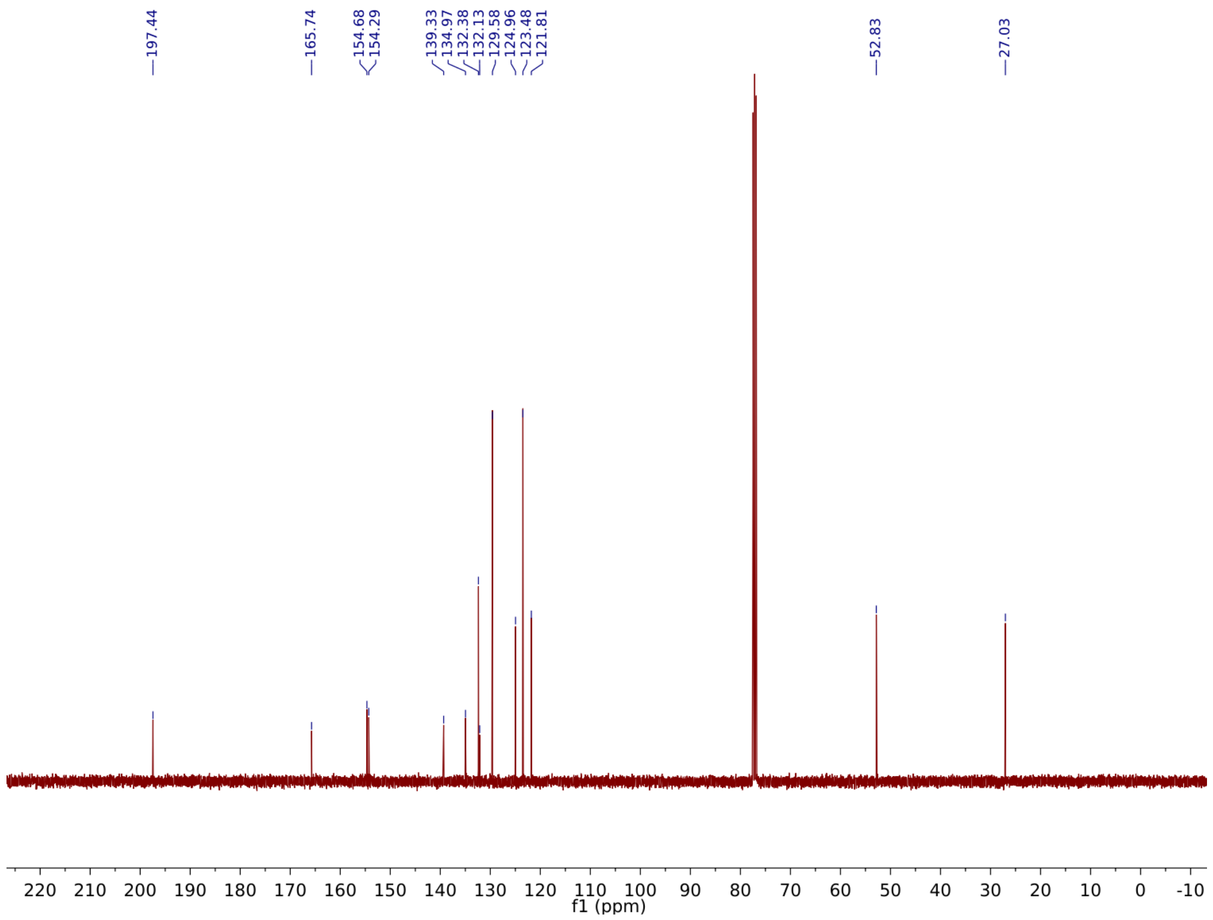

Compound S19a (^{1}H NMR, 400 MHz, $CDCl_3$)

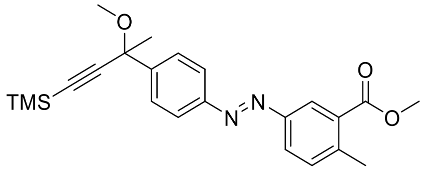

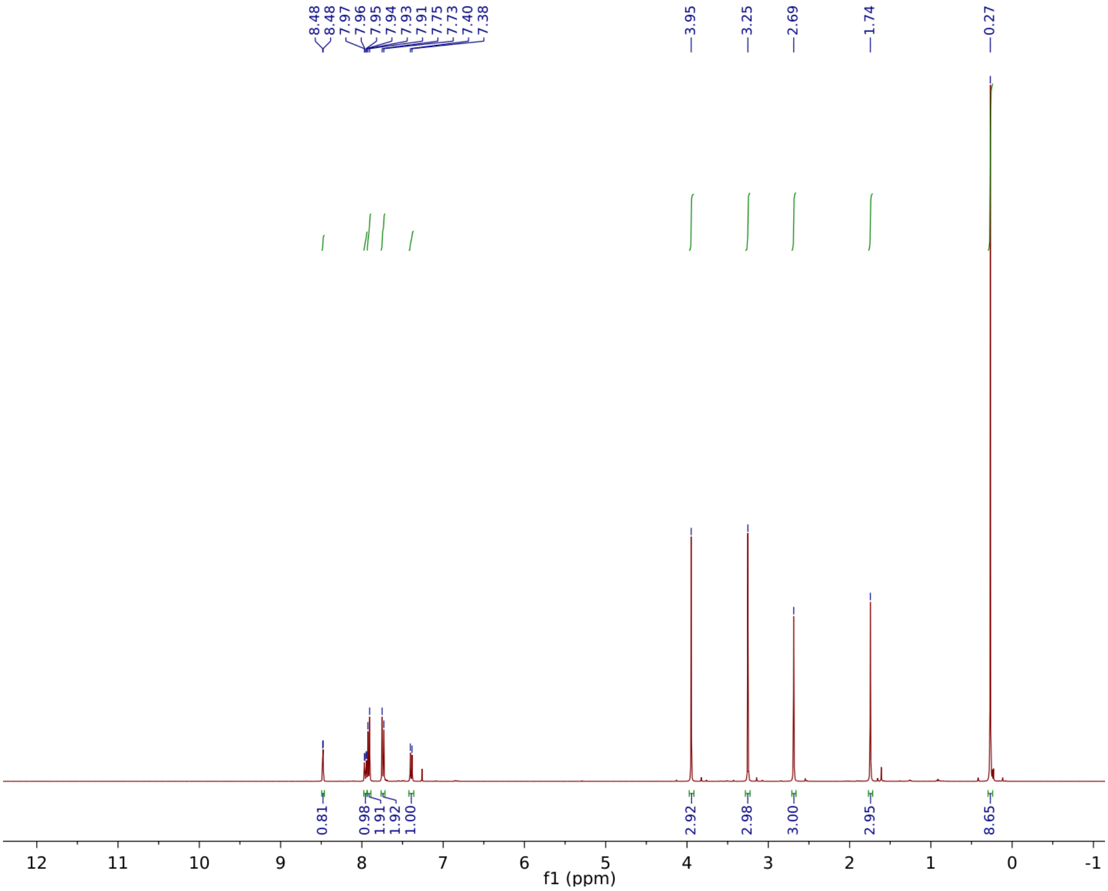

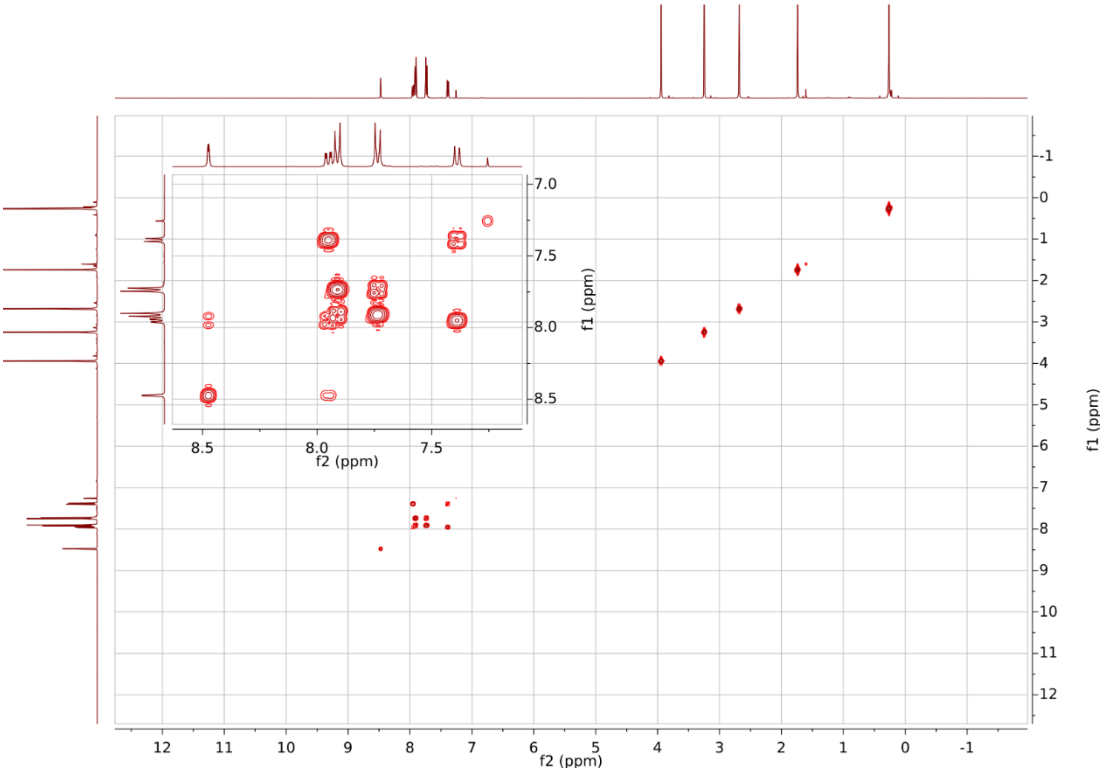

Compound S19a (^{13}C NMR, 101 MHz, $CDCl_3$)

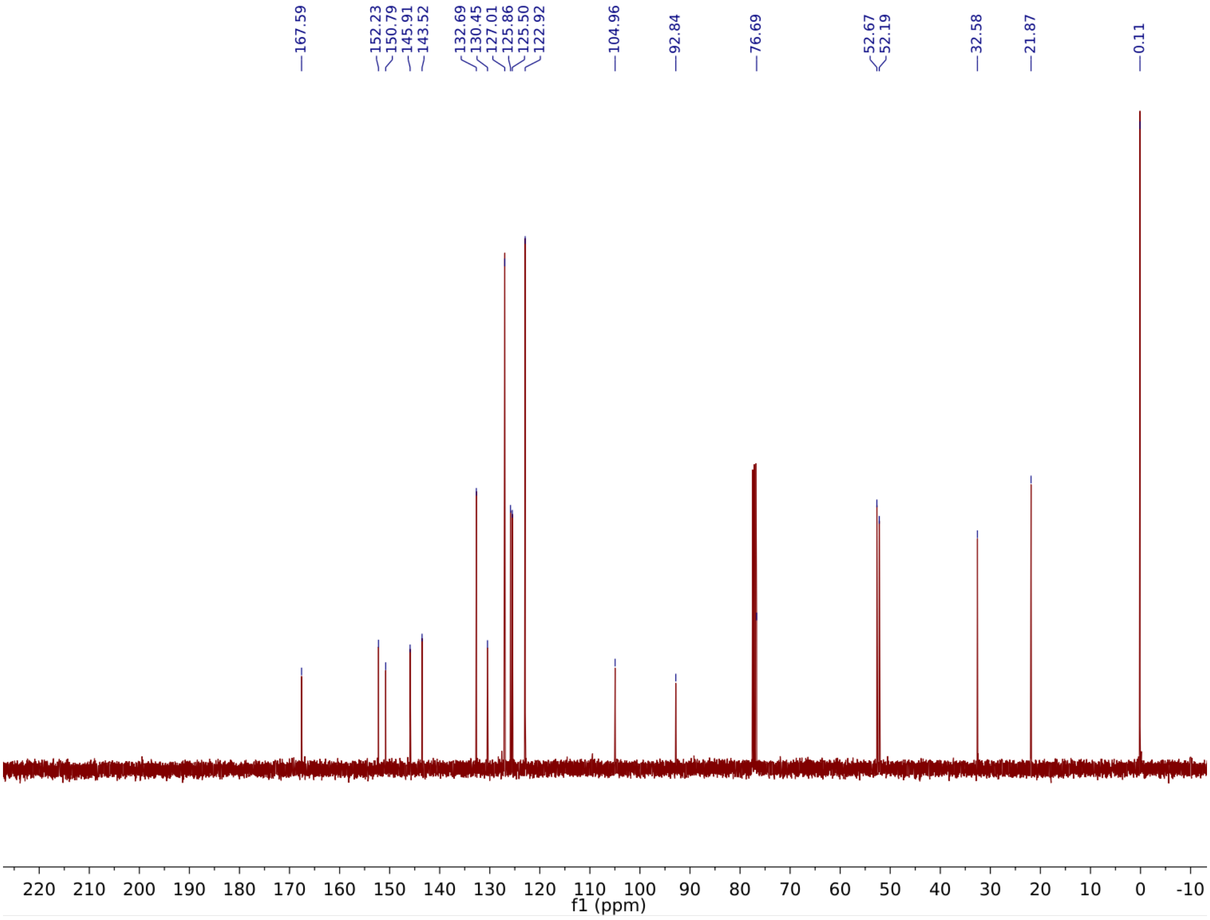

Compound S19b (^{1}H NMR, 400 MHz, $CDCl_3$)

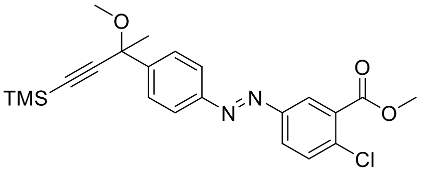

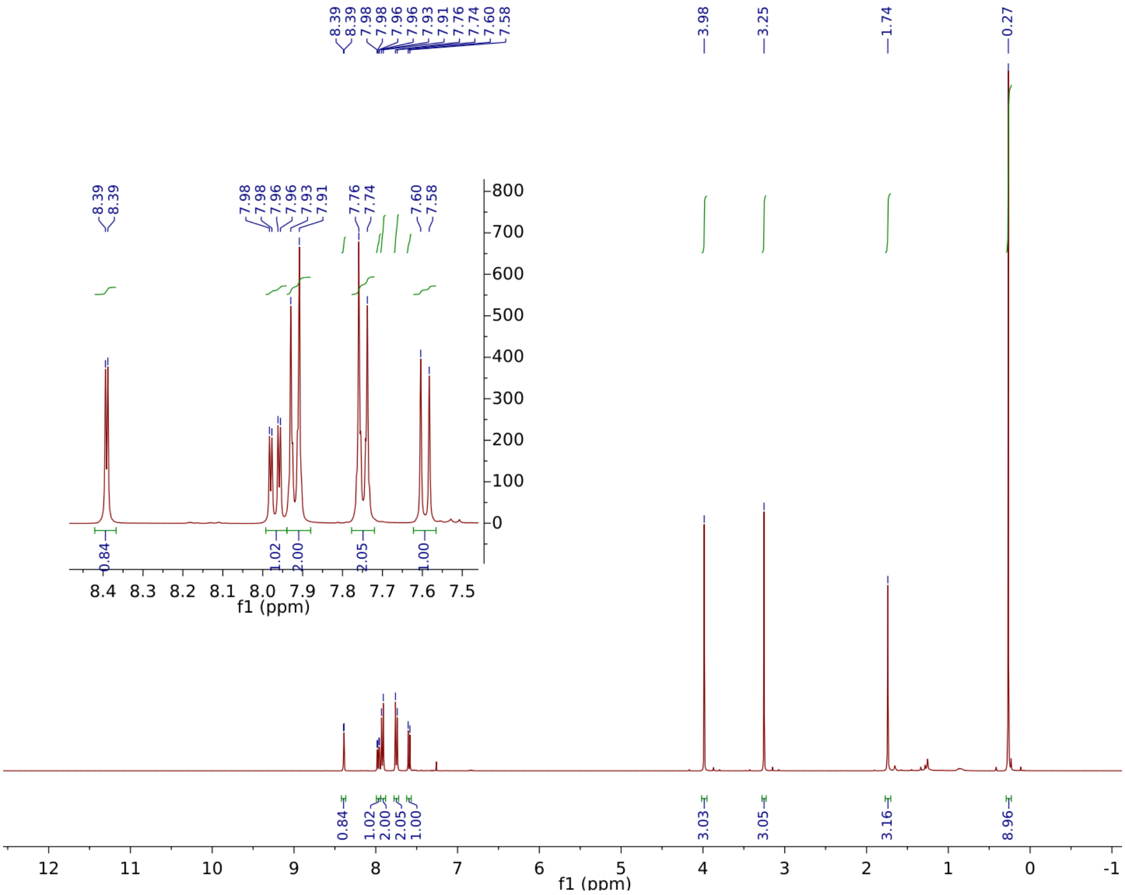

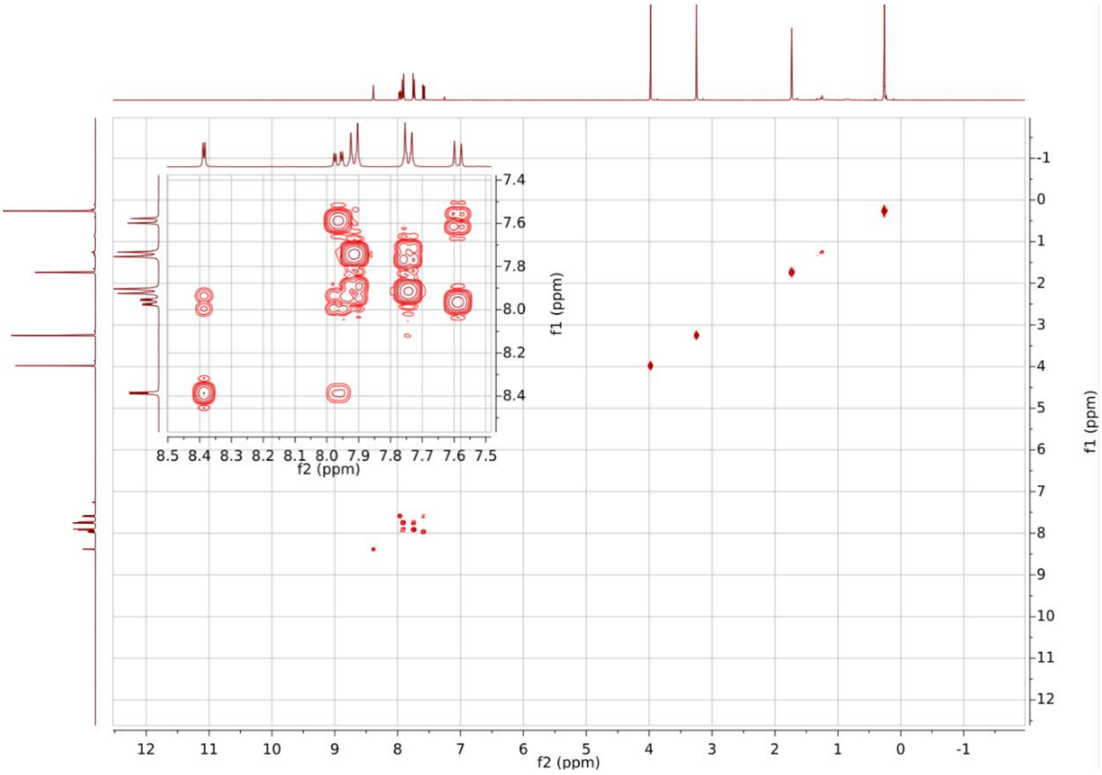

Compound S19b (^{13}C NMR, 101 MHz, $CDCl_3$)

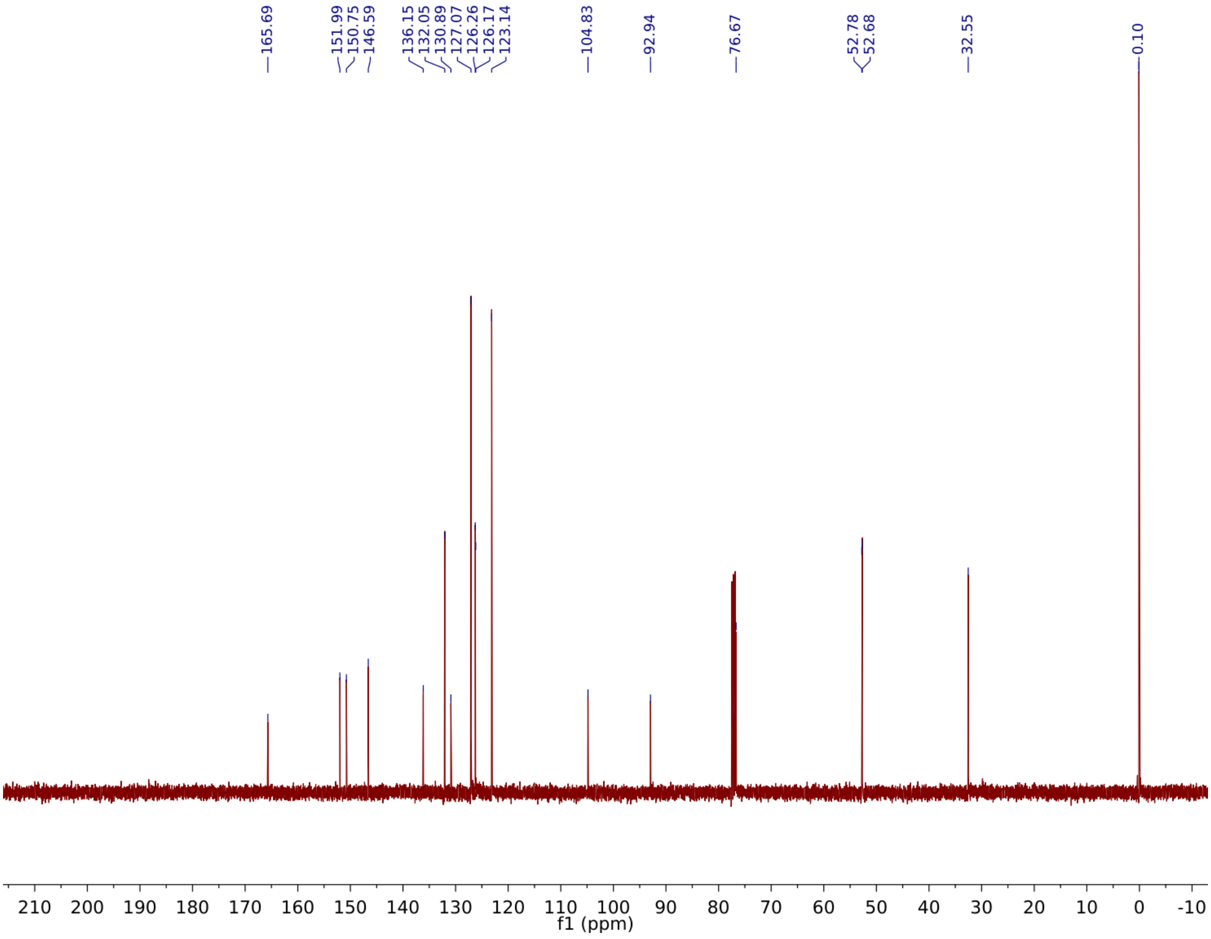

Compound S19c (^1H NMR, 400 MHz, $CDCl_3$)

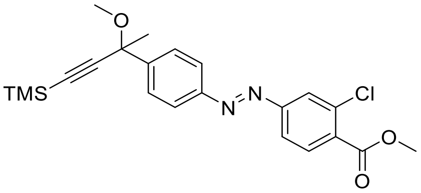

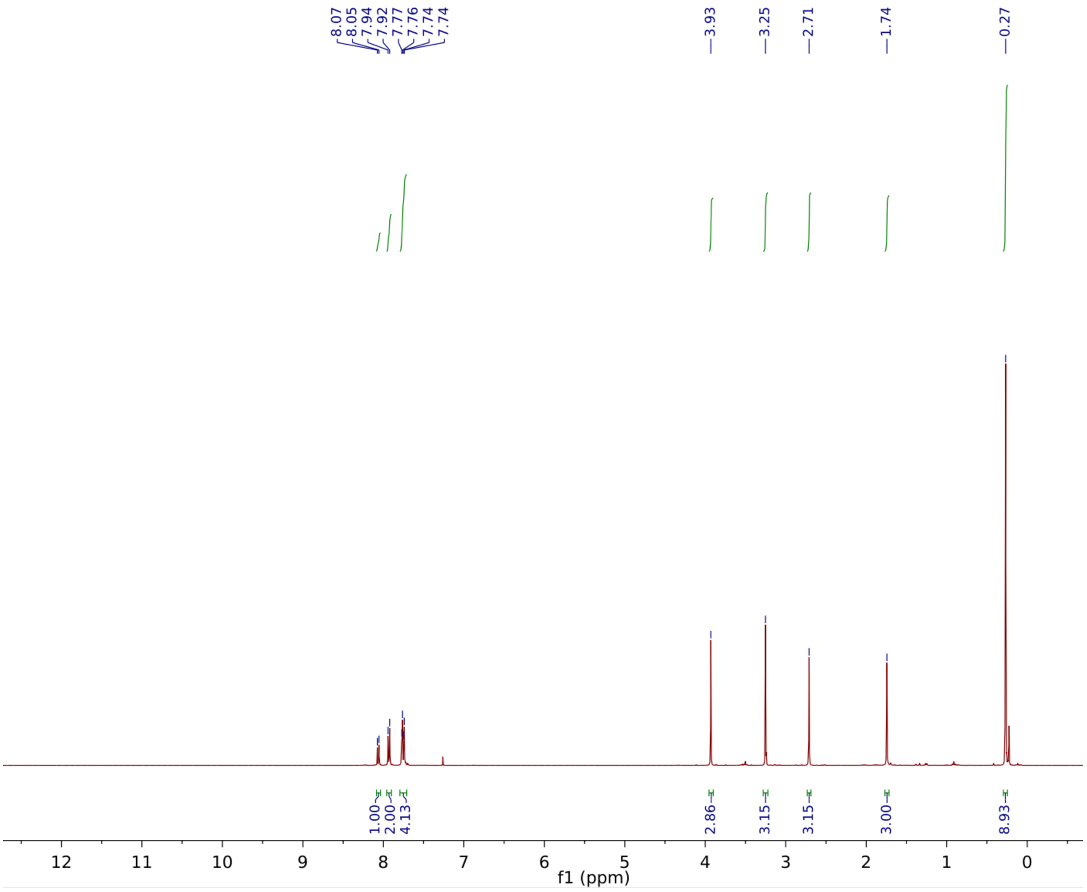

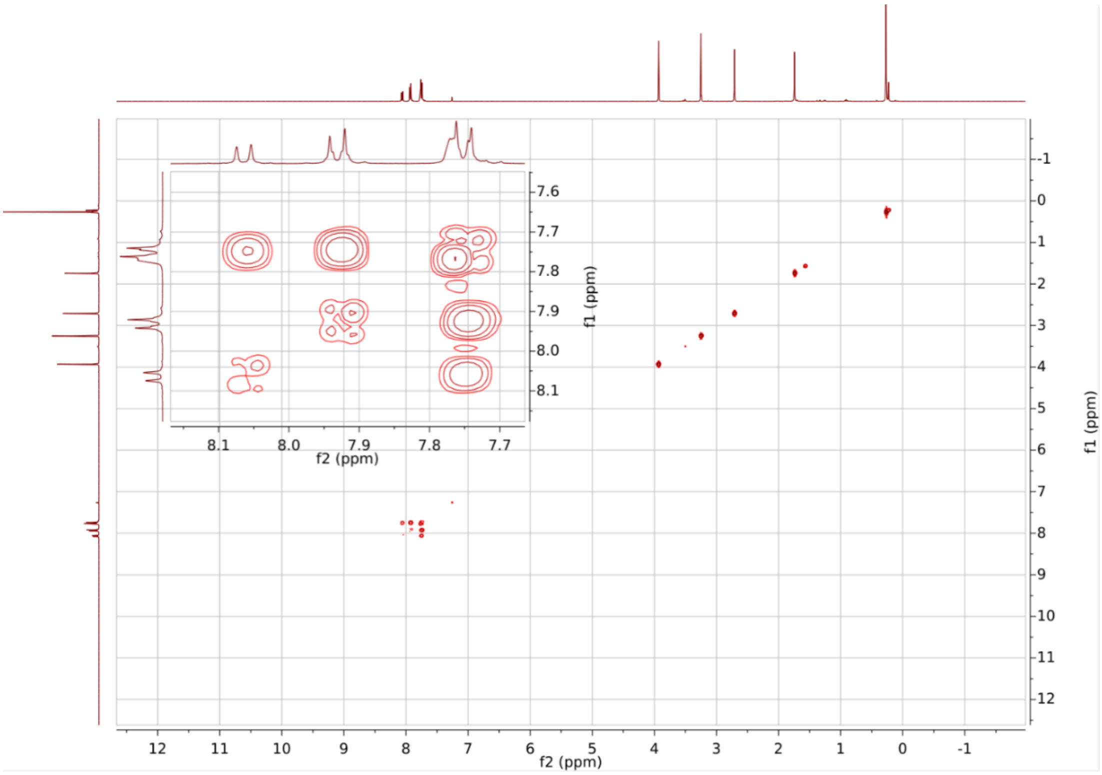

Compound S19c (^{13}C NMR, 101 MHz, $CDCl_3$)

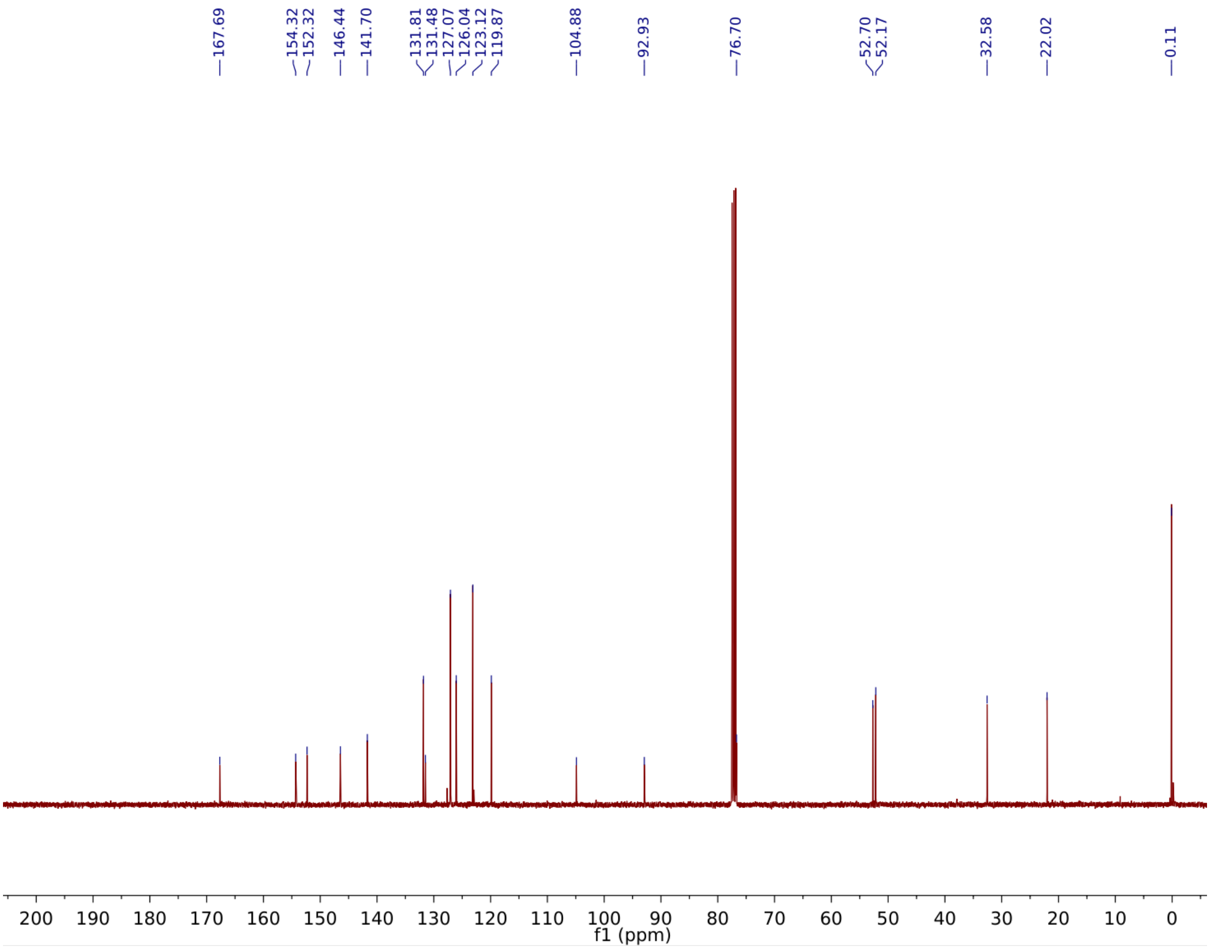

Compound S19d (^{1}H NMR, 400 MHz, $CDCl_3$)

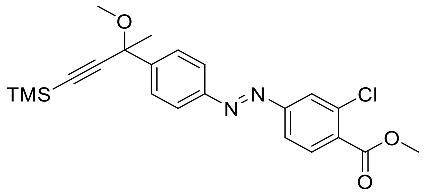

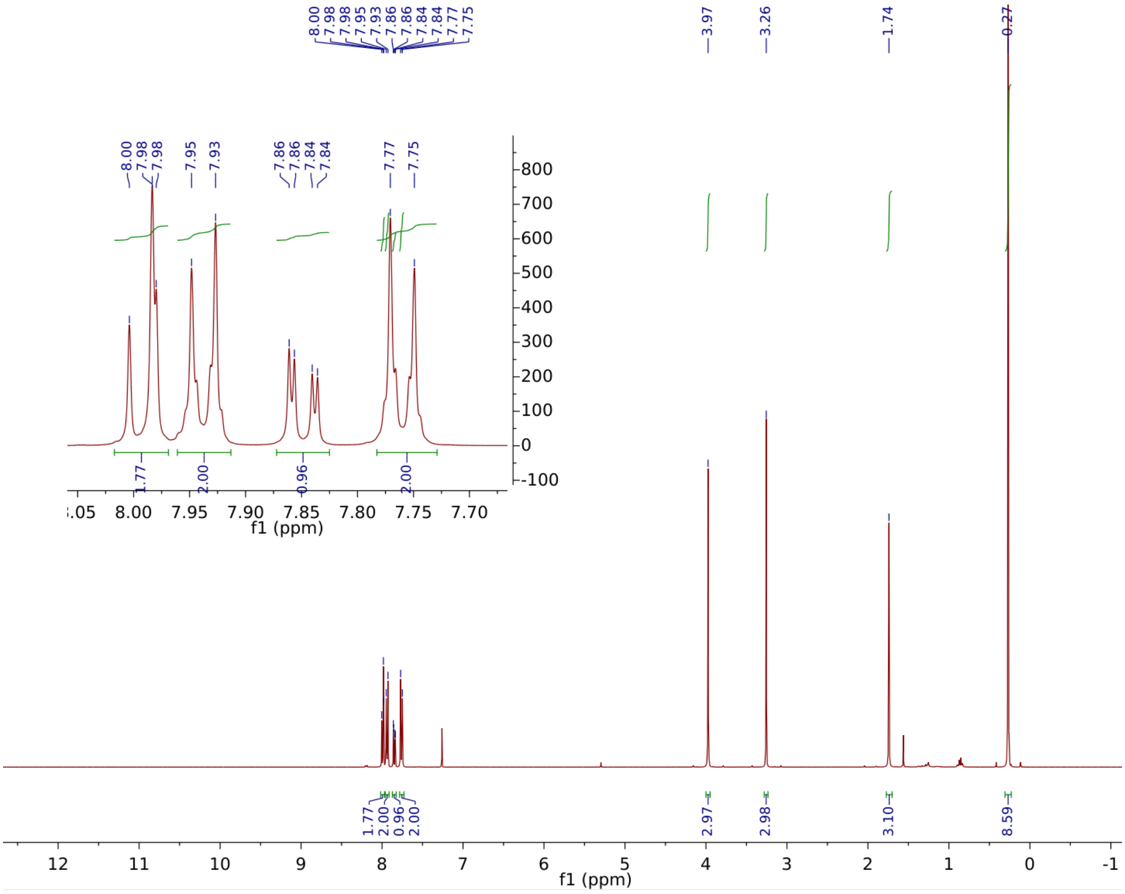

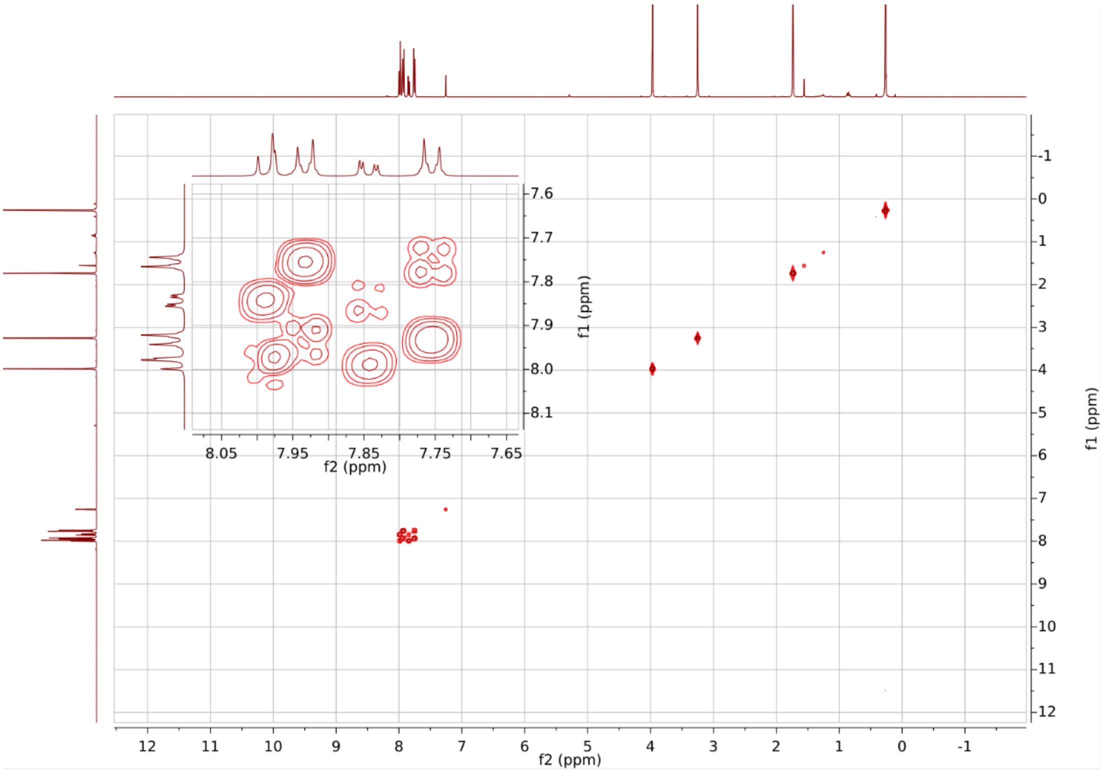

Compound S19d (^{13}C NMR, 101 MHz, $CDCl_3$)

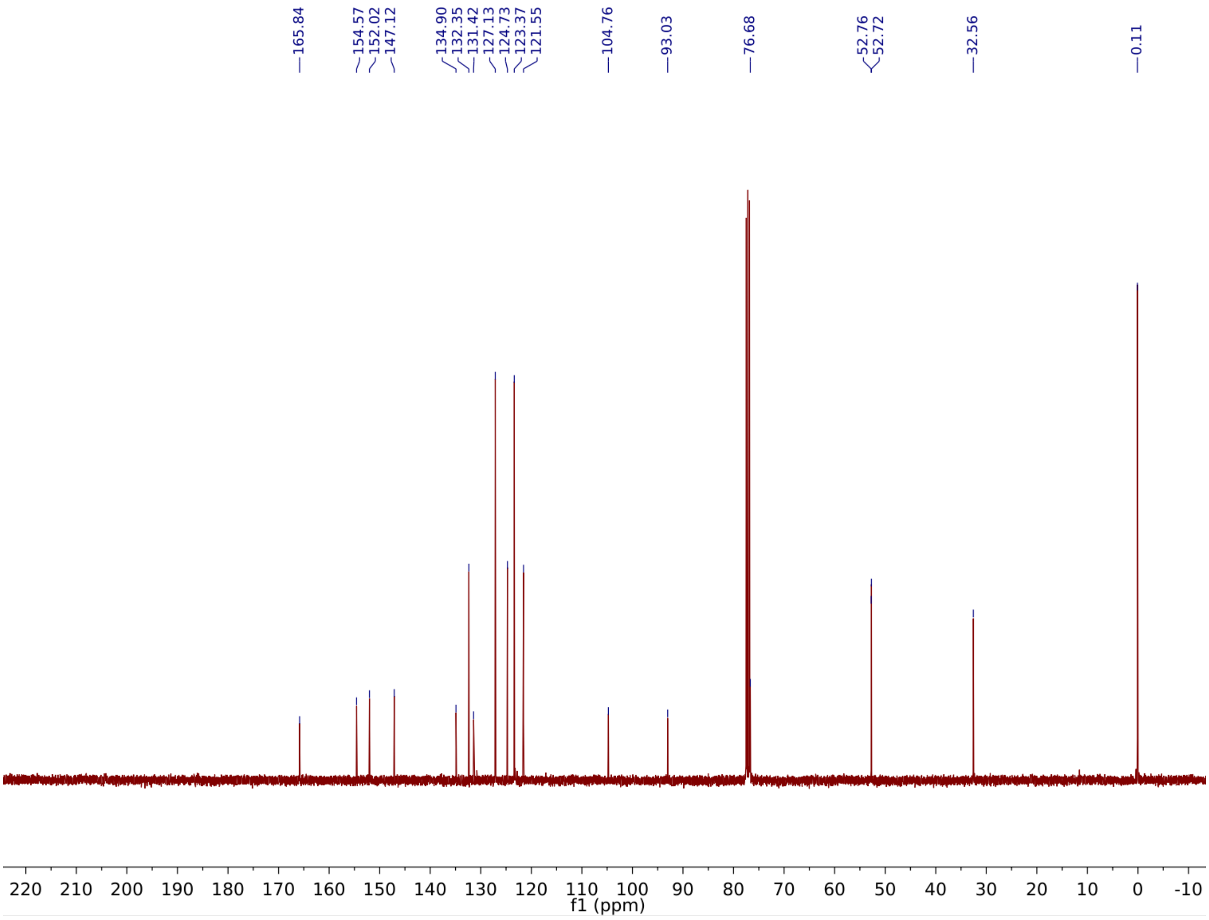

Compound S20a (^{1}H NMR, 400 MHz, $CDCl_3$)

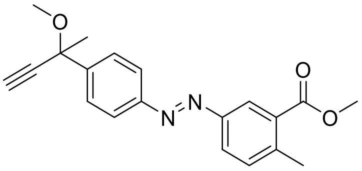

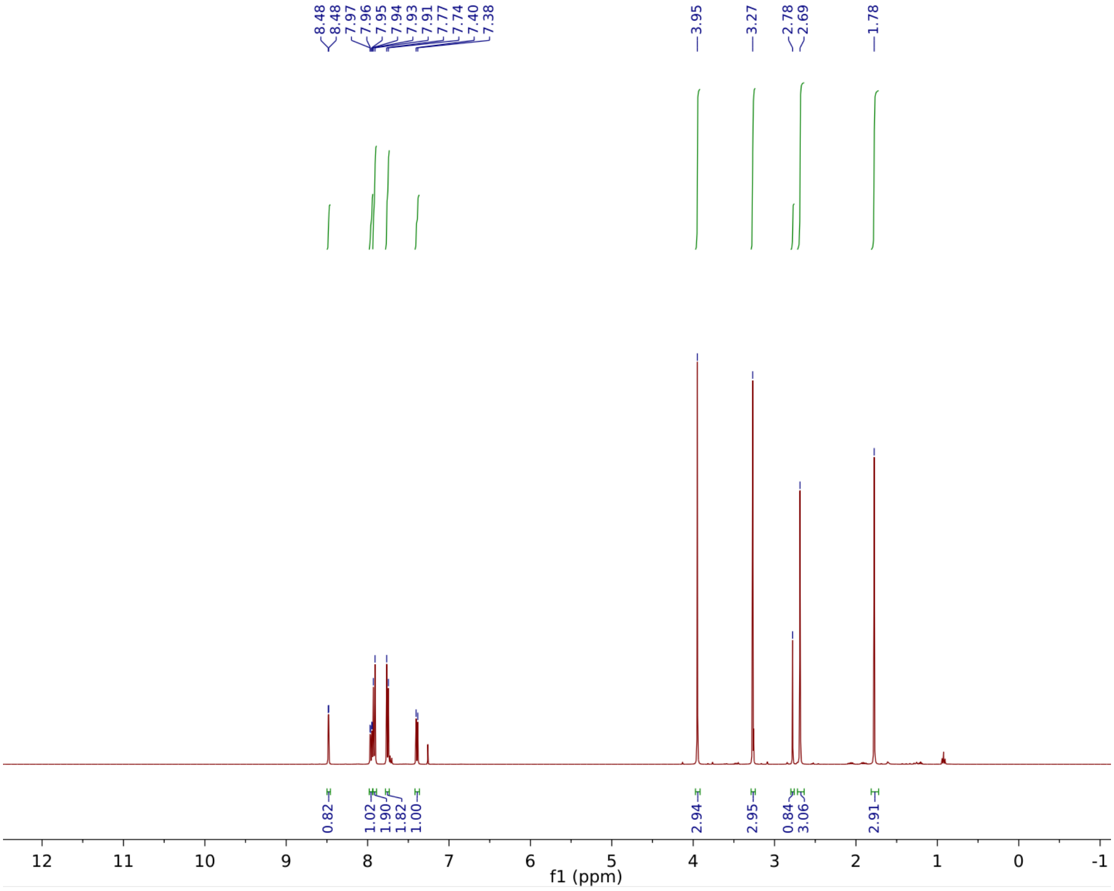

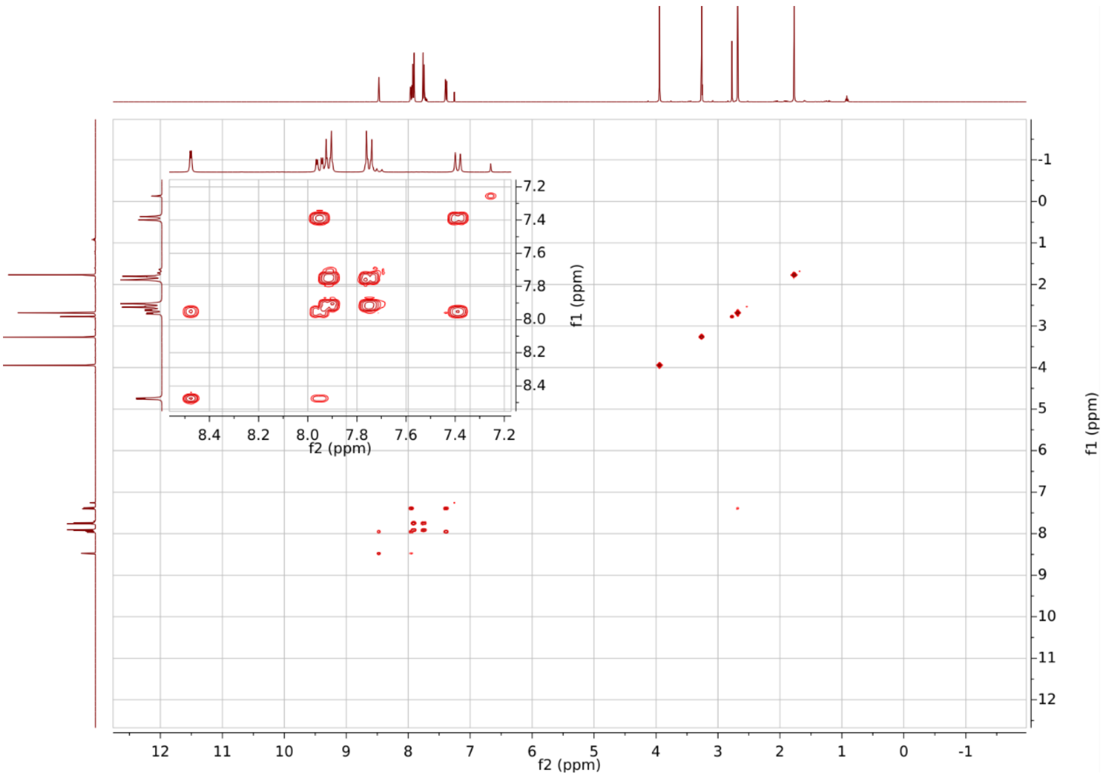

Compound S20a (^{13}C NMR, 101 MHz, $CDCl_3$)

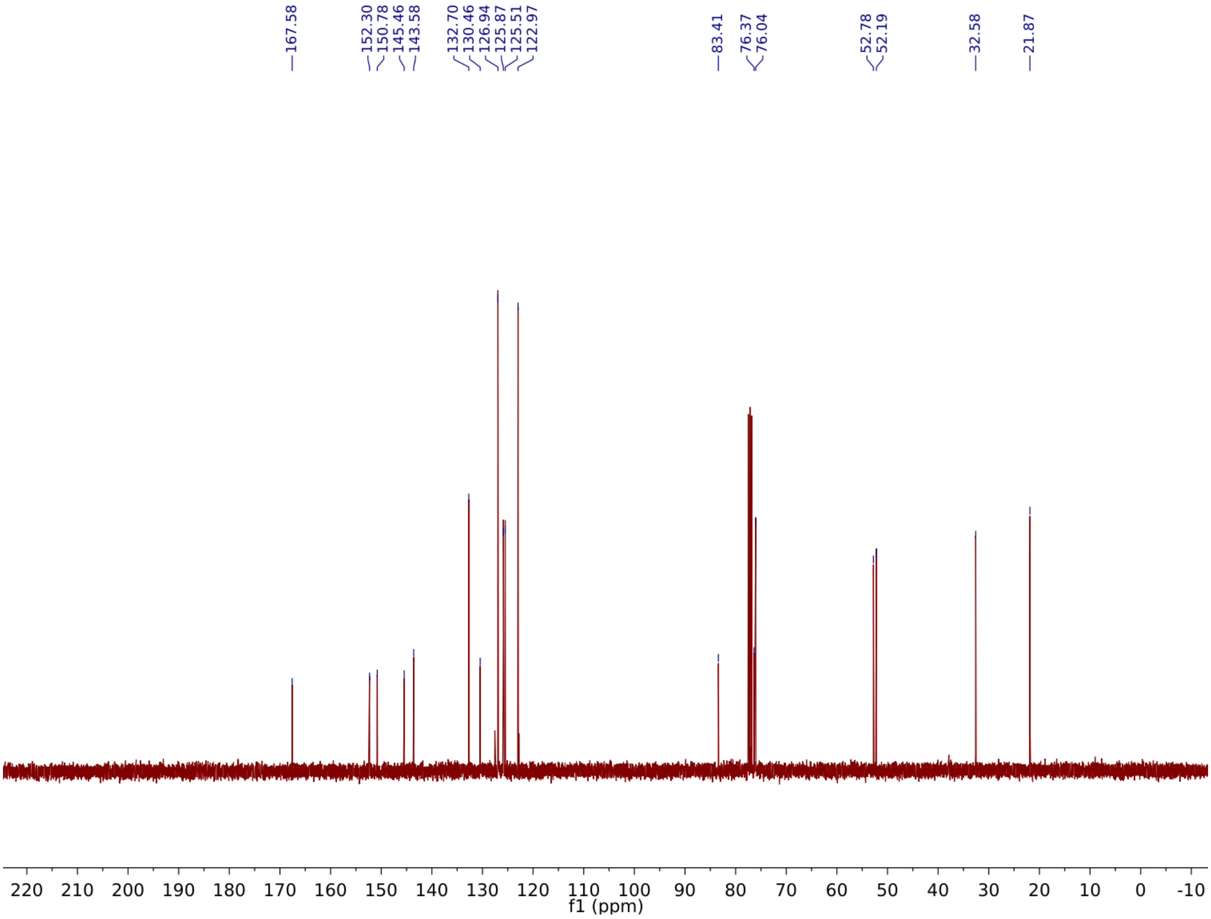

Compound S20b (^{1}H NMR, 400 MHz, $CDCl_3$)

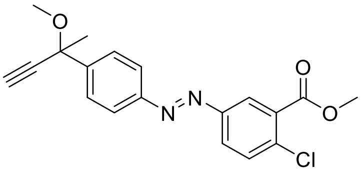

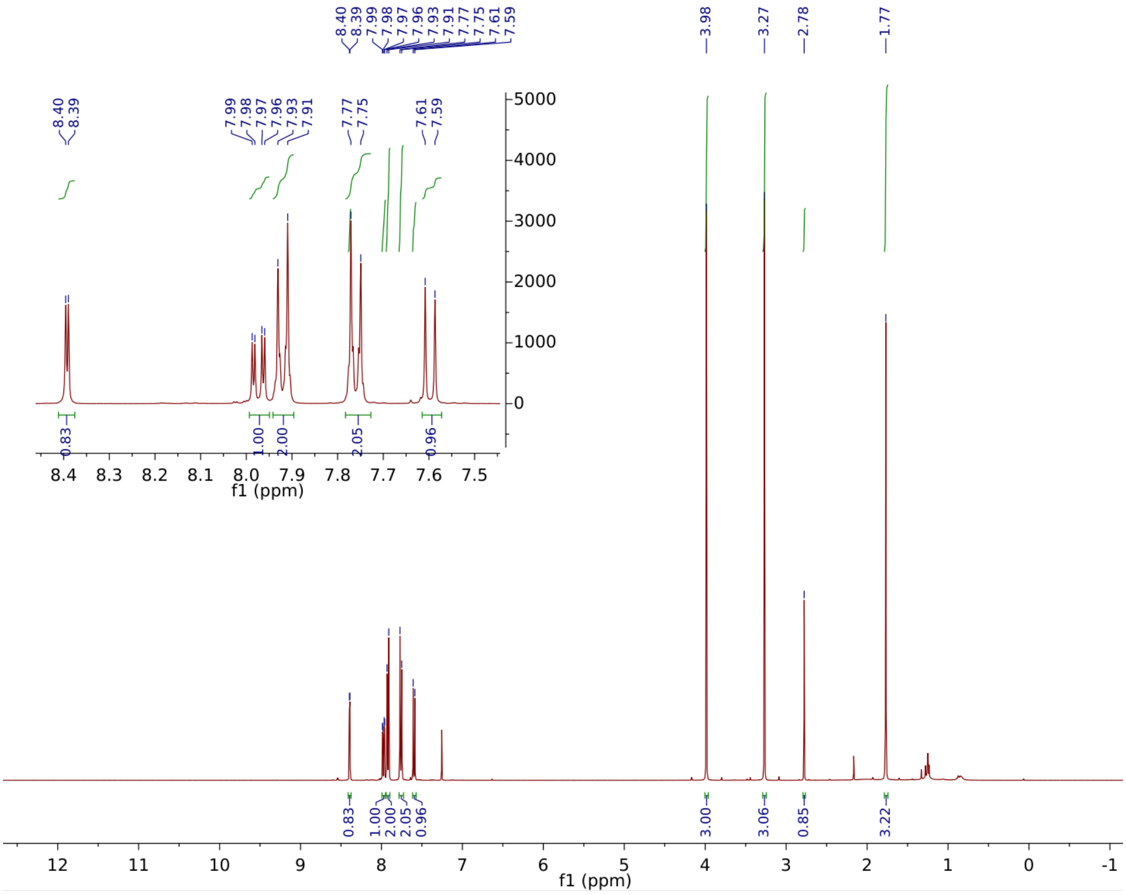

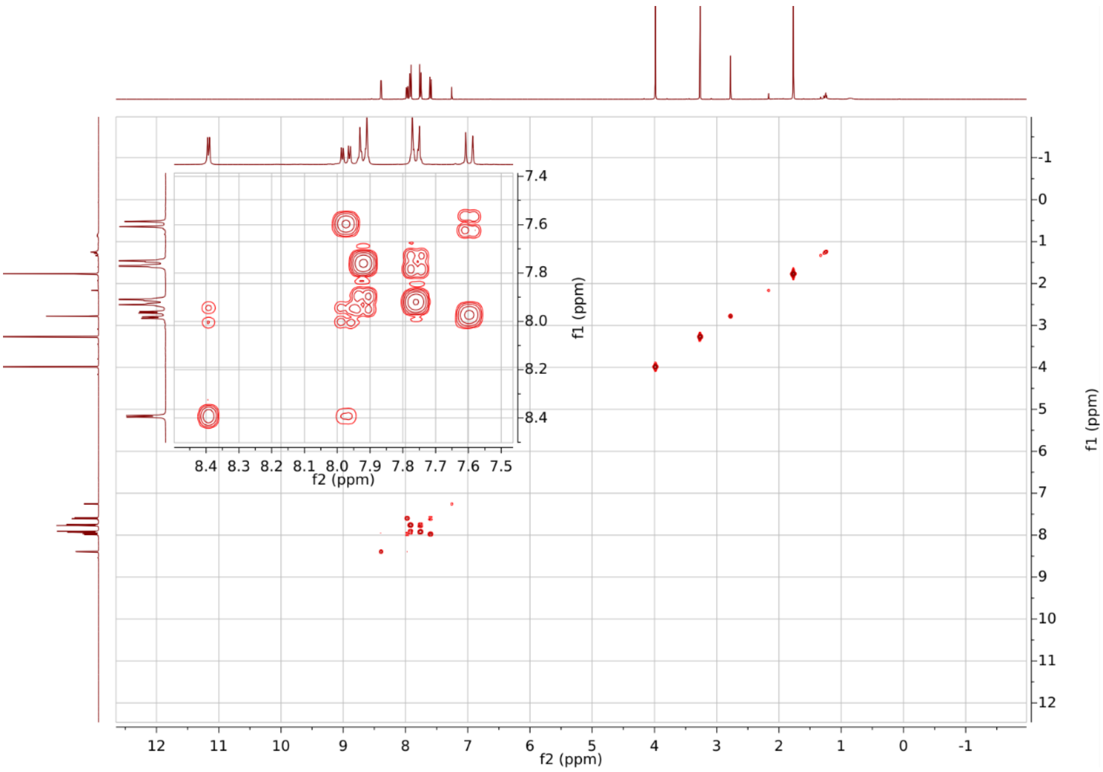

Compound S20b (^{13}C NMR, 101 MHz, $CDCl_3$)

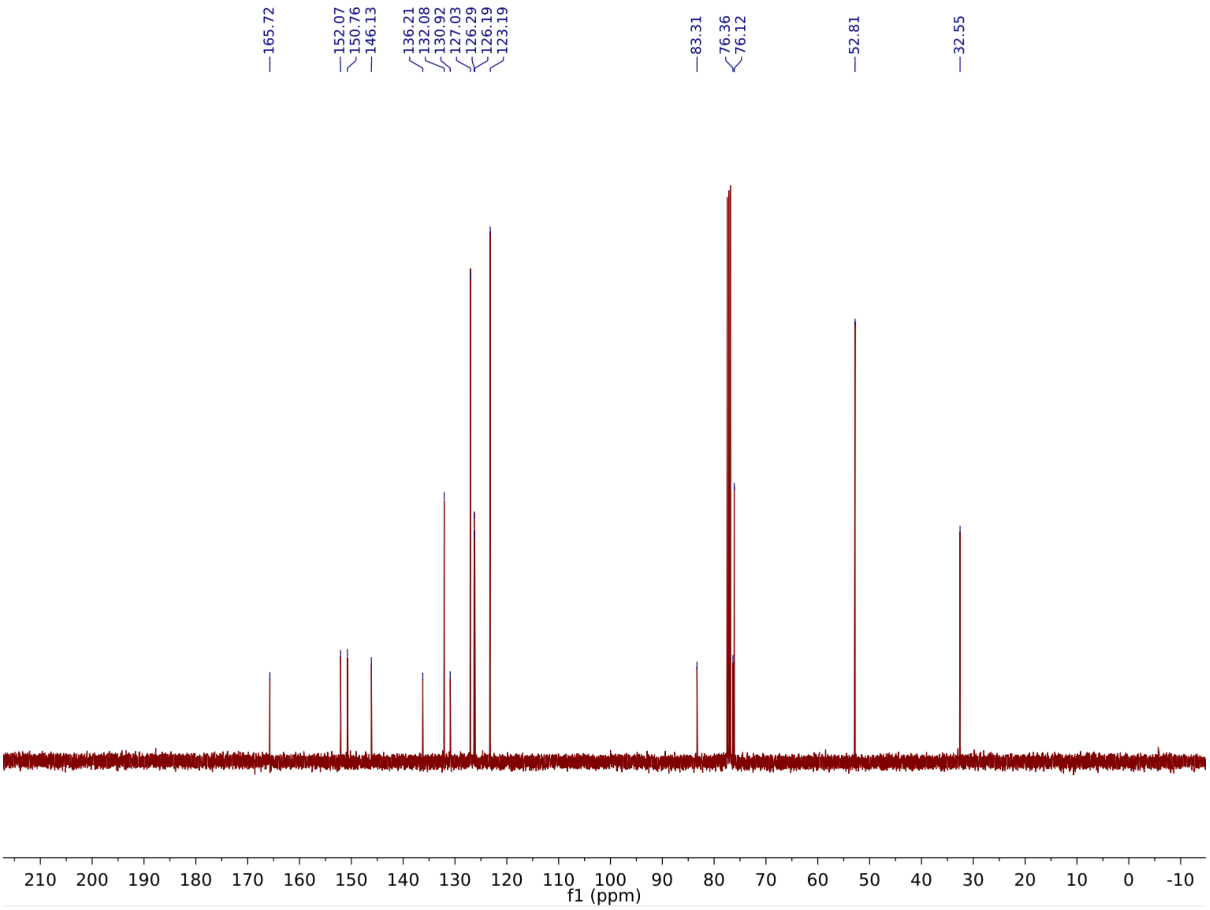

Compound S20c (^{1}H NMR, 400 MHz, $CDCl_3$)

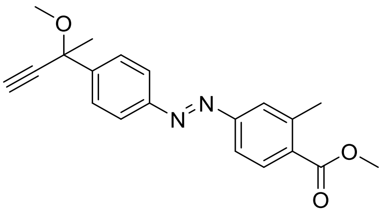

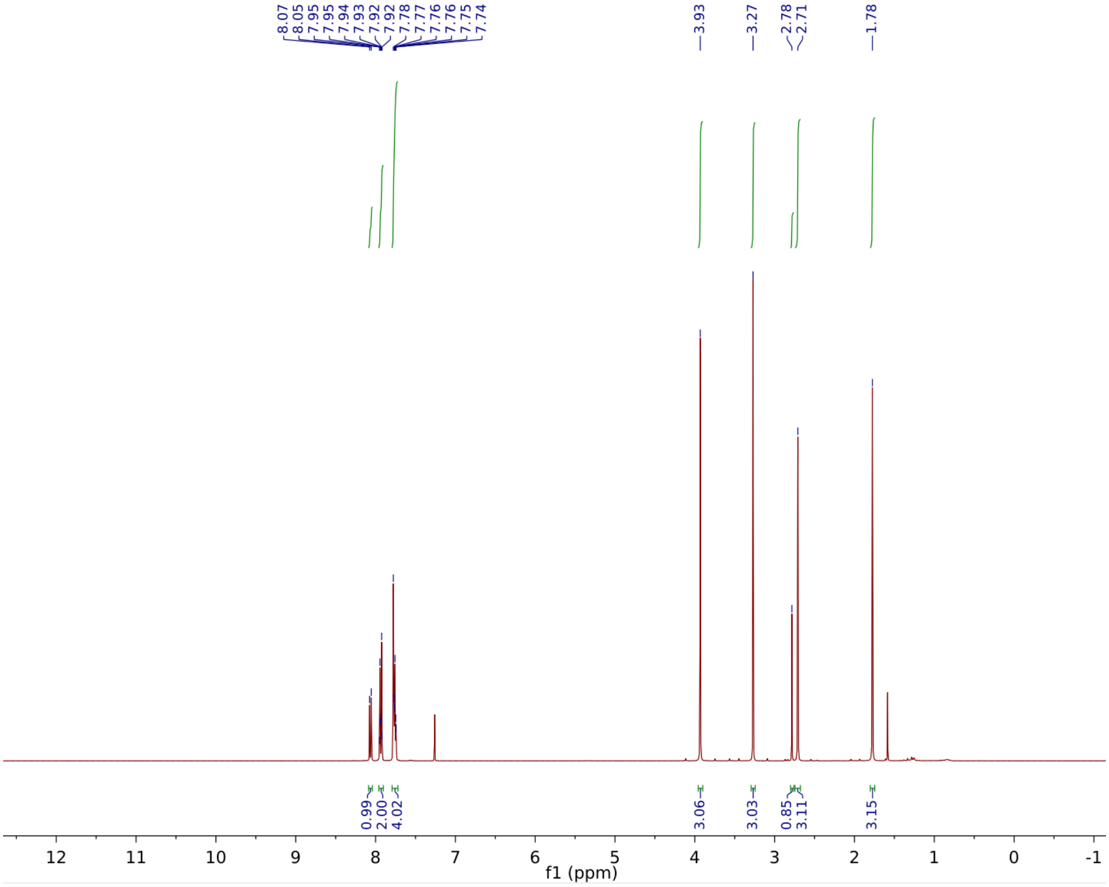

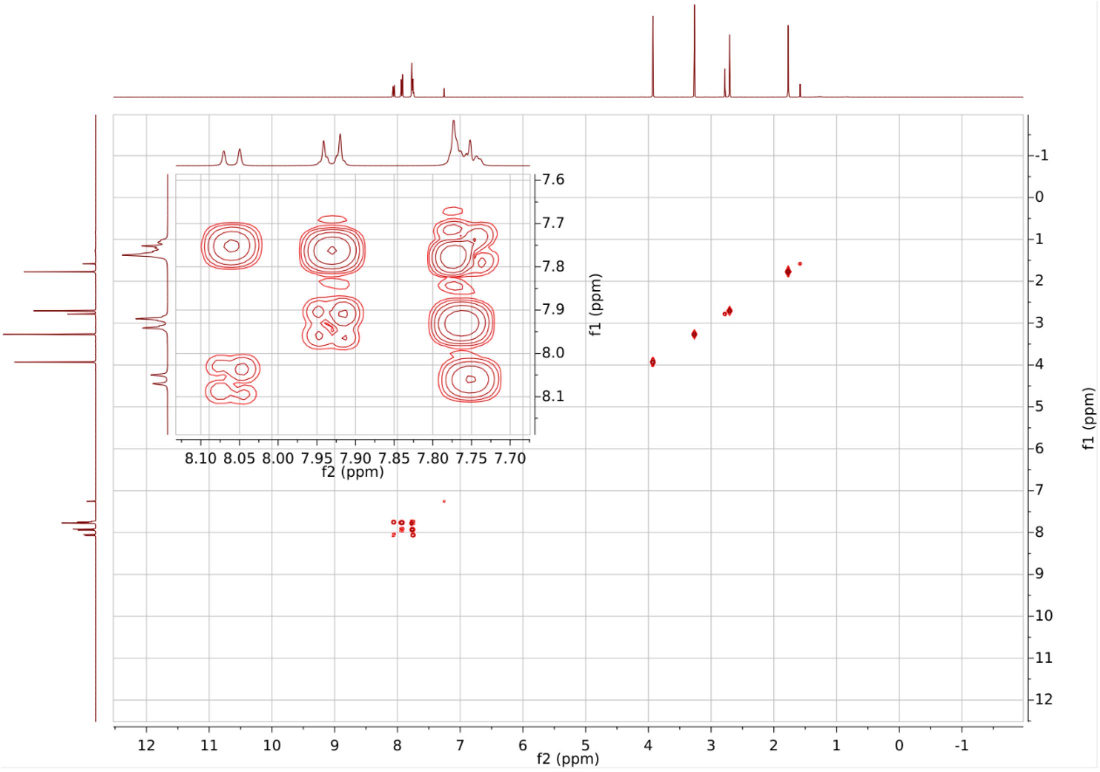

Compound S20c (^{13}C NMR, 101 MHz, $CDCl_3$)

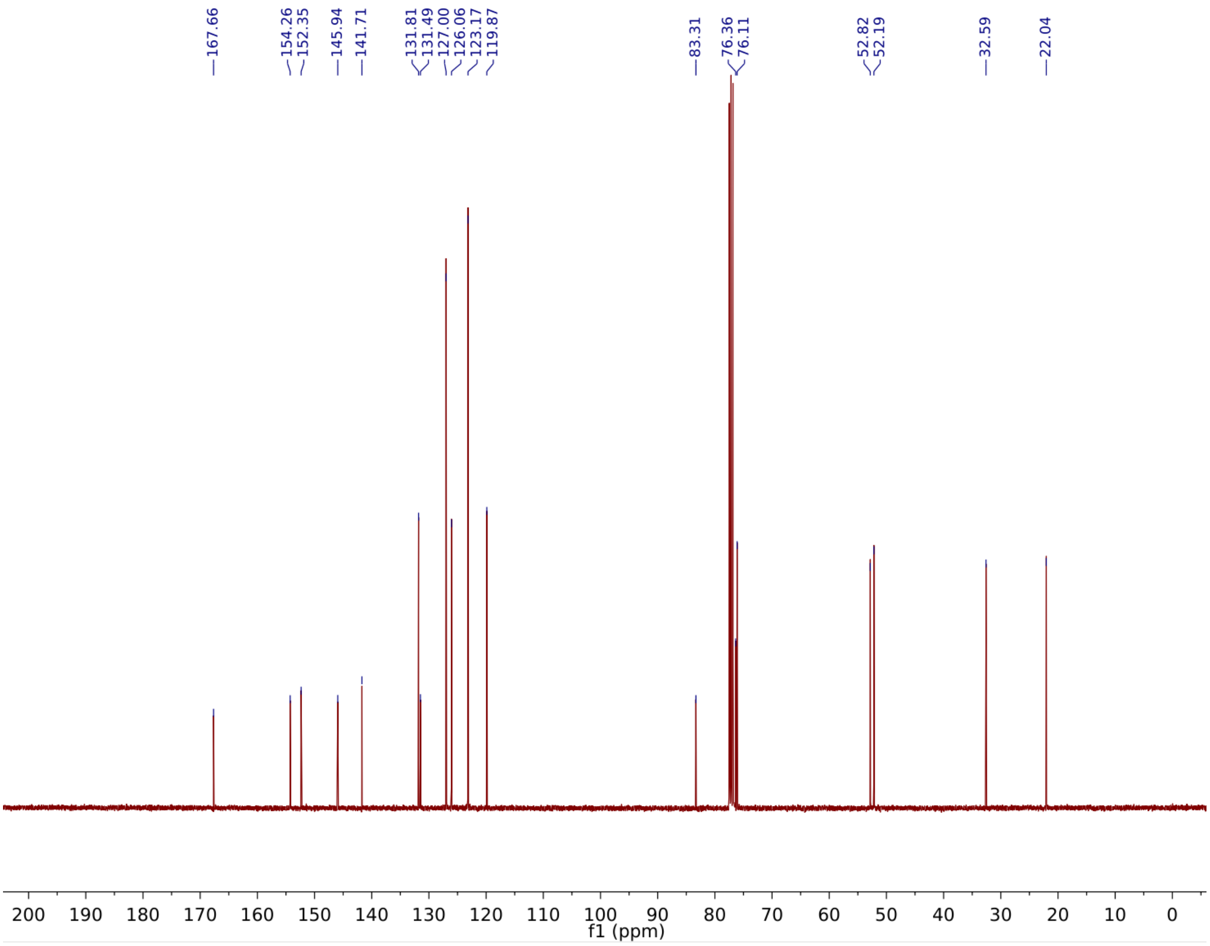

Compound S22 (^{1}H NMR, 400 MHz, $CDCl_3$)

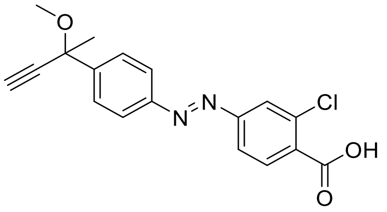

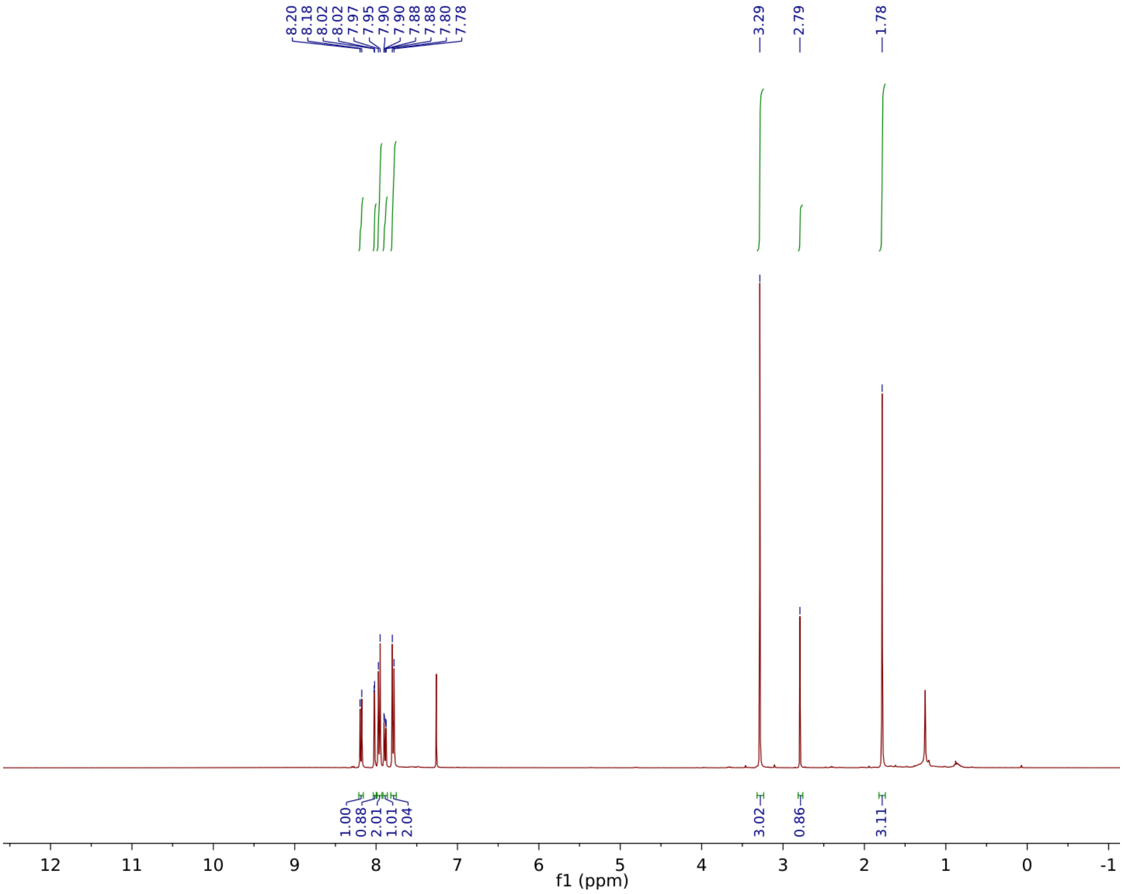

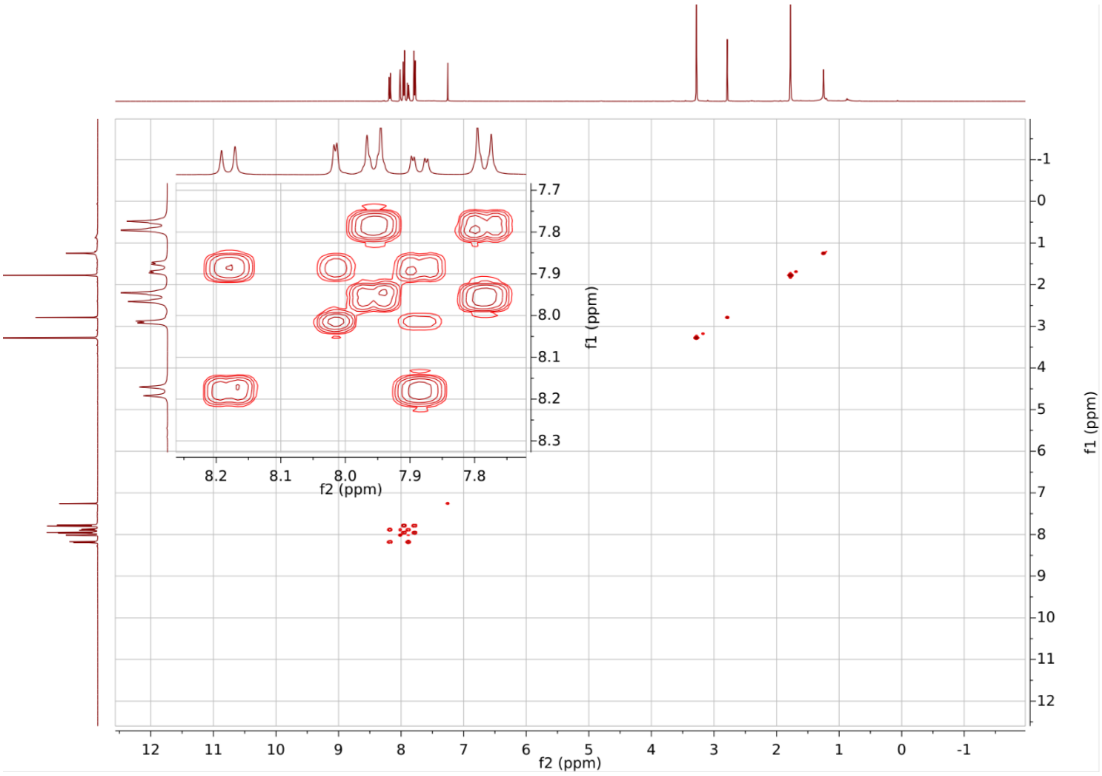

Compound S22 (^{13}C NMR, 101 MHz, $CDCl_3$)

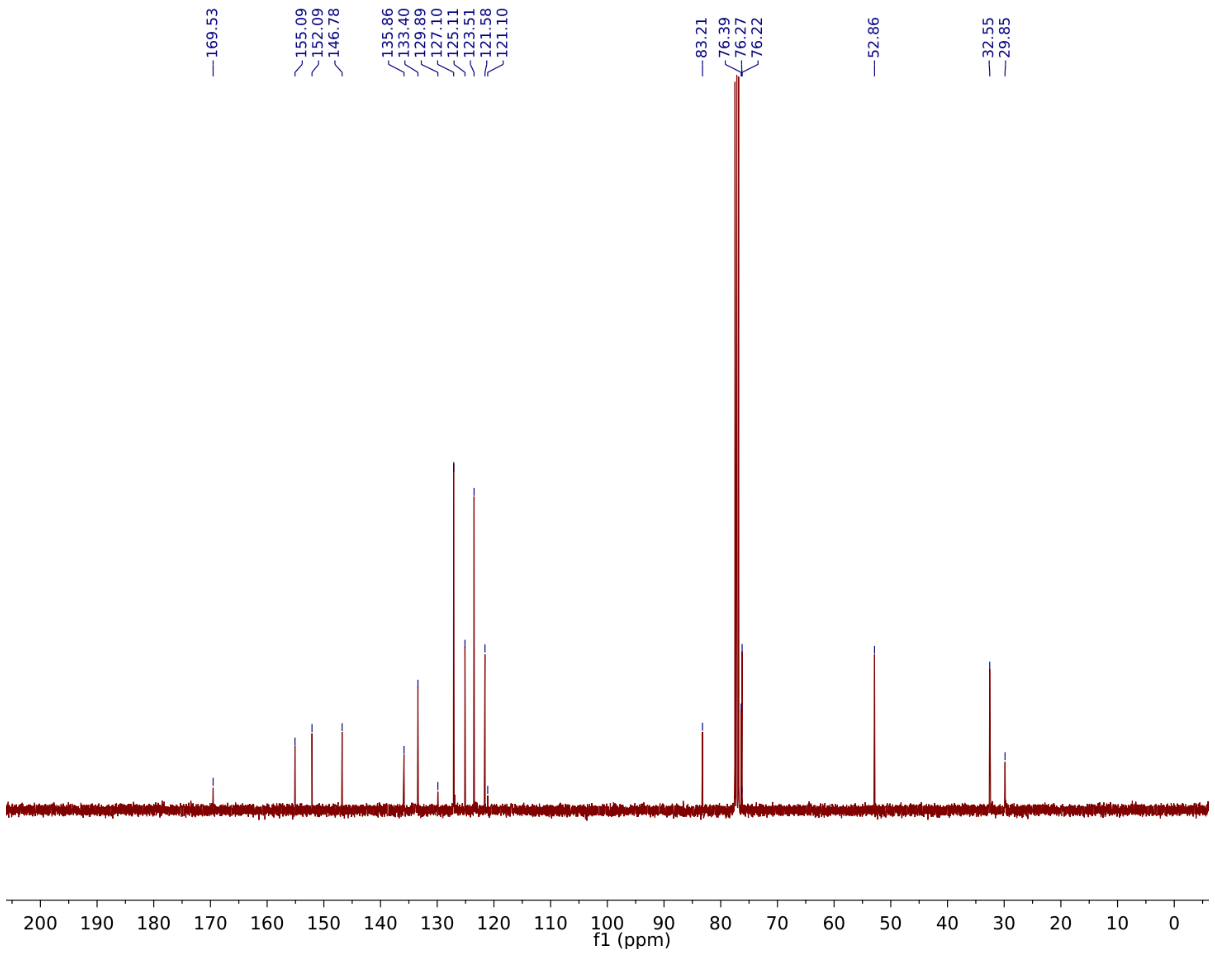

Compound S21a (^{1}H NMR, 400 MHz, DMSO-d_6)

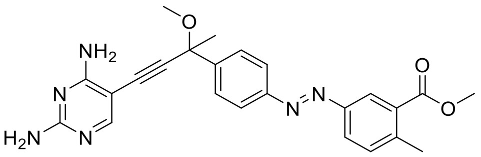

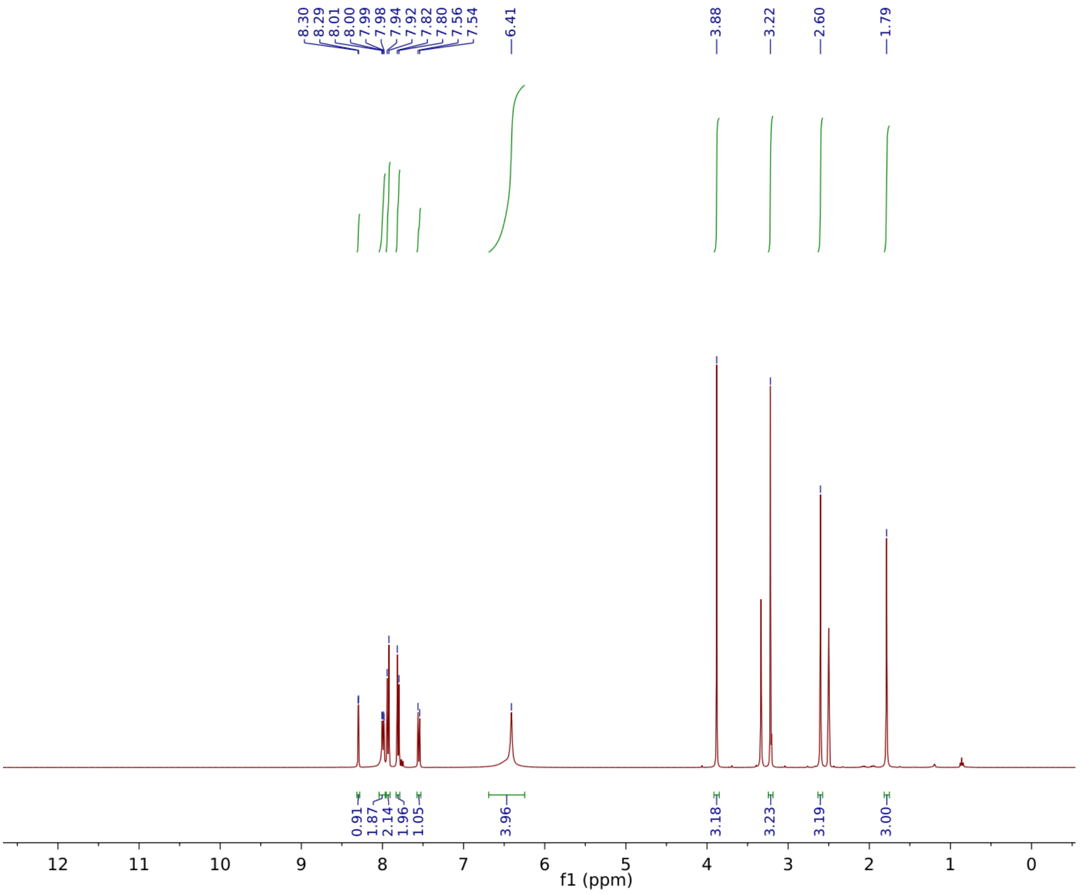

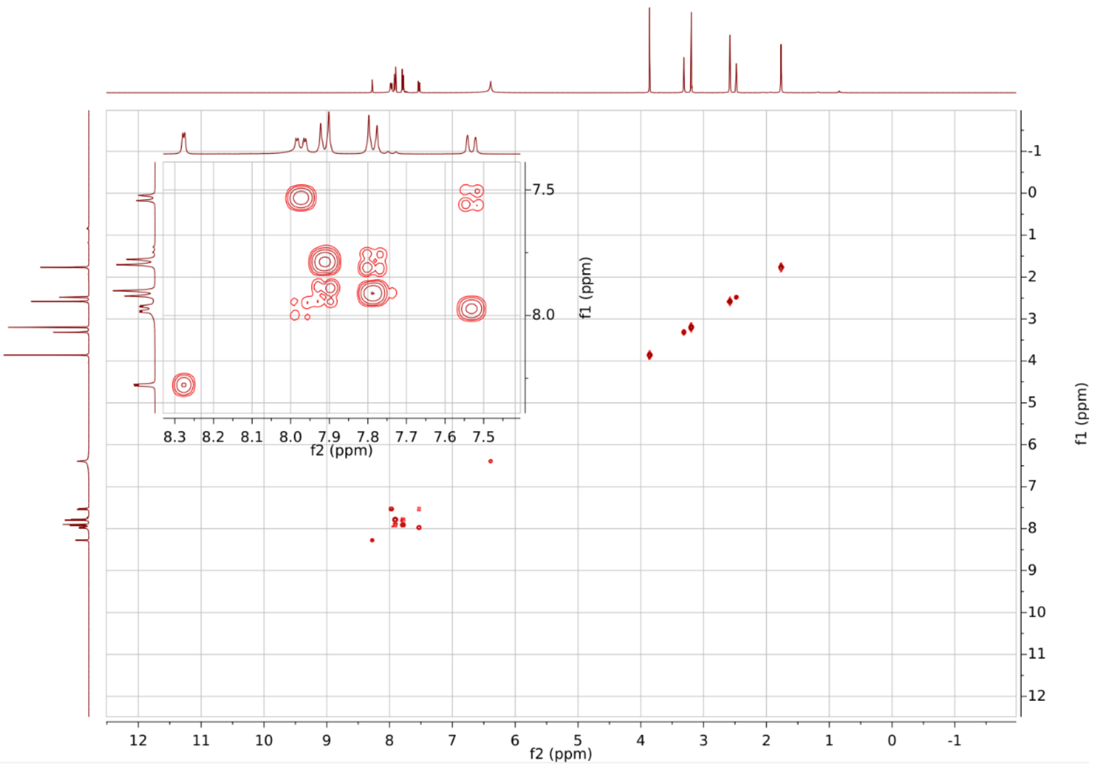

Compound S21a (^{13}C NMR, 101 MHz, DMSO-d_6)

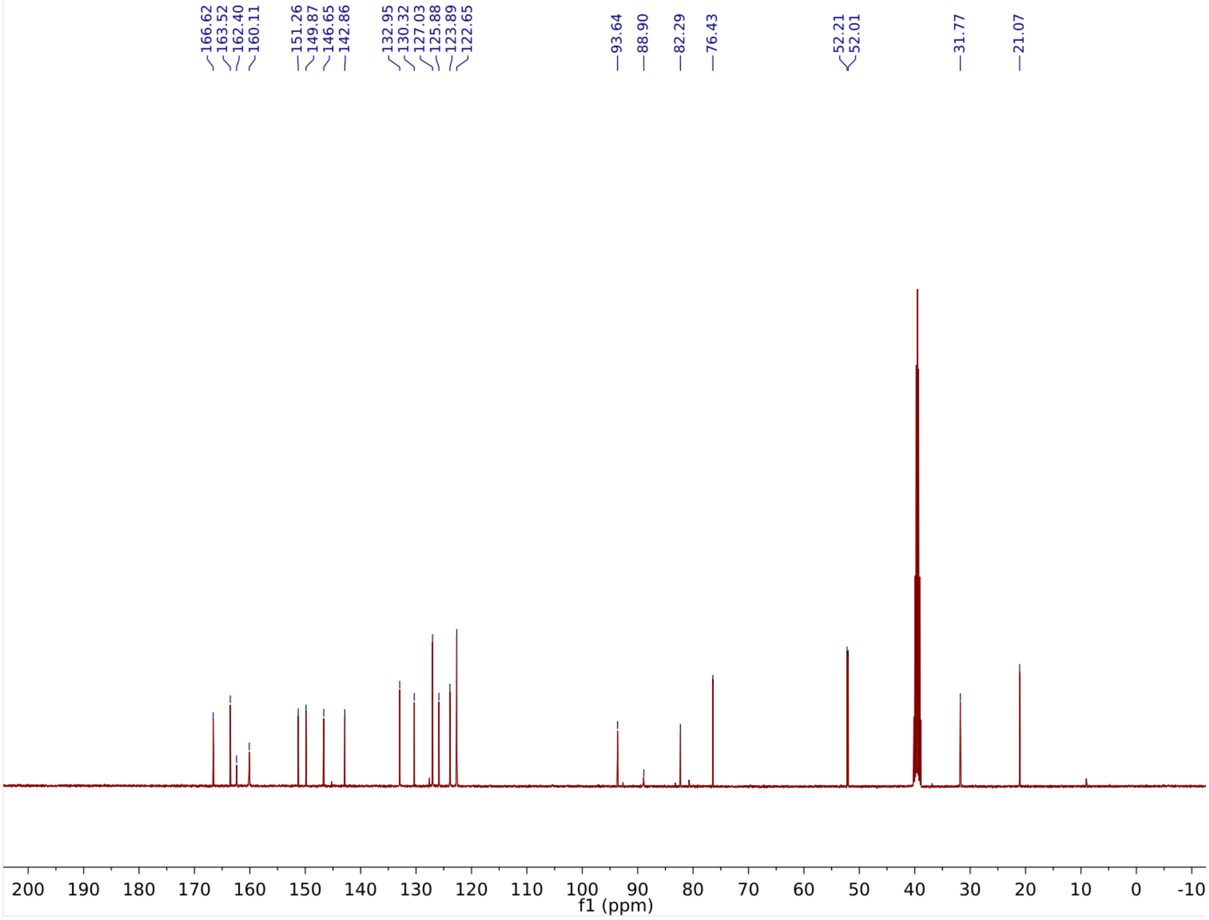

Compound S21b (^{1}H NMR, 400 MHz, $CDCl_3$)

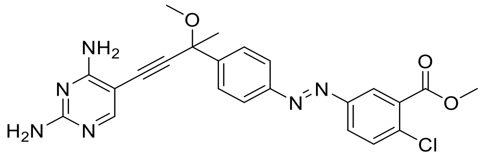

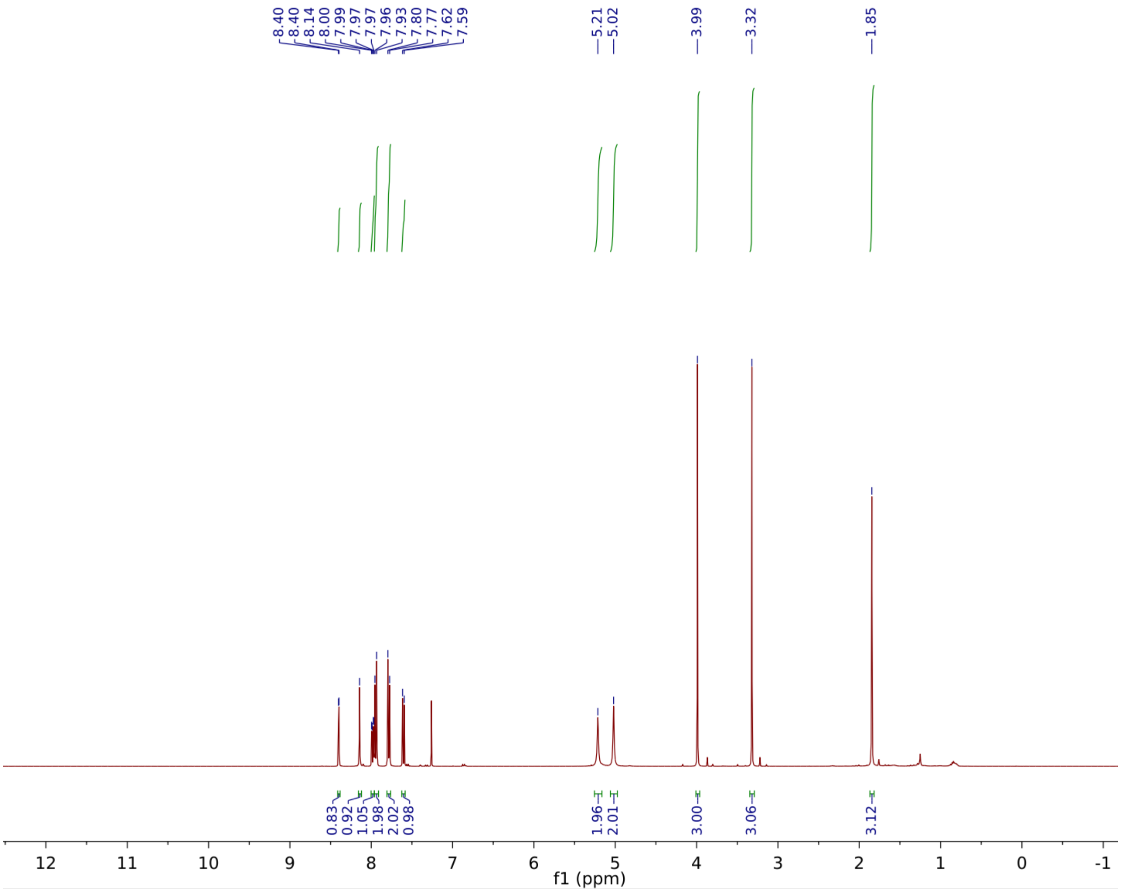

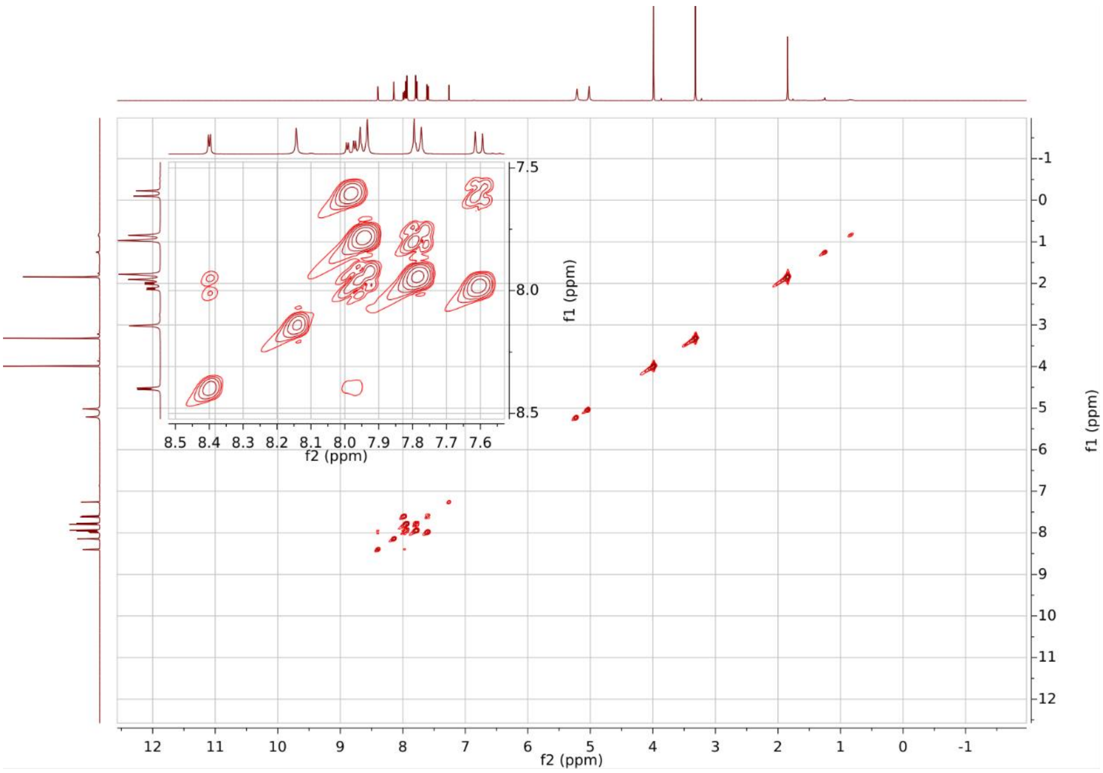

Compound S21b (^{13}C NMR, 101 MHz, $CDCl_3$)

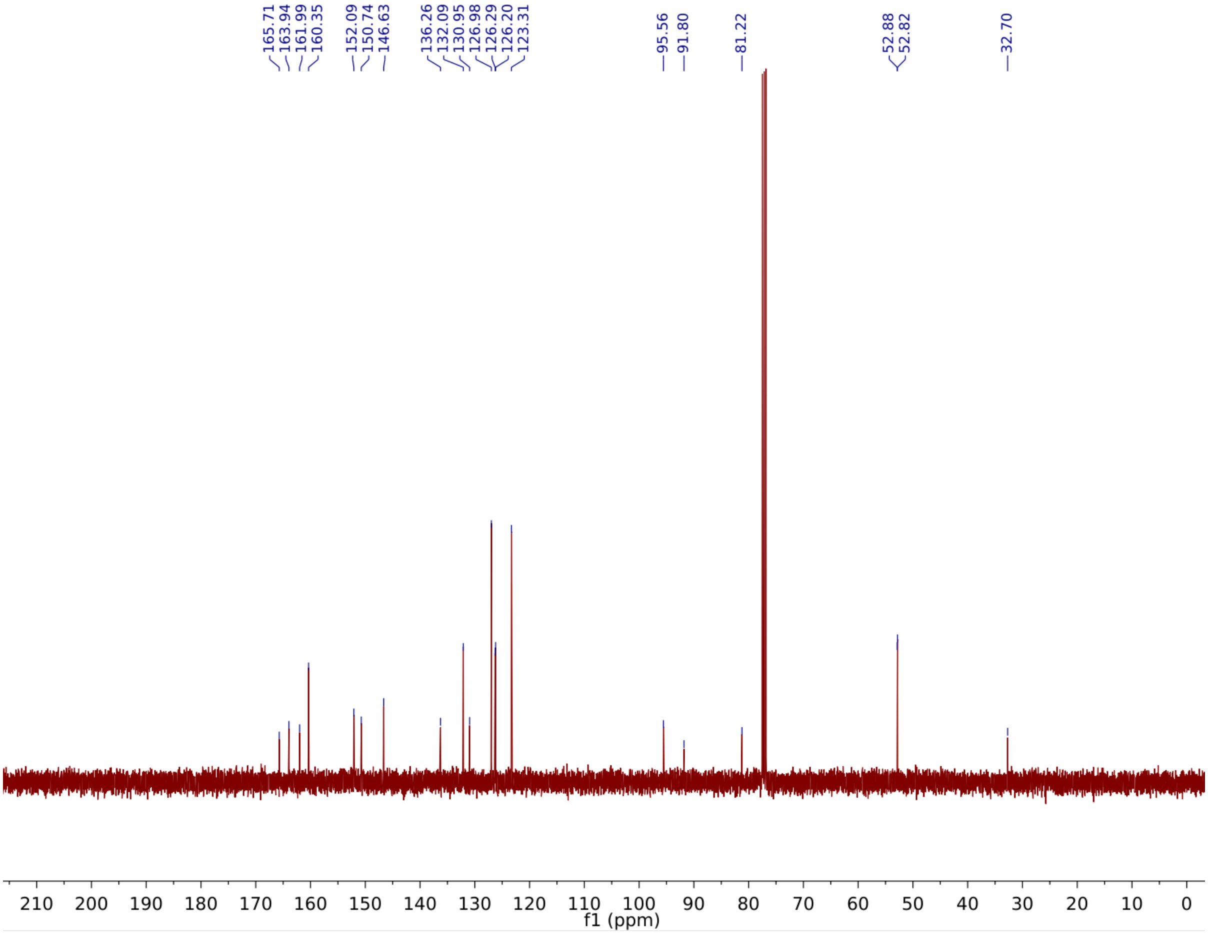

Compound S21c (^{1}H NMR, 400 MHz, $CDCl_3$)

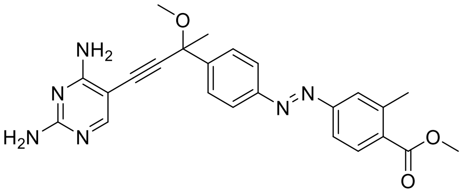

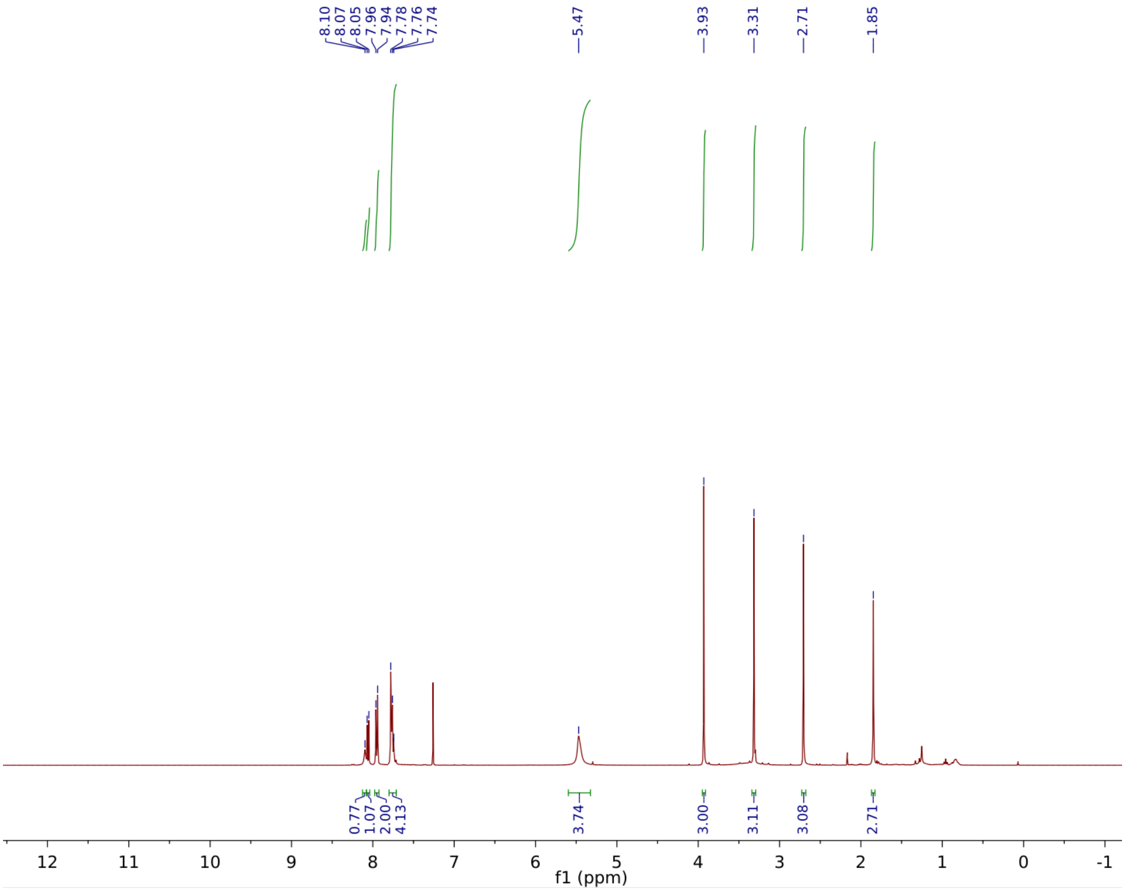

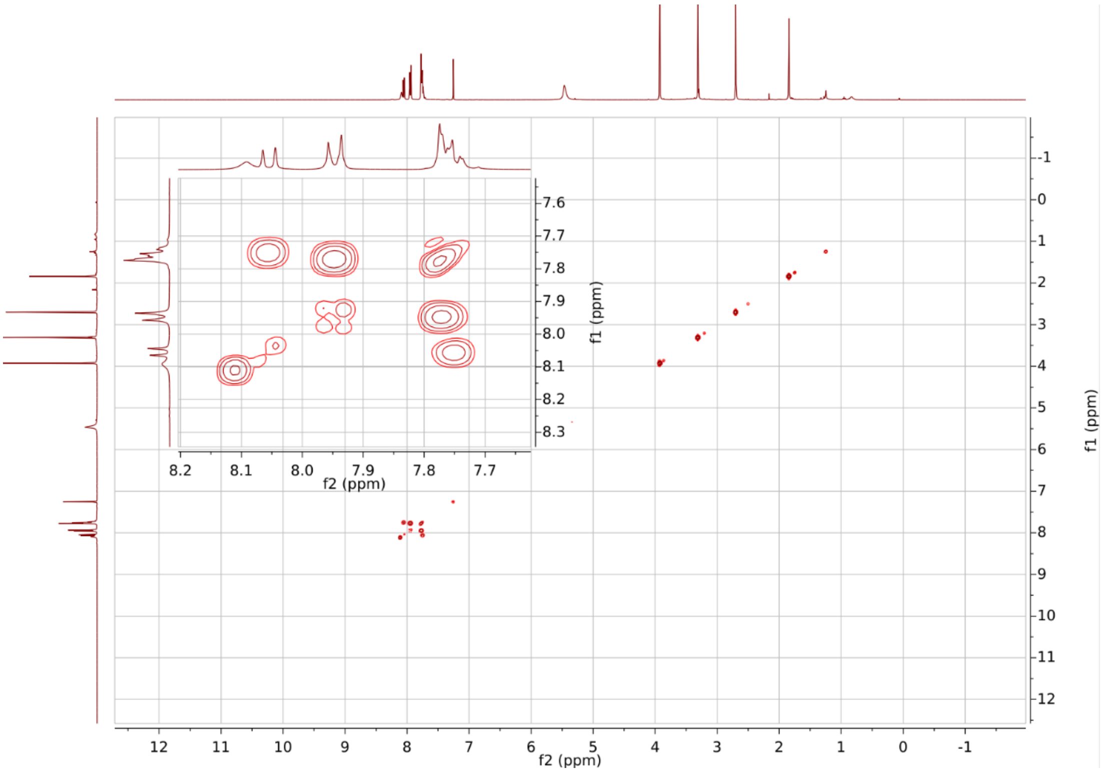

Compound S21c (^{13}C NMR, 101 MHz, $CDCl_3$)

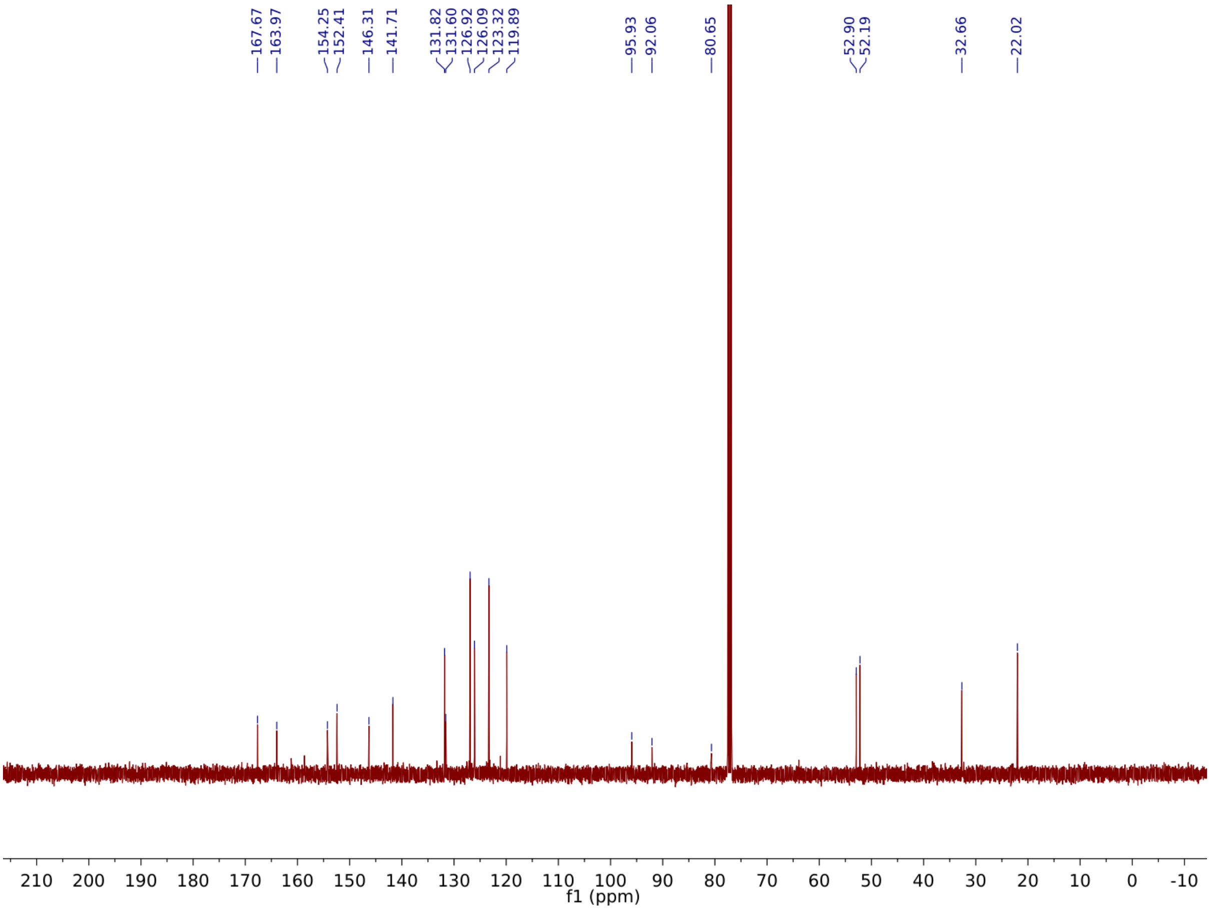

Compound 12 (^{1}H NMR, 400 MHz, DMSO-d_6)

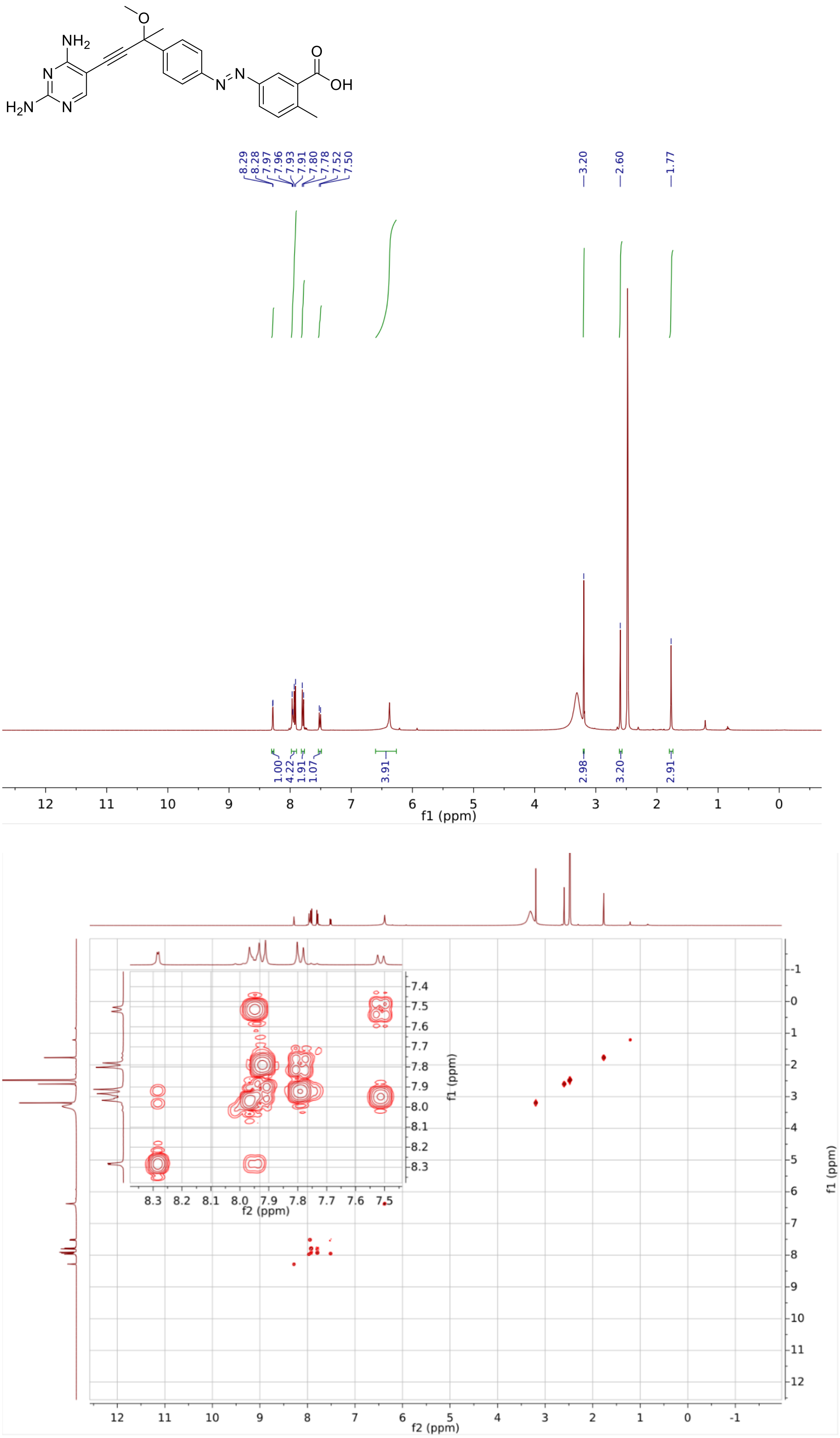

Compound 12 (^{13}C NMR, 101 MHz, DMSO-d_6)

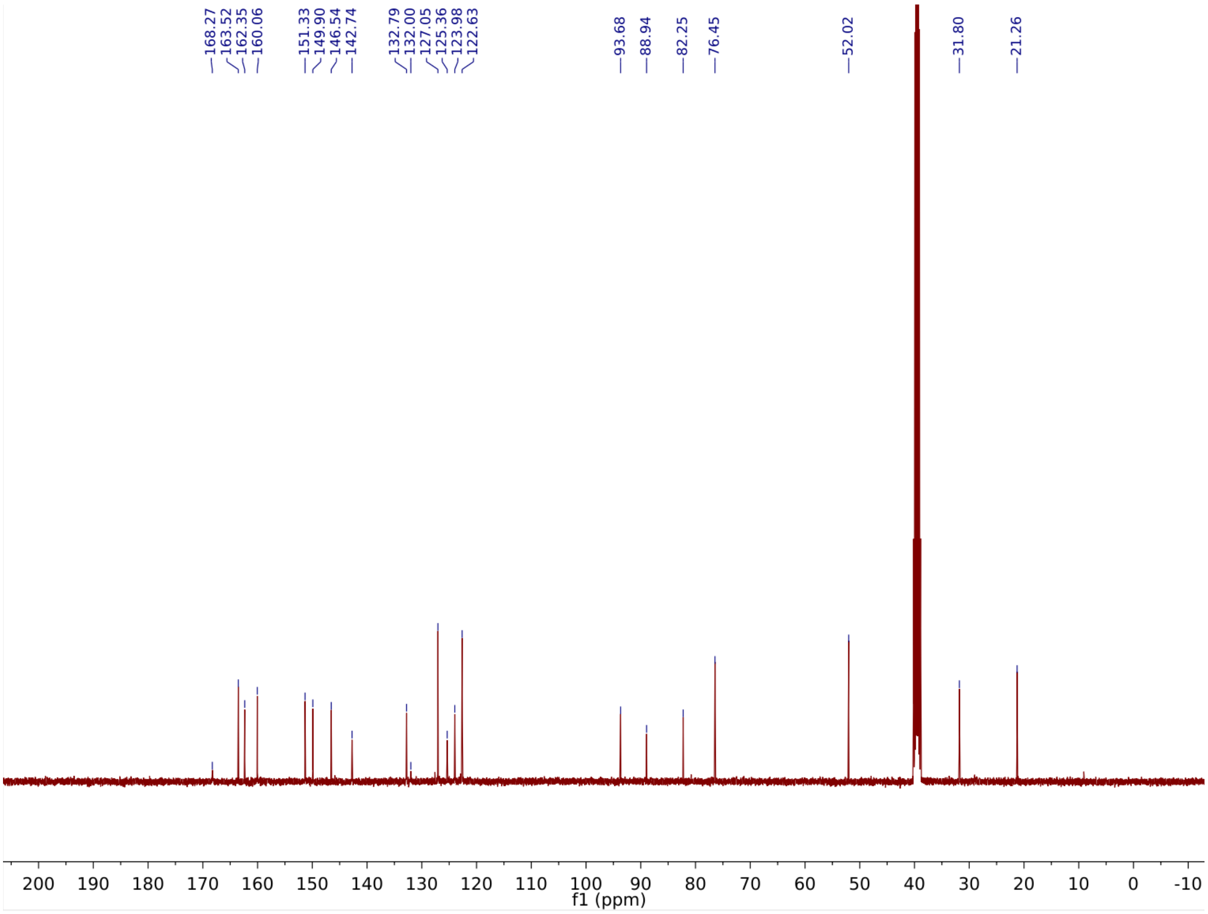

Compound 12 (HRMS)

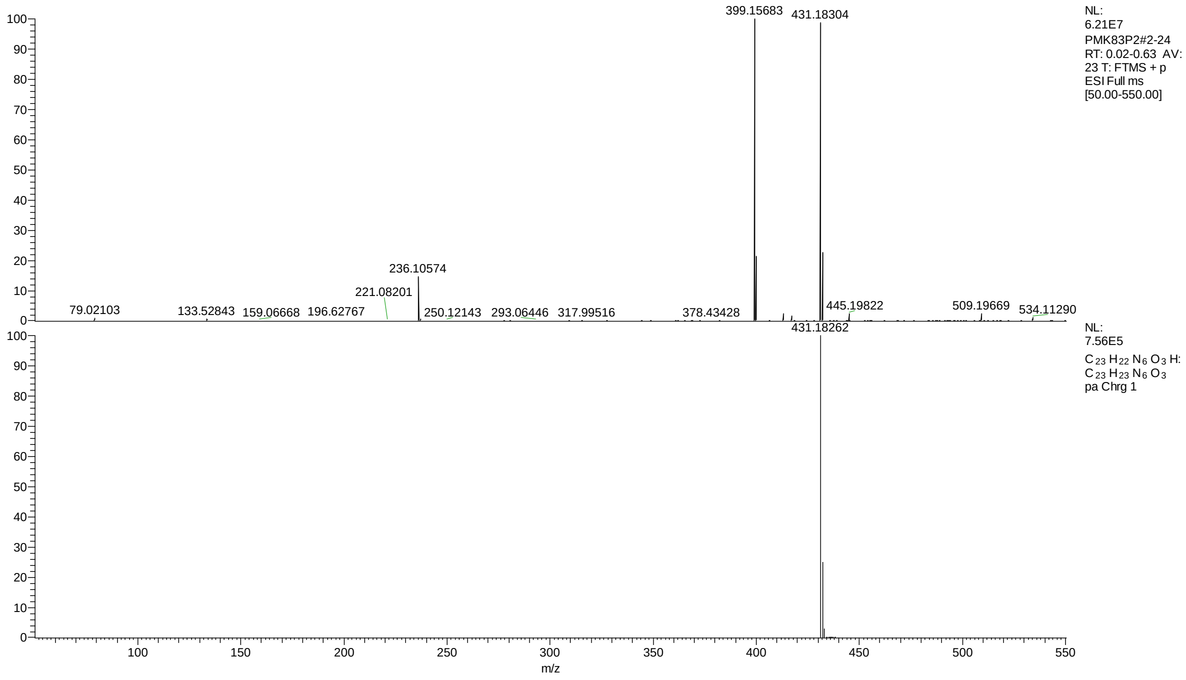

Compound 13 (^{1}H NMR, 400 MHz, DMSO-d_6)

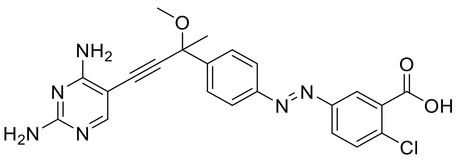

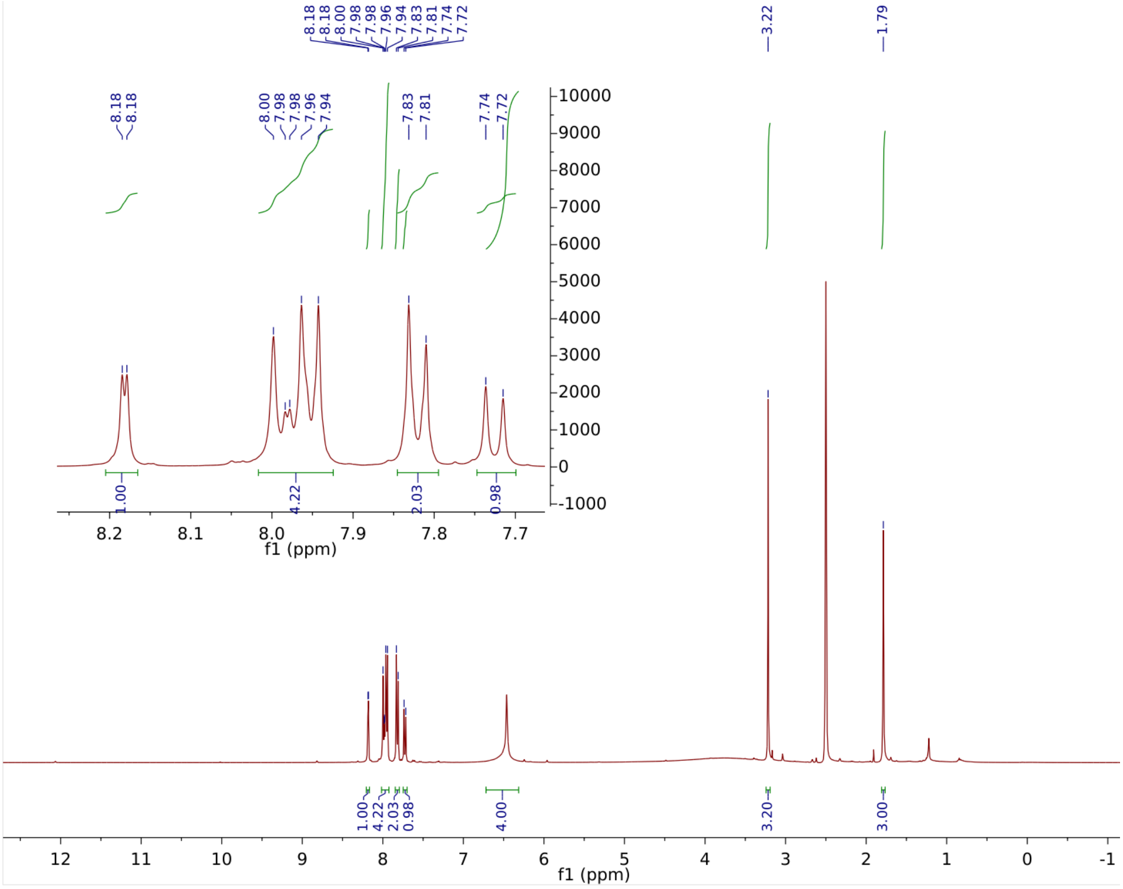

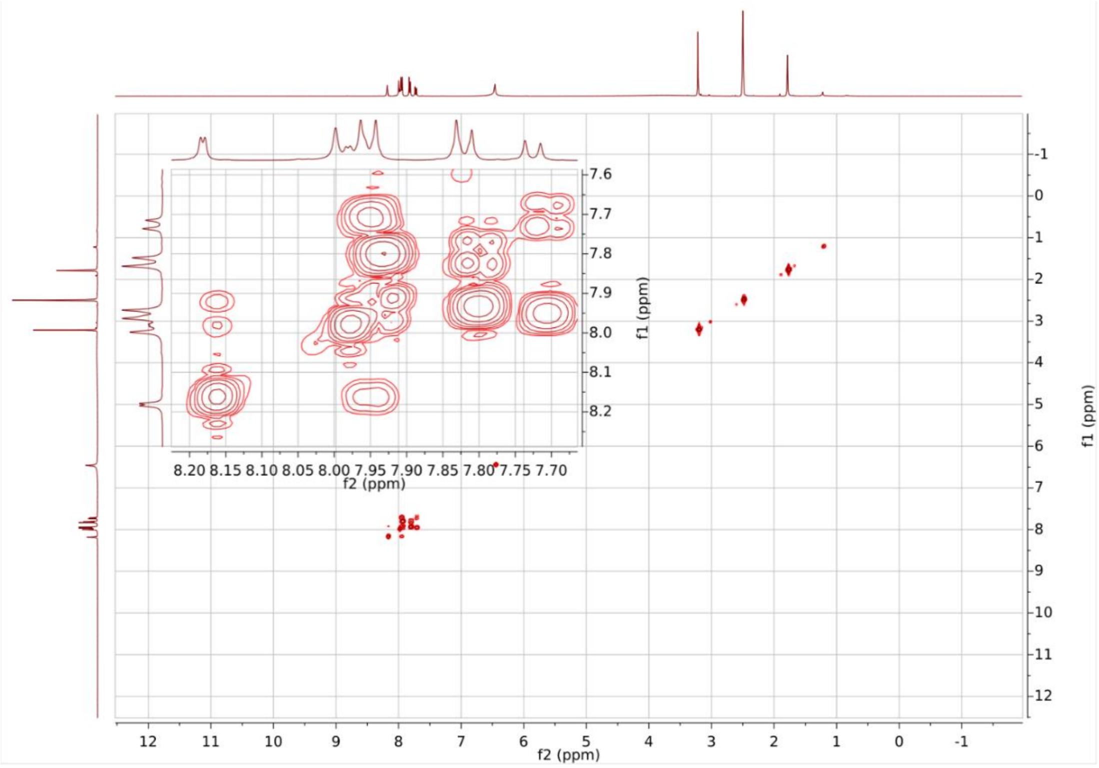

Compound 13 (^{13}C NMR, 101 MHz, DMSO-d_6)

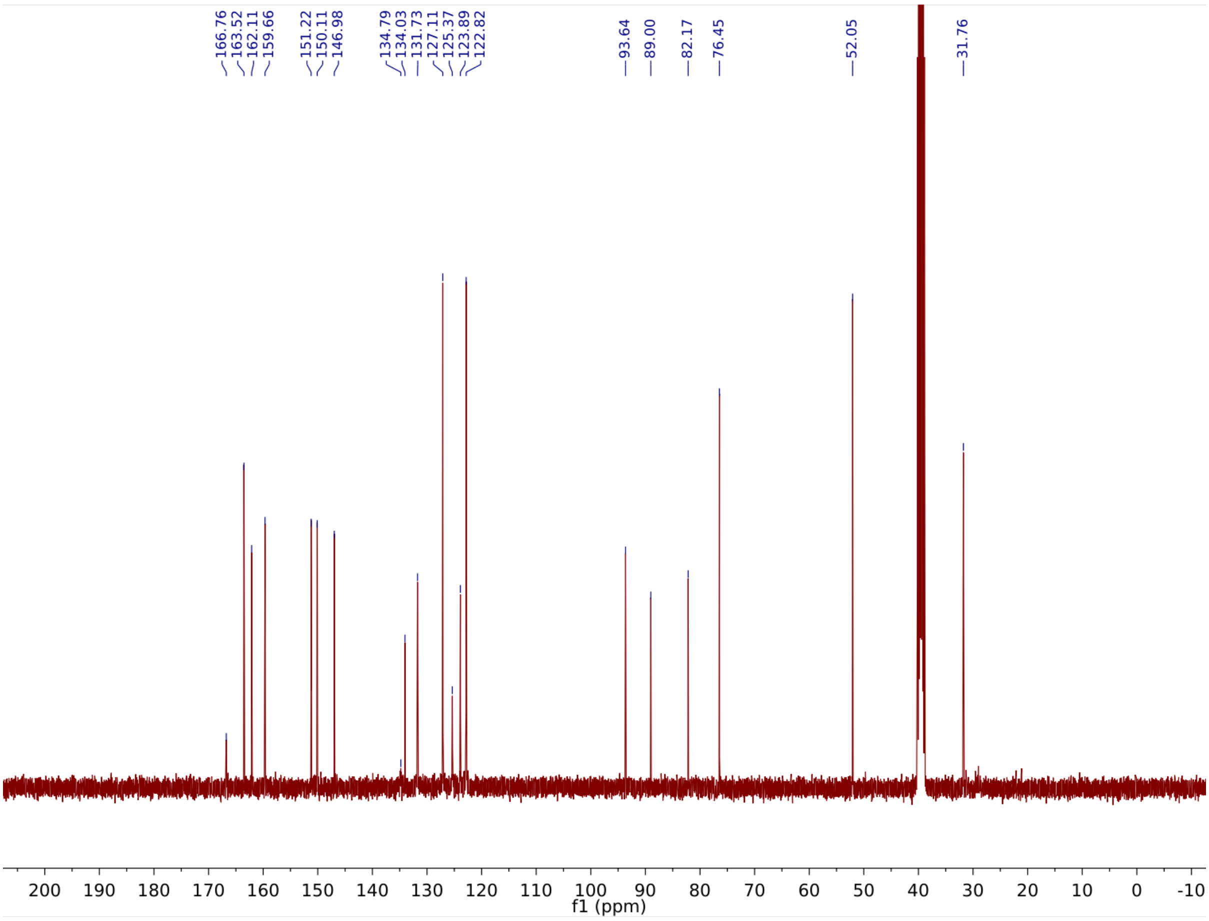

Compound 13 (HRMS)

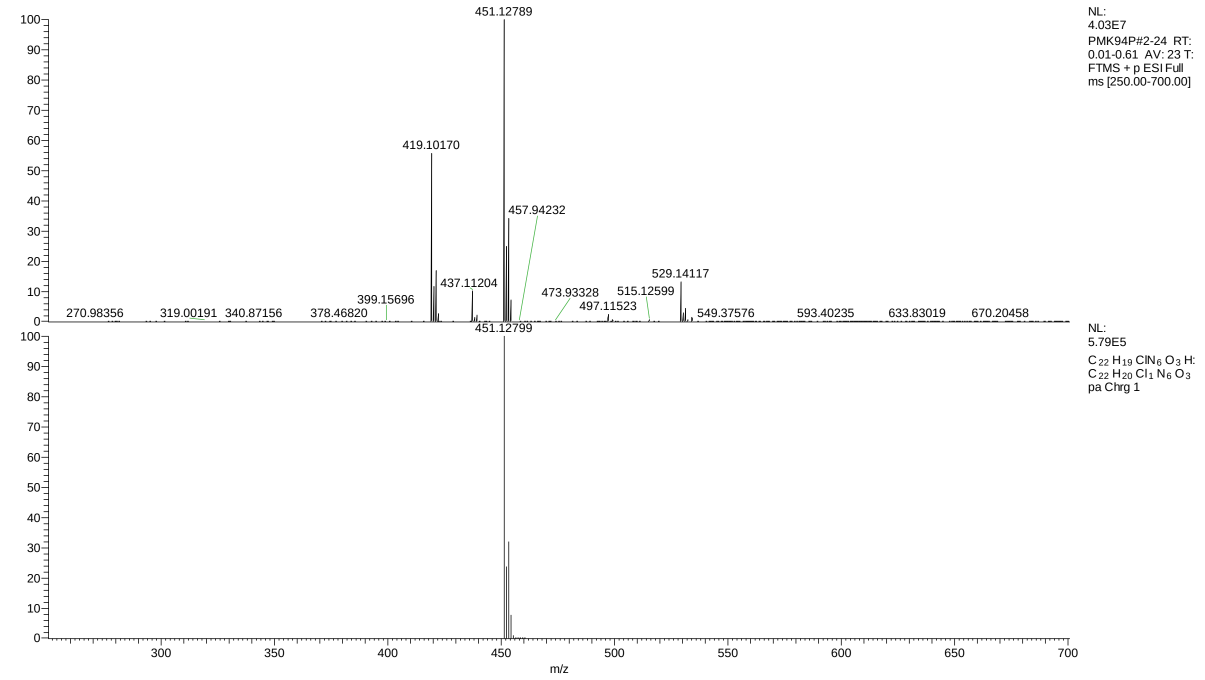

Compound 14 (^{1}H NMR, 400 MHz, DMSO-d_6)

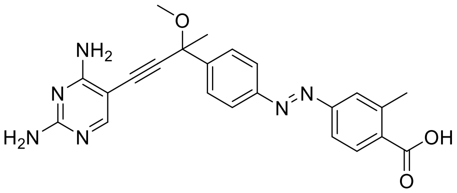

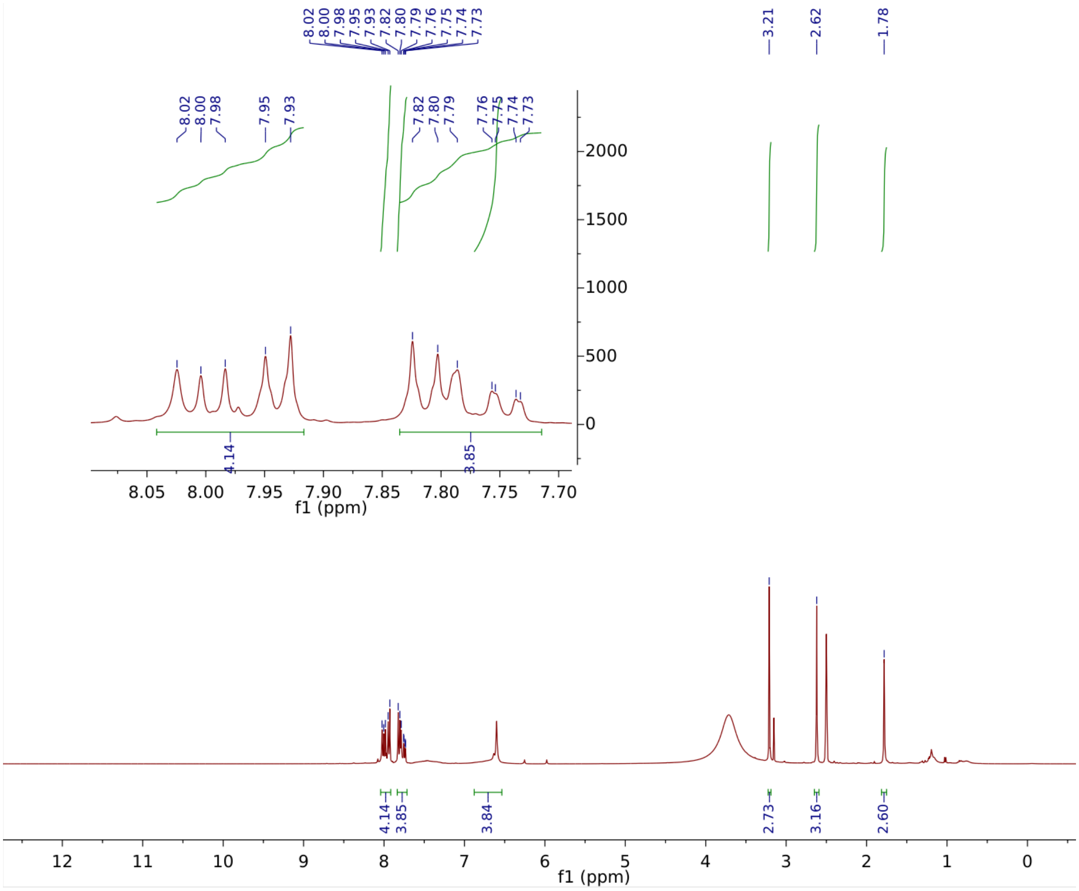

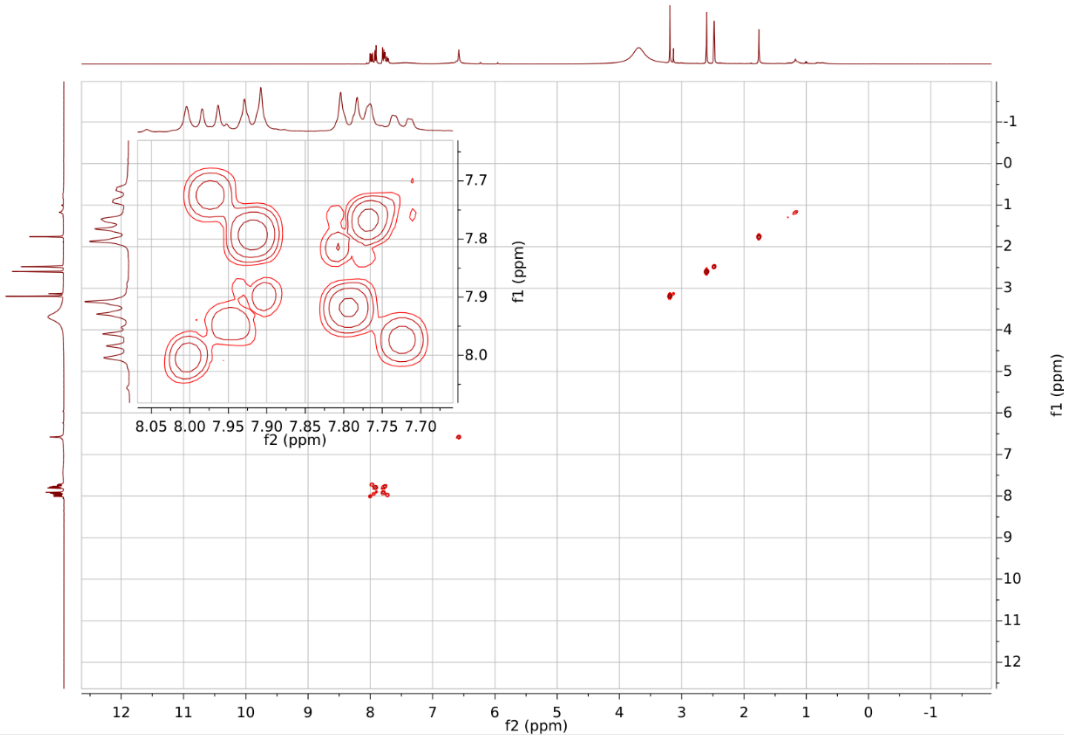

Compound 14 (^{13}C NMR, 101 MHz, DMSO-d_6)

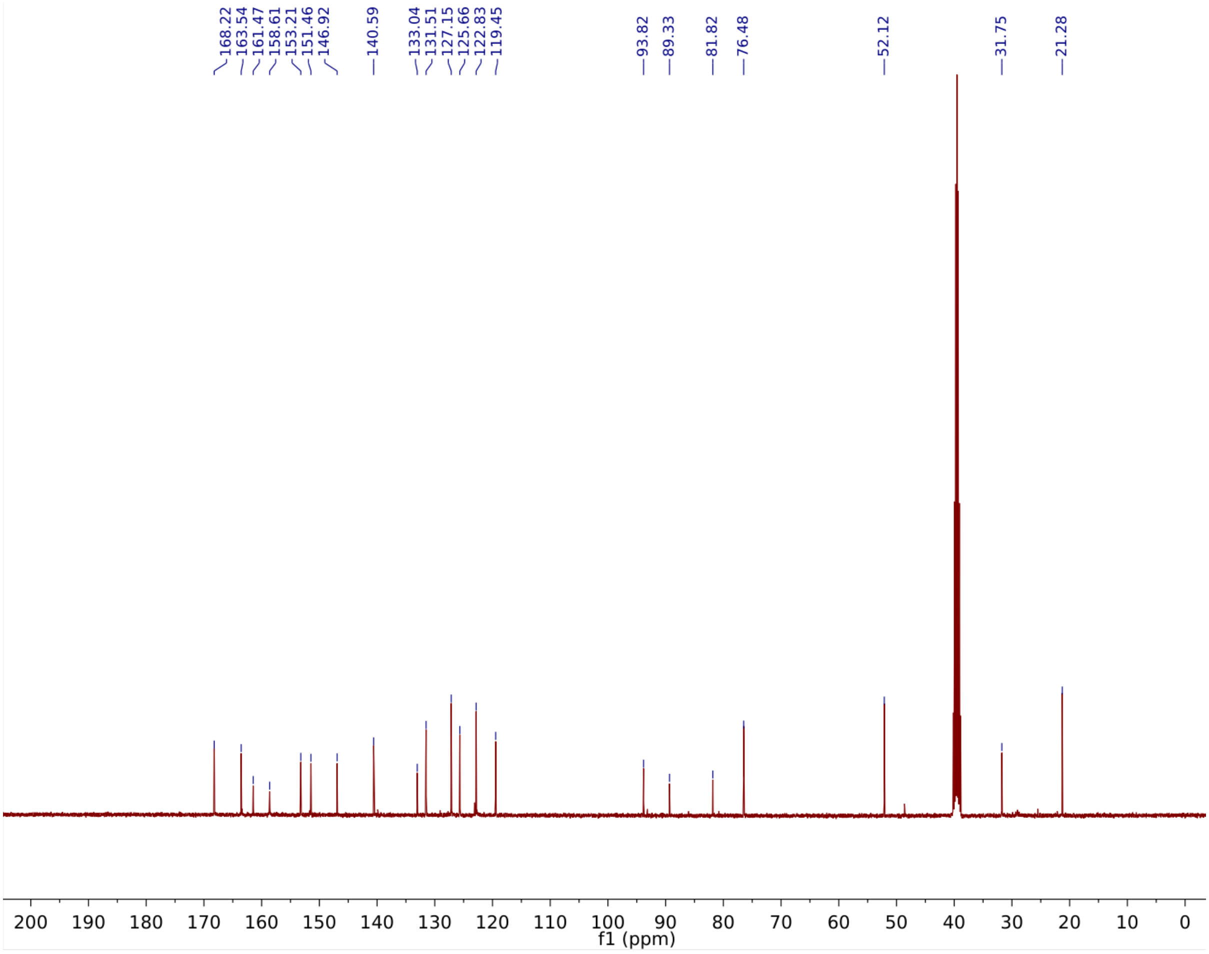

Compound 14 (HRMS)

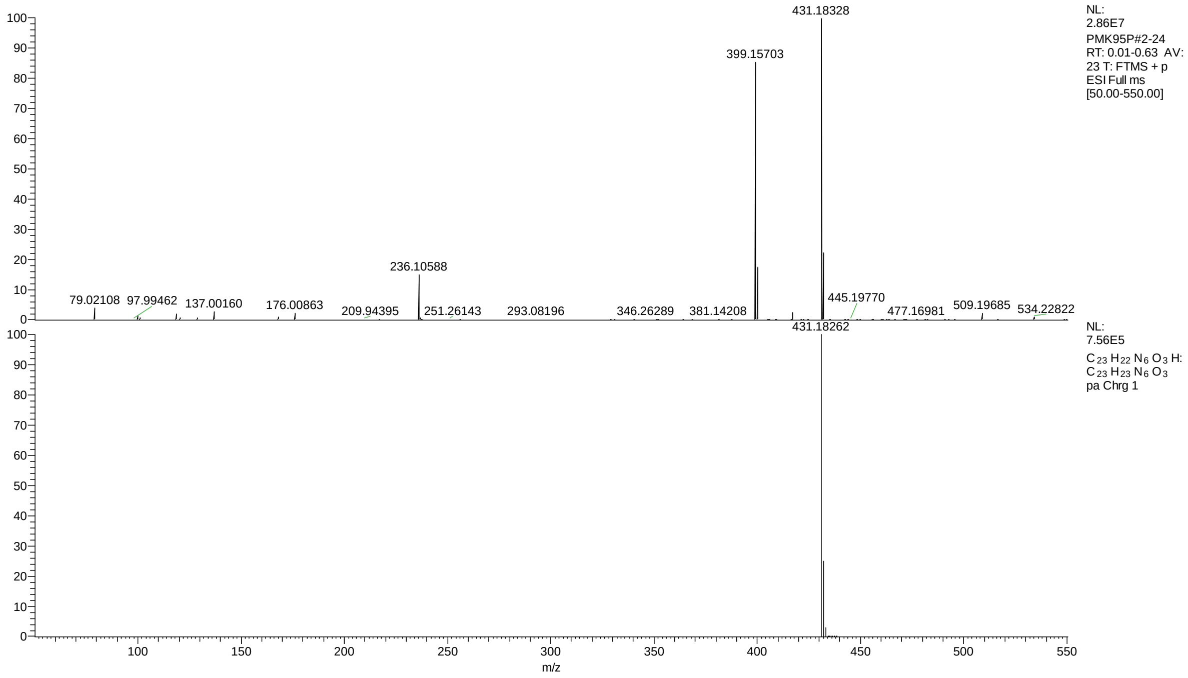

Compound 15 (^{1}H NMR, 400 MHz, DMSO-d_6)

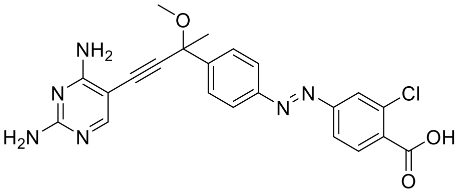

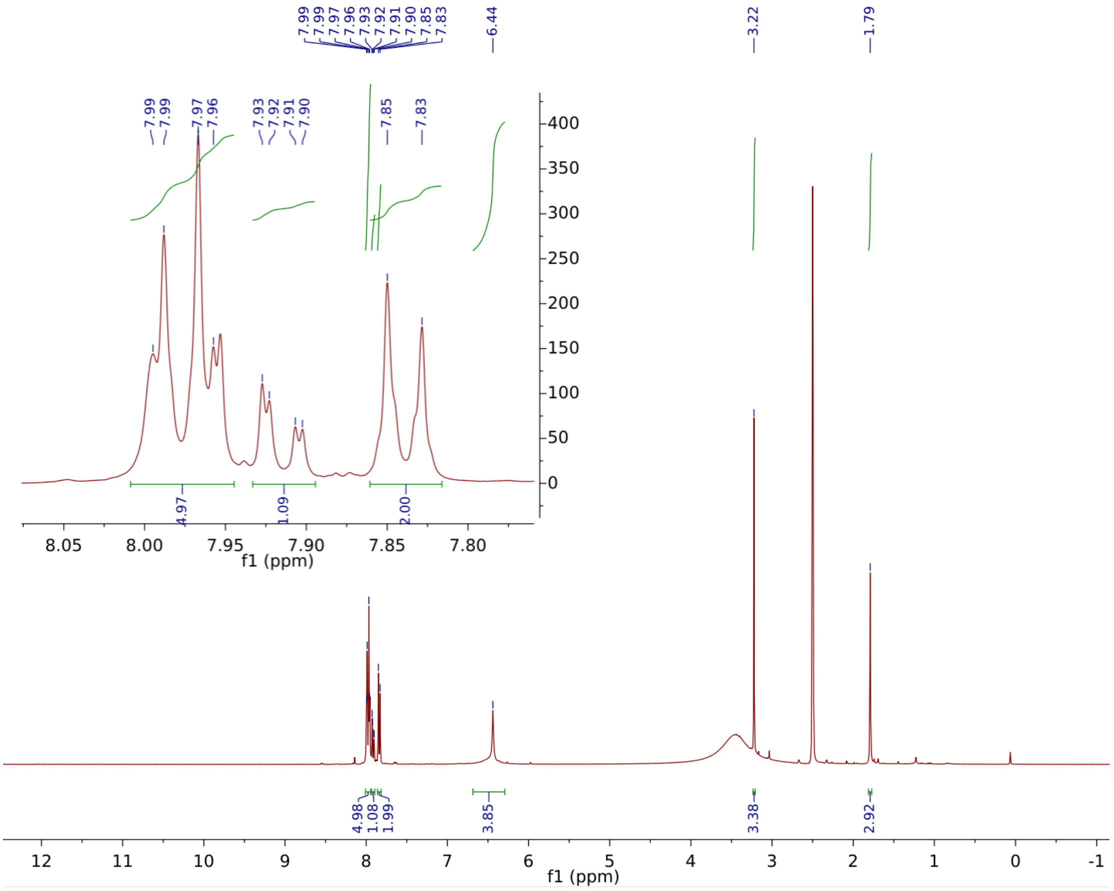

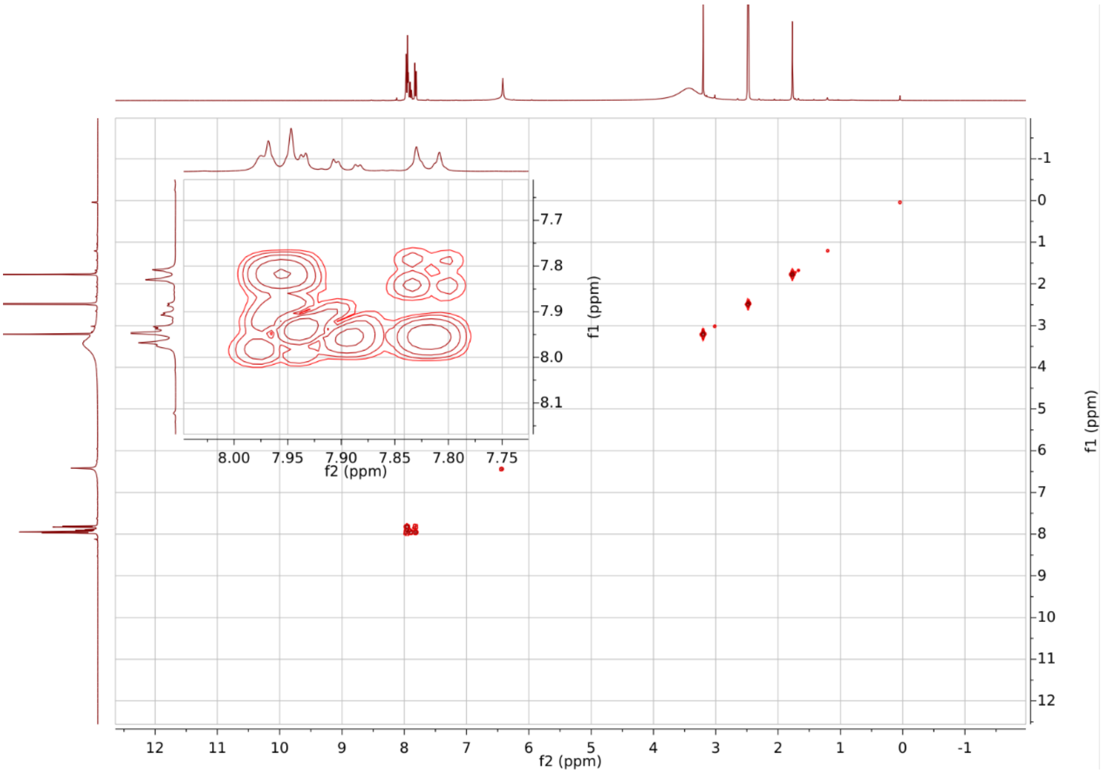

Compound 15 (^{13}C NMR, 101 MHz, DMSO-d_6)

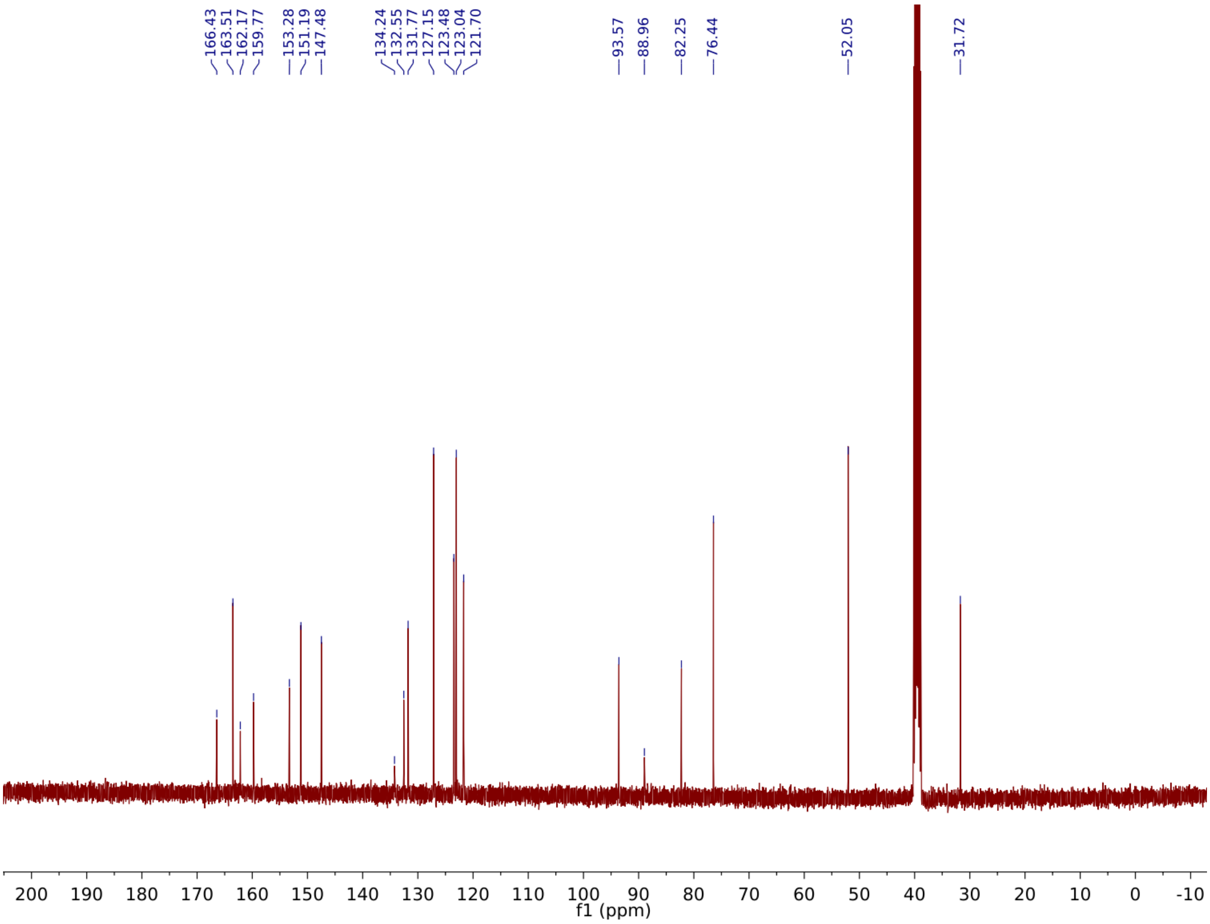

Compound 15 (HRMS)

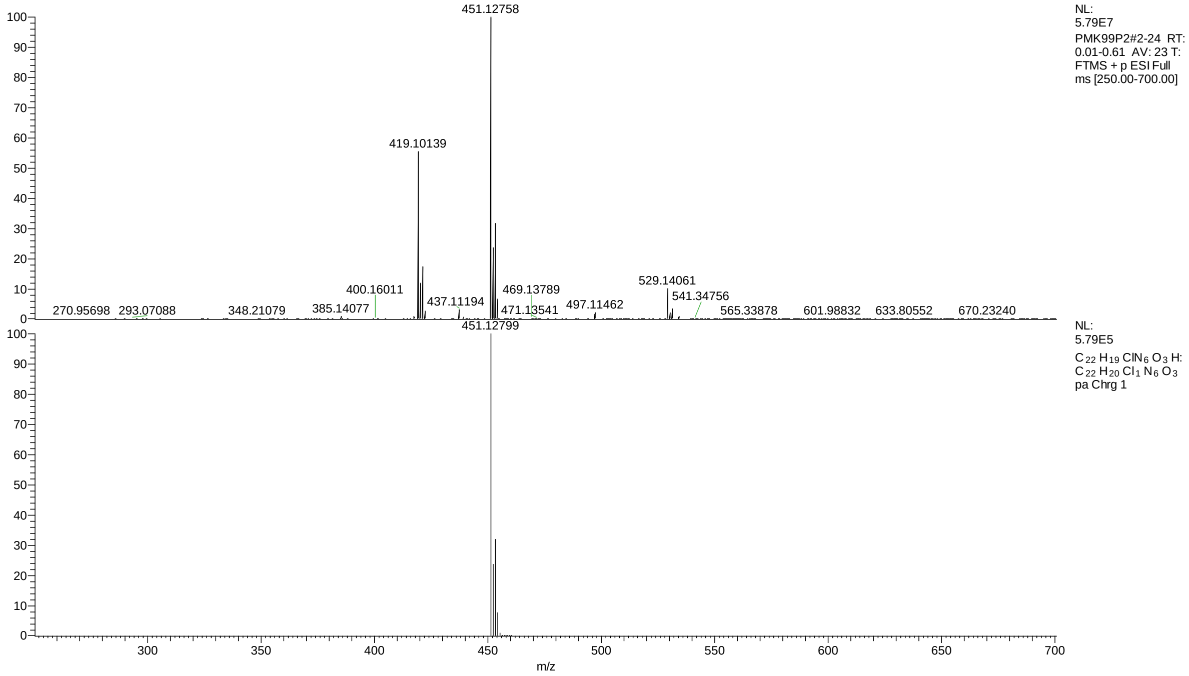

S8. References

(1) Pelphrey, P. M.; Popov, V. M.; Joska, T. M.; Beierlein, J. M.; Bolstad, E. S. D.; Fillingham, Y. A.; Wright, D. L.; Anderson, A. C. Highly Efficient Ligands for Dihydrofolate Reductase from Cryptosporidium Hominis and Toxoplasma Gondii Inspired by Structural Analysis. *J. Med. Chem.* **2007**, *50* (5), 940–950.

(2) Erb, W.; Hellal, A.; Albini, M.; Rouden, J.; Blanchet, J. An Easy Route to (Hetero)Arylboronic Acids. *Chem. - A Eur. J.* **2014**, *20* (22), 6608–6612.

(3) Lavastre, O.; Ollivier, L.; Dixneuf, P. H.; Sibandhit, S. Sequential Catalytic Synthesis of Rod-like Conjugated Poly-Ynes. *Tetrahedron* **1996**, *52* (15), 5495–5504.

(4) Xu, C.; Du, W.; Zeng, Y.; Dai, B.; Guo, H. Reactivity Switch Enabled by Counterion: Highly Chemoselective Dimerization and Hydration of Terminal Alkynes. *Org. Lett.* **2014**, *16* (3), 948–951.

(5) Lavastre, O.; Cabioch, S.; Dixneuf, P. H.; Vohlidal, J. Selective and Efficient Access to Ortho, Meta and Para Ring-Substituted Phenylacetylene Derivatives R-[C≡C-C6H4](x)-Y (Y:H, NO2, CN, I, NH2). *Tetrahedron* **1997**, *53* (22), 7595–7604.

(6) Iwakura, M.; Jones, B. E.; Luo, J.; Matthews, C. R. A Strategy for Testing the Suitability of Cysteine Replacements in Dihydrofolate Reductase from Escherichia Coli. *J. Biochem.* **1995**, *117* (3), 480–488.

(7) Wegener, M.; Hansen, M. J.; Driessen, A. J. M.; Szymanski, W.; Feringa, B. L. Photocontrol of Antibacterial Activity: Shifting from UV to Red Light Activation. *J. Am. Chem. Soc.* **2017**, *139* (49), 17979–17986.

(8) Ebejer, J. P.; Charlton, M. H.; Finn, P. W. Are the Physicochemical Properties of Antibacterial Compounds Really Different from Other Drugs? *J. Cheminform.* **2016**, *8* (1), 1–9.
